# LTRtype, an Efficient Tool to Characterize Structurally Complex LTR Retrotransposons and Nested Insertions on Genomes

**DOI:** 10.3389/fpls.2017.00402

**Published:** 2017-04-04

**Authors:** Fan-Chun Zeng, You-Jie Zhao, Que-Jie Zhang, Li-Zhi Gao

**Affiliations:** ^1^Institution of Genomics and Bioinformatics, South China Agricultural UniversityGuangzhou, China; ^2^Plant Germplasm and Genomics Center, Kunming Institute of Botany, Chinese Academy of SciencesKunming, China; ^3^Agro-biological Gene Research Center, Guangdong Academy of Agricultural SciencesGuangzhou, China

**Keywords:** LTRtype, LTR retrotransposons, nested insertions, structural complexity, genome evolution

## Abstract

The amplification and recombination of long terminal repeat (LTR) retrotransposons have proven to determine the size, organization, function, and evolution of most host genomes, especially very large plant genomes. However, the limitation of tools for an efficient discovery of structural complexity of LTR retrotransposons and the nested insertions is a great challenge to confront ever-growing amount of genomic sequences for many organisms. Here we developed a novel software, called as LTRtype, to characterize different types of structurally complex LTR retrotransposon elements as well as nested events. This system is capable of rapidly scanning large-scale genomic sequences and appropriately characterizing the five complex types of LTR retrotransposon elements. After testing on the *Arabidopsis thaliana* genome, we found that this program is able to properly annotate a large number of structurally complex elements as well as the nested insertions. Thus, LTRtype can be employed as an automatic and efficient tool that will help to reconstruct the evolutionary history of LTR retrotransposons and better understand the evolution of host genomes. LTRtype is publicly available at: http://www.plantkingdomgdb.com/LTRtype/index.html.

## Introduction

It has long been recognized that transposable elements compose an important fraction of most eukaryote genomes. Transposable elements are usually classified into three groups by transposition mechanisms, known as LTR retrotransposons, non-LTR retrotransposons, and DNA transposons. Among them, LTR retrotransposons are particularly prevalent in most plant genomes, where they appear to be the major determinant of the tremendous variation in genome size (Wessler, 2006; Wicker et al., 2007; Wendel et al., 2016). They have been found to serve as a major contributor to large plant genomes (Flavell, 1986; SanMiguel et al., 1998; Kumar and Bennetzen, 1999; Meyers et al., 2001; Nystedt et al., 2013). LTR retrotransposons are a class of mobile genetic elements containing two identical or similar long terminal repeats (LTRs) and one internal region (IN) between them, which are transposed through the reverse transcription of an RNA template via “copy-and-paste” in the genome. For decades it has been recognized that evolutionary dynamics of these elements actively act as a motivating force to drive the genome evolution. LTR retrotransposons can largely make contributions to the variation of genome size through the amplification and recombination, and likewise bring about genomic structural variation and organization. For instance, LTR retrotransposons determine the growth of genome size through retrotransposon amplification (SanMiguel et al., 1998; Bennetzen, 2002; Kellogg and Bennetzen, 2004), and they also cause genome size reduction through unequal homologous recombination and illegitimate recombination (Devos et al., 2002; Vitte and Panaud, 2003; Ma et al., 2004; Bennetzen et al., 2005). Taking the *Arabidopsis thaliana* genome for example, rapid loss was meanwhile revealed to counteract genome expansion through recombination despite a large number of recent amplification of LTR retrotransposons (Devos et al., 2002).

Extraordinary quantities of TEs, particularly the LTR retrotransposons, have greatly hampered the genome assembly and annotation. Thus far, a multitude of computational tools have been developed for the detection of LTR retrotransposons in the rapidly emerging genomic sequences. Common structural features of LTR retrotransposon elements make it possible to *de novo* detect novel LTR retrotransposon families having low sequence homology to known queries or families with a typical structure. With this regard, several programs were specifically designed for the *ab initio* computer discovery of LTR retrotransposons. LTR_STRUC (McCarthy and McDonald, 2003) is the best known of these programs, which has broadly been applied to numerous genomes, such as the *Glycine max* (Schmutz et al., 2010), *Mus musculus* (McCarthy and McDonald, 2004), *Oryza sativa* (Tian et al., 2009), and *Pan troglodytes* (Polavarapu et al., 2006). Furthermore, the other programs, including LTR_par (Kalyanaraman and Aluru, 2006), LTR_Rho (Rho et al., 2007), LTR_FINDER (Xu and Wang, 2007), LTR_harvest (Ellinghaus et al., 2008), and LTRdigest (Steinbiss et al., 2009), were developed for the *de novo* prediction of LTRs, and these programs consider further features of LTR retrotransposons in post processing steps to enhance the quality or sensitivity of the predictions. Most extensively implemented approaches of LTR retrotransposon identification were based on similarity searches against a target genome. These tools, such as REPuter (Kurtz et al., 2001), RECON (Bao and Eddy, 2002), RAP (Campagna et al., 2005), PILER (Edgar and Myers, 2005), RepeatMasker^1^ and LTR Annotator (You et al., 2015), are able to detect repeat sequences in the genome, but they incorporated almost no defragmentation and have definitely resulted in the overestimated number of LTR retrotransposons. Limitations of such sort of approaches are apparent, since they are able to find elements in the database without difficulty but they may scarcely predict repeat elements with distantly divergent sequences. All the above-mentioned annotation tools may limitedly apply to detecting full-length elements (LTR-IN-LTR) and their fragmented elements, but they are unable to reconstruct structurally complex elements as well as their nested cluster.

Although TE-nest (Kronmiller and Wise, 2008, 2013) and REannotate (Pereira, 2008) can identify nested events of transposable elements, they fail to adequately locate the structurally complex LTR retrotransposons. Until now, little has been known regarding genome-wide patterns of structurally complex retrotransposons and their contributions to the modification of genomes. The difficulty increases without a doubt to efficiently annotate such elements due to the recombination, DNA losses and nested events in a genome. Single Molecule Real Time (SMRT) Sequencing technologies are able to generate long reads that are essential to complete plant reference genomes that will provide unprecedented opportunities to obtain in-depth knowledge on evolutionary behaviors of retrotransposons and explore their contributions to the genome structure, function and evolution on the strength of large-scale genome information. Thus, it is urgently needed to develop efficient tools to genome-wide characterize various types of structurally complex retrotransposon elements and better annotate genomes in rapidly deposited large-scale genomic sequences.

**FIGURE 1:**
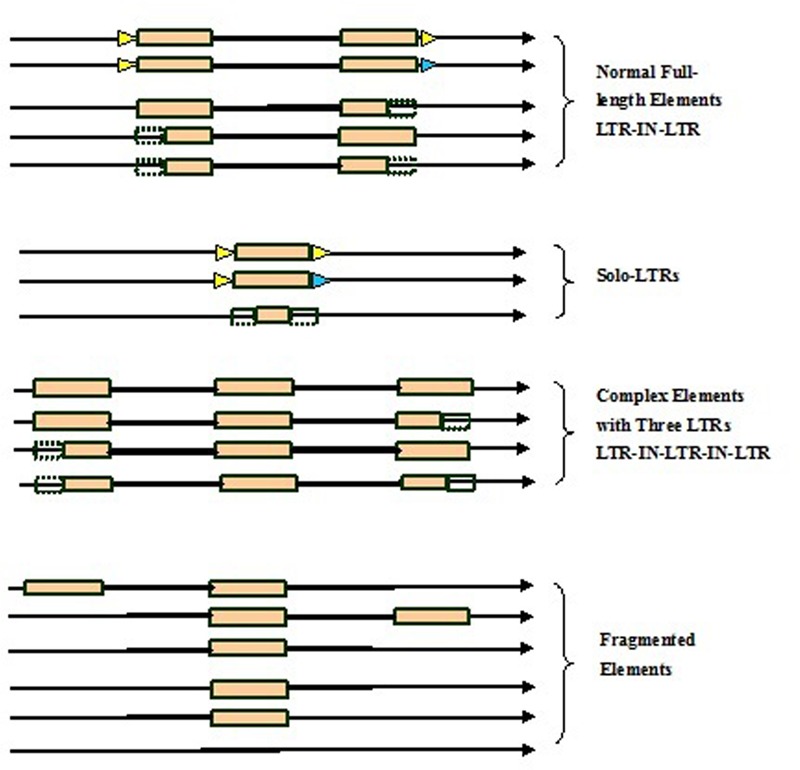
**Structurally diverse types of long terminal repeat (LTR) retrotransposons identified in the genome.** Triangles in blue and yellow indicate the two different target site duplications (TSDs), rectangles in brown represent LTRs, dotted line rectangles represent fragmented LTRs, black thick lines denote internal regions, and arrows show the direction of DNA strand.

**FIGURE 2:**
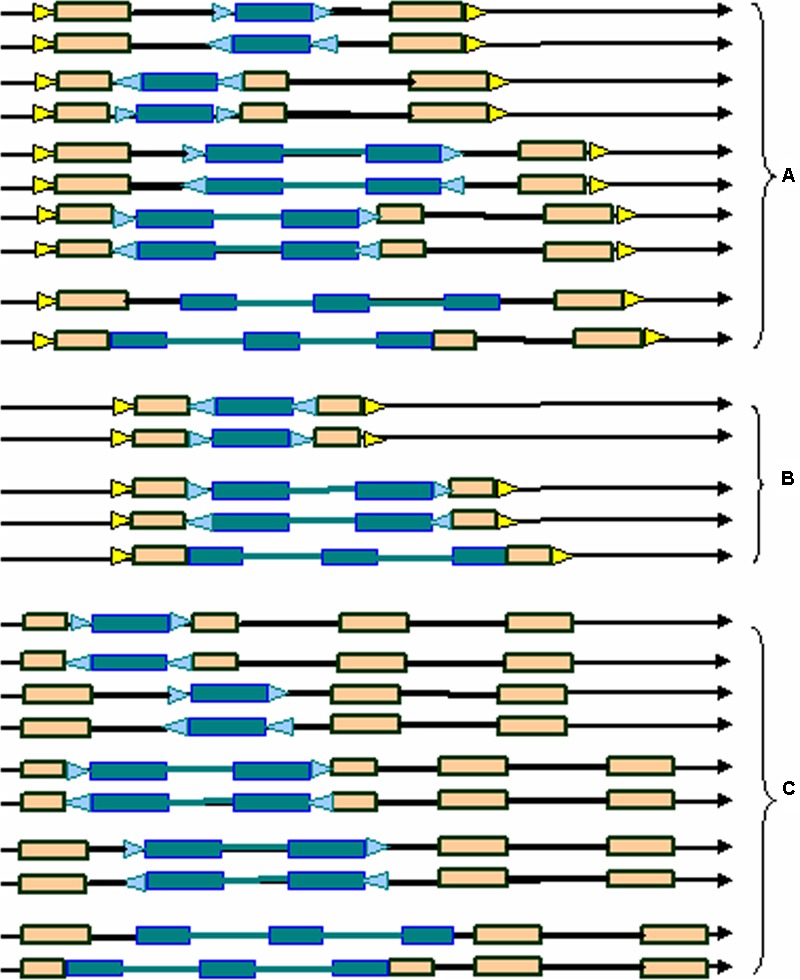
**Typical nested structural types of LTR retrotransposons in the genome. (A)** normal full-length elements nested by solo-LTRs, normal full-length elements, or complex elements with three LTRs; **(B)** solo-LTR elements nested by solo-LTRs, normal full-length elements, or complex elements with three LTRs; **(C)** complex elements with three LTRs nested by solo-LTRs, normal full-length elements, or complex elements with three LTRs. Triangles in blue and yellow indicate the two different TSDs, rectangles in brown represent LTRs, dotted line rectangles represent fragmented LTRs, black thick lines denote internal regions, and arrows show the direction of DNA strand.

Here we developed a novel software program, named LTRtype, for the purpose of proficient discovery of diverse types of the structurally complex LTR retrotransposons (**Figure 1**) and nested insertions (**Figure 2**) in large quantities of genomic sequences. In addition to an effective solution of fragmented retrotransposon sequences from BLAST searches, this program has incorporated a combination of rapid algorithms, which may not limit to LTR pairs but indeed is able to identify retrotransposon elements with three or more LTRs. These elements include: (1) normal full-length elements (LTR-IN-LTR); (2) solo-LTR elements; (3) complex elements with three or more LTRs (e.g., LTR-IN-LTR-IN-LTR); (4) fragmented elements of the above-mentioned structural types; and (5) nested insertions among these different LTR retrotransposon elements. The program LTRtype reported here is able to proficiently mine increasingly sequenced genomes by using multithreading technologies, and thus provides a convenient and friendly service for users.

## Materials and Methods

### Flowchart of LTRtype

**FIGURE 3:**
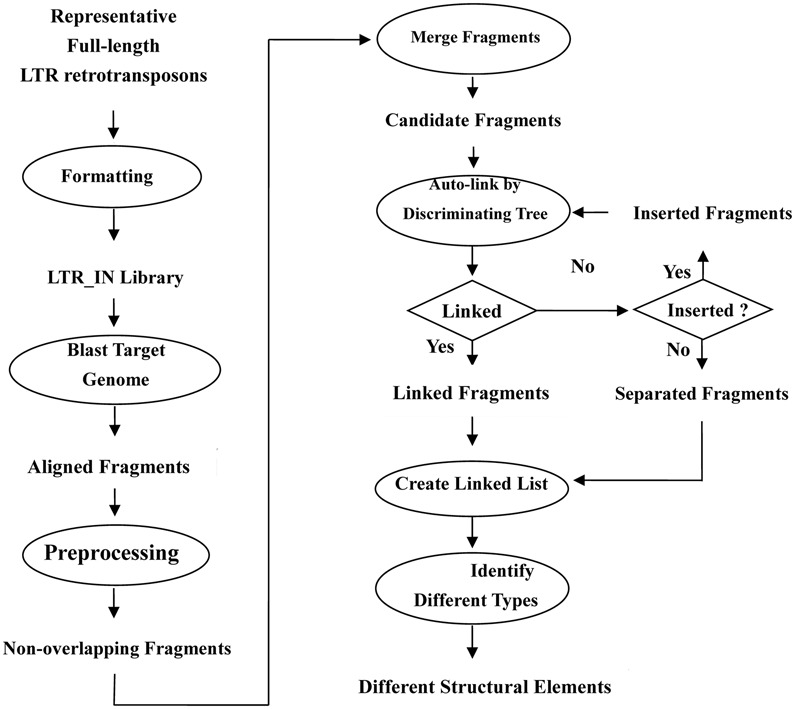
**Flowchart of the characterization of LTR retrotransposon elements in the program LTRtype**.

The first step of LTRtype in the flowchart (as shown in **Figure 3**) is to collect all full-length LTR retrotransposons and construct library files in FASTA format. There are two major methods to retrieve the full-length LTR retrotransposons. One is to straightforwardly download from the known repeat databases, e.g., Repbase: http://www.girinst.org/; PlantGDB: http://www.plantgdb.org; Plant repeats: http://www.tigr.org/tdb/e2k1/plant.repeats/, while the other is to mine target genomes by using authorized tools of LTR retrotransposons, such as LTR_STRUC (McCarthy and McDonald, 2003), LTR_FINDER (Xu and Wang, 2007), LTR_harvest (Ellinghaus et al., 2008), and so on. Then, the library will be merged by their overlapping lengths and sequence similarities.

The second step is to align the LTR and IN libraries with target genomes using RepeatMasker and preprocess the aligned fragments by eliminating the overlapped elements.

The third step is to merge adjacent fragments into longer or more intact LTR or IN sequences that belong to the same family, as RepeatMasker usually align a complete LTR or IN fragment into several fragments that may be separately or partially overlapped. The yielded datasets become candidate fragments for further analyses.

The fourth step is to link candidate fragments by discriminating trees according to their physical positions. If something can be connected one another, then we construct the linked fragments and take else as inserted elements or unconnected fragments. Note that inserted elements should be linked again until nothing could be connected.

The last step is to create a linked list and categorize different structural types of LTR retrotransposon elements.

### Auto-Link by Discriminating Tree

**FIGURE 4:**
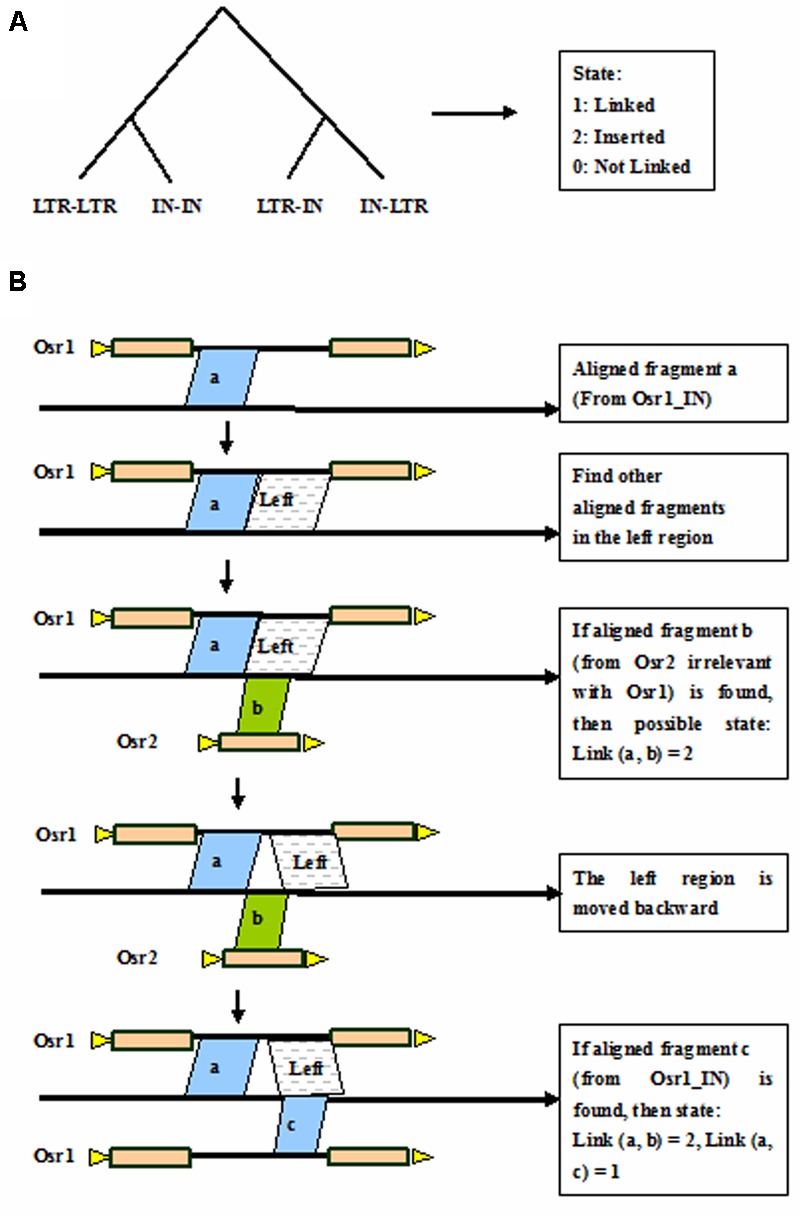
**Auto-link by discriminating tree. (A)** Discriminating tree; **(B)** Link the aligned fragments by taking the linked IN-IN for example. Triangles in yellow indicate TSDs, rectangles in brown represent LTRs, and black thick lines denote internal regions.

Discriminating tree is a method to discriminate whether the aligned fragments should be connected that belong to the same LTR retrotransposon element in the genome. The discriminating tree is classified into the four branches: LTR-LTR, IN-IN, LTR-IN, and IN-LTR (**Figure 4A**). There are three output states of the discriminating tree, that is, 1 is linked, 0 is not linked, and 2 stands for inserted. Taking the linked IN-IN for example (**Figure 4B**), the three separated fragments are aligned to adjacent coordinates in the rice genome. The fragment a with coordinates of 1,001–1,500 in the genome corresponds to coordinates of 1–500 in the Osr1_IN, the fragment b with coordinates of 1,501–1,800 corresponds to coordinates of 1–300 in the Osr2_LTR, and the fragment c with coordinates of 1,801–2,300 corresponds to coordinates of 501–1,000 in the Osr1_IN. Here, the known length of Osr1_IN is 1,000 bp. Then the state of link (a, b) = 2, and link (a, c) = 1, that is, the fragments a and c is connected as a single element, and fragment b is regarded as an inserted element.

### Create the Dynamic Link List and Identify Different Structural Elements

**FIGURE 5:**
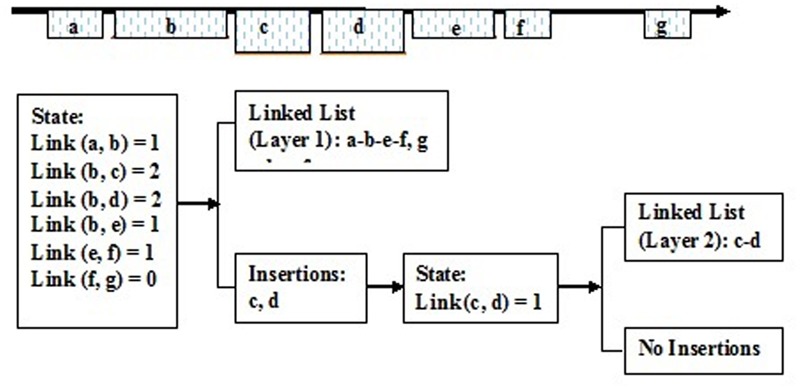
**The creation of the dynamic linked list.** a, b, c, d, e, f, and g Show the aligned fragments of LTR retrotransposons.

It is feasible to create a series of dynamic link lists according to the link states of the aligned fragments. Here, a dynamic link list stands for one linked block domain in the genome, and constructs one structural type of LTR retrotransposon element. There are seven adjacent aligned fragments in the genome, and these link states are individually given as follows: link (a, b) = 1, link (b, c) = 2, link (b, d) = 2, link (b, e) = 1, link (c, d) = 1, link (d, e) = 0, link (e, f) = 1, and link (f, g) = 0. Then, they may construct three dynamic link lists: (a-b-e-f), (c-d), (g), and form three LTR retrotransposon elements (**Figure 5**).

### Comparisons with LTRtype, REannotate, and TEnest

We downloaded the sequences of maize LTR retrotransposons from Repbase version 18.11 (Jurka et al., 2005), and built the library file (Supplementary Library S1), including a total of 302 full-length elements; they were further subdivided into two sequence libraries containing 302 pairs of LTRs and INs, respectively. Maize genome sequences of Chromosome 10 (gb: CM000786.2) were downloaded from NCBI. LTR retrotransposons of maize were mined by running LTRtype (default), REannotate (-n -t -d = 10k -s = 10k -c), and TEnest (default), respectively. Because default parameters are loose, we adjusted them when running REannotate.

### Mining and Analyzing LTR Retrotransposons in the *A. thaliana* Genome

The sequences of *A. thaliana* LTR retrotransposons were downloaded from Repbase version 18.11 to build the LTR retrotransposon library files including a total of 146 full-length elements (Supplementary Library S2). This library file thus contained 146 corresponding pairs of LTRs and IN, respectively. The *A. thaliana* genome sequences (TAIR10) were downloaded from http://www.arabidopsis.org/. We identified different types of structurally complex LTR retrotransposons and characterized their nested insertions LTR retrotransposon on the *A. thaliana* genome by running LTRtype (default).

Nucleotide sequence divergence among pairs of intra-element LTRs was used as a molecular clock, as they are identical at the time of insertion. The ages of full-length LTR retrotransposons were then determined by comparing their 5′ and 3′ LTRs (SanMiguel et al., 1998). MEGA5 (Tamura et al., 2011) was employed to calculate the number of transition and transversion mutations. Insertion dates were estimated using the Kimura two-parameter method (Kimura, 1980). The average mutation rate of 1.3 × 10^-8^ substitutions per synonymous site per year (Ma and Bennetzen, 2004) and 7 × 10^-9^ substitutions per synonymous site per year (Ossowski et al., 2010) were applied to estimate insertion times of the LTR retrotransposons in the *A. thaliana* genome. The time (*T*) since element insertion was estimated using the formula: *T* = *K*/2*r*.

## Results and Discussion

### Hardware Requirements

The hardware requirements vary with genome sizes. Both Intel and AMD x64 architectures are supported. Note that there are no specific requirements including CPU, memory and disk space to run the LTRtype. Taking rice genome (∼400 Mb) for an example, it is needed to have ∼500 Mb RAM, 4+ cores, and ∼1 Gb disk space.

### User Input

LTRtype provides some default parameter settings to make users convenient, but the four parameters (-P,-p -i, and -d) must be set by users themselves. -P follows the path of RepeatMasker; -p follows the path of blast searches; -i follows the LTR retrotransposon library file (FASTA format). Reference LTR names may not have the gap and ‘| ’, but they must be suffixed with the string ‘_LTR.’ Likewise, reference IN names must be suffixed with the string ‘_IN’; -d follows the genome sequence file (FASTA format). In order to facilitate the view output, the sequence names do not contain the gap and ‘|’.

Usage: perl LTRtype.pl [options]

Options:

–P <dir> The path of RepeatMasker program, required
–p <dir> The path of blast program, required
–i <file> LTR retrotransposon library file, FASTA format, required
–d <file> The genome file, fasta format, required
–o <file> Output file of RepeatMasker (default = RM.out)
–a <int> Number of CPUs to use (default = 6)
–D <int> Masks only those repeats < x percent diverged from consensus sequence (default 20)
–C <int> Sets cutoff score for masking repeats (default 600)
–L <int> Minimum sequence length after blast searches with query LTR retrotransposon sequences (default 100)
–s <int> Similar sequence length after blast searches > x percent of query LTR retrotransposon sequences (default 80)
–I <int> Minimum sequence identity after blast searches with query LTR retrotransposon sequences (default 80)
–h help

### Program Output

Running LTRtype generates output files that record the detailed information that is a step-by-step guide for users (Supplementary File S3). Users can read the results from each running step. Loopx directory is the most important output and records detailed dataset for each layer of LTR retrotransposons. For example, type.num.x provides copy number of all structural types of LTR retrotransposons; and type.all.x gives the physical positions and content information of all structural types.

### Comparison of LTRtype, REannotate, and TEnest

We tested the efficiency of LTRtype to characterize LTR retrotransposons by comparing with REannotate (Pereira, 2008) and TEnest (Kronmiller and Wise, 2013). The expected running times depend of course on the cpu speed/number of cores used for the analysis, but LTRtype only requires nearly half time compared to REannotate and TEnest. Considering that TEnest is a time-consuming program for the analysis of a large number of genome sequences (Kronmiller and Wise, 2013), we merely tested 2 Mb of genomic sequences from Chromosome 10 by running these three tools. We found that LTRtype captured more numbers of normal elements and solo-LTRs but fewer numbers of truncated and particularly nested events than TEnest (**Table 1**). The pattern holds true while comparing LTRtype with REannotate (**Table 1**); our results showed that LTRtype reported 438 copies, whereas REannotate identified up to 580 copies (**Table 1**), suggesting that LTRtype had a better solution of the defragmentation of LTR retrotransposon elements. Detailed analysis of structurally different types of LTR retrotransposons in 2 Mb maize genome sequence identified at least 79 elements, including 59 normal, 7 complex, and 13 truncated retrotransposon elements that all contained a LTR-IN-LTR structure (**Table 2**). We further analyzed the Chromosome 10 of maize by comparing REannotate and LTRtype. A consistent result, as obtained by using 2 Mb of genomic sequences (**Table 1**), showed that LTRtype characterized more numbers of normal retrotransposons and solo-LTRs but fewer numbers of truncated and nested elements than REannotate (**Table 3**). Apparently, LTRtype is able to identify a large number of structurally different types of elements, showing its exclusive applications for LTR retrotransposon discovery.

**Table 1:** Comparisons of long terminal repeat (LTR) retrotransposons in maize genome sequence (Chr10: 0–2 Mb) predicted by LTRtype (default), REannotate (-n -t -d = 10k -s = 10k -c), and TEnest (default).

Software	Normal	Truncated	Solo-LTR	Complex	Nested	Total
LTRtype	59	200	172	7	182	438
REannotate	64	401	115	0	230	580
TEnest	49	281	119	0	427	449

**Table 2:** Structurally different types of LTR retrotransposons identified in maize genome sequence (Chr10: 0–2 Mb).

Structural types	Layer 1	Layer 2	Layer 3	Layer 4	Layer 5	Total
LTR-IN-LTR	37	17	3	2	0	59
LTR-IN	28	11	5	0	0	44
IN-LTR	23	9	2	1	0	35
LTR-IN-LTR-IN-LTR	5	2	0	0	0	7
LTR-IN-LTR-IN	3	3	0	0	0	6
IN-LTR-IN-LTR	4	0	0	0	0	4
IN-LTR-IN	1	0	0	0	0	1
LTR	106	40	19	7	0	172
IN	48	39	16	3	1	107
Others	1	1	1	0	0	3
Total	256	122	46	13	1	438

**Table 3:** Comparison discovery of LTR retrotransposons predicted by LTRtype (default) and REannotate (-n -t -d = 10k -s = 10k -c) in maize Chr10 genome sequence.

Software	Normal	Truncated	Solo-LTR	Complex	Nested	Total
LTRtype	4512	14697	12233	142	12120	31584
REannotate	3771	28712	8452	0	15646	40935

### Detection of Structurally Complex and Nested LTR Retrotransposons in the *A. thaliana* Genome

**FIGURE 6:**
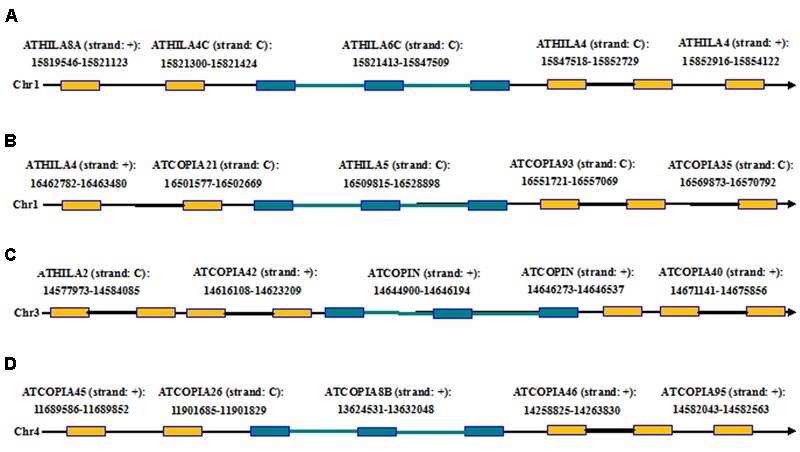
**The chromosomal locations of the complex retrotransposon elements with the three LTRs including two adjacent elements in flanking genomic regions of the *A. thaliana* genome. (A–D)** The four complex retrotransposons with the three LTRs. Rectangles in brown and blue represent LTRs from the two different elements, black and blue thick lines denote internal regions from the two different elements, and arrows show the direction of DNA strand.

Here we report an example by running LTRtype against *A. thaliana* genome to further confirm the usage of the program. After running LTRtype we collected a total of 2,263 structurally different LTR retrotransposons that consisted of 474 LTR-IN-LTR elements, 10 LTR-IN-LTR-IN-LTR elements, 883 solo-LTR elements, and 892 fragmented elements; we also found the four structurally complex retrotransposon elements that contain more than three LTRs in the *A. thaliana* genome (**Table 4**). Of the 10 structurally complex retrotransposon elements with three LTRs, seven did not contain any nested insertion, while the other three were inserted by other types of retrotransposon elements, evidenced by younger ages of insertion events (**Figure 6**). All these elements were nested over five layers, counting 1,884, 338, 35, 4, and 2, respectively (the elements of layer i are inserted by elements of layer i+1). Our results showed that the average ratio of solo-LTRs/LTR-IN-LTR was 1.9:1, which is in good agreement with a formal estimated ratio (2:1) (Pereira, 2004). In this study, 2,263 retrotransposon elements were identified that account for 5 Mb sequences, representing 4.2% of the *A. thaliana* genome. The results are consistent to the two best studies to comprehensively analyze LTR retrotransposons in the *A. thaliana* genome (Pereira, 2004; Peterson-Burch et al., 2004). This analysis thus indicates that, besides the advantages to detect structurally complex and nested insertion events, LTRtype is able to efficiently mine LTR retrotransposons in *A. thaliana* and other plant genomes.

**Table 4:** Structurally different types of LTR retrotransposons identified in the *Arabidopsis thaliana* genome.

Structural Types	Layer 1	Layer 2	Layer 3	Layer 4	Layer 5	Total
LTR-IN-LTR	415	54	4	1	0	474
LTR-IN	173	21	4	0	1	199
IN-LTR	193	20	1	0	0	214
LTR-IN-LTR-IN-LTR	9	1	0	0	0	10
LTR-IN-LTR-IN	11	0	0	0	0	11
IN-LTR-IN-LTR	16	3	0	0	0	19
IN-LTR-IN	13	0	0	0	0	13
LTR	747	122	12	1	1	883
IN	303	117	14	2	0	436
Others	4	0	0	0	0	4
Total	1884	338	35	4	2	2263

## Conclusion

LTRtype is an efficient software tool to identify different types of structurally complex LTR retrotransposons and characterize their nested insertions in the genome. Comparing with REannotate and TEnest, LTRtype was found to perform high-quality discovery of LTR retrotransposons with an improved solution to deal with the fragmentation of repeat sequences and particularly to identify structurally different types of elements together with their nested events. The application of this annotation tool in the *A. thaliana* genome has further proven its capability to correctly and efficiently identify structurally different LTR retrotransposons. The use of LTRtype has precisely identified a large number of nested retrotransposon elements in the intergenic regions of this small plant genome, showing a great efficiency of LTRtype to characterize nested LTR retrotransposons in more and more other sequenced flowering plant genomes. Such a reconstruction of these past insertion events can not only reconstruct different structural makeup of LTR retrotransposons and thus decipher genomic processes of LTR retrotransposons, but also shed light on the evolutionary dynamics of the entire genome. The obtained results confirm that LTRtype is an automated methodology for efficiently genome-wide mining structurally different types of LTR retrotransposon elements that may have largely contributed to the function and evolution of LTR retrotransposons in the eukaryote genomes.

## Author Contributions

L-ZG conceived and designed the study. F-CZ and Y-JZ developed the pipeline and drafted the manuscript. Q-JZ performed data analysis. L-ZG revised the manuscript. All authors read and approved the final manuscript.

## Conflict of Interest Statement

The authors declare that the research was conducted in the absence of any commercial or financial relationships that could be construed as a potential conflict of interest.

## Footnotes

1 http://www.repeatmasker.org

## References

[B1] Bao Z. Eddy S. R. (2002). Automated de novo identification of repeat sequence families in sequenced genomes. *Genome Res.* 12 1269–1276. 10.1101/gr.88502 12176934 PMC186642

[B2] Bennetzen J. L. (2002). Mechanisms and rates of genome expansion and contraction in flowering plants. *Genetica* 115 29–36. 10.1023/A:1016015913350 12188046

[B3] Bennetzen J. L. Ma J. Devos K. M. (2005). Mechanisms of recent genome size variation in flowering plants. *Ann. Bot.* 95 127–132. 10.1093/aob/mci008 15596462 PMC4246713

[B4] Campagna D. Romualdi C. Vitulo N. Del Favero M. Lexa M. Cannata N. (2005). RAP: a new computer program for de novo identification of repeated sequences in whole genomes. *Bioinformatics* 21 582–588. 10.1093/bioinformatics/bti039 15374857

[B5] Devos K. M. Brown J. K. Bennetzen J. L. (2002). Genome size reduction through illegitimate recombination counteracts genome expansion in *Arabidopsis*. *Genome Res.* 12 1075–1079. 10.1101/gr.132102 12097344 PMC186626

[B6] Edgar R. C. Myers E. W. (2005). PILER: identification and classification of genomic repeats. *Bioinformatics* 21 i152–i158. 10.1093/bioinformatics/bti1003 15961452

[B7] Ellinghaus D. Kurtz S. Willhoeft U. (2008). LTRharvest, an efficient and flexible software for de novo detection of LTR retrotransposons. *BMC Bioinform.* 9:18 10.1186/1471-2105-9-18 PMC2253517 18194517

[B8] Flavell R. (1986). Repetitive DNA and chromosome evolution in plants. *Philos. Trans. R. Soc. Lond. B Biol. Sci.* 312 227–242. 10.1098/rstb.1986.0004 2870519

[B9] Jurka J. Kapitonov V. V. Pavlicek A. Klonowski P. Kohany O. Walichiewicz J. (2005). Repbase Update, a database of eukaryotic repetitive elements. *Cytogenet. Genome. Res.* 110 462–467. 10.1159/000084979 16093699

[B10] Kalyanaraman A. Aluru S. (2006). Efficient algorithms and software for detection of full-length LTR retrotransposons. *J. Bioinform. Comput Biol.* 4 197–216. 10.1142/S021972000600203X 16819780

[B11] Kellogg E. A. Bennetzen J. L. (2004). The evolution of nuclear genome structure in seed plants. *Am. J. Bot.* 91 1709–1725. 10.3732/ajb.91.10.1709 21652319

[B12] Kimura M. (1980). A simple method for estimating evolutionary rates of base substitutions through comparative studies of nucleotide sequences. *J. Mol. Evol.* 16 111–120. 10.1007/BF01731581 7463489

[B13] Kronmiller B. A. Wise R. P. (2008). TEnest: automated chronological annotation and visualization of nested plant transposable elements. *Plant Physiol.* 146 45–59. 10.1104/pp.107.110353 18032588 PMC2230558

[B14] Kronmiller B. A. Wise R. P. (2013). “TEnest 2.0: computational annotation and visualization of nested transposable elements,” in *Plant Transposable Elements* ed. Peterson T. (Berlin: Springer). 10.1007/978-1-62703-568-2_22 23918438

[B15] Kumar A. Bennetzen J. L. (1999). Plant retrotransposons. *Annu. Rev. Genet.* 33 479–532. 10.1146/annurev.genet.33.1.479 10690416

[B16] Kurtz S. Choudhuri J. V. Ohlebusch E. Schleiermacher C. Stoye J. Giegerich R. (2001). REPuter: the manifold applications of repeat analysis on a genomic scale. *Nucleic Acids Res.* 29 4633–4642. 10.1093/nar/29.22.4633 11713313 PMC92531

[B17] Ma J. Bennetzen J. L. (2004). Rapid recent growth and divergence of rice nuclear genomes. *Proc. Natl. Acad. Sci. U.S.A.* 101 12404–12410. 10.1073/pnas.0403715101 15240870 PMC515075

[B18] Ma J. Devos K. M. Bennetzen J. L. (2004). Analyses of LTR-retrotransposon structures reveal recent and rapid genomic DNA loss in rice. *Genome Res.* 14 860–869. 10.1101/gr.1466204 15078861 PMC479113

[B19] McCarthy E. M. McDonald J. F. (2003). LTR_STRUC: a novel search and identification program for LTR retrotransposons. *Bioinformatics* 19 362–367. 10.1093/bioinformatics/btf878 12584121

[B20] McCarthy E. M. McDonald J. F. (2004). Long terminal repeat retrotransposons of *Mus musculus*. *Genome Biol.* 5:R14 10.1186/gb-2004-5-3-r14 PMC395764 15003117

[B21] Meyers B. C. Tingey S. V. Morgante M. (2001). Abundance, distribution, and transcriptional activity of repetitive elements in the maize genome. *Genome Res.* 11 1660–1676. 10.1101/gr.188201 11591643 PMC311155

[B22] Nystedt B. Street N. R. Wetterbom A. Zuccolo A. Lin Y.-C. Scofield D. G. (2013). The Norway spruce genome sequence and conifer genome evolution. *Nature* 497 579–584. 10.1038/nature12211 23698360

[B23] Ossowski S. Schneeberger K. Lucas-Lledó J. I. Warthmann N. Clark R. M. Shaw R. G. (2010). The rate and molecular spectrum of spontaneous mutations in *Arabidopsis thaliana*. *Science* 327 92–94. 10.1126/science.1180677 20044577 PMC3878865

[B24] Pereira V. (2004). Insertion bias and purifying selection of retrotransposons in the *Arabidopsis thaliana* genome. *Genome Biol.* 5:R79 10.1186/gb-2004-5-10-r79 PMC545599 15461797

[B25] Pereira V. (2008). Automated paleontology of repetitive DNA with REANNOTATE. *BMC Genomics* 9:614 10.1186/1471-2164-9-614 PMC2672092 19094224

[B26] Peterson-Burch B. D. Nettleton D. Voytas D. F. (2004). Genomic neighborhoods for *Arabidopsis* retrotransposons: a role for targeted integration in the distribution of the Metaviridae. *Genome Biol.* 5:R78 10.1186/gb-2004-5-10-r78 PMC545598 15461796

[B27] Polavarapu N. Bowen N. J. McDonald J. F. (2006). Identification, characterization and comparative genomics of chimpanzee endogenous retroviruses. *Genome Biol.* 7:R51 10.1186/gb-2006-7-6-r51 PMC1779541 16805923

[B28] Rho M. Choi J.-H. Kim S. Lynch M. Tang H. (2007). De novo identification of LTR retrotransposons in eukaryotic genomes. *BMC Genomics* 8:90 10.1186/1471-2164-8-90 PMC1858694 17407597

[B29] SanMiguel P. Gaut B. S. Tikhonov A. Nakajima Y. Bennetzen J. L. (1998). The paleontology of intergene retrotransposons of maize. *Nat. Genet.* 20 43–45. 10.1038/1695 9731528

[B30] Schmutz J. Cannon S. B. Schlueter J. Ma J. Mitros T. Nelson W. (2010). Genome sequence of the palaeopolyploid soybean. *Nature* 463 178–183. 10.1038/nature08670 20075913

[B31] Steinbiss S. Willhoeft U. Gremme G. Kurtz S. (2009). Fine-grained annotation and classification of de novo predicted LTR retrotransposons. *Nucleic Acids Res.* 37 7002–7013. 10.1093/nar/gkp759 19786494 PMC2790888

[B32] Tamura K. Peterson D. Peterson N. Stecher G. Nei M. Kumar S. (2011). MEGA5: molecular evolutionary genetics analysis using maximum likelihood, evolutionary distance, and maximum parsimony methods. *Mol. Biol. Evol.* 28 2731–2739. 10.1093/molbev/msr121 21546353 PMC3203626

[B33] Tian Z. Rizzon C. Du J. Zhu L. Bennetzen J. L. Jackson S. A. (2009). Do genetic recombination and gene density shape the pattern of DNA elimination in rice long terminal repeat retrotransposons? *Genome Res.* 19 2221–2230. 10.1101/gr.083899.108 19789376 PMC2792168

[B34] Vitte C. Panaud O. (2003). Formation of solo-LTRs through unequal homologous recombination counterbalances amplifications of LTR retrotransposons in rice *Oryza sativa* L. *Mol. Biol. Evol.* 20 528–540. 10.1093/molbev/msg055 12654934

[B35] Wendel J. F. Jackson S. A. Meyers B. C. Wing R. A. (2016). Evolution of plant genome architecture. *Genome Biol.* 17 1–14. 10.1186/s13059-016-0908-1 26926526 PMC4772531

[B36] Wessler S. R. (2006). Eukaryotic transposable elements and genome evolution special feature: transposable elements and the evolution of eukaryotic genomes. *Proc. Natl. Acad. Sci. U.S.A.* 103 17600–17601. 10.1073/pnas.0607612103 17101965 PMC1693792

[B37] Wicker T. Sabot F. Hua-Van A. Bennetzen J. L. Capy P. Chalhoub B. (2007). A unified classification system for eukaryotic transposable elements. *Nat. Rev. Genet.* 8 973–982. 10.1038/nrg2165 17984973

[B38] Xu Z. Wang H. (2007). LTR_FINDER: an efficient tool for the prediction of full-length LTR retrotransposons. *Nucleic Acids Res.* 35 W265–W268. 10.1093/nar/gkm286 17485477 PMC1933203

[B39] You F. M. Cloutier S. Shan Y. F. Ragupathy R. (2015). LTR annotator: automated identification and annotation of LTR retrotransposons in plant genomes. *Int. J. Biosci. Biochem. Bioinform.* 5 165–174. 10.17706/ijbbb.2015.5.3.165-174

