# Supplementary material for: LTRtype, an Efficient Tool to Characterize Structurally Complex LTR Retrotransposons and Nested Insertions on Genomes
Source: Front Plant Sci. 2017 Apr 4;8:402. doi: 10.3389/fpls.2017.00402 (PMC5379124; doi:10.3389/fpls.2017.00402)
Supplement: Supplementary file 1 [file Data_Sheet_1.docx]

**Supplementary Library S1. The LTR retrotransposons sequence library of maize.**

>CENTA_IN

agtggtatcaaatttcaggttgctcggtgagagatctcaatcttccttgttttgtttacctacagtccacttttgcccaaagatatatttagagcagaaattcacctaaaaacagtttgagcctttgctttactacttagttttcgacttgttgaattccggtagctgcatttgggtcgagttgctggtctaaagttttcttaccgctagagtttcgagttcgcgccaccttgtttcaatcaccagtttagacctcttgctgcaattcaaccaaaaagaagagaaagcaaaaggcgagtgcacaaaaaaagccgcactaatcagcaaaacaaaaaaagacacgtgcaaaacaaaagagagagaaaaaaaccagttctgaattttggtagataaaatttgtaagtgcaacaaaacaaaaggcagtttgtgtgccttctttttatagtttcagaaatcagattgttgttctgagcttttggtgatactatttgtgtaacggctcgcgtctctattacggtttggactaggaccagcacaacaccttgtggaacgtttattcaacttgttgtggctaacgtggtactagctattccttggaactattgtttaaacagccacctataaatccacaaaattttctacaacaccaccaggttgtgctagcagccactgttgttgttgttcgtgctgtttgccagcgcctcctgctttgcgtggtgagaacttgtaagaacttgtttaaccagtttgagagtgagagattacaacaatgattcctagtagtttatagaatcaaagatattttttattgtttcttgtctttactaaacatggcaggtgatatggacatttttgacccaaccgaacgttatattggaggcatcattcaacacttgcctttatatgccggtaaattcgatcctcatgcatacattgattgggagctaaagctagataaggaatttgataagcatgatctatctcaaaaacaaaagatttatattgcctctaatttgttaactgagcacgcattgatggaatggaaatacatttgtaggcacaacaaagttccacaatcttgggaagacttcaaacttcattttagagatgcattcattcctgcatactatgctgatcatttgctttctaaattagacaccttaaagcagggtgctaggactgtgaaagattattattatgattttaaaatttttaccatgtttgctcgtttagatgaatgcatggaagatgtcatgactaggttcatgaaaggactcaattctgaaattcagactatagtcatgcatgaagcatacaaacacatttctcacttgtttttgcttgcatgtaaagctgaaaatgagattctattatacaattatacaagcactgaacatgtgagccataattcctcttttgcatcttctctacatgctgatcaagaacacaaaataatgaaaccagctgttgtttttccatcatcacaagaagaattgattgctgacacttgtgatagtgaagatttgtgggataatgattcacatgtactaagacaacaactagtaaatgaacatgttacatctattattgaaccaaacattttggctaaaaaggaacatgtaatttgtattgcaaacgaaactgaagaaataaatttgctctcttctttaaatacttggggctatattgaatttgatgatctttttgagctcggtaatttggaaaatattttatttgctagattcaactataccatgtccttctcatgatatattttatattgctggcaagtacaacaacataggacaatttcttgtgcatagaatttctatttcatctagatatgttgtttcttcactttgtgcaaataagatattggtatgttctcaagaagaaaagaatctcttgtttccatgtactttagttgaagtttcaggtttatatttgaaagacattaataaaagcttagtcatcaacatcaatcatgatgcaaaaccgaggacggtttgctatcaagaaggggagaa

>CENTA_LTR

tgatgagaacataacccgcacagatatgaccatgttaatggctcctgctacaaagacattgaggaacaaagaagttgattggggaccaagtaatgatatttccaacatttccaacaaagcaagcacatcatcaaatttaaagatatacttgggtgaggagcatacactagagtcgaggacgactctattacaagaaggggaggatgatgaggacatcactgccatcaatacaccacaccagcgacctccttcaccatttaataatggaccagtaaacgagtccgtgcacgtaaattttattatcaggtgaactcgttccttattgttgaagctaatcattccttaaatgaggtactaataccttgtgattactttattaatctaaggtgtttgggaggtgaaccatctagaatttgagaaggcaacaaggcaataaaagctgctccacttgaggggatttcgaaactacaacaagtgcaagtttaagagggcatatctttcagctcctaaggttgtttaatgcaaataagcacttgttggaaaggtctctttgtctactttctagtggatcaagaatcaacgagagatcagacactaagtgtccagaaactgccgagtgaactcctgctctacccaagtcaatttcgtaactgcagcatgcaccaaattaaatggagcataactttccactcccaaggttgtttagtgcaaataactacttgttggaaagctctcttcgtctactttcatgtgcatcaataatcaatgacagaaaccaaacgaggcgtccagaaactgccgagagagtttcgttctccattagaactcctttctattcctctatttaagcaactagcagccaccaaagaacttgggtttttgtttgatgtaagtttagcctttgctacttccttgtaaacgcatgtgtcggctagaccacccggatacttgaaacagaaccccaactctatcagatccgtgagtgtctgctttttatcttgttcttgcttgttctcgattgcttgcaggttcaaggctgttcttggcacggcaagggcagcaacaacaggagccgatgtaactatcgctaaggcgcagcacccttgtggttgttgtagtcggatagcacaacgtcgacctccaccccaaatcgtagttatcaggagacggtgtacctgtcgctcaaggcgccacaccatcttggttgtggtagtcgggcagccaacgtcgttctccaacaagtttccacctccatcatctctcatcgaaagatcgggcacccttctacccgttgggtttatca

>CINFUL1_ZM_IN

ttggcgcccacttccggtgaactcacttccacttttttgagctgatggcttcgttcaacaatcaagctagagctgcttcggctccgaagctggtactcccgataacaggcggatcatgctcagagcgagccaacaagaagcagaagaaggaggcacagagaagggtacaacatgtcggggtgcaaggacccttcatcaaatcaagatggtctcacattccaattaccttctctcaagaggatcttcaactcaaggattacccacacaacgatgctatggttatctcttgtgtgatcaaaggattcctggttcataatgttttggttgatactggcagcgcagctgatatcatatttgctaaggccttcagacagatgcaagagcccgaagacaaaatccatgatgccacacatcccctttgtggcttcggaggaaggcagatcgtagcacttggaaaaatcacaatgccaataaccttcggattcattaacaacacaaggactgaacaagttgtatttgatattgttgacatggaatacccttacaatgcaatcattggtcgtggtactctcaatgccttcgaagcaattcttcatccagcttatctttgcatgaagataccttcggaccaagggcccattgcaattcatggaagtcaggaagctgctagaagggccgaaggaaattggacagactcaaaagcaatccataacatagatggagctgaagcttccaaggcgcaactcgaagctgatactaagcaggctgaagccgaggctacaacaattcaggctgaaaccgaagctgggccttcagtgcccaccgagatggagcgtgctgaccctaaggaaaaatcaatagagcggattacaactgaaaaaatcgaagctcctggtcccgaagcgtcgaacaaaagtattgactacattattcgtcatgcttcgggaaaggtactatcccaagaagaaatgttagaagcgcaacactatgcccaaaaattgagatatccgaagggggcgttagtgttcaatggcagtggcgaagaagactttttgtactgtctcccggatgacaaagaaatatccgtttgctgggagataggtaaaagcatcggattcccgaagctggaagacggtctttcgatttatcgaaggacgaacttgctgacagcgtagcata

>CINFUL1_ZM_LTR

tgttgggaccatgcttcgtcgccgaaggtcctgcagaaagagacagcttcggctgaagctgctcgtacgtgacgccgaaggcaccattcatgaagcttcggtattacggcatatccgaagatgaagggtcgaaccgacttaaagatagaatgaccttttagtccataggggtctgagtcattgttgtaaacttttatgaggggcatgattgtaattcttcacaggctgtgtcctgtgcctataaatagtgaacaatattcctttactgttcacgcattcctgtaattgcaaacgcttcaatcgggaattattatttgtcaaggcagaggtataaatgttcataaaacattatgttcaagcatcttaaattcatatgataagatatgtgaataatgtcacttattttcgatatattcatctttaatatttcacattgcatattactttgtattaactttgtaattttaattacgaaggtataaccttcgtaatattctgctcatgatcttcgtccgaagttcattaaatccttggggaaataatgcttcagcggacgaagggcattaatatttaacattttatgttgccttgttcttaatccatagcatttgagagcaagtccccaaca

>CINFUL2A_IN

ttggcgcccacctccggtgaactcacttccacttttcgagctgatggcttcgttcaacgatcaagctggagctgcttcggacccgaagctggtgctcccaatcacaggtggttcatcctcagagccagctaacaagaaacaaaagaaggaagcacagagaagggtacaacatgttggggtgcaaggacccttcatcaagtcaagatggtctcacattcctattaccttctcccaagaggaccttcagctcaaggattacccacacaacgatgccatggttatctcttgtgttatcaaaggatttctggtccacaatgtcttggttgacacaggcagtgcagctgacatcatatttgctaaggccttcagacaaatgcaagagctagaagataagattcatgatgctacccatcctctctgtggcttcggaggaagacaaattgtagcactgggcaagattaccatgtcagtgacctttgggttcatcaacaacactagaactgagcaagttgtgtttgacattgttgacatggaatacccttacaatgcaattattggtcgtggcaccctcaatgcttttgaggcaattcttcaccctgcctatctttgcatgaagatgccttcggatcaaggacccatcgctattcatggaagccaggaagctgccagaagggccgaaggaaactggactgattcaaaagcaatccataacatagatggagctgaagcttgtgaatagtacaaattcagaagggagaaagcagcttcagcagatcagccgaagcccatgctcttatgtgaggatatagcagagcagaaggtgctgttgggctctcagttatccgaagaacaagagaaaaccttgataaggtttttgttcaacaacaaagatgtttttgcatggtcagccaatgatctctgtggagttaatagggatgttattgagcattcgctcaatgtcgatccatccttcagacccagaaagcaaaggcttcggaaaatgtcagatgataaggccgaaggtgctcgcaacgaagtcaaaagactcctcagtgcaggagttatcagagaagtaaagtacccagaatggttagctaatactgttatggtaaaaaaggccaatggcaagtggcgaatgtgtatcgattttacagaccttaacaaggcttgtccaaaggatgaattcccattgccaaggatagactctttagttgatgcaacagcttcttcagagctcatgagtctgttagactgttattcaggctatcatcaaatctggatgaagagggaagatgagccaaagactagcttcataactccaagtggcacatattgttatcttcggatgcctgaggggctcaaaaacgctggaggaagtttcagcagaatgactgcgaaggttctccagtctcagataggcagaaatgtgctaacttatgttgatgacatcattgtgaaaagcacgaagcaggagaatcatattgctgacttgcaggagaccttcgccagtttcagacaagctggcttaaagttgaatccagaaaaatgcgtcttcggagtaaagaaggggaaatttctaggatgcttggtttcaacaaagggaattgaagctaatccaagtaagattgaagctatacttcggatggagccaccaactacaaaaaagggggctcaaagattgacaggaaggttggcatctcttaatagattcatatccagatcagcagagagaaacttaccattcttcgaagtgctgaagtcagccgaagtctttcaatggggaccaatccagcagaaggccttcgaagagctgaagcagtatttaatagatctaacagcactaactccacctatgccaggggctcctttattattatatgtggcagcttcgcactcagcggtaagtgcagcacttgtccaggagaagcttgatggccaaaccaggaaacaggtcccagtatattttgtatctgaagttcttagtatatcaaagaaaaactacacagaattagaaaaggtgttatatgttgttttgatggcatccaggaagcttcggcactattttcaagcttacaacataattgttccttcttcacaacctctgaaggatattatgaggaaccgagaagctactggaaggattggaaaatgggctgcagagctcaatgaattttgtattgaatatgttcatagatcttcgattcagtctcaggcgttagcagacttcattgctgactggacgccaggggctcaggaggaagaagcaaataaagacaccgaagcatggacagtgttttgcgatgggtcctggggaaccttcggagcgggagcggctgctgtgttggtttcaccttccaaagttaaaacttgttatgtggcaaagcttgattttagttgcacaaacaacattgccgagtacgaagccctgcttctgggtcttcggaagctaaaagcaatgggaatcagaagggctgttcttaaaactgattcccaagttgtttcgggtcatatcgacaagagttgcaaggctaaagatccgaagcttgaaaagtatctggacatggttcggagagttgaagcttcctttgagggattttctgtcaaaaatatccctcggggacaaaatgagcatgctgatttgctagctaagtcagcagcacaggggctgcccttaccttcggatgtcttcttcgaaacaataaaagcaccttcggtggaacttcttgaaagagcagtcctcaatatatctcctgtttatagcgaagattggaggactgagataatctcttatcttcagggtaaattcctttcagatgacgaagcctataacaggaggatagaggcaagagctcgtccatatgtcatgatagaaggagagttgtacaagcatggagtttgtgctccgctactcaagtgtttatccagaaccgaaggtatagagttgatgaaagaaatacatgcaggcctgtgtggatctcacattggatctaggccgttacttggaaaaattttccgccaaggattttattggccgaaggcagcttcggatgcagcggaattagtccaaaagtgcgaaggttgtcagaaatgtgcacgagatcaaaaacaaccttcgtctttaacacagctcatacaacccatttggccattgcaaaggtggggccttgacttgttaggtccattaccaccggcccaagggaacctaagatatgttgtagtggcggtggaatatttttctaaatggattgaggcaaagcctttagctacaataacttcggccaccgtccaaaagtttttctggcagaatattgtttgtcgtttcggggtaccaaaggctatcactgtggataatggaacacagtttgactccgaagcttttagggatttctgtgatcaaattggtacgaagatccattttgcatcagtcagacatccggagtcaaatggactcgttgaaagagccaatggtattataatgacaggaataatgaagtcaatcttcaatcaaccaaggggaaagtggccagatcagttaaccaaagtggtgtggagccataatacaacaacatcaaggtctacaggctttaccccattcaagttattattcggtgacgaagcaataactccagaagaagctaaaaccggatcaataagggtagtagcttcggcagcatcaggttctgaagctgattattctgtggaaaaagatgctttagaagggatcaggttgcaagccgtggaaaacatcaacaaatatcaagccgaaacaattaaatggcgagatagaaaggttcggctaaagaatattgagccggacacttggtgcttcaggagagtggccaacccagaaacagtgggcaagttacagttgaaatgggatggacctttcttagtagcatcttcgtcaagacccggttcatacagattgaaggatatggacggcaacgacattcctagatcttggaatgcggatgagcttcggcgatattatgtataattcgatgtaatttttcatattttttatttttctttcatggcacccttttcctttccgaagggggagaaaggtttttaatggggccatcacatgtaatttccttttttagttctataagagcaaaaccccccaaggaatgtaaatgtaaaaactgaggacgcaccatcgagtgccgaaaagtaaaaacgaagaagctctaaagtcgttcctaagggaatgcagagcttacagcgaaaagtcaacgctgattccgccaaaagtaaaggcgaagaagctccaaagtcgttcctaagggaatgcagagcttacagcgaaaagtcaacgctgattccgccaaaagtaaaggcgaagaagctccaaagtcgttcctaaggggatgcagagctgaggtgtgattaaggaaataatggctatgagtatggcttgaggtgtgattgtttggccattcatttgcacatcgcattacatcataacatttgcattcataaacattcatctaggcatatgtaggataatcatcttcatggtataagcggttgcttcggcaaaataaaaaaaagagatagaaaaaaaaggggaggaaaagaaagagcttcgtgtataaaaaggagagcttcggaaagaaggaaaatgttgttttctatgcttcgttgcgtacgaaaagaagggaaggtgtttttctgcttcggctcaaaaaagaaaatttcgtccacatcaaagcatttctcatacatcaatggaaggataaggatatattacaaggtatgaacagaattcattaaagacaagttttagttacatttacaaaagttatctcaaaagtttctcaagtactgtctacaatctactgtttaatctccaaggagcttcagcttcggcgtcatttttaatcatcggcttcggcttcgtcatcctacatgaataaggtcgttgaaagtcaaaatcgagcttgcaagaaaaggtaagcacaaggtatggtttcttactggttcaaggtggctacgagcttcgtctccagccttttctcgcccaccttttgtccatatcatttttacaaatctattagagatacttcgggcgagatcaggaatgtcatccaggattgatgatgataaagtgaaatttggcctattgacaatttttccatgatcgcagccagctttcaggaaggctgcagcagtgcctcgagaagtcacccaggcacagaagtcaccgtgcccagctatgacttcgtcgagctcgtcaatctcaccctcaatatgttcgaaggtcttcggtaaatcttcagctgaggggacgaatttttcactgctggctccaactgagtgaaaaaattttcttaatcaatgaatgcatttgttgctaaattccagacatttttcttgaaggcttgtcaatagttttctcaaatccgaattttgttgagcttcggcttcaagttttgcattaagatcttgcttttctcgttcgaactattcagactggcggataagcttcatgttcagttctgttatttttgcttcagcttccgccaatgaaccttcggttgcctggagctcaaagttctttttctcgataacatccgattgctcctttattttgctttccaaattttcaattataacttcgtgtttcttgtcttcaaaatcttgctgcattttcaaggctttgctcaatagcatactctgcacggaaacaaccttcgtcagacatgttctcgttattagaaacaataaaggttaagggaaaaatagttcaccttgaaattggaataaaataagctaccaacgatatgctgtcgtcggtagcggctgatgtctgtttctagcttcggaaaactgatactctttgacagagtactgataactttggccccagtttgatctcgaatacaatctaatttttcgtcatcaatgcctccgaagaggagtgctccaggtttgtatccgcagattttgcatattctctaagctcttctctttcagcttttgacagtttttctccaactaaattctggaatatgaacgcttcgttctctgaagcttcatcagcaatttctttccctttctccgacgctgcggtcgcggcctcttcggcggcggtggtggcttcttctgcagccatgtctagcagtattttgtcaatgtgtgcgattgtgctctccaggttcaaatcttcagctgcggcagctgcgacctccgaaggtacaatttcaatatctgtcccctccgcagccgctggtgttttctgagctgaagctcttggcggtgtcttgtcaattacttctgtcacagtgataattctttgtcttttcgctttgattactttcttcgatttttcgggctccttgtccttctgaaaaaactttgtcagtcggggcctcagtggacttagcttcgcaggtaaggattcagtcattaccttcagaatttcctcaaggtcagcgacagagggtgatgcgggggtttcttcgtcggatagatataaacaataagtctgacaatagcgttaggggtcagctggtgaaaataaataccgaacctcttcagtacctctgcaataatcccatgtagggggaatcttaatccagcttttagaaaacttttgaaaataacaatttcatccttctccggctttggggtagtctcttctcccccgaagcgcaatagcttcttctgattttcagtaaaaaagcccgattttaccatcttggacagatcagccttcgaaacagtagatttcccgaagtccaaatggctgggtttgcttggcatagcaatgcgataatcatcttcgggatcagtttcctcaatatcttcttcctctgcttcagcgattgcttgttctgcatcttcactaggaatcttttccgaagtcactagcccggatcgttgcatgacttcggaaatggggacagtctccgaagcttcagtttcgtccccctcacgttcaaccctggcggtagagcgcactctggccatttaattatgaacttgtggaactttaatactttcttcctccgaagcaggtttcaaacaggagcttcgttcgattccgacagacaagcttcggcgacggttaaaaattttggcagcaaaacagtgcaaatagcaatgaatgctgtggtaacttcacacctactcgtctgtttatatagtgcggcaggtaagaagcgaagcgccaggatttttacaccagcggacacccgctcgcactcgctgcgtggtggaccgcagagaccgaacagtaactctgaaaggtggggccgatacacgctgagaaattgaatcgtttctcgacaacgagctcagggaaggtgttttttagaccttcggcgctccgaagcttaagagacttttttcacggatcaagctcgttacgaaaaacgatctagcaccgcgaaaggggctac

>CINFUL2A_LTR

tgttgggcctgtgcttcgtcgccgaaggtcttctaggaagaagcggccttcggctgaagctgtttgtataagatggccgaaggtccctcttcatgaagcttcggtattacaaaccgacttaaagatagaatgaccttttagtccataaaggtctgagtcaatgttgtaaactttcataaggggcatacttgtaatccctcacaggctgcgccctgtgcctataaatagtgaacattattcctttactgttcacggattctggtaactgcaaccgcatattctggaattcaaccttttgtcaaggcagaggtattattgtattcaataattggatatattaagaaaatataatataattcgtccatgacttatttatctcttttgtaccttttattttatgtcatcttgcaatatctattgaaatcttagtacgaagatctaaacttcgtaattttactctcatcaaccttcgtccaagacccattatcctcaagggaataatgttttatggacgaaggacgttgacatttaacattttatgttgccttgttcttaattcatagcacttgagaacgagtctccaaca

>CINFUL2_IN

ttggcgcccacctacgatgaactcacttctacagctataacaatggcttcgcaaagcaaccaagttgtagcatctttggccacgaagctagtgtttccaatcacaggcggctcaagttctgaaccagccaacaagaaacagaagaaggaagcacatagaagggtacatcatgttggagttcaaggacctttcatcaagtccaagtagtcgcatattccaatcaccttttctcaggaagaccttcagcttaaagattatcctcacaacgatgcaatggtcatatattgcatcatcaagggatttttggtccataatgtcttagtggacacatgcagtgcaacagataacatctttgcgaaggccttcaggcaaatgcaagagctagatgataagatacatgatgcaacacaccctctttgtggtttcggagggagatagatagtggcacttggcaagataacaatgccagtcaccttcggctacgttaacaacacaaggacatagcaagttgtctttgatattgttgatatagagtacccatacaatgcaatcattggtcgagggacgctcaatgccttcgaagcaatactccacccagcatacccttgcatgaagataccttcggaacaagggcccattgctgtacatgggagtcaagaagctgtcaggagagccgaaggaagttggatggattctaaggctattcataatatagatgaagtcgaagcttatcagtagtataagcacaaaagagaaaaggttgcttcggtagaccaaccaaagcctatgctcttgtgtggagacatagccgagcaaaaagtattgctgggatctcaactatctgatgaacaagaaaagaccttgctaagatttttgtttaacaacaaagatgtttttgcttggacagccaatgacctctgcggtgtcaatagagacatcattgagcactcactcaatgtggactcatcctttagaccaagaaagcaaaggcttcagagaatgtctgatgacaaagccgaaggaccacgaaatgaagtaaaaagacttctaagtgccgacgttattagagaagtaacatacctagagtggttggccaacactgtcatggtgaagaaagctaacggaaaatggagaatgtgtattaattttacagatcttaacaaggcatgtccgaaggatgaattcccattgccaagaatagattccttagtggatgcagcagccacttcgaaactcatgagcttattggattgatactcaggctatcatcaaatctggatgaaaaaggacgatgagccgaagacaagcttcataactcctagtggcacttattgttatctttagatgcctgaggggctcaagaatgctggaggaagcttcagcagaatgactgccaaagttcttcactctcaaattggcaaaaatatgctaacatatgtcgatgacatcatagtaaaaagcacaaaacaagagaaccatgttgctgatttgcaagagacatttgccaatttcagacaagctggtcttaagttaaatccagagaaatgtgtcttcggagtaaagaatgacaagtttcttggctgcttggtgtcaacaaagggtattgaagctaacctaagcaaaatcgaagctatccttcggatagagccgccaaattcgaaaaggggggctcaaaggttagcaggaaggctggtatcattgaacagattcatttcaagatcaacagagaggaatctacctttctttgaaatattgaaatcagccgaagtcttccaatggggtccaacccaacaaaaagcctttgaagagatgaagcagtatttgatagatataacaactttaactccaccttcatcaggaaccccattgcttctatacgtggttgtttctcatactgcggtcagtgcagcacttgtacaagagaaacaagatggccacataaaaaagcaagcaccgatatacttcgtctccgaagtactcagcccatctaagaagaattacacataattggagaaggtattgtatgttgtattgatggcctccagaaagcttcgacactacttccaaacatatcatataatagtgccttcatcacaacccctgaaggacataatgaggaacagagaagccacaggaagggtcgaaaaatgggccgcagaactcaatgaattcaccattgattatgtacataggtcttcgattcaatctcaggcgttgtcagacttcattgtcgattaaacgctaggggctcaggatgaagatagaataaatgatatcgaagcttggatggtattttgtgatgggtcctggggaaccttcggtgcaggggcaactgttgtcctagtagcaccatccaaagttagaactcgttatgcagtacaattggattttagctgcacaaataatattgtagagtacgaagccctcctcttggggcttcgaaaacttaaggcaatgagtataagaagggcagttttcaagactgattcacaagttatatcaggtcatgtggacaaaagtagcaaggcaagagatccgaagcttgaaaaatatctagatgcagtccgaaggttggaagcttcttttgaaggtttctttgtaaagaacatcccaaggggagaaaatgagcatgcagacttattggctaaatcagtggcacaggggcttccgctaccttcggaggtgttttttgaaataataaaagcaccatcagttgagctcatggaaagagcggtgctcacgatttcactggtacatagtgaagattggaggactgagattgtatctttcctccagggcaactgtctttcagatgacgaaggttataataacagaatggatgcatggacaagaccatatgtaataatagaaggggagttatataagcacggagtttgctccccattgctcaagtgtttatccagaactgaagggcaagaactactgaaggagatacatgcaggactatgtggggcccacattggatctaggcccctattggggaaggttttcagacaaggtttctactgggcgaaggcagcttcggatgtagcagaattagtacaaaaatgtgaaaattgccaaagatgcgccagagaccagaagcaaccttcgtctttgactcaactaatacaaccgacttggccactgcaaagatggggtttggatctgttaggaccactcccaccggcacagggcaacttgaaatatgttgtggtggcggtggaatatttttctaagtggattgaagcaaagtctttggctacaataacttcggccacagtacaaaaattcttctggcaaaacatcgtttgtcgcttcagcgtgccgaaggctatcactgttgataatggaactcagttcgatgtcgaaactttcaaagccttttgtagccaaattggtacaaaaatacacttcgcatcagcgagtcatctggagtcaaatggattggtagaaagggcaaatggaattgtaataacaggaataatgaagtcaatcttcaatcagcctaagggaaagtggccagatgaattaataaaagtggtctcgaaccacaacactgctgtatcaaggtcaatgggatttactccattcaaacttctgttcggtgacgaaacaattacaccggaagaagaaagaacgagttcaataagaactttagcttcgacagaggacgaaggtgactgccaagtaacaaaggataccatagaagagaccaggcttcaagtcatagagcacattaataaataccaggctgaaacagtcaaatggcgtgacagaaaagtaagattgaaaaacaccaagccaggtcacttggtgcttcgaagggtggccaaccctgatatagtggggaagcttcatcttaaatgggaaggagcctttctggttgtatcttcgtcaagacctggctcttacaggttgaaagacatggatggaaatgacattccaagatcttggaatgcagatgagcttcgaagatattatgtgtaaacaatgtaattttcttattatttccttatggcacccttttcctttcaagttgggagaaaggtttttaacggggccataggttgtaatttttattctttgttttggcctatgcaatataaaggcaaaattcccccatatgtaaattgtaacatccagattcgcaccttcgagtgcaaaaagggacatggagaagctcaaaagtcgtccctaaggggatgcagagccaaaatatcacctgaaaggtgaagaagctacaaagtcgttcctaagggagcgcaaagtaagttccgaagctcaaaagttgttcctaagggaatgtagagctaagatgacacctaagtaaaaggtgaagaagctcaaaagtcgtttctaaggggatgtagagcttgaatgccacctaagtaaaaggtggagaagctccaaagtcgttcctaaggggattctgagcttggctccaaagtcgttcctaaggggatgaagagctgaaataccacctaagtaaaaggtgaagagactccaaagtcgttcctaaagggatgcagagtttgggtgtgtttttggcatacatttgcatagctcattacatcattcattgcattcatacaagcattcattagccattcttaggatcatcatcatcatcatgacataaagtactaacaggaaaagacagaagatgtctgtcttcgctggaaaaaatgcttcgttgaaaatggagatgctttgtctcagacaaaacttcatggcatatgaaggtgattcgtctttgataaaaaaatgcttcaatgtgaacgaaaagaatggaaggtgtttttcgcctatggctcaaaaattgcaagtgttggtttcgtccatatcaaagcaattcatacaggagcaaataggtaaaattatattacaagatattcacaaatttgtacaatattttgttcaactaacaattacaattccttgcaaagtaatgcaaagagaccctaagttttctacagatagatacaaaagaatgtcctaagagtcttcaggccttcagaacaagcttcggctcagcaaccttcctgcaaacagatgaaggtataaggtaatcagaaatttaataaaggaaaaccaaggtaaaaagacaggtaaaatagtcatactgaattgagcaatttcctagcttcatccccagccaactcacgtccgcccttagcccaaatttgtgtaatgaatctgttccctatacttcgggcttcggcggggatatcaactatatctgacgtcgaaagattgaaagttggcttgttgactgtcttggcatgagtacatccagccttcataaaagcagcagctgtgcctcgagaagctaccagggcacaaaaatctctatgcccgcttataacttcgtcaagggcttcaactttgttttcaatatgattaaaggctctaggatatcttcggtcgaaggggtaaacttttcagaagcagctccaaatgagttgaatatccccttaagccggtcagcacaccgagtcgcaaaatctacacacttgttccgaaggtccttaatagactcgtacaacttcgtattcttctcagttttggcctcgactctttttttaagtttcttcgtctcctggtcgaagcaccgtgaactctcatccaagatcgatcgagcttcatttagttcctcgcttaacttggtattttgggatcgtgattctgcgagggaactttcagctatttgcagtaggatagttttttcttctagataagtttcaagttcctttattttttcctctaatcctttaactataactttgtgtttcatgtcctcactatcctgctgcattctcagcgctttgcttagcagcatgctctgcaaacaaattaaagttagaacactcaatctatctcgaaggtaataaaaatcttgatgaagatcttaccttaaaattagaataaaataggctaccaacaatgtgttgtcatcggtaaccactgatgtttgcctcgagcttcgggaatccgacactcttagaaagagtgccgattatctttgccccggcgcggtcgcttatacatcctaaaatttcttcatcaaccctaccgaagagcaaagatcccggctggtaaccacaagatattacatattctttcagctcttccttgtcttcctcggaaagctcctggccgcccacgtgacgaaggtcaaaatccccttcatccgaaggagttttaacaatttctttttctttatcaggcactgtggccatggtttcttctggagctacatttgtctccacagtcacatctgcaaatacgtctgacacaagcctatcaatatctgacattgttgtttcggcttcatcagcatctttggcttcggcagcaccttcagcttcagctttggtatccgcatgaatcgctactttggccgccgaagctgaggacagtgtttgttcaattgcttccattacatttacaattcgttgcttcttttgttctttaactttctcctctgtagccgaaggctgttcttttctctgtaaaatttttgtcagttctcatcccagaggacttagcagcttgaagggtggggattcagtcattaccttcaggatttcggctgcttcagcagcagagggcgtcagaggagcttcttctcctgtctcatctacttcggtccgaagagggtattgtgtcaagcttccactttttagaaattgttgtcttaagttcaggagttgttttttatttcttcgaaactttttcatcctccttcactacccgagcagcttgcctactcaggatgctaacaatccttttcctttttaccccttcggcccctttgtcaagcctcttgtaatccgggtattcgaagtttaacgtgtccatcacccgattcagccttcgtttcggccgggtgccgaaggcggtagtcattaattggtcttcctttttggtgtagttgccaaggatttcgttgcacattgtttcgatcatttctagcaactctttgcatggcttcttgaattgcttctcaaatttaaatcagtaagggagccgaacaagttcgtactttttaccccccccttctttttcttaggcattccccagccactagaggtcggaaatattctatttgccaggtactcttgtactagatctctaatgctgatttcggctgccaccactttgaattctacttcggctaactggcatggggaaccaagctgcatgtggcacaagggccttgtcattccgaagcttaacttcaatggactcatgaccatagtcattagcttctcccttttcttctcgtctgcttttatgtagaaccattcgcttgtccaaccggtcggccacttggtacgatagccaattaccggggccttcgtatctttctgatatgcgaagttgtagcagccgaagttattatgaagaccatccgctctgcccttcgtttgatagtgcagctcatgcacttggcagaaagcttcgacattaggactcataccctggcttcggagagcccatatgtagacttgtgtaacgattgcattaggtgtcaactgatgaaagtatatctcaaaattctttaaaacctctccaatcatctcacgaaaagggaatcaaagccctgccctaaagaagctcttaaaaacaacgatttaagcctcccttggctgcggaactacctcctccccggcaaaccggacaagcttgctctcgacttcgccaaagtagcccaatttttttatcataaccatgtcgtctgccgtcacagtagatttaccaaattcaatatggctaggtttcattaggctcagaatggtgttgtcctctt

>CINFUL2_LTR

tgttggggtttttctccgccgaaggtcttcaaggcaaaaacaccttcgacaaaatgatacaaaatcttactgaagttatcttcagcctcggctcaactcctcgagacaaagctcaagggctttggcgacagtgcaagaagccgaaggtcaatagcttcggcccagaccaagcaaagaaggaaaaatgactagatagaccttattatttttaattacttataaacaaatgtttgagggcatgaatgtaattgtacccgggctgcgtcccgcgcctttaaatatatgaacagtacctcgtactattcacgctggattgtattcactctcacgtcattcttgcatccataccttctgccaagccgaatgtatcaatgtaacacaaatattattcatattttctcatattcactgaatatataaatatgaatatattgttgatttataattgtgatctttatttccttatattttattgcttaattgttcacatgataaaatgatgaaggtattcccttcatgaccttcgtctgaagatcattatatccaaagggaaataatacttcgaaggatgaaggtctttaaccaataacaactgtgttgctttgttcttcatttatagcatctgagaacaagtggccaaca

>Copia-34_ZM_IN

ttttggtaccagagccttggtggatgcaatacgacggctgtgtgagcccgccgagagagccagcaaagaaaaagagagttatcccacgccgcgcgccgccggtaagcaccatgagtgagacctcgagctcgagtggtggagtcagggaaagttccctgatgtggcccatgctcacccgagacaattatgccgagtgggcaatgcttatgcagtgcaatttcgaagccatggagatctggggggtgattgttcctggaatcgcccccaagcggtcgcaggatcgtcaagccatgagtgcgctgttgcgatccgtgcccaaggagatgtggcaatcactcggtgcgcgcaatacagtgaaggaagcttgggatgcggtgaaattgatgcgtctgggagccgatcgcgtgaaggaggtgaacgcacagaagcttctccaggagtttgagaatatcaagttcaaggacggcgagtctgttgaagatttcggcatgcggatcacaaatctcgtcggcaaccttcgcgccctcggcgaaattgttgaagacatccgcgtcgtcaagaagttcctacgtgttgtaccagcgcggttcacccaggtagtagtcacaattgaaatgttttgtgatcttaaggcattgtcggtcgacgagctggtggggcgtctacgtgcggcggaggagcgcattgaggacaaggtcgaacaggtggtcgacaagacggggcgtcttcttctcgccgaggaggagtggttcaagaaaaacaagcatcgcttccgctccaactacaaggagggaggtggtggctccggtgccagtgctcccaagcacaaggcgcagccacgatcagaaggtgggacctcaggttcagtcaagctgacctccgaggggacaccacggagaaaaggtcggtgtcgcaaatgcggcatctatggccattgggcggttgactgcaaacgttcaaggaaggagaaagcgaaagaaccacagaagtcggaggccaatctggccgtcggcagtgttgaggatcatggtgccctgatgttgtccatgtgcgatgttgcgcataactcatcttatgatgttcatttgacagagaagataataccaatgactgtccctgatggtgtgtgggtgctcgacactggagcaagcaaccacatgactggaaacatgtcgatgctgacacaacttgatcaaaaggttcaaggaacagtgaagtttggggacggatctcgcgtcaagatcagaggtatgggctctgttgtgatccaagaccgtaagagtggtcataaggttttgactgatgtttattacattcctgagttgaagtgtaatatagtaagtttgggtcaactggaagaaaaaggttttctttttgagggcaaaaatgggaagctgtgcatttatgatctggaacacaacctgttgatttctgctcctagaactggaaataggctgtatactgtcagatttggtttatcttcacctatatgtctgttagcaaattcagatcaaaaggcatggcagtggcatgctaggtatggacatttgaatttcagagctttacaggaattgggtgagaaaaatctggtagtggggatgccaaatgtgaagaaagtagagcaagtatgcgatgggtgtgtcttaggaaaacaacacagaaagcctttcccacaggtatctggttttagagcagaagaaaaccttgatttgtttcatgcagatttatgtggtcaaataactcctaagagtgtggggggtgcctcatattttctgctagtggtagatgatcatagtagatatatgtgggtggagatgctcaaaacaaaagatcaagcatttgaattgttcaaaggcataaaaatgagggctgaagttgaatctggtagaaggctgaaagccatgaggacagacaggggtggggaattcatttcaaatttattctcaaatttctgcagccaagaaggaattaaacactacactactactccatactctccacagcaaaatggggtagtggaaagaagaaaccaaacagtggtggaaatggcaaggtgtttgctgaagacaatgaaagttccttcagtgttttggggagaagctgttagaacagttgtctatcttctgaatagatcaaccactaaagctcttatctcaaagaccccatatgaagcatggcatggtaagaaaccaagtgttggtcatctaaaagtctttggatgtgtagcacatgtaaagctagctggtcctggtttgaataagttagctgacagatcaagaaggatgatgttcattggctatgaatcaggttcaaaaggctatagattttttgatcctgtcacaaataaactggttgttagcagagatgttgttttcgaagaaaacacaccatgggactgggagagtttagtgcttaacacaggtgataaggtgactgaaacatttgttgtagaccatcagttctgtgaccaaactccgacaatatcaactgaaagcaatagtgaagcatcctctgaaccaactgctgaaggggatctctcagcaagccaggggagtgcagtgggacatgatgcagttcccagctcaccacacactccattgacaagtacagctcagactacaggtatggtgtctcctcctgtacagagtcctcaattttcagaaggagtaccattgaggtacagaacactagcagatttgtttgactcaactgaagaaatccaagatttcgagtacagtgggctgtgcatgttggctgccgatgaaccaaaaaattttgagcaagctattgaacaagactgctggaggaatgccatggaagaagagataaagtcaattcatcagaatgagacctgggaagttacagaactgccaaaagatcacaaggctatcggtctgaagtgggtgttcaaggtaaaaagggatgcaaccggaaaggtggtaaaatacaaagccaggctagtggcaaaagggtatgcacaaatccatggagttgattatgatgaggtttttgctcctgttgcaaggctggagacagtgaggttactcctggctttggcagctcaaggtgagtgggaggtacaccacatggatgttaagtctgcatttttaaatggagatcttactgaagaagtgtatgtgcatcagccaccagggtttactgatcccagttctcccaagaaagtactaaaactgaacaaggcattgtatggtctgaagcaggcacctagagcctggaatgctagacttgacaaagagctagtaaaacttgggttcagaagaagtagagaagaacatgctgtctacaaaaggggaaaggatgtcactttattactgataggagtatatgtggatgatcttatcatttgtgggccagacaaaagggagatacacatcttcaaacagcagatgatgaagagtttcagtatgagtgatcttgggttgctgagctattatcttggcattgaagttaaacagagacctggggagattaccatttgccagagtgcctatgctgccaaaatcattgagagttgtggtatgaaggggtgtaatcctgccgatactccaatggaacaacatgttaagctgttgcctgggaagcctgatcaggtactagatgtaaccaggtacagaagcatagttggtagtctgagatacttggtgaacaccaggcctgacttagcttattcagtgggaatagtaagcaggtttatggagactccaaacgctgaacattggtctgccattaagagaattgccaggtatatagctggaacagttgggtatggctgcaagtatttgaaagggaaagattcagacttattgggttactcagacagtgatcatgcaggtgatcttgaaaagaggaagagcaccagtggtgttgtatttttcttgggaaggaacttaatcacttggacctcccagaaacaaagagtggtgtccctgtcctcctgcgagtctgaatatattgcagctgctactgcgacctgtcaaggagtgtggctgagcagattgttgagtgatctgctagatgtaaatgtgaagaagttcagtttactgattgataacagatcagctcaagaactctccaaaaatcctgtctatcatgaaagaagcaaacacattgatacccggtatcattacatccgagagtgcgtcagcaatgggatggtagatgtggctcatgtgagttctgaaggtcagttagccgacgctctgacaaagccacttggccggatgaaattctcagaacttaggaggcaattgggcgtcgtcaagatagaaggttgacaagattaagggggtgaa

>Copia-34_ZM_LTR

tgttgcatttagccattctgtaatcatgtcaatatgttatcagtgcatttagctgcattagctcgggtagttaaattataaatctgtttgtgtaccgcgtagaatcgctggtgtactggagactgcccagtatgcgcagtgaccgcgtccagccctggttttccgccacgccgtgcatgccgtgcaagtcgcgcgagctcccgcttgaaaggggcgtcgtgcagctgtaaggagtggcggggaaaacggatcggggggtttaagaagactgtttcaatgcatgagaggtgctgcgcgaatttgcaagtgcccagaactcattgagtctttctctgtgttgtgcctgttcttgtctctggcggctgaactctcaatcgtcgttgccggtccaaca

>Copia-36_IN

cgtcatcagcacttgcttttacaaaagagagcaaaggccaaaacaagaacagcggaggcaagaagttcttcatcaaaagaaaaacagtcgggtacgcaatgatctacaaagatctttctgttgcccttcttaccttcatccttcggtttttgaacggtggccatcgtcgccggcgaggcgctgaccttgtcgcacgtcgtcttctacgtgctgcgcatcgacatggtcgccaccatgccaagaagcgcagcaagaaggacgcccgggccacacttgaccctgagcgcgtccgccggaccggtggtgatcctggcgctggtcccagcaacgctgcggcacctgctgcaccgccgcagcaaccgccagcgccacgccaagaccgatgggtgcgtcctccatcgccttcgccggagcgggttgcagagcggttgcgtgagtccgagacccagcagccgctccgtccgatgcggtactgccccctccatggatgggggccgtgtcctgctcgccaaccgcgggctcctccaacggatgcagacaccgccgcccccgctgttcgatcgagcggactgctcgttcaagtcacctctccagcgcgccctcgtgcccggacacggctgactgcgcggaaatcaaccggccctcgcggtcgccccgtcggccagatcgcatccacgtccactgcgacggttgccgtcgacgccacctcttcctcctccgacgatgagaggatcgccttagtcctcggcgacggggtgcagcagcgactgcctactccgactcccgacgacggcccatccggaacggtctcaccgtcgacgctcttgagcccgagcggcaacagggtggtccgtcggctggcgcaccacctttctccacggccacgcgcgccggggtcgtggtctgcgacagcaagaccgtccgacgacgacgtccgtgagcgctcgcgctcaccgcctcgtcgtcttctctgagtgtggcctcgcgcgtgctcggccctgcgcgcgcggaccgccccggcagcaggcggcgcggcctcgacagggcccgcggcgcctcgccctggcctgcgcggctcgcccgccacggcagcgggcggcgcggctgctcgcccgmgcccggcagcaggcggcgcggcctcggcagggcccgcggcggctcgcccggcctgygcggctccgcccccgcgcccggcagcaggcggcgcggcctcggcagggcccgcggcggctcgccttggcctgcgcggctccgcccccgcgcccggcagcaggcggcgcggcctcggcagggcccgcggcggctcgccctggcctgcgcggctccgcccgcgcccggcarctggcggcgcggcctcggcaggccgcggcggctcgccttgcctgcgcggctcgcccgccccggcagcacgcggcgcggctgctcgcccgcgcgcggcagctcgcagcccggcctcggcagggcccgcggcggctcgcccgcctccgtcgcgcgcggccgcgcggatgccctgcgccctgaccggcgcggcggcagccctggcctgcgaggtctcyctgcgacggacgaggacggcgcggcctctgcccctggcggctggcgcaggcagcggcggctgatggggcaaaaccctaactgccatcccttaaagagaccgcgcacgcccatagatgggccggcctgcttacccatgtcgtaggcccagttagccattttactttttattttagctattttcagttttctaaattctagaaawtttagattgctttsattaaagatgtttaggaattaagactcagttcttaaatccttcagtagcagcttgcgtcaggaatttttccaaagttttagtacttggaaatgatgtcaataaatattttctttaaatgtgatatttattgccatgtgttctattttgcattccttaaaatgcaaactcaaaaaaaaatattcatctcaattataaccaaaaaggtttaatacgagcagaattaattttttttttgatagaacatatagaataattaatattcatctcaattaagttattcaaaacttaatacgagcaagaatatttatttattyaaaattccatacgcaattcatgctactcatatattcatcttattaagttactacaagtacttaatttcatctgaatattttatttactacatcattaacatcataatgaaattttcgatctcttgcatcactttgcaattgagaccttaaattatcattcatttatgcaatatcgcattatattgcatattattcatctcaattgggtttttctcttaaatccaatacgagcaagaatacatgtatgttgtgaacaacacacatattacaacacacccatgatgatttcttaacgaaattctctggttgcttgtgtaggatcatggcccgcgagttcgaagaacttgctcttgatggtcacaactatcctacctgggcgttggatatcaagatcagcttagcctcaaagaatattctgagtgcattactccctcccaacgagagggttgacccactacatgatgcttataagtacaacgctttgtacattataagacatcacctccatcctgatctgaaagcagaatatgtgatggaagaagagccaaatgtattatggttatctcttaaaaacagatatgagcagcaaaaggctgtaatcttaccagaggcgaatcatgaatggacccatattcgtctgcaagactataagtctattggtgattacaaccatgctgttcataagatctgtgctcgcttgcgtttctgcggcaaggaaccatcagatgcggataagattgaaaaaacacttcaaactatgatcccatctgatagggtcctgcagcatcagtaccgtgctcgcaattatcagacttactctgaactcatacatgatctacttcaggcagaaaagcatgatgagcttacgatgaaaaatcataagcaacgtcgtgttggagctgctcctatgcctgaaatacatcatgctgtgaagaatgagaagaaaggggatggccccaaaagccatcccaagaagtctgataattcaaagaagcgcaaacgcaacaagcgtccggataagcaaaatacaaaggctccgaggaaagacacccccacttccaagcaagagaagtgtaagaagtgtggatgcttcaaccatcctactaagaagtgtcgcattcctcgtcatttagtggaactgtaccagcaatctcttaagggcaaaaatgctgaaggacagggatatgaagctcacttcactaccccacctaacttgaaggatggagctggttgctcaagcatggctacaatggaaccaagcacgaccaatcctccgcttctgaccgacaccgattacatggacttggacaatgccatcatcgaatatgcttcgagtgatgtgtttggagacctcaactaggatccttatgctccaccagatatcatctatctgtagtctatgagtgtgtgcaacatgtgtgttgtaataagtacctagacattggtatttgtgtgtatactccatactctactgtataattatgtatgtacttatatgtataattaattgagttcttgcaattcttgattatatagattatgcggaagtcaatccaatggaggaagaattatgccttgtagacagtggtaccaccaactctattcttcgggaaacaaaatatttccaaactctcacaaagaggtcaggaaatrttttgacaatcgctgggcgcgatgcgaaaatagttggctctggacgagcgactattacgctccctatgggtacacaagtcactatcgaggatgctttattgtatcccgattcaactcgtacccttctgagttttcgagatattcggaaaagtggtttacatatttctactcatacagaaaataatgaggaatttctcctcatttccaaaccttctgaatatggcgctaacattctggaaagaataccttcacttccatccggattgtactacacatacataaaaccagtacctcatgttacgtacaaagtgatttttcagaatgtagattcattcaagacctggcatgcacgtcttggtcaccctggaatagggatgatgcagaaaatcacaggtaattgcatcggtcatgatttaaagtctgccaaattccctaaaccatctgattttatatgtacatcatgtgcaacgggaaagctaatcttaagaccatctcctcttaagattcatgctgaaccactcaagttccttgaacgaattcaaggtgacatttgtggacccatacaaccattatctggaccgttcagatacttcatggtattaattgacgcatctacacgatggtcacacgtgtgccttctgtctacacgtaaccatgcttttgctaaaatcatgacgcaagtcattcgattgaaagcacattatcctgaacatcaaatccagaaaatccgcctagataatgcggctgaattctcttctagagcattcaatgattattgtatggcccaaggaatacacattgaacattctgtgccttatgtacatactcaaaatggtttagctgaatctcttattaagaggatcaaacttatcgcaagacctttacttcatgaatgcaatttacctacatcctgttggggtcatgcagttttacacgctgctgatcttattcaacttcgcccaactgcatatcatacaacatctccgttgcacctggtacgaggaaatcctccaaatatatcccatctgcggaaatttggatgtgcggtctacacccctgtatcaccgcctcagcgaacagctatgggacctcataggaaattgggcatctatgtgggatatcaatccccatcgatcatcaaatacctagagccccttacaggggatttgtacacagctcgtttcgctgattgcatctttaacgaggaccatttcccggcattagggggagaattcaagtatcctaatgaatgccaggaaatcaattgggatgacaaatccatcctgtcctcagatcctcgaacccaggaatctgaacttcaagttcagaaaattatagaattgcaacactttgctaataatctgcctgatgcgtttactgactataatggtgtcacaaaatccttgaatcctgctgttaatgcgcccagtcgagtggaggtaccacgaaaaaccattcaaccatcttctcaaccgaagagggtgagggctgctaagcaggataatgcttctactaagcgtcaaaggaaagagaggaacattcgatcctccaaatcagtaaacgaaagtcaaccaccagttgatggacaccaagtggatactgtacatccacaaaccagcactcatgtgcacaattcagatgttgctggtacatcggaacaccccatatctactcttcctgtgaatcaagaagactttcatggggtacaggagatttccattaattatactagttctggagaattgtatgatcgtaaaactacggtcatcaattcatgtttctcatcaatcatagctggtaaccttctatctgacccagaccctaaatccatggcagagtgtcaacaacgctcggactggatcaaatggaaggaagcaattaactctgaattatcctcactagcaaaaagaaaggtattcacctctgtgatacctacacctcccagaatccatcctgttggattcaaatgggttttcacccggaaacggaatgagaacaatgaggttgtgagatataaagcgagattagttgctcaagggtttacccagagaccaggaattgattacaatgagacatattctcctgttatgaatggaataaccttccggtatttaatttcattggcagtacaaaaacgtctacttctgcagttgatggacgtcgtgaccgcatatctttatgggtcactagattcggacatatacatgaaagttcctgatggaatacctgtaccgaataatggcaaaaatcgtaacatgttttgtgtcaaactcaccaaatctctatatggcttaaaacagtcagggagaatgtggtacaaccgactgaaggaattccttatgaataagggttattctaacagtgatgattgcccatgtgtctttataaagaaatcttctacaggtttctgcatcatatcagtatatgtggatgacttgaacatcatcggccataaacgtgatattgatgaggcacgcaatcttttaaagacggaatttgagatgaaggatttgggtaaaaccaaattttgcttgggtttgcaacttgagcaccttcccacgggcattctcattcatcagtctgcttatgcgcagaaagtattgagtaaattcaatatggataaagcatatccgtctaagactcctatgatcgtccgtaccctagaaagggataaggatccttttcgaccacgagacgaaggagaagaaattttgggtcctgagtacccatatcttagtgcaattggagcacttatgtatctagcaaacaatacaagrcccgatattgcttttgcggtaaatttacttgctagatatagtgcatcccctaccaaacgccattggactggaatcaaaaatattcttcgatacctccatggcacggcagatctcggtctattctacggaagaaatcaagatcctagcctygtagggtacacagatgctggttatctttcagacccccacaatggaaggtctcaaactgggtttgtatttctgaatggtggaactgctatttcttggaagtcatccaagcaaactctagtaacaacatccactaatcattcagagattattgccttgtatgaagcctcacgtgaatgtgtctggcttcgaagggtgattaatcacattcaatcatcatgtgggattcattccattgaatcacccaccattatctatgaagataatgcwgcttgtgttgctcagatgcaacaaggttatatcaagagcaatgttaccaaacatatctcccccaagttattctaccctcatcaacttcaacagagtggtgagattaaggtcatgcaaactaagtcttgtgacaacctcgcagacttattcactaagtcgctaccttattccacatttcggatatgtgttaagggtattggtatgcgtcgacttagagatttgcttgcatcagggggagtatctcttgattaatctaatactgacatcacattgtactcttttcctatatgagttttactaccatgttttctcatgttaggtttttaatgaggcaatgtcaacacaagcatatgtcagtctcttgatttttcccatcagggtttttcgatagagacaccaaagacataccatatactgttgattttctccaaatttttcccactgggtttttctggagtttacacagtatattatcgcaaaccaccttttgcttacaggtttcaagggaggatatacataatggacaactgcttaagggggag

>Copia-36_LTR

tgttatgaaaaataagcagttgctgactgctttctaaccggcgttctccaagggacgtgcccctgcacgatccccatgtacggacgtggcttttggccactcccctttagtgtctccttctcttgtataaatatcaaaagtcaataaagaatgggtgcccctttttcattctctcgcacaacagtttctatttctcaca

>Copia-37_IN

agtggtatcagagccgaggactccattgtttgtggatctaatcatcccggagtgggacaaaatggctagtaacaacaatgtgcacattgggaagccaccgcactttgatggaaacaattatgattattggaaaataagaatgtcaatgcatcttagagcaatgggtggaaaaatttggccaattgttagagatggatttgttgtattgaaggaagatgaaccatccgttagtgataatgagaatatcctcacaaatgatcaagccatgaatgtcttttatgatgctcttgatataaatgagttcaatcgtatcaagaatctcacaaccgctcatgagatttggataaaactaatggaaattcatgaaggaactacaattgtgaagagtgccaaactatatgtgtgcaaagggaagtttgagcaatttattatgaaagaagatgagagtgtatccgatatgttcaatcggttgaatgagatagtaaatgaactcaaaggacttggttttaatgtaccggatgtggatttcacacacaagttccttagatcacttccggagaaatatgagactattgtgacaatgctagtaaggagtgatctatctacaacttctccaaccgaagtattgggagaaattctcactcaagatatatttaagaagtcacaagccgatgctttaagtttggcaaagaaggtgaaaaatgaatctattgctctcaaagccaaggcctcaaaggcaattgaaaaagaagatagcaatgatgaagaaaatgaaagtgagagtgatggtgacatggctttatttgtaaggaaattcaataaattcatgaagatgaaaagaggtcaacctagaagaggtcaaacatctagaagaaatgctttcaatgatagaaaatgttttgagtgtggggaacccggtcacattgccatgaattgcccaagcaagaagaagaagggcaaagatgaaagtgacaagaagaaaaagaaatactataacaagaagaaagatggcaaagcctacttagttgaatgggactcggatgatagctcggatgatgatgatgatgacacctcatccaagcttaatgccggaattgccatcaaggaagctccctcactcttctcatccccccattgtctcatggcaaagggagatgctaaggtaaaaatcatcaccgatttaaatgatataaaagatgatgatgatatcgatgatgttgatgatgatggctattcatatgatgatcttgttagaatgttaggtgaggccgatgactacatgcacaaagaaaaagaaaaatttaagaccttgaaggaattgtataaaaacctccaagtgtcttttgaggagctcaagacctctcacaatgacctcaaagaaaattgtgagaagcttgcggatgctcaaaaatcatctattgtgcatgaagttgaggtggtcactaaggatgttggagtcacttgtgacttacttgatagtcttacaagtgaaccactacctactaactctatttgtgataaatgcaaaatttctctcaatgataacattgctcttgatgaatcaaaaattattgttgagaatgaagtgttagtagatagaataaatactctaactcatgacctagaaaaggcatatggtggtaagactaaattggatttcatattgggaagtcaacgatgctctcttaaccgtgaaggtcttggatatgttcccaagaaaggaaagaatgcttttgtaaagcaaaagacattgtttgtgaaagaatgtgacaaagtgtgtcacaagtgtcacaaaaagggacatgttaagaaaaattgtcccaagttcaaaaatgtatcctccacttgttttgagcattgttatgttctttctcataatgcaaaaggtgttcatgctaaatttgttggtacttcaattgttggaaacaaaaagaaagctatttgggtaccaaagaccttggtcactaacatccaaggacccaagcaagtttgggtacctaaaagagattgatttccttttgtaggtaaactacaaggcgggagggaatcattgggtgttggatagtggatgtactcaacacatgaccggggatatgaagatgtttacccaaatgagtgaggaagattgctcaaattatgatagtatcacatttggagataatcgtaaaggaaaggttaaaggtttgggtaaaattgctatctcaaatgatcattctatatcaaatgtgctattggttgagtctttgaattttaatttactttccgtagctcaattatgtgatttaggattttgttgcaatttcacggttgaagatgttattatctctagtgttgatggtagcaatcttaagtttaaaggttttagacatgaaaatctttatcttgttgatttctcatctaatgaggcaaagctcacaacttgcctattttcaaaagcttcactaggttggttatggcatagaagacttggtcatgttgggatgaagcaactcaatcggttaaccaagcatgacttagttcgaggcttaaaagatgtgaagtttgaaaagaacaaattgtgtagctcttgtcaagccggtaagcaagtggcaaacactcatccaaataaaagtcaaatgtccactcatcgacctttggaattacttcacatggatttatttgggcccacatcttttgtaagtattggtggtaattcttattgtcttgtgattgtggatgactattcaagatttacttgggtttattttctacgagacaaatccaatgtgtttgaaacattcaagtcatttgctatattggctcaaaatcaatttgatttcgacatcaaaaaggttagaagtgataatgggtcggaattcaaaaatgcaagaattgatcaatattgtgatgacaagggtatcaaacatgaattctcttccaaatataccccggaacaaaacggtattgttgaaagaaagaatagaactcttattgatatggcwaggtctatgttagcggaatataatatatcggattcttattgggccgaggcaataaataccgcttgtcattcatcaaatagactatattgtcacaagctcttgaagaaaactccatatgaattgctcattggtagaaagccaaatatctcatattttagggtattcggttgcaaatgttatattttgagaaaggggagccggctttctaaatttgagaagaaatgtgatgagggttttcttcttggatattcatcaaatagtaaggcttatagagtcttcaacaaaactcatggtattattgaggaagcatatgatgttgaattcgatgaaacaaatgggtctcaagatgagagtgataatctcgatgatgttgggggaactcaattgataaatgcaatgaaaacaatggccattggtgaaataaaaccaaaggaagatgatgatgaaaatagtgtggttgtcattccttcatcctcaactataaatgaggaagatcaccaaagccaacaactcaatgaaaccatggatactcatgatcaaggtacttcaagaccttcggttcctcctaatgcttcaacaagcaattatcaaacggtatcaagaattcatcattccattgtgaaggatcacccggttgatcaaattgtgggtgatattagtaagggtgttcaaactcgctctcgcatagcttcgttttgtgaacatttctcttttgtatcttgtatggaacctaaccgtgtagatgaagctctacttgatgttgattgggtgaatgcaatgcatgaagaattaaataacttcgctcgaaatgaagtttgggagcttgttgagagaccaaagaaccacaatgttattggtacaaaatgggtgtttcgcaacaagcacaatgaagatggtgtggtagtaagaaacaaggcaagattagttgcacaaggatatactcaaattgaaggattggattttggggagacatttgctcccgtagcaagattagaagcaatccggattctacttgcttatgcttgtgcacataatatcaagctctaccaaatggatgtaaagagtgccttcctaaatggtaagattagtgaactagtgtatgttgaacaacctcccggttttgaggaccccaaaagacctaaccatgtgttcaagctttctaaagctctctatgggcttaagcaagctccacgcgcttggtatgaaaggcttagggatttcttgctttccaaagacttcaaaattgggaaggttgacactactctattcactaaaagaattggcaaagacttatttgtttgtcaaatctacgttgatgatattatttttggatctactaatgaattattttgtgaggagttcggaaaaatgatgtcaaaagaatttgaaatgtctatgataggagaattgagcttctttcttggccttcaaatcaaacaattgaaagatggaatttttataagtcaatcaaaatatttgaaggacatgctcaagaagtttggtttagaaaatgctaagcctatcaagactccaatggcaacaaatggtcatctagacttagatgaaggaggtactatggttgatcaaaaactttatcgttcaataattggtagtttgctttatattaccgcatctaggcccgatgttatgtttagtgtatgcatgtgtgctcgatttcaagcttctcctagagagattcacttgaaggccgctaaaagaattctaagatatttgaagtatactcccaatattggtctatggtatcccaaaggtgctcaatttgaattaattggatattcggattcggactatgcggggtgcaaagttgatagaaaaagcacctccggttgttgtcaatttcttggtagatctcttgtttcatggtcttctaagaaacaaaattcggttgctctatctaccgccgaggcggaatatatatcggccggaaattgttgtgctcaactattatggatgaaacaaaccttattggactatggaattattttcaagaatgtgcctctcatgtgtgataatgagagtgcggtaaaattggctaccaatccggtccaacactcaagaaccaagcatattgatatacggcatcacttcttaagggatcatgttggcaagggagatatctctatttattccattggcacggatgatcaattagcagacatttttacaaaacctttggatgaaacaagattttgttctctaagaagtgaaatgaatgtgatcgatatctcaaatgtggcttgaagttgatcacacatgtgtcgtattgcatctcgggtctaaatatgctctttatgctatttatttggtattattggtgcatttttcatttctaattgctctagatagtttaattcaaaaattttgcattactcaattacatcatatgtatatgcatgcatactaactcttggattggtcaaaatgtccatatatgagcaccaattcggattcgaaattcaaaatcagaaagtggacagcaattggaaaaatgagtatcaggccgcggaccgtccgcccatggaccgcggaccgtccgcagcatcaggaaatggacagtccgatcagcctcgcagaccgtctgaaatcatcagggcgcggaccgtccgccgccccagcgcggaccgtccgcgacagggcagtaaaatcgggcagtggccgcagacttgtcatttgtgctcattcgctctttctctcttgctctccactcgccaaatactctcctgtgtcgagtgtgcgcggacggtgaagtgaagttgcgcgcggacagtccgacagcttgcggcaacattccatcaacatgtcttcatcacggtatcttcaaatcttgtggatttcatcaaatttcttcaattttgagattatttgggtttcctctctgatctgaaattgagcagtgggtttcaaaatctgattggtgggattgatagttctcctcaatgtgaatgctatgcaaaattttgaacttgtttggatgggtattctctgcaaaatcatcaaattgaacatttgtggcggctagggttctttggagtcgcccctggtcggtcgcggaccgtccgcctggtggacgcggaccgtccgggcctctggcgcggacagtccggccccctagcgcggaccgtccggaccctggcagagcattacagttccattcctttttataagtggtgattggtatcaattatttatctatggatgtctgtcacattgttgcagtggttttggtggtctccgcaagaaacttgcaggtcgctttaaatccaagcgccctcgtggaaatgatgatgactatacaccaaccactgattcagaggcccaatcctcagctgggggcactgaatccatggatgctgaagattttcctcatgttcatcccgattatcccattgatayccaaggatggacttatcccaagcggagattttctatggctgagtactgctctagacggactgtgaaccagtacgctctgccatctgatacaaatatccagtttttccacactcagctgcagtttgatgtcttttggggcaccctggtggataccaattttcataagcatcaggtgattgattggtccttcttgctcagtcaatcagtcatggagggtctcatccctaagtttgaaacatgtggattatatcagtttatggggcaacgcacagatttcaatgaaatggctgttaagcaattcctggctacctcagagattgatattgaagaagaatctatcacctggatgactggcttcaagcgctattctgcttcttttgctgactttgccgctgccaacagtctggactatggcaccatttctgctgggctggatctttatactgaagataattttgaggattttgtgcagttttatgagccagccaggctcggtatacccaggaggtttggagagacagcaggactcagacatcatcctgctgtcatcaacaagattgccagagtcaccattcttcccaagagtggtgataagagtaaaatacgagataagttctggaacatmatccagcacatcatgaatggtgaagtcatgaatattgttcttttcatgatgaggcagcttaatgatttgaaaatggacaagaatcaaaatttggcatatgctccttacattatggctctcatcaaatccaagacaagatttgagggcccatgcgagattgttcacaccccattcaggccttttaagaatgagatagggtttctcaccaggccactcactcccttcccagatgatgaggaagaccctggctatgagggtggagcagctcctaatgttgagggacagcagatgcctcctcctccacctcctcctcagcctcagcactattgggagcctgctccaggatactttgatccttattttcacaatatgcagcaagggcttgagactcatattgatactcgctttgagggcttgatgacacatgtggatcaccgcctcgatgacatgcagtctcgctttgacagtaactgggatgcgctcaactctgaattttctgactttcgtgatcaaattcacactaatgtcactgatcccatcatgtccaggctgaataatatgcaacagagttttcaagataacatgggtgctctctccagtcaatttgacaatctttctacaaatgacaacatgcatgatataagtcagaggcagcagcagctccagcaggattttggccagttctcctccctgtttgacaccttcagcactcattactacaacatgcatcctccgccgagtgatcagtgaggcctctcctttgtggcaattgatgccaaagggggagagaagtggatgagaagttgtctggcgtctccttcagggggagttggtggttctatgtggacattaagaccatgcatatgcattttaccttttccatgccgcttgcattagtttatgtcttacgcatttggaacttggtttgatctattaactctatgtcgtatgtctgtggactattatctgtaatattctgtgggatgaactatgttttagttcaaattactctgtgtgtggttaatcgaggtgtgattataattgctgcgctcactctacggatcatctcttgtatgcctcgataaccatgattgtaggaaagtctccatcaagctccaatatattatgtgtgttatatcttcaacttcaaataatcctgcacacacttagggggagcctttcttacatactgagtttttattgggatttatctatctctcggacagaacttcaaatcaagataagtcctatgaaactcatctccaagatctatcttataccttaggtatgagagagatttggagaaactaaacttctctttaatatactatgtggtgttgtcatcaattaccaaaaagggggagat

>Copia-37_LTR

tgtgaatcatctaggcccctttggtaatgttttggtaattaatgacaactacttgtggactaacgattcttggagaaataagaatgcagggttggaccacatagaacaggaaatgttgaagagtcatacgcattggttgtggatcagatgaaggcaaaggtataatataggttttacttttgccggtcttgaggagtttagagaagatacctgaccggattagtaggctagatagccgtactattaagaggggtcaatgacttagatctgtgtaaaacttagtgcctcatagagcatcaagtagttgcatttgcatgaggactaacagcgcttctcatttcgcgagttaaaagttcwttcaaaagcatgtttgaaaattgcgaagtcwtggwcgcgcggactgtccggcccttgggggcggaccgtccgcgatcctccagaggggtccgaagtctgaccatttgtgttgacactgtgttctattgcactgcggaccgtccgggccttagggccggaccgtccgcagtcctgaccagaggaaggtggcttcgggccagtccctgaattataggcggacggtccgcctgtgaggggcggacggtccgcaagtgtcaaactcgtttttggacagggactgtgtgtttttatagatttgtactacggacggtccgggggaccagtccggacagtactgtctcaggtcgcggacggtccgggatttatagccggacggtccgcgtgtgttaactccgtttgatccgaggctcgggtgtttgaatttgccaggtcgcggacggtccggccttgaagggcggactgtccggacccaccttttgagtcagctctgacatgtttcaaacggtcattatagccgttatggtgtacggcggaccgtccggccctagggcgcggacggtccgcgtgtgcgcagaactgcccccttttgcacataacggttggttttagatgggggactataaatagaagggtagctcgtgtgagagasctctcttggccattccttgcacacattgagctcatttgtgatcctccaactcactctctcacactctttgcttgagattgcattctagtgagagattgagggttcctagtgcatttgcatcatttggtgattcttgaggcactaggtggtacaccgagcaagcgtcgttggcttgttactcttggaggttgccgcctcctagacggctcgggtgattgtctccgtcgagctctccaagaagattgtggagaagccgcggtgttgattgtgaggggttcgcgcctacctcgccggagcggcaaaggtgacattagtggaatcgaggtattgagtgatttcttgtccacttggctcaaagatcaagtcgtgtcttgatagaggagcaagtgagagcttgaagtccacctcaacgtggattaggggtgatcggcaaatcaccgataccacgggataaatttcggtgtctattccttctagcattacttattgccttgcaattgattagtgttttgcttattgttcttcaagtattcaatctccgtagttgtctttcatacattgtttacttattgacactagtaaatttattcctcttgttgtattgattaaatttacttagtattgttgtttttagtcaaaaccgtctattcaccccccctctagccggtgtcctagatcctaca

>Copia-38_IN

atccaaaaaccttattgtagacaatttcctagtttttctatggctgcttttgccgacgcgctgaagccagaaaagtttaatggtatgcactttaagagatggcaagtcaaggccacgctctggcttactgctatgaatgtcttccatgttagtaaaggcagacctgagggtccactgactcctgaacaggagaaagagtacgaccatgccaatactatgttcacgggagccgttcttagcgcccttgttgaccgtctggttgatgcgaatatgcagtacacagacgggaaagagttgtgggatgcacttactactaagtatggtgcatcagatgctggcagtgacctgtatatcatggagagctttcatgattataagatggttgataatcgctctattgtagagcaagctcatgaaatacagtgtatagccaaggagctcgaccaccttaagatagtccttcctgaccgatttgtggctgggtgcattattgcaaagttgccttctacatggaggaacttcgccacagctctgaaacataagagacaggagatatcagttgaaaatctgatagcgtctctggatgttgaggagaaagctcgggctaaggacacgggatctaaaggaggcgagggccactccagcgccaacatggttcarargaaccacaacaagggcaaaggaaagccaaaatctaacaagcccaacaaaactaccaacttcaagaagaagaagaacaaggctgaattgacatgtttcgcatgtggcgaggcgggtcattttgccaaggattgtcccgatcgagcggatcgccgtggcaaaaagggcaatgtcaacacagtggtcgctagcaatgaggaagacaaagggtatggtaatttacctttyatcttctcagtatttcaatcacctagctggtggcttgatactggtgctaatgttcatgtgtgttctgacatyaacttgttctcttcttatcagggcgcccgggattcttccgtgctaatggggaatgggtcacatgcttctgttcatggcactggcacggtggatctgaagtttacttcgggaaagatcgtgcagctgaagaacgtgcatcatgtcccttctatacacaagaatctcgttagcggaacccttctatgtagagatgggttcaaggtagttttggagtccaataaattagttgtgtccaagtctggacaatttattggtaaaggctatgattgcggaggcttgttccgcttttctttgttagatttcaataataagtctgtgaaccatatttgtgctaatgttgatgatcttgcgagtatttggcattctcgtttgtgtcatattaattttggctctatgtctcggcttgcaaccatgagtttaattccgaatatcaccatagtcaaaggttctaagtgccatagttgtgtgcagtcgaagcaacctcgaaagcctcataaggctgctgaggagagacacctggcaccactagaactcatacattctgatctttgtgagatgaatggtgtgttgacaaaaggtggtaagagatacttcatgacattgattgatgatgcgtctagattttgctatgtatacttgctaaaaactaaagatgaggctttagactactttaaaatctataaggctgargttgaaaaccaactagagagaaagatcaaacgtcttagatcagatcgtggtggcgagttctttcccaaagtctttgaygatttctgtgcagaacatggcattattcatgagaggactcctccctattcacccgagtcaaacgggattgctgaaaggaaaaaccgtacgttgactgacctggtgaatgccatgttagacacttgtggtttatctaaggcatggtggggggaggcagtcctgacttcatgtcatgttctgaatagaattcctatgggcaaagaagagaaaaccccttatgagaagtgggttgggagaaaaccatcactttcatacttgcgcacttgggggtgcatggcgaaagtcaatgtaccaattaataaaaagcgcaagcttggtccaaggacagtggattgtgtctttcttggatatgcttcgtgtagcatagcatatagatttttagtagttaaatctgaagttcctgatgtgtatgttgatactattatggaatcacgtgatgctactttctttgarcatatatttccaatgaaagacattcatagcaattctagatactcttctgagataaytcctgaacatartacacctattgagagttttgaacagccacatgaaattgtcctagaggaggatgacaatgatgctcctaaaaggagcaagagacaaagggttgaaaaatcctttggtaatgatttcattgtgtaccttgtggacgatactcctactaccattgcagaagcatttgcatctccagatgcagatgattggaaagaagcagttcataatgagatggactccattctttcaaatggtacgtgggaagtcactgatcgaccctatggatgcaaacctgtgggttgtaagtgggtgtttaaaaagaagctcaagcctgatggtacaattgaaaagtacaaggctaggcttgtggctaaaggctatactcagaaagaaggagaagacttctttgatacttactcacctgttgctagaatgaccactattcgagtactactttctttggctgcctcgtatggtctccttgttcatcagatggatgtaaagacagcttttcttaatggagagctggacgaggaaatctatatggaacagcctgatggatttgtagtaaagggtcaagaaagcaaggtgtgcaagttattgaaatctttgtatggtctgaagcaagcaccaaagcagtggcatgagaagtttgacactactctaacgtctgcaggctttgccattaatgaggcagacaggtgtgtatattatcgctgtggtgggggcgaaggagttatattgtgcttatatgttgatgatatattgatatttggcacaaacattgatgtgatcaatgaagtcaagtcttttctatcaaagagttttgatatgaaagatctgggagaagctgatgtgattctaaacatcaagctgattaaggcagatggtgggattactctctcgcaatctcactatgttgaaaaggttttgaagcgatttggcttctctgagtgcaaaccttctccaacaccttatgatcccagtgtgacactgcgaaagaacaagagaattggtttagaccaattgagatactctcagattgtcggttcactcatgtatcttgctggtgcaacaaggcccgatatctcgtttgctgtgagcaaattgagtaggttcatgtcaaaccccgggactgatcattggcatgcacttgagcgggttatgcgctacctgcaaggtacaatgagttatggaattcactattctggtcagcatgcagtacttgaaggatatagtgattcgaactggatatctgatgcagacgagctttatgccaccagtggttatgtctttactattggtggaggtgcggtatcatggaggtcatgcaagcagaccattttgacgaggtcaaccatggaagccgagctagctgcacttgacacagcaaccgttgaggcagaatggttgcgtgaactcttgatggacttgccggtggttgagaaaccaataccagctatccttatgaactgtgacaatcagacagtgattgctaaagtgacgagttctaaggataatggaaagtcatcaagacatgtcaaaagacgattgaagtctgtcagaaagttgagaaactccggagttataagtgtgacttatatttcaacagataaaaatctggcagatccttttaccaagggactaccacgtaatgtgatagaaatcgcatcgagagagatgggtatgagacccgaataaagttgccatggtggaaacccagtctatgtgatcggagatcccgtgaattaggtcctgggaagaacaagccattggtgaactgaggagagtaacctttgaccctctctaagtaaagatgcaatactctcaaatgctgtaaggcaggttggctttgtgccttaatgtgttctgttggcttgtattagcgaagatgttgtcctgcagaacattctttgaaagaacacacctatatgagttagactgtctaacgtcgcagtctatgagatttgggtgatctctagtaaactcatgaagagaccttggagtacgacgtatatgctccacccgagaaggggactactggtagccaagtactggtcatgacttcaagtgaaacccattcacgcaaaacttgcaattcaaggcatagtccattgtccaagttgtgggttggtgtarcttggagttctaggcggaagttcaacttaacagtctctgctgaaaaactagtatattaaacagtagtgaacagtggcgaaaactgcagatgggcatttgagatctggtgggggat

>Copia-38_LTR

tgttaggatttatgggcttggcccaattaagawattcwaataaatcccaggaaaatctcaaaagcccatayaagtggatggcwargggataggtggaaccaatagcaccatattgctagctctwgtggagtagagctagcttaaatatggaagccacactcactcaccaagtcatggatgagaggagagagtgtggagagccacacgcgcgcgcgcgctcgctcgcctcgcctsgcctggcctggcckggccggggcgaagggcgcgggcgcacgacatgcgcgtgaatggtccgccraaatccggcccctcgccttgcgggggcgcggctaccttttgccgtttgattttttggtttcttggctgttacgcttatcctaaccgatcgctataaaatctcaactgattgcgagattttcgtgggtggataagcacggggggtcgcggactcggccctataaaaggagcccggcagccagcctccaaatcatcccagatcccagttcgctttcgcctctcttcatagctgagccgccttttagttcccttcgtcccgaccgcagaggtgcatctgcgatcaggagagcaggtctccggaacccttcgtcttctagatcctgcaccgggagagggcgaataaggtttttgggaagcgtcttcacgcgactgctcgtgatcttctgacctcgtcgaccctgctgatttcgctacttcgacatcgtcgaccctgctgattctggcgcgcgccatctatcagtaagtctaatcagtacgcatcatctgatttggctttttatttcagttcttctgatttggtcatgatttatattcggaatttaatytggaatttgtctaattattcaaca

>Copia-39_IN

atccaaaaatctgatattaggcatttttctgttagtggttttgctgctgcgttaaaaccaagtgaaccttttgatgggacgttttacaagagatggcgtagtaagatgatactgtggttgaccgcaatgaactgctatcacgccgcacaggggaaacccgaacagttcactcctgaagaggagaaaatgttcgacgttgccgataacctgtttcgaggcgccgtgattggcgctcttgccaacaagtatgttgattcttatctaacgtgcacatctgcaaaagagttatgggatgcattagatgagaagtttggtgtttctgatgctggtagcgagctgtacatcatggagcagctatttgactataagatggtggaaaaccgtcctgtagttgaacaggctcatgaaatacaggcactggctaaagaactcgaacaattcccatgtgtcttgcctgacaagttcgtggccggcggtattatcgctaaactgccaccttcttggacggactttgctaccactctaaaacataagagacaagagtttagcgtggctgagcttattggttctcttgatgttgaggagagggcgagagcaaaagacactcgtggaaaaggagttgagacttctagtgccaatatggtacaaaagaagaactccaatgcatcacataataataaaaagaagaacaagcaacagaatgccacgaagcccaagcaggcagcctcgttcaaaaagaagaacaaaggagctggttgctttgtttgcgggagtactgatcattgggcaagcgcttgtccagaccgcaaatttaagcaagagaaaaaaccagctcaagagaagaaaacagtaaacatggttgttagcgagactgcagaaggaacatcggggtatggtaatcttttacctactgttctttcagtgtgtcaatcccctgagtggtgggctgataccggtgctaatattcatgtgtgtgctgatatttctttattttcttcttatcagtgcaaagggactggagccttgctgatggggaacggatcacatgcgcgtgttcttggtgttggtacagtcattctgaagtttacttcgggaaagacggtgctattgaagaacgtgcagcatgtcccctccatcaaaaagaatctagttagtggctctcaattgtgtcgagatggctacaaaattgtctttgagtctaataaatgtatactgtctaagtatggaacgtttgttggaaaaggctatgacagcggaggcttgttccgcttatctttgcatgatgcgtgtaataagtctgtgaacaatgttgtttcgaatgagtcgtatatttggcattcacgactttgtcatatcaattttggttgtgtctcgcggttagcagatttaaatttaatcccgaaatttgatttagtcaaaggttctaagtgccaggtgtgcgtgcaatctaagcaacctcgcaagcctcacaaggctgcggaggcgaggaatttggcaccactagacttaatacattccgatttatgtgagatgaacggaatattgactaaaggaggcaagcgatattttattacttttatcgatgactctactagattttgttatgtgtatctcttaaaatcaaaagatgaagctttgcattattttaagacctacaaagctgaagttgaaaatcaactcgagaggaaaattaaacggttaaggtctgatcgtggtggagaatatttttcgggtgatttttctgatttttgtgtggaacatggtattattcatgagaggacaccgccatactcaccacaatccaatggggttgctgaaagaaagaaccgtactctaactgatttggttaacgccatgttagagacttcagggctatctaaggaatggtggggtgaggcgatcttgacggcgtgtcatgtcctgaataaagttcccacaaagaacaaagaaatcacaccattcgaggaatgggaaaagaagaaattaaatatctcctatttgcgcacctggggttgtttggctaaagtgaatgtgccaattaacaagaagcgcaaattaggaccgaaaactgttgattgtgttttccttgggtatgctttccacagcattggatataggtttttaattataaactctggagtaccggacatgttggttggtacaattatggagtccagagatgctacgttttttgaggacgaatttcccatgaaagctacacatgatacgtctaatgatgaaccaacgataccccatgagcattttattccggtagaacacactgaggaatcccatatacataatcatgtggaaaatgacaatgtatcaactcgaaagagtaagagaccaaggattgcaaagtcctttggtgatgattacattgtatatcttgtggatgacacaccaagtaccattgaagaggcatattcctctcctgatgctgacttttggaaggaagcaataaggagtgagatggattctattatgtctaatgcaacttgggaggtagttgagcgtccttatgggtgtaagcccattggaagtaaatgggtgttcaagaaaaaacttaggcctgatggtactattgaaaggtacaaggcgaggcttgtaattaaaggctattcacagaaggaaggtgaggatttctttgatacttattcacctgtggctcgattgaccacaattcgtgtgctactttctttggctgcctctcatggtctactcgtccatcaaatggatgttaagacagcattcctaaatggagagctagatgaggaaatttacatggagcagccagctgggtttgtagcaaacggtcaagaaggcatggtgtgtaaattattgaaatctttatatggcctaaaacaagcacctaagcaatggcatgaaaagttcgataaaactttgacatctgccggttttgttgtgaacgaagcagacaagtgtgtatactatcggtatggtgggggcgagggagtaattttatgcctatatgttgatgacattctaatcttggggacgagtcttgatgtgattaaagagacaaaagactttctgtctaataattttgaaatgaaagatttgggagaagctgatgttattcttaacattaagctactgagagaaggcattggtgggatcacacttgtgcaatcccattatgtggaaaaggttttgagtcgctttggttttagcgaatgtgaacctgctccaacgccttatgatcctagtaagctattaaagaaaaatcgaaggatagctagggatcaattgagatattcccaaataattggttcactcatgtatttagctagcgctacgaggcctgacatctcatttgctgtgagcaaacttagtcgatttgtttcaaatccgggagatgatcactggcgtgctcttgagagagttttgcgctatctaaagggtactatgagtttaggcatccattataccgggtacccaacagtgctggagggttattgtgatgcaaactggatatctgatgctgatgagatatatgccacaagtggatatgtgttttcacttggaggtggtgctgtttcatggaagtcttgcaagcagaccatcttaacgaggtcgactatggaagcagaactcacagcattagataccgcttcagttgaggctgagtggcttcgtgaactccttatggatttaccggtggttgaaaaacctgtgccggctatttccatgaactgtgataatcagactgtgataattaagataaacagttctaaggataatatgaagtcgacaaggcacataaagaggcgtttgaaatctgtcaggaaattgagaaactccggagtaatagcgttggattatgtccatacgtctaaaaatctggcagatcaatttactaaggggctatcacgtagtgtgatagatagtgcatcaagtgagatgggcatgagacccacctaaagtttatcatagtggtaacctgttctatgtgatcggagatcccgtgaattagaatggtgaaacaagctagtggtagactgagaggaaagacccttaataaggctcatttcagatgcatatctttccttactgtaaggtaggttggtgtttacaccttaatatgttccaagtggctttgtgaagcaaagatgttgtcctacagaacatctttagaggaacatacctatatgggttaactgctagtcacagtttatgagatctgggtggtttctagatacccatgaaaggctatggagtttgacttatatgctccaaccagaggggatgcgttcagcaatctagtactggtaaagagtttagatgaaactcattccacgcaaaactgccaattcaaggcctagtccattgtgcagttgtggtcaagtgtagtctaagttctaggtggatgttcaacttaacagtctccatcgaaacaccagtatatcaaacgtttgagatgatgagagcttttttgtgtgacttagcatttggtggggat

>Copia-39_LTR

tgttggattaatgtgggcttggcccaaattaatattcaataatagtcaatgctaatggcccactttaatgctatggtgtactaattatttagtaccatattggaagttcaaaggacaaatcaatcaacttaaataggtggaccattggtgcatctattgagaagttgagaaaaggatgaaggactgccacacgcgcgcgcgcgccgccgccgccgccgccggccgggccgggccgggccgtggccgtggccgtggccgtggccgtggctcgtggctcgtggctcgtggctcgtggctcgtggctcgtggctcgtggtagatcggaccttggtccgaatattcctttcaaacggttgcgcattttgcctggagtgatgaccgtcatgataaccgtccgtttcctgtcttatggctagtaacggacgtcagttactgtcgtcagtttccagctctaatgcgcgaccgtttctgtccgttgtccttctcccttcttctgaccgcctataagaatggagagggagggctcttccagtcacgcgaattatctcacgcgaattgcaaacaacacattcccgtcccatcttctgcgagcacagagagagtgggagagcaggcctccgaaatcaccgaccgcagagatacacttgcacgggtgtgcgggcgatcagatttttggggagcgtcttcgcgactgctcgcgtgatcgtccacagcttgctgttcgtcgccttcccaagttgacgcgtgctgctgttcttcttcccggcgaccgttcgagggactgcactgcgtacatcttcctgcaccgacttcgtacggctacatcgaacaaacacacgagatgtctcgtgtgaatggagccactggtgccttgagcatcggtccctccgctgggtacactctgttcttcgtatttatgcatgtttcattgctgtttactgcttatgcgagtagttatacacacatgcacatacatgtcatcacatatatcgcactgattatctggattaaattaaaactaaaaatgcctaactttctaaca

>Copia-40_ZM_IN

tctggtatcagagcgcggtttatgttctacggtggatctgtctgtccaccgcgagaaccccaaggaagaagttcgtcgttccttggagatcgcctgtgaaggatcaggagatgggtgacaccaggaccagttcaagtgcggcgtcgtccgacatcaaggagagttccctggtatggcctatgttgacttgatcgaattatgcagagtgggccatgttaatgcagattaactatgaggcaatggaaatctgggatgtcattgatccgggaaccactgtcaagcgctcgcaagatcgataggcaatgggtgctctgatgagatctgtgccaaaggagatgtggggaacccttggcgccaaaaggacagtgaaggaggcatgggaggctgtgatgaacatgaggattggagccgatcgagttaaagaggtaaacgtgcagaaacttttaaaagaatttgaaaatattgagttcaaagatggagagagtgttgaggattttgggatgagaataacaaatcttgttgccaacatcaagtcacttggggaatctgttgatgacactcgggtagtaaagaagtttctgcgagttgtaccacctcgttttaatcaggttgcagtctcaattgaaatgttctgtgacctgaagacactcacagttgaagacttggttggacgcttgcgtgcagctgaggatcggtttgaagacaagcttgagcaagtaactgacaaaactggtcgactgttgttagctgaggaggactggcttgaaaaacataagcatcggtttcagtcttactctcaaaaggatggtggcagtggtagtcagtggaaaggtaagggacctcatcgttctgatggtggcagttcaagcaattcaaacaagccaaagctgacatcaaaggggacaccaagaagaaaggggagatgtcgaaattgtggcatttatggccactgggcacaggactgtaaacgtccaaagaaggatgacaaggatcaaaaacaacaggaagcaaatgttgtagttggagatgtggagaatgcagcacttctattagctgaggtgcaaggggcagtgagtgaagcatctcaagtaattcatctgtctgaagataaagtagtgccagtaatctgcccagatggagtgtgggtactagacactggtgccagtaaccacatgacaggcaccaagtgtgcactttctcaacttgatgacactgtcagtgggtcagtgagatttggagatggttctactgtcagaatctgtggattaggctcagtggtgatgaatactagacagggagatcacaaggtgcttacaagtgtgtactatattccacaattgaaaagcaacataattagtcttggacagctagaggaggcagggtgtgacattagactgtttgctggcagactgaaagtttttgatccagagtataacctgttggtcagtgcacctcgcactggaaacagactttacacagtgaacttgggagttgtacctccagtgtgtctgctgtcaaaggcagatgatgtagcatggttgtggcatgcaagatttggccatctaaattttagagctcttagagatcttggaaagaagagtatggttgaaggaatgcctattgtagaccgagctgaacaggtgtgtgatggttgtactcttgggaagcaacatcgaacaccttttccaaggctgtcctcatatagagccacaaaggggttagagttattccatgctgatctgtgtggacagatcagtccacagacagtaggtggaaaaagttactttttgctagtggttgatgacttcagcaggtttatgtggattgagttgttgaagacaaaagatgaagctctacatttcttgaagaagatcaagcaaagggctgaagttgatcaggagggcagattgaagggcataagaaccgatggaggtggtgagttcaattcaagtatgtttactgccttttgcactgaacaaggcatcaagcacttcacgaccactccttactctccccaacaaaatggggtagtggagcgtagaaaccagactgtagtggagatggcaaggtgcatgatgaagagtaaaggggtgccatcaagattctggggtgaggctgtgacaacagcagtctacttgttgaatagatcccctaccaagagtgtacagggaagaacaccatatgaagcttggtttggcaagaaacccagtgttcagcaccttaaaacatttggctgtattgctcatgtgaagaaaattggtccaggggtaaacaaattgtcagataggtccactaagatggtcctgttgggttatgagtcaggtacaaaagggtatagattgtttgatcctatcactgagaagctgtgtatcagcagagatgtagtgtttgaagaaagtgaaagatgggactgggagagtgcagaaaagaatcagcagactgaaactacaattcctttcactacacagtttcagtttactgtgccagatcagacaatacaagcaccagctggttcaggtgatacgagtgagtcttcagggggtgctgctgcgtcaccaccctcgccacaaactcctatgacaccttcagtgtcccagaatcaatcccaagctgcatctgctgcctcaggttcaaataatagctctcagtcagtgaatgcatgaagttcagacatggttcctttgagatacagaacaatatctgacttattagactcaacacaagaagttcatgattttgagtacagtgctttttgtttactagctgcagatgagccaaaaaatgttgaaagtgccttgacagatcagtgctggagacaggccatgaattcagaacttgactctattgaacaaaatcacacttgggtgtgggcagatctgccaaaagaacaaaaagcaattggtttgaagtgggttttcaaagtcaaaagagatgctgctggaaatatagtgaaacacaaagcaagacttgttgctaaggggtatcctcaaaaggagggtgttgattttgatgaagtatttgctcctgtggcaaggtttgaaacaataagagttttgatagctcttgcagcataggggaattgggaaattcaccacatggatgtaaaatcagcattcctaaatggagaccttcaagaagaagtatatgtcactcaacctccaggtttccaagatacaaagaaaccaggcaaggtactcagactgaacaaagcactatatgggctgaaacaggcacctagggcttggaacatcaggcttgattcagaactggtgtccctgggattcagaaaatgtgaagtagaacatgcagtttacagaagaggaactggcaaaaccatgctggttgtgggagtatatgtggatgatctaatcatctgtgggccttctgtaagtagcattgcagagttcaagcaacaaatgatgaagacattcaatatgattgatttgggactgctcagctactacttgggaatggaggtgaagcaaggagaaaatagtatcacaatctgtcaaaaatcctatgcaaccaagattctagagcagtgcaacatgacttcctgtaatcacactgacactcctatggagcagcgagtaaagctcagtactgctcagaaagggactgaatatgatgagaccagatatagaagtgttgttgggagtctgaggtacctggtgaatactagacctgatctagcttttgcagttggtctggtaagcagattcatggaggccccaggtaaagaacactggactgcagtgaagaaaattctcagatatgtggcaggtacacttaacctgggagtgaatttcaagaagggggcagcagcagattatacattgcttggctacacagacagtgattgctcaggtgacttagtgcacagaaaaagtacctcaggtattctattcttcttgggagataacttggtgacttggtcctctcagaagcaaagggttgtagccctgtcatcatgtgaagctgaatatgtggctgctgcattgggagcttgtcaaggtgtatggctgagaatgctgatagcagacatcatggataagaatcctcagaagttcagactgttaatagataacatgtctacaattgagctgagcaaaaaccctgtgtatcatgacagaagcaaacacatcaatacacgatatcactatattcgtgactgcattgacaaaggcatagtggatgtggagcatgtgggaactgaacaccaacttgcagacatcctgacaaagcctttgagccgaatcagacttgtggaactgagatcaagactagggatggttcaagtccaacaagattaagggggtgata

>Copia-40_ZM_LTR

tgttagtctgaataaggtttgtcacgtgtaatctagttggaacatttgtgtttacgttatctagtcttagtggcgtagctttgtgggtgatcagtgatgtgcctggcaccacaaaccgctttgctgtttgttgaccccgtcgcactcgcgctgcgcatgcacatgatctgcgtgatccgaaaatggtggagatcatggggcacgatggcgacggggtggagggctttcaagcctgtgtaagcactgttcatttattcagtgaaaaaacccagaaaagctgcggcacattgcgcaggcaaaaatcatctgttctaacctgtgtgtgtccgcgcgctcagccttcctcgctctcgccgcagtcaccgcgcctgtctgcgttcaccgtcgcggctatactcgtcgcttgttgatccggccggggatccaaca

>Copia-41_ZM_IN

atctaatcttttaacttggtatcgcagagccaagatccattcttccttccatcctccctcgccatgtcaacaaccactacacacagcagcttctcaggcgttacctctgctccttccctcagcaccgccatcaccatccgtctctcccgcaccaattttttcctgtggaaagctcaagtcacgccgatccttcgtgctcatcagttgttcggccatgttgatagctcgcttccaccaccgactccggtcatcaccaccggaactggagccgatgctcttcaggttcccaacccagattaccttcgttgtttcgcctttgatcaactcattctttctgcactccttgcatccatgtctgaagaaatgctaggccaaatgacccagcacaccagtgctgttgctgcctggtctgcgttgcacgctatgttctcctcccaaaatcgtgcccagatcatgcaggttcgctatcagctttccaacgccaagaaggcagatctcacagccgctgcgtacttccagaagatgaaggggtatgctgacaccatggcttctttgggtcaccccctttctgatgaagaagtcctcggctacatgttggccgggttgggctctgattttgagtccttcgtcacgactgttgccctgcgtgatacatcgatggatctcaataccttcttcgctcatcttctgagtgccgaagctcgtctccaacgctacacctccactggtgagatccattcctcggcgaatgctgctactcgccatcccttcaacactcgtagtggtggccatggtggccagaaccgtggacgaggcggttctggccgtggcagcggtggcggcggtcacaactctggacgtggcggtagcggacctaagccaacatgtcaggtatgcaacatatatggtcatgatgcgctacactgcaggcagcgtttcaatcatgcgtatcagcctgacgatactcgtgagcgtcaagtgaatgccgctgtgactccttcctactcggtcgacacaaattggtatctggacagtggtgccaatgatcatctgaccaacgaccttgatcaactcagtctacatgaacgttacactggcaaagacatagttcaggtggctaacggatcaggtttgtctattgctcatattggtcactcattaatacctggttcctcacgtccactctatcttcacaatattttacatgttcctagtgttcgtaagaatcttctgtccattcaaaaattagctcatgataatgatgcatttgtggaacttcacccatcctttttctgtatcaaggatcaaaaatcacggagaacacttctacgcggtaaaagcttcaatggtctgtacccggtgccatgctctaaatcttcatcacctatgtcccgccatgctgctctgtccagccactcagtttcctctgatctgtggcactgttggcttggtcatccatcctttagcgtcgtcgactttattgtcaagtccaataagttagcgtgtacaccgcgtcagtcatctagagtctgtgatccttatcaacgcgccaaggctcaccaattgccatttcaaaaatcaaagcatgtaacctctccccctcttgaactagtccactccgatgtttgggggccagctgttacatccgttggtggttttaaatattatgttagtttccttgatgatttcagtcgtttcacatggatttatcttctcaaacgaaaatctgatgttgagcatgcctttcatctattttaagcgcatgctgaacgccagcttaattgcaaactccgcactcttcagtccgattggggaggcgaattccgtcgcctctctcaacacattacacgacagggcattcatcatagaattacctgtccacatacctcccaacaaaacggcgtagcagaacgcaagcacagacacagtcgaaactggtcttactcttctcgccaactcgtcccttcctatacggttttgggatgaagccttccttactgcatgctatctcatcaatcgcatgcccacccgtgtgcttcagaattcttcccctatggaagctttgtttcatgagtcacctaactacaattttttgcgtatatttggttgtgcctgttggccaaacttacgcccctacaacagtcaaaaactagctttccgatccacccagtgtgtgtttctgggctatagctctatgcacaaaggctacaaatgtttagatcgatcttccggccgaatttatatttctcgagatgttgtgttcgatgaagtcgtttttccttttgcctcatcttccacaccctctgattctatctccacttcttccttgtttccttctgaagaaccagctatttccaatgaccatatgcagcattatagacttgatttattgctacctaatggctcgctcgagcctccttttgccactgtttcaggttctacaggtagtcgctgttcagatgacccacctgctgctgtcgatcaacactccagtgcaactgacgatgttccaccaccacacactgagcagcattccatttcaccaacaaattgcttggcttcaccatctgctgcgcctgcaccccctaaagttgtggcagaagagtcaacttttgtatccattccatctggtattgatgctgcagctccacaaatggtaactcgatccaagcataatattcacaagcctcggattcatgctgatggaactatcctttataatccaagtcgacgggctttctttactacaccctcgtcatataaaacagccctggctgatgacaaatggtgtcaggctatgcagaatgaatttctagcccttcggcagaataacacttggactctggttcctaaaccccctggtcaaaatattatcagttgtaagtgggtttttcgtgtcaaacaacatcatgatggctccattgacaaattaaaagctcggctcgttgctcggggattcacacagcagtatggcatagattaccatgaaacttttagtccggttgttaaaccggctactgttcgtctcgttctttcccttgcagtctctcgtggatggcacaccaggcaaattgacatcagcaatgctttccttcatggttttcttgatgacacaacatacatgcaacagcctccgggttttcaagatgcctctcgacctgactatgtttgtaagcttcacaagcccatttatggtctgaaacagtcccctagggcgtggtactctcgacttagtgaacgcctttaccagcttggtttttcttcttcagttgctgattcttctttgtttatctacttggccaacgacatcaccatgttcatgcttgtgtacgttgatgatatcgtcattaccagctcctctcctgaagcaacaaggcaattactgcagcaactctcagtctcttttcctgtgaaggatctaggtcagttaaattactttcttggtattgaagtgacttacaattctgggggcatgacactcactcaacagaaatatgctagagatattcttagtcgagttcatatggaaaactgtaagtcagctgccactcctctttgtgccactgaaaaattatccagagcaattgggaaacctcttacagacaaggatgcttttgtctatcggagtacagtgggggctcttcaataccttactctcacacgacctgacttgtcattctcagttaacaaagtatgtcaattccctctccaaacccacggtcctgcactgggaggctgtcaagcgcatactccggtttgtaaaaggaactttgaatactggattgtcattacgaccatcaagatctagtttactaagcatcttcaccgatgcagactgggcgggatgcattgatgatcggcgttcaattggaggctatgccattttctttgggccaaaccttatctcatggaatgctcgcaagcaaccaactgtatcacgatcttctacagaggccgagtataaggctttagcaaatggtgcagctgaagcaacgtggatacaatcattgctgaaggaattgcatgttatgcagtcaagaccgcctgttttgtggtgtgaaaatttgggtgctacatacttgagttctaatcctgtgttccatgctcgtaccaagcatatagaagtggatttccattttgttcgagagaaggttgcattaggaaatctggatgttcgacatattgcttcaggagatcagattgcagatatcttcaccaagcgagctactaaacaaatgttgcagcgactaagacccactctcaaccttgtggctaccggttaagattgcggggggc

>Copia-41_ZM_LTR

tgttagcgtgtatcaagatatgtaacaacatcctagtttatagctatatcagtttagattagcatgtaaacacatagtctgttacgggggcttatccctgaaattatgctgagcttatcctgtaaatcgtacctataagtaatcaagtcatgccaccagaatgaggtgagcagccca

>Copia-42_ZM_IN

tggtaccagagccaaggatattctgactacccaccacaaatttttctcctcctcctcctctccacctccatctttccatggcttcacctgtccccatttcccaagccgtcacaatcagactgactaaggccaattatctcctatggcgtgctcaagccctcccctacctccggagctcaaagctgatggggtttcttgatggctccaaacctgcgccggctacaactgtcgtcgcatccacggttgacggcgccacgccaatacccaatccggagtacgaccgctggttcgatcaggaccagcaactactcagtggtctcctctcgacgatgactgaggatgttcttcgggatgtcgttctcgccaaaacttctaaggaggtatgggactccctccagaagaaatttgcctcatccacgaaggcgcgcacggtgcaaatccgtgtcgaactcgcaactctcaaaaagcgtgatctttctgctgccgacttcttccacaagatcatggggctcgccaacgagctcgctgccgccgatgctccactttgcgacgaagaggtgctcgcttatttgcttgcaggtcttccagtcgagtatgatcccttcgtcacctccatgacgaccaagagtgaggccttctccctcgatgatgtgttcgcgcacctcgttgccttcgaggctcgtcgcctccacaccagacggacatgcaactgcagttcgatgcttccgcgaactacgcgggccgtggcggcttcaatcgtggccgcggtcgtggttatcgtggtcgtggtgaccgcggtcgtggaggccgttcccgtggcggcgcccccgcgcgtggtcctcgccgcggcagcacctctcggcccgagtgccaaatctgcggcatagtcggccacaccgccatcaaatgttggtaccgcatggatgaatcgtatcaggaagaaggaccatcagcggctttggcatcttcacactcgtaccaggtggatcccaattggtacagcgataccggcgccacggaccacatcacgagcgaccttgatcgtcttgccatgcgtgagcaataccacggtggcgacacagtgcaagttggcaatggagcaggtttgcgaattttgcatactggttcttgttcaatttatactgctactcgcccacttgccctcaataatgtccttcatgttcccgacatatcaaaacaacttctttcagttcataaattatctcgtgataataacgtcttttttgaatttcatccatggtattatttcataaaggatcgggcaacacggaagctgcttctggaaggaaagtgtgagtccggtctctatcccctcaagccgtccgacatagaatcccttcatcaagcccttgttggctactcggcaaggccggagcaatggcatgctcggtttggtcatccatctcctcagatcgttcggtctattttgcatcttaataatctcccttgtctcaaggagtctagtgcgtcctcggtttgtaatgcttgtcaattggctaaaagtcatcagctaccttatactaattccattcatcgtacgactatgccacttgaaattattcattctgatgtttggggtcctgctccaatttctattggtggttataagtattatatcagttttattgacgattttaccaaatttacatggatttatcttatgcacgatagaaccgaggctcagcgtatttttctccaattccacaaacatgttgagcgccttcttgacatcaaaattcgatgtgttcagtccgattggggtggggaataccagaaacttcataatcaattttttacttctcttggcatcgctcatcgtgtttcttgtccccacacccaccaacaaaatgggtctgctgaacgaaaacaccgtcacattgttgagacgggtcttgctttattagctcatgccggcatgccactcaaattttgggacgaagcctttttgacagccacatatctcattaatcgtctccctactcgtgtccttgataacttgtctcccatggaacgattatttcaaagtccgccaaattattctatgctaaaaatttttgggtgtgcttgttggccccatcttcgaccttaccaccgtcataaacttgaatttcgttctaaaccttgtgtctttataggctatagctcccttcacaaaggatacaagtgccttgatatggaaaccggacgtgtctatatttctcgggatgtaatttttgatgaggctgtttttccattctctaatccttcatccaactctgctgagcaaccgggtgatagcagttttaatttgaacactaatcacttgcacaatttgcttcctgttaactctatgcctgtagctccgtccaatgcagcagcttcggccaccggtacgacagcaccatctatggagtgcacgccctgcgtgccatcactgacggcgacccagccgctcccgtgtgctgtgcccagcggtcagcaggcaccgtcaggtccacacggagcgccatcactgacggcgaaccagccgctcccgtgtgttgtgcccagcagccagcaagcaccgtcgggtccacatggaggtgccccgactcctgatttactgggctcttcccccacggccgatcagcatgccgcgggaccagccccagctgatttctctccatctgatcccggcactgattctccagaagatactgctgggactattggatctcctgttgatgtggataatcaacaagttcctcatgtccatccgtatggtactcgccttcggaataatattcgacaaccgaagattcgtactgatggcactgtcacatattcagtatcccgggtttgttcttcagaaccatcctcccatgtcactgctatgaagcatcccctatggcgacaggctatgcatgatgaatttgatgcacttctcaagaacaagacttggcaccttgttccaccacgagctggcctcaatgttattgattctaaatgggtttttaaactcaagcataatcccgatggatctattgctcgctacaaagctcgtttagtggccaagggattcaagcagcaatatggtgttgattatgatgctacattcagtcctgttgtcaagccaaccactattcgcctactcctatctttggcggtttctcgtaattggtttattcgacaaattgacatccaaaatgcatttttgcatggtgttcttgatgaagatgtgtacatgaagcagccgccgggtttcgaggattcagcacatccaaagtttttatgcaaacttgataagtctctatacggccttaaacaagcgccccgggcatggttctctcgcttgagcggcaccctgatacaactgggatttcaagcatcgaaggctggtgtctctttatttatctttaaccaagacggtcttcagatttatatacttatttatgtggacgatattattattgttagctcctcatcttcggcgacggacaagcttctccaacaacttcgttgtgagtttgctgtgaaggatcttggtcagctaaattattttcttggcattgaggtgcatcacacttcttcggggctgattttaacgcaacgcaaatatattcgtgacctcctcctgcgcacaaacatggatacttctaaaggggtttctacgcctatgctgcctgctgataagctctcccttcatgatggtgttcctctatctcctgatgatactacgaagtaccgaagtgttgttggtactctacagtatttgtcccttacaaggcctgacatcactttctcagtaaatcgggtttgtcagtttctctccacacctactacatctcattgggctgctgtgaagcggattctacgctatctccacgctactattgatcttggactttgcttcactaagtccacgtcttctctattgagtgccttttctgatgctgattgggctggcaatcccgatgaccgtcgcagcacaggtggattcaccatcttcttcggcggaaatttgatatcctgggcatctcgaaaacattcaacggtttctcgctctagtacggaggccgaatacaaagaagttgcaaatgctacagcagaagtaatttggatccaagtccttcttcatgagcttgggatttctcagcatcggcctcctattttgtggtgtgacaacattggtgccacctatcttacggccaatccaatttttcatcgccgtatgaagcatgttgaagttgattatcactttgttcgtgaacgtgtggcatcgcggcaacttgatgtgcgcatcatttcttctagtgaccaagtggcagatattatgaccaagcctctagcaggaccggctttctctaagatttgtaccaatctgaacttaatttcatatcgtccagattgagggggaa

>Copia-42_ZM_LTR

tgttaaacatagaaggtcatgtatattaggaatgggccatgcgcacttaaatccgttgattgaggctcggttgattgaggcctcctgtaatcttggcatgtcaatcacggcattgctgtcatgtaatacaccctgctgtacgcgcatatatatacatgcctatagccccaagagggtagagtcttttgcaccattatcaaca

>Copia-43_ZM_IN

tggtattcagagccaagttcaccttgcaaatcagccatgtcgagttccccctccaatccactcattgcacatccagtatccgaaaaacttacccgagcaaaccatgccatatgggaagcccatgttcgtgcggccatgcgtggctctcgcctcattggccatcttactggaaccacaccagtgccggagaaggagattgccgacgctgatggcaagaagacgtcaaatccggcgttcgaggagtgggatgctcgggaccaacaggtcctcagctacctgctctcgtctatctccaaggaaatcctggtccacgtctcaagatctgagactgcagcagatgcgtggagaaaaattcaggcgatgttcgcctcacagacccgggctcgggctgtgaatctccgcatcgccctctcgacgaccaagaaaggcagcatgaatgttgctgaatatttcgcgaagatgaagggatatgctgatgatatggcggccgcaggacgtccgctcgaagacgacgaactcgtcgaatacatcatcaccgggttggaccgtgattttacctcacttgtctctgccctcgttgcaagggtggagccaatctcagttgaagaattatattctcagatgctgagttacgaaacaagaatggatctaattcatgaaggagagcagcaggcatccgctaatcttgcaggtcgtggaggtcgctctcgaggaccgggccgcggtcgtgggcgtgctcggcctcctagccgcggctacagcggtgcaggtgtgggcggccaggactcccgtggagcaggtggacagggacatggccctggcggacaagacagccatactgtggcaggcaagcaacgtggctacaggcgctcaagtggtgacccatgccaagtttgctttaagaaaggacatacggctgtcaactgctggcaccgatttgatgaaagctatgtccctgaagaacgccatgatgctgccgcgaccagctcatacacagttgacacaaactggtatgctgatagtggcgccactgatcacatcaccggtgaattagagaaactgtccatccgagataaatacaacggtggagatcagatccacaccgcaagcggtacaggtatggaaatcagccacatcggtaattcctctgttagcacctctagtcgtaagcttcatctaaaagatattctatatgttcctgaagccaagaaaaatcttgtgtctgtccatcgcttaactacagataattctgcctttatagaatttcacccagatttctttttgattaaggatcaagcaactaggcacacgctgcttaggggaccatgtcaccgaggactgtaccctcttccttcgtcatcatcaataaagcgtcacgcctgtggagtcagccagccatcaatctctcggtggcatgatcgtcttggtcatccttcatctgttatagttcggcgaattattaatagaaataaactccattgtttagttgaatctaataatgagtctgtatgcaacgcttgccaacaagcgaaagcccatcagctgccatatcctatttcttctagtaaatcgaactctcctttggagcttattttttctgatgtctggggtcctgcgattgagtctgtgggcagaaaacaatattatgttagctttattgatgattatagcaagtttacctggatttaccttattaagttaaaatctgaggtattccagaaattcattgatttccaacgtcttgttgaaaggcaatttgatagaaaaattcgtgccattcaaacagattggggaggagaatatcaaaaattaaattccttctttaccaaaattggcatctcccatttagtctcttgccctcatgcacaccagcaaaatggggcagccgaacgaaagcatcgccacattgttgaagtcggcctttccttgctatctcatgcatctatgcctcttaaattctgggatgaagctttcctatcagccacatatcttattaatagactacctagtcaagtcattaataatcaaactcctatagaacgtctttttcaccaaaaaccagattattcctctttacgtgtttttggttgtgcctgttggccaaatttgcgcccctacaatactcgcaaacttcaattcagatcaaagcagtgtgcttttcttggctatagtcctcttcacaagggttttaagtgtctcgatatctccacaggacgtgtttatatttctagagatgtcatatttgatgagactgtttttcctttctccgctctccatccaaatgctggcgccagattacagtctgaagttctcttaatgccatccctcacgactaaaataggtgctaatagacgtgatactgatgttcctgattttctccctgttaatactaacttgtctgatgagaatactgcagtgcacagacatcaatatgcagggggtcctgttcaccgaattgatggatcactacccactgttgcaagtttgacgccaggggaggaacctgaccagtctaccgcgcatgcatctgagggaatccgggacactgtacaggcgcacgctgctcctgtgccggaccactgtaccacgcatgctgctcctgtgccgctccactgtaccgcacctacagcccctattccccctattactacaggcgctgatgtgactatacaggacgcactctctttacagcaaacaggggaagcagccgcgttcttgccagagccggtccatgcccatgatgatactgctgacacgtccctacgtccacacacgagactgcggagcggcatccggaaagagaaaatatacactgatggcacaatcaggtatggctacttcacgtcctctggtgagccacaatgtcttgatgaagcacttagtaattctcattggaagaatgctatggatctggaatatgctgctctgatgaataacgaaacatggcatctagttccaccaaaaaaaggcacaaatgtgataggttgtaaatgggtatataaaattaaaaggaaggctgatggaagcttagatagatataaagctcgcctagttgcaaaaggttttaagcaacaatatggaatagattatgaagacacatttagtccagtcattaaagctactacaatacgtattattctatctatggctatctctcgaggttggagccttcgacaacttgatgttcaaaatgccttccttcatggttttcttgaagaagaagtctacatgcaacaaccaccaggttttgaggatccagaaaagaaacaatatgtgtgtaagcttgataaagcattatatggactcaaacaagcaccaagagcatggtactctcgcctaagttccaagctggtgaacctcggtttctcaccatcaaaagctgattcatctttatttttctataacaagggtaacactacagtatttgtgcttgtttatgttgatgatataattgttgcaagctctacataagcagcaaccgcagcactgctaagagatttaaaaggagaatttgcactcaaagatcttggtgatctacattatttcttgggcatagaagtgaacaaggtacatgatgggattattcttactcaagagagatatgcttcagatttattaaaaaaggtaggaatgtcaaactgcaaaccagctagctcaccattgtccactagtgaaaagctatccctgcatgaagggtcattgttggggagcaatgacgccacacaatacagaagtattgttggggctttacaatacttaacactcactaggcctgacattgcattctctgttaataaggtatgtcagttccttcatgcccctacaactgttcactgggcagcagtaaagagaatcctcaggtatatcaaacagtgcacaaagttgggtctgcatatacacaaatcaccctcaactttggtaagcgcattttctgatgcagactgggctggtagcattgatgatagaagatcaaccggaggttttgcagtgtttctaggatctaatcttatttcttggagtgctcgtaagcaacccacagtatctagatcaagtacggagtcagaatataaggctattgctaatgccacagctgaaattatgtgggtacaaattttactcaaagaactaaatattagcagtccgagaacagctaaactctggtgtgataacatgggagcaaaatatttatcagccaatcctatctttcatgcaagaaccaagcacattgaggtggattatcactttgtaagagaaagagttctacaaaagctactagaaattgattttgtttcctcccaaggacaaattgcagacggtttcactaaggcattatcggtcaaactgctggagaactttaaagtcaatctcaacctccgacggctgtgat

>Copia-43_ZM_LTR

tgagggaggctattagaatagtcatattatttaatgtatatcccgaactccttgtaacaacctctagctacggatatagattgtttgttagctgttagctagatttactctcatgtaaccgggtcaagggaaggttgttacatctgatgttatccttgcgcaatgggcgcctatattaacacggaacccgcggcccctgaaggggtatgacgcttcctgccctctgaactttcaca

>Copia-44_ZM_IN

tggtatccgagacccaggcatcgatctaatttcccaaccccctcaccttctcccctccagccgcgccgccttaaccagaccgcgccgcttgtaaagcaggccagccagggaggttctggtgcccaggctgggcgcaccctgggcattctcgggtcttccccgcaggcccacaccgccttcgctccactccaggcgtcctccagcagctccaacacctccaccagcacctgggatgcagcaggcctcatcgctgccctgcaaaacatgcaacttcagggcaactcgccctgggttgttgactccggcgcctctacgcacatgacgtcctcggatggtatgcttactcagcgccttcccccctccatctcctcaataacagtaggtaatggcactagtatacctgttatgtctagaggtcattccgtccttcctacacttacaacaaattttgctcttaataatatccttgtcgccccatccatagtccgcaacctgttatccgttcgtcaattcactcgcgacaacaattgttcctttgaatttgacgcccatggtttctctattaaggatctcaggacggaacgcgtgattctccgttgtaactgcgacggggacctatacaccatgcctgcctccacacctggcgctccacctcatgctctcctggctgcatcgtcaactctctggcatcaacgtctcggtcaccccgcccccgccgttttagagcgtctcaataaacttcatgttatttcctgtaataaagtacaacactccctatgtcactcatgtcaactaggcaagcatacgcatctaccttttagttcttcgcattccataactcatgcaccctttgaactcgttcactgtgatgtatggacttcacccatcaatagtctgtctggtttttcatattacttggtctgtttggatgattacactcactactgttgggtttttccgctccgaaaaaaatctgatgtccaccaacacttagtcgagcttgctgcctctgcccaaacacaattcagcatccccgtcaaatgctttcaggctgataacggcactgaattcgtaaacactgccaccatcaaattcctcgctgctcaaggcactcaccttcgcctctcctgcccctacacatctccccaaaatggcaaggttgaacgtatcatccgcacactcaacaattccattcgcaccatgcttctacatgcttccctaccacccacatattgggccgagggactcctgacagcctgctacctccataataggcgcccatcctcctctattcagcatgaaattccctatactcgtctgcataatcaaccaccaacttatagccatctgcgtgtttttgggtctctctgttaccccaacatgcaagcaacctctaaacataagctcgcccctcgctccacggcatgcatcttcctaggatatcctagctcacacaaaggttatcgctgtctcgacctatcaacccgtcgcatcatcatctctcgccatgtcacattcgacgagaccacctttccctttgcagccacctcagacgtctcgtcctctgcttccctagaattcctcctcgacgacggtatggtttcggtgccctgtcctactgttgttgcaggtgggactccatcctcgacacccgtggttgctccatcccactcggatgttgagcaaccgcccccagacgttgcagcctcacatgactcgccttctgcgacaccgggcgggcgtggtcccgtgcccccgcctggacccgacgtggttcccttcccacgagtctacgtctgtcgttcccgtacgacgtctacgtccgagccggcagctgtcccagcagcccctatgacttcatcgcctccaccgtcgccgcgcgtcactcgcaccatgacgggtgccattcctcgtgcctgctatgaggggttggcggccacgacgtcatcctcaccatcgccgcttccgacaaactaccgcagcgcgctggccgacgccaactggcgtgccgcgatgatggatgaatatcaggccctcgtcgacaacaacacctgggagcttgttccgcggccccccggagccaacgtcgtaacggggaagtggatcttccggcacaagttccatgccgatgggtcccttgctcgccacaaggctcgctgggttgtccgtggcttctcccagcgcgagggcgttaactacgacgagaccttcagcccggttgtcaaaccggctaccatacggtctgtactcagcatcgctgcatcccgcgcctggcccattcatcaactggacgtgaagaacgcctttcttcacggccacctcgaggagaccgtctactgccaacagccgccaggattcgtcgaccctggcgctccagatcacgtctgccatctgcagaagtccttgtatggcctgaagcaggctccgcgggcatggtaccagcgcttcgccaccttcgtccggcacctcggcttcgtcgcctccatctccgacacgtctcttttcgttctacgagaggggacaagtctcgcctacctactgttgtacgtcgacgacatcatcctcaccgcctcgtcgtcggccctgctacaacgcattatgactcggttgagctccgagttttccatgacggatcttggcgccctccaccacttcctcggcatcgctgtcacgcgctcctctgatggtctcttcctctcccagcgacagtacgcggtcgagcttcttcagcgcgcaggcatggctgagtgtcatccgacatcgacacctgttgacactcacgccaagctatctgcgaccgacggggatctactgtcagagaaggatgcatcagagtacaggagcttagcgggagctctccagtacctgacgttgactcgtcctgatctggcatatgctgttcagcaggtgtgcctcttcatgcacgccctgcgcgagcctcaccgtgcgctggtcaagcgcatcttacgcttcgtccaggggactctctcctcgggtcttcacattggcaccgggtctgtcaccaagctgactgcctactctgacgccgactgggcaggctgcccagactctcgacgatccacctcgggcttctgtgtctatctcggcgacaatctagtttcatggtcctccaagcgccagaccacggtgtcccgctctagcgccgaggccgagtatcgggctgtggctcacgttgtggctgagtgctgctggcttcgacagttactccaggagctacacatccagctgccttctgccacagttgtcttctgcgacaatgtcagcgccgtctacatgacggccaaccccgtccaccacaagcgaacgaagcacatcgagatcgacattcacttcgtccgcgaaaaggtggcccttggtgaaattcgggttctccatgtgccctcctctcaccagttcgccgacatcatgactaaagggcttccgacagcactctttcgagaatttcgatccagtctttgcgtccgggagcctcccgctgcgactgcgggcgggtgttaggcaatagaatatgccatctgtggatatcctagc

>Copia-44_ZM_LTR

tgctatagatagcagatgtaaatcagggatagaatcctagctgctatagatagcagatgtaaatcagggattagttgctagatatagcagatgtaaatcagggataaatcaggggaattatccctgccttgtaaagcaggccagccagctctatataatagaaggtgccccctctcattgaggtgtggccaattgccctcaaccctgtttctctaca

>Copia-45_ZM_IN

tatcctaatacccgcccgcagtctgagcgggaggatcacggacgcagagactgttccgaaattcagtgaagagatgtacgggcagtcccttggtcatgatgtcggcgaactggtgtgtggacggaacatgaagaacacggaactgccccaaagctaccttctcacggacgaagtggatgtcgatttcgatgtgcttcgtgcggcggtgatggactgggttggctgccatgtacacagcgctcacattgtcacagtagaccacggtcgcactggggatgaagatgtgcagctcatgtagaagctgacgcagccagcagcactcagcaaccgcatgggccacagcacggtactctgcttcagcgctggagcgagagacagtcgtctgacgtttggacgaccatgacaccagattgtcgccgaggtagacgcagaagccggaggtggagcgacgcgtgtccgggcagccggcccagtcggcatcggagtaggctgtgagctgatcaacggcgccaggacccaagtgcagacctgcggacagggagcccttgacatagcgcaggatgcgcttgatgagcgcaagatgcggctcgcgagggtcgtgcatgaagaggcagacttgctggacagcataggccaggtccggcctggtcagagtgaggtactgaagagcaccagcgaggctccggtactccgagggtttggcgactggagcgccctcggaggcagacaacttggtccgggcatcccaccggagtagctgtcgggtggcattcagacatgccagcgcgctggagaagatcaacggcgtactgccgctgggagaggaacagcccactgctgtcccgtgtcaccgagatgccgaggaagtggtgaagatccccgaggtccgtcatggcaaattccgacgataggcgggaggtgacgtgctggagaagagccggcgatgatgcagtgatgatgatgtcgtcgacgtagaggaggaggtaggccatggcgtctccattcttgtagatgaacaaggaggcatccgttttggaggtggtgaagccaatactgcgaatgaagctggagaagcgctgattccaagcgcgtggtgcctgcttaagcccatacagggacttctgcaacagacagacagagttaggtgcagcggggtcgacgaatcctttcggctgctcgcagtagacagtctcctcgaggttgccgtggaggaaggcattcttcacgtcgagctgccggatgggccagtctcgagacgcagtaatgctgaggacggtgcggatcgtggccggcttgacgaccgggctgaatgtttcgtcgtagtcgacgccttcctcctgggagaagccacgaacgacccagcgcgccttgtgccgggcgagggagccgtcggagtggagcttgtgtttgaacagccacttgccagtgacaatgttcgcaccaggcggccggggtacgaggcgccaagtgccgttgtcgatcaatgccttgtactcgtccgcccatcgcagcacgccaatttggatcggccagggcactgcgatagttcgccgggattggcgaggcgacggaggcggagaagccgaagcgctggacagtgcgaagggagccggtctgtgagcgtgtcacaggccggttcgctgcagtagagaggagccgagctgcaggagccgctggtggcgaagttggtgcagctgctgttggcgctgaaggtgcagcagccacaggggccaccgctgcggtgaccggtgctgctggaggctgggcgcggcgggtgtaggtgttgccgaagcgctgaccatcagtcgggggagccgccggctgccctgcttggggcccatgagcgcccagttgtggcccgcccgcagggtgaacccgggggccgagctggagtatggccgggtccaggaacacctggagatcatcctcgctgctggcagcgagcgacggatgggtgtttgcaggagccgtcgaggaggtgaggtcctgcatcagaaaatcaagtgacgacggcatggaggccgccgatggtgcagccgagaatgggaacacagactcatcaaaaataacatggcgcgagataatgatgcggcgagtggacatgtcaaggcagcgatagcccttgtgagaggatgggtatccgaggaagacacaaggagtggatcgaggggcgagtttgtgaggggaggtggcagtgaggttcggatagcagaggcatccgaagatgcgaagggaagcgtaatcagggagtaggccgtgaagaatctggtaaggtatttggttttgtatggaggaggaggggcgcctattgaggaggtatgttgctgttgtgagggcttcagcccagtatggaggtgccatggacgcatggagcagcatggtgcggatggtgttgttggtagtgcggagcatgcgttcggccttgccgttttgtggtgaggtataggggcaggaaaggcggagagtgatgccgtggctagcaaggaaggaagtggtggcgcggttgacaaactcggtcccattatcggcttggaaggatttaggtgtggtactaaactgggtctgggcgtatgcaatgaattcaatcatgtggcggtgcacctcagatttgtgacgtagtgggaaggaccagcagtaatgggaataatcatcgagtataataaggtaatacttgaacccagaattgctaagtattaggcatatatctacagccggctgtcctaccctgtatagatgacagccttcctagtttagccttaccttgtataggtgacagccttcctagtttaggagattgtatccttgtacaccaagccacttggctatatatataagaagtcacgcacctgattgtgtgtgcggtgctttcccctaaaccaattctctacatggtatcagacaggatcaattcgatcccggccttccttccgctgctctctgctttctctcacgccgccggcacccctccacctctcctccatggctgagcagaccttagacgccaccgccaaccttggcgccatccagcagctcatggtgccctcgtatgccacggtttcggttcaatctcatgtaccgaccaagctggagatgaaggcctccaactactcccgatgggcatccttcttcaaatcgatgtgcggcaagttcctccttcgccaccacatcgacggctctgctcccccacgcccgcaagacccgatctgggatcaagcggactgctgcgttcgcagctggatcttcggctccgtcgacgactccatcctcagcctcgccgtcgacggtgacaaccagaccgcccgcaacctttgggtcgccatcgaaggtctgttccgcgccaacaaggccccgcgggcgattctccacctccacgacttccactccatgacccaaggcgactcatccatcgacgagtacgctgagatcatgaagcggaaggccgccgatctccgggacgtcggccatccagtcgaggactcgcagctcgtcctcaatcttctccgcggcgtcaatccccgcttcagcaacaccgcagacgacatcgccaactccaccgtccttcccgacttcgcctctgctcttgatctcctgaaactcaaggagctccgcctcgccaacgaggtcaagacggcggccgccacagctctccttgccgccacctcctcctgctctggcccgggcggctgccgctcctctgctggtcctcagcagacaccggcaggcggcggcggctatggcaaaggcggcggtggatacggcggcggcagcaagaagaagaaaggcggcaactacggcaaacatgctggcgcatctactcaccagtggccgcagtcgggccgccagccgtcgggcccctggttctgcttctccccctatggggcgccgcagggaggcccaggcggacagggcgggtggcgtgctcctggcgcaggagtcctgggtgtcgctccccaacctcaagcgcacaccgccttcgcaccgctccagatgtcgtcccccacgcctaactgggaccaatccggcctcatcgctgccttgcaccagctcgcccttcagggacagggcagctgggtgatggactccggcgcctccagccacatgacgtctacggatggtatactactctcccgtctcccaccctcttacacttccatcactgtcggcaatggtcacaacctacccatcacctgccgcggctcctctgtccttcctactagttcatccactttcactctgcgtgatgtcctagtggttccttccctcgttcgtaacctcctcttggtacgtcaatttactcgtgacaacaattgttcgattgaatttgacgctcttggtttttctgttaaggatatggcgagtcgtcgcgtgatgcttcgctgcgatagtgatggggacctctacaccattccagctgccacccacacctcgccgtacgcctccattgccatcaccacctccctgtggcactctcggctcggtcatccgtcttctactgcaataaatactcttagaaacacttccgccatttcttgtaataaagtagaaccacctctatgtcattcctgtcagcttggcaaacatgtgagactaccttttactccttctcaaacacgcagttctgcaccctttgatttgattcattgcgatgtttggacgtctcccgtggctagcatatcagggtaccagtactatttggttttgttagacgattactctcatttttgctggacttttccacttattcgcaaatctgaagtagcctctcacatcactgatttttgcgctcttgtgcaaacccaatttggcgtcacaataaaatccttccaggctgacaatggcactgaatttgctaaccacactctcacctctctattttcatctcgtggcattcttttccgattttcctgcccttacacatcccagcaaaacggcaaggcagagcggatacttcgcacgctgaataacatcagccgcactctcctcatacatgcccacatgccgcctccttactgggccgaggcgctcgtcaccgccacctacctcctcaaccgccggccctgttcctctgtgaacaacgccgttccctacaccatactatataacaagacccccgaatacagtcaccttcgtgttttcgggtgtctctgctatcccaacctctctgccacaacaccacataagctagctcctcgctcggcagcctgtgtcttcttgggatatccttcttcccataaaggctaccgctgtctcaacctcaccaccaggcgcgtcatcatctcccgtcacgtcgtgtttgacgagacatcctttcccttctccaccacactcctccctgtgacggatatggattttttgattgcccctaaccctgcagcagctgttcccgtggtagctccgtcaccacgtgacgtcgagcgaccacggccgtcgcctgtcgtgcatgaggaagaccctgctatcctcctgcgcggccctgtcctccaggagcttgcctctaggacacctccttcgtcagctgcgccaccgcctccgccgactacacttccggatgcaccacgcgaacgtccgcgtgtgtacgctcgacgcccgcggactacaccagctctgcccgagtacgtggctgagccggccatccacgtcgtcgacgaccccgtctcatcttcaccggctccacctccacctccgccgcctctggagccccgtcgggtcacccgcacccagtctggcgccatccaaccggtgcggtacgtgggactctcggcgactgcgacaacgccggcctctccgattcccggcaactaccgcagcggtttggctgatccaaactggcgtgcggccatggccgaggaataccaggcgctcctcgacaacgacacctggcgcctcgttccccgacccccccggtgccaacgttgtctccgggaaatggatcttcaagcataagtaccactccgatggcactcttgctcgtcacaaggcacgctgggtggtgcgtggtttctctcagcagcacggcatcgactacgacgagacgttcagcccggtggtcaaacacccgaccatccggactgtcctcagcatcgccgcctctcgttcgtggcccatccgccagctggacgtgaagaacgcttttcttcacggccacctcgaggagacggtctactgccagcagccgcccggcttcgtcgaccccgctgctcctgaccatgtttgtcttttgcagcgctctctttatggcctaaagcaggctcccagggcgtggtaccagcggttcgccacgtacatacgccagctcgggttcaccgcctcctcctccgacgtctccctctttgtctacaaagagggtgagagtctcgcctacctgctcctctacgtcgacgacatcatcctcaccgcctcttcgacggatcttcttcagcgcttcatcacccttcttcactcggagttcgccatgacagaccttggggacctccatcacttcctcaacatctccgtcacgcgctcctccgacgggatcttcctgtctcagcgacagtacgccgttgagcttctccagcgcgctggcatggctgagtgtcactctacatcgacccctgtcgacactcatgccaagctatccgccactgagggtgctcccgttgctgatccatccatatacaggagcatcgccggcgccctccagtacatcaccctgacccgacctgacttggcctacgccgttcagcaggtgtgtctcttcatgcacgacccgcgcgagccccacctggcgctgctaaagcggatcctgcgctatttgaagggcactctctcttccggccttcaccttggtgtcggccctgtacagtccctcaccgcgtattccgatgcggattgggctggctgccccgactctcgacgttccacttcaggctactgcgtctacctcggcgacaacctcatctcttggtcctccaagcgacagaccacagtttctcggtccagcgcggaggctgaataccgagctgtggcccatgctgttgctgagtgctgttggctgcggcagctccttcaggaccttcaggtctctcttgcctctgcgacggtcgtcttctgcgacaacgtgagcgctgtctacatggcagctaatcccgttcatcatcgccgcacgaagcacattgagatcgacatccattttgtccgcgaaaaggtggcactggggcaagttcgggtgctccatgtcccctctactcatcaattcgcggacatcatgaccaaaggcttacctgtccagttgtttactgagtttaggtggtctttgcgtccgtgactctcccgctgcgactgcgggcgggtattaggcatatatctacagccggctgtcctaccctgtatagatgacagccttcctagtttagccttaccttgtataggtgacagccttcctagtttaggagattgtatccttgtacaccaagccacttggctatatatataagaagtcacgcacctgattgtgtgtgcggtgctttcccctaaaccaattctctacactaagcacgggagatgtccatacatcgcagtgaagtaaatcaagcggagcatgagtaacagatgtcgagtgactaaatggaaggcgcacatgcttgccaagttgacaggcatggcagaggcatggcgagcttttattgaaggaaatgaccgacatgcttcgaagtttggcgaggctggagggaccggggtggccgaggcgatgatgccacaaagacgacgtggtggcgaggctgcaggagggagttgtggtggaggggtaggtgtagaggtcaccaccactattgcagcgaagagtcacttgccccgtctgtttgtccttgacagaaaaaccataagcgtcaaattcaattgaacaagagttgtcacgagtgaattgacgaacagataaaagattgcgcacaagggcaggtgcaacaagaacattattaagacgataggtagaggtgggggaggatagtgtagatgtgccacgacaggtgatgggtatggtggtgccatttccaacggtgatgccgaatgaaggaggggggagatgggacaggagtataccagccgaagaggacatgtgagctgtggcgccagtgtcgaggacccaaggggaggacccctggagggacatctgattcagtgcggcgatgaggccagcctgatcccaaccgccctgcgctgccataggtggagcagggttgtcgggggactgaacaggggcgagcgcagtgtgcgcctgagcctgagtgtaggggccgaggatgccaggactgcgccaggcaggggttggcgccggcgggcgccagggctgctgatgaaccccggcggtccaaggggggtagcagtaccagggggcggctgggaacctgggaccgccagagtgagggccgcccttgcccttcttcttccagcggctgccgccaccacctgggttgccgccgctgctggagccactgccgtgaccgccctgtctactggcagtggagccgccgccgttggtggtgggggtggtgcggcagttggtaccgcacgcggcgtggagtgtcgtctgggccgcaaccgtgccttcattggcgaggcggagttccttcagcacaagcttctcgcgagtggtggcgaagtccggcagcgggtgggagtcggcaatattgtcggcggtgctggcgaaacgtgggttgaggccgcggaggaggttgaggacgagctccgcgtcggtgatggtgcggccgacatcacggagcgcgtcagcggtggccttcatacgctgggcatagtcattgatggaggagtcgccctgagtcatcgagtggaactcgtggcttaggaagatggcgcgtggagccttgttggcctcgatgaggcgcttgatggcgacccagagctcacgcgcagtctggtttggagccgcggcgaggccgatgacgtcgggcccagccgagccgagaagccagctgcggacgcaggcatcggcgatgccccaagaaggatctgtgggacgggcttcctgcagtccgtcgatatgcgagaggagagcgaattttccacagagggaggtgaagaatggttcccactggttgaagttgggttttgccagttcaagagtcaccgggatgtggttcttgacattgatcgtcgcatagggggcgacaatcacagtgggagcaggagtggtggcgtcggtcatggtgaccagggggggaggagttgcggcgcagcagggaggagttggcgctgcggcgctgcgcacagcagggagatgggcgcaggatctagagagctgatacca

>Copia-45_ZM_LTR

tgtagaaaggggaagaatggattgaatcaccacacacccattgagggtggggagcctgttatatatagccaataggcatgcatggaaggtacatgtagggaaggctattccctgtacaaggaaacagacctaggctatagatagaaggtaatcctagtctatacaaggtagctagccaatctaggggattacaatcaattgctaatcaggctctgattgatcctgatagataca

>Copia-46_ZM_IN

tggtaccagagcctagggttaatcatcctagcaatggcgtcctcaaactcgtcatcgaatcctctccttggtgttcaagtcacagagaaactcactcgccagaatcacggtatgtggtcggcgcaagttcttgcgatcttacgaggcgcaagactggagcggtatgtcaacggcaaagccgtcgctccatctgaagaaattgatgagaagcagagcgacggcaagatcgctaaggtttccaatccagcctacgacgaatggttcgccacggaccaacaagtcctaggatttcttctcacctccctgtccagggatatcttggcacaagtcgccattgcaagaaccgccgctgaagcatggaagatcatcaccgacatgttcgcctctcacacgcgtgcacgtgctactaatgtgcgtcttgccctcgccacgacgaagaaggacaacatgactgttgcccaatactatggcaagatgaaggggcttgcagacgagatggctgcagcaggaagaccccttgacgatgaggaactggtgatgtatatctgcaacggtcttgatcttgagtacaatcctctagtttctgctcttgtaactagactagaacctgttactccttcagaattgtactcccaacttttgagctttgagactagattagagttacagcttggcatcgggtcatcgtcatcagcgaattcagcaggaagaggcggcggccgtggtgggagccagcaaatgcgtggcccaaatcgtggccgaggtggacggcagaactccggccgtagctctggtggccgtggccgtgggcagcagcagccactacggcaaaaccagcaacgcaggagcttccccacgggcccaactgcaacccacgacaacaatgggcgcccaatctgtcaggtctgcttcaagacgggacacacggcgattgactgttggcacaggtttgatgaaaactatgttccagaggaacgccatgttgctgctgcaatgagcccttattctatggatcaaaactggtacatggactctggtgctacagacaatatcactagtgatcttgagaagctcgccgttcgtgacaagtatcatggaagtgaccagattcatacagcaagcggcgcaggtatggaaatcaaacatattggtcattcattagttcacaccccaactcgcaatttacatctaaacaatattcttcatgttccaaaggccactaaaaatcttgtttcagttcatcgcctcaccaaagataattctgctttccttgaatttcatcctgattattttttgatcaaggatcaggcaacgaagaacaccatccttaaggggccatgccgcaagggtctttaccctcttcctctacccgccataaagcaagcctttggagttgtcaaaccatctttcgaaagatggcacagtcgtttgggtcatgcttctgcccccattgtttctaaagttattagcacaaataatcttccttgtttagatgaatcaaataaagagtcggtgtgtgatgcctgtcaaaaggcaaagagtcatcagcttccctatcccaagtcacatagtgtttctagtaaacctttggagcttgtgttttcagatgtttggggtcctccacctaattcggttggaaagaacaaatattatgttagctttatcgatgattacagcaaatatgtgtggatttatcttatcaagcataaatctgaagtttttcagaaattcaatgaatttcaaaaccttgtcgagagactatttaatcaaaaaattattgccatacaaactgattggggaggtgaatatcagaagcttcactccttttttgagcgtgtcgggatatctcaccttgtatcatgtcctcacgctcatcaacaaaacggggctgcagaacgaaaacataggcatattattgaggtcggtttagctctccttgctcacgcctcaatccctcttaaatattgggaagatgcttttgtagctgccacttatttgattaatcgaactcctagtcgagtcataaattatgacacgccccttgaacgtctgtttcagcaaaaaccagactattcttctcttagagtgtttggttgtgcatgttggccacatctacgtccctacaacacacataaatttcagtttcgctctaagcagtgtgtgtttcttggctatagtccctcgcacaaaggctttaagtgcttagatccttccacaggccgggtctacatatctcgggacgtcgcctttgatgagaatgtctttcctttctcacaacttcatccaaatgcaggagctcgacttcgtcaggaaatttctctccttccatctgatctccagccaataccatctgatcaagggggagaactaatgggtgatcacaggtttgattctaataatctatgtgatgaagtgtgtgcagaaaccgcgtcgccagcacgtgtgtccgctctcccgtccagcatcgctgatcaaccaaatcaagctacgtcgccgaccgagcttgggcagcagacagaatctcatgcgcctgattcctatgacccgatcggcgccgattccacagaggatttgccaccgccagcaccgggatcgcctatggccgagggagctgcgtcccaaattgtacagcctactgcacctgaagattcaaggccaaggacacgtctacaagctggaattcgcaaaccaaaggtatatactgatggcactattagatatgggtgttttacatcatcaggggaaccacataatataaatgaagctttagaagataagaattggaaagatgctatggatctagaatactctgctttgatgaaaaataagacctggcatcttgtacctccaaaaaagggcactaatattattgactgtaaatgggtgtataagataaagagaaaggcagatggaagtcttgacaggtacaaggctcgtcttgttgctaaaggcttcaagcaaaggtatgggatagactatgaagatactttcagtcctgtgattaaggcagcgacaattagagttattctttctgttgccgtgtcaagaggttggagtcttcgtcaactagatgttcaaaatgcctttttacatggtttactagaagaggaagtttatatgaagcaaccacctgggtatgaggacaaatctttgccaagttatgtgtgcaggctcgacaaggctttatatggactcaaacaagcgccaagagcgtggtattccagacttagtatgaagttacaagacttgggtttcaaatcatctaaggcagacacatctctgttcttttataataaaggtaacatcagtatgtatgtacttgtttatgttgatgacatcattgtggctagttcaacaccaagtgcaacttcagctttgctcagtgatcttaacaaagagtttgctctaaaagatctaggtgatctgcattattttcttggaattgaggtaaataaggtatccgatggtctgattttgacgcaagaaaagtatgcctcagatgtattaaaaagaattggaatgagtgattgtaaaccagtcgctactcctatgtctacttcagagaagctttcggtccatgaaggagatctactcggtccaaatgatgctactcagtatagaagcatggttggtgcattgcagtacttgactttaacaaggcctgatatctcttttgctgttaataaagtatgtcagtttttgcacgcacctactactgttcattgggctgcagtcaaaagaatcttgagatatctaaagcacacaacaaggcttggcttaaagattggtaaaacaacgtcacttctagttagtggtttcagtgatgcagattgggctggaagtcttgatgataggcgctccacaggagggtttgcaatttttctaggatcaaatcttgtttcctggaaagcaagaaaacaagcaacagtgtcaaggtctagcacagaagcagagtacaaggcgttggcaaatgcaactgcagagatcatgtggattcagattttactgttagaaattgggattcaagcaccaagaaaggcaaagctatggtgcgacaatataggggctaaatatctttcggcaaatccggtatttcacgcacgtacaaaacatattgaagttgattatcattttgttcgagaaagagttgcaaggcaattacttgaaatagaacatgtgtcaacaagagatcaggtggctgatgggtttacaaaaccactggcggcgcgacagttggaattgttcaaaggcaatctcaacttggagaagatttagattgagggggag

>Copia-46_ZM_LTR

tgttaacatatatgtgttgtattccaaatatggttgagatattgccggctaagatctataggcagttaatagaaatagatcaattgtaaccatgtaaaacttatcctttcctagaatagatctaaagctggccatactcatctatatatatgtaactcgcgccttgtttttgtgccaccagaaatatcaaacacgaagccgtcgtgatctctggagcggagaccgacgttccacctatttccatcgcgcctctgatttctaca

>Copia-47_ZM_IN

tggtatcagcctagcctaggtttttctccagccgccgccgccgggatctgccctgcgcgccaggcggtcgccgcccgaagtccctgcgcgccaggccgcgcgcccccagcgccgccgccgggacctgcctctgcgtgcctggtcgccgccgccaggagccctgcgcgccaggccgcgccgccgtccagcccctgcgcgccaggccgcgcccggagccagccgaccgagcgccgcccagggatctgcgccgccgtctgcgtccggccgtccggctgtgcttccgcgccaggcggccgccgccaggagtccctgcgcgccaggccgcgcctccagcctgccgtccgcgcgccgcccctgcacgccagcgaccctcccatggctggcgagcagcctcctctgtccccctccactttccctcccctctcctcctgggcgcgggctgtctcccctggcaggcccgccgtcgccgcctctcctgccgctgctgcagccaccgtgcgtgggcgtgcccccctggcccctccccaccccctcccagccgccgccgctgtctcccctgcaggtgcgcaggcgctggtggcaggggcgtgggggtccgtgcccgcctcccctgcgctcgggccgacgtggggggacgggcccaccgccgccggtggggccctcaccgctgcgcgcaccgcctcggcccttgcggctgtggcggatgcagttcccgcgagggttgccggtccggaggaccctcccccgccaattcccccgtcgttggtcgttgatccgcctccggtcgtcgatcagccacctcctccaccgatcacccaccagggatacaccgtcgccgctctcactgctgcccgtgcggagcacgcggcacgacaggcccgcttgcgggaagcggccctcgtatgggagcgtgagcgtgaggccgccgacgctatcgctgctcagattgccgcggcggaacaactcctcgcctcgcctgcgacccacgacggcggggtcacctcctctgacaccatgggcggcgtgtacggggtcccgaccgcaccgaccgtcggaaccgggccccgcaccatccccacggtgctctggcatgacccggctgacccgctagtggctcagctccaccttcaggctgggagtgtccagaacattcgcctgatggttcctgtcgtcctggagcccgagtcgccgtcctacgcacgctggcgggacttactcctcctcacccttcgtcgctacgctctggacgaccacgtcctctgcgaccccaccggcatggcgccgactgccacgtgggtgcgccttgacagcatcgtactcacctggatcgtggggacgatctccgttgacctccacagcctcctccggaaccttcctcacgctcgggctgtttggcttgccatcgagggccagttcatgggcaacgccgaggcccgggctctccgcctcgacgctgcttttcgcaccttcgtccagggggacctcagtgtcagtgcgtactgccgcaagatgaagaccatggcggactctcttggcgatctgggttgccccgtggaggaccgcatcctagtcctcaacgtcctccgcggcctcggcgatcgctacacccacctccggtcgttgatcatgcgccagcgcccctttcctaccttcctccaggttcgcgacgacctcgccctggaggagatcactctgggcgctcaggctgcatcgatctccggcacggggtcctcgtcctcttcgacagcactggcggctttcaccccgacccgtcctcctactccgtcacagtctgcccttggccctcgccctcccgggccgagcatgggcggcgggggtcgaggtggcagaggcgggggcggtggtggtcgtcgccgccgtggtggccgcggtggtggttccgggggtggtgcccgtggccacgccccgacgccgggaccccagcagggtgcgccttggcccacttttcaccacccgtggtcagggcgcatctccatgtggccgttccaggggccaggctctgaggctcggcccccggcggccatgttcgctggtgcgcagccagggttcgccttcgcctccccgtcaccatggacctcgacacctgccgcttcgtcgtggcccacgccaccggctactcctccgtccgggctggttggttgggacgcggccaccctggctgcctttcagactcctactctgactccgccgatgggtcccgagtggatcgcggacaccggtgctacctaccacaccacccccgaccctggtatactcacctctgttcgccctccttcttccggctcacctctgttcgctctcccttcgtccatcatggtggcgaatggctcatgtcttcctgtcacatctgtgggtgccgccagccctcccggctcttttcgcattcccgatgttcttgtcgctccttctttggtccacaatcttctttctattcgtcggtttactgctgataattcttgttctgtcgagtttgactcttttggtcttactgtgaaggactcggcaactcggcgccccctcctcagatgtgacagcaccggccccctctacacccttcggcttccgcacgccacatcttcttcgtcttcgtcacctgacacagctgctgtttttgctgccgccacgtcttctaccacctggcatcgtcgccttggtcaccccggacgcgatgccttgatgcagcttactcgtagtgccactatcccatgtactagatcccatgatgagcatctttgtcatgcgtgccagttaggccgccatgttcgtcttcctttttcttcttcttcctctcatgctactcatgcttttgatcttgtacactgcgatttgtggacttcacccattaccagtatgtcaggctacaaatactatcttgtggtgcttgatgatttttctcattatgtgtggacttttcctttgcgtgccaagtctgagactttccccgccctccgccacttcttcgcctgggtgtccactcagttcggcctcaccattaaggccgttcagtgtgacaatggtcgggagttcgataactccacctcccgcgacttctttctctcccacgagatgcagttgcggatgtcttgcccgtatacctcctcccagaacggcaaggctgagcgcatgatccgcacgaccaacgacaccatccgcactcttcttctccaggcgcaccttccggcatgtttctgggccgaggccctccacacctctacctacctcctcaaccgtctcccttccactgcgtgtccggcccccactcctcaccaggcactcttcggtacccctccgcgctatgaccacctccgtgtcttcgggtgtgcttgctacccgaacaccactgccactgctcctcacaaactagcaccccgttccaccctctgtgtgtttctcgggtactccccggaccacaaaggctaccgctgctttgacctctgctctcgtcgggtcctcatctctcgccatgtggtgtttgatgagtccgtatttccctactcctccaccaccacaccaccctcctccgaccctgaccttgaccctttcactctctttccgactgacacggtggtcgagccacctctccttcctctttctgcaggtactcgctcaccacctgtcggtcctacccccggcccggtaccttgctcgggtccggtggtgtcgcctacggtgctgtcgcctctccgcggagccccgtctccgatcgcccccgggccgagcgagggtggtgggactgcaccgcctacggggccatcggttctgacccctccggcccgcttcgcccagccggtgcgggtctaccagcgccggctgccgccgccggggttcgcacctccaccgtcgccgcccctgccgctgtcaacgccggtggcccagtccccgccagggacacccacacctccgcctcggccacccgcggcccgtgtcgagacgccggtgtaccacccaccgctccttcaccgcgacccgcggcacgtccacccgatggtgacacggcacgcggctggtaccctgccgccccgtgtcctggcggccacgaccgcagactcgcagatttctccggtaccctcccccgttcgcaccgccctgctggacccccactggcgtcgagcgatggaggagtacgcggcgctcgtcgccaaccagacgtgggatctggtgcctcgtccgcccggcaccaacgtcgtcaccggcaagtggatctggacgcacaagcggcgggccgatggtaccctggagcggtacaaggctcgctgggttcttcggggcttcactcagcgccccggagtcgattacgatgagaccttcagcccggttgtgaagccggccaccgtccgcacggtgctatcgctggcccttgctcgcttctggccagttcaccagctcgacgtcaagaacgccttcctgcacggcgctctcaccgagaccgtctactgctgtcagccagcggggttcgtcgactcctctcgccctaccatggtctgccgcctcaacaggtccctctacggcctcaagcaggccccccgggcgtggcactccagactggctacgtttccggtgacactgggcttcgtggaggccaagtcagacacgtctctgttcatccaccatcatggagcagagactgcctacttgctcctctacgtggatgacattgtcctcaccgcctccagtcagtcacttcttcgccgcctcgtcgacgcacttcagcgggagtttccggtcaaggatctgggtgtgcttcaccatttcctgggggtcactgctgagcctcgcccctcaggactcctacttcaccagcggcagtacactcttgacattctggagcgggccaagatgattgactgcaagtcctgctctaccccagtcgacacgcaggcgaagctctctgaggccatgggtgacccagtcgaggaccccaccgggtacaggagtcttgccggtgcccttcagtacctcatcttcactcggccggacatctcctacgctgtgcaacaggtctgtctccatatgcacgacccccgagagccccacctcgcggctctcaagcgcctcctccgctacctccggggcactgtcgactacgggctgcttcttcaccggtctacctcctccgagctggtcgtctacaccgacgctgactgggctgggtgcccggacactcggcgctccacctccggctacgccgtcttcttaggcggtaacctggtgtcctggtcgtcgaagcgccagccggtcgtctcccgctcgagcgctgaggcggagtaccgtgccgtggccaacggcgtggcggaggctgcgtggctccggcagctcctcgccgagctccacagtcccctctccaggagcacgttggtctactgcgacaacgtcagcgccgtctatctctccaccaacccggtgcagcaccagcggaccaagcatgtggagatcgatctacacttcgtccgagatctggtcgccgtcggtgatgttcgggtcctccacgtcccgaccacctcccagtttgccgacatcttcaccaagggtctcccgtcctcgaccttcgccgagtttcgctccagcctcaacatcagtagtggctagttgagtctgtggggggatcctagtgtgtgatgtacttcttctttttggatccagtctgtaaactccgctgcgacggatgtttagactgcggggggg

>Copia-47_ZM_LTR

tgttagattatatgtggttaggcccatgtggctaggcccatatggcccatgtacaaacactatatagctaccctctagggttagccctaaggatacacaattatttacctctaaca

>Copia-48_ZM_IN

tggtatcagacgcctaggtcctgattcctgccctccatccccgcgcgcctcctctacggcgctgcttctccccatggccgacctccagccaacctctccggccatcaccgatcttgacggcgacgcctctgctgattctgagcgcgcggccctggctgcccgtgaggctgctttggctcgcgccgccgctgcagagcaggacgccgccctcgcccagcgcgagcgcgacgccgccgacgcccgcatccgtgcggccctcgcccgtgcagctcacgagcgcgcggcggcggccctctctccccccatcaccgacctcacctccgacggcactggtgctgactccgtcgcccagctttctgccgctcttctccaacacgaagcggctgcacttctcaatctgcacgctcaagccgtcgccgtgcagaacatccgggccctcgttccactcctcctcgacgtcaactccaccttctactctcggtggcgtgagtccttcctcctaaccctcaccaaattctctctggattgccacgtgctgtctgatgctgtccaccactcgcccgactggactcgcatgaacgccgttgttctgtcttggatcaacggctccatcaccgacaacctcgccgatatgatctccgagcgcggtgcatctgctcgcgtcctgtggctcgctattgagtcccaatttcttgggaaccgcacgacgcgcactctatacgccgaccaagctttccgctccttcactcagggcgacctctccgcagctgattactgccgccgctacaaaaagcttgccgaagatcttcgcgatcttggcgagccggtctccgacaagacccttgtcctcaacatcattcgcggcctcaacgaacgctttcaagcgctcggccttcacctccgtcgcacctcccctctccccacttttttgcaggtccgcgatgatcttactctggaggagctcaccatgactaatgctgctcctgcagcagctctcgctgctgttactacaccacctggcgagaacagcagctccaagtcatctacacccaccgctcctgctgggcgttcttcacatcagcgcccgccctcccaccattccgggggcggttccggtcatggctcctcctccggacaacgcggcaagcgaggcaagcgcggtggtagaacctccagcaacagcagcagcggcatcattggccctgcaccctcctccaactacaactactacaacccttggacgggggccatctacatgtggcctggccaacgtccaccttcgactcctcgtccgcctgctgcctcgcccccaccaccacatgccttcgtcgccggcactcctggtcagtgggctgtccctgctgcagcatatgggccgccccctgcctacgccgatccttctacgcctgcacccgtctgggatcaacacaccctcgctgccaacttccaaactgtcactcttcagcagccaccccaacaagagtggcactttgacactggtgcctccagtcacatgacatctgacgctggtataatatctcatcccccctcttcccagcatcttcctactcatattgtcgttggcaacggccatcttatccctgttacctccactggcaccactcgtcttccctacaacctttctcttcataatgtcctagtttccccctcgctcattaaggatctgatttctgtccgtcaatttaccactgataataattgctctgttgaatttgatccttttggctgctctgtgaaggatcttcccactcggcgcgagatcttcagatgtaacagctctggtccactataccccctgcggttcccggcctctgctcttcttgcatctaccctatggcaccaacgacttggacatcccggtcacgccgttttatcacatttagcgcagtcatccgcaatttcctgtaataaaaacactagtactcatccttgtcatgcctgtcaactcggacgacatgttcgtctaccatttcctatttcttcctcccgcgccacctgtaattttcagttaattcactgtgacctctggacatcacccatcaccagtgtgtctggccacaaatattatcttgttattttggatgactgctctcactatttatggacatttcccttacgtctcaaatctgacaccctttccaccttaaccaatttctttgcctacgttcgtacgcagtttggtgtccctatccagagcatccagtgcgacaatggtcgcgaatttgacaaccttcaagcccgcaactttttctcctcccatggagtccatctgcgcatgtcttgcccctacacttcagctcaaaatggtaaggctgaacgtgtcatccgttccatcaacaatatcatccgcaccctcctcgttcaatccagtgttccccccaatttttgggctgcggctcttgggactgcaacatacctcctcaacatcctacccacaaaaacactcgcactctctacaccgcattttgctctcttcggtcacaacccatcctatgaccaccttcgcgtttttggctgcaaatgctaccccaacctttctgccaccactccacataaattatcccctcgctcaaccctttgtgtcttccttggctattccccccaccacaaaggctacctatgctttgatcgccactccaatcgcaccattatctcccgtcatgtggtttttgatgaaacctcgtttcccttttctgagggttcccatccacccccaccagcagccttcgatttccttgacaattacactaacccggttgcagctccttttggactgtcgcccgtgtcttctttttcaggtactcgtatgccaccaccacccccaccggtggccccttcgccggctgtgcccctcgtctcctcagaggtggcatttcagccacctgccgcgccagctgggcctgccccgatgtcccagtctcctgcccggatggctggtctcccaggcgcatcggcaccaacaatactgccgcctgggtctactaccgtgcccgacgtccatagaacgacagctcctgctgatcgtttctgcggacgggtctactctcgacgcccgcccaccgctactgctccagcaccttctccgacaaccgccactgcaccacctattcctgctggtgcagtccctgtcgatccagtgatcaaccaccacaggatgacaacccgtggcaagagtggtcttcggtttcctgctctttttgaagccgccgcactgtcccccattccgaggacatatcgcgcagcccttactgattcaaattggcgctccgctatggaacaggaatattctgctcttattggtaatcacacttgggatcttctgcctcgaccgccgacttgcaatgttgtgactggcaagtgggttttcaaacacaagttcaaagctgatggttctcttgagcgctacaaggccagatgggttcttcggggtttcactcaacgccctggtattgatttttcagaaactttcagtccagtggtcaagccagctacagtccggaccgttctttccctggctctctcccacagatggccgattcaccaacttgatgtcaacaatgcttttcttcaggggaccctgtcagaaactgtttactgtgttcagcccaccggttttgaagattccactcatcctgattatgtgtgccgtctcaatcggtcactttatggcctcaaacaggcccctcgtgcctggtacagtagatttgcttctcacttgattcaaattggattcgttgaggccaagtctgacacttcattattcgtctaccacaagggcacagatatgatttaccttcttctttacgtggatgatattgtgctaactgcttcgtccatgaatcttctccggcggactattactgctctccaacaggaattctcactgaaggatcttggcccccttcatcacttcctgggcatgcatgttcaacaatctgcttctggcatcactctttctcagcgccagtatatgattgagatacttgagcgagctggtatgagtaactgcaaaccctgtacaactcctgttgatatcaaccccaaactttcagccgacggtgaccctgtctccgatcccacggaattccgcagtcttgccggcgcattacagtacctcaccttcacccgtccggatattgcatatgctgttcagcaagtctgtctccacatgcacgatcctcgggaacctcatttggctgctctcaagcgtatcctgcgctatatccaaggcactcttcagatgggccttcacctcagttcctctccgcaaatggaacttgtggtttactctgatgctgattgggctggctgtccagacactaggagatctacttccggttatgctgttttcctcggtgctaacctcgtctcatggtcttcaaagcgccagaacacggtctcccgctccagtgccgaagccgagtaccgcgctgttgccaatgcagtcgctgaagtatcctggttacgccagttgttgacagaactgcatgttcctctccccaagtcctcattggtctactgtgacaacatcagtgctgtttatatgtcctccaatcctgttcagcatcagcgcaccaagcatgtggaaattgatcttcacttcgttcgtgacaaggtcgccctcggtgttgttcgcgtgctacatgtcccgacgacttcccagtacgccgacatcttcaccaaaggcttaccatcagcagtcttcactgaatttcggaccagtcttaccgttcgctccacggacgctaacactgcggggggg

>Copia-48_ZM_LTR

tgttagaaactgtatcctggctgtcgcctggcagatatgccggccgccctatatggaagtattgtctgatatagatagagaggattaggggaggatctcaatcactatgattgaggatctgattgaggatctcaatcactatgattgaggatctgattgtactctagatctttccttaggtgtagaaggcttccttttacctctcaccttcctcccctatatatacctgtacatgtgctgtctatcttcatcaatatacaaacacacgcttccaactctcttaca

>Copia-49_ZM_IN

attggtatcggagccaagttaactggagaagcatgtcggcgtatggccgacgcgatgacggaggaggcagaaggcgctcactgtcgccggtcttccctcgcggacgacgcggtggcagggagatcatcgtcgagcgcatcatcgagcggagctcgacggcggccatctaccccatgctcacgaaaaccaactatgccgagtggtcacttgtgatgcaggtaaatctccaagcacaatgcctctgggaggcaatggacccgggaaccgacaacttccgcgacgaccggaacgcgctggcggcgcttcttcgcgcagtaccgcccgaaatgcaggctgggcttgctcggaaggcgactgcgaaggaggcctgggactccatcaagagcatccgtgtgggcgcggagcgtgtcaaggaggcaaatgcagatcgattgcgaaaggagttcgctgatctcgtcttcaagcccggcgagtcggtggaagactttgctgctcgggcaaacaccttggcgaaccagctgagggtggtcggcgacgacatcgccgagaaggaggttgtgaagaaactgctccacgcagccccggctaatctcgaacaaatcgcattgtcgattgagaccttactcgatctcaatgacatcacggttgaagaggtggctgggaggctgcgtgcggtggagcagaggaagacgacgattgcggccccgaacaaggacggtggaggccgtctgctcctcaccgaggaggaatggcgcgaaagggcaaagatgaaggaccatggcggcaaggcagcatctagctctggctggaaacaggcctccgggcgtggacgtggccgagggcgcggtcgttcacgaggaagaggccaaggtagaggtggtgcacgcaaagacgacgagtgtctgagctgcggcaaactggggcattgggctcgcgattgtcgtagcaagaagaaggcggagcaggcacacgctgctcaggcgcaggacgtcgagggcgaggaggaggattccgccctcctcatggccacgaccgagatctccatgtcaggcggttcacttgcttcagaagaaccaggcacactggcacttcgccgtgtgcatctcctggaagccaagatcatccctcgactcgacgcaccggaggaacgcgacgagtgcagctgggtactcgacacgggtgcgtccaaccacatgaccggcatccgatccctcttctccgagctcgacaaaggggtcggcgggtctgtgcgattcggcgatggctccactgtggcaatcgagggtcgcggcaccattgtgtttatgtgcaagaatggagagcagcgagcgctgaccggggtctactacattccccgactcacggccaatatcataagtcttggccagatggacgagggaggctgccgagtcgacatcaaccacggcatcctccgcatcttcgatcgcaagaagaagcttctggtacgtgtgagacgtacagcaggacgaatttaccagctccaagcgaccatcgggaggcctctctgcttcgcggcgcacagctccgaggtctcatggctgtggcatggtcgcttcggccacctcggcttcgacaatctccggaagctggctcgggaggaaatggtccgggggttgccaaccctggaccctgttgatggtgtctgcgatgcatgccttgccgggaaacaaaggcgctcaccgttccctgtccaggctcgacgtcgcgcagcacaagtactcgatctggtccacggggatctgtgtggaccaatctcgccgtccacaccaagtgggaacaggtatttcctactgttggtagatgataaaagtagatatatgtgggtcaagcttcttgccagaaaagacgaggctgcctcggcgatcaaaaattttcagtcagctgttgaggtcgaaaccggacgccatctcaaggtgcttcggaccgatcgtggaggggagttcacctcagtggagtttgggcaatactgcgccagccgcggtgtccaacgtcaactcactgctccatactcgccgcagcaaaacggagtggtggagcggcgcaaccaaagtgttgtgtcgatggcgcgctgtatgctcaagtcgaagggtctccctgggtacttctggggtgaggcagtctccactgccgtcttcatcatcaatcgatcgccaactcgagcactctctggacaaaccccctacgaggcgtggtttggagagcgcccaccggtgcacttcatgcgcatctttggctgcgtcgcccacgtcaagaacacccgtccaggtctgaagaagcttgatgatagaagcacaccaaccatcttcgtcggctacgaacaaggttctaaggcgtacaggtgttatgatccacaagccggtcgcgtcgtggtgtctcgcgacgtcgtgttcgacgagggcgcgaagtggacctgggacagaacctcggacagacgtcttgaagatgctgtggatgatgatgtcccgttcatggtggaagacgaagtgcattctgctccaccgatttcttcacgcgcgacgacgcctgctccgactcaagctacaacgacgtcgctgtcaccagcttcaccggcgactgtccaacctgctgcgtcgcctgcctcgtcagcagagtttgtgtcaccccccacaggctttgaaggggtgctcgacgctgaccatgacgaggatgcgccgttgcggttccgggtgattgatacagtccttgggccagcttcaccgccaggattggcgcccagagtacttgacgaggaactaatgttaacgacagccgatgaaccaacttcttttgcagaagcagagcaggaggagtgctggcgccaggcgatgaaagaggagatgaaatcgattgaagccaatggcacctgggagcttgccacactccccactgggcaccgagctatcgggctcaagtgggtcttcaaggtgaagcgcgacgggagtggcaaagtcgtccgccacaaagctcgtctcgtcgcaaagggctatgttcagcgggccggaattgacttcgatgaagtgttcgctcccgtggcgaggattgagtccgtgcgagtactgcttgcactggctgctcatcacggatgggaggtccaccacatggatgtaaaaagcgcctttctgaatggggacttgaaggaacaggtgtttgtggcacagccccccggatttgtggtcgacggcaaacctgacaaagtgctgcgtcttcataaggcgctgtacggtcttcggcaggcaccaagagcctggaatgctaagctggacgccgccatggctcagctgagtttccagaggagcaggtccgagcatggcgtctacacccgttccagtagcacgtcgaggttggtggttggagtttatgttgacgatctaatcatttctggttcgagctccaaagagatcgcggcgttcaagcaggagatgaggagcatcttctccatgagtgatctcggtctcctctcctactaccttgggattgaggtgagccagaatgcacatggaatcactctctgtcaagcagcctatgcaaggaaacttctggaacgttgtggcatggcaagctgtaactcgagctcaaccccgatggaagaaaggctgaagttaagcaagcacagtaactcacccgctgtgaacgccacggagtataggcgcatgatcggtggtctgcgatacctggtgaacacgcggccggatcttgccttctcggtgggctatttaagtcgctttatggaggcaccacatcaagatcatctgatggcagtgaaacgtgtcctcaggtatgtagctggaacctgtgagcatggcattcactacaagaagagcagggaaggacaagctcaattggtcggcttcagcgagggaggacgagctcaactggtcggcttcagtgacgcggacctagcaggtgacgtcgacactcgcaggagtaccagtggcgtcatcttctatcttggagataatcccatcacttggcagtcttcaaagcaaaaggtagtggctctgtcatcgtgtgaagccgagtatatcgctgctgccactgcaacttgtcaggctttgtggcttgctcgtctggtgactgacatggcaggagtgcagcccgggacgcctgaactgaaggtggataaccaggctgccatcgctctaagcaagaacccggtcttccatgacagaagtaaacacattgacaccaagtttcactttatccgtgagtgtgtggatcaaggtcggatcatccttcagcatacgtcaacggaaacgcagttggctgacatcctgacaaagccgattggaaagacccggtttcataagctgtgctccctcataggaattcagcaggtcggcgagatcatggagtagaggggagaaa

>Copia-49_ZM_LTR

tgttgcatactcctagctctcgccgagtttgacaattttgtcagttaattcagattattcagttagtgcagttcagtcagttagtgttcgtcagtctgttagctaattcagtttcattcagagttagcagtgagcgcacgccggaaatagctggacgtgtgcgcgtctttcggagctcacctgacgcacgctgcgttcgtgcggggtggagcgcgtcgtgcgcggcaagaacgtggtcgtgggatggcacggctgtagaagcgttcgtgccgtgcgtttcggccttctttgtttttgatcagataagtccttcgatatatgaataaacgagtcacagaaaagcgcggccagttgggcagcaccaaaaacaactctccacgagttctcgcgcatgtgtactgcgtcctcaggattcatcaccggcgatcatctccgatctagtccaaca

>Copia-50_ZM_IN

tggtatcagccttcttcgttcctacagcaatcctgtgtctcaccttccacatcaatggctcactccccactcccctccgatgactcctctgatgacgagctcgtcgttccagcacccgcttccgttatccaaagcattcccatccgccagcacgttcctgtcgtcctcgacatggatgaggggaactatgggcaatggcgatgcttctttgaatctgcgctcggaaaattcggcctcaccagccatgttcgttcttccaccccataccgtgaccgtcctggtgaatggcgcatggtggattcctgtattgccaactggatcctcaccacagtctccaagggcgtcttcgacatcatccgtcgcgactgcaatgacgccttctctctgtggcacgccatcgaagacctgtttcaagacaacgaacttcagcgtgctgtgtaccttgaagccgagctgcgttccctgcagcaaggcgacttgtcgatgaatgcctactgcaccaagttgaagcgtctcgccgatcaacttcgcgacatcggccatcccgtctccgaacccagtcaggtgctcaaccttcttcgcgggctcaatccaaagtaccgctacgtcaagcccgtgatcacctccaagttcccagcgcacaccttcatgagcgctcgctccttcctcatgcttgaggaggccagcatcgaacacgatgtcgccgttgaggccacccacgcactgactgtcacacatggtgactcgtccagtgctgttcctccatctgcatcttctggcaccagggacaactcctccttctccaacacgccacgccatgacaatcgcttcaacgctggtagcacctcccgttctaccaaccgatctgatcgcaggcgcggtcgtggcaacggcggtcgtggacgcttcaacaaccagtctggaccatggacagcaggtctcaatccatggcaaggcatggttcaggcatggcaaatgcctttccgtgcacccggagcaggagttctggggccacgcccgccgttccaacctcagcaagccatggcagcctatcatcagccacctcctgcatctaccggcaccttcgacaacagcgctctttatgcggcactgcaggctgccgcagttccaactcatccgcccaacactactgactggtacttcgacaccggtgcgacgtcgcacatgtcgtcttcccctggtaactttccaccctcttcccttctccctttttcgtcttccataacagtgggtaatggtgcccagctacctgttacacatcatgcacacacatccattcctactgccacttcccctcttcagttacatgatgttcttatttccccgtcactcgttaaaaatttggtttctgttcgccgtcttactcgtgataataatgtttccattgaatttgacccttgtggtttttctatcaaggatcttccttccaaagcggagattctccgatgtgagagcaatggcgatctctatccactccgtcttccccaccagcatgctctcactgcatcctcgatggcgtcgttatggcatcagcgactgggtcatcctggacaaccagtcacctcccatattttaaaatccctgtcttttcaatgtaataaagctgatgctcattcctgctcctcttgtcgattgggcaaacatactagattaccttttcgtgtttctgaatcacgttcatttttccctttccaactagttcattcagacgtctggacatctccttgttatagttattccggatataaatattatgttatttttctggatgattatacacattatctttggagcatccccctccgcaacaaatctgacgtttttcctactgttagagcgttcatatcttatgttcatactcaattccacctccctattcttgcgtttcagactgataacggtggggaatatgattctactgctatgcgtcttcttctgtcatctcttggcacccaactacgtctttcttgtccatacacatcccagcaaaatgggaaagctgagagaatacttcgcactgtcaatgattgcctgcgtacactactaatccatagtgcagctcctctcgcactttgggcagaagctcttgctacagctacgtacctgatcaaccgacgcccctgccgtgcgacccgctctacaacacctcacgagctcctctttggcgtggcgccatcgtataccgagctccgggtattcaggtgtcgctgcttccccaacaccaccgccgtgtccccccacaagctcgccgcgcgctcgacaccgtgtgtcttcatcggctatccagcagaccaccgcggctatcggtgtttcaacatcaccacgggtcgtgtcatcacctcccggcacgtggtcttcgacgagggagttttccctttccgtgacacctccaactccactctacaacaagcaacgcgcgcgtcctcctcgtcagcagtatcgtgtgatactgacagcgacatcgcgccgcgcaccggaccttcgacgccgtctcccccacgcctcctcgacgtcctgcttcagcatcccacacgcgccaggactgcacgtccggggcgtactcccgtcgctggttcttcaaacccctccgccgtggctggttcttcgaacccctccgccgcggctcctctggcacctgcagcaccgtcggccacgccatcatcgtccaaccatcacccgactcatccaatggtgactcgtgcacgcgccgggatctccaagcctaacccgaagtacgccttcgtgaccacggagacaatattgccgattccccgcagcgttcgcaccgctgtcaaagatccccattggtatgctgcaatgaagtccgaatttgatgcactgcaagccaaccacacttggactcttgtctctcggcctccaggtgctcggatcatcaccggcaaatgggtcttcaagcacaagatgaatccagacggcaccctcgcgcgatacaaggcacgttgggtcgtccgtgggttcaaccagcggcccggtgttgatttcggggagacgttctcaccggtggtcaagccggccacaattcgtaccgtgctgacgcttgtggctactcacaactggccagctcaccaacttgacgtctctaacgctttcctccacggcaaccttcaagaacaggtgtacagtcagcaactcactgggtttgttgatctcagccgcccagacgacgtctgcctgctctccaggtcgctctatggtctccgtcaggcaccacgggcatggttccagcgtttcgtcgagcacgtgacatccctcggcttcattcaatcgaaggccgattcatcgctcttcgtgtatcaccaccatggcgagacagcttatcttcttctctacgtcgacgacatgatcctatctgcctcgacgcgtcgtctggtccagcatgtcatcgcgcgtctacacgatgccttcgctgtgaaggatatggggcctgttcaccattttctcggcatcggcgttcgacggaaccgcatcggcttcttcctctctcaaggacagtatgctgaagatctcctcgagcgtgcaggaatgacgaactgcaaacctgttgccacccctgccgacacccaacaggaggcatccgctggcgatggcatactgctcgacgacgccacttcataccgtagtatcgtcggcgccctgcagtacttgacgatcacccgtccggacattgcctacgctgtgcagcaggtgtgtctccacatgcacgcgccgcgggacgtgcatctcaccatgctcaagcgtattctccggtacatcaagggcaccatccacttcggcatccagctgcgcactgcctcgccttcgacgatcactgcatactccgacgccgactgggcaggatgccccgacacacggcgctctacatcgggtttctgcatcttcttcggcaactccctcgtctcatggtcgtctaaacgacagaccaccgtctctcggtccagcgcagaagctgagtatcgggccatcgccaacgccatcgccgaatgctcctggcttcgccacctcctttctgagctgctttacagggtcccctcagcaacagtggcattctgcgacaacatctcttcggtgtacatggcacgcaatcccgttcatcatcggcgcactaagcatattgagctggacatccattttgtgcgggaaaaggtcgccattggtgagttgcatgttacgcacattcccagtgtgcgacaaatcgcagatgtgttcaccaaaggtctaccttcggcactgttcaacgacttcagagacagcctctccgtcaccaacgcgaccgtcgagactgcaggggg

>Copia-50_ZM_LTR

tgaaagcggactacggttactaccgttccgctgtgaccgggtctcttgcgtatactcccacgactcgcactactccgtattgtacgcaccactctaacggcatagtgtgcgcaccactcaaggacccagtcctctcggtgcccagttcatgtaatctatatatgcaccgtgagttatcaatacagagattgagttcatcgtattcattctaca

>Copia-51_ZM_IN

tggtaacagagccaatggttttctgaatgtccaccatctcctccacaatatcctcctcctcccctgccaccaattcctccgtggtgcccatggctgccaactcccaatctgctatggcgtccctgtcccatcatcaagctgtgacaatccgactcaacaaggggaattatctcctctggcgcgcccagcttctgccgtttcttcgtagcacgaagctgctgggccatcttgatggcaccacgccggcgcccctgatgttcgtcgctgcatccacggcgaccggtgccggacaaatttccaatccggagtacgagcgttggtataatactgaccaacagctcctgagtggcctcttatcctccatgccggaggaggtgctgcgcgacgttatcgatgccgcctctgccaaggacgcctgggactcggtacagaagcgcttcgcgtcctccacacgagcacgcacggtgcagatccgtgtggaactggcaacatgcaagaaacgcgacatgacagcagccgacttctttcacaaggttcgtggcctcgccaccgagctggctgctgctaatgctgctcttcgcgacgacgagatcctcgcatatttgtttgcaggattacctgctgaatatgatccgtttgtcacgacgatgacaactctgcccgccgctccaactcttgatgatgtcttcgcccatctggtggcatttgaagcccgacaacttcgtcatcatgcggacctgcaactgaacctcggtgcctcagcaaactacgcttgccgtcgaggatctcatcgtgatcgtggtcgttctggccgtggctgtggcgcgccgccccgtggacgaggtggacctcctcgagctggtggtcgtggatccatctctcgcccaacatgtcagatttgtgagaaagaaggtcacaccgctcttcgatgctggtaccggatggatgactcctatcaggaagcaaatccatttgcggcacttgcttctacatcttcgtacaaggtggatgccaactggtacactgacacgggtgcgactgatcacatcaccagcgatcttgatcgtcttactgttcgtgaacagtataccggcggtgaacaagttcaagtcggcaacggggcagtttgaagattttacatattggtcattcttcaattaatactgttgatcgccctcttgttttgcgcaatatattacatgtgcctgaaattgctaaacatctgctttccgttcataaattctgccttgataataaagttttctttgaattccatccctggcatttttctattaaggatccaatctcccggaaaatgctcctggacggacgatgtgaagggggtctctactctctaaagccatcggatgttgcagatttgaagcaagctcttcttagtcggttgtctcctagtcgtgctcaatggcatgcacgccttggtcatccatctcctcaagtggtgcagtcgattttgcgtctgaataatatagcgtgtcctccacagtccgatgtgcaagtgtgcaatgcatgtcaattagccaagagtcatcagttgccctatgttagctcgagtcatcgtgttttgtcccctttggaactcatccactcagatgtctggggtcctgcgcctcaatctgttggtggttttaaatattacattagttttattgatgagtttagtaaatttacctgggtttacttgatgcatgatcgttctgaagcctctcgtttatttcgccagttccaaacacatattgaacgcctcttaggcacaaaaatcaaaactgttcaattcgattggggaggtgagtaccagaaaatccacaatactttctttcgttccttgggcatcacacatcgtgtttcctgtcctcatactcatcaacaaaatggttcggccgagagaaaacatcgccacatagttgaaactggattggctctcttagctcatgcttctatgcccataaaattttgggatgaggcttttctcactgccacatatttcattaatcgcatgcctactcgggttattgataacaagtgtcctcttgagcgtctttttaaaacctcacctaactactctcttctcaaaatttttgggtgtgcatgctggcctcatcttcgtccatacaccaaacacaaactttcttttcgatccaaagagtgtgtgtttcttggctacagtgctttgcacaagggctacaagtgtcttgatgttgattcgggtcgtgtatatatttcgcgtgatgtaatctttgatgagggtgtctttcctttttctagatcaccaccacattctccttcatccatgccagcatccactcctgtttttcatttcagcgcacctcaattgggcaccaacagtactaatacgaattatgaccaaatacaaatatctttgcctgctaaccctttaattgcagaagtttcggcacctaccatatccacttcgggaattgcaacatcaactgaccacctggagttgatgccattgggatcgccatgcccccctgatccatctgctccaccggctccagatttgctccatcctctggcgcccgatacctttgcacacgtttgtcctggtgctgctgcgtctgccgatttgttggctcctgatgatggggtgcctcctgctgcttcagcattacagccacatccctatggtacacgacttcaaaacaatattcgacaaccgaagcggcgtacagatgggacagtcgcatactcggtggtacgtacctcctcttccgagccaacatctcatactgaggccatgcatcacccatcttggcgacaggcaatggttgatgaatttcaagctttaatacgcaacaaaacatggcatctcattcctccacgtgaagatctcaatgtcattgattgcaaatgggtttttaaactcaagcgcaaagcagatggaaccattgatcgccacaaggctcgtttggttgccaaaggatttaatcaacaatatggtattgattatgatgagacatttagtccagttgtcaagccaaccactatccgtctactgctgtctcttgcagtgacacgtggatgggttattcgtcagattgatattcaaaatgctttcttgcatggttatcttcatgaagatgtttatatgcatcaacctccaggttttgtggactccaaatatcccaattacttgtgtaaattggataagtcgctatatggtctcaaacaagcccctcgagcatggttttctcgattaagtgacaagttgataaacattggtttttccccatcaaaggccgatgtctccttatttattctcaagaagcaagacattcgtatatatatgctgatctatgtggatgatattattattattagctcctcaaatgctgttgtcactcgccttcttcatcagcttcgagatgactttgctgtcaaagatcttggttccctacactacttccttggtattgaggtacgacacactctgcagggagtcttcttgatacaacgcaagtatatacaggatctattaactaggacgaacatgctgaattcaaaaggagttcctactcccatgcttccgaaggacaagctgtcattacaggggggcaccccattatcatcagaagatgccactcgataccgcagtgtggttggctccttgcagtaccttgcgttaacacgaccagacatttctttttgtgttaatcgtgtgtgtcaatttctctctgctccaacaactgagcactggtctgcggtcaagcgtatacttagatatcttcatgacactattgatcaggggctttacttcactaaatctggctcctcgctactcagcgctttctcagatgctgactgggcacgcgattccgatgaccgacgaagcaccgggggttttgcaatcttttttggcggcaatttgatttcgtggagctcaagaaaacaacaaactgtttctcgctcaagcacggaagcagagtataaggcgattgctgatgccacagctgaactcatttggcttcaagtgcttcttcgcgaaattggtatcaccctatcacgacctcgtacactctggtgtgacaatattggggctacttatctgtccgccaatccaaattttcatcgacgatccaagcatgttgaagtggattatcattttgttcgtgagcgtgtgtctacgcgccaacttgacgttcgggtcatctccatgaaggatcaaattgccgacatcatgaccaagccacttccggtgacatctttcaactcttttcgacgcaatctgaacattgtcactctacgtccagattgagggggg

>Copia-51_ZM_LTR

tgttagatgtatagcatgtatctcgtatatatggttaggatagaaccctttccatcctaggagctgcgtctcctttattctctcctcaattatctcatgctgttactatcgttgtacacggtgatcatctatataagaagggccatcgcatcccattattgtgcgctgcctatcacaaccaaca

>Copia-52_ZM_IN

ttcttcatggtaatcagagctaggttcaacttcatccacaattaaacactactagaaaaggtagcgccctatcgggcgctatacttgccgccccctctcgtgtttcaggcaattcttaagcaaacaaaacaaagtattttatccaataacaaggaaccatcacatctgtatggaacaaacatagtaagcacaaaaccgcaacatgcacctcggtgcaggtatctgctacaaaggttgtggattcctagaattgagctatattcccctaacacaaaagtaaggaaatgcagaactaagaaaaatgtgtctctcagagtgttgtagaaggtgtttctgtttttgaagtgcagtgctcactggtcgtattcgtgataaactgctcctgcaactgaaaattgtgggtaaatccagacatccagttcaatagcacgcacatgatcccattcaaaacaacaaaccaaagagaaacaaaatcaaacaacttgaaataaattacagaatattatgtcatcgtatatgtgcaaatggctaatttgatcaacaataatgatactcgattttaaagatatctcccagcacccgatcctcagggagtcggagagaagggaggagatgagagagtttttttaccaacctctaggagttccaaaaaaacttaccgcctctgggatatggttccatgaacttgagaagagcaataagcagtctaaaaaatggccaacctttctatgaattgcacatgagcattttttggcatgaagctcctgtgactcaccagctcaagccaagcaaagctacataatacagaagcaactcactatattggcacaacatcatacctttgcttaataatgtatatagtgcacaagtaaattgtcttcctaataaagcaacagacttatgcgtacatgtctaacaacatcatcattcctgggaaaatattatattatgtttgactgcataagaaacaagcaccgagtgtcccacatacatcaatgtccttgacctttacattcttagttttcaccaggcttggattctcaatctcgatctgacttgctatgcctttgtgtttctaacaatgataatgaaagtagtttatcatatgatcgaacaacaatgaagactatggaatctgtatatttcaaaatgatataacgtcatactggtttcgtaaaatcttctgattattcttctggtccccctttcactcttctattgtagacatccttctcatcatgctatattttgattacagaaaacaagtcaaccaccaaaataggcaggaggtacagttactaaacagagctcggtattgttgctgatatgttaagcagatttgttgagcagaacactgccaaacttaaatagatgcataaccaatgtagagggaaacacacattttaactgtagactgcagtggtgcttaccgcttggagcctacactgagggttcttacagtaaaaaaatatgtagatgttatattagcagcataatatggaggatagcttccatcatgacacgataggggccctctgcataggaacaaaagaagctgatcaaaatgcatcgacaaggcttatctgtaagtgtagataatagtaacaggaataggacaacaaagcaatctagacaccgaaaggtggcctcattaacaacttggttctcgagtcgagggcgaaccttgttacgacaatgttggccccaaagtggttctgaaatatatttaatgataaaatcaatcccatcagcactgaatacaaactgtattttctaaaatatattttcagagaagaaagcatgacaatccacaagttgaaactaattcttgtgaaaagccaacataaacattagtttgcaatcaacgtttaggaagctttactttttctcaatatttttaatgttgtaaggtgatcttatgacatggtcatataagagtgcccaataatgatgccttaaatgatacttgatgatgcaaatggtggatgttctttcttcaatcggttccttcaataaagcagaaaacaaaatatatagtcttcaaaatcaattcaatattacattatatacccactaaatactatagaattcatggtttcattcggtaatgcttgaaataactttctgactggaatagtctgaatgctaacatcctggtttgacagaacaaaacatggtaaacagaacacatatactttagctaaaagttaaaggaatactgcttcatctaatgacacatggtactcatacaggatggaggtagctcatatgcctttgtttcttatagttgaataaaaagtgttcaggaaagtcattagcaaatcttagtagggatgatgttatgcaaacatgaacaaattactgctataccaattagtgttgataccttgttgagctcgctctaccgtggctctctggttcgcagcaagcggaagctcgagatccaccccgaagtcccgaacgcttgggcgtaggtggtctatgggcaaaaacattgtgtgatggtaaaagttttggggataaaagtagggtacaaatagaaaaaaacaaggtgcctttctcttagtatgcagaaaataacagcttcggtgatggtgagtgtaccctaaagatcacaaagcgtatcaaactttcactcctctttcttaacagcaacaaagaattgtttgataccctacaaacatgacactaaaaaacttagtagccaaaaaggcaccaaaatagttcaaagcataactctcaacggtattttacctctagggtgagctcatcacacttcacaagggtcctaactggtttagtcgagagctggctgtcaaaggtctaccaatcctagacagtattgcttatataagtttggtactatgcttctaagtgaagtgaacatgcaggttcactgcttagatcaaattgtatatctaattttggaggagcatcgtcactacaaccacatagatctgttgattagtctgtaactccctgtgcaaaagtaggtgtgttgatcttaagaactctgaaaccacacaaagtgttgttgtcttatagtaaaagtggaatgtgaaataaaaaaatgggtcgatcttaagaactctgaaaagagacaacatggtaagcaggtagctcaagacaaagaataatatattctagtatattcaagacaaataatagttggagccttaaatgacaagctaaggtaagctaaaaacaataaataaattgaaggacagtgccaatggagtcttaattctaacaaagggccaagctattagcacatccgtaccttgtgggttagcagttcccatagaacaatagcaaagctaaaaacatctgctctacgatcgtagggcttatgttctatgacctgaaatttcaataataacatggaatataagaaaaggcaaaatatgcatttttttcttctgaaatacatattcatgacggtgcacctcaggtgccatccaaatctaagtttcagtttgtacagtcatcactctagattggtcttccacacgtgcaacaccgacttatgaaacctttacatcctgcaaaatatattccaaatactttttagtggcgtcggtcaccactttagtatttcaggaaacctggtagcattacaatttttatggcggtaatcatacacactccctcctgacacgaaatctaaccaagacattaaaaaagggtacggggattagacaccgcacaaatttattaacatatctactgcaaagatgaatcccactgttgtgtacctgttactatatacaatgttgtttgttttgtccatgaatcgataaagacagtttgtgtgaagaatgttgatttagatttagcttttcctacatgcttgacatgttaacacaaaataaaatcaatgattgaaggtgtaaacatactcatcggatggcccccgagctcaccagggcacggtcaagcagggagaaagcggacgtgtccaggttcagttcaggagcacacacgcgtgcgcaggcatccatttgggcgctacgacggtggcagcctccaaggcagcggagaaggaggggggcagggaaggggaggccggtgatctcctgcaccatgctttggaacttagcggggccggtcgtgatgtaggtggtccgcgggcgccgaacgagcgtggcttgcgcttggtgacatggccccctctaggtctcgccagggccccctgcacatcgacgcaggagctggtgtccgctgtataggagctcggagacaggaggtcgaggaggacggggaggagtaggcatgggcaggcgccatggaggcgcagaggtgagtacctcattctcaaaggcaacgcagaggtgaggcagccgagggacgagctgtagtagcgcgagatggaggtgatggtggcgtcggcgtcgacggagcgaacggcgtcgaggaaaggccgtagcacgaaggcgggagcgcggcggctggcgaagaagagggggagaaggcgaagcggaggagcgcgtggagggaccgagggaggtggatgagagcggacttcaaccagatcgacgcactaaaacgaatgaaaagttggagtccggacacgcctacgcatcgtcacggagcgctgtggtacccttgaaacgcggagagagctcgaaaggggttgaggttttaaggaacaagatttctcttcttcttccccacccaccctcccatggcgtccggcgttctacagcctgacccctctgctactccccaatccggtgcactcatttcatccctcgtcagcgcagcccagtcagtaaacatcactagtcttgttccaatccgcctcgacatggtctccacagcctaccggcgctggcgacgtctgttccacgtcgttctaggccgcttccatcttcgtcaccatgtcgacggcacgccaccacgtccggacgatgctgcctggattcaagaggatctcactgtcctcatgtggctccatgccacgctcgctgatgacctcctcgacatggtgatggatgacgacgacggcacagctcatcaggtctggtccaagatcgcgaacttcttcctcggcaacaaagacagtcgcgctgtccaacttgaacaagaccttcacaatctggagcaaggcgatctgagtgccgctgcattctgtcatcgtctcaagaccctcgccgatgctcttgccgattgcgatcggcctatcgacgatcgcgcgctggtacatcaactgatccgaggcttgcaccctaaatttcatgtcctcaagcagatgctcccggcgatgccttccttccccaccttcatggaggcgcgagatcatctcatcgtcgccgagaacacgcttgggtcctccaaagcatcgggcaacgaatcagccctcacggcgaccacaaccacagacaacagtggcggcttcaccaactctccgtcaactcgtccagacggcgaccgttctccatttgcctcccgtggtcgtggccgtggtggcggtggccgctggaacggccgcggtcgaggtcgtgggggccgctatggattcaacccccgtcctccccaactcagcccagctgctcttctgcagcagctgcacgcctggctcatgtctggatctgcctggcgtgcaccctggacggggactacaggccctggtgttctgggaccgcgacctcctgttgctgctcatgcgtacaacgccgccatctccccacctctggcaacaccatttgccactccgaccacgcagatgtcaccagccacgtcgccatctttcgacacgacggcacttattcacgctcttcatgcggcttctcagcctcagcagcctgcccaatctgactggtttatggacaccggtgcaagcgctcatatgacaagtgatccaggtaccatgcccaattattgttcctcatcattgcataattcttcatgcatcctggttggaaatggttctgctcttccaattttaggcagtggcaatactacccttcgtactccccaccgctcctttcatctttcatctgttcttcatactccccacctcatcaaaaatctgatctctgtacgcaaatttactgctgacaattcttgcactgtcgagtttgaccagtttggtttttccatcaaggatcttcgcagccgagccgagatcatgaggtgcagtagccgtggtgacctctacccgttccagtccagttcgtctattcaggctccaatggcgctccacacctctacaccatcgaccttgtggcacagacgtcttggccatatgggacacaatagtcttgatcatattttatcgcaattttctatttctaataatcgcacacatgtttgtgatgcatgccagaaagggcgtcatgttcgcttaccattctataattcgcagacgccaacctattttccattccaaataatacattgtgacctttggacctccccagtctcaagttttacgggctacaaatattatcttgttattctcgacgattttactcattatatctggacgtttccgatgcgccataaatctgatgtcccttctattcttatgaatttctatgctttcactcaaacacagtttcatcttcggattcaatctgttcagtgcgacaatggcactgaatttgacaataaaactcttcgttcctttctctctaaaaatggcaccacatatcgtctttcttgcccctacacttcagctcaaaatggcaaggccgaacgatctattcgcacaattaatgatattctacgtacattactcttccaggccgatctgcccccttcatattgggttgaagctctccatacggccacgtaccttctcaatcgacgtccatctaaatctataaattttaacactccttaccaaattctttttggtcaatcaccggactattctcatcttcgcgtgtttggatgtctttgttatcctaatctttccgccaccatgccacataaattatcccatcgttctgctccttgtgtgtttcttggttatcctgcagatcacaagggttatcgttgcttaaatcttaacacaaacaaaattatcatttctcgccatgttaattttgacgaggatactttcccatttcgcacacagcagccacttctgaccacacccacaacagatcccaacaactctccctcagttctcgttccagggcctcccgtggtgctacttccctccaattcagcttcaacgtcacataccacttcatgtccaccgctgcaacctgattccactagggcccctatttccacgccacgtacttctccacatgcctctaccagcacgctcccgtcagctccagtcttccgttacgcatcaccggacatcacacgtcatcacatgcagacccgaggcaaatctggaatttttaagcctaaagtcttcacgaatcttctgtccacaatctctcccattccaaaaacatatcgttctgccttaactgatccccattggcgccaggccatgcacgaagaatataatgctctgatgtctaacaatacttggtgcttagttcctaaacctgcaggtgcaaatattattactggcaaatggatttttcgtcacaagtacaaccccgatggctcactggcacgctacaaggcaagatgggtggttcgtggttttaatcaggaatatggtgtcgattatgaagaaacattcagtccagtcatcaaaccacctactattcgcacggtactgagtattgcgacatcacattcttggcctattcaccagttagatgtaaaaaatgccttcctacatggcaatctctctgaaactgtttactgtgttcagccttcaggctttgttgatccatctaaaccacatcatgtgtgcaaactagtcaaatctctttatggtctcaaacaagcacctcgtaattggtttcttcgctttacttcatacatccggtctcttggtttccaggcttccaaggctgatacatctttgtttattcttcacacgtctacaaacactgcatatctccttctttatgtggatgacataatactcacggctaacagtaaccagtttctatcttccattattcttagtctttgccgagaattctctatgaccgatttaggtccattacgccacttccttggcatccaggtccaccagactcgaactggactttttctcagccagactcaatatgcaaaggatatccttcagcaggcaggcatggctcactgcaatccggtttctactcccatagacaccaagtccaaactttcttcaactactggtgatccggttgatgaccctactgaataccgtagattggctggagcgttgcaatacctcactctcaccagaccggacatcacatatgctgttcaacaggcctgcttattcatgcattccccacgcacgacacatatgcaaattctgaaaagggtgcttcggtacatctctggtaccatcaatcatggtctgcatcttcataagtcctctggccaccaccttattgcttactcagatgcggattgggccggatgccctgacacaagaagatctacctcaggttattgtgtattctatggcggcaaccttgtgtcatggtccagcaaacgccaacctacagtgtctcgttcaagtgcagaggcagaataccggggagtggccaacgctgtgtctgaagcctgctggcttcgccttcttctgcaggaattgcgcagacctctaactcaagcaacagttgtattctgtgacaatgtcagtgctcagtatctatctacaaatccagtccaacaccagagaactaaacatattgagattgatcttcattttgttcgggacaaggttgctctcggcgaagttaaggttcttcacgtaccgtctgcctcccaatatgcagacatatttacaaaaggattgccgtgcattttattccaggaatttagagacagcctgaacatcaaggatggtacaggctgaggggggctattggagtatatcttct

>Copia-52_ZM_LTR

tgtaactgtagctgaccgattcttatgcgatgaccgattcttaaggctgcatgcaaggcatcgcggttagtagctcagtcatcgtcctactgtaaacattggcctataaaggcttcagtgaaatgtaatagatgtgtgcgctgttgtcca

>Copia-53_ZM_IN

attggtatcagagccaggttgcccgaccttggaggctgaaccatgtcggcccatggtcgacgcgacgatgcagacggaggaagacggaggagatcgctgtcgccggttttcccgcggggacggcgcggcggcagggaagtcgtcatcgagcggatcatcgagaggagctccacggcagcgacctaccccatgctgtcgaagacgaactacgccgagtggtccctcgtgatgcaggtgaacttgcaggcccaaggtctctgggaggccatggaaccggggacggacaacttcagagacgaccgcaacgctctcgcggccctcctccgagcagtgccgccggagatgcaggcgggcttggctcgcaaggcgtccgcagcggaggcgtgggactcgatcaaatcagtccgagttggggctgagcgcgtcaaggaggcgaacgccgaccggctgcggagggagttcactggcctgtcgttcaggccgggtgaatcggtcgaggacttcgccggaagggcctccacgctcgcaaatcagttgcgagtggtcggtgatgacatcgccgagaaggaggtagtgaagaaggtgttgcattctgttcctgacaaccttgaacagattgcgctgtccattgagactctactggacttgaacgacaccacggtggaggaggtgaccggacgtctccgggcggtggagcagcgcaagacggcggctgcaacgactaccaaagaaggcagcggtagcttactcctcaccgaggaggaatggctggcgcgattcaagtcaactgatggaggaagccgaacaggctcgagctctggcgcgaagcagtcctctggacgcggccgtggccgcggccgtggtcggtcgcgtggcggaggccgcggcggtggccgtgcacgcagggacgacgagtgccacaattgtggcaagctgggccactgggctcgtgactgtcgagggaagaagaaagtcgagcaggcgcatacgactcaggcagaggaagatgcagatgcagccctgttgatgatcacggcgaacacagccgtgacgaagcccaagacggaggtccacctcgacgaaggcaagctcttcgttcatctcggggaagggagcaatgatgactccacgaggtgggtcctcgacaccggagcaacgaatcatatgacgagctcgaaggaagccttctccgacatcgacgctggtgttcagggcaccgtcaagttcggcgacggatcagtcgtcgcaatcgaagggcgcgggacggttcttctcaactgcaagaacggcgaacatcaggcgctcacggacgtctaccacattccgcggctcacgaccaatatcgtgagtctcggacagttggaggaggacggattcaagatcctcctgctgaatggctttctgaaggtgtgggatcggcgccgacgactgcttacaaaagtgccgagggcagcgaaccggctctacgtgctgacggtcgacatcggcaagccggtttgtttggcggcgcagggaaatgatgcagcatggcggtggcatgcaaggttcggccacctcaactttagatcgctgcggaggttgtcgcaacaagagatggtaagggggttgccgtcacttgatcatatcgatcaggtgtgcgacagctgcttggcagggaagcagcgaagacggtcgttcccgactgaagcaaaattccgcgcaaagaacaaactggagcttgtccacgctgatctgtgcgggccgatcgcaccaattacaccaagtggcaacaaatttttcttactgttggttgatgatttcagtcgctacatgtggctgatcttgctgagttctaaagatcaggcggcggagtcgatcatcaggcttcttggcggcgtggagacgcaggccgggcggaggctggggactctgcgcaccgatcgcggtggcgagttcacggcaaggagcttcgccgactactgcgccgcgcaaggcatcgccaggcatctcaccgcgtcgtactctcctcagcaaaacggcgtggtggaacgccgcaaccagaccgtcatgggaacagcgaggagtatgatgaaggcgatgcgaatgccaagctggttttggggggaggccgtaaccacggctgtcttcctcttaaaccgcgcgcccacgcaaagtgtagaaggaaggacgccgtttgaggtatggcatgacgccaagccacctgttcattttcttcggacattcggttgtgtagcacatgtgaagacggtcggcaagtatctgtccaagctcgaggacaggagtgtgcccatggtgtttatggggtatgaaatcgggaccaaggggtaccgattctataacccaacaacacagtgtgttcttgtctcgcgtgacgccgtgttcgaggaggaacgcgcgtgggactggactgctgaacacccaagcgcgctggaggatgcctttgagccattttgtgtcgaacacgacgtggctgcagtagcaggaggcgtactggaagggacagcttcaccgacgacgccgaatgcggcgttggcgacgccaccttcgctgtcggcgacctcgacgtcgaggacgtcaacacccacgtgcgggtcagcatcccgcacacctcaggcaccggggtctggctcaggcgcccgatctgcattactgccggaggttgatgacaacctggacgttgaccccaaccacgctcctcttcgttatcaatccatcactgatatcatggggcaagaaccagcacctggacttgttgaacaggcgtcagatcaagagctactgatggcaactgggaatgaaccactcactacagaggaggcaatgagtagtgagcaatggcgaacagcaatgttggaagagatggcgtccatagaacacaataagacctggacattggtgaaccttccagcaggacagagagcaattggtctcaaatgggtgttcaaaatcaagcacaatgagcatggggacgtcaccaagtacaaagcgcgtctggttgccaagggctatgtacagaagaagggcatcgactttgaagaagtgttcgcgccggtggcgcggatggagtcagtgagactcatgttggctgtggctgcacatcgaggttggtcagtacatcacatggatgtgaaatcggctttcctcaacggggaattggaagaggaagtttatgtctcacagccaccaggctttgtggctgcagaacacaagaacaaggtgctgaagttgcacagagcgctttatgggcttcgtcaggctccacgagcatggaactcaaagttggatacaagcctactgcaattgggatttgcaagaagcaggaatgaacatggcttgtacacaagagacactcaacagtccagattggtggtggggatttatgttgatgatctcatcattacgggggagtcaactgaagcaattgatgtcttcaagcaagaaatgaaatcactattcagaatgagtgatttgggtggactcacctattatcttggtatagaagtgagacaggaaccaaactgcatcaggctatcccagacatcctatgctgtgaaagtattggagaatgcaggactgagcacctgtaacccaagtgaaacaccaatggaaccaaggctcaagctgctgaaggacagtgattcacctcaggttgatgcaaccaagtacagaagcctgattggaagtctgcgctatctgatgaacacaaggccagatctcgccttctcagttggatatttgagtcgtttcatgcaggatcccagacaagaacacttggtggctatgaagcgagttctcagatacattgcagggacagtgaaccatggacttgtgtacaacaagatgggtgaagaactaagtctgactgggtacagtgacagcgaccatggtggagatgcaaatgacgggaagagcactacaggtgtgatatttttcctcaacgaatgtcctgtcacttggcaatcacaaaagcagcgagtagttgctctatcatcttgtgaggcagagtatatagcaggagcagtcggtgcttgccagggagtttggcttaaacgtttgttggccgacatcctgggcaccaacttggcttcaccagtcttgaagatggataatatgtcagcactggcactgagcaagaatccggtactccacgaccggagcaaacacattgatttgaagtatcactacctacgggagtgtgctgaaaatggagaagtgcagcttcagtttgtaagatcagaggagcagtgtgcagacatccttaccaaggcgttggggaaaacaaagttccaagaatttcgaaagaagataggagtggtcaggcatgacggcgactgatctcagggggagaat

>Copia-53_ZM_LTR

tgacagtgagatgcagacgccagtatgctgcattcagagtttcagtttcagagtctttaatcgttctttgtttcagtttcgtcaggtttcttcaatatctagtcagcttggttgagtcgtagttagtcagtgactaagcttgtaattagggagtctgctagcatcatggacgtggtcaaggatgatccatgcgtcacgttcgccgtgggatggcacggagctcacgaacgtggagcggtcatgcgtcgtgcgcgaggaaatctcgcatcactctttctgttcgttttggtatgaatacagagtcaaaaaaactgtggcttaaccgcaccaaaaaaaggatctcgtgtattccagccttcgcgtttgtgtcttgtgtgcgtcgacgttctcaactccggcaacaccttgtcggacttagttggaaca

>Copia-54_ZM_IN

cgtggcatcagagccgaggttggaggaaggtgacaatgtccctgccgccggagcgctccaggacgtcgcagtaccggcgagggcgttcaccgccgcgacggccgcggactcgggaggtcgtcgtcgagcgcgttgtggagcgcgtcgtggcgcctggaacgaccacttatccgactctcacgaagacgaactacaacgattgggcgttactgatgaaggtgaagatgcaagcgcgcggactatgggaagctgtggagatcggcgacgtcgacgaccatcacgatcggatggcgctcgacgtcatctgcagcgccgcgccgccggagatggtctccaccttggccgtcaaaccgacggcaaaggatgcctgggaagctctgaagacgctgcgaatcggcgatgaacgcgtgagaaagtcgacggcacagaggctacgtcgcgagtacgagatgctctcgttccgagacagcgagtcggtggaggctttcgcacttcggctcaccgacgtcgtcgcgcagctcgccatgctgggtgacccggagtctccagagaaggtggtggagaagtatcttcggatcgcgcgtccaaggtacaagcagctagtaatttctatcgagactctactggatgtctcgaccctcacggtggaggacatcaccggccggctgaaagcggccgaggatgctgacgatgcaccaccaccttcgacggggggtaaactgtacctaacggaggagcagtgggaggcgcggcggaaggagcaacagaagggaggagacggtggcacgtctaaatccggtgaccggcggcggcatccacgtcgcggcgtgcctgcgtcgagcggcagaacaggcacgggacccggccgcaagctaggccgtgaccagtgcagaaagtgtgggcaaaccggccactgggctcgtgattgcaaggcaaggccaaagcaggaggcggcccacatggcccaggaggaggaggcgctcatgctgttgagggccgcaccactgcgtcgatccccgccagtagggtccgcgcctccaccgccaccggcagggtccgcgcctccaccgccgccgggaggatccgcgcctccacctctgcccccaccggccggatccgcgcctcctccactgcccccactggccggatccgcgcctccaccaccgccggcgggatccgccactgcgccgccaccggtgtctccagccccgtcgcgcacctcatccccatcgagcccctactctccacctccgcatgatgaggtcctcggcgcgcccagtgcagtggcgagctggtcatcgcaggaggccacggcggcgagcactgatgggcggatccagctacaggagggaaaggtgttcgtgcagctcggcgcgaaggagcagcgggaccctaggtcctggatctgcgacaccggggccacgaaccacatgacggggtctcgggcggcgttcgcagatctggacacggcagtgtctgggtcggtgcggttcggcgatgactccatggcggagattgaagggcgcgggaccgtgctgttcaagtgcaagaatggcgagcatcgctccttcaccggcgtttactacatcccgcgactcacggcgaatatcgtgagtctcggccagttggaggaggccgactacgacgtccacctctggcgcggtggcatggagattcgggagcctgaggggcgactgctggcgaggatcccacgcgcaggcaaccggctgtatgtgctcaacgtcgacgtggcacgaccggtgtgcctggcagcgcgcggcgaagaaagtgcctggcggtggcatgccaggctcggccacatcaacatgccggcgctgaggaagatggcgcgggaggagctcgttcgtggactgccttccatcgagcaggtggaccaactctgtgaggcgtgtctcgccggcaagcagcgacggacggcgttccctgaccagacacagtggcgggcggaccgcgcactggagctggtgcacggggacttatgtggacccgtgacaccggcgacaccaagcgggaacacctacttcctgcttctcgtcgacgatcggagtcgctacatgtggctgacgctgctgcggtccaaggatcgagcggcggctgccatcaaggagttccaggcacgtgcagaagctgagtccagctgcaagctgctggcgctccggacggatcgtggtggcgagttcacatccaaagagttcatggagtactgcgcggcggacggcgtgcaccggcagctgacagcgccgtattccccacaacagaacggcgtcgtcgagcgccggaacgccatggtggcaggcacagcacgctgtctgctcaaggcaaaggggctgccggcttggttctggggagaggcggtgaacactgccgtgtatctcctgaaccgggtaccgacgaaggcagtagaaggcaagactccattcgaggcatggtacgggaagaagccggcagtacaccacctgaagaccttcgggtgcattgtctatgttaagaatacgacaccgcacctgaagaagatggaggatcgtggccgcaagatgattttcgtcgggtacgagcgcgggtctaaggcatatcgagcctacgatccggccactaagcgcgtgcacgtgacgcgcgacgtggtctttgatgagtccgcccagtgggactggtcagagggagctaagaccggcactggcgcggatcatggcggcgacgacacgttcaccatccaatttcaggtggtggcggggccaggaggtgcccaggcgggggcaccggtggctgcattgcagcatatgacgccaccaggaacaccaggggaggccggagtagcttctccgggtgtgcagttcgccacaccacctgctgcgttagacgaagacctggacaccgaccacgacgacaacgcaccactgcggttccgaacagtggaggacgtcaccggagctgcgtcgccacctgggtacgccgtgcgcgtactgtacagcgagcaactctttgcagtgagtgcggaggagcctgggtcgcttgcacatgctgggcaagacccgtgctggcgacgagcgatggaggaggaactgcgcgccatagaggacaaccacacctggacactcactgagttgccatctgggcagcgagccattgggctcaagtgggtattcaaggtcaagaaagatgaacacggtgccgtggtacggcacaaggcacgacttgtcgcaaaaggctacgctcagcgccaaggaatcgactacgatgaggtgtttgcaccggtcgctcgcttggaagcggtgcggctgctgctggcgttggcagcgcaggagggatggcaagtccatcacatggacgtcaagacggcgttcttgaacggcgacctccaggaggaggtgtatgtgcagcaagcgcctggatttgctcaaccagggcaggaacacaaggtataccgactacacaaagctctttatggtttgcatcaagctccccgtgcctggaatcaaaaactggatgaaaaaatgggtgttctggggttcactagatgtccatctgagcatgctatttactgtcgaggagaaggtgcagagagactagttgtaggtgtctatgtagatgatctagtgatcacaggcactagcagcagcaacatccagactttcaaggctgaaatgaccaaggtatttaagatgagtgatcttggtttactatcatattacttgggcattgaagtgaagcaggatgagctgggaattaccctttctcaagagaggtatgctaataagatactagatctaagtggttttgcagactgcaactcgtgtgatataccaatgcaggcaaagctgaagctgagcaggaaggatgacagcccctgtgtagatgcaacagaatacaggagcttggtaggaaaattgaggtatctggtcaatacccgaccggatttggctttttcagttgggtatgtgagtagatttatggaggagccccacatggagcattttgcagctgtgaagcagattctcagatacatagctggaacaaggagttggggccttttctaccctaggaagaatgaaaagaaaactgaattgttgggcttcagtgacagtgaccttgccggtgatttggatgagaggaagagcacaactggagtccttttcttcctaggcaacagtccaattagctggcaatcaacaaagcaaaaggtagtggcattatcaagctgtgaggctgaatatatatcagctgctactgctgcttgtcaggctgtttggctagctcggctgctagctgaaattacagattcagttgtgagcaagccggtgttaagggtagacaacaagtctgccatttctttgataaaaaatccagtgcatcatgaccggtcaaaacatattgatacaagattccatttgatccgggagtatgcaaattcaggccaaattgaagtgaggttcatcaggacagaggatcagcttggagacatcctaacaaagcctctcggcaaactgaagttcagagaactctgctccaaaattggcattcagtctctgtaggtaaatcagtgcacaagtcttaggaggagatt

>Copia-54_ZM_LTR

tgtcaaataatacttgtgcctgtagttagtttgtcagttagcgtcgcattgttataggagctttctgttagccatgcacaaagggagagcctggtcgtgcgatcgtgtgcgcagtccgcacgaccggcgcgtcgcacgctgtgactgaggtgagtgggatggcactcaacgatgaggggcatggcgtgcaaatcagggtgatcgtgccccacatttctttttcctttctttaagcggtaaactgtcaggttgtaatctcatggtctgtacacgtctctttatatacagagaaatagagtcagaaaagcagttagttgttcacagcaccaaatcagagtgtgcctacttgtgttcgtgtgtgtgcgtgagtcttttctgtgaccagcagggtgagctcgccggtaactagaggttgcttctccggcgaggtctgaca

>Copia-55_ZM_IN

tggtatcagagcctctgacactggatgatgttcaggctcatctcctcgcctatgaagctcgccagatcaggcaccacacggcggctcgtcttcaagtcggcgcatctgctcatgtcgccggccgtggtggcccttttggtcgccggggccgtggcactggccgtggccgtgggcgcggtcgtgcccctccttcttctggccgaggtgttccttctactcgtcctcgcgttcgctgtcagatctgcgacaaagaaggccatagcgccattcggtgctggtaccggatggatgacgcatacaatgaagaaccaccctctgcagcagtagcggcgacaagctcctatcaacttgatccgagatggtataatgatactggtgctacggaccatattaccagtgatttggatcgtctcaccatgcgtgagaattataatggtggtgacacggtgcaagtcagcaacggacaaggtttgcaaatcttgcacactggttattcttctcttaataccgatagtcgccctcttgcactcaataatgttcttcatgtgccacaaatttctaaacatttgttatctgttcataaactaactcgtgataataacgttttctttgaatttcatccttggtattatcttattaaggatcgagaaacacgtcagctactccttgaaggacggtgtgagtccgggctgtatcccatcaagccttcggatattgcttctcttcatcaagcctttgtgtcggtgcggccagatcagtggcatgcacgttttggtcatccgtcatcgcaagtcgttaggtctattttgcgtcttcatcatttaccttattccaaagagtccagtttatcatcggtttgtaatgcctgtcagttaggcaaaagtcatcaacttccttacaatatttctattcatcatactactagacctcttgaaataatacattcagatgtttggggccccgctccactctcagtgggtggctacaagtattatattagctttattgatgatttcactaaattcacttggatttatcttatggttgatagaactgatgtgcaacgtatttttcttcaatttcaagcacatgttgagcgtctccttgattccaaaataaaatgcatacagtccgattggggtggggagtatcaaaaacttcataacaaattttttacctcacttggtattgatcatcgtgtttcttgtccccacactcatcaacaaaatggatctgccgaaagaaaacaccgtcatattgtagaaaccggcatcaccttgttagctcatgcgcacatgcccttaaagttttgggatgaagcgttcatgactgccacctttcttataaataggcttccttctcgtgttattgataatttgaccccccttgaacggctatttaaaactcctccaaattattccttggtccgagtttttggttgtgcttgctggcctcatcttcgaccgtataataagcacaaactagaatttcgttctaaaccttgtgtttttatcgggtatagttctcatcataaagggtacaagtgtcttgatctggaaactggtcgtgtctatatttcacgcgacgttatttttgatgaggttgtgttccccttctcaaaatcttcatccaactttggccagcatgagggtgctaccggtactaacgagaatactaatcagttgcttgatttgttgcctcctaagttttttcatgcagcaccgagtgccacagaccacccgtcacctgcgcccgacaattccttcccgggggacaacgcgtctgcgcccattactgctgcaccaaattctgcgccagggccctccacagtacctgccatctgtgatggcagcattttgcctccggttttctcgccagatttattctctccggcatctactgattctgatcaagtttatattataccgtctgctgaaattgcatcagctcctgctgaagatctggctgctgctcctacacactcatatggtactcgcctaaagaataacattcggcaacccaagcaacgaacagatggcacggtcacttattctgtttctcgggtctcttcatctgagccatcctcacatattgatgcaatgaaaaatcctctctggctcactgctatgaagactgagtttgatgctctcattttaaacaagacatggcgtcttattcctccacgagatgatcttaatatcattgattccaagtgggttttcaaattgaagcacaaagctgatgggtccattgatcgctacaaagctcgtcttgttgcaaagggttttaagcaacaatatggtgttgattatgatgataccttcagtccggtaatcaagcccactactattcgactattattatccttgtctgtcacaaataattggtcgcttcgtcaaatagatattcaaaatgcttttcttcatggcatacttgaagaagatgtctatatgaaacaacctccagggtttgaagattccagtcatcctgaatatatatgcaagcttgacaaggcattgtatggtcttaagcaggctccccgagcatggtttgctcgcttgactagcacactaattaaattgggttttcaagcctcaaaagctgatgtctctctatttattttcaaccaaggtgccactcagatttacatattggtctatgttgatgacattattatattgagctcctcttcacaagccactaaaaagcttcttgagcagctgtcacgtgtttttgctgtcaaagacttgggtgcacttaattattttcttggcattgaggttcatcatatttcatctggacttctgttaactcaacgcaaatatatacaagatctcttaacacgtaccaatatggagaactccaaggctgctcctacaccgatgttaccagctgagaagctctctctacatgatggcacaaaactttctcctgaagatacaactcgatacaggagtgttgttggcacacttcagtacttatcttttacaagaccggacatctccttctctgtaaaccgggtttgtcaatttttgtcggcgcccacaacatcacattgggctgccgtcaaacggattcttcgttacctaaacggtacacttgattatggtctggctatcaccaagacacacactccgttgcttcgggccttctctgatgctgactgggccggcaatccagatgatcgccggagtacaagaggtttcactatcttttttggtaacaatctcatatcttggggatctcgcaaacattcaactgtgtctcgatccagtactgaagcggaatacaaggaagtcgctaatgcaacggcagaaattatttggcttcaagtattacttcgggaacttggtgttgttcttcctcgaccaccagttctatggtgtgacaataccggtgctacatatcttactgccaacccaatatttcatcaaagaatgaagcatgtcgaagtcgactaccatttcgtccgagaacgtgttgcttccggccaattggatgttcgactaatctcctctaaggaccagcttgctgatataatgactaaggctctaccaggacctgcttttcgccatatttgtagcaatctgaacttgatttcatcccgtacagattgaggggggg

>Copia-55_ZM_LTR

tgttaggatagagaggaaatctcgctatagtagttattattgtttccatccttgggctgcgtcccatgatactcggcatgattattgccattcattgtaatcctctaattgctatataatgatatgcggtggccctccttgggtatcgccttccaccattctcaca

>Copia-56_ZM_IN

tttggtaccagagccgaggttcgtcgctagtttcgtcaatacgcctaagcaagctacagcatgccgccgcgacgaagatcaagcacgccgcgacgtcggcgatcgccgtccccaaccgacagccgcgcaggaggcagcggcgggcaagaccgagggctggtgattcaccgctcggtcagggagatcggtaactccgggtggcccattttgacgaagacgaactacaccgagtggtcggcgctgatgcgcgtcatgccccagggacgacatctctgggaagctattagcgccgtcaccggtgagttcatcgacgatcgcaatgccctcgagacattgtgcaaagtcgtgccccccgagctacaaggggttctcgccaacaaggccacagccaaggaggcttgggatgcgctgaagacccgtcaccttggtgtcgatcgggttcgcaaggcaaaggcgcagacactgcgacaggagttcgacatgatcgccttcagggaaggagatttcgttgatgacttctcatgccggctcaccaagatcaccgaccagctcgcaatccttggtgaagtttatgaagaagaaaccatcgtccgtaagtttcttcaagctcttcccgagcgtttccatcaaatagcagtggcgatcaagacccttctcgatctcgaggaggtgtccctggatgaattggtggggcgactcaaagcaacggaggagcgtatggattgcgcgaagtcaaagggaggtgtcggaccgagcgctggaggcaaagaaatcaacgacaagatgtacttcaccgaagaacaggtgatagctcgccttgcgtcccgtctcaacctcaacaccgatgggtcggggacgcgcggcagagcgcaagccgaccctggcggacgtcgtggcgggagaggccgcggcagggggcgcggcaagaacacgcgctcagctaagggtggcggcgaggtcgacgacgacgcctgccgctactgtggcaagttcgggcattgggcccgggagtgccgcaagaaaaagcgtgacgaggcgctggcccaggcgagccagcctcaggccaacctcactcaggtggaggaggagacgccctcgctgttcatggcggttgtgcaaccgtctgtgaagactgttcacgacacacaccgcgtcgagcatgtctacctcaacgagcataaggtgctgccggacttcgacggcgaacagcaggagatgaagtggtacctcgacactggtgcgtcaaaccacatgaccggcaacgcagacatcttctccgactacacccgcgacgtcgtgggctccgtgcgtttcggcgatggatctctggtggagatcgctgggtggggatcgatcctgttcgagtcgaaggatggagggcacaggacggttcatgatgtctaccacatccctcgtctttggagtaccatcctcagtgtcgggcaactcgacgagaacggcagcaggatcgacatcgaagacggtgttcttcatttgtgggatcgccgcagccacgaactcctcgcaaaggtacatcgttcatcgaatcgcttgtacatacttccactcaagccgacgaaaccggtgtgcctcgcggcaagctacaacgacgacgagtggcggtggcacgcgcggttcggccacctcggcttccaagctctgcagaagatggggcgcgatggcatggtccgtggcttgccgtcgatcgagcacgtcgagcacgtctgcgacgcatgtttggcggggaagcaacgtcgagcacctttcccgcaagctgctacgtaccgcgccactaagccgctcgacctcctccacagcgatctgtgcggtccgatcactccggcgacacccgacggcaagcgctacataatgcttgttatcgacgacatgtctaggtacatgtgggcagtgctgctcgccaacaagtctgatgctgaggacgcgttcaggaagctgtgcgccggtgtcgagaacgaagcggggcggaagataaaggcattcaggaccgatcgagggggggagttcacctcgaattccttcctcgagttctgctccgagcgcggcatcaagcgtcatctcactgcgccgtatagcccgcagcaaaacggcgtggtggagcgccgtaatcagtccatactcgccatggcacggagcatgatgaaggcaaaggctgtccctgccaggctatgggctgaggccgtgatgacggccgtattcctcctcaaccgcgcctcaacgagaagcttggtggggcgcacgccgtatgaggcgtggcacagtgaaagaccagtcgtgcactttctgcgggttttcggctgcgtggctcacgtgaatatcaccaaaccgaacgccgggaaacttgatgatcgaagcacgatgcacgaagatggtgatgattgggtacgaggctggttcgaaggcctacagggtgtacgaccccgtcgctaaccgggtgcatgtcacccgcgacgtggtgtttgaagaaggcgcaacctggggttggaactcgtccgacaaccgcgaccaggaaggctcctgcaacgatggagaccaggacacgttcgtggttgaagagtgggaggagaccactgctgcttcgccaacaagccatgcctcccctctttacacgcgggacggaggcgcttcttcatcaccgtcgggttctactttcctggttccgaccccagtgccaagatcacagggctcttcatcggcatcgcagtcggtcgagacatcctcgactagctccacgtcgacatcttcaaccacctcatgcccgcgcactcagcacgtcaaaggagggcctgagccgtggcagggcccactgccaccgaggggtgtgatccccgtcgagttcgtgtcacccagctccggtgcgctgcaggcactggacgcagatgacaactctgaggacgcgcacagattccgggtcgtcgacaacctcttgtacgacgacgacgagcttgtagcagatggcgatctgctgctggcagttgatgaagaaccctctacgtacggtgaagcagcagagcgtcaataatggaggcgggcgatggctgaggagctcaagtccatcgctgacaacaagacgtggacacttactgatgtgcctcctggcaagaaacccattggcctgaagtgggtattcaagatcaagcgtgacgcggatggcaacattaccaggcacaaggcatgactcgtggcgaaaggatatgtgcagcgcgccggcattgatttcgacgaagtctttgcgccggtttcacgcctggagtcggtgcggttcatcctcgccatcgccgcacattatgggtggatggtgcaccacctcgacgtgaagtctgcgttcctgaatggggacttggtagaggaagtctacgtcgagcagccaccgggattcatcagccgaggcagagaaggcaaggtgtacaagctgcacaaggcactgtacgggctttgccaagcgcctagggcctggaatgccaagctcgacagctcattgctgtcgcttggattcaggtgcagcaaggaggagcacgctgtgtacgtgcgaggcaccagcgatgatttgctgatcgtcggagtctatgtggacgacctcgtcgtagtgggagcaaaccaggcagaagttgtcaagttcaaggacgagatggtgaggctcttcaacatgagcgatctcggaccactccactactacctgggcatagaggtcagccagagcgcaaggggcattacattgtgccagagtacctacgcacgcaagatcattgagaaggcagggcttacgggatgtaactcatgcaacactccgatggagccacgcctcaagctgagcaaggcgagctcaaatccaccagtggacaagactttgtaccgcagcatcgtcgggagtttgaggtacctagttcacacgcgtcctaacattgcgtttgcagttggctatgtaagtcggttcatggagaagcccacgactgagcactggagcgccgtcaaacatttgctgaggtacattgccggaacaaagacccaagggtgtgtttatcgccgcgggaaaggaacattggagttgatcggatacagtgatgccaatcacgccggagacggtgatgacaggaagagcacctctggcgcaatcttcttccttggccagagcccggtcagctggcagtcccagaagcagcgtgtggtggcgctatcgttatgtgaggcagaatacatcgctggcgccaccgcggcgtgccaaggagtatggctgtcacgcctgctttcagacttgctgaacacaaaggtcgtcgcgccaatcctctacatcgacaacaagtctgcgcttgccctcgccaagaacccagtacttcatgatcgaagcaagcacatcgacatccgcttccatttcattcgagactgtcagcaacggctcgttggagacggatttcgtccgcaccgaagatcagatagctaatatactcacaaagccacttccgcgcgagcgtcttcaggaactgcggatacgaagtggtgtcaccagtattgaaccagcacaacacatttagggggagat

>Copia-56_ZM_LTR

tgtagaagaataatttgtagcgctggtgtctagattttttagagtctaaagttagttgggttagtggtcagcacgttgtggccttctcgcgcggtcgttgcggaagtgcgttgagtagtctctgcttgcatgtagagtcaaatggtggaactagaaaactgggcatagtggccacgattttggctgtgggatggcacagccgcttagggagcatggatacatgcaaccagggactcagctccacctgtaaatgcgtcaaataaataggaatggaaatcagagaagcagacatagtacgctgcgccaaaagactcgtgtactcattgtcttcctcatttttcagttaatcgtattcgtagagaggaagagacagttcgggtgttctagcgacggattggtccttgcttagaagccaaca

>Copia-57_ZM_IN

attggtatcaaagccaaactatcctgtagcctaaacatctcttgctcatctccttcccgcgcctctccccacactcagtcggcagcaagagctccaactgttccttcctctcctgtttagacgagcaatttttcgtctgagacagcctcttccacacgcagcgacccctcactagcccaccatgtccctacgctcggtcacttcgagtgcgcggcgccagcaggaagccgaggtcgccgcgacacaagaacgagagcgagcagcagcagcggctgcagcgacagcggcgagggcagcacggctggcggcagcggaactggcagcagcgagagcggaagtagaagcagcggaggcggcggatgctgcacgtgcggcggcagcagagctcgaggttctgcgcggcagtagagctggcagctctgcttctgtcgacgacaacaccgatgaagagctcaggctggcgagggaagcagcgcgagagcaggctgcacagtgggcagccgtgcacccccatgggggcgcgcgtggcggcagcccagatagggccgatgcgctgagggcgctctaggcgagggcgcacgcggcggcagcccagacgggcgtagacgcgctggcggtgctcccggcggtggcgaccgggtcgacggagatcgcggcctctacaggcggcgcgactctccctccccggatcggtaccatggtcgccgcatggcccaggccattgtcagggacatcggtcccggcggtgggtggcctaccctcaccaagaccaactatgtcgagtgggccgcggtgatgaggctacggctccaggttcgccaaatgtgggaagcagttcggtacggcgacgtcgactaccacgaggatcggcgggcgttggatgccctcattgctgcagtcccgtccgagatgcagttttcgctttcccagaagcggactgccaaggaggcctgggacaccatcgctgcgacccgcatcggcagcgaccgtgcccgcaagaccacactgcaggcacttcgcaaggagtgggagaacctggccttcaagccaggtgaggatgttgatgactttgctctccgcctcaacactctgttgcagaagatggtgcagttcggcgacgacacctacgatgaggagagagctgttgagaagctcttccgttgcatcctcgagaagtacaagcagatcgctcgctcgatcgagtctctgctagacctctccacgatgacgatcgaagaggcgataggtcgtctcaaggtggtcgacggcgacgaaccacagactccctctggccctatcactattggcgggaagctacatctcactcgggagcagtgggaggcctgccagggtgaccagaagaagggggagtcctcctcgacacgaggccgcaactgcggcaagcttggccactgggccagggactgtcggcagccacgacgtggtcaggccaacgtcgcacaggcggaggcggaggaggaggccctgctcctggcacatgcaagcatcgagatatctccagcggcaccggccgcagcggcactcctccaccttgatgagtcgaaagcacgcgctttcctcggcgacggctccaacaaggacatgatcgaagggtggtgcctcgacaccagcgccactcatcacatgaccggccgacgggagttcttcaccgagcttgactctagcgtccgaggctccgtcaagtttggggacgcttccggcgtagagatcaagggcgccggctcagtcgtcttcaccgccgcatctggtgagcataggctgctcaccggagtctactacatccccgcgttgaggaactctatcatcagcttgggacagctggatgagaatggttcgcgcgtggaggtcgagcacggagtcatgaggatctgggacccctctcgtcgccttcttgccaaggtacgcaggagttcaaatcggctatacatcctcaatgtgaaggtggcacaaccttgctgccttgctgctcgtcgagacgacggggcatggcagtggcacgagcgcttcgggcaccttaacttcgaggccctgaagcggctcagtgccaaggagatggtacgaggcctgccgtgccttgaccatgtggagcaattctgcgatgtctgcgtgttgacaaagcagagacgactcccctttccctagcagtcgagcttccgagccaaggagaggctcgagctcgtgcatggggacttgtgtggcccggtgacaccagccacaccaggaggacgacgctacttcttgctgctcgtcgacgatctctcccgctacatgtgggtgatgatccttggcagcaagggagaggctgcgaacgccatcaggcgtgtgcaggtcgctgcggaggcggagtgcggccgcaagctgcgcgtgctgcgcaccgacaacggcggcgaattcacggcggctgagttcgcgtcgtactgcgcggatgagggcgttcagcgccactactccgcgccgtacagcccgcagcagaacggcgtcgtcgagcggcgcaaccagacggttgtggggatggctcgggctctcctcaagcagagaggaatgccagctgtcttctggggagaggcggtggtgacagcggtctacatcctcaaccgctcgcccaccaaggcactcaacgggatgacaccgtacgaggcttggcatgggcgcaagccggtggtctctcacctacgggtcttcggctgcctcgcgttcaccaaggagcttggccacatcggcaagctcgacgacaggagcaccccgggggtgttcattggctacgcggagggctcgaaggcctaccgcatccttgacccaggaacacagcgtgtgcgcacggcgcgcgacgtagtgttcgacgaagggcgaggatgggcgtgggacaaggcggtggacgacggcacgactccgacgtacgacgacttcaccatcgagtacgtccactttgagggagctgggggagtaggcaattcttctccgagcaggtctaccccagcccccaagtctccaccgactccagcgccacgctctccggctccggctacaacgagctcttcaccaccacgcactccagccacgactccggctacaccgagctcttcgccaccacgtactcctgcaccgacggtaccctctccgggaacgtcctctctgacaccagctcgtgtcgagcacgacccagtggagctcgtgaccccgctctcccgcgacgaggagcgcgttgacgcgtgctacgacggcgagccgttgcggtatcgaagggtggagggccttctcatcgacccgtcggtgccgggccctgcgtctcgcattctggcaggagagttgcatcttgcatgcgacgacggtgagcctcggtctttcgcggaggccgagaaacatgcggcttggcgtgccgcgatgcagtcggagatggacgcggttgagacgaaccgcacttgggagctcgctgatctccctcatggtcatcgcgcgatcacccttaagtgggtgttcaaactgaagagggatgaagccggcgccatcgtcaagcataaggctcgcttggtggcacgcggtttcttgcagcaggaggggatcgacttcgacgatgccttcacccctgtagcacggatggaatccgtgcgactcctcgcgctggcagccctggagggctggcatgttcatcacatggatgtcaagtcggcgtttcttaacgacgacttaaaggaggaggtctacgtacaccagccgccaggttttgcgatccctggcaaggagggcaaggtgttgcgcctgcgcaaggctctctatggcctgcgacaggcaccgagggcgtggaatgccaagctggattccacgctcaaaggaatgggtttcacgccaagcccgcacgaggcggccatctatcggcggggcaatggaggaagtgccctgctggtgggtgtctacgtcgacgacttggtgatcaccggcgccaaggatgcagaggtggcaacgttcaaggaagagatgaaggccaccttccaaatgagtgacctggggcatctctccttctacctggggattgaggtgcaccagggagactccgggatcacacttcgccagaccgcctatgccaagcgcattgttgagctggctgggctcaccgactgcaacccagctctcactccgatggaggagaggctgaagctgagtcgcgacagcacgacggaggaggtggatgctacacagtaccagcgtcttgtggggagccttcgctacctcgtccacacacgacctgacttggcatactccgtcggctacgttagtcggttcttgcagcgaccgacgacggagcatgagcaggctgtgaagaggatcatccgctatgttgcggggactctcgaccacggtctctactacccgaggtgccctggggaggcacaccttgtcgggtacagcgacagcgaccacgccggtgacatcgacactagcaagagcacaagcgggatcctcttcttcctcggcaagtgcctcattagctggcagtcggtcaagcagtaggtggtggccatgtccagctgcgaggccgagtacatagcggcgtccactgcttcgactcaggcgctctggctggctcgactgctcggtgatctcctcgggagagacactggagcggtggaactcagggtggacagccagtccgctctggcattggccaagaaccccgtgttccatgaacggagcaagcacatccgactaagataccacttcatccgagactgcttggcagaagggagcatcaaggcgcgctacatcaacaccaaggatcagcttgcagacctgctcaccaagccccttgggaagatcaagtttgttgagctttgctccaggtccgggatgacccaactttcccacaagacgacgcacaagacttagggggagaatgatggaataagtcatgtggtcagcagcagttgcagttttgactgctgcaggacagcagcagttgcagcaggaacagtagcagcagtcttttgactgtgcagtcttttgactgccagttttgactgcactttagtttctaaaggacagcaccagcccctagggctataaata

>Copia-57_ZM_LTR

tgtgtccccaacccctctaagggtatgacattgtgtagtgtttgaggaaataaacagaaaattgccccaactcatagtgtcatcctcttgatgagagttagagtccctctacttaca

>Copia-58_ZM_IN

ccccccgcagtaggaacgtcggctggccgaacattcatactggagcgaaactcaataaagactgtagagggaaggcccttggtgaaaatatcggtgaactgggaggatgtcgggacatgtagaacacgaactgcaccggcggccacacgctcacgaacaaagtgaaggtcaatctcgatatgtttggtgcgttgatgttgcactggattagtcgagagatagacggcactcacattgtcacagtaaaccagggtagcctgctgcaagggttgtcccagttcctgaaggagttgccggagccaggaagcttctgccacaccattagcaatggcacggtattcagcctcagcactggaacgtgacacggtatgctgtcgtttgctggaccaagaaacaagactggtgccgagaaaggcagcaaaaccggaggtggagcgacgagtctcaggacaaccagcccagtcggcgtcggtgtagatggtgagtgtggtcggggaggaggcgcggtcgatgaatagcccgagatcaggtgtcccctggagatagcgaaggatgcgcttcgcggcagcaagatgtggttcccgaggatcatgcatgtggaggcacacctgctgaacagcgtaggtgatgtcgggacgagtgaaagtcaagtactgaagggcgccaaccagactgcggtagagggtagggtcggacacgggtgcgccagaagaagacaacttggattgggtatctgctggggtggcgcaggtcttgcagccagtcatgccggcgcgctccaatatctcaagcgtgtactggcgctgagagagcaacatgccggtggcggaacgagtcacggtaatccccagaaaatggtgaagtggaccaaggtctttcatcgggaaggactgctgtagtgaactgatgatctggcgaagcagagctgaggtagaggccgtcaggatgatgtcatcaacgtagagaagcaaataggcactagcagtaccatgccggtagatgaagagcgaagtatctgtcttggcctcaatgaaccccagagaccggatgtgagacgcaaaacaactgtaccatgctcgaggagcctgcttcaatccgtagagtgatttgttgaggcgacatacgtgatctggatggtgagggtcaatgaaaccaactgatgaatcggccagtcctgatgaagcgccaagctgaggacggtgcggatggtggcgggcttcacaacgggactaaaagtctcgtcgaaatcaacgccagggcgctgagtaaatccacgaaggacccaacgcgctttgtagcgctcaagggaaccatcagagtgaaacttgtgtttgaatatccacttgcctgagacgacgttggcatgagacgggcgtggaacgagagtccaagtgttgttggtgaggagggcttggaactcgtcctccatcgcacgccgccagagggggtcgtcgagggcgcgacggtacgttgatggtataggggaggcagcaggagtcgtagcttgcaggttgaggcgcggttggtgaaaaccgcttttcgcgcgagtgcgcatgccgtggctgttgaccacgggaagagctggaacggagcccacaggaacaggcgatgtcgtagcagccggatgacgagcagatcggtgcctgtcggtagtaccccaacgccagggaagattgtcgctggcgcccgcaggatgcagctgggcggccgcgcgcgtcccaggtgaaagcggtagcccaggcacgatgggcccagacgacagccccggggaagttggcggcccaggcgcgggaggcccaggtggaggccgcggctggtcgttgagggggccaggcgaggcaggtgtccgggcggccggatgggcagcctgcggaagcggcctaggtggaggctgcggctgtccgtcgagtgggccagccctggcggccggttgggcagcctgctggagcggtccaggtggtatggcgctggtgcgctgcccagcagtggacgagggcaatgcgccaatgggtggaaccgggacctgttcagaaaataaatccaaaaaatcaaggttagtgggaagagccgagctgggaggggtctcggcaaacggaaaggaggattcgtcgaacacaacatggcgggagatgatgatacgattggtggagatatcaagacaacggtagcctttgtgttcagacgagtaaccaagaaagacacacatggtggagcgtggagcgagtttatgggcggcagtggcagagatgttgggatagcacttacagccaaagacacggagatggtcgtaagatggggggcgaccaaagagggcaagatggggagtggaggaatttagggtgctggtggggtggagattgagaagatatgtggcggtgtgaagagcctcaacccaataaggagcaggaaggtgagcttgaaagagtagggaacggacgatgttgttaacagtgcggaggacacgttctgctctaccattctgctgggaggtatagggacacgacatacgaaggtggacaccgtgggagaggaagaaagctcgtgtgtgggagttgtcaaactcacgaccattatcacattgcaagctttttatggtggtgccaaattgagtggcaacaaaagagaaaaaattagcaatagtgggaaatgtgtcggatttgaggcggagagggaatgtccacaggaagtgggagcagtcgtcaaggatggcgaggtagtatttgtaaccagaaatactgggtattggagaagtccatagatcacagtgaacgagatcaaagtgacgtggagcacgagactgggaaatggaaaaaggaaggcgaacatgacgaccaagttgacatgcgtgacagatatcgggggcagtagagcctttattacatgaaatagaggatgaactagttaaatgacgaagggcgtcgtggctgatgtgaccaagacggtgatgccaaagctgtgaagaggcgctggtgaggaagacttggggggtagtggtggaggacaacgggatggagagtggatagaggtcgcctgagctattgcacctggcgatcacgctccgtgtcctgagatccttcacggaaaaaccagcggggtcaaattcaacagagcaattattatcaattgtgaattgacgaacagagacaagatttttgataatagatggtgagacaagcacattattaaggtggatcggacccatatgtgtggatcccgtggagacaacgggcaaggaggtcccatcgccgacaataattgaagaaggagtggaagaggaagggggatggcaataagttaggataccggaggtggaagccatatgagcggtggcgccggagtccatgtaccattcaccggttggcggggtgagtgtcatggtgttgaaagcacctgctaaagcatgttggtcccatgggccaaaaggaccaggagccgcatgctgctgggtcggcgaggcctgctggacggcccagggtgcagccggaacgggagcaggccaggtggacgcgtttgccgtgggccaggtgaatggggcccagccggtgggagtgggcgggggaggcccaagtgcgctcggcggcccacccacgagcgcctggggggaggcagggtgccgatgcccctggactgggccgggccacatctgaatgctgccggtccaggggttgaagatgttgggccagctgccctgaggcgtggcctgaccacgggcgccgccaggggcaccgcgagcgcctccagggttggaaggaccggatccgccgctgtgcttgcctttccggcggcggcggttgtagccgccatttgcttggggaggaggaggtcccgcggcgagggcagtagcggtgccgggcgcggaggttggtgcagaggtgacgagtgcggatggagcagcagaggcggcgcccggtcccatggagagctcttccagaaggagctcggagcgggcctcgacgaacgacgggaaggggcgctggcgcttcaggtaggagcgcagatgagcgaagcggtcgttgaggccgcgcagaaggttgatggtgagcagccggtcggagatgggctcaccgagctcgaccagggagtcggcaagggtcttcatccgccggcagtagtcggagacggagagggcgccttggcacagggtgcggaactcggtgtcgacgaggacgatgcgggtctcccgattgcccacaaactgctcctcgagagccaaccacgtcgtacgcgcagtgggggcggcggtggtgacaatctcgtagagctcgggcgagatggagccgtagagccaggagagtacatggagttccatccggacccagggcgcgacgtcggggaaagaggcatcgcacagcacgtggtcggcaagggcgtacttgccgaggacgaggaggaagaggcggcgccacttggagaaggatggcgtcgcaaggtcgaggacgacggggacgaggccgcggatgttgaggatgccgagagcctgggcatggagcgcagcgacgggatcggggtcggaaccgtcgcgacgcacgtccctacccggcgcgtcggcggctgggacttcgtcgtgctcgatctcgccgtcgtcggctgcgcaggcggtctcaaggcgttggcgagccgcggcgagggcctgctcgtggcgggcgacgtcggcagcaagacggcgacgctcggcagcggcagcagccgcggcgtctgcgcgggcttggagagcggcggctgtctcagcagatcgacgctgggcgccgtcgttagcctcctgctggagtcggcggtcctcggcggcacgaagatcgtcggcagaacgacgggcgtcggcggcgcgacgagcgacgatgtcggcgtcgctgggagcgtcggcggccatgactggaagagggaagcggagggtgggctaggcgaggaacgtagccttctttgatacca

>Copia-58_ZM_LTR

tgaagacgtgagagtttggttgagtacacggcttgattaagatcagcagtacaggttacaatatatagccacagatatacccagcttatagaaatagtatctgggtaatcgggcaagcaaccacaggattgatatcctgggatgggtttccataacagctgatagcctactaagagtgactgtctaaca

>Copia-59_ZM_IN

agtggtatcagagctttggttgtttggtgatcggtgtgctagggtttcctgccggcgcacggatgtcgacgacaatgaagtacgatcttccgctgctggatctcgacacgcgtttctcgctgtggcaggtgaagatgcgggcaattctctcgcaatctgatcgcgacctggatgatgccctagatggatttggcaacaaggatgccaggacgtggactgatgaggagaggcgcaaggatcgtaaggcgctcgcacatattcaccttcatctgtctaacaatattctgcaggaggttctggcagagaaaactgccgctgctctctggttgaagctggagtcgatctgtatgtcaaaggatctgaccagcaagatgcatgtcaaaatgaagttgttctcccacaagttgcaggaaggaggatccgttctgacgcacatatcggtgtttaaggagatcgtggccgatctgacgtctatggaggtaaagtttgatgatgaagacctagctcttcttctcctgtgttccctccctgcatcgtttagcaattttcgagataccatcctatacagtcgtgatactctaaccgtggctgaagtgtatgaagctttgactgcgaaagaaaagatgagacagatggttaactcggaagatgctgctggctcgagtggagaagctttgtatgtccgtggccgcactgatcagaaaaagtccaactcaggtggcaaaggaaaggggaagaaccagaggggtcgctccaagtcaagggggccatccgatgaattattttgcaaatactgcaagaagacgaaccatgtaattgagaactgttacaaactgcagaacaaggagaagaggaacaaggagaagggtaaaactggaggtactgtctctgttgcatccgaaaataattctgataatggtgatgttcttattgcctttgctggttgtgctgctgatgatgcccaatggatcctcgattctgcttgctcatatcatggttgcactaaaaaatctctgtttagtacctatgaagctgtgcagaacggaggtactcttcggatgggtgataattccccttgcactgttgttggcatgggcaccgtgcagatcaagatgttcgatgggattgtacgcacattgactgaagtacgacatgttccatccatgtccaggaacctcatctctctgagtactctggacacaaagggctacaagtataccgccggtgacggtgtcatgaaggtaacaaaaggctctcttgtggttattaaaggtgatttgaaagctgaaaacctatatgtgcttcgaggtagttctggctctgctaatgctgttgttgcatctgattctgagactactaaaatttggcatatgcgccttggccatatgagtgcacctggtttggcagaattgagcaagagaggccttcttaatggttgccatgctgatactcttgatttctgtgagcactgtgttttcggtaagcataaaagggtaaaattcagttctgttgttcataataccgaaaatattcttgattatgttcatgctgatttgtgggggccgtcacgtataccttcacatggtggtgctcgttacatgttgactatcattgatgataattcacgcagagtttggccttattttcttaagcaaaaatcagatgcctttgagtctttcaaggtttggaagactatggttgagaagcagactgagaggaagctgaaggttctgagaactgacaatggtatggagttttgttctggtgattttaactctttctgcaggaaggagggcattgtgaggcaccacactataccatatacgcctcagcagaacggtgtggcagagcgtatgaatagaacaataatctccaaggctcggtgtatgctgtccaactcaggtttgagccgcaagttttgggctgaggctgcttccactgcttgtcatttgatcaattgttcaccatccactgctatcgataagaaaactccaattgaggtttggtctggttcccctatgattattctcagttgagagtgtttgattgtactgcatatgcacatgttgataatggtaaacttgagcctagagcaattaagtgtgttttcttgggttatggatctggtgttaaagcatataaattgtggaatcctgatacacagaaggcattttttagtagaaacgttgtgttcaatgaatctgctatgtttccttcggttgtatctaccagtgctactaaccagaattctgagtctatcagtgtgcaggtggagcacgggggggatgttgatgatcatgctccaccatctgctgaacctgctgaaaattctgtttctgaaatttcatcacctgttgtggagtctcatccccagtctcttgctgaaggccgaactaggaggcagatagttcggccccaaagactgattcaagagtgcaatgttgcttttgctttagctgttgctgaagaagttgataatgttcaggaaccattgaattattcagaagctatattgtgtactgatagtgaaaagtggatgggtgctatgcatgaagaaatggagtctcttgataagaatggcacttgggaactagctcggctgccttctggtaaaaaggcaattaagtgcaaatggatcttcaaaagaaaagaaggtatgactccaaaagagcctccgcgctttaaagcaaggctagttgcaaaaggttttagtcagattccaggaattgattactctgatgtctattctcctgttgtcaagcatagttcaattcgtacctttcttagtcttgttgccgtacatgactatgagcttgaacagttagatgtgaaaactgctttcttacatggtgatttggaggaggacatttatatggaccagccagaggggttcattgttcctggaaaagaagactatgtgtgcaggttgaagaagtccttatatggtctgaaacaatctcccagacaatggtataagaggtttgattcatttatgctttctaagggctttcagaggtctcaatatgatagttgtgtttatctaaaatttgttaatggatcacctacatatttgatgctatatgttgatgatatgttgattgctgccaagagcatgaaggaaatcactgctttaaaggcacagcttagtagcgaatttgatatgaaggatctgggtgctgcaaagaaaaatccttggcatagagatagtcagggataggaagtctggactgctgtacttgagccagaagaattatattgagaaggtccttcgtcgtttcaacatgcaaaatactaaacctgtgagtactccgttggctcctcattttaaactgtctgctaaacagtgtgctgaaactgatgctgagcttgaatatatgtcaaaagttccatactctagtgctgttgggtcactcatgtatgctatggtttgttctcgtcctgatttgtctcatgctatgagcgttgttgctagatacatgtctaatcctggtaaagagcattggaaagctgttcagtggattttcagatatctacgtggttcttctagtgcttgtttatgttttggtaaatctggagatggtctgattggctatgttgattcagattatggtggtgatttagacaggaggagatctctttcaggttatgtctttactattggagattgtgctgtgagttggaaagctcgtttacaggatactgttgctttgtctaccacagaagctgagtatatggcaattgcagaagttactaaggaagccttatggttgaaaggtatatattcagagctatgtggaattaagtcttgcattatcatctattgtgatagccagagtgccattcatctcaccaaagatcagatgattactgagaaatccaaacatattgatattcgttatcacttcattcgtgatatcattgataaagggttggtgaaggtatgcaagatcagtactgataataatccagctgatatgatgacgaagcatgtttctgttgctaagtttgagctatagttggtattataaattagcccttagtggctgttggcgccggcaagatgatgacacatttgtttaaggtggaggttgctattatgatgctacaaggaatttgtctcaaggtggagtt

>Copia-59_ZM_LTR

tgttgagtttgtgatccaaattctgaagaaggcggccaagttggcgagcgaagcggaggcccgtcgccgggaggcttgggccggagcgcagcgcgcggcccagggacgcctggggggtaaggggggcggagcccccccgcggtacggatcgttttagggtttcacctctataaatatctttgtaagccgcaggagatatctatctctattgtaaatctcctgcgatctgtaaccacccaaaaatagtgagaagttgccggccggcgcccgtggttttttccccttcactttggaggggttttccacgttaaatccgtgtcttctctgtgattgatcctatttgtgtcgcattcatttataaca

>Copia-60_ZM_IN

gtgcctctaatattctttatggtaatcagagccaatccataaacaccaaaatccaccagagaatcatcatgtcgagcactgctgcaattatcaatcccctcgtcggtcaagctgtttctgagaaactggccaaggggaactatcaactctggaagatgcaagtcctagccgtcgtcaggggtgtgcgcctggagggttacctcacgggtgctgctgcagctccggcgaccaccatcaaggacaaggacgtcgacgttcccaatccggcatgggaggactggaagaccacagaccaacaggtcctaggattcttgctctcatccatgaccaaggaagccctcacccaggtagcggcatgcaaaactgccacagaaacctggaaggtcatcgaaacaatttactcctccatgaccaaggcacgctccatcaacacaagaatcgccctcgccaccaccaagaaaggggacctcacaattgctgaatatgtcggcaagatgagggccctgggagatgagatggcaaactccggcaagccgatcgacgacgacgagctcatctcgtacatcatcaccggcctcgactacgactacaatccggtcatcacctccctcgtcgcgcgagctgatccgctcaccattcgggaggtgtactcccaactgctcagttatgagcaacggctggatctcctacgcgagtcagactcctactccgtcaacgctgccacccgtggaagaggaagttcccgaggaagaggcggcaaccgtggtggaggaagtagagattctagaggccgaggacgtggcagatctcagtctcgctagattggcatcggcaacagaaacgctccacaacaagacagcaggcccaagtgccagctctgtggcaaaagaggacatctggtaatgaagtgttggcatcgctttgacgaaaattttgttcccaatgaaaaatatgcaggggcagcagccacatcatatggagcagactcaaactggtatgtagatactggagccacagatcacatcaccggagatcttgagaggctagcagtacatgaccaatacagaggccatgatcagatccacacggcaagtggtgcaggtatgaacattagtcgcattggttattcaactgttccaactcataaccgtgatcttaaactcaataacattctttatgttcctcaagttacaaagaatcttgtctcagttcataaactagccaccgataactcagcctttcttgaatttcatcccaatttctttgtgatcaaggatcaggccacgaagagacctctgcttaaaggacgctgccacaaagggctctatcctttgccattgacatcatccaagcaagcatatggagccactagaccatctattgaaaaatggcatagtcgtctaggtcatccctctgttcctatagtagaaaaagttattcgtcgttttaatctcccttgttctagtgagaagaataaacagtctgtttgcgacgcctgtcaaaaggctaagagtcatcagttaccttatcccaagtcaagtcatgtgtcgagtcatcccttagaactcgttttctctgatgtttggggtcatgcacccaactctataggaggcaataaatattatgtcagttttatcgatgattatagtaaatttacttggatttatcctctcaaattcaaatccgaagtctttcaaaagtttgttgaatttcagactctcgttgagcgtcttttcgatcgcaaaataataaccatgcaaaccgattggggcggagaatatcaaaaacttcattccttctttgctaaagttggaattagccatcatatctcttgtccttatgcacatcaacagaatgggtcggcggagagaaaacaccgccatattgttgaagttggtctagcccttcttgctcatgcttctatgcccctgaaatactgggatgacgccttccttgcagctgtttatttgatcaaccgtactcctagcaaaaccataaattatgaaacacctttggagcgtctttttcaccaaaaaccaaattactcctttttgcgcattttttggttgtgcttgctggcctaaccttcgtccctataattctcataagcttcaattccgctcccgtcaatgtgtctttctaggatacagtaattttcacaaaggtttcaaatgtcttgatctttcttcaggacgagtctatatttcaagggatgtcatctttgatgaaacaatttttcccttctcctctctccgccccaatgctggtcgtcgtctccaacaagaaatttcccttcttcctccatcccttcaacattctgatattatcaccggttatgggggccaacatgtggataatcatgtgcctacatgttctactaacactactaatccatgtgctttgagctctccacctgtgcaggacacagccgggtcaaatgatgcttccggcccaagtactgaggccgagtcacctatgccagctcctgttgacgatgctgccctgcccgccatgcagttggatgagagcgccatacacgatccgcctgcagccacggacgccccgcctcttggatcctctgcacccgggggggaccaacagcagtcgcagcatatggaacagcggcgcccgagtacacgtctccaacatggaatccgtaagcccaaagtttatactgatggcactatcaagtacaattttcttacaacttctggtgaaccacgaaacttagaagacgccttgagtgataaaaactggaaaaatgctatggatgctgaatttatggcactagaaaagaatagaacctggcacttagttccacctcaaaaaggcaggaatataattgactgtaaatgggtttataaaattaaaagaaaacaagatggaagcttagatagatataaagctcgcctggttgcaaaaggatttaaacaaaggtatggtattgattatgaggacacttttagccctgtggtcaagccaactactatcaggattgttctctctattgctatctctagggggtggtctcttcgtcaattggacgtgcaaaatgcttttcttcatggaatccttgaagaggaagtgtacatgaaacaacctcctggatatgaggacaaagagaagccaagttatgtttgcaaactagacaaagctctttatggtctaaaacaagctcctcgggcctggtatgcaaggttgagtaagaaacttttttatcttggttttagaagttccaaggccgacacttccctatttttctatagtagcaaagatgtgtctatgtttatccttatatatgtagatgacataattgtggcaagctcaaagcaagaagctgtttcagcactactgaaaaatctgcaggaagagtttgctctaaaggacttagggaatcttcactacttccttggtatagaggtaaacaagacacaaaacggtatactgttaacacagggaaaatatgcaactgatttactacggaggacaaacatggcgaattgtaaacctgagagtactccgttatccacaagtgaaaaattacatcttcatgaaggttctctgcttgggccaaatgatgctactcaatatcgaagtgtagttggggccctgcagtatttgaccttaacaagaccagatattgcatttccagtaaacaaagtgtgccaatttctacatgctccaaccacatctcactggacagcagtaaaaagaattttgagatatgttaaatctagtattagtcttgggctaagcttgtgcaaatcttcctcctttctagtgagcggattttcagatgcagactgggcaggatgcctcgatgacaggagatccaccggaggttttgctatctttcttggaaataatttagtgtcatggaatgcaaagaagcaagctacagtatctcgatccagcactgaagcagaatataaagctctagctaatgccacagctgagatcatgtggattcaaactctcttgaatgaactgaaagtttcaagcccctcagtcgcaaaactttgggtagacaatatgggtgccaagtatttatcatttaatcctgtctttcatgccagaacaaaacacattgaggtggattatcattttgtcagggagcgtgtcgccaggaaattactggacattgactatgttcccacaggagatcaaattgcagatggttttaccaaagctcttacagtgaagaaattagaaaactttaaatacaatctcaacttgagaaagttgtgattgagggggcgtgtaaagcaaagcgcc

>Copia-60_ZM_LTR

tgtaacagataacgcagagttagtatatcaatcttatctcatatagtcggttgtattagagtttatcttgtgtaaccaactctgcgtgattatctgaaacagactcatgattatccaaatgtaaaccacgtcaatggttgttattgcccatatataaacatgcagcacaccggccaggggcatcacggtgttttccaatctcttcca

>Copia-61_ZM_IN

tggtaatcggagcacctcttccaaatacatctagtttttcttgctagttcatatggcgtcatcgagcagcttctcatctcttggtcatccgatcacggagaaattgacaagagacaacttcatgttgtggaaagcgcagttcatcccggctgtgcgaggggcacaactgtttggatttcttgatggctctgtgaaggtgccagaacaagaaatcgagaactctgagaacaagaaggaagaaaatccagcgtacgcacgttgggttgttcaggatcaacagcttttaagcttcctgaactcgtctttgtcaagggaagtccttggccaagttgcagatcaataaacttctgctggtgtttggaaaaagttgatggagatgtactcttcgcaatccagagcaagagtattacatctcagatccaagttgtcatctacccgcaagggtgagatgacatgtgttgtatactttgctttaatgaaaggctatgctgatgagatggctgcggcaggtaaaaaaattgatttttttattttgtgtcatatatcatatctggtcttgatgcagattataatcctatggtggaagccatatgtgtcaaagctgaaccaacatctcttagcgatctttacgctaatatgctcagtaccgagtcccgcctagaagcacaaaatactccatcctacgtgactgcaaatttctctaatcgtggtgctcgtagtggtgggcgctctggacgtgggcgcgagagaggccgtggccggggaggccgcaacaacttctctcaggttcagtgccaattatgtggtcgcaatggccatgtggtgcaaaaatgttggaaaagatttgataaaaacttcaccggtgaagagaagattgtcaatgctgcagccaactacaactcatatggcatagacacgaactggtatacagacaccggtgccactcaccatattactggtgagctcgataaactcactacacgagagaaatatcaaggtcaggagcaagtccatgtcaccaatggtgcaggtatgagcataagtcatattggtcatacaagttttcgtactcctcttcgtactgttcatctcaacaatatcctctatagttctcaagccaaaaagaatttaatttctgttcatcgcctcacaaaagaaaataatgcctatgttgaatttcatcctaactattttcttgtaaaggacaaagcaacggtgaaaactctactccaaggcagatgtgaaggaggcctctatcctctccctcgtaacatccgaagcttgtcttcttctaaacaagcttgtgctaccctcaaaccaactatctcccggtgacatgatagattaggccatccttcttcggttattgttcaaaaaatagttcgagagtcagatttgtcatgtggtatttctcaagatgagtcagtatgtgacgcttgtcaacaggcgaaaagtcatcagttgccataccctaagtctagtagtgtgtcaaaagttcccttagaacttattttttcagatgtgtggggtccagccaaacaatctattggcagaaataaatattatgtcagttttattgatgattatagtaaatttatttggatttatccaattaaattcaaatctgatgtttttcaatggttccttgagtttcaatcatttgttgagcgtcaatttgatagaaaaatccttgctattcagacagattggggggggggtgagtatcaaaaacttagtcccttcttcacacgcataggtattgcccatcatgtttcatgtccgcatgcgcaccaacaaaatggatcggctgaacgtaagcatcgacatatcgttgaagtcggcctttctctactagcccgtgcatctatgccccttaaattctgggacgaagcgtttgctacagctgcctacttaatcaatcgcttaccatctcgtgtcataaattatgatactccccttcatcggttgtttaattcttctccagattattctatgcttcgcacgtttggttgtgcatgctggccccatcttagaccgtataacacacacaagctccaattccgatccaaaaagtgtgtgtttcttggctatagtcccttacataaaggatataagtgccttgatactagtaccggtcgtatctatatatctcgtgacgtcgtgttcgacgatacttgtttcccgttatctgccttgcctcctacaaaaatactaattcccaaatccatagtgagcaaaaccttcttgaccttgatccaactttgtaccctactggagacgaaacacttgtgtctgataatgtgattaatgtgtctaaccctgttggacacaatggaactgcaccggtgcattttatttcaggtactacagctgtggaacctggctccactcggacattttccttaggacatgcttcggaggctgcgacacctgtaatgtctctcgtgccttctatgggtgatgtggcgcccttatctcatctgccagcaatcaacgtgcctacgttttctccactgatagcaacagatgtggccaatgtaccgtctcctgttcctgcttctgatgatgtggcgccattgtgtcaaccagtagcaccatcttcaggttcggcgctccctctgtcgtcacctcatcatgatgtgtctgaagcctctacacaattggaatcagtagcgcctgcagatcaagagcagggtgatctgacctttgctaatcctcgaccggttaccagacttcagcggggaattgtaaagtcaaaatagtatactgatggcacaattcgttatgggctctcctctgctatttgtgagcctcaaacggttcaagaagcaatgcaaagtttagattggaaaatagcaatggatactgaatttttagcacttcagcagaataagacttggtcgttagtacatccaaagcaggctcaaaatctcattgattgtaaatgggttttcaaggtaaaaaggaaggctgatgggagtattgacaggtataaggcacgtctagttgcaaaaggttttaaacaacgttatggactagattatgaggatacctttagtccagtagttaaagctgctactattcgcctcatcttgtctatagcagtctcaagaggatggatccttcgtcagctagatgtacagaatgcgttcttacatggtgttctggaagaggaggtttttatgcggcaaccaccaggatatgaagataaggcacgtcctgaatatgtgtgtaagcttaacaagtcactttatggacttaagcaggctcctcgtgcctggtattcacgtctcagtgcaaaactgctacagcttggtttctcagcatcgaagggtgacacttcactattcttttattccaaaggtcccgtgacaatttatttgttggtttatgtggatgacataatagtagccagttcctctcagtccgctgttgatgcaatgctcaaagacttgcgtgctgaatttcctctaaaagacttaggtgagctgaattatttcttgggtattgaggtcaataaaattcagaatggaattcttctatctcaagaaaaatatacccgtgatattcttgggaaagtgggtatgagccaatgtaaacctgctagtactccgctgcttgtatcagaaaaattgtcgcgagttaatggaacaccactagatgctaatggagccacaaaatataggagtgtagtgggcgcccttcagtacgtcacactaaccagaccagatattgtgttagcagtcaataaagtctgtcagtttctgcataatccgacagatgttcattggacagcagtgaagagaatacttcgctatcttggtggaacccttggtgttggtattaagatttgtaattcatcttcattgcttgttagtgccttttcagatgcagattggggtggatgtcctgatgatagacgatccaccggaggctttgctgtgttcctagggtcaaacctggtttcgtggagtgcaaagaaacaagctacagtgtccagatcgagtacagaatctgaatataaatcgctagcaaatgccacagcggagataatttgggtacaatctattttaagagagttaggtgtaccacaggaaaaagctccatgtctctggtgtgacaatcttggagctacatatttatcagctaatccggtgtttcatgcaagaactaagcacatagaaattgattatcactttgtgagagaaaggatggcaaagaagatgctggatatcagaattatatcaaccaacgatcaactggctgatgggttcacaaagcctctcggagcaaagcagatggaattatttagatacaatcttaacttaacaaagttatgattgagggggga

>Copia-61_ZM_LTR

tgttaagatattcctagtctgttacgatattctagcatctaatcttactttgttaagatattttagcatttatccttatcgtttttctgttttgagtcagactttatctctatccaaagagtccgttgtaatatggttatcagctattataaatagattaggtgcctggcctgttaaagccatgacaccagattacattttaca

>Copia-62_ZM_IN

tggtatcagcctaacgatcctcttcccagtctttcatcctagttcatcatgagtgatacacatagctccgtccacaatacccccacaggcaatcttcctctgtcccaggcgattcacagcgtcaacatcaagtcactggtgccatataccctcgatactcaaattcacaactacggcaagtggagtcagttgttcaccattgtcctcgggcgttttgatcttctccaccatgtcaccacctccaacgatctctcggaggatcctgcctgggttcgtgagaacctgactgtcctctcctggattttcgccaccatctccgaggacctcgtcgacatggtgattcgtccacccggccgccaagcgcatgccatctggcgtcacctcgagaacatcttctctggaaacaaggccagccgcgttgttcaccttgaggctgcgtttcattccctcactcaaggtgatctttcggcgcacgattactgtcatcgtcttcagcaactcgccaacaacctcgccgactgcgatgctcctgtcgacgaccgcacccttgtccaccagctagtcgctggcatgaacactaagtaccacacccttcgtaccttgctgcctgctcttcctgtcttccccaacttcatgcaggctcgcgacatgcttctgcttgaggaacgctcccagtccgtgtcaaaatcgccatgcggccgatgcggcactcgttgctgctgctacctcgtcgcgctcgccagctcctctccccgccaccgacaacaacaacacaactgcaacccccacagttgacaaccgccaaagcagctatggccgtggccgtggtcgcggtcgtggtggtcgcttcaacgctgctagacgcagcggccggtaccctccggcgacctttcttcctccatggcctgcacactgggctcccacctggcgcgccccatggacagggacctctggccctggcgtccttggtgcacgtcctccggcgcaagcctatccggtattccagcaaccacaatctgtgtctgcgccgctgcatgctccgtcttgggacacttcggggcttgttcacgcgctccaggctgcttctgttcaacagcaagcacatcccagtgattggtttctcgacacgggagctagctcacacatgaccaaccattcgggtaacttacctatttattattcttcatcattgcctaattcttcgcatgttattgttggtgatggatccaaaattcttgttcatggcactggtcatacctctatccgttccccgccaaacacttttgctttacgttctgttttgcatcttccacacttagtcaagaatttaatttctgttcgccgcttcactactgataactcgtgtgccattgaatttgatccgtatggtttttctgtgaaggatctagccaccaagaacacaatcatgaggtccaatagtgacggggatctttacccgttctttggaactagctctccttccttgacggctctggtggctcacactgcatccgctgatgtctggcatcgccgtcttggccaccccaataatcactccttagctcgatttcctttttcatgtaataaatctttagctgcttctactctgtgtgacgcctgtcagaaggggaaacatgttcggctctctttccctcgttctacctccttcacatattttccctttcaaataatacactgtgatttgtggacatcccctatttctagtatttctggttttcactattatttgattgtcctagatgattatactcattacgtctggacatttcccattcgtcacaaaaccgaggtgccatctattctgcgcaatttttatagctatgtacgtacccaatttcatctttctatccaatctattcaatgcgataatggccgagaatttgacaataacgatctgcgcacttttcttttagccaatggtgtcaccctccggctctcatgtccatatacatcttctcaaaatggtaaagccgaacgtggcatccgcacaataaatgatattcttcgcactttgcttgaacaagcctccatgcctcgttcatattgggttgaagccctccatacagccacatatcttcttaatcgacgaccttgccatacccctgttgacaccaactctaaacccgcctctaccgatggttgtcttctttcacaacccactatttacagaagcttagcaggtgctttacaatatcttactttgactcggcctgatatttcttatgctgttcaacaagtttgcctgttcatgcatgctcctcgtgacactcatatgtctttgctgaaacgcatccttcgctatgtccgtggtaccttggactatggtttgcgtatctatcgtagttccagtcttgatctgttggcatactctgatgctgactgggctggatgtcctgacacccgacgatctacatctggttattgtgtttttctgggtgccaatcttgttgcttggtcgtctaagcggcaacatacagtatcccggtccagtgccgaggcagaatatcgaggtgtggccaatgctgtggctgaaatttgttggcttcgccaattgttgactgagctttgtcatcctcctcgtcgtgctgctgtggtcttctgtgataatgtcagcacaatgtatctggcatccaacccagtccaacatcaacgtacgaagcatgtggaaatcgacttgcattttgtacgcgagcgcgtatctttaggagaggtgaaggtgatacatgttccttcgtctcttcagtttgctgatatttttaccaaaggtctctcatcatcattgttcaaggagtttcgatccaacctgcaaatcaagccagctacagattctacaggttgaggggggc

>Copia-62_ZM_LTR

tgttagataatgcctattttcttgtttctgtccgagtctttcggccacttcgtagcccattattttaggatcacaggagacttcgtggccatcttttcaggatcagacttgcatggtccttggttgtaatctatatatatttgtactcgtcacatcaataaagcaatacagagaggacatttctttctattcctattctccgtttcttaca

>Copia-63_ZM_IN

tggtatcttgagcggctcgcttcctgcgaccctcgcttccgcatcccagccgccggcgtttctcgcccgctgcatcttccctcccgtcatcccctccccaccatggccgacaacagggcagaggcagagcgccttgctgctcaacagcaagttcttgatggagaacgtgctcgccttgaggctgatcgccggaccttcgaagaagcccagcgcgcaatgcaggagcagcaacgtgcgctggaggccacgaccattgccgcactccatacccaggcagtggctgtcctcaacatccgcaccctcattcccgtcgtcctcgacaccgactcaccaaactacacacaatggcgcggcctcttcctcctcgccgtgggcaagtacgagctgtccgaccatgttctctccgacacctcgctctccaccgatccggcctggactcgcatggagtgcgttgtcctcagctggatcttcggcaccatctcggcaagtctagtccagcttgtcatgtcgcccggcacaactgcccgacgtgcatggctcagcctagaagaccaattcctcggcaacaaagagacccgtgctttgtacctcgacgccgagtttcggaacttcacccaaggcgacctcaacgtctctgactactgtcgccggttgaaggccatggctgatgctcttggtgaccttggtgaacctgtgcaggatcgcactcttgttcttgccgtcttgcgcggtctgaacgagcgttttgctcatctggcgtctctcatccgtcgcgccaagcccttcccgtcctttgtcgaagttcgctcggatcttcttctcgaggaactaaccatgcagaatcgccccgtcgttgctccaacagccctcgtcgccaccggccagcccgccagcactggtcgccgcggcaccaacaacagccgcaacaatcgtcgcggtggtcgcaacacccgcggcaactctgcgtccactcccgctgctggccaacgctcccaggtggcccaggccgcgcctcctccacgtgctgacctcccctggaacccatggtcaggcactgttcagatctggccacatcctggggcaacctccatccttggaccaaggcccaacttggctcctcaagcgcccactgggctctacgcggttggtgggctggctgcctcgtccccaactgctgccgtgcctactgggtacgcaacaccacctcctgcaactccaacgcaaggcattggatggaaccaggcggatcttctccactccttcaacacgatgacgctcaaccctccctcctcgggtgaatggtatatggactccggcgccacatctcatatgacctcagatgctggtaacatcaccacttcttatcctccctctccttcccactcctccgtaatagtgggcaatggatctacacttcctgtcacatccatcggttccactaccattcccttatataatcgtcaattttctttacgcaacattcttgtcacccctaatattatcaaaaatttaatttctgttcgtcaatttaccactgacaataattgttctatcgaatttgacccttatggcctttctgtgaaggatcttcacactcggagcgtgatcgccaggtgcaatagctctggacatctctaccccttgttgccatcgccgccccaagcattcatcacctcttcatcctccacgttgtggcattgtcggcttggtcacatcggtgatgaggccctctccaaactatctagcactaattccatttcatgtaataaagccactgagtcattatgtcatacatgtcaactcggtcgccattgtcgtttaccttttagtcactctagctctcgtgccaaagccaattttgatttaatccattgtgatttatggacttctccggttgtcagcatttcagggtacaaatattacctcattcttcttgatgattgttcacattattcttggactttccctctacgccttaaatctgacaccttctccactatttctcacttttttgcctatgtccgcactcaatttggcactgtgattaaaagtattcaatgtgacaacggccgtgagtttgataactctgtcgctcgtcatttctttctcaaccatggtgttgtgcttcgcatgtcatgtccacacacctcccaacaaaatggcaaggccgaacgtattattcggtccaccaacaacatcatgcgctcccttatgtttcaagctagcctcccaccgtcctattgggctgaagcccttcataccgccacatatcttctcaaccttcatcctacaaaaacacttcaatttcgtacaccataccatgctctctacggtgtgcagccatcttattctcatttacgtgtttttggatgtcgctgctatccaaaccttgcatccaccgcacaaaataagctttcccctcgctctacattatgtgtttttctaggatatccatccgaacataagggctatcgttgccttgatttatcttctaaccgtatcattatatcccgccatgtcacttttgacgaaacatcttttccttttgccagcaaccaatgccccattgaaccaacgtcgcttgattttctcactgacatggactccgtccagcttcctgtcttttctcctgcaggtaactcacacaatgtgcacacgtctcctacacaggctgctgctggccagcaacccagtagtccatgtgcccctgtagtcttgcgcctgcagcctgcaaatggacctccaggcgacagcccgccatctagccctcctgcacagcccacgtgcgggcctcgcagggaattggttgggccatccttggctgctgcgcaacccacgtctgctcctcgtgcatactctccaggcgaggtccaagcaacgtctgggcaaactttgccaactcatgaacttccaccctcaacaacgcatgagtgtcctcctgtttgtgttgaacacacccgtcaccagatcaccactccagctgctccaggaggattggctcccacggtcccagtcgtgtccactcgaactggccaccgtactgcagtctcagtctccccgacggccaatacacatcagatggtcactcgtggcaaatcaggtttccgccttgacactgaccggctcaatcttcatgttgcagctatttcaccggtcccaacttctcttcgtcgtgccttggctgatccaaactggcatgctgccatgaccgaagagttcgatgctttacaatcaaatgagacatgggatcttgtccctcgtccatctggcgtcaatattgtcactggaaagtgggtctaccgtcacaagctgcgtccagatggctctctcgagcgttacaaggctcgttgggttcttcgaggattcacccaacgtcctggtattgattttgatgaaacttttagcccagtcgtgaagcctgtcactattcgcaccgtgctctcactagctctctcacgtgactggcctattcatcaacttgatgtgaaaaatgcttttttacatggaactttgtctgagacagtatattgtgtgcaaccctctggttttgcagattctactcacccagattatgtctgtcgccttaacaaatccctctacggattaaagcaggctcctcgggcttggtacagtcggtttgcatctcacatttcttctctcgggtttattgaggcaaaatcagatacctccttgttcatcttctctcgaggctcagatatggcttacctgcttttatatgtggatgacattattctaacagcaacaaactatagcttacttcagcgcattatcacagctttacagactgaatttgctatgaaagatttaggtaaccttcatcatttcttgggtatctcggtaactcgtcaacccagctctttatttctgtctcagaagcattacattcttgatatactggatcgtgctggcatgacagattgcaaaccttgcaacacacctattgatacttcatccaaactctctagtgatggacctcgaatatcagacccgacacaatatcgcagtttggctggtgctcttcagtatctcgtgttcacaagaccagatattgcttatgctgtgcaacaggcctgtctcttcatgcatgatccccgcgagcctcatctagccttggtgaagcgtatattgcgatatcttcgtggtactttggactatggattcttaattcacaagtcttcattggacaatttaattgtttacacagatgctgattgggcaggttgtcctgacacacggaagtccacctctgggtatgccgtttttcttggagataatctattgtcgtggtcttccaaaaggcagccaactgtgtctcgctccagtgctgaggctgagtatcgtgctgttgctaacggcgtcgctgaagctacttggctccgacagctgcttctcgagcttcacaaccctttgcatcatgctgctgttgtttactgtgataatatcagtgcagtatatctctccagcaatccagttcagcaccagcgtacgaaacatgtggagattgatctacatttcgtccgcgagcgtgtcgctcttggccacgttcgggtccttcacgtgccgactacttcacagtttgctgacatcttcaccaagggccttccatcttctgtcttcactgagtttcggactagtctgaacgttcgacctcctcccgctccgactaaggggggg

>Copia-63_ZM_LTR

tgttagacggcttgctcatgcacccactaccgtcaggtgacctggatgagcaacattagttgcgtcatcagcggctccatctgcatgcacgtccaggtgcattgctcttcaccggtgtaatcatttagcatcccatgattagctccttccatgtatagctgcatgtaatctaggattgcttgccgtgattgttgctatcactggctatttgtatctgccgatgtaagggaacagagtatcgtgtgaatcattctctctgtaatacaccttcacactttca

>Copia-64_ZM_IN

attggtatcagagctaggcacttcatcttgagtctaacaactcgaagtgatggctcgtagaagatcccaaaagaacaagaagactcctccaacgaacgcaactcaaaaggaggtaactcttgagatattatttgatgattgttctaattatgattcatggtccactagtgtgataaatgcttttagaaccatagatccacgattagaacaaattatagacaagagtatttttccctctaatttcgatggagaaattaattccgaggaggatcaaagatgttatcgcttaaactatctagcttttgacatcttaactaattctcttagcaaagaagattatcgtgccttcatatcaaactatgacgaatccattcgtgatgcgcatgatatttggactagaattaaaagcaaatttgatgagtccaaacatgatagttcattttgtgcttctacttcctttggtacttgtgatactaacccttgcaaggaagaagaagaaaatgaacgatggagaccaaacgatgaatccacctctccaaaaggtttgtcttcccatttcgattcccacatgtgttgtgtggctaatgaaaatgatagcggaagcacaaatgaggatgaggaggaagaaagaagcttcgtgcaactctacgctcgcctaagccaagaagataaggcggtcatgctcaaacttctagaaagagcgagagagcaaggcgaagctcgtcaaaggctagaagatattctctccatgaaaatgctacgctttgacgagttgactaaagaacatgaggagctaaagtgctctcatgttgatttggtccaaaggtatgaaactatttcaattgagcaagataacgctttacattgtatcgctcaattagtaaataggaataccttgcttaaggaccaagtagaaaagctaaaagttgaaaatctagcttttcaagaaaaatatgatatgctcctatgctctcatgaaaatcttatggatgatcatatcatgttaaacattgctcatgaggttgtgatagaaaacttaaaattccaacaacctcactcatgcacatgtgttcatattgatactatattaccatgtgctaatgcttgttgtccgtcgacaagcaaatcttcttttgagctagaattcgcaggaacaaatgatgatacatatcaaaagctcaaagaagaaaatgagaggctaaagatgagcttgacacaacttaaagggaagtgcattgctcaaccttctcaagataaccgtgatcacatggtgaagaagcttgagacgggaacaaccgtggcatgcactacatcccttgaagaaaatgtcaaggacttgaggattgccaagaagaggaagcaaaagaagaaaatcaacacctcttccaaaagcctcaaccatgcctccataaaaggtaacatccaaggtaataatcaagccacacttcacactaagaaaaataagaagtgtagtgaatgctttgaagagggacacttgattaggtcatgtccctacattaaaaatggcttgattattaacaaggatgatagactttgttttaaatgctcaaagaagggacacttaatcaaatcttgtccccatttgaaacaaagaggcatagggttagaaaagaaagtttttactaaccatgtagcaagcaacaaacaaggaaagaagaaatcttcaagacttcaaaaacgcctatgctacacatgccggaaaaagggacatcaatgcaaggattgtcccattggtaacaaccccacccctagcttgtcaatcgtttcgcatgtaactaggcaacccaaaattgcaacttgtgctagaaaggtaacgagtttaccaagcgctaacacaaaggacttttgggttcctagatctttgtttactaaccttaacggacccatcaagcgatgggtaccaaaatgtgcttgacaagctttgtaggaggaggagatggtatgaagctttggggtgcttgagggagttaattcaattcttatcgactcaagctatcaatcttcaaatgatctacatattcaagattgacccaaggttacttcaatttattatattcaaaacccatatcatctcgggaagatattgatgttgtaggaattgaagaatcatcatgtgcggaaaatcaaggcctacaacatggaggaatgtcaaaggatggtaacacttatgcttttaagtgcaagtatcttaaattatcatttcttatgtgtcttgtgtagtcacatagaaaaaggaagcacttaagatgtttttaaaattatgtcaccattctttgaagaaattcctctcatatggtagattgcatattcttcatttctatattatggcaatctacatgctttaaaattgttgcaagttcccatggcatgttttactttaattatcctattattgccatgatctagatatagaaagagattccatgttcttaaaagaataaggtgtcatgtaaggaattcaaatcctaaggacacttataaaagggaatctttctttatgtctagaatagtgagactaatgtttttatctaagtaacccaagtagtctcacttgtagagaataagtttctctttggagatgcgtaatccatcatgacaaggaaaatcaatccaataatttatgttgtatgtcttattgcttaaattggatatgattcttctacatttatcgctttccatatcatgaattagatttattcccatgatcttaaaggattggttatgcttgttttatgagttaaaattgagcataacatcttctatggataatctagttcatgctatgaagtttccatgcttttagtaatctagtttttcattccttatcaaaatgattacaaaaagggaaactagtgcttgtgttgccattaacctttcgcttgagcttacttatgaaaatcaacaagagagtatgtgcaagtttaatgacacaagcctcaaagggttgatcttcacccataggaagaaaggtaaaagcaaaggtatgggaactcatctccttaaccaaatgtagttcccatcaatgactgtttactataattatatctttgtgaagaaatagattctttattgaatttaaattggctaagacgtgcattcaacctcaatctcattaatgcacttgtccattagaatctatttcatacctttgctataaatgttctcatattgacttgtttttaaagtaatgattaataaactcaatatgaagaagtaaattcctatgattgatcaaatggttaaatagtgcatattcctcttttcctatgatgtgcactaatgctagggattttacttcatgtctgtgtgacttatggatgatgaattgttcatatttattttctaaaagaaatcatcattcatcatatactcccttgcgctattgacatctctcttgagtattttcaataagtttggaaggaaaaagagacaactacaaagggaggacactcaaatgaaaaaggaagaacatcaagaacaacaacacctacttggacaataaggtcttcacaacaatcaatggtgtggttgtaagcattctaaatctttttcattgtgagaatacaaggcttaagttgtttattgcaaattttataagccttgatcaagatgtataatgggtctccctttatgtctttaaaagtgaatgcatcgaaatcaattctttcaattacttgatgcatatctttagggggagtccattcctatattttgattatgttgagactattgctttatcttagtaatttcatatagtctcttacatgagaataatgtttctcataaagtatgctacttcacatcaatccaacttgttaaaataatgtcgcttttatcaaggattgttgctttcattgctaaagtagcttgcttattggattctcctattttaagcttaattcatattccaatatgttactctcttgatcgcatccattgtttgtttgatcatagaataacatcaaatgacacttagtgcctcattccttcaaatcgatatctctctaagatatgcactaagttaaaaggagaattcatgtttcatttatgcaatttgtgatccacttgatatatgttaatcatgacttggaaaaggatcactagttgtagaactctctcttgtgcaaaatatttccatatattgtcttgagtataggtcttaagcaactaagactaaggacaaagcacaatgaagagagcatctctatttgaaggtacaaaagggtaaaactacttcctttcattcaaacttgtacctaaaatcttccttttctatactttccttacatatcttgtataaaaggaagagaaagcatgtccatgcatttttccctgcttcatacctagtttaacctctcaaacatttcattcctgcatttatgcatctttgttaaaactggatgaagtaatcttattgtcaaagagcttaaagcttaaccttgtaacgaggataagctgcctttgttccaaaggtggatggtccttaagcctctttgaaatccttaagggaaaatgcttaagacgcttataacatgctttcataagtgcataaactgtcatgagcatcacacttatactaggacacaatgcacttcacattctgtatggtatagatatgttctcattaacctcattatgtgcaattggcatttcaaggccaaaatatgtattcctatcaaggcacatatttagggggagcaatctatattatatagaactatgatcatgcttaattgacatatcttttgatcatatctctttcattttggtacaaatgcatatatcttattattctcgtaccatgactacgactaatatgtttccaagtatatttctatactaagtcgtagattgaaagggaaatggagtcttcggcgaagacaaggcttccactcaactctatcggtaccatttatccttcgccatcactccacactagctctccacattggtataatctttcactcatatattatttgccaaagggggagacaacttacaaaagggcttatatttcactcaaagtatccgtttttggcgattcatgccaaagggggagaaagtattagcccaaagcaaaaggaccgcaccaccaccaattttcaaaatttaaaaggtcttgaaatgatgaattttcaattggtatagtctttcaatttggttttaaagaagtattttcaaattggtatcttacttgtgatataatttcaaattggtataccctcttcaaaattaatatcaaaaccctcttgaacactaagaggaggatttcattaagggggagttttgtttaagtcaaaggaaaagcatttgaaacagggggagaaaatctcaaatcttgtaaatgcttctcaaaattcttattcatctacctttgactatttgcaaaagactttgaaaagaatttccaaaacgtttgcaaaaacaaaacatgtggtgcaagcatggtccaaaatgtcgaaaatataaagaaaccattcatgcatatcttatgaaatcaatattggtttaattccaagcaatctttgcacttaccttaggcaaactagttcaattatgctcttatatatttgctttggtttgtgttggcatcaatcaccaaaaagggggagattgaaagggaaataggctttaaaccttttcctaaatgattttggtggttgaatgtccaacacaaataattggactaactagtttgctctagattgcatgttctacaggtgtcaaagattcaacaaaaaccaatagaaagaacaagacagggttcaaaacgaaggagcaaaaagaaaccgaagtgctccctggtctggcacaccggactgtccggtgcggcaccggacagtgtccggtgcaccacggactgtccggtgcaccagggaggattgccttcgaactcttcaccttcgggtttctcaggcgcagcccactataattcaccggactgtccggtgcaccaccggacagtgtccggtgctccagaggaagcggctccgaactagccagcttcgggaaaacggaaggccgctccgctaaaattcaccggactgtccggtgtacaccggactgtccggtgcgccggcggagcaacggctacctcgcgccaacggtcgactctgacagcgaacagtgcgcaacagtacacgcggcagaagtcagagcagagagcagaggggcaccggactgtccggtgtgcaccggactgtccggtgccacacgaggacaaagcctccaacggtcgaccagctccaaccccaacggataggatgacgtggctggcgcaccggacactgtccggtgtgcaccggac

>Copia-64_ZM_LTR

tgtccggtgcgcccatcgccagcagccttctccaacggctacattttggttggtggctataaataccaccccaaccggccacttcaaggtgtgggagtccaagcaacattccaagtcatctagttgacatactcaagccctcccaaccacatatattcattgatacatcctatacacaagatctagcccactacaaccaacacaagtgccacaaaagagagagcgagcaattgagagctactcaattgagtttagccctagtgccttgtgagatccattgagagatagtgtgtgctwcatctttgtgttcatttgcgcgtggagttttgactcccattcgaacttcctccaaagtgttggaggcttgtaaaagctagcaagagacaccaaagagtgtggtggtccttgtgggatcgagagtgatccttgagaagaagaagagctcaccgatccttgtgtgatcggtggagagagggaaagggttgaaaaagacccgtccttgagtggactcctcaacggggactaggccttcgagggccgaacctcggtaaaacaaatcacccgtgtcmtttgtgtttattgcttgtgatttgtttgttttcccttctctaagttttcttgcactattctttgctaatatcatttggtgttgcttcaagttaaattctcatttagtgaagcaacacttcgcaagaaagaaacttgacttattgctcttctcgtctaagccttcttgctttactatccatatatctagtagttgtattgattatcaattccgcattatttcaaagcaacactctttacaagcaaaggacttagcttttatactccgataattgtttatcttgttctaaccactaatcaagggatctagttgggggataaagttttaattttcaggtttcgcctatccaccccccctctaggcgactttca

>Copia-65_ZM_IN

attggtatcagagccgttctcttcatcaagggactaaccgcccgaagagatggatcctaaaggcaaggggatcgtgatcgacgagaaggagaaggagaccgtcaacgacaacgagccaaagggtgaaaagcccatcgactcaggctcaaacaacaagaagaaggacgggaagaagaagaggcgcatcaagaagatagtctactacgacagcgacgactcttcttcttcaccaaaggacgacgacgacgactcttcctccaagcaaaagacggttaaacaaaactactctaaaacgtcttttgattattcccgcattccgtacaactccaatgctacattttttgtctattcctcttggcaaaccccctcattttgatggggaagattattctttttggagccataaaatgcgtagtcatcttttttctcttcatcctagcatttgggaagtagtagagaatgggatgcattttgatagtagtgataatgctatttttattcatgagcaaattcataaaaatgcccaagctactactgtgcttctagcatctctatgcagggatgaatacaacaaggttagtggcttggacaatgccaaggagatatgggacaccctcaagatcgctcatgagggaaacgacgccacaatgatcaccaagatggagttggtggaaggcgagctggggaggttcgccatgaagaggggtgaagagccaacagacacatacaacaggctcaagaccctggtgaacaagatccgaagctatgggagtacaagatggacggatcatgacgtcgtgcgcctcatgctaaggtcatttaccgttattgatcctaaccttgtaaaccttattcgtgagaaccccaggtacaccaaaatgtcgcccgaagaaatccttggaaaatttgtaagcgggcgcatgatggcaaaggaggcgaggtacgtcgacgacatcgccaacggacctcttccacactatgagccgcaacccgtggctctcaaggcgacggcaagcaaggaggcgctccccgacaaggtggcgcaaattgaggcggccggccttaatgaggaggagatggcgcttgtaatcaagcgcttcaagaccgctttgaagggacgcaaggactaccccaacaagagcaaatcaaggggcaagcgtacatgcttcaagtgcggtaagtccggtcattttattgctcaatgtcccgataatgaaaatgaccaggaccaagagaagaaagggaagaaggagaagaagaagttctacaagaagaagaagggcgaggcgcaccttggcaaggaatgggactcggactgctcttcatccgactccgacgacgagggactcgctgcctccgccttcaacaagtcctccctcttccccaacgagcgacacacatgcctcatggctaaggagaagaaggtacataccgtgatactcctaagtatacttcttctagtgatgaggamtctgatgatgatgtagattatagcgatcttttcaagggcttagatagatctaaagtagataaaattaatgaattgattgatgctcttaatgaaaaggataggttgctagaaaagcaagaggatattctttatgaggaacatgataaattgtagatgttgaaaaatcccttgctttagaaatcaagaagaatgaaatgcttgcttttgaattgtcttcttgccatgactctatttctagccttaagagtttaaatgttgatttaaatgctagaatagagaaattaagtgttgctagttcttctttggagcatgtttcaatttgcgatagatgtaaggattttgatgttgatgcttgcaataatcatgcttctactatttcaaagttgaatgatgaaattgccaatcttaatgctcaacttaaaatttgcaaaaatgaatgtgaaaaagttaaatttgctagggatgcctacaccattggtagacatccctccattaaggatggacttggtttccaaaagggaaccaaggacacaaagagccaaaaggcccccagcttcattaaggagaaggggaaggcgcctatggctagtagctcgcattcttttcatgagaagaagaaccatgcttatttgtatgctcatgttaagaatgcttctcatgatgctcatatgttcatcgtgatgttatgtctgttttacctgtgcgtcatgatgctgtttttgctcctcgcaccatgaatgcttcttctagtgatctcatgctcatggtaggagtagacctaggcgccgtgctcctaatgttgtttctcacgcgcctaggaacgcatctcatggtccttccatgctttaccgtacttatgatgcctcctatgtgcttcattgtaagaatggtagagtatgtgctaaaaatgtgggacctaaatgcaagagaggtaagacttgcatttgggttccaaaacttatgtaactaaccttgtaggacccaacaagagttgggtacctaaatcccaagcctaaattgccttgcaggtttatgcatccgggggctcaagctggatcattgatagcggatgcacaaaccacatgacgggggagaagaagatgttcacctcctacgtcaagaacaaggattcccaagacatgatcatctttggagatgggaaccaaggcaaagtcaaaggtttgggtaaaatagcgattactaccgagcattccatttctaatgtgtttttggtagaatcgcttggttataatttgctttctgttagtcagttatgtcacatgggctataattgtctatttacaaatgttgatgtaactgtctttagaagaagtgatggttcattagcatttaaaggtgtatcagacggcaagctttatttagttgattttactaaagagaatgccgatctagatgcatgtttaattgctaagaccaacttgggctggctctggcatcgccgtctagcacatgttggaatgaagaaccttcataagcttctaaagggagaacatgtgttaggactaaccgatgtctgttttgagaaagacagaccttgtgcagcgtgccaagcagggaagcaggtgggaacaagtcatccaagcaagaacgtgatgacgacatcaagaccattggagcttcttcatatggatctcttcggacccgttgcttaccttagcatcgggggaagtaagtatggtcttgtaattgttgatgacttttcccgcttcacttgggtattctttttgcaggataaatctgaaacccaaggcaccctmaagcgcttcctaaggcgagctcaaaatgagttcgagctcaaggtgaagaagatcaggagcgacaatggatcggaattcaagaaccttcaagtggaggaatatcttgaggaggaaggcatcaagcatgagttctccgctccctacactccacaacaaaatggtgtagtggagaggaagaaccggacgctcatcgatatggcaaggacgatgctgggagaatacaagacgccggagcggttttggtcggaagccgtgaatacggcttgccatgccataaaccgcctctaccttcatcgcctcctcaagaagacggcatacgagctcctaaccggtaacaaacccaatgtttcttactttcgtgtatttgggagcaaatgctacatcttggtaaagagaggtagacattcaaagtttgctcccaaagccgttgaagggtttttacttggttatgattcaaatacaaaggcatatagggtcttcaacaaatcttcgggtttagttgaagtcactagtgacgttgtatttgatgagactaatggctctccaagagagcaagttgatcttgatgacgtagatgagaatgaggttccgacggccgcaatgaggactatggcgataggcgatgtgcgaccgcaggaacaacaagtgcaagatcaaccttcttcctcaacgatggtgcaacccccaactcaagatgaggaacaggtacctcaagacgatggcatggatcaagggggagcacaagaacaagaagataaggaggaggaagaagtaccacakgcacctccaacccaagtccgcaccaacattcaaagggatcatccggtggatcaaatccttggtgacatcagcaagggagtaactactcgttcacgtattgctaatttttgtgagcactactcctttgtttcttctattgagcctttcagggtagaagaagccttgcaggatccggactgggtgttggccatgcaggaggagctcaacaacttcaagagaaatgaagtgtggagtttggtgccacgtccgaagcaaaacgttgtgggaaccaagtgggtgttccgcaacaaacaagacgagtacggggtggtgacaagaaacaaggctagacttgtggcaaaaggatatgcccaagtcgcaggtttggattttgaggagacttttgctcctgtagctaggcttgagtctattcgcatattattagcctatgctgctcaccactcttttaagctctttcaaatggacgtgaagagcgctttcctcaatgggccaatcaaggaggaggtgtacgtggagcaaccccctggcttcgaggatgacaggtaccccgaccatgtgtacaagctctctaaggcgctctatgggcttaagcaagcaccaagagcatggtatgaatgccttagagatttccttatttctaatgctttcaaggtcgggaaagctgatcctactctttttactaagacttgcaatggtgatttgtttatatgccaaatatatgtcgatgacataatatttggttctactaatcaaaagtcttgtgaggagtttagcagggtgatgcaacagaaattcgagatgtccatgatgggagagttgacatacttcctcggatttcaagtaaagcaacacaaagacgggacgttcatctctcaaacgaagtacacacaagacttgctcaagaagtttgggatgaaggacgcaaagcccgctaagacgccaatgggaaccgacggacatttggacctcgacaagggaggtaagtccgttgatcaaaaggcataccggtctatgataggttctttgctttatctttgtgctagtagaccggatataatgcttagcgtatgcatgtgtgctagatttcaatccgaccccaaggaatgtcaccttgtggctgtgaagcgtattcttagatatttagttcatacgccttgcttcgggctctggtatcctaaggggtctacctttgacttgattggatactcagactccgactatgccgggtgcaaggttgataggaagagcacatcagggacttgtcagtttctgggaaggtccctggtgtcttggagctcaaagaaacaaacatccgttgccctatccaccgccgaggccgagtatgttgccgcgggtcagtgttgcgcgcaactactttggatgaggcaaaccctccgggactttggctacaatctgagcaaagtcccactcctatgtgataatgagagtgcaatccgcttggcggataatcccgttgaacacagccgcactaagcacatagacatccggcatcactttctgagagaccaccagcaaaggggagatatcgatgtttgccatattagcaccgatcaccagctagccgatatcttcaccaagcctttagatgagaaaaggttttgcaggctgcgtagtgagctaaatgtcttagattcgcggaacttggattgatctatagcatacatgtgtttttatgcctttgatcatatttttgagctttattgtttatttttgtgctcaagttgtagaacgtcatccccggacctcacaagtccttgtgcaaatgatgcatatgtttagggggaggatatgctacaacttgaccctttgagactaatgtgtttgttgagttatgtgatgtagtctcaaaggtgcattgaaagggaacatgtgaacttggacaaagaaaaaggcttccactgcattacggtatcaatgtattttgttccaagttgcccttgtgtttttctcattgctttcgatgtttattcgcaatttttggtgaggcaatggggttaaaggccactagtttctccgttttggtgcttaatgccaaagggggagaaattaaggccaaagcaactggatcaaccgccacttgtgaatttcaaaaatttttgtgttttgaacttgtctatttgatcaaaaccctcttgatagcaaagaggagctctctaattgcaaaactactctcttttgggggagaaatctttttaagagaaaaagggggagcttttggtttttgatcaaaactagtctttgaaaatggtttgatttgcaaaaataaagtgatttgacttagaagtaagaaaaatgaatttgttttgcaaaaataaaccaagtggtggcaaaatgatccaaatatgccaaatcctgtgataattcaattggtttttagtttgacttcaatttgcacattttggctcacatttggttatgttagtgcattttaagttgcttttgatgtgttggcataaatcaccaaaaagggggagattgaaagggaaatgtgcccttgggccatttctaagtgttttggtgattaagtgcccaacacatcactttgaactaacatgccttctatgagcgtgagcacaggtgtaaagaaagcaaattgaagtgatcaagtccaaatgaggtgaattggaggaccaaaatgcaacttgttcaaaaccaacatggaaatatggtttgagtgtttgataagttgcatatccctcacctttttagcactttgttttgaacctgtcaaagaaagtgcattttggacttgagatggagcatattgcaaatcctttggagaaatggataaaggtatgtttctagcacttagtcaattgttttagttgctaactatgttcatctaagtgctaggaactcatcaagtcgagcaaaattgaagaaaagaagtttggctaagtttggccaaacttgggccagtctgggcgcaccggactgtccggtgtgcaccggacagtgtccggtgcccgcctggtcaacggtcgactcgcggccgtactggccgctctcgggaaaagagccaggcgcctggctataattcaccggactgtccggtgagccagccggccaacggctctcgccgcgccaacggtcggcgcgcaatcagcggcggccacgtggcgggcgcgtcaccgcgccaacggtcagcaggttgcaccggacatgtccggtgcgcaccggactgtccggtgcgccaggcgagcctcggctgccaacggtcggctcggcccagtaaggaaggaaatcggcactgtgcagtgtccggtggtgcaccggactgtccggtgcacccgcgcgcagaaggcagacagaggccttccaaatggaggascaacggctccttggccccttggggctataaaaggggcccctaggcgcctskagcagatacccaagcacactttgagcacactacaactccgagactccgcgaccacgctttgattcgttagagagagatttgagcgcgttcttgagctgtgactctgtcgttttgattcgtgcgctctcttcttcgcttgtgtgcgtgttgttgctgcgattgtgctcttgtgtgcgttkctactccctccttactcttgttttgattgwgatcatttgtgtaaggcgtgagagactccaatttgtggagattcctcacaaacgggattatgatataaggaagacaaccgtggtactcaagtttgatctttggatcacttgagaggggttgagtgcaacccttgaccaaaggaggtcaccacaacgtggatgtaggcattggccgaaccacgggataaaatcgctgtgtctctttgtccatttactttattgcgattattgttcttcctgagctctctacttcacttgcaatattgctcctaagtttaatactcacctcgwgggagcaatcaag

>Copia-65_ZM_LTR

tgaagaagtcctctttctcctctcactcttctcattcgaacttggttttagatttcactaatccatttttggaccaagtttgttttgtttagagctgtttttacaggatcacctattcaccccccccccctctaggtgctctca

>Copia-66_ZM_IN

attggtatctagagttagggtttagacacatcagtctttattctagggaagtcgtcgctgtcgccgttcttcgtccgtggaccgtacggccaccgcctgtgtgcgtccgtcaagagccaccaccgctctctggttttcgtcagatccgtcgccaccgccactttggttctcatcagatcggtgatcgcgtagctgccatctacacgctgaggcgatctacgcgttggcgcttcctcggactggattcaagacaagccggtggtttcctttcttcgcgtggtctgttcaaggtgaatagttgctctgcaatctcacgcgaaggctgctactgggatcatcgttgcgcatgttttggaatctccgcgcaaggattaaaaggatattgttggtgctcaagaaatccgcgtgaagtccacgtatttccttactacgtgcactcatcagattgcacacggattatttcacttgggatttttatttcggattatgatgtcgactaaaattgatattccgaagtttgatggcaaaatcagttttgctatttggcagattcagatgaaggctgttcttactcagttatgtgtgcgaaaggcgttgcaaccgcgacctgctgatatggctgatgataaatgggaagatcttgatgaaaaagctttgtccgccttacagctcagtttatctcctgatgttctgcgcgaagtaatgaatgcaaaatctgcggcagaattgtggaagaaattggaggagctatatatgaccaagagtcttgctaataagctccgtctcaaagagcgcctttatactattcgtatggcggaaggtacatctattcaatcacatctcaatgaatttaattctatttgtgtcgaccttgagagtctggatgtgaagatagatgatgaagataaggcaattttgttggtagtctcgttgcccccctcttttaagcattttaaggaaattatgctctacggaaatcatacttcactgacatttgagaatgttaagtcaaatttattgtccaaggaaaaatttgatgtcgattctcgttctgagtctaagggtgaaggttttattgttcgaggtagaactcaacaagcgggcagttctaataaaccaaaatcaaggtctaagtcaagagatcgtaaatccaacaatttttgtcgttattgcaaagctgataatcatgtgatctcccagtgtcctaagttgaaaaataaggaagaaaggcaaaagaaaaaggaagctgataaatcagctgctgaggctagtattgttgaaaatagtgatggtggagaagctcttatgatcacttctaatgatgaaaagcgtatggcttgggttcttgattcagcttgctcttttcacatttgctcacatcgagagtggttttctgattattctcatgttcatgatggtgacgttattattggtgatgaatctccacttgaaatttctggaattggttctgttcaaattaaagttcatgatggcacattcaagacccttactaatgttcgctatgttcctaagatgaagaggaatcttatttctttgggcaccttagaagcaatgggattcaaattttctgctgataatggtgttcttaaggtttctcaaggcaaccgtgttgttttaaaggccgaacgtataaataatctctattatttgcaaggttccacagttacaggtactgcagctgtttctattgcatccaacacttcaaataccaagctttggcatatgcgcttgggacatatgagtgaaaaaggtatgcatctcttgcacaagagaggttatttaaatgatattggcaaattggaattttgtgaacattgtgtatttggcaaacagaagagagtcagtttctctttatctactcactgcaccaaaggcattcttgattatattcattctgatctttggggtcgggctcctcattcttctattggaggttgtgattatatgatcacttttattgatgatttctctcgcaaagtctgggtttattttctgaagcataaaaatgatgctcttactgcattcaagcaatggaaagctttagtggaaaatcagactggcagaaaaatcaagaaattgagaaccgacaatggattggaattttgcaatagtgagtttaattctctttgtgctgatcatggtattgctagacacaagactgttccaggtactccccagcagaatggtgttgctgaaagaatgaatcgtactattctggagcgtgttcgttgcatgctttctaatgctggtttatgggataagcatggtttgtgggcggaagctgctnntgccgcctgctatttgattaatcgctctcccaattcagctattgattttaaaattccagaagaggtatggacaggtaaaccagttgattattctaatcttagaatctttggttgtcctgcttatgctcatgttaacaatgggaagctagtgcctagagctcaaaaatgcacttttattggctatggttctggtgtcaaaggatatcgtttattgtgtgctgattctaagaaggtgattgtcagtcgtgatgttacttttgatgaaagtacattttcttcctctggaggtgtttctgatagtggttcctcatccacatctatgcctgaaactactgatgagaatcttgaggtagacgtacctattaatgttgattctgttactcctattacacagtcagtttcagtttctgtacctcctgttagcaacaatcactctattgctcaagatagacctagaaggaatattgttttgccacatcgatatcgtgacactgatagtatggctcattatgctctcattgctgctcaagaaactaatgttgcatttgaaccttcttcttattcggaagctatctcatgtgataattcatccaagtggttagttgctatgaatgatgaatttgagagtcttcaaaagaattctacatggaaattggttgagcttcctgaaggtaaaaaacctttaaagtgcaagtggatttataagaagaaggaaggtatatctggagttgaacctgcaagatttaaagctcgtttagttgtgaaaggtttcgaacaaagggaaggtattgatttcaatgaggtgttctctcctgttgttcgtcacacttctattcgagttatgcttgctattgttgctttatttgatttggagttagagcaacttgatgttaaaactgcatttcttcatggagatttagatgaggagatttatatgacacaacctcagggtttttctgctcctggtcaagagcatttagtttgtcatttgcagaaatctctttatggtttgaaacaagctcctaggcagtggtataagaggtttgactcgttcatgcttgcacatggttattctcgaagcaattatgatcattgtatttatttgaagcagttccctaatggatcttttgtgtatctactgctttatgttgatgatatgttgattgcttctcatgacaagtcactgattgttgaattgaaggctcaacttagtcatgaatttgatatgaaagatcttggaccagcaaagaagattcttgggatggaaattcagcgtgatcgtcgtgctggtactctttttctatctcagaaaagttatattgagaaagttcttgaaaagtataatctcagtaattgcaaatctgttgctacaccatttgcttcacattttaaattgtcatcgaggcaatgtcctgttactgaagatgagaaagaacacatgtctcacataccgtattctaatgctgttgggaatctcatgtatgctatgatctgtacaagacccgatttggctcatgctgttagtgtggttagcagatttatgcacaatcctggtaaagaacattggaatgcagtaaagtggattcttcgctacttgaaaggtacttctcattttggattattgtttgacaagaattcagtgaaggaaattggtgttatgggatttgttgagggatttgttgattctgactttgctggtgatcttgataagagacgctccatatctggctatgtattttcattatgcggttctgcagtgagttggagggcttcacttcagtctgttactgctctttccactactgaagcagaatatgtctcagctactgaaggggttaaagaagctatttggatgcgaggtcttatttctgaacttggtgttcctcaggatgtcattaaggtttattgtgatagtcatagtgctatttgtctgaccaagaatgacatgtatcaattcaagaccaagcatattgatatcaagtaccacttcattcgtgatattgttgctgaaggcaagattaaagtggacaagatacatacngatgaaaatcctgcagacatgcttacaaagccattatctaataccaagttcaagcattgtcttgacttggtaggtgttcgtggagcttagcaccctagtggtgtttggggaaacagttttatagatttagcgtcaaggggagatt

>Copia-66_ZM_LTR

tgttggataagatnttttgaccctatctctatctcctttgcctatataaataggcccccttgtaccatgtacgacacaccacataatagatctgttttcccgtggttttttttctcctccacatcgggggagttttttccacgttaaactctgtgtctcctttttcccacctacgatcctaaca

>Copia-67_ZM_IN

acatggtaccagagctctaggtccagccgccgccgccaccccacccctccagccgccgccgccgcctctccatggccggccggccgccgccccctcccttctccggcgacccttccctccccttccccttcctctctcctacagggcggccctcgccgcgcccctggcgggcctctcccccgcggccggcgcgccgccgggcttcgccgcccccgcggcggtcctctcccctgcggccggcgctcctgcaggactcgccgcccccgcggcggccccctcccccgcggctggcgcgccgccgggcctcccggccaccgcggcggccctctccctctcccctgcggccggcgcgccccccgcggcggccctctcccccgcgtccggcgcgccgccgggcctcgcagcgcccgtcgcggccctcccctccgcggccagcgcgccctcggccctcgctgcgccccctgcggcccctttccccgctgtccccggccctgcacatcccttcgccgctgccgggcgccgccgggccacgccttcgtgctcgccgccgcgcttccccccactgccgagccacctccgcccgtgccgacccaaaccgccaccctcgcggcagcccctggccacgacgggtccccgccggctcctgcgctctccctgccggatgccgccctccttcgctcccagctgctggcgcccggttttccacatgtcaccacgactggcgggggccccgttcgtggctgccccctgccgacgactgtctacagccagccgtccgccatcacctcctccctgcgcgctccttcggatgacgctgctgccatcctcgccgccacccgggcggccgttacggctgctcgccagcgggcccaggacgccgcccgtgctcttgagcaggagcaggcggttgtcgacgccatcgagcgccagtacgccgagacctaccgccgtctcgctggcaagggcgtgtcggatggctcccccacgtccgctcgccacagcgccgacaccttcgagcccgcgcccgccccggcctcaacccttcgcgcctctcttcatgctcaggcggcggccctcaccagcatccgggccaccgtcaccgacgtcctcgcgccggactccactcagtacccccggtggcgcgaccaggtcctgcagaccctccgccgctacgccctcgccgaccacgtcctctcagcggtccctgacccgtcggaggattggctcctgatggacgaggtggtgctctcctggatccatggcaccctcacggccgaacttcaggacatcgttcgcgtgccggacgacactgctcaccgggtctggggtgcccttgaggctcagttcctcggcaaccgccagactcggattctgtacctcgagaccgccttccgccagcttgctcagggcgatctctctgtggacgagtactgccgtcagatgaagacgatggcggacactctccgcaccctcggggcccccatcaccgacgagagcctcgtgctcaacctcctccgcggcttgagccctcgcttcgatcgcgtgacgcccatcctcacccgcatgaagccttttcccacctttgcggaggccaagaacgatctgcttctcgaggagcttcgcctctccgccactgcgaccgccgctcccgccacggcgctctacagcgctcctcgggctgccccctctgcctccgggggggtcctcttcaccgcactccggccccccgccgtctggagctcttcggcagcctgctggctccgggggggtcgcagtcgtggtcgcggccgcggtcgcaagagtggccgcggcggccggggccgttccccgggtggctcccagtggccatccttctacaacccgtggactggcaccatccacatgtggcccgggccgtccgcgggcgcctcggcccctcgccccgccgcccctcagcaggtcttctttgccgctccacctccggcggcgccctcggcacctccccagcctcagcaggggctccttcccctcccggggcctccggccccacccgtgtgggggccctggaccaatgggtgggacacgcagtctctcgccagctccttctccacgatgacgctggccccacccacctcggtctctgactgggtggctgactccggtgcctcctaccacaccaccccggacgcaggtattctgtcttccacctctcccccccacccctcccttccgtcctccatcgttgttggaaacggctctgcccttcccgtcacctcagtaggtgacgcggtccttcccggtcctttccgtttaaccaacgtccttgttgccccccacatcattcaaaatcttctttccgtccgtcagttcactactgacaactcttgttccatggagtttgacccgtttggtctttctgtgaaggatcttgccaccaggacccttctcgctcggtgtgacagccccgggcccctctacacgcttcgcctgcctgcttccactacatcgacctctgccccgcctgttcttgcagcggccgcctcttctgtgacttggcatcgtcgtctcggccaccccgggcgtgatgtgatgtccaagctctccagcagcacctccgtttctggttgtaggggatcttttgagcacctctgtcatgcttgtcagttaggtcgccatgttaggctcccattccccacctcctcttctagagcagcaggcatttttgatctgatacactgtgatgtatggacatcccctgtaatcagcatttctggctataagtattatttactcattctcgatgacttctcgcattatctgtggacttttcctctacgtcagaagtcggacacttttcccaccctgtctcacttcttcgcttgggtctccactcagttcggtcgcaccatccggagcatccagtgtgacaacgggcgcgagttctataacaatgcgtcccgtgacttcttcctctctcgcggcgtccacctccgcatgtcstgcccctacacgtctcctcagaacggccgggctgagcgcatgattcgcacgacgaacgatgtcgtgcgctctctcctcttccaggcttcccttcccgctcactactgggctgaggccctcgccactgccacctacctcctcaaccgtctccccaccaaggcggttgcccaccccaccccttacttcgctctcttcggcatccacccctcctacgaccatcttcgtgtcttcgggtgcgcttgctaccccaatctcgcctccactgctcctcataagctcgcccctcgctccactcgctgtgtcttcctcggctactcccccgaccataaggggtatcggtgtcttgacctcgcctcccacagagtcctcatctctcgtcacgtcgttttcgacgagtcggatttccccttttcctcctcccccactgcctctctcaccgacctcgacgtgttcctcgacctcgaccctgtgtcccccactgtcgtgcctcccttccccgcaggtccgtccactgcaccgcctcgcgcggccccgcctgcgccgccccccccgcactgccacgtgcggccccgacgccctctgcgccgccctcccccgcacagccacgtgcggcctcggcgtcccckgagtcgcctcgcgcggccccgcctgcgccgccctcccccgcacagccacgtgcggcctcggcgtcccctgcggccccgtctgccccgccctcccccgcacagccacgtgcggcctcggcgtcccctcgtctcccgcacagcccagtgcgacaccggcgcctgccccgccgtctcgctacaccgaccccatccagacgtaccagcgccgtggtcgacgcggcgccccggctcgctctcgcgccgagcccccggcctatcaccccgtcgtcgtccaccgggatcctcgacacatccacccgatggtcacccggcgcgcggccggtgtcctccggccacctgatcgcctgattctctccgcgacctcttctccggctcttccgccggtgcccacgactgtccgtggtgcgcttgccgacccacagtggcggcgcgccatggaagcggagtacgaggccctccaggccaaccacacctgggacctggtgcctcctccccctggcgccaacgtggtcaccgggaagtggatcttcaagctgaagctgcacgccgacgggtccctggagcggtacaaggcccgctatgtgctccggggcttcacccagcgccccggggtcgactacgacgagaccttcagtccggtggtcaagcctgccaccgtccggactgttctgactctggctctgtcccgggactggccggtccaccaactcgacgtgacgaacgccttcctccacggcaccctgacggagacggtctactgcacccagcccgttgggtttgtcgaccctgctcaccccgacatggtctgcaagctcaacaagtccctctacggcctcaagcaggccccccgggcttggtacagtcgcttcgccaccttcctgtgttcgcagggtttcgtcgaggccaagtcggacacgtccctgttcatcctccgtcgcggtccggacaccgcgtacctcctcctctacgtcgacgacatcgtgctcaccgcctcctcccctgggctcctgcgtcgcatcatctcctgcctccagcgggagtttgcgatgaaggacctcggggcgctccaccacttcctgggggtcaccgtcgagcgccgtccccagggcatgttcctccaccagcggcagtacaccgtcgacctcctcgagcgcgctggcatggctgagtgcaagccctgcgcgaccccagtggacacgcagggcaagctctccgccgccggccccccggtcgccgatccgaccggctaccggagccttgccggggcccttcagtacctcatcttcaccaggcccgacatcgcctacgccgtccagcaggtgtgccttcacatgcacgaccctcgggagccgcatctcaccgcgctgaagcggatcctgcggtacttgcgkggcaccctcgacttcgggctccttctacgccggtcctcgaccacggagcttcgcgtctacaccgatgccgactgggcgggctgtcccgacacgcgccggtccacctcgggctacgccgtgttcctcggcgacagcctcgtctcctggtcgtcgaagcgccagccggtcgtctcccgctccagcgccgaggccgagtaccgcgccgtggccaacggcgtggctgaggcggcctggctccgtcagctgctccamgagctccacagtccgctggccacgagcaccctggtcttctgcgacaacgtcagcgccgtctacctctccaccaaccccgtccaacaccagcgcacgaagcatgtcgagatcgacctccacttcgtccgcgaacgggtcgcctgtggagctgttcgcgtccttcacgtcccgaccacctcccagttcgccgacgtcttcaccaaggggcttccgtcggtggtgttcgcggagtttcggtccagtctcaacatctgcagtggctagagtttcgactgcgggggggg

>Copia-67_ZM_LTR

tgttagctatctgtgtacatgggcctagcccacagcccactaggggtatatatctgttagggttagggtttactgtagcgactactgttcattcgctctcca

>Copia-68_ZM_IN

acacgtcatcagcactgctctaccccgactcgtggatccgatcggcaccgactctccatcacgccggcaggtcctccggccttgccgaactgtcctacgcgacaggcttcgctcccgttcgtcgaacagaaacgcggtatttaagtggtcatgtttttaatacaatcacaatgatttcgtcgaaccataaagataacgtgaccctagcatacgatccgaattcttttacttccgttatgattgtcgcacttggataaacttccggtgaatttattagtattgtacagtgcagacttatttttgtatgcattatttatcccgtgttttcctgctgaaaatagataatgcgagcttaatttttttttacatacaaccatttaaatttctgttatggataattggcacgcctaattaacctggagttaattttgcgtgacaggctatggctgaaggcagcaagactgacttcgcggagcttgccctgaatggcaagaactacctgacctgggctgacgactgccagtttcatctggaggcgatgcagccgggaaaggcgatcgttcggctaggcccaaacgacattggcctgcagcttcatgagaaggcaaaagctgcgatcttcctcaggcgccacatccatccagacctgaagatggagtatctggaggtgaaagaccctctagtactgtggacgaagctgcgggagcgcttcggcgtgcagaagcatgtgatgcttccacgggcgcaacaggaatgggccacactccgcttcctcgacttcaagactgtggaggcttacaacactgccattcatcgcattgtcgctcagctacgtttctgtggccagatagtcacagacctggagatgattgagaaaactctccagacctttcacccctccaacatggtgcttcagcagcagtaccgaaacgacaagtacgcgaagtactgtgagctcgtcaacatgttgcttggcgcagaggctcagaatgagctcctgatgcaaaactaccagaagcgtcctgtcggcgctgcggccgtaccagaagcacatgccaacttcccgtctcaggggaagagaggctcctccagaggaaggggtcgtgggcgccgcaataaccaggggccgacgaggggaacttttaagaagcatgtcacggctaatggcagcggcagtagcagcaacaatggtcgtggcagaggcagaggcaaaggcaaaagcaacccccagcagggcgatgcaagtgcctcaaagcatgctggcgaaggatgtttcaggtgcgggtctcagcagcactggtcccgtacatgcaccactgagaaatacctgattgacatatatcaggaatggaagaaacgccagaactcagaggctcactttatccaggcgcctgtagacgccacaactggagagcaccttgagttgcctcagcctgctgcacagtttgagcatgctgccataaatgttgatgccacaactaagagcgatactccgttaggcaaagtcgacttcgatttcgatactgatgatctcctgtagttgatcacgttatcagccattagctccacatttatttccatagcaattttatttctagaagaacatttatttggttatgatgtaataaatgctcctgctgagcccatactatttggttgattgagattaattgctttgcaattttatttattcctcattcatggtttaattgttcttgctctattatatgttgtcatcatgaatgatgaactaaatcaatattatatattatttatatagaacaagactcacttcccacgatggaggaaaacgatgaaccatgcctgattgatagcgcagccacaaattcgattcttagagagacgaaatattttcagacactcctgaagaggactgagaatatcactacaattgtcggcaacaatggccgcattgtaggctccggtcgagccattgttgtcctccctaacggtactaggatcttcattgaagaagcattcctatacccaggagcaactcgtactctcctcacgttcaaagatatccgtcgtagcggctatcacgttacaactgcatgtgtgggtggtgctgaatacctccatatcacatcaacaaacgaatgtgaaacaaaagtggtagagaaagcgcaaggcacctcctctgggttgtattactcaagaattaaaccaccccctgaatttgttgcgatgtctactatatttaaaaatcctgagtcttttcgagtatggcatgaaagactaggacaccctggattaaggatgatgcggaacatcataactagctctattggccatggcatgaaaaccacacaaattcctaaagacttcctatgcgtctcttgtgctaaaggaaaactgataactaagccctcttacctgaaagtaaaggctgagtccccgagtttcctgcagagacttcagggtgatatctgtggtccaatcaaccccctatcagggcccttcagatattttatggttcttatagatgcttctactaagtggagccacgtgtgcctgttgtccacgcggaaccacgcatttgcaaaatttattgcccaaattatccgtttgcgtgcgagcttcccggagaaccgcattcagtcaatcaggatggataatgccggagagtttacctcaaaggcctttaatgactattgtcttgctttgggcataaatgtagagcattctgtaccgcatgtccatacacagaatggacttgctgagtccttaattaaaagaataaaattcatagccaggccacttctacaagatagcaaactcccgaccagttgttggggacacgcagttttgcacgctgcggccttaatccaatatagaccatctgcttaccactctgcttctccccaccagttaacgcgtggtcaacaaccagtggtttcccatctgcgaaaattcggctgtgctgtatacgtgccgatatcaccaccccagcgtacatctatgggacctcaccggaagttggggatctatgttggttatgaatctctgtccataatcaaatatttagagcctagaaccggggatctgttcacggctcggtacgctgatagcatcttcgatgaagagcatttcccggcattagggggaggattgtaccttaataacaaagaatgccgagaaatagaatggagtgctagtagcatccaatcgcttgacccacgcactagagaaaccgaacttgaagttcaacgaataatacacctgcaaaacttagcaaataatctgccagatgcctttactgatataagaggagtgtcgaagtcgcatattcctgcggcaaatgcaccggagagagtggagatacctctgcagggtatggactctactcgtactccccatcctaggaaaagggggagaaaaccggatgattcagtatcagctaagcggggacgcctgcaccagcaagttactgaagagaatgcaacgcgctccctttcagatgttccagcagtaacacaccttgaaggtgaacgtcctagtgcaaatgtgcgcacaaataaaagcacaaggacttcggaacaaccggtttcaaataacttgggaaatcacaaagaaccggatgattcagttgaagaaattgccataaactatgttgagacgggagagttatataaccgaaagactacagttgtcgacactgatttcgtctctatgatcgctgttgtgattgctgaggatcctgaaccaaagtccatggcagagtgccagaagcgctcagattgggtcaaatggaaagaagcaatagagacggaattgctctcgctttcaaagaggcaagtatttgggcctgtcgcccgcacgccccccaatgttaccccagtcgggttcaaatgggtgttcgtccgaaagagggatgcgaacaatgaggtggtgagatacaaagcgagacttgtagcacaagggttcacgcagagacccggcatcgattacgatgaaacttactccccggtaatgagtggcacaacgttccgatatttgatatcactggcagcaggtttaaacttaaaaatgcagatgatggacgtagtgaccgcatacttgtatgggtcattggattcggaaatctatatgaaggtcccggatgggctgagagttccggatgccaagtcaaatcgcaacatgtacagcgttaggttgcagagggcgctgtatgggcttaaacaatctgggcggatgtggtacaatcggctcagcgaattcctcctgaagaagggatacgtaaataatccagattctccttgcgtattcattagaaaatcccagaagggtttttgcatagtttcggtctatgttgatgacttgaatatcattggatatgctgaggatattgaggaggccagcgcctacctcaagacagagtttgaaatgaaagacttgggagaaaccaagttttgcttgggcctgcagatcgagcatctcccagaaggaatatttgtacaccagtcgacctattgtaaaaaggtccttgagagatttaatatgattaaagctcaccctttaaagaccccgatggtcgtgagatcccttgaaatggataccgacccatttagaccaaagagtgatgatgagaaatcgttgggacctgaagttccttaccttagcgctattggagcactgatgtaccttgcgaattgcaccaggcctgatattgcattcgcggtaaatctactagctagatacagtgcagacccaaccagaagacattgggtaggtgttaagactatacttagatacctcaaaggcactcaagatcttggcctgttttttccgaagaatcaggaccagactatggtgggctatgctgatgctggatatcagtcagatccccataatggtatatctcagactggctttgtatttctatgtggtggcactgctgtatcctggagatcgtgcaaacagacattagtaacgacgtctactaaccactcagaaataatctcactatatgaagccgcacgagaatgtgcttggctacgacggatgactaactacatccagaagtcatgtggttataacaccgcaaatacccccaccattatctatgaagataatgctgcatgtgttgcacaaatggaaacgggctatattaagagcaatatgactaagcatatctgtcctaagtatttttatcctcatgaactccagaacgagggtgaggtaaaaatcctgcaagtcaagtcttgtgataatctggctgatttattcactaaatccttacctgcagtcacattcagaaaatgtgtacgaggtatagggatgaggcatttgagagacctgcagggttcagggggagatcacctctgaatttcagcccttacaattcgacctgaagatcgtatattatacaccttgaaggttatagttgtcctgaagacacccgttgtactctttttcccattgtgagttttcctgaagtttctcacagatggtttttaacgaggcaacaagtgcaatacatcgagtggcactatgctctctttctccattttttcccactgggtttttcggagttttgatgagacataagtcgtcacggtattcgcccaagggggagtgt

>Copia-68_ZM_LTR

tgagaaaccctaaaaggggtttaatgggctaataccgaaactgcccttaggggtacacaggttatatatatgtacccatacccatcataataaagatagacagaaagagcttccatccactgtgtctcttgttcctctctgtgttgggttgctgggaagggagcgggcttctacaacttctcacgtccctactgctgttgggagggttcggagtccggatcggggatctgcgacaactacgttattca

>Copia-69_ZM_IN

tggtatcaaagagccaattagatccggcttattagccgctgatcccggttcctagttgggagaccgccgcggttccgtcgtcgcggttcgtcggccgccctcgcggttcctcgcgcgtctcgtcgtcctcgcggttcgtcagccgccgtcgcggttcatcggcagccgtcgcgtttcctcgtcaggcgtcggccgccgtcttctctgcgctgctgtcttctctgcgccaagcacctcggccgcgccctaccctgctggatccgcgcccctgcagccacgccctgctggagccgcgccctgctgcagccgtgcactgcagatctcgcgcccctgcagccttccctgcaaccgctggatcctgcgctccggccatcgtccagactgtccagccctctgctcctgtccgcatcaagctgaggtccagccctctgctcctgtctgcatcaagctcctcctctggtccgttttgattggaacaacattcagtgctagtataccatgtcgcacactactgcaattattatcaatatcactttggacggtcaaaattaccgtgaatggtccttctgtgtagaaactgctttacgaggtcatggccttgcttttcatttgatcgatgatccacctttgctcactgcaaataacagtaatgcttctgaaatcaagacttggacaattaacgatggaaaagtgatggctgctattgttaacagtgtcaagccatccatgattatgagtctttcttcattcaagactgcaaaagctatgtggttttatttacagaagcgttatgttcaagatagtggtgctcttcttcatactcttatgcagaaaatacatttaatagagcagcatgatttgtccattgatgagtattattcagcttttgatcgtctgatgggtcctttgatgtctatggttccttcatgcaccgctgatgagtgcactgcccataagttcattgaaaaattcttcacatacagatttgttatgggtctcaggacagattttgaggctattcgtaccaggctgcttcatggatctgcaagtctgaccatgacagaggccttatctgatttgcttgctgaggagactcgcctctcatctatgtctatacctcatactcctgtgtcacataatatgttagcatcttctcacaagtatggtggaaaaggtggttactttgagccttgcaagcattgcaacaagactacacattggtctgatcaatgttttcttaagtatccagagaagttagctgagtttcgttctcgccgtactaatcaagggcgtgggccatccaagggttcagtgtcagttgctgctctctctggtaatgcttcttcgtccacatgggtacttgattctggtgcctcatttcatgtaacttcagatcagtctcagctggatacttgtgcaacagtcactaatgggtcttctgttcaaacggctgatggtacttcctgctctgttactcacaagggctccctctgtacctcccagttttctatacctgatatttcttttgtacctgagctctctatgaatttactttctgttggtcagattacagaccacaattgttttgttggttttgacagctcatcttgttttgttcaggatcgtcgaagcgggactgtgattgggactggccatcgccgtagagactcttccagactctatactctggactctttacgtctacctcctgcctacactgccagtgtttcatccgtcgtgtccacttcatcttttgctcaatggcatcatcgtcttggccatttgtgtgggtctaggttatctactctagttcaaaaaggatgtttaggtcataccaacattgagtcaaattttcattgtaagggttgtaagcttggtaaacaaatacaacttccatatttatctagtgcttcttactccactcgcccctttcaacttattcattctgatgtatggggtcctgctccttttcccacaaaaggaggtcataagtactatgtcatttttatagatgatcattccagatatacttggtcttttttcatgaaacatcgttctgagctatattctatttatcagtctttcacacggatgatacatactcaattctctagctcaattaaaatttttcgctctgactctggtggtgaatacttgtctgataggtttcgtcggttcttgacatccgagggtactcttgcccaactctcatgtcctggagctcatgctcagaatggtgtggctgaacgaaagcatcgccatattatagaaacagctcgcacccttcttattgcttcttttgttccatctcacttctggggtgaagctgtgtctactgctatttatctaatcaacagacaaccatcaactaaattagccaacaaatgcccgggtgaggtactttttggtactcctcctagctacgaccatcttcgtgtttttggttgcacctgttatgttctcctagcaccccgcgagcgtaccaaattaactgctcaatctgttgagtgtgtctttttaggatatagttctgagcataagggctatagatgttatgattcttcagctcgccgcatgcgtatttcacgtgatgtggtatttgttgaaaatcgtcctttcttttacaattcttctacttcctcttcgtattctcctttacagaccacatctttattatcctttccttctaataatgacaacgttctatccccacacttgcctgatccccttttgtctatatctccaccagagacatcttcaccaccagctttgtcagatcgactacctattactcgtgtctatactcgtcgtcccactaaccctccacctgtacctgttccgtcgcctctggtcagtcctgatactcctgtttttgatgatactaacaattctgatgagttgcaggttgcccagagatataatttacgtgatcgtactacaatagcacctccagatcgttatcgttatccatgttctgctgtcgtaattgttgaacctactacttatcaggaagctgctggtattcacgagtggcaacttgctatgattgaggagcttgctacacttgaccgcaccggtacttgggatattgttcctttaccgtcacatgctgtaccgataacatgcaaatgggtcttcaaagtcaagaccaaatcagatggttctgttgagcgatacaaggctcgtcttgttgctagaggttttcagcaaagtcagggtcgagattatgatgagacttttgctcctgttgcccacatgactacagttcgtactatgattgccgtagccgctactcgttcttggaaaattcaccagatggatgttaagaatgcttttctccatggtaatttaaatgaagaagtttatatgaagccacctccaggtgttgaggttccctctggttctgtttgtcgccttcggcgtgccttatatggtctcaaacaagctccccgtgcttggtttgagtgttttgcttctgctattatggctgctggtttctcacctagtaatcatgatcctgctctttttgttcatcaatccacacaaggacgtactttacttctactctatgtcgatgatatgttgattactggtgacaatgaggaacacatttcctctgtgaaaaagcaacttggaaaattgttcatgatgactgatttaggtcccctaagctattttcttggcattgaagtagtgcactccaccaaaggttactacctttcacaacacaaatatattcaagatcttcttgcacgatctggtattactgatactcggacagctgccacccctatggatcttcacttacagctccgctcatctgagggcacacctcttgccgatccttctcggtatcgtcgtattgtgggaagtctggtctatcttactgtgacgagacctgacatagctcatgcagtacatatcttgagtcagtttgtgagtactccaactttggtgcattatggacatttgcttcgtgtcctacgctacttacgaggaacatccactaggtgtcttttttatgctcaaagcagtccgcttcagctttatgcttattctgatgcaacatgggccagtgatgtgaatgatcgatgctctgtcacgggctattgcatttttcttggttcctctcttattgcatggaaatctaagaaacaagttgctgtttctcgttccagtaatgaagcagaacttcgtgcaattgccaccactactgcagaaattgtctagcttcgttggttactggctgatttcggtgttgtgtgtgatggctctacacctcttctgtgtgataatactggtgctatacagattgctaatgatccgatgaaacatgaccttcaaaagcacattggtgtagatgccttctttactcgatctcattgccgtcagaagacaattactctacaatatgtaccttcagatttgcaagtggcagatttcttcactaaagctcaaactcgggagcatcatcgactacatttactcaaactcaatgtctctgatccgccttgagtttgagggggg

>Copia-69_ZM_LTR

tgttaaggcccatattggcccatatacctatctatatatatgagagagtcatcaggagacgttatcattgtcattatcattccaaatctctcggtacgtaaca

>Copia-70_ZM_IN

tggtatcagagcaggttacgattagggtttttcctaattttttagccgccgtcgcggccttcccatcctcagccgccggccgccgtcgcggccttcccatcctcagccgccggccgccgtcgcggcctccctatcctcagctgccgtccgccgtcgcggccttccatcctcagccgccgtcgcggtccccccaccctccgctgcgtcgcggcgccgccgtccctgcgcggatctccgtcccgcgctgcggcccgcgcggatctccgccctgcgctgcgccgtcggctgcggatccgtcgcggccacccatcctccgctgcagtcgcggcgccgccgtctctgcgcggatctccgtcccgcgttgcggcccgcgcggatcttcgccctgcgctgcgccgtcggctgctgcggcctgctgtcgcggccacccggtgccgccgctcctgcgcgaactccgccccgcgctgcgccgtcggcttctgcggcggctcctgcgcggactccgccccgcgctgcgccgttggcttctgcggcgccatctcctgcacggatctccgccccgctgctctgcgtcatccatctgcgctggtcgcctgcaccgtcgtggatctgccccgccactctgcgcggatctctgcccctgcgcggatccttctttctgcgcggatctctgcccgtctgctgcgccgttgctgtcgctgcagtctcctgcgcgctgtccccgcaggctcctgcacggatccgtccggactcctgcgtgccatcagccgcacacaggcctgtgcactgtctgcagcgccggtcagatctgctgctgcagccccggccagatctgctgcagcagcccccgtcagatctgctgctgtcctgtgacttcgcacagtctcggactctcggtaccatcaagccagttttttttcagatctgctaatcttcagcattttattgctmttcactatgtcggccaacgctattgttgtcaatattgtgcttgatgggcagaattatccagaatgggctttttgtgtccagactgcattaagaggtcacggattactatttcatttgactgaagattctccagttttagcagctgatagaagcaatgctgctgcaatcaagacttggcagataaatgatggcaaggtaatggctgcaatggtcaacagcactaaaccaactatgattatgagtctgtccaaatttaccactgctaaggctatctggtcacatctgaaggatcgatttgttcaggatagtggtgctcttctacatacccttatgcagcagactcatgtgattgagcaacatgatatgtccattgatgaatattattcagcctttgatcggmttatgagtgcattgacctccatggtgcccgcttgtaccgctgtgccttgtccagcccacaagttcattgagaagttctttacctacagatttgttatgggagttcgacctgaatttgattcccttcgtgcaaggctscttcacagttcagatactctcaccatggctcaggcwttgtccgaattgcttgctgaagagactcgtctgaagtctatgtcctctattactggtgtgagttctcatagtgtgttggctgctgctcagaggtctaggaacacctcctttcmgccttgtgaacactgcaaaaagaccactcatcggtctgagaactgctttgctaagtttccagagaagctggctgattttcgtgtccgtcgtgctactcgtggtcgtggtacaggaccatctcctagaggctcagttgcggttgctgctacttcatctgctggtgctttgtcatcatcttgggttcttgattctggagcttcctttcatgtgacatctgatcagtcacggctggcgtctactacacctgtcaccgagggtacttctgttcagactgctgatggtacattatgtcatgtcacccacaagggttctctttctgattcaacttttactgtcccaaatatattttttgtacctcagttatccatggatctactatcagttggtcaaattaccgatcacaattgttttgttggatttgatgactcatcttgttttgtacaggatcgcaaaacaggggatgtgattgggactggccgtcgccgtaaatcttcgcctcgcctctacatcttggacactttgcgtcttccttcatcccctaccactacacctcatgtgctcgctgcttcgggccccacgacgtcattcgcccagtggcatcatcgtctgggtcacttatgtggatcccgtctgtctactctaataaagtcaggatgtttaggctctactcatgttgagtctagttttcattgtaaaggttgtcatcttggaaaacaaatacaacttccatattttactagtaattcccattctgctaaaccttttgatctaattcactctgatgtttggggtccagctccgtttgtttctaaaggtggtcataaatattatgtcatttttattgatgatcattctcgttatacttggatttacttcatgaaacgccgctcggaattgccttctatttacaagtccttcactcgcatggttcacacccagttttctgctactattaaaatttttcgttcagattctggtggtgaattcctatctgataattttcgccagatattgaccatagagggtactctagctcaactttcttgtcctggtgctcatgcccaaaatggtgttgctgaacgcaaacaccgtcatattattgagagtgctcgtactcttttgatatcttcttttgtgccttcacacttttggagtgaagctgtttccactgccgtgtatctcattaatagacaaccttcatctaaactttctggtaaatcaccgggtgaagttctttttgggacttctccacgatatgatcatcttcgtgtgtttggatgtatatgctatgtcttgttaccaccacgtgagcggactaaattgactgctcaatctgtagagtgtgtattccttggatacagtcctgaacacaaaggctatcgttgttatgatccatctactcgtcgtattcggatttctagagatgtgagtttcaatgaaaatcgccccttctttcacaaccagtctactcactccacctattatcccacagaatctacttctttcatgtgtcttccttccattcctgcatctgaaccatcatcttctacatccacatctgatgttctcattcccataacaccaccttccacctccacgtcatccacctctttactcttccaaaccacctatcatccaaacctacattcgtcgttcccgctctatccctacggctggtcctgatactgatcctgtacctgattcttgtactaacaactctgagtctaatgatgtttttaatcagggatatcgtcttcgtgatcgtggtactattgaacctccagatcgttatggtttcccacggactgctgtggcaattgttgaacccactacttatcaggaagcttctgggattcttgagtggcagctagctatgattgatgaattggctgctcttgaacgcactggtacatgggatattgttcctttaccctcacatgttgttcctatcacatgtaaatgggtcttcaaagttaagacaaaatccgatggttccattgagaggtataaagctcgtcttgtggctcgtggtttccaacagactcaaggacttgattatgatgagacttttgcacctgttgcgcatatgactactgttcgaaccttgattgcagttgcagcctcatcttcttggactatctcccagatggatgtcaagaatgcttttctcaatggtgatctacatgaagaagtttatatgcatcctcctccgggggttgatactccatcagggcacgtttgtcgtctccgtcgcgcattgtatgggctgaaacaagctcctcgtgcttggtttgagagattcatctctgtcatcacagctgctggtttttcttctagtgaacatgatcctgcattgtttgttcatgtctctccaaaaggccgcactttacttctattatatgtggatgatatgctgattacaggtgataactcagaacatatttctcatgtcaagcagcatcttagtaaggaatttcagatgtctgatttgggtccacttagctatttcttgggcattgaagttcagcagactccaaaaggtttttacctgtctcagtccaagtatatacaagatcttcttgatcgctctggaattactgatactcgcacagctgcaacaccaatggatattcatttaaaacttcgtccgaaggatgggacaccactagcagaccccactcgatatcgtcatattgtgggtagtcttgtttatctcaccatcaccagacctgatattgctcatgctgttcatatgttgagccaatttgtatccacaccaacttcagttcattatggtcatttgcttcgtgtgcttcgatatttacgtgggacacgatccagatgtttactctatgcttctgatagcccacttcggctacatgcgtattctgatgccacttgggcaagtgatcctgttgatcgttgttctactacaggttactgtattcttcttggatcctctcctattgcttggaaatccaagaagcaagcggccatatctcgttccagtgctgaagccgaacttcgtgcccttgctactaccactgctgaaattatttggctccgctggctattggctgatcttggtgtctcttgtgacactcctacacctctgttatgtgataacttgagtgctatacagatttctcatgatccagtgaagcatgaacttactaaacacattggtgttgatgcatcatttactcggtctcattgtcagcagaagactattgatcttcaatatgtgccctcagaatctcaattggcagatttcttcaccaaagcacaaacaagagcacaacatcagttccacttgatcaaactcaatgcttcagatcctccatttccacattgagtttgaggagggg

>Copia-70_ZM_LTR

tgttaaggcccattaggcccaggtctactgcctatatatatatattatatagtccttagggactaatcatcaattgtcaatcctaaggttagtaaca

>Copia-71_ZM_IN

attggtatcagagccagtgcttcattaagagtctaacaaactcgaagtgatgtctggagatcacgccaagagggagatcgtcaccggcgacaagcccgcaggctcggggaggactctatcaagggagtccggcgacaagtacaaggaggaatcctcttcctccatcaagtcacataggaggggtgacaagaagaaaaagaaaatgaagaaggtggtctactacgagaccgactcttcatcaccctccacatccggcgccgagtcgtccactacttcgaagcgccatgagcgcaagaagtatagtaagatgcccctacgctatcctcgcatttcaaagcgcgctcctctactttccgtacccctaggcaaaccaccatattttgatggtgaagattattgcatgtggagtgataaaatgaggcaccatctaacctcactccacgaaagcatttgggatattgttgagtttggagcgcaggcaccacaggtgggcgacgaggactacgactcggacgaggccgcccaaattaggcactttaactcccaagcaacttctatactcctcgcctccttgtgtcgagaggagtataataaggtgcaagggttgaagaatgccaaagaaatttgggacgtcctcaaaacggcgcacgaaggggacgaggtgaccaagatcaccaagcgcgagacgatcgagggagagctcggtcgattcgtcctcaacaaaggagaggagccgcaagcaatgtacaaccggctcaagacgatggtcaaccaagtgcgcaacctcgggagcaccaaatgggatgaccatgaaatggtcaaggttattctaagatccctcgtttttcgtaatcctactcaagttcaattaatccgtggagatcctagatataaacagatgtctcccgaggaggtgattggcaagtttgtgagctttgaacttatgatcaaagactccaaacacattgtcaacttggagcaaggcgccacctccacacccgaggtgcaacccgttgcattcaaagcaacggaagaaaagaaggaggagtctacaccaagtaggcttccaatcgacgcctccaagctcgacaacgaggagatggctctcatcattaagagctttcgccaaatcctcaagcaaaggaaggggaaggactacaagccccgctccaaaagggtgtgctacmggtgtggtaagtccggtcactttatcgctaaatgtccatatacaagtgacagtgacagggacgacgacaagaaggggaagaagaagatggagaagaagaggtactacaagaagaagggtggcgaggcgcacatggggcgggaatgggactccgacgagagctccaccgactcctcctccgacgaggacgccgccaacatcgccgtcaacaagggcctcctcttccccaacgtcggccacaagtgtctcatggctaaggacggcaagaagaagaaggtacattctagagatacccccaaatatactacatccgatgatgagggtagctctagtgataatgaagatgatttaatttctctctttgccaaccttaccatggaccaaaagaaaaaattaaatgaattaatagaaaccattaacgagaaggatgatctcttggaatgccaagaggacttgctcgttaaggaaaacaaaaaatttgttaaattgaaaaatgcttatgctctagaagtagaaaaatgtgaaaacttatctaaagagcttagcatgtgcaatgattcaatttcctgtcttagagatgagaatgctagtttaaatgctaagattgaagaattgaatgtttgcaaaccatctacatctactgttgagcatgtcactatttgcactagatgtagagacgttaatgttgaagctattgatgatcaccttgccatgattaaagaacaaaatgatcacatagctaaattaaatgctaaaattgccgagcatgagctagaaaatgaaaagtttaaatttgctmgaagcatgctctataatgggagacgccctggcattaaggatggcattggcttccaacaagggagccaaagcaacgccaagcttaatgcccccaagaaattgtctaattttgttaagggtaaggctcccatggttcaggatagagagggttacattttatatcctgcaaactatcctgagcataagattaggaaaattcatgctagaaaacctcataccgtttctcatcatgcttttatgtataaaaatgaggcttctagctctaggcattccactcatatcaaaatgcctaaaaagaaaattcctaatgcatcaaatgagcataacatttcatttaagacttttgatgcttcttatgtgcttactaacaaatcaggcaaagtagttgccaaatatgttgggggcaaacacaagagtccaaagacttgtgtttgggtacccaaggtgcttgtttctaatgtcaaaggacccaagaccgtttgggtacctaagaacaaggcctaaatttgttttgtaggtttatgcatccggtggatcaagttggataatcgatagcgggtgcacaaaccacatgacaggggagaaaaggatgttctcctcatatgagaaaaatgaagatccccaaagagctatcacattcggggatggaaatcaaggtttggtcaaaggtttgggtaaaattgctatatctcctgaccattccatttccaatgtttttcttgtagattctttagattacaatttgctttctgtttctcaattatgcaaaatgggctacaactgtctatttactgatgtaggtgttactgtctttagaagaagtgatgattcaatagcatttaagggagtgttagatggtcagctatatttagttgattttaatgataacaaagctgaactagacacttgcttaattgctaagactaatatgggctggctctggcatcgccgacttgcccatgttgggatgaagaatcttcacaagcttctaaagggagaacacattttaggactaacaaatgttcattttgagaaagacaggatttgtagcgcatgccaagcagggaagcaagttggagtccatcatccacacaagaacatcatgacgaccgacaggccgcttgagctactccacatggacctattcggcccgatcgcttacataagcatcggcgggagtaagtactgtcttgttattgtggatgattattctcgcttcacttgggtattctttttgcaggaaaaatctcaaacccaagagaccttaaagagattcttgagacgggctcaaaatgagttcggattgagaatcaaaaagattagaagcgacaatggaacggagttcaagaactctcaaatcgaaggatttcttgaggaggagggcatcaagcatgagttctcttctccctacacacctcaacaaaatggtgtagtggagaggaagaatagaactctattggacatggcaaggaccatgcttgatgagtacaagacaccggaccggttttgggcggaggcgattaacaccgcctgctactccatcaaccggctatatcttcaccgaatcctcaagaaaacatcatatgaactcctcactggtaaaaagcccaatgtttcttattttagagtttttgggagcaaatgctttattcttattaaaagaggtagaaattctaaatttgctcctaaagcagtagaaggctttttacttggttatgactcaaacacaagggcatatagagtcttcaacaagtccactggattagttgaagtttcttgtgacattgtgtttgatgagactaatggctcccaagtggagcaagttgatcttgatgaattagatgatgaagaggctccatgcgtcgcgctaaggaacatgtccattggggatgtgtgtccaaaagaatccgaagagcctacacaagcacaagatcaaccatcatcttccatacaagcatctccaccaactcaagatgaggacgaggctcaagaagaagaggatgaagatcaagacgatgagccacctcaagaggaggacattgatcaagggggagatgaagatgatcaagacaaggaagatgatcaagagattcgggatcaaagaccgccacacccaagagtccaccaagcaattcaaagagatcaccccgtcaactccatacttggtgacattcacaagggggtaaccactagatctcgagttgctcatttttgtgaacattactcttttgtgtcctctattgagccatacaggatagaggatgcactaagagatccggattgggtggtggcaatgcaagaggagctcaacaacttcacgaggaatgaggtatggcatttagttccacgtcctaaccaaaatgttgtaggtaccaagtgggtattccgcaacaagcaagatgagcatggtgtggtgacaaggaacaaagcccgacttgttgcaaaaggttattcacaagtcgaaggtttggattttgatgaaacctatgcacccgtagctaggcttgagtcaattcgtatattacttgcctatgctacttaccatggctttaagcttatatcaaatggacgtgaagagtgccttcctcaatggccctatcaaggaagaggtatatgttgagcaacctcccggctttgaagatagtgagtatcctaaccatgtttataagctctcaaaggcgctttatgggctcaagcaagccccaagagcatggtatgaatgcctgcgagactttcttatcactaatggcttcaaagtcggaaaagctgatcctactctcttcactaaaacaattgctaaagacttgtttatatgccaaatttatgttgatgatattatatttgggtctactaacaagtcatcttgtgaagagtttagtaggattatgatacagaaattcgagatgtctatgatgggggagttgaagtatttccttggatttcaaatcaagcaactccaagagggcaccttcatcagccaaactaagtacattcaagacatacttaagaagtttggaatgaagaatgscaaacccatcaagacacccatgggaacmaatgggcatctcgacctcgacacgggaggtaaatccgtagatcaaaaggtataccggtcgatgataggatctttactctatttatgtgcttcacgaccggatattatgctttctgtatgcatgtgtgcaaggttccaggcaaatcctaaggaagttcaccttagggccgtgaaaagaatcatgagatatttagtttacactcctaagtttgggctttggtaccccaagggatctacctttgatttaattgggtattccgatgccgattatgccggatgtaaaattgacaggaagagcacatcagggacttgtcagtttctgggaagatccctggtgtcttgggcttcaaagaaacaaaactcagtagctctttccaccgccgaagccgagtacattgctgcaggccattgttgtgcacaattactttggatgaggcaaacccttagggactatggctacaagttgagcaaagtccctctcctatgtgataatgagagtgcaatccgcatggcggataatcccgttgaacacagccgcactaagcacatagacatccggtatcactttctgagagatcaccaacaaaagggggatatcgaaattgcttatgttagcacccacaaccaattagccgatatctttaccaagccactagatgaaaagacttttagcaaacttaggaatgagctaaatatacttgattctcggaactttgattgaaacattgcacacatagctcatttatatacctttgatcatgtctctttcatttggtacaaatgcatatttcttattcttcttgtgccaaggctaagactaatgtgctttcaagtgtatttctatgattagtcttagattgaaagggaaatggagtattcggcaaagacaaggcttccactccaactctgacggtatcatttatcctttgccgttactcagaaactctcaattggtatgacccttcactcatatttattttaccaagggggagaaagtattaggccccaaagggggagaattcaaaagggctctcaagttctccgtttttggcgattaatgccaaagggggagaaagtattaagcccaaagcaaaaggaccgcaccaccacattttcaaaattcaaaaactatttcacaaaatgaagaaattatttcaattggtatcaaatttcaattggtatctcatttggtttcaaaatttcaattggtataatttcatttggtatccgtttcaaaaaccctcttgaaagctaaggggagaatttcttcagggggagcttttatttagtcaaaggaaaagcatttgaaacagggggaggaatttcaaatctcgaaaatgcttcttgaaatcatattcctatacctttggctatttgcaaaagaatttgaaaagatttttccaaaagatttgcaaaaacaagcaagtggtgcaaatgtggtccaaaatgttaaatagaagaaaagcaatcacatttattcttagacatatgtataaagtctttcaattggtttaattctaagtaacctatgcacatctatctcaattgcaaactagttacatttctacacttcatatttgctttggtttgtgttggcatcaatcaccaaaaagggggagat

>Copia-71_ZM_LTR

tgaaagggaaatggacttaatcatttcctataatcgattttggtggttgacgtccaacacaaaccacgtggactaactagtttgtctagatgtcatttatctcaggtgcataaggttcaacacaaaccaagaaagaaattcagttggggactcaataaaatttggagcaagacttggagtgtgctgccagtggcgcaccggacagtgtccggtgcaccaggccgagcaccagcgaaccggccactctcgggttttttcgggcgcgctccgctataattcaccggactgtccggtgtgcaccggacatgtccggtgagccagcggagcaacggctccctcgcgctcaacggtcgactgcgaacagtaaaagtgaacagtgcgggacagaagtcagagcgcgaagtcagagtcaccggacatgtccggtgtgccaccggactgtccggtgcagctagaggacaaaggactccaacggtcaaccgctccaaaccccaacggcgtgctgacgtggcgcgcaccggacagtgaacagtgcctgtccggtgcaccaccggactgtccggtgcgcccatcgacagccaacggctaggaagtggttggggctataaatacccccaaccacctccattcaaagccatccaagttttctgaacttcacattcaatacaagagcaaaagcattcactccaagacacattcaaaagatcaaatcctctccaagtcccaaaatcaactcaatcgcttagtgacttgagagagggtgttttgtgttcttttgttgctcttgttgcttggattgccttctccttcccattcttatttctctaagtgctttgtaaagctagcaagagacacctaagtgtgtggtggtccttgcggggtcttagtgacccatgtgattaaggagaaggctcgcccggtctaagtgaccgtttgagagagggaaagggttggaatagacccggcctttgtggcctcctcaacggggactaggttctttggaaccgaacctcggtaaaacaaatcaccgtgttcacttgcgttgatctccacttgatttgtttgccctctttcctctctctaaagtttcccttgctaatattgatttgagtttgctcccaaacgtcatccgcatcgattgagcaactcttagcaagaggaacaatcttccgcactcggatttaattctaacgctaaccccggccttagtgcgtgtttaaagtttataaatttcaggtttcgcctattcacccccctctaggcgactttca

>Copia-72_ZM_IN

tttggtaccagagccagttctccgccatcgcaatgtcgcacaagtcgccatccatgaagaggccgacggacgacagcgacggcgagaacaaggtcaggcccttgcgatcgccgccacgacgccgcggccgttcttacagccgccgccgctccggctcgcgcatcgtcgagcgcgtcatcgagcgcccgtccgcgaacgttgcatggccaatgctcacgcggacgaactactccgagtgggcgctcgtgatgcaggttaattaccagactcttcgtgtctgggacgccgtcgagatcgggatcgatgaagacgccgacgaaaacgagtatcaaaagaaccgccaagccatggcgggtctcctgcgctcggttccctccgagatgtgggcgacgctcggacgcaagcagaccgtcaaggaggcatgggacgccgtcaaggtactccggattggtgacgaccgcgcgcgcgacgccagcgcgcagcacctgcgccgcgagttcggcgcaatcgtcttcaaggaaggcgagaccgtctccgagttcggcatccgcatctcgacgctcgccaccaacctccgcgtcctcggcgacgaaatcactgacgcagaggtggtgaagaagcttctgcaggttgtgccagaacgcctgtcgcaggcggccgtttctctcgagatgttcttggatctgaacaaggcctcgatcgaggaggtcgtcgggcggctgcgcgtcttcgaagaacgcgggagacccaaggagatcaccgactccatgggacgcctcatgctttgtgaagaggattgggaggcgcgccgcaaagcccgccgggaggaggaaagctccggcggcagatcgtcttcaggcagccgcggaaggggcagaggaggcggtcgcggtcacggcggtgatggatccacgccgcgggatggccgtttcggtcagatctcgggccatggcggcggcaggccaccgcgcggtacccgttgcgacagctgcggcaaggtgggccattgggccaaggactgccgtggcaagaagaaggctgcgacccacgtcgcccaagcagaggagaacgaccgggatcacgcgctgatgtacatcgctgcggatcaggaagtgttcacgccactcgccgtgaagatgcagcggcatgtgcacatctccgaacccaaggtgctccttcacctgagccaggaacaaagggaggacgctggggtgcctcgccgctgggtgcttgacactggggcgacgaatcacatgacgggaacacgccacatcttcgccgagctcgacaccgccgtcaccggatctgtccgcttcggcgacgggttcgtggtcgccatcgaaggaaaggggacagtcctcttcgcttgcaagtccggggaacatcgccggttggaaggagtgtactacatcccgcgcctcaccaccaacatcgtgagtctcggccaaatggatgaggacggctacgcaatcaacatcgagggaggagtgctgcgcctccatgatccgcagcaccagctgctggcgaaggtgcagcgctcacccaatcgcttgtacctccttgatatgactatcgccgctccggtgtgtttcatggcgcggatcggcgacgtggcgtggcgctggcatgagcgctatggccacctcaacttccaggcgctgcgcaagctgggacgcgagaagatggtgcacgggctgccgactgtcaaccacgtcgagcaagtatgtgaaaattgtgtccttgccaagcagaagcgcgcgtcgttccccaaggtggccaaatatcgggcgcaagagcagctggaactgatccacgaagacctctgcggccctgtctcaccaccaacaccaggcgggaacgcctatttcttgctgctcgtcgacgacatgagccgctatatgtggctcaccctcctgcgttccaaggcggacgcaccggcagcgatcatgacattccaagccaaggtcgaacgggaaaccgggaagaagctgaaggtgcttcgaactgataatggaggtgaattcacttctgtgcagtttggtgaacattgtgcaggggaaggcatccagcgacacttctcggcgccaggcacgccacagcagaatggcgttgtggaacggcgaaaccagatggtcgtggggacagcgcggagcatcctgcgtgcccgcaacatgcctggtcacttctggggagaagcagtacacactgctgtcttcctgctgaacagggcgcccaccagtgctcttgatggcataacgccgtttgaagcctggcatgcaaagaagccacccgtgcacttcctgaaggtgttcgggtgtgtggcctacgtcaagaaagtacgcccacatctcagcaagcttgatgatcgtggaatcaaggtcgtcttcatcggctaccaggacggctccaaagcgtatcgcttctacgaccccatcgccaatcgagtacatgtctcccgtgacgccatcttcgccgaggacgagcgctgggactggggagcatcctcggacgtggactgcgagcatcccttcaccatctcggaccactacatgctggaacgccagcaccaggagactgcagatcgggagcagctgatgtctgatcaccaaggttcgccatcgcactccctgtcccctggaggtgggagctcccaggacgcgccaccatgttctgcgacgcgggcagcatctccagcagcgcaaagcccgcagcaagccgagcagctgccttcgacaccagcccacgctggaacaggcatcgagttcgtgacgccactttccaccgacccaagcttcgacgacgatgatgtaggagagcgtcggtaccggacgctggacaatattcgctgcgtgggtgcagcggccgggccgatgcatcatggcgatgaggaggctgagctgcatgtcgtgagcgtggaggagcccaggaccttgaaggaagccaacggtgatccaagttgggtcgccgcgatgcaggatgagctttcctcgatccgcgacaacagaacatggtcgctggtcgagctcccgcgtggtcaccgggccattggcctcaagtgggtgtacaaggtcaagcgtgatgagaacggcaacactgtcaagtacaaggcccgcctcgtcgccaagggctatgtgcagcagccgggcattgattttgaagaggttttcgcgccggtcgcatgacaggagtcggtgcggctgcttctcgccattgcggcgcactacggctggggcgtccaccatatggacgtcaagtcggcgttcctgaacggggaactccaggaggaggtgtacgtacaacaacctcccggctttgtcgacgagaagcataggcacaaggtactccggctccacaaagctctatacgggctccgccaggcaccacgcgcctggaatcagaagctcgacactgagttggtgtcactcgggttcactcgctgtgttgatgagcacgggatgtatacccgtgtcaaaggaggagtacgcctgatcgtgggcgtgtacgttgacgacctaattatcacaggcggtgatgcagttgacgtggccaagttcaaggtgcagatgaagaacatcttcaagatgagcgaccttggccttctctgctactaccttggcctcgaggtctctcaggggaagcaagggatcacgctccgtcaaagtggctacgcctcaaaagtacttgagaaggcgggactggctggatgtaatgccagtgccacgcctatggagccgaaactgaagttgctaaaggacggcatgacatcgagcgtggatgccacagagtaccgcagcctaatcggcagcctgaggtacctgtgcaattccaggccagacctgggatatccggtgggttaccttagccggttcatcgaggcaccacaccaagaacaccttgcggctgtcaagagggtccttcggcatgtggcaggcacactggattggggcctgcactatcgcccaggacagaaaaatggaggggcactgaagttgctaggctactctgatagcgatcttgccggagatgtaaacgaccggaagagcaccagtggcctcattttcttcctagcaggtggtccggttgcttggcaatcggctaagcagaaggtggtcgctctgtcttcctgtgaggcagaatatatcgctgctgctgcggctgcgtgcgaggcagtttggctatcacgtttgttggctgaactcgtcggaggagctgtcctcgcacccaagctgaaggtagacaacaagtccgccatagcaccgatgaagaatcctgtccatcatgaccggagcaagcacatagatgtgaagtttcatttcatccgggaatgctgcgacaggaagctgatcaacgtcgagttcgtcggtactgagcaccagctgggcgacattctcacaaaggcgctcggtcgaactcggttccaggagctccgtggttggatcggcatggaaaaacttgtataaataataatcatgggcttaggaggagat

>Copia-72_ZM_LTR

tgttatagtaagtccagaattagtgtttatttcttagcaagtagattagcacacaattaccttccagtattgtgatttacttaccacattctactgcatgtaatttctttataaagagatagaaaacaaccagaaaatgcagtctgattgacagcaccaaatccatcgtgtcctctgtgtctgcttcgcgtgtgtcatcctcctcgtcggagttctaaca

>Copia-73_ZM_IN

cgttatcacgcacgtggctctactaaaggagatcgagcaaaaggtattaagaaagatgttgaactcatgtattttatgcatatgttttgaagttcttttttcctttttcacagcacaagcatgacatcccctagcagatttatcgccggcacgaagccgatggggatgcacacgtgattgctgctgatttgaagtcgccgacgcacacgtcgatcaatcacgtgtgatacgaacacgtttatttactgcatcatcgcaagaacagagaaaaggacagaaacaacagtagcagcctcgcttcgcgcactcgcgtccccctagccgcgagcagcgcgcgccgccgccgctccacgcaggcgcggccaggtggccatcgctgcccagcgcgggcacgcagcagctgcagcaggccatcgcgatcgcacggtggcaggcgctgcacagcagcaggcccactgctgcatgcctcgaggcatgcgttcgcttccgtccacgcatcggccaagcatgcgaacagcacagcaagggcgagagctagggaatacaacgtccattgacgttctggagcagccacatgcaaaaggaagtkcccctgcactggaaacaaaagcaggagtgggacaaggctaacggtaaaacgctggctgatgcacgtcaccacgaaagatgtggaatgaagagctgaccgctccctttacaaggcggcgtggcccagcatgatgtcatgcataacccacccgccgctgtctcatgcgggcacgaagcgccaacgcattgtagacatcattgatgctgcagtcccatgcgttagccggtttttttttaaacgaaatataattgaatgtgactgcattattatgcatctagaaatttatttctgaaataatacaagttaaacactcaaagttaccttcttgtgcatttaaatttgtatattgcaccatttattttattatatttttttgcaattatttatttcattctctgaaatgattacaagaagtaatctaattatgaaatttatcacatgtagatgactggtattgtgagccgtgaattcgaggtgttggccccgcatggccaaaactacctcacgtgggcatctgatgtgcaaattgtgcttgggggcaagaagctcaaagttgcaatcgggcttggccaaaaagatgaagtggccaccgacgagcaaaatgaccaagcattgcactttctccggcatcatctctcgcctacactcaagaatgagtacatgtcagagagaaaggcaagcaacctctggaatgcattgcaacaacgttttgagaggttaaagtacacagtcttgccccaagcacagcaagactgggctcgccttagatatgccgactttaaaactgtgggagaatacaatgcagcactgcaccgaatatgcaccaaattatctctttgtggtaaggtgataacagatgaggaaaagattgaaaaaaccttgtctaccttccacccaagcgccatccaatctgcccgcaactatcgacaagacgcatacaagcagtatagcgatcttatcgatatgatgcaagtaaatgaggcacaagatgatgcwttgaagacgaacttcaatgcttatccaaatggaaagggcattagcactgaagttaatgtcgcctcgtacaagataagaaagcctatccagagaaagagaggcaagcatggtgctggcaagggaaagaaagaaaccacagtcaatcccatcaatgctcacggaaagaaaacaagcaaagagaagccacaacataagccatacggacttcaagaccaaacatgctacaaatgtggcgtgtggggacattggtccaaaatatgccagtcaccaaagcatgtcatagaagcatacaaggcaaagaacatatccaaacagaaacctgaggcacacttcaccatggcaaccaacaatgaagccatggaagaagaccctacgcttgaagcatctggcatgcctcttgatcttgcaggtgctgtgaagatgattgccaccgaagagaacacctccatagctctagtggaggctgacaccgatgccttccttgattccatttgagtgcttgcatgcacatttatttttcagagtttgaaaaaaaacataattatggtctgtaagctctataagagcattatctttatttcatgaatgttgaactatttaaatttactatgcaatatttcccttatgataattactaattgcaataattgtattatgaaaggtctatggaggatatatgtattgtcgacagtggcacgacaaataccattttaagggaaacaaaatactttcacacgatcatgaagaacgttggtagtattaccacaatcgcgggaagcgattcatgtgtaataggctcaggtaaagccacaattacactcccgatgggtactcaaatcgaaatcaaagaagctctattataccctgaatcaaccagaactctcttgagttttagggacattcgtgcaaatggttttcatgtagaaaccacagaggaaaatggtaaggaatacctatacatcataaagtatggggaatatgacaaagaagtcatagaaaaattcccatcgctagaatcagggttatattatactaagattagtgcacctgcagtgtacacttccctaaagacggtgtttagacattctgaattattctctctatggcattgtcgactagggcaccctggtcttagaatgatgagaaatatcatcaataactcacaaggacactgcataaatgtgaaacacttcccaaatcaagaagattttgtgtgccagggatgtgccatgggaaaattgatagtgagaccatcccccttgaaagtaaagaatgaaattcccgccttcttggaacgaatccagggggacatttgtggtccaattcaaccactttcgggaccttttcgatattttatggtcttaatcgacgcatcctcgaagtggtcgcatgtatgcttactatcaactcgaaaccatgcatttgcaaagttgatctcgcagatcatccggattaaaaatactttccctgaccatcgaataaagtccattagaatggataatgctggagaatttacctctaaggcatttgatgattattgtaccgcatcgggaattgcggttgagcactctgtgcctcatgttcatacgcaaaatggactagccgaagctttgattaaaagaataaagctaattgctagaccactcttgcagagttgtaacctacccaacacatgttggggccatgccatcctgcacgcagcagcacttatcaatttccgtccatcagcatacaacattcactcaccaatgcaactagtgcaaggagtcatcccaaaaatctctcacctaagaaaatttggatgcatggtatatgtacctataccacctccacagcgaagtgcaatgggtcctctccggaaaatagggatatatgttggatatgagagtgcatccataattagacaccttgacccaatgaccggagaattgcatacggcccgattcgccgactgtatttttgacgaggataatttcccatcattagggggaggaaaagaacctttggatgagaaatgccgagaaattatatggcaatccatgggaatccccgctcatgaccctcgcaccaaagaagcaaatctagaagttcaaaagataatcgatttgcaaaacttagcaaatgaactgcctgatcatttttgtgatttgaagagcgtggttaagtcgcatgttcctgcacggaatgcaccagagagggttgaaataccaaagaaaattgaaggtatacccgatcctgttaagaggcctcataacaaaaggacaggaactctggcctctcaaaccccaagtactcggagacgtccgaagaaaagagggcaagaggacttgccacagccgatcacagaaatgccccaaagcaagaaggggagccagacgagacctggcaccgggaaggaacacatgaagaaaagmattccggacttgaaccttcccgtwgaagaaaccgccaacccaaacatgtgcgcacacaaaggcgatgaaaaactaaaggaactcgctcccgcaacaagaagcaatagggaaacacaagaagaaatgcaagaagcatcccatgatgaaatggccataaactatgtattaactggagaatgcatgaacagagcaacagccaacatagacatatatttcgcaaaaaaagttgcctcgatcattgatcatgacccggaaccggcatctatcgcagagtgcaagaaaagaccggattgggataaatggaaaaaggcaattaccgcagaattaatttctctagacaaaagagaggtgtttgggcccgtaagccgcacaccatcccacatacaccctataggttataagtgggtatttgtccgcaagagaaatgaaaataatgaaatatcaaggtacaaagcaaggctagtcgctcaaggtttcactcaacgccctggagttgattatgaagaaacttactctccagtcatgggaggaattacctttagatacttgatctcattggcagtgaacttgaatttaaaaatgaaactaatggatgttgtgacagcttacctctatggaaacctggatacagacatctatctgaaaataccggaagggatcccagtcccaaaccgagatgagaaaaatagagcattatacagtgttaaacttaagaaatcactgtatggattgaaacaatcaggaagaatgtggtataatcgcttgagtgacttcctaagcaagaaaggctacataaacaacgaggattgcccatgtgtatttatacagcgatccccaaatggattctgtattatatcagcatatgttgatgacttgaacatcataggaacacataaagaaattgaagaggcaagctcatacttgaagtctgaatttgagatgaaagatttgggtaaaaccaaattttgtttaggcctgcagcttgaacattttgaaggtggcatattaatccaccaatcaacttacactcaaaaagtattagaaagatttggtcttgagaaggcatatcctgccaaaacccctatgattggaaggtcattacaggtagaccaagacccatacagacctagggaagagggggaggaagttcttggagctgaatatccgtacctaagcgcaattggagcattgatgtatctggcaaatagcacaagaccagatattgcatttgctgtgaacttactagcaagatacagtgcggaacccaccaaaaggcattggaaagggatcaaggatatcttccgttacctacaaggaagcaaggatcttggtttgttttatcgaaaaaatcaagatctaaatcttatcgggtacgctgatgctggatacctgtctgatccacattcaggcaaatctcaaactggatatgtatttctttgtggaggaactgcaatttcatggaagtcgtcaaaacaaactttggtatcgacttcaacgaaccactcagaaattattgctttatatgaagcctcgcgtgaatgtgcatggcttcgaagaatgatcaatcatatccaatccgcatgtgggttgaatattgagcaaacacccacaatcatttatgaagatattgcagcttgtgtcgcacaagttcaaatgggctatgtgaaaagtaatcttacgaagcatataaaccctaaattcttctatgcacatgagctgcataagatgaatgagattaaaatattgcactctaagtcatgtgaaaatctcgcagatatgttcacaaagtcactccctgcgtcatcttttgaaagatgcgtccgaggacttggaatgatgagacttagagaattgcagatctcagggggagaaacttcacacacacccaaataatcttaaacgtaaattttaagtacccaaataatctagaagattaaattgttatattgggtgaattgtactctttttcctttatgtgagttttcatgaaagtttctcactaaggttttgatgaggcaattcatgcgacacagcaagtgtgagctatgtactctttctccaattttttcccactgggttttttatggagttttgggacatatgcatctcgcggcattgggccaagggggag

>Copia-73_ZM_LTR

tgttaagaaacctttcttgggccaggaaggcccatgtggcccatatgcctgaaaccctagttttgtctataaatagagaggagatccccatatcatgtaacctttggagagcagcaccatatagtaacaaagaatcagttcctctctacactcttgtctccttgtgttctactattattcagttaagcagcccttggactacactcggtagcaaggggttcttaaca

>Copia-74_ZM_IN

tggtatacagagctagggttaggcgccgccgcagcccgccgccgctcccgcagcccgcgccgccgacgcccgcgccgcgccgcctgctcccgtcgagcgcagccgcgccgccggcccgacgccgctgctcccgacgcccgcgcggccgagccccgccgctggaaggcgcggggcggcgagagcgaggggcagcggctcaccgcctccgcgtgggccggctgggcgcagccgcgcggccaccggccgcagaccgcgccgagtcgccagccgcagcgccctgccacctccacgcatcgccactccgacaaacgagctgctgctgacttgcgtctgcgtcctgtctacacggaggctgaaacaggaggaaaggaggagaggaggacgaggtctgcggtcggagtagagagctcgagaggctgcgagaaaggagtcgagtgtgtgactctgtgcttctgtgaaaatgagtacagaggaggctgcgaagaagacagaaattgatggtagtggttcaggagggattacaaagagtgagatgatgagtgaaatgagaagtatgatccaggagctcatggggttagggttgattggtgctaaggccaatacaggtacatctgagttgaagttggaacttgtgcctaatgatgtgaagttagaaggtagtaagaattatctaagctggtccagaagagtgcgtgtactcctaggaggcaagggagtggagcattatttggaggagacatgtgttgagccgattgataagctcggcgcagaatggagaatctggcatgcgacaaactctgtgatagttgcatggttattggcatccatgtctcccactgttagcaagagggtggaagccatgcgtactgcatcacaaatctggaagaccttgagtaatatatactctcgaaaggggaatgtgatggtgatgatggaaattcagagcaaggcagatgcagtaaagcagatggggagaccagtggaacaatatgctagtgagctccagcacttatggggagagctggatcattatgctcctctccaaatggtttgcccacaggatgcacaaatggttcataagtgggtagaggataggagagtgacccactttctaaagaatctggattctgagtttgagagcaggagagcggccttttgtcaccaggagagtcttcctaccatggatgaggttgtgtctgccatgatagatgaagagagcagacttcgagtgatgagtagtggtaatcatgtgaagccagcatacgttgcaattgaggatagagaatgctataattgtggggaaaggggacacctgagttacaattgtcccaaccctagaggaaatggtggcaggggaggatctcgtggaggacgagggggtactcgtggtcgtgggagctatggaagaggtcgcggaggtcgtggtagaggccgtggtggctccagggccaacatagctaccactgaagagagtctctctgttactctgtctggggaacaagctaagcaatgggagcagtggcagaagggcaagtcttccgagtgcctcacttcacctgatgggcaagccaccaactccttcggtaactttgccaactacgcccacatgggcgaaggtactcaggcacaagcttttgcatcctcatgtaggcatcacatagattgggtcatagattcaggagcatcaaagcatgtgactggcatgtccacccctttcaaaacatacattccttatacacattctgagtcagttcaaattgctgatggtacatctcaacaaatccgtggtataggatctgttgagtgtacaccatctcttagcttgtcatcagttctgcatgttccatcttttccagtcaatctcctttctgtaagttcaatcattgaccaattcaagtgcacagtaatatttgatgagaattcttgtgtctttcaggagaagagtactgggagaaagattgggactggagtcaggcataacgggttgtggttcatcagtcatgaggagtcagcattgaaagcaactgttgagggggatggcaaggagattctactacatcatcgtcggttaggacatatatcttttgagaacttaagtcggttgtatcccatgatgtttaagggagtggacaaaagtagacttgtatgtgatgcatgtgaacttggcaaacatactagatctacctatcctagtgttggtcttcgtagttgtgaaccgtttattctaatacattcagatgtttggggtccttgttcagtcacttctgtgaatggtgcaaagtggtacgtcacttttattgattgttacactcgtatgacttggatctatatacttaagcataaaaacgaggtgttaaagtgctttcaagactttcataaactggttgcaaatcagtttgatgcaagaattcgggttatcaggactgacaatggaacagagtatgtgaataatgaatttagatcatatctgtcaggtcaaggaatcattcaccaaacaacttgtcctggaactccacctcagaatggtgtagcagagaggaaaaatcgtcatttgttggaagtagcaagatctctcatgtttcagatgaatgtgcctaagtatctgtggagtgaggcagtaatgacggccacatacctcatcaaccggatgccctcaagaatacttaatatgaaatcacctgcagaacttctattgggaaagtgtgactttcgtgttccacccaaggtctttggttgtgtctgttttgtcaaagatcatcgacctatggtgagcaagttggatcctcaagcagtcaagtgcatttttgtgggttattcatctactcagaaaggatacaagtgttgggaccctattggtaagagattgttagtgagtatggatgtaacatttcgtgaggaagacccatactatagaaagaaagttgatccatatcaaatcctggaggacttctctccggtcgatggaggagaccgtagagagggggaggatgacagtggtcgaggtggtgatgaggctagtggtgatgaggctagtggagcttcaggaggggtgattgttggaggtatgattccaccagaagtggagaaagggataactacgcagatattgagtgatgatgaaagtagtggggatcatggtgatccaactattaatattcaggaggagacagaggatgatgaggcagtgattgttgggtcaatcccatgtcctactgcagggaagttgaatgagaaacagggggagcaacatgattgttccattggggcaaaggtgaatgacaaacagggggagcaacctatagtatactatcgaaagcggacgaagaagcagggggagcaaccacaacctggatgtgttgaagctccagtcccatatctctcttcagactcctctccagacccagctggtaatgtctctccaatccatatttctcccattcctgaacatgaagaattacctcttgcgcaaagaagagttcctagagtaaatgctgggaaacctccttctcgttattgttatgaacatgacattgctaagtatgtctcatactctagtgtctctcctgcatatagaactttcattgcatctctacaaacaatatctattccgaaggattggaggtgtgccaaacaggatccaaagtggaaggatgccatgaaggaagagctgcttgcccttcagaagaacaagacatgggaacttgttcaccttccagaaggaaagaaggcggtggggtgcaagtgggtcttcacagtaaagcagactcctgaaggaaagattgacaggtacaaagcaagactggtggcgaagggatatagtcagacatatgggatcgactatgatgagacctttgcacctgtggcaaaaatgggcacggtgaggaccttaatctcatgtgcggccaactttggttggcctcttcatcagctggatgtgaagaatgcatttctccatggtgatctgaaggaagaggtctatatggaaatcccacctggatttgcaaataaacagactattgggaaagtgtgcagacttaagaagtcgctctatggcctaaagcagtcaccacgtgcatggtttgatagattcaggcgagttgtgtgtgatatgggctatcatcaatgcaatggagatcacacagtcttctacaaacatcagggctcttgtatcactattctagcggtttatgttgatgatatagtgatcactggagatgatgcgaaggagatcaagaaactgaaggaaaggctaagtagagcctttgaggtaaaggaccttggaccactccgatacttccttgggattgagatagctagatcatctcagggaattattctctcccaaagaaagtatgtcctcgaccttttgtcagagacaggaatgcttgggtgtcgcccatgtggctcacccattgataggaatcaccaaacttgtgctgagtcaggtgaccctatagatcgggaaagatatcagagggtagttggtcgtttgatatatctttgccatacaagaccggacattgcttatgcagtaagtgtggtgagtaggtatatgcatgatcctagaactgggcatatgaagatagtttatcaaatcctgagatacctgaagggaacccctggcaagggattgtggtttagaccaaatcaacatatgaatttggaggggtattgtgatgcggactgggcaagcagtagggatgatcggagatccacttctggatattgtgtatttgtgggaggtaatctggtttcatggagaagcaagaagcaagctgtggtatctcggtccactgcagaggctgaacatagagcaatggctctagcagtgtgtgaaatgatatggttgaagggtctcttgaaggagcttcaagtgttgaagaatgagacaatgttgctccattgtgacaatgtggcagcaatcaacatagcaaataatccagttcagtttgatcgcatgaagcatgtggagattgataagttctttatcaaagagaagattgatagtggggctttgaagttgaagtatgtcaaatctcgtaatcaagtagcggatagtctcacaaaaggtttaggtcctaaggataatgagttggcatgtaacaagatgggaatgttaaatatatttagcccatcttgagggggag

>Copia-74_ZM_LTR

tgttggtgtaattatgaacaagtgtaagggtctatttgtaaagtggtgaagtataatggaagggagtgagggtgctgtgtgagattgacacacaccagaaaatcaaca

>Copia10-ZM_IN

tggtatcagagaggttcgtttcgaaccatcgcttccgcctttcctaaccacgccatctcgctgccatggccgacgacgaagtcgcagctgctgctgctcgccgtgctttggagcagcgcgtcgccgcctttactgctgaagaacgccgcgaggctgctcgccaagaagctgcacacctggaggctgcgcgcatcgaggccgagcgtcgcgaagccacgcgcttggagcaggaacgccgcgacgccgatgcccatgccctggtcatcgctggtgaggtcgctgcggctactgccgctcttcatgctcaagcagcagcgatcctcaacgtcaagtctctcgttcccatcgtcctcgacttcacgtcgccccactacaatcgatggcgtggcctcttcctcaacactcttgagcgctatgcgcttgttgatcatgtgctttccgacgacgaccgctccgccgacgccatgtggaagcgcatggactgcactgtcttgtcctggctctacggcaccatcaatcccgagctcctcgaggttgtcatgaaccgggacgatggccctcccactgctcgtcgcgtgtggctcggtctcgagcagcagttcattgggaacaaggagagtcgcgctcttctcctcgacgccgagttccgcaccttcgtccaaggcggtctgtccatctccgactactgccaccggctcaagtccatggctgaccagcttgcggatctcggcgaacccgttcgcgaccgtacgttggtgctcaacgtcattcggggcctcaacgacaggttctcctacctcggcgcgctgatccagcgtcaacgtccgttcccgacgttcgccgaagtcaagtccgatcttcgcctcgccgaaatcaacatggctgccaagtctgcccagccgcctcaggccttcgctgcctcggcccctcctccgcacaacagcggcggacccggcggcggtggacgcaagcagacaaggcgtggcggcggcggcaagaaaacctctggaggtcgcggtcctggttccgtgccccctaccgcgacttgggytggccagccgccacggcccacaccctggcaacctgcatttggcactttccaggcgtattgggccggcccgccaggaccaccaggcgtcccgccgcgtgcacctggcccacctccgcagactttctacgccggcccatccgtgctacctgggccgcctccgacggctgcttcaccaggcctcctggcgtccccgcctgggccatcctagccggctccaccaggcttcatcggcgccaacccgccaccaccaggatggctgcaacatcctggcgccttctcctgggacgccaacatgttggctgcaaatttcaacacgatgacgcttcagccaccgccaacaaatgaatggtatatggatacaggcgctgaaactcacatgacatccaactccggtaatctttgcacctcgcagcttccttctttttctactccgtccaatattgtcgttggaaatggttctcttttacctgtcactcacaccggctctgccacccttcctgccatatgtggtcctcttcatttaaataatgttcttgtttctcctcacctcattaaaaatctcatttccgttcgacagtttactattgacaataaatgctctgttgaatttgatccctctggcttttctgtgaaggacctcaaaacccggaacgtgatcgtcaggtgcaatagttccggtccgctatacccgcttctatcatcggtttttcagcccttagcactcactgccggcgtctcttcatccactctttggcatcggcgtcttggacatctcggtcatgaggccttgtctagtctcgtgtcatcatgtgctatttcttgcaataagagtgattccgaacacttgtgtcatgcttgtcaattaggtcgtcatgtacgtctcccctttcccacctccagttcgcgagcaatcaataattttgatttaatccattgtgatctttggacatcccctgttcctagtatttctggctacaaatattatttagtcattcttgatgattgctctcactttctctggaccttccccttgcgtctcaagtctgacacattttctactctcactcaattttttgcctatgtctccacccagtttggtgtcaccattaaaagcgtccagtgcgataatggtcgtgagtttgataactcctcctcccgcacgttctttgccacacatggtgtcactctccgcatgtcttgcccccatacctcgccacaaaatggcaaggccgagcgcatcattcgcaccactaataatatccttcgctctcttctattccaagccagcatgcctccgtcgtactgggttgaggctcttcatactgccacctatcttctcaatcgccatcccactaaaaccttggcctcggccacacctttctttgtcctattcaaaacccaaccctcctatgaccatcttcgtgtctttggttgtcgctgttaccccaacctttcctccaccgctgcacacaagctagccccgcgctccactgcctgtgtcttcctcggctatccctccgaacataaagggtatcgctgtcttgatctttctaccaatagaattatcatctcgcgtcatgtcatttttgatgagacctcatttcccttttctgaagtctcttcgcctccttcctctgactttgactttctctcggagttggatgctgctcctgttatttttcctggtgtctcacatgtttcaggtaccatggctcctcgcaccgctgcccctgttggctccttgccccaggttgctgccagcggtgccacgctgcctccggcctcgacgtccgcgggggtggtctctgacgcgaccaccactgcgtatcctggtgctgccagcggtgctacactacttccggcctcaacgtccgcgcgggtggtcggtgctccccatgcgacttcctcagcctcgcccactcctgcggaccgcactgggagtgcgtctgagcgcaccgcctctccggtgtctggtgcgggctgcaccaccggtgctccttcagggagcatgagcctgcctgagatcatgccacctctgagcggttcgacgccacttgttgagcggccggcgcctccaaatgcggtcactactcagcctctagccaatgaccacagcatgctaacgcgtggcaaacggggctttcgtcaaccaaaggtactgcttgatctgcatgttgctcctttgtctcccgtgcccaaatcctaccgaggagcattggccgatcaaaattggagagccgctatgtctgaggaattttttgcacttcagaagaataacacctgggacttggttcctcgtccctcgggtgctaatattgtgactggcaaatgggtttatcggcacaagtttcgctctgatggttcactagagcggtacaaggctcgctgggttcttcgtggtttcactcaacgtcctggaattgattttgatgagactttcagtccggttgtcaaacctgctaccattcgtacagtactcacagtggctctcttccatcgttggcctgtccatcagctcgatgtgaagaatgctttcttgcatggcacccttgatgagactgtctattgtgtacagcctgctggtttcgttgatagctctcggcctgatcatgtctgtcgtctgaacaagtctctgtatgggttgaaacaagctccgcgtgcctggtacagtcgttttgccactcacattctctcttttggctttgtgagtgccaaatctgacacttccctgtttatatatcaacatggccatgacacaatttatttacttctctatgtggatgatattgtgctgacagcatcttcagcctctctgctccagtgggtgattacagctctacagcaagaattctctatgacagatctcgaccctttgcaccattttctaggtctcacggtgactcgccagcccaatggatttcatctttctcagcgacagtatattcttgaagttcttgaacgtgcaggtatgatagactgtaagccatgttctactcctgtcgacacgaatggcaagctttctggtactgacgggccaccggttgatgatccgactcactatcggggtcttgctggcgcactgcaatatatcactttcactcggcctgacatttcctatgctgttcagcaagtttgtttacacatgcatgatcctcgggaaccacattatacgttgatcaaacgtattcttcgctatctgcatgggacattggatcatggcttacatcttcgccccacagatgtttctgctttggttgcctactctgatgctgattgggccggttgtcctgacacccgtcgttcgacctctgggtatgctgtttttcttggtgataacctcatctcttggtcatccaagcgtcagcctactgtttcccgttcaagtgctgaagcagagtatcgtgcagtggccaatgccgttgctgaggctacctggctccgtcagctccttcaggaaatgcattctcccctgaagcacgccactgttattttctgtgacaatgttagtgcagtctatctttcctcgaacccagtaaatcaccagcgtacgaagcacattgagatcgatctacacttcgtccgtgagcgcgttgcggccggtgttgttcgagtattgcatgttcctactacctctcagtatgctgatatcttcaccaagggattgccttcatcggtgttcacagaattcaggtccagtctgaacattcttccaggtggctgaccacgttcagactgcggggggggggtgttggattactatatttagattgatcatcttcct

>Copia10-ZM_LTR

tgttttactattgcatgccaccattattgtatacgtgcatgacacggtgatcaccttccatctctagtacatggctatacttagaaggtactaatcaggtgatcaccgtccatctctaccgggcactgtttctatgaaccggttggagcctagtctatatattgttggtcttgttaatcaataatacaacaatcattcgactatca

>Copia11-ZM_IN

agtggtatcgaagccgacgatcatcctgtgaagatgtctggcaggaggggcgacgagaacatctccaaagatggagtggtgatccagagggtgattcgcgaagttaatggcgggagtagctaccccaccctaaccaagacgaactactccgactgggcgctcctgatgaaggtgaaactcaaagcccgagctctctgggacgcgatccagcagggtggcgtcgattctcatgaggagatgatggcgctggacgtgttgtgcagcgccgtgccacctgaaatggtgccaacgctggcaaaaatggagacgaccaagcaggcctgggatgcgatcgcaaccatgcgaatcggagacgaccgggtgaggaagtcgacggcgcaacaactgcgtcgaaaattcgacctcgccgcgttcgacgacggcgaaaccgtcgaggagtatgcactccgcctcaacaatatggcggcgcaactcgccactttgggcgatgaagtgatggacgacgtgatcgttgcgaagatcctccgaagcttgccgcctcgattcaagcaaattgcaatcgcgatcagaacgctgctggacgtgacgacgatgtcagtcgcggatctggtagggcgattgaaggaagcggaggaggcatttgaggaagtgccgacctcgttgaagcatgaagggaagctgtacctcaccgaggaagaatgggatgcacgacggaggaggcgcgagctggagaaccacttcggcggcggctgccagaggcggcgcgagctcaagcagaggaggaggctggcgaggacgcggtcgtgggcgcgcgtcaggtgggccaggcgggtccaacaaactggcctgggatgagtgccgacggtgcggcaaaactggtcactgggcccgcgagtgccggtcgaagcccaagagggagatggcccacgttactcaagaagaagaggaggccaccctgttgctcgtgaagtcttccccagagaaaattacggtgccagaaaaattccccattgctcaatccgaaagagcgtggaaatccagcgcccaaatcgtgatcaaagaggaacgggtgctcgcccacctcgatgataggaaggagcgtgatgcggagacatgggtgctggacacaggggctacaaatcacgtgtcggcgccagagctgccttcatcaagctcgaagagatggagctgggatctgtgcgtttcggggatgactcgatggcacggattgaaggccgcgggaccgtcgcattcctgtgcaagactggagaaatcagatcgctgggcggagtctactttattccccgactgacgacaaacattgttagcatcggtcaactcgacgaggctgggtacaaggtcgatgttagcgcgggagtaatgaagatccaggagcctggcggtcgactgctggcgaaggtacggcgcgaaacaaatcggttgtatctgctccaagtcaaaatcgccccggcaacttgcttagcagtgcgtgggcgaaatgatgaagaggccaggctctggcatgagcgttttggccatgtcaacatggcggcgctccggaagctagctcgggaggagatggtgcgtggactaccattgatcgggcaggtggacagggtgtgcgaggcttgccaagccggcaagcagagaaggacctcattcccgatagaagcagagtaccgcgctcatcagcgcctcgagctagtgcacgcggacctgtgtggtccaatcacaccggcgacgccacgaggtaacaaatattttctgctcatggtggatgatttcacccgatatatgtgggtagccgcaatcccttcaaaggattgtgctgcggctgccatcaaggagatacaagcacaagcagaaggtagatctggggcgaaattgagagcacttcgtacagatcgaggaggagaattcactgcaactgaattcgcgaagtactgcgcagctgaaggtatacatcggcagcacactgcaccttacagcccgcaacaaaatggagtcgttgagcgcagaaatggctcagtcgtagccactgcaaggagcatgctcaaggccaaaggtttgcccggctggttctggggtgaagctgtaagtaccgctgtttatgtgttgaacagatgcccgacgaagagcgtggacggcatgacgccattcgaagcatggcatgggaagaagccagcagtgaagcacctcaagaccttcggatgtcttgtgtatgttcgaaacacaacaccgcatctgaagaagctcgaggatcgcgggaggaaaatggtgttcatcggctacgaacacggctccaaggcgtacagggcatacgaccccgtcacgaagcgtgtccatgtgacgcgcgacgtggtattcgacgagcgcgcccagtgggactggggatctgttgaagaggccggggaacccagtgggggcagcgatgtattcaccatcgagtacactgtcacaagtccagcggccccggctgttgaagcaagggcagatcaagttccgggtgaaatcgaagacatggtggagacagaggccagtgcagatgatgatgacatggatgcagaccatgacgatggtgtgcctctccgattccgcagcctggacgacctctatgggccagcgtcatcacagggcgtcgcaccgcaaggtacattgcgccgcagctcacgagtgccagtaccgcgtgttctggcaacagaagaattacatgcagtgagctgcgacgggccatcatcattcacagaagctgaacgtagcccaagctggagaaatgccatgatggaggaaatgaaggccatcgaggagaatgggacatggtgtctcgctgatctgccacccggacgcaaagccataggagtaaagtgggtgttcaaggtgaaacgcgacgagcgtggggcagtgtccaagcacaaggcacgccttgtggtgaagggctatgcgcagaggcacggcatcgactacgacgaagtgtttgctccagtggccggctggattcagtacgcctgctcatcgccctcgcggcgggaggtgcaccatatggatgtaaaatcagcgttcttaaatggcgacctccaggaggaggtgtatgttgaacagccggttggtttcatcgctgcagggaaggaacatcaggtgctcaagttgaagaaagcgctttatggcctacaccaggcgccgcgcgcttggaacgcgaaactggacgacaccctggtgtcgctcggttttcagaggagtccgtcggagcatgcaatctatgtcagacagagaggcaatgcccagttgacaatcggtgtctacgtcgacgaccttgtgatcactggctcaaatactgacgacatcaaagaattcaaaaaagaaatggcagcagcattcaagatgagcgaccttggcttgcttcactactacctcggcattgaagtgaagcagagcgcagagggtatttccctgagtcagggtgcctatgccaggaagattctagagaagaatggcatggcgacctgtaatccctgccaagtgcccatggaggcacatttgaagctgagcaagttcagcactgaaccaccggtggacgccacggcatacaggagcgtcgtcgggagcctaaggtacttggttaatacccatcctgatatagcatttgctgttggctatgttagtcgttttctggaggatccacgtaaggatcacttggctgcggtgaaacatattctgcgctacctggcgggaaccaaaacttgggggctatggtttgagcggaggaagaaagaggaagcaaaactgataggtttcagcgacagtgattttgctggtgatgttgatgctaggaaaagcacaacatgggtaatgttcttcctcaatagcagcccaataacttggcaatccatgaagcaaaaggttgtggcacaatcgagctgtgaagcggaatacatagccgctgcaaacgccgcttgccaagctgtgtggctggctcgggtactggcagaggtgcagggaacaacaacaaaggctccaatgttaagggaggacaatcaatcagccattgcgttaatcagaaatccagtacaccatggacaaagcaagcatattcaggttaaatatcattttgtcagggagtctgaagatgaaggcctaattaatgtgaagttcatcaggagcgaagaacaacttagcgacattctgacaaagccactgggaaaaatcaagttcctggagctaagatcaaagatcggcctgattaatgtacagcagctggcacctcaaggtttaggaggagaa

>Copia11-ZM_LTR

tgtagaaagtaatccttggatgccatgtttagcagtttatcttgattagcttaaattaggtgtatgattggaataaggctgattgagagccgttatttagtttccttattacagtagttcctttccatgtaatagtctatataaaccaagagatatcaatgaaaaagcagtcactcgacagcaccaaaaataagatcgtgtccactgtgtttcgcgtgagtttctgagtgttgtgttggccgtgatctcgccggcatcctgtgcggtgtcactccggcgaggttctagttctaaca

>Copia12-ZM_IN

attggtatcagagccttgttgctcatagattagcttaaccgctagagttacgatgtccggtggggatggacctcctcccgtttttgatggtgacgattttccttattggaaaattcgtatggaagcatacttagaggctatagacattggtgtctataaagccgccacacaaggattccccgaacctagagatcccacaaatcttgtaggtgatgagttcaattatgagaaatggaatgctaaggccaaaaacaccctttttagaggcctttgcaaagatgtgttcaatagagtaagaaatcataaaaatgctcatgatttgtggatggacatttgtgctctacacgaaggaactagaagtgagcgtgaggagagatatcacatagccatgcgaaagttaaattcttttgaaatgcttactaatgaaaatgcaaatgctatgtactcacgtctcaatattcttgtagaggaagttaatggattggggcttactcaaatctcacaaccggatgttgtgaggaagattctcagtgtcctcccaatagacaaatatggacacattgtcactgtgctacatcaaatggatctttcagttaccacacctacacaaattttgggaaagatcaatgcccatgaaatgtacatgcacatcaacgacaaagaagaatcatcttccaaaagaaaggatttggctctcaaagcaaatcatgaaagaaaaggaaaagcaaaaatacaagttgaggaagaatcctcaagtgatgatgaccttgatgcaaacattgccttgatggtaaggaagaccaccaagatgttgaagaagctaaatagagaaggcatcaaatttgattcaagaaagaagaaattcttttccaacaaaagaaagcccatttctgagatggactgctacaactgtggtgagctcggtcatcttgctcatcaatgcaacaagcccaagaagaataagttcaagggcaagaaagaagatgacagcgatgatgaaaagaaggaaaagaaattcttcaagaggaaggatgggaagcaaaagaggttccacaagaagaagaatggaaaggcatatattgttggcgactggctcaccgacattgaatcatcaagtgggtcttcttcaagtgaagaagagaatgatgaaaaagttgccgccatcgccggggacttctcttcaccaccaccatcgccatcatcgacttctcacctatgcctcatggctaggggtgaacggaaggtacaaaatgatattaatattgctgatgatagtgatagtgatagtgatgaggaatttgcttcaccttcatatgatgagttagctgacttgcttaaagaatatactcaaatcattagaaagtcaaaagctaaatgtgataggttgaaaaatgaaaatgaatctttaaatgccaaatatgacatagttataaaggctagtgatgaaataaaagaagaaaataaaactatgtcatcaactgtaaatgagctcacaaactccctaaaagatgctaaggaaaaatatgacaaattaaatgaagctaatagggagttgcaaaatagactagtaaagatcaaggaagactatactcaaattaaatttgatcatgacaatcttcttgttgaaaatgaacttttatcttgcaaaacacatgaggctattaaccctgttgttaagattgatgtagcaacctcatgtgatgatttgagtcaagaagagcaaactagtctacatgctgaattgactgaaaaagttgaagtcctgactttagacaaccaaaaattgaagaattacttgactgatgcaactactagaggaaaggttgctattgagaacaatgatttcaacaatgagttggcagtggataatcaaaggcttaaaaatgaggtcaagaaactaaaaagtgaaaatgaacatcttgcaacaagtgtgcaaaagttcaacaagggtcaatacctccaaaatgagctgcttatgaacactgttatgaaaaacaacaagagtggtattggatacaatgcttttgtgcaaaagaaagctaccactcaatacaagccaaagcagactcacaagcctatcaaatgctttgagtgtggaaatgaaggtcattttgcccacaactgcaaagccaaaccaccaactcccttgcctaagcactcaagaccatttgctttcaatgctcattatgttctaagaaaggttgcaaatggaaaggttaaagttacattcctaggtccaccaaacaagagtagacctagacaaatctgggtggcaaagtccttaattgagagagtcactggtcctatgcaatatagggccctcaaaactcaagcttgaattgtctgtggatgtaggtgaactacaagaccggtgggagccattgggttattgacagtggatgcacacaacatatgacaggcaacccacggatgttcacctcactagatgagaatgttgatggtcaagataaaatcacatttggagacaattcaaaaggaaaagtgcaaggacttggcaaggtggcaatctcaaatgatttatcaatatcaaatgttctcttagttgcacctttgagcttcaatttattatcagtgggtcaactctgtgatcttggacttcaatgcttattcactccaacagaggttattgtaacaaagatggatgatgaatcaatggtattcaagggatttagatacaacaacctctacttagtggatttcacttcagaagatgcagacttaagaacttgcctcttcaccaaagcttctcttggatggctttggcataggaggcttgcacatgttgggatgagcacactcaagaaggtattaaagaaagatatggttagaggattaaaggatgtggtgtttgaaaaagacaagccatgcagtgcatgtcaagctggaaagcaagttgctaatacacatcctactaaagctttcatgtcaacatcaaggccactggaactacttcatatggatttatttggacctacaaattatgtaagtgccggtggcaacctctactgtctagtgattgttgatgacttctcaagatacacttgggtgttctttctccatgacaaatctgaagttgcatctatattcaagaagtttgccaagaaagcacaaaatgaatttgattgcaagatcaagaagattagaagtgacaatggcaaagaatttgacaacaccaacattcatgagtactgtgatgaaattgggatcaagcatgaagtatctgccacatacacacctcaacaaaatggagttgttgaaaggaaaaataggaccttgatcactcttgcaagaacaatgattgatgagtataacacaccggagaggttttgggccgaagctgtcaacactgcttgttatgcatcaaacaggctatttcctcaccggctacttgcgaagactccttatgaactgctaaatgggaaaaagccagacgtctccttcttccgggtgtttgggtgcaaatgctacatctacaagaaacgccatcacctagggaagtttcaaagacgttgtgatattggttttcttttgggttattcattaaagtccaaagcatatcgagtattcaaccatgccactggcgtggtagaagaaacatatgatgtggaatttgatgagactaatggctcccaaggagcacttgaaaatcttgatgatgtaggtgatgagccacttagggaagcaatgaagaacatgcctattggagctatcaaaccaaaagaagatgaagaagaggtgcaaatcattgacaggccttcttcatcaaatgtaccacaagatgatgaaaaagatgagaggcatgcaaatgaagatacatttgtctctcatgaacaagcaagggtacaagccgaagatgttgatgctccaggatcttcttcccaagtggttgacaggagaaactcatcactacttcaagctcatcctcaaaaccaaatcatcgggagtccttcacaaggggttattactcgatcacatagacatgcttcttttattgaacatcactcttttgtttcttgtgttgagcctacttgtatagatgaggcgctacaggatccggactgggtgaatgccatgcatgaagaacttaacaacttcacccgtaacgaagtttggaccctggagaagcccccacaagatgcaagaatcattggaacaaagtgggtgttcagaaacaaacaagatgatcaaggtgtgattgtaagaaacaaggcaagacttgttgcaaagggattctctcaagttgaaggcttagattttggagagacctttgcaccggttgctcgactggaagccatccgtatcctacttgcatatgcatcatgctatgatataaaactttatcaaatggatgtaaaaagtgcatttttaaatggcttcataaatgaacttgtatatgttgagcaaccgcctggatttgaagaccctagatatcctaaccatgcttacaggttgtccaaggcgctatatgggctaaagcaagctccaagggcttggtatgagcgccttcgcgacttcctcatcgaaaagggcttcaagatcgggaccgtcgacacaactctcttcacaaagaaacataacggtgatattttcatttgtcaagtatatgttgatgatataatctttggctcgacaaatgactatcattgcaaggaatttggtgagttgatgtcgaaggagttcgagatgtcaatgattggtgagctaacgtacttcctcggctttcaagtcaagcaaatgaaagatggtaactttctctcacaagagaagtataccaaagacttgttgaaaaggttcaacatggagaagtgcaaaccaatcaagacccccatgcctacaaatggacatctcgacttagatgagggaggtaacccggttaatcaaactctctaccgttctatgattggtagtttattgtaccttaccgcatctaggcccgatattatgtttagtgtctgcatgtgtgctagatttcaatctaatcctaagaaagctcatcttcgcgctgttaagagaattcttaggtatctcaggcacaccacaagcgttggtctgtggtatcccaaaggagctacttttgatttaattggctattccgattcggattatgccggttgcaaaattgataggaaaagtacttctgggggatgccatctgcttggtagatcactagtttcatggacatccaaaaagcaaaatagtgttgccttgtcaactgccgaagcggaatacattgccgcaggtgcttgttgcacacaaattttatatatgaaacaaactcttctagactatggcgtagttctagaaaaggtacctttgttgtgtgataatgagagtgctgttaaaattgctaataatcctgtacaacactctcgcaccaagcacattgatatccgtcatcacttccttagagatcatgttgctaaaggagatatcattttagaaggagtgaggtcggaagatcaattagcggatattttcactaagccacttgataagacccgcttttgcatgttgaggaatgaactaaatattcttgatctcagaaattttatgtaaaatgtcaaatggtgttgtcaagcttgcattgcatgtttaaattccttgtattgcatctagggcttgtctaacctagttgagataaccgccaacaaagcgagtgaaaaagcttaactcgggtcaaacttgacaagtcttagttttaagcttttagcacttaaattcttactttttatgcaattgttggttcttgaaatatgcatgaggtactgcacttagggggagtattcaaaactcaaatcactcatgaaaacccctagtgcaagccaaaatgcaaatttcaccatttgcctattttctctaaaaattatctagcctatggcaaaatattttgaaaagtatatgagggtgccaatacctgtcccaacaagtgttattttgtgtgattataagttgggatttggtttggttgggaattagatagaaaaattcaaaattttccaatcttccctctgtctgggctcaccggacagtccggtgtgcaccggacactgcactgtgcaatgtccggtgcaccggcagccgcgcgctaaaaatcctttttcctgtgcgctgtccggtggttcaccggacagctactgtgcgctgtccggtgtgcaccggacaggcactgtagactgtccggtgcgcccatatcgcgttttaaaaaaaggcctccagcccgagaccgagccagaggctcttttccctctgcgctctctgttctctgcgactgctctctctctccggcgatcctcccctcgccggcgatctcccctccccggcggcgatcctccggcgacctcgtgcctgtgctcctcccctctccggtgagcagcactcctctcttcctctctctctctctctctctctctctctgttctstaggcagtgcactcccccatttccacccttttgcacaattttcaaatccctgtgaaatccagtgaatccaagtggtggaatgtgttcctytgtgtcccttgagtatccctgcaggttcctagctccttcgggagggttttcccacctcaaatagccatttcaccgaaaccctaattccccgcacgccacgtgttcggtgaaatgcccaaaccagccaaaaatgctccaattgaacccaaatttttcaggcaccttcacaacacttccagtaacatacttgccaaatttcacgccaatccgagattccaggcttaaatttcaccaaattccccgtttcgagcgatcgtttccgagccgagtgcccaaatctctttttcttcgcctaaattcaaaccaagcacacacttcactcatattcacctatataaacctattcgtgaagtatcggctcaattccatgtcgtttcgtgcctcaattcgaattccaaacctcctatggcactatttatcgttcaaacgtcgttttcacgtcgtttgacctctcgatcgtctcgtctcgtgttttctgtgccatatttctctgtgtatgtatttattcacatttcatatacttatgactatgtgactaatacgtgctcacctcttctgtcatttcagtgaccttgattgcccgtgtgccgtctcctgtcctgcctggatcgtcacctctcgtgtgagctttccaggtagtcatctcatcctttgctaaatctatcggacattttcgctctgtgcttagtcactctctctcatttgcagacatcagttcaggatgccgcgcacgaagaatgtgtcggcgccagggggaggcgatgatgaggatcctcgtcgccccttcaggcaggtcaagggcaagacagtttacttggagcagcaggaaggccgcaagaagcggcgtacggacagagcagcccgtgcagcggcagcagctgcagcagccgctgcgcaggccgagcttggagatcagccgcagactccgtcagatcagattgcataccgtgttcgtcgtctcgcctcccggcctcgctcctccactcacacctctgcttccactccgccacccactctgcctgctcccgtcactcctgttgcgccatccaccaccacagccatacccgctacctccactacgccagcttccactgctcctcctcctcccgctcccgcttcagctcctcctgtccctccacctcgattccgggagcgcgatgagactgaggtacgaccccttgctgcggatcctagactgtttgaccttcagcgtgctacagcggcacgggtacgtaggttcagatacgtacccgtggagtcttggttaccagctcagagggaccctgcagcagttgacctattcagcacacgtattcaggagtcgtttttcagagctcagatgtctgctcagatagctctgcgagtgcaccggcttttggatcttccagcctttctgcttgcagccggtgctgactctgaggcgcacctcacatatctgcctggccttctgacccttttgactaccagcggcaggtatgttgaggagtgggtccgagtcttctacgcctctgtatggattgacccggatcatcactggatgaggtttcgttttgagcgagaggatgtcaccattactgccagtcagatccgccagctctttggatttcccgagtcgacgactcgtcttcacagcctctgctacggcacttctgaccctcctcgtcgccctcacggcggtgtggctccgggtacagctcacgtcgcggctctgttccgcccgcccttcacagatgggtcgcgacgttcaccggcagattttactacagcagccaagtacttatatgagctcatcagacggactcttctgccgaggatgggatacagggaggctaccacacatattcagctctggctccttggtgccctggtctcccactctgagtttgacgtcgtggacttccttatttgtgagatcgaggacaccgttttggatgggattcgtgctcgtcggcagttgccatatgctcactacttgtgccacatctttgcgcagctgattcagcctcctcggtttcagggcacccttgaggcctcacgccttgtttttggatcctaccgtcctgcgcctgagactcctgtgccagcttctgctcctgtttttgactctcaggccgaggatgcagctcttcgtcagttcgacactcaggatacagcagctgatgatgatgatgatgatgatgattttggggttcctcctccgcctccgcctcctatgcctccacgctcacatgatcatgaggctgggagttctagtgctgcccctgctgcccctccggctatggaccctgctcttgcttcgattctccagtctctcactcagcagcaggctcacctggcagccgagcaggcccggttatccgagaggatgctctcgatgtttcagaacatgcaggacaggcaggatgcgcttcagcagcagctacttcaggatcgggctgagagtagggcattcatggctcttatgctacagcattctggtgtctcagttcctccggttcagtctgctccacctcctccgcttcacgctccagttgtgccatcgattttgtcaggaccccctattccttctgttggtacctctccgctccggccggtcactttggcgttcacctctccggttctcagctctgtctgccctgcagccgccagtgccaccagcatctgctgttaccactactgctgtggcagtatctgtgaccacttctgttccggtagctcctgcagcgcggcctcagtccgagtcagtaccagctccagcttctaccgcagatcctggatccgagactgactctgaccctccgccggcgttcgctctgctgcctcgaccgcgaccggatgcgcccccgccgcctcctcctgcttcagatgtttaggttcgggtacccttttggtgtttgacgccaaagggggagagatatgagttgtgagagctagggggagttagagagttagtagagagtcattttgatgtaatatatgtgcttgctactctctgtactagcttgatgtttttggctcacaaactcgatatatactctcgttgcttatgattgactgtgtgtattatgttttcaccttatattttatcaccagtcttctagttcttgtttcattgatttaacttcacttttatatgaacaagaatcttatgatgtgtatgcgctcactcttattattatggtacacgttctttctgtcaaagattactgagtataactaaccatcctttctattgacagaaacttcaaaacaaactactctcacaaaacttgtagggttgtcatcaatcaccaaaaagggggagat

>Copia12-ZM_LTR

tgaaagcatctaggcccctggttggttttagtgattaatgacaacgtaatattatatgtgactaacgtgtgttttgcagagacaaatggtaagttaggtcgcatgacaggtagaagtactacaacggtgaaaacaatcccggagataagaactcgaagcgacggctaaaacgacgaaacaaaaggtgaaggtctacggagtccgagtgtcaaggagatgtggacactcgcgatttagttaggtcttttattctcttttagccgtactataaagaggggttgtcgatgagtagtttgaccaagagagttctagtgtagtgttggtgcacaatcacactcacatacagtgctaggtgtcactctagaactcactcacaagttagaacgaaaaccgatttgaaaatcagctgaaaaacaagagttagggtttctggccctggggcaccggactgtccggtgtgcaccggactgtccggtgcaccctctgccaggtggggccaggctggtccggggaagaggcttcccctcgcagaaacccgagagcgcaggtttcagagttgaattttagtggcgcaccggacatcgcaccggactgtccggtgtgcaccggacagtggactgttcactgtccggtgtgccatgagtccaacggctagctgtcagaactagccgttggaaacgaccgttggcgcaccggtggcgcaccgttggcgcaccggtggcgcaccggactgtccggtgcgccattgcgcagtgagttgcctgtaacggctagttggtgggtgagggctatttataccccttccacccaccatattcaatgtcttgcactccacatttattccagcacattgctagagcattgcaagcaccaaaagcctagtgaggagattagagaatcttaatccgcgtttgttcctcattagcgctagcgagagccacctagagcacacaccacttgcattaggcttctcttggtcaagcgaaagtctacggcttgttactcttggtgatcggcatcacctagacggcttggtggcgttgggagctcggtgatcaccgtgaagatcttgttggtgacccgactcaagtttgtaagcggtctcgagggatccaccgcgccggagtggcaaaggatcatctcgtagtgagcacttggttcttgcgaggaccaagggggagcgatacccttgcgcgggtgctccaacgaggactagtggagagtgccgactcttcgatacctcgggaaaaattggaggagtcttctaaaccttgctttacattccgcacttaattcaagcactttacattgtgtatttgtttagcaagtatttgaagtattgtcttagcattgttgcatttctagtattatattcttagtgctagttgttggggtgaagttgggctcttgcttagctcttgcttaggttttaattagtgttgatttttagaaaagcccaattcacccccccccctcttgggcatcgtgatcctttca

>Copia13-ZM_IN

attggtatcagagccgttctcttcacgaagggactaatcgcccgaagagatggatcctaaggggaagggaatcgtgatcaacgataaggagaaggagtccttcgtcaacgagccgaaggatgacaagcctaccgactcgggctcgggccacaaacgcaaagatgggaagaagaagaagacaaggcgcatcaaggagatcgtctactacgacgacagcgatgagtccacttcttcccaaaaggacgacgacaacgactacgagaaaagaaagacggttaattcgaacttttctttcgattattctcgtattccgcaaagttcaaatgcacatttgctttccattcctcttggcaaacctccacactttgatggggaggactacggattttggagtcacaaaatgcgtagtcacttgttctctctccatccaagcatatgggagattgttgagaatggaatgaaatttgatagctcggatagtcctatgtttattaatgaacagattcataaaaatgcacaagctactactgtgttgttagcctctttgtgcagggacgagtaccataaggtgagcggcttggacaatgccaagcagatctgggacaccctcaagatctctcatgaggggaacgacgtcaccttgctcaccaagatggagttggtggagggcgagcttggaagattcgcgatgataaggggcgaggagccaacccaaacatacaaccggctcaagacccttatcaacaaaataaggagctacggaagcacgcgatggacggaccacgacgtcgtccgcctaatgctaaggtcctttactgttcttgatccacatttggtgaacaatattcgtgaaaatcctaggtacaccaagatgtcgcccgaagaagtccttgggaagttcgtaagcgggcgaatgatgatcaaggaggcgagatacgtggacgacgcgttgaatggtccaatccatgagcctcaacccattgctctcaaggcaacgaggagcaaggaggcgctacctagcaaggtggcgcaagttgaggcggccgggctcaatgatgaagagatggccctcatcatcaagcgcttcaagacggcgctaaagggtcgcaagggacagccaagcaagaccaagacaaaggggaagcgctcatgcttcaaatgtggtaagattggtcattttattgctaactgtcccgataatgatagtgaccaggaacaagggaacaagagggagaagaagaagaattacaagaaggccaagggcgaggcacatcttggcaaggagtgggattcggattgctcctcctccgactccgacaatgaaggactcgccgccaccgccttcaacaagtcatccctcttccccaacgagcgtcacacatgcctcatggcaaaggagaagaaggtatgtactcgagacaataccacttatgcttcttctagtgatgatgagtctagtgatgatgatgaaatagattattctagtttgttcaagggattggatagaactaaaatagataaaattaatgaattgattgatgccttgaatgaaaaggatagacttttagaaaaacaagaggatcttttatatgaagagcatgataaatttgttagtgcacaaaaatctcttgctttagaagttaaaagaaatgaaatgctttcttgtgaactatctacttgtcatgaaaccatttctactttaaaaggtgttaatgatgatttaaatgctaaactagaagtagcaagtaaatctaactcttgtgtagaacatgttatgatttgcactaggtgtaaagattttaatgttgatgcttgtagtgaacacctagtttcaatttctaaattaaatgatgaagtggctagtcttaatgcccaacttaagactagcaaaagtgaatttgataaactaaaatttgcaagggatgcctacacgattggtagacacccctcaattaaggatggacttggcttcaagagggaagccaagaacttaacaagccataaggctcccatctccgccaaggagaaagggaaggcccctatggctagtagtgtgcaaaagaaccatgcttttatgtatcatgataggagacaatctagaaatgcttataggagttgtaatgcatatgatgcttttgattctcatgccatgtttgcttctagttcttcctatgtgcatgatagaaatgttgctaggagaaatgttgttcataatatgcctaggagaaatgttgttaatgttcctaggaaagttaatgaaccttctacaatatatcatgcttgcaatgcttcctttgccatttgtagaaaggataagaaggtgattgctaggaagttaggggcaagatgcaagggagataaaacttgcatttgggtccctaagacaattgtgactaaccttgtaggacccaacaagagttgggtacctaagacccaagcctaaatttgccttgcaggtttatgcatccgggggttcaagctggattatcgacagcggatgcacaaaccacatgacgggggagaagaggatgttctcttcctacgtcaagaacaaggattcccaagattcaataatattcggtgatgggaaccaaggcaaggtaaaagggttaggtaaaattgctatttcatccgagcactctatttctaatgtgtttttagttgagtcgcttggatataatttgttatctgttagtcaattatgcaatatgggatataattgtctattcacaaatgtagatgtgtctgtctttagaaggtgtgatggttcactagcttttaagggtgtactagacggcaaactttatttagttgattttgcaaaagaagaggccggtctagatgcatgcttaattgctaagacttgcatgggctggctgtggcatcgccgcttagcacatgtggggatgaagaaccttcacaagcttctaaagggagaacacgtgataggtctaactaacgtgcatttcgaaaaagatagaccttgtgcagcttgtcaagcagggaaacaggtgggaggctctcatcacaccaaaaatgtgatgaccacatcaagacccttggagatgcttcatatggacctcttcggacccgtcgcctatctaagcataggaggaagtaagtatggtcttgttatagttgatgatttttcccgcttcacttgggtattctttttgcaggataaatctgaaacccaagggaccctcaagcgcttcctcaggagagctcaaaatgagtttgagctcaaggtgaagaagataaggagcgacaacgggtccgagttcaagaaccttcaagtggaggagttccttgaggaggaagggatcaagcacgagttctccgctccctacacaccacagcaaaatggtgtggtagagaggaagaacaggacgctcatcgatatggcgaggacgatgcttggagagttcaagacccccgagcgtttttggtcggaagccgtgaacacggcttgccacgccatcaacagggtctaccttcaccgcctcctcaagaagacttcgtatgagctgctaaccggtaacaaacccaatgtgtcttattttcgtgtatttgggagcaagtgctatattcttgtgaagaaaggtagaacttctaaatttgctcccaaagctgtagaagggtttttattaggttatgactcaaatacaaaggcgtatagggtcttcaacaaatcatcgggtttggttgaagtctctagcgacgttgtatttgatgagactaatggctctccaagagagcaagttgttgatcttgatgatgtagatgaagaagacgttccaacggccgcwatacgcaccatggcgattggagatgtgcggccacaggaacaattggagcaagatcaaccttcttcctcaacaatggtgcatcccccaacccaagatgatgaacaggtacctcaagtggaggcgtgtgatcaagggggagcacaggatgttcaagttgaggaggaagaagcacctcaggcacctccaacccaagttcgagcgacgattcaaaggaatcatcccgtcgaccaaattttgggtgatattagcaagggagtaactactcgttctagattagttaatttttgtgagcattactcctttgtctcttctattgagcctttcagggtagaagaggccttgctagatccggactgggtgttggccatgcaggaggagctcaacaacttcaagcgyaatgaagtttggacactggtgcctcgtcccaagcaaaatgttgtgggaaccaagtgggtgttccgcaacaaacaggacgagcacggggtggtgacgaggaacaaggctcgacttgtggcaaaaggttatgcccaagtcgcaggtttggactttgaggagacttttgctcctgtggctaggctagaatcaattcgtattttgctagcatatgccgctcaccattctttcaggttgtaccaaatggatgtgaagagcgctttcctcaacgggccgatcaaggaggaggtgtacgtggagcaaccccctggcttcgaggatgaacggtaccccgaccacgtgtgtaagctctctaaggcgctctatggacttaagcaagccccaagagcatggtatgaatgccttagagactttttaattgctaatgctttcaaggttgggaaagccgatccaactcttttcactaagacttgcgatggtgatctttttgtgtgccaaatttatgtcgatgacataatatttggttctactaaccaaaagtcttgtgaagagtttagcagggtgatgacgcagaaattcgagatgtcgatgatgggcgagttgaactacttccttgggttccaagtgaagcaactcaaggacggcaccttcatctcccaaacgaagtacacgcaagacttgctaaagcggtttgggatgaaggacgccaagcccgcaaagactccgatgggaaccgacggacacaccgacctcaacaaaggaggtaagtccgttgatcaaaaagcataccggtcaatgatagggtctttactttatttatgtgctagtagaccggatattatgcttagcgtatgcatgtgtgctagatttcaatccgatccaagggagtgtcacttagtggccgtgaagcgaattcttagatatttagtcgctacgccttgcttcgggatctggtatccaaaggggtctaactttgacttgattggatattcagattccgactatgctggatgtaaggtcgataggaagagtacatcggggacgtgccaattcttaggaaggtccctggtgtcatggaactctaagaaacaaacttccgttgccctatccaccgctgaggccgagtatgttgccgcaggacagtgttgcgcgcaactactttggatgaggcaaaccctccgggactttggctacaatctgagcaaagtcccactcctatgtgataatgagagtgctatccgaatagcggaaaatcctgttgagcacagccgcacaaagcacattgacatccggcatcactttttgagagaccaccagcaaaagggagatatcgaagtgtttcatgttagcaccgagaaccagctagccgatatctttaccaagcctttagatgagaagaccttttgcaggctgcgtagtgagctaaatgtcttagattcgcggaacttggattgatttatagcatacatgtgtttatgcctttgatcatgttccttatgcattttgttgcttacttgtggtgctcaagttgtacaaacactccccggacctcacaagtccttttgcaagtgatgcacatatttagggggagatgtgctacaacttgaccctttgagactaaccgtgtgcttgagtttgcttgatttagtctcaaaggaggtttgaaagggaaaaggtggacttggaccatgaaagacttccactgcactccgatgagagggtaacttattccaagttcatctcatgtactcttattgcctttgtattcttatttgaagattttggtgaggcaatggggttaaagggccaagattgatcccgttttggtgcttgatgccaaagggggagaaaataaaggccaaagcaataaatggatcagctaccacttgagaaattttgaaaatagtagaatagagcttttggtttgtcaaaactcttttattgtctcttttgtcaaaagttggcctcttgtggggagaagtgttgattatgggaaaaagggggagtttttgaaatctttgatcaatttcttttggaatacctctctttatgtctctacaagtgtatttgacttagagataggaatttgagtttgatttgcaaaaacaaaccaagtggtggcaaagagtgatccatatatgccaaatttgaatcaaaacaattttgagttttcatttgcattgatattgcacttgttctagttgctttatgttgtgttggcataaatcaccaaaaagggggagat

>Copia13-ZM_LTR

tgaaagggaaatgtgcccttgggccatttctaagtattttggtgattgagtgccaacacaagtgcttttgtgttaatctatgcaaagtggtggacaaagtgcaaatcaagtcaaaaggtatgtttctagacttagtacattgttttatggactaatgtattgtgtctaagtgctggaaacaggaaaaattgaattggaaaagagatggctttgttcagccaaagtctgctcggtctgggtgcaccggactgtccggtggtgcaccggacagtgtccggtgcgccaggctggctctggcgaactggctgctctcgggacttcgacggcggtgtacggctataaatcaccggactgtccggtggtgcaccggactgtccggtgagccgttcacaggcgaactcgtcgctctcgggagatgattaacggcgtacggctaaaattcaccggactgtccggtggtgcaccggactgtccggtgagccaacggtcagccggggcaacggtcggccgaggattccgcgcgtgacgcgtggccgagccaacggtcacattggtgcaccggactgtccggtgtgcaccggacagtgtccggtgcgccaacggctctcaatctgcaacggtcggcttcgccaaataaggaaggaaatccgcaccggacagtgtccggtggtgcaccggactgtccggtgcgccagtcgacagaaggcaagatctgccttcctggaatgctctcaacggctcctagctgccttggggctataaaagggacccctaggcgcatggaggagaacaccaagcattctctaagcattcctaagcaccaagacttcgattccacgcatttgtttctttgtgatagcatctagagctctagttgagttgtgaactcatcgggttgtgttgcgagctcttgttgcgacttgtgtgcgtgttgtcactctgattttgtgtcttgtgtgcgttgctcatccctcccttactccgtgcttctttgtgaacttcaagtgtaagggcgagaggctccaagttgtggagattcctcgcaaacgggataaagaaaagcaaagcaaaacaccgtggtattcaagtgggtctttggaccgcttgagaggggttgattgcaaccctcgtccgttgggacgccacaacgtggagtaggcaagcgttggtcttggccgaaccacgggataaaccactgtgccatctctgtgattgatctctttgtggttattgtgttttgttgagactcctctctagccacttggcaattattgtgctaacacttaaccaagtttttgtggcattaagtttcaagttttacaggatcacctattcaccccccctctaggtgctctca

>Copia14-ZM_IN

attggtatcagagccggttaaggatttcccttccctaatcggttcgaaatccacgtaggcgacatggaacgaggtgttggcaaacctccgttcttcgatgggaccaattacccgtactggaagattcgcatgtctgcgtatcttcagagcatcagttaccgggtttgggaaatttgcctcgatgcgattttcgatgctacaagtgaccggatcactccaattcaaatggagttccatgattcgaacaacaaagctcgaaacgctttgttctcgtgtctctcgcttggtgagtttgagcgagttggacatctgactacggctcatcagatctggtctaccctcgagagattccatgagggcaacgatcatgtgaagaccagactgtttgagacgtacaggcgagagtacgagaacttcacacagttggctggagagaccattgattctatgttctccaggttccagtcaattgtgaacaagatgcgtgccaacaaggcacagctaccctatagtgatcatgagagagcgctgaaattactacatgctctagatcggagggtttgggaggtgaaggtctcggcgatcatcgagtcgcccaactacgagactctcaccgtggacgagctcttcagcaagctcaagtccatagagattgaccaccagactcgggccaagattgagaaccctagtgcacccaccatggccctggtctctggaggtggttctgcttctaactcctcacctgctatgtttgctctatcttctttgttgtctattacagaggagcaggtggagagccttggggatgaggagctggcacttgtggccagccggttcacgcggttccacaacaaccgtatgagccggcggcgtggcgggtccaaggacggatgctacaactgtggcgatcccgaccacttcgttgccagctgccccaagaagggcaaatcggagtctggcccgcgcgaccatcactctggtcggcgcaagggcaagtactcctccggcaagtacaagtccaagggaggattcaacaaggaggcgctcaagaagaagtaccttcagaaggcaaagatcaaggagcgtgccttcctcgcctccctcagcgacctcgaccacgactccgacgatgttgtatcttcctcgagcgacgaggagactgagaggcgggtcgaggacaagctgaacgggttgtgcttcatcgccgacaccgcaggaggcttctgcaccatggcacttggtgaggacgcggtcggcaccagcgacgacaaagacatcggcgacgacactacttctgaggtactaccttccgccgatgatctcgccgctgagatagaagagctgaacgctgctttggctagtcaggataagttgcttaggcaggctgctcgtgagaggagagagtttagatccaagtacgagagcacgcttagggaacttgagtctgctagagcttcagttgaggtgtccgacgagactgaatgtgatgagtgcgctctacacatgtcgaacatcaccactttgcagaccaagtactccaccttgctagatgagcgcgacgagttgagatctaggtctatcctgttgggtgcgtgtaccgtttgtcctrgcttgcagtctgagctagcagagagagacgctaggatagctttgcttgagaaggctagctcagtgagtgcccctgcacctgcgcagtgtgcactttgtgagggtttgcaatctgcccttgagtcctgcagacacgacaagacaagaatcgaggaagaaaacacataccttcgatctatcttgagttgggtgtcgtgcagtgagccccagttgggcatgatggtcagtcagttcaagaggggaactggcggaccggggttaggctttgctactaaggatgggagtgtcgctcgctttgggaaggttggtgagtgcagtggtttgacaccttcagagaaaccttcacccactcccaagcttatcaaaaccacctctgctaagcctatcacccctgtgagagatggtgtgattgatgaacctatgagagctcctcctcagaaacaagtgtggcttccaaaacctaaccatcttaggaacacacttgacacctttcccgacatctctagcgatccccttcctagagcccctcagccatccaaaaagaaagccccctcccacaaacaaaatccacccaagagagaggtgaggtaccattgtgaatattgtgagagggatgggcatctagcttctttttgctttaggaggaagagagatgagcggcgggtttctgagtcgagcaggaaggacatgaaccgcccctctcatggtgttcatgctcagcctgttcagagacgtcctgsgaggcctagaggtgttttgcctcttgccgctaggcctcaggcagtgagaccacgtggtggtcgtgcccgacgggatgctggtcgtgtgccatatggccaaggaccatgtgacagaggctttggttcatacttccccagcggaccacaatttccttcttgtggtgatcgcttcccttcgggaccggggatgtttggtgttttccctaacacttttcaggggcaaatgccgcagcactggtattcttcacagtttacttaccccagtgttgtgccatttgctcaccccgtgtcttactattgatgcaggacggaggcctggagaacacgtggctcatggattccggttgttcgcgccacatgaccggaagctcaaaatggttctccagcctcgaccccgtgattggtaaggaatacatcacattcggggataagtcaagaggtaaggttgtttctcgtggcaccattcgggtgaacgagagctttattctcaaggatgttgctttggtttcgaatctgcatttcaatctgctttcagtttcgcaactccttgaggatgactatgaagtgcgctttaaaaagggcttgtctcgagttttggatgcccgtggggatcttatttgccagatttccccttttggtcgagttttcagtgctgatttttcacattcttctggcccttctcgatgtttgttggcaggatcttcctcttctctttggaagtggcataggaggctaggtcacttgagctttgatcttttgtgtcgtttgagctcacttgacctcatccaaggattgcccaaattaaaatttgagaaagatcttgtctgtcacccttgtcgtcatggcaaaatgattgccgcttcccattccccggtcaccaaggtgatgacctcgcatcctggcgaattgcttcatatggacactgttggtccagccagggtttgctcttttggagggaagtggtatgtgcttgtggttgtcgacgacttctctcgctactcttgggtgttctttatgacggcgaaggatgaggcttttactcatgctcgagatttgattcttcggttgcaaaatgagtttcctaaaaatgccatgagagctatacgcagtgacaatggcacagaattcaaaaatactcaatttgcaaccttttgtgcttctttgggactcgagcatcagttttcctctccgtatgtgccccagcagaatggtattgttgaacgcaaaaatcggacccttgttgagatggccaggacgatgcttgatgagcataggactcctaggcgtttttgggccgaagcgatcaacactgcttgccatgtgtccaatcgcatctttcttcgggcttttctgaacaaaacttcttatgagctgcgatttggacggccacccaaggtcagtcatttcagggtatttggctgcagatgctttgtgctaaaacagggtaatctggacaagtttgagtcccggtcttctgatggggtttttctgggttatgcattacattctcatgcttatcgtgttcttaacctagagactaaccgcattatggagacttgtgaggttacattcgatgagactgcaccttgtccatcccctgtctttgagcctgcaggtccagatcagatgggacagaccatctttgtggaggaggagcacgacgacgccgattgggttgctctcgagccaactccaccggccgccccagtcgagcctgcttccactactacggctgacggacccgaccccactccttccaccacttggggtccgctcgagccagctcctgctgagactggaggagttgaagctgctgttgagggggaggccacttcctcaagggaggctccacagcatattcagcgccgtcacccacctcagcagatgatcggggagcttcacgagcgagtcactcgctctaggtcacaacacatttctcattttgctcactcagcttttgttgctacttttgagccccgagatgttggacatgctttatctgatcctaactgggttaatgctatgcatgaggagcttgaaaattttgaaagaaatcaggtttgggttttggtgccacctccttctaactgccatcccatcggtacaaaatgggttttcaaaaacaaacagagtgaggatggtttggtagtgagaaacaaggcgaggttggttgcccaagggtattgccaaaaagagggaattgattatgaggagacctttgcccctgttgcccgtttagaggccattaggatccttttagcctttgctgcttcaaagggttttaagctttttcaaatggatgtcaaaagtgcctttttgaatgggtatatagaggaagaggtatatgttaggcagccccctggttttgagagttccaagtttccaaaccatgtttttaaactccaaaaagctttgtatggtttgaaacaagcccctagagcctggtatgagcgtttgaaatcttttctgctttctaagggttttaaaatgggctctgtggacaaaactctttttctcctcaagcataataatgacacccttttagtccagatttacgtggatgatatcatctttggtggttcttctaatgctcttgtgtctagattttcagatcttatgagcagggaatttgagatgagcatgatgggcgagctgaatttctttcttgggttgcaaatcaagcagactcaagacgggacctttgtgcatcaaggcaagtatacaaaggacgtcctgaagaagttcgacatgggcgagggcaagcctctttcgacgcccatgtccacctcgacgacacttgacactgatgaggatggcgaacccgtggaccagaaggagtacaggagcatgattggctctctcctgtacttgactgcgacgaggccggacatacacttcgcagtgtgtttgtgcgctcgtttccaggcttccccacgcacatctcacagaaaggctgtgaaacggattatgaggtacctacgtttcactcctgagtttggtttgtggtactcctcatcgtccgttctgtccttgtgcggttattctgatgctgattttgcgggttgccttttggagcgcaagtctacttctgggacttgtcagtttttgggtacttcgttggtttcatggtcttctcgaaaacagtctagtgttgccctgtctaccacagaggccgagtatgttgctgccgctagctgttgttcgcagttactttggatgatggctaccttacgggattttggcttggattttcatcatgtgcctctgctttgtgatagcacgagtgccataagtgttgccaagaatcctgtgctgcattcgaggaccaagcacattgatgttcgctttcactttcttcgtgatcattatgagaaaggcgacatcgaattgcgctatattgataccactcgccagcttgctgacattttgaccaaaccccttgatcaaaaaacttttgttcatttgcgaggggaattgggtgtttgtttccctttttgattagagggctccctttttggtcttttcttcttattttctgctttcactttcttttgtattatatctgcatgtcatattgagcttcacttgtataactgctagaatgtgattatgctcctagtagtatgttctttgtgtacatgataatgctatggcatgcttaggctctttttgctcatattgatacaatattgttgagctaagttgttttcataattgtgaacttgactattacttgctcaatctaaaattgttcaccattaagattggcacctagcatgtgtaggatgtgttgaaatcctttttgctattactagtatgctaggtggctatgtctcatgctttgagtcattcacttgtcttgatttattcatatcgtgctaaaaattcaaaatcaagataagtgataatcagaaagatcgagatgggtttgtatttgtcaccacatcactgtatcgagactgcttccatttgcacttttggatgaacttgagcagtgtagtggacgatcgaaaccggtgtttttagcttctgattgcttgtctgcataggctacacccggttgaaaagtyagacaaagacaatgcttacaacctctcgcactcacatgcttttcaatgaaatttggaatccgctgcaaaatccgataaatcttgaaaatttcaaatggtatctatttcagatatgtttctagcatgatgagtgatcactgtttggggtagttcaccttgttcacttgtgactggcacttagttgatttcctgcttactatatgcttagtgttgtctttgtggtgaacatctctttgaaatgcttaaaactgaatcaaaatacttacacttcacatatcttgtgcatatgatcagtttgagcatctgctagcacgtgcatttattgctgcacacttagcacaacaccacttagagcattttttacactctgactggtatactcctgaagctcatgcattttctctatttcatactctytgttgcatttctgctttttctcaggggaggttctcttgtcaagggggagtttttctttctacccctgtctgtgcttgatgagttcacttgtcaacaagggggagagattttgagatttcccgtttcctctatctagggggagaattttgtctttcgggcttcctctcattcaagagggcgagatatgttatttggagctattgctagttgtgttgagccgtttgcctcyttctggaggaactagctttgattttagcttttgtgctgattttatgctctgaattggttttttatgctctgataaagccttttgtgctccgatgagattttggcttctggctagtggtgttgaaccctgcttttgcctcttattgagggctagccttttcttgcttggtcgtgttgagccgttgcccatttctttggggaccaagttttgcttactctaaatgacttgtctggcttwtggtcatttgagtgagtttctcttcttcttcctctttctgtttacatatgctcawatggtggtgttgacaatgcactcatcaagggggagat

>Copia14-ZM_LTR

tgcgaacacaaggttgacttgtcccttgtggttcgggttgatgatgagtgattgtcaatgggtagcactctggggtagtcagtggactgaccagagtgtgagattatgcttgtgcaaccatgtcggtcgacttgtggtgtgcaggtgtggagcacaggaacggtggtcgatggctgaggtgaaggtcatgcgggctcgtgctgatggaccgggagcggtgaagggcgagtgctgggtcttgactgacggatcagagcagccaggtgaccaaccgcggtggctgacacctgggacacgcacttgtgcgaggacgtggtggagtggaccgtgttgccggcgtggtcgaggactggcgggacacgcgtctgatcgtggaggacgtgcacgaggtgcggtgctcacggcaggtttggtggtttgggcctcaaaaccacccagcgctacggatggcgggttttgctgagtttgggcctcaaaactcggcggtggcggttccggagggaaccggtggcggcacgcggcgtgatcgcggagggtgcgtcaaggcgaagcaactccgtgtgaaggacgtggccgtcggatcgaaaacctaggagttggtccattttcgcccccggtggagtggataggctctatgtaaataggggtagtttaggaagtgagaataaccctctataaatagaggggagggctggttagttcagcatcccttggctgccatttgttttgagcactcattctagagctcctagttttctctctaggtagacaccagttgagccgagggttccgcagcgattttgtacttcgactgtgttcttgattttcaagtttaatcagaggaagggtcgccggtccggcccacagggctctttggcttcaaatctcagtttgtacccttgctgaaatttctgaagttttatagtcttggtttctccttttcccgatcttgtgttgttggtgaaaaactttgattcctttgagaggatttggtttggggattgattggtggtgagttgcactcttcggaccactgcatacacctagttgttgcagattttggtcgcgatttgagaaaaactcagtttgaactcgtttctagaaaaccccaacaaaaccccaatttctgctgtatttttaaatctgcgggtgattcacaagtccgattgagctcaaaatttggggagatcttggaaacgtatttgctcaggtgtgtgtaaaatttcatctcaatcggagttcgtttggttcagttttgaattcaagaacagaatttcgtactgctgaaaacaacacttgttgacgatactgtattggaccgttttggactttttggtgtccgatttgcgatccgtttgttttgctggtctcatgtgagtattaggaacgttttgtaatcatgagatcactcgttcgctgatgtgtttatggtttgagcacttgcttcatctcaattgaagtgaagtgttcattttcagtgtttggaaacaaaaatctgattggctcccattcacccccctctggtcgccttaccggtcctaca

>Copia15-ZM_IN

attggtatcagagcttcgtacctcgttttacgcttaaccgcgtgaggaaacgatcatgtctactcaacgggaccatgtggatcctctcctcgaggaaatccccatcacatcttccggtgaggaagtggaccccaaggtcctcgacctcgccatgaagattgccgagagaatgttcctcaaaatgaaagaggaagatgctaagaaaaaggccgaagaagaagaatcaagaagaaaggccgaagaagataaaggtaagggaaaatttgattacaatgacgatctagtggatcttttggtgtctaaggtgttgagcaaggtaagtctcaacaccgaaggatcatctaccaaaagcaaaggtaacgaatttagtaaagttcaattcgattactctagaaattttattcccaacttctcttccgccccacttggaaagttaccaactcttagtgagttgaactatgacgagtgggccgacaagatgaagtcgcatttaatcggtgtgcatcctagtctttgggagattgttaatgtaggtatgtataagcccgcccaaggagaagagatgactccggaaatgatgcaagaggttcatcgcaatgctcaagcagtgagcataattaaaggaagtctttgtccggaagaataccggaaagttcaaggaagagaagatgcccgtgacatttggaatattcttaaaatgtcacatgaaggagatcccaaagctaagagacatagagttgaagctttggagagtgagcttgcaagatatgattggacaaagggtgagtcgcttcaatcactctttgatcggttgatggtgttagtaaacaaaataagagtgcttgggagtgaagattggagtgactccaaggtcacaagattattcatgagagcatataaagaaaaggataagagtcttgcaaggatgataagggatcgtgatgactatgaggatatgacgcctcatcaattatttgcaaagattcaacaacacgagtccgaagaagcccccatcaagacaagagactctcatgccttgatcacaaatgaacaagacaaccccaagaagaacaaagaccacaaagcaaagaaagtggtcgagacctcaagtgatgaagatagctcaagtgatgaagacacagctatgttcatcaaaacattcaagaaatttgtaaggaaaaatgacaagttccaaaggaaaggaaagaagagggcatgctatgaatgtggccaaaccggtcatttcatagcggattgtcctaacaagaaggaacaagaagccaagaaggaatacaagaaggacaagttcaaaaagggaggcaagaccaagggatacttcaagaagaagaaatatggtcaagcccatattggtgaagaatggaactccgatgaagagagttctagctccgaggaagaggaagtggtggcaaacgtggccatccaatctacatcaagctcgcaactcttcaccaacctacaagacgactcctacactccaacttgcctcatggcaaaaggagataaggtaaccttatttagtaatgattttccaaatgatgatgatgatgaacaaattgccatgaaaaataaaatgattaaagaatttggcttgaatggatacaatgttatcaccaaattaatggagaagctagataaaagaaaagcaactcttgatgctcaagaagacttgcttatccttgaaaaggaaagaaacctagagcttcaagaattgcttcacaataaagatgaaatgctagatgtcttgactaaggaagtatctttagtcaagataactatagagaataaagataaagaagtaattaatatgaaaacctctatagctaatcttgcaaatgaaaagaatgcacttgaatcaagcatgttaagcttgaatgttcaaaatcaagaacttcaagtgcaacttgaaaattgcaagaacatcaatgcctcatctttagtgattgaatctaagtctagctcctcaaatgataatttttgcaaacattgtgccaaatatcatgcttcttgttgtctaactaaccatgcaaggaagaatagcccacaggtgaaggtcaaagaaattttgaaaagatgctctagcaatgatgggttaaagaaagttgaacccaagtacaagtccctaaagcccaacaatggaagaagggggcttgggttcaactcatccaaggaaaaccctagcacagtgcataaggggtggagatcccccaagttcatagagggaaccaccctatatgatgccttggggaggattcactcctcaaatgacaagtcacctcaagtaaaggtaaacttgagttccacaaagagtaagatgaaggaagtgggatcctcaagtggacaaaaatttaatgctcccatttctcactcttatctttgtgattatatgttgacttgggatttagggaaattggttgtcaaatatgtgggtgcctacactaaaagaaaagtcatgaaaagaagtgtgtgggtacccaaggctataactaacactgtaggacccaattcaatttgggtacctaaaagcatagcctaaacttgttttgcaggtctactcctctggtgggtcaagttgggtgcttgacagtggatgtacaaatcacatgaccggggaggaagacatgtttcattcattgcaactaactcaagaagcacaagaaattgtgtttggagatagtggcaagagtaaggtgattggtattggtaaaattcctatctctgaccaacaatcactttcaaatgttttattggtagattctttaagctacaatttgttgtccgtttcacaactttgtggaatgggttataattgtttattttctgatgtggatgtgaagatccttagaagggaggactcctcagttgcctttacgggtcgcttgaagggcaagctttatcttgttgatttcacaacaagtaaagtgacgcctgagacttgtttagtggcaaagtccgacaagggttggctatggcatcgccggctagcccatgtcggtatgaggaatttggccaaacttcaaaaggataatcacatcattggactaacaaatgttgtatttgagaaagatagggtttgtggcgcatgccaagcaggaaagcaacatggagtcccacatcaatcaaagaatgtggtcacaacaaagaggccattggagcttcttcacatggacctcttcggacctgtggcttacattagcattggtggtagtaagtatggtttagtcattgttgatgatttttctcgattcacctgggttttctttttgagtgataaaggtgaaactcaagaaatattgaagaaattcatgagaagagctcaaaatgagtttgagctcaaaatcaagaaagtgagaagtgataatgggacggaattcaagaacacaggtgtcgaagaattcttaggcgaagaaggaatcaaacatgagttctcggttccttacactccacaacaaaatggtgttgtggaaagaaagaaccgaactctaattgaagcagctagaaccatgttggatgagtacaagacacctgacaacttttgggcagaggcggtcaacaccgcctgtcatgcaatcaaccgtctctatcttcacaagatctacaaaaagactgcttatgagcttctcactggtaacaaacctaaagttgattattttagagtatttggttgtaagtgttttattcttaacaagaaagtcaagagctcaaagtttgctcctagagtggacgagggcttcttgcttggttatgcatcaaatgcgcatggatatcgtgttttcaacaataccaccggtcttgttgaaatagcgatagacgtgacatttgatgagtctaatggctcgcaagggcatgtttctaatgacactgcaggaaatgaagaactaccttgtgaggccataaagaaacttgcaataggtgaagtgagacctcaagaaaaggatgatgaggaaggaactttgtggatgaccaatgaggttgttgatgtgggtgcaaaggtggtgggtgacaaatcctccacccaagcaaacccatcaacctcaagtcatccaagtcttgaagaaaatcatcaaccccaaaggatgccaactgtggtagaagatgaacacgaaagtgttgatggtgaagtgcctcttgatcaagtgaatgatgaggaggagcaaatacaaagacaaccatcagtgcctcatcctagagtccatcataccattcaaagggatcatccggtggacaacatcctgggtagcatcaggagaggggtaacaactcgatctcgtttagctaatttttgtgaattttactcgtttgtttcctctcttgagccacttaaggttgaagaagcattgggtgatccggattggataattgccatgcaagaggagttgaacaacttcacccggaatgaagtctggtccttagtccaaagacccaaacaaaatgtgattgggactaaatgggtctttaggaacaaacaagatgaacatggcgtggttacaagaaacaaagcacggttggttgcccaaggctacactcaagtggaaggacttgattttggtgaaacatatgcgccggtagcaaggttagagtcaattagaatattaattgcctatgctactaaccatgatttcaagctatatcaaatggatgtcaagagcgcatttctaaatggaccactacaagagagggtctatgtggagcaaccaccgggctttgaagatccaaagaagccaaaccatgtttatctacttcacaaggcactctacgggcttaaacaagcccctagagcttggtatgactgtcttaaagattttttaattaagaatgggtttacaataggaaaagctgactctacattatttactcgcaaagttgacaatgaattatttgtgtgccaaatatatgttgatgacattatatttggtagtactaatgaaaaattttgtgaagagtttagcaaagtaatgacgaacaggtttgagatgtctatgatgggcgagcttaaatacttcctgggatttcaagtcaaacaactcaaggaaggtacttttctatgccaaaccaaatatacacaagatatgctcaagaagtttggcatggaaaaggcaaaacacgccaagactccaatgtcatcaaatggacatctcgacctaaatgaggaaggtaaacctgtagatcaaaaattatatagatcaatgatagggtcactgctttacttatgtgcatctagacctgatatcatgttgagtgtttgcatgtgtgcacgttttcaagcaaaccctaaagattgccatcttgtagccgttaagagaattctaagatacttagtccacacccaaaacctaggattatggtatcccaaaggctcccttttcgatttacttggctactctgactcagattatgccggttgcaaagtagatcgaaaaagcactactgggacttgccaattccttgggcggtccttagtgtcatggagttccaagaaacaaaattgtgttgcactttccactgcagaagctgagtacatagcagctggggcatgttgtgcacagttgttatggatgaagcaaacccttagagattttggttgtgagtttaacaaaattccacttttgtgtgacaatgagagtgccataaaacttgcaaacaatcctgtgcaacactctagaactaaacatattgacatcagacaccatttcttgagagaccacgaagccaaaggagatatcgaattgtttcatgtgagcaccgaaaatcaactagccgatatcttcacaaaaccccttgatgagactaggttttgctttcttaggagtgagctaaatatcttggattctcgaaacgtgtcttaaaaactaaaattttgatcaaatttgatggatttaaattgattgtttgaacaatatgtcatgagaaatamcatttttatcattaaaattactcatgtcatatttgctaagtgttattatagcccttccgggcaatatctggatcaactggttgaggccagctaaacttcggctgagtaggccactcaaccggttttcccctggtggaaaccggttgaaccggtataaaaaccggttgaaccggttttgacctccgcgccgtcagtctgcgcgcagtctgcgctgacagctgcgcagtctgcgccgtcagtttcgcagtctgcgctgtcagtctgccagaaatttcgcgcagtcaacacctttttctgcgcgcttttactgcgcagtctgaccagaaaccggttgaaccggttttwtaaccggttgaaccggttttctgtcttctgaccggccgactcctccccttttatttctctccctctctcctcttctcttcttctcagtttcattttccagccgccgccgcccactctttttcctctctctccttcacactctccactccaaaaacccacttgtgccctcacatctttggaagatttgttggagatccgttcatctcggcgttctccatcatctccctcaatcttgttgggatttcttgattcaatctccggatcgaggtattgccctatttcatttctttttactcgatcctacgcgttcttgattatctagtgtatcattcgttgggtacattttagtttaagtgcttgcaacacttagattatcttcatttgtcacgaacattgttagagtttagcggtttgatattgttcgttgtagttgaaccgctcatatgaacggttgaagtgcatgctcaatatcttatgcgtctctactcaaacgtgtttgttaagatccttgctcatcatamccggttctattttttammactttttaaggatggtgcgtcggaggaatccttctgtggttgaatctagcgactcctctgacgagatccaagaggaattcatggagacctctactccccctcgtcgctcccgatccaagcgtgggcgaggatctggcaaygtcatgcaaggtggtgaggcctccgggtctcaaggagctcgcggtcggacaacccggaatccctccggtcggtcccgacccgcgacgaatccgcaggcttgggagcctacaagtgatgatgatggggggaaggatgatgaaattgaycttcccccggcccacgctctctatgattcgagggttggtcctccaccgggcggaggctcggcgtgctggcaatgagccagtcactgatttcactgccagtggaggttctgcactccttcaggatctccgtttccagaatcctgcattgcgcattcgtgatgccaggatcgatggcaatcggttctggactcttcatcatgtggatttctacaattctgtgattcttcccaagaaacatcaacccatcctgcatcaacgctatatcaactgggaaggwtgtgaggctattggagatccagagatgacacaggctttgagggcctgtgagagaaagaagatgaagaatattatgacattccaatatgattggaatgatgaagtcattgctcagttctattccactctctggatcaagctagctgatgaggaaagtcgatgggtacaactatccttatctgaatttctacatcgaaggtagctggtacaaggtgagctaccgtcggtttgctcacattcttggcttctctgataatgatatttctggcgacaagatgcaagattcatgattttcggcagcccaccagagatgaagccaaagatcttcatctctctgagtctgggaagttttgggagtccacwaacatgcataagtattacagatacatcaactctctctgcaggatgaccctcattccaaaagggggtaatcagatgaacattcttggagagagcaaggtcttgctctcattcatgaagcccaacagctcagagagcatcaatgtctttgacatgatctggcaggaaatcatccatgctgcctgctttcctctgaaggggtgtcttcatgcaccctttatcatgaagatgattgaagtkgtgacccagttccgatttgaaaaaggtaccagacattccagtcatatacccctttctggattgatcccaacaatccagcagggcgtctgaggaaagccccctccagctctcgtgcacatacctcttccactgagcgggttcgtctgctggtcctgatcctgctgctgctgctgccgctggtgcctcgcgtccctcccctggccgcggatctcccacgccacgtggtcgtggtcgtggtcgaggtcgtggccgagggatgggtgcccgcttggcccatgggtttgcggcatttttctccatgtgccggaacatttctgctgatgtccatgaggtggcacggcgtcagcgggagactgacgacaatcttcgtcgtcaagcttccwctatgggtatgccctttgcccctcgctcgcctgacgtgcctctccatcctcctcccccggagatcaatgagtggcaccagcaggcctacggggtgccttttatgtcagcagacgacgacgaagaagaagaagcctattttgatgatcgtgagcagtttgccccgcctccctaccacggggatccgggtcaatcatcctctcaccctccgcctccttacccgggtccaggatccttctggcagtgctcctcccccggatcagtctggggaagaacactttgcctctcacctggcgcaccacttctttgctcctcatcatcctcctcctaactggtgatctttggattggtgkctctctctctctttttggttcttgttgccaaaaagggggagaaggtttattatctagtgcagtacctttttatttttgtaatgaacaatcggtttgtaataactattgtgatgcatgcatggctttaatgccataaacatttttatgatgtgatgagatgagttctcattattgtaatagccgtagtaatttttcatatcactatgctatgcaataatgagcttgaatgtgtaatttttactctttctatcataattatgttgcatccctcattgtttttgtgtatggtgttgattgttctactctctctaaatatgttgcaaatatgacatatcaagtcaaaatgttaatatcacaaaactcatgcacatatttagggggagctacctacatacaatcattcaaaagaaattttatctcgcaagtacttgtaacttttaatttttatttctactctagtttgttttggcatcaatcaccaaaaagggggagat

>Copia15-ZM_LTR

tgtaagtgcaatcaaccctaattgagggttttggtgattaaatgacaaaacaaacagagattctaacaagtttgctcctagcatgtacacagtaattctacagacaggagaagcacagatcttacgagcataaaaatataaaagcacagcggatttaaaattctacatcaaatggcgtgctccaagtgcttagaaacaacggcagtgctcaatttataattcttgagttataggaatcgccgtgcaattaagagggatccgcaatggttaagtaagttatgcaatgagctaagttcaaaatcctttgaaaacatcttggagaacatttttccagatcatgaatgcccttgagaaaaaccaattttacttcccacaaaaactccacctttgttctcttcaaaatactcaaaatttggttttcagccctcaaaaccggttcaaccggtcctgaaaccggttcaaccggtttcgggactgttcctctgccatctctgctctgacctgtctgacagtcagagctgtcagaaaagtcgctgcagatatttttggaaaaccggttcaaccggattttttccctactcaaccggttttgaatccggtcgagtctccggctgagtaggtcagtgaaccgggatctccctggtagaaaccggttcaaccggtctgaaaaccggttcaaccggtttttaccccttttctcccaacggctgccagcttttgggggatcctttatataccccctcacactctctctcttatttacttctgcctaactcccacgaattcttggctgaccaaacttcaaacaagtgcattcatttcacctttcacacccgcaatcgcatctccttcaatcatttgaaggagcccttgggtgtgaggtgaactcgatcgaacacgctgtcaatttcatctcgattctcccattctctttgttcttgagctcgttgcaaacttaaccttgtgcggatttgttactcttggaacctcgtgttccttgacggttagaggttgcttgggagtctccaaatttgtggacgaccccaagaagtttgtatcacccgctctttgagctwatttgagaagagattgccttgacctttgtggtcggcttgtggaggattagggttggaaaagacccggccctttgtgggctcctcaacgaggagtaggacacctttgtggtggttgccgaacctcgggttatatcgcgtgttcttgtgtgtttgcttgttaagcgctttaaatatcgttggttgcaaggttcatattatctattcgagtagattttgtgtaggagcaaaagtctttagctatattcttcactacttgcgtaatatttgtttaacagattttaatctaaagtttaagttgagcaggttcaaacttgttcagcttgtaaacaaaatcgactgaaccggtttgcaccagtgcaactttatatttaaccatatttcgaagtttttagtgaaaattttcaggtgtagcctattcaccccccctctaggctactttca

>Copia16-ZM_IN

acttggtaatcagagcattcgatccattttttttcccccaccccgcccatcgtccatcgctgctccagtatttatggctctcaccaattccaccttcccaggcaccattgcctcctcctccccgagcctatctctttcttcccttggtcacgccatcaccgtgcgtttgactcgggagaattttttcctttggaaagcccaagcttccccggtgctgcgtgctcatcagcttttcggctatgtggatggttccatcaaggctccgtccgagttcatcactaccggatctggtgctgaagcacgtcaggtcccgaatcctgcgtatttgctttggtatactcaggatcagcttgttctgagcactcttgtctcctccatgagtgaagacatgctgggccagatgacacagtacaagaccgccgtcgatctctggacggctctgcatgagatgctttcttctcataatcgtgcacagattatgcaagttcgctatcaactctccaataccaagaagacgtcggacatgactgcttctgcttattttcagcggatgaagagttatgccgacaccatggcatctctgggttatccgctgaatgatgaagagattcttggttacatgctcgctggccttggtgccgagttcgagcctcttgttgctgctataactgcccgtgatgatcccataagtctcaccagttttttttcccagctcctcagtgcagaagtgcgtctctgtcggcatgctccgaccactgaaatcctgtcctccgccaacactgcaactcgtcagccatttactcgtggaggcagccgtggaggacctttccgtgggcgtaaccagggtcgtggccgcggtggccctggtcgcggccattctcatggtggcgtcaaacccacctgccaggtgtgcggcaaatatggtcacgatgcgctgcgttgctatcagcgtttcaatcatgcttttcagcctgaagattctcgcagccgctctgccaattctgcaaacactggtgcatacaacattgacacagactggcttctagatagcggggccaatgatcatttgacaagtgatcttgatcgcctaaccacacacgagcgtttttctgggaaagacactgttcaggttgccaacggttcaggtttgtcaatttctcattttggtcaatccttgctacccggctcctctcgtcctctctatcttcgtaatgttctccatgttcctggtttaagcaaccatcttttatctgctcaccgattagcttctgataacaatgttttcattgagcttcatccaaattttttctgtgtgaaggatcgggtaactcagaaaacgctccttcgaggtaaaagtcataatgggctttatccggtgccatgctccccacattcggcgtcccccccaagtcgattaggccttactgctgtcatggcgtccacagatctctggcatcagcggcttgggcatccctcgtttagtattgtgtcttcagttcttaggtccaataaactagcatgtgcacctagtcagtcgtcttcagtttgtgattcgtgtcagcgtgccaaaattcatcagttacctttttataaatccaatcatgtttcatcctctccactagaacttgttcactccgatgtatggggtcctgctatcacctctgtcggtggttttaaatattatgtgagttttttagatgattatagtcgatacacctggatctatctccttaaacgcaaatctgatgttgaacatgccttccatctatttcagaaacatgttgagcgcttgctcaatgcaaaaatacgtatcttccaatccgactggggaggcgagtatcaacgtctctctcgtcatcttgcctccaccggcattcagcatagagtcacttgtcctcatacttcccaacaaaatggtattgctgaacgtaaatatcgccatatagtcgaaactggtcttgctcttcttgcccactcctctctaccggtcaaattttgggatgaggctttcattactgcttgttatttaatcaatcgtatgcccacacgtacacttcataattccactcccttagaacttatattccatgaaaaacctgaatattctcttcttcgtgtttttggttgtgcctgctggcctaacctccgtccatataacaatcacaagctatccttccgctccaagcaatgcgtttttctcggctatagttctatgcacaagggctacaaatgtttagatcgttctaatggacgcatctatatttctcgtgatgttgtttttgatgaacatctgtttcctttcgcatcttctaatccttcttgtgagccggatgcttatgttgatcatatgagaaattatagacctgaattattggcttctaatctgtctgtcgagtgttctcctgtgtctgtttcagatgcatcgtcttcggattcttcaaatcacgcatcggcgccctcatctcgattggatataattccagctgcagtatgcactccaatacgatcagatactgatcataattccttgccatctgctgctttgccgatcacatcctcagacatgtgcacagctgcgtgcccatcgcataaccatccaacattaaccagcccaccagctgctacttcttccagcccgccagatgctacttctccactgccagctgctacttcgcccagcccgccagctgctacttctccactgccagctgctacttctccaccatctatgctgcgcatgcatgaggacacgaacacaaatcctgctgaaacctatccagcagccgtacatgcacctcatgtgcactcgcctgacgcgttgtctgctgatgcgcttccgcctgctaccgagtctgctgccggccctacagacttggttatttctaccacggctccaacttcgaccattgtcactcgccttcgaaacaacatcagcaagcctctgctgcataccgatggaactattcggtataatccatcaagtcgaagttttggcttatctgctgttcctatgaattatcatcatgctctagccaacgaaaaatggcgccttgccatggatgctgagtatactgcactgatgcaaaatggtacatggtccctggttcccaagccatctggacacaacatcattagttgcaaatgggtttttcgcataaaggaacacgctgatggctccgtggataaatacaaagctcgtcttgttgctcggggattcactcaacagcacggcattgattactttgagacgtttagtccagtcgtaaaaatgtctactgtccggcttgtgctctctattgccatatctcgtcaatgggatatccgtcaacttgatattagcaatgcttttcttcatggtgttcttaatgaggatgtgtatatgcatcagcccccggggtttcaagatccttcaaaacctggttttgtctgcaagcttcataaagctatatatgggcttaaacagtctcctcgtgcatggtactctcgtcttagtgatcgtctctgccagcttggttttgttccttcggttgctgacacttctctcttcacctttcatcaaggcgcagttatcatgtatttattggtttatgttgatgatataattattgttagctcttcaccgtccatcactcagcttctcttgcagcagctttcagtgacttttccagtcaaggatcttggccctcttcatttttttctcggcattgaagtggcttccaattccgggggtatgacactcactcagcagaaatatgcccgtgatattcttcgtcgcgctgacatggagaattgcaagtcagtcccgactcccttatgcgttactgacaagctttctcgacatcatggcaacaagctaggtgacaaagacgcttttgtttaccgcagtatagttggggctcttcagtatcttactctcactcgacctgatttatcttttgcggtaaataaagtatgtcagtttctcgctcagccagccgatattcattgggaagctgtaaaacgcattcttcgatttgtaaaaggaacacttcatactggattatatctccgaaagactggttctactttattgagtatctttacagatgcggattgggctggttgtattgatgaccgacgttctactgggggttttgctattttctttggttccaatcttatatcctggagcgctcgcaagcaacctacagtatctcgttcatccactgaggctgaatataaagcactagctaatggcactgctgaagctatctggatacaatctttgctcaaagaacttcgtattatgcagccacgtcctcctgttctttggtgtgataatttgggagccacatacttaagcgccaatcctgtttttcatggtcgaataaagcatgttgaggtggattttcattttgttagggagaaagtggctcttggagcattgcaggtccggcttatatcatctggagatcagatcgctgatatatttactaagcctgcaaccaaacagatgttggagcgcagtcgtttcaatcttaaccttgtgtccaccggttaagattgcaggggga

>Copia16-ZM_LTR

tgttagcattctgttgtatagcgtagccaaggttgttagcatctatgctagattaactcctagattagctcctagacggttttagttttgtatctatcctgtgcacatgctatctatataaagaaacaaagcaagcaccgaggtgctgtgccaccaattatattcttcctattgcattgttca

>Copia17-ZM_IN

attggtatcagagcctaatcctcgttctaacgcttaaccgcgtgaggaaaagatcatgtcggggggactaagaaacgaaagtgcttctaagcttgaaaaagttgaggttgcctcgacttcatcttttggtgcagatgttgatcctagggcaatagatcttgccatgagaatcgccgaaaggatgttcctcaaaatgaaggaagatgaggcgaagaacaaaattgaagaagaaaatgatcgatggagaccaaacgacgaatccacctcttcacaaggttcgtctttcaaatccacttctcatatgtgctttattgctaatgggagtgacagtgaaagcgaaagtgaggatgaggaggagcaagaaagtgatagtgaagatgaggatgatcttcaacaattcttcgctcagctaagcaagaaacatcggatgagcttgctcaaactcatgaaaagagcggaagaacaaaaagaaatgcttcataagcaagaagacttcctcatcagaaaaatcgaagacttagagaagttgaccaaagagcatgagaagctaaagtgctctcatgatgatttggtccaaaggtatgaagacatttcaattgagcaaattaaagttgttaatcattcatcatatattgctcaattagaaaataaaaatgctatgttcaagaacacgatagaaaggctaaatattgaaaatctagctttgcaagaaaaacatgatatgcttgtgtgctctcataataaatttatggattcacatatcatgttagaaatggctcatgaggttgtgttaactaatttgaaatcataccaacctcacatatgcacatgtactcaaatagaaactatattatcatgtgctaacaaatgttgctctcaagaaagccaatcttccattgagctagaaatttcaggaattagtaatatttcctatcgacacaagaaaataaagagctcaaggaagaaaatgagaggctaaaaaggagcttgattcaattgaagggaaagtgtcatgctcaaccttctcaagataaccgtgataatatggtgaaaaagcttgagaaggggacaacagtagcatgcacaaaaccccttcaaaagaatatcaagctttccaagaaggacatgagcaaacatcaagagaagaaagccaaagctcatgacaagtgcctcaaccatgcctccacatgctccacacaaagtaacaaacaagtcactcttccaaataagagaagatgcgctagaaagtgttatcaatgtcatgagaagggacatgagattaagtcatgtccctacataaaggatagtggcttaactttagaaagaaagaggctcactaaccatgtagcaaacaagaagcaagggaagaaggagtcttgcaaaattaaaaatcacatttgctacacttgccgaagaaagggacacctatgcaaggattgtcccatgagtaagtatcctaagcctaccatgtcaattcattcatattcgcttaggagacccaaaaatgacacttgtgctagaaaggtaattagttcacctaaaactagcacaaaggccatttgggtgcctaagtacttattagctaaccttggtggacccatccaaaaatgggtaccaaaatgtacttaataagttttgcaggtacccagagatgatatgaagctttgggggtgcttgagcggtttaactcaattcttatctcaagctatcaatcttacattgtctatcctttaagattgacccaaagatgaattgagttgttatatcactaacttcatattcatctctagcaagaacttgtgttgtagggaataaggattaacctttgtgggaatcaagcaaaaggcctacaaccaagtgatatccaaaggatggtaacaattaattcttaagtgcacattgcttttaattggtatattcctttgtgtcttttgtagccacataggaaaaatgaagcacttgagaatgaacttaaaagattttaccttgctttggaaaggatctaattatatggtagattgcaagttcatatttttatattgtgacaatctacatgctttaaattgattgtatgtatcttgtggcatatttcaaattaattatcatattattgccatgacctagacataaagagtattatcccatgttcttaaatgaatagagtgctaagtaagaaattcaaattcttatagcacttaccaaatggggaattctctatatgactagagttgtgagactaatgtttctatctaagtgtttatgtagtctcacaacatgagaatgtgtttcccaaatggagtgtgccattcaacattcaaagaagatcgaaccaatactatgtgatgtattcattcttgtttaaattggttttgagtcttctacattaatcactcaccatgctatgaattaaacttgccatgtaaacttaattaaataatcatgctactttcatcttttttacaattgatgttatgcatgatgcttgtaagggtaatttagttcatatcatgaggtttccttgttttagtaaccttcttgtcattcatatactagaggttgctaaataggaaactattgcttgtgttactaatacaatctcttttaagatatttcatggaaattcataagtagagattgtgctctttcaattggtaaaatcacaagctttaaaggttaatcttcacctaaaagaaagattaggaatgctcaaggcaaaggtatggaactaattgcttcaattggtagttgatcaatagtagttcctttcaagtggcattctttcaattggtaataatctattctatggaaagaaatgtcaatatgaaggttaaattccttttggcttaaatttgtaaattagtgcatattgtgaattcactgctccatgtgcattaacttaaaaggaatttaatttatgcctttacgcctcataaatgatgatttgtttgccattcatatatagatatcatcattcattaaatacttccttgtactactaatacctcttttcaaagtgttttatcgactagttttaaaaggaaaagagataacaagctaaagaaggaagtctccacaaatgatgaaaagaagaatactaaggtgacaccaccacatatagtaagatctttcaaattggtataacaaatggtacattctatatggtggtaagtattcttaggcataagttaattcattgcaaatttgtcatgccttgaccattatatgtaatgtactcctcatgagactcttaactgaattgaaagtgcatgtggcaagtcattcaaatacttgatgcacatatctagtgggagaacattatatatatcttgtgtcatagagaataagtttcacttgagtaagctatactaagacaatccaaaatggtaaatttgtatctcattcatatggattgtaatctttgttttctaaatagctactcttgatgcagtttaaaattcccttatcgttaaatctaaaagtgcataagaatcctttttgttgatattcatcacatacatatgcactaacatggatatgatttttaaactgtaaaaggtacaattagtgatccatactctctaaattttggaaacccatattttgatatcttagaaatctacttcaatcggtatccatatgcttcacattgaattggatcactaagttgtaaattccatgcataacttgtctttatgatatgaatgcttgtgcgactaaccttgataagctaggtgcacccaaggtcaagctaggttcatcatcaagaaggcaacacaatgatgtatcaagaaaaggagaaaaggctcctattcttaactctagtttttatctatgtgattaaatattgacttgaaaccatgtaagatggttgtcaagtatatgtgggtgcctacacaaagagaaaggccacaataaggattgtgtgggtactcaaggctcttactactaatctcaaaggacccaattcaaattgggtaccaagatatgaagcttaaacttgttttgcaggatcactccttcaatggatcaagttgggtgctcgataatgaatgtataaatcatatttggagatagtagcaagggtggtaacaactgaatctcaagtgcatattattttcaaatcttatctcttttgtgacttgtgtagccactaaggaaaaagaagcacttgaggatatacatcagttgttgattatgaaataaaggtccaattggaagatgaatgaatattatcatatggtcaacaatccaaaattatgtgatgtattctctatctagttaatttggatttgattcttctatattagacactcaccataatatggattaagtcagccctttagacttcattgaataaccatgcatatttaaatccatgtcattcaataatatattgtgcatgagggctcaagggaaatttagtccatattatgaggtttctctatttttagcaactcgcttgccttccacatataaaagggttgctaaataagaaactagtgcttgtgctactaatgccatctcttgtgttgagtttatccatagaaaatctatgttaagagatggtgcttgttattaaattggataaatcacaagcttaaaggatactattcaactaaagtaagatgaagttgataagaggccaaggtatgaaaacttcctctccttcagttgttttagtattctatccttagtgggatgtaccatagtataggttaaattccttgcaatataaaatgttcaatagtgcatataactttgacatcaactatatgtgtgcactaatttaaaggaatttagtctatccttttgggtttcaagaatgatgattcatttgccatccacatataaggatcatcatttgtgaaaccctcccttgtgtttttaatgctctcttttctaagtgtttcaacaaggtccttccaagtgctttcaagatggattcaaaatctacggatataagagtcttggttatttcaatcattgagtttcaatgggtctcatatcaagtatgattgaaccaagcaaagatgaatgaagttcaagatcacttggttggatgaaatcataaagagaaaagaaatcacagtgattcaagaagggagttacactttttgtcaaccaaataatgaagatgtagtgacatatttatatgatatccttcattatgaggtttgaggttcatattagcatgctttacccttcaggatttcaattgatatactttcagttcttaaactttcaattggtttaattttcatcatctatgtaaagcatgttatgttaaaccaagatatagtgcaaacatctcctttaacctcgtattgttgatgtgcataagtgtactttgtgtactcttgcatgcacaaattgagggagagtttagcctatatcttgtgaggctaatgatttctttcaagttcatgttttgtagtctcacaccttggagaacatcaaatgaggatgagtggttccaaggaaatgatcaaatatttaccacatgtcaccccatggattagttctattggggaacgatatgtgttctctagcataaattcaattgtttcaaatatattatgccttgaccaagatatatgatgaactcctctaagaatcttaattgaatcaagtgcatgttacaagtaattcgagtacttgatgcatacatttagggggagctcattctatatcttgtgtccttaaagactaacattttcttttagtgaatttgtgtagttctttgaagagaatgagtttctcttgatcgtttaaagaggataagtttctctttgaaatgtcaagcctatcaaaagctaagatagaaattcatgattataatgaagaccatatggcatagaacaaaggtgtgtcactccatgaactattgtattacatttgtgtatctaggctcttacatgctagtgtrtgcatgtatatgcaatatcttaagtcacaycataaggttgcacttgtggtgatgattawagaatctctagatatttgattaatacctcttgtggtgatgtcataagtttgtcttaagtcatcttagtgaggtatgagtcaaacatgctaacttctcaagaatcttggcttaaaatattgcatatcatgtctattagtataccttttgattatgagtaattccttcaattggtgcaatttcatattttcatactttatttgtaccaaggttcaaattgtagaacttaatcccctggcctcacaagtccaagtgcataagctaaaacaagtattcatcacttgtatgcacacatttagggggagaatggtctataatttgaatctttgagactaacttcattttcaagtctattatgtgtgtagtctcaaattagaaaggaatcttcgggcaaagacaatcgcttccactgcaaattttgatgggtatcaaagattctttgctccaataaatgtatcttctatacatttcataagagaatgagtttctcttgtatatgctactccaacgtcatctaaaattggtaaatatgtatcacatccttttggattgcaaatgtctgaagtagcatagcttataaattctcctgtctagaacttaattgattaaatagtgcacatgtttcttatgttgcatgattatgttcaattgatactccttttatgccaatggtactttaagttgcaaataagtactctcaacaggtatcaattgaattcatatatatgtgtgcactaaaacttgaggagaacttagtgtaatatgcaattagtgatctgattatctatcaaattgtatcaattggtggttttcaattgatatttttcgtacatgatcactagttgtataatccatacttgtgcaattttcatgctgcctcttgttatgataagaaaatgctaggtgacatctagactccaggtatcaagatttatctttcaattagtatgacaatattcatttgatatcttggacctatgtcacaattatgccaacaagagattgcaaaatgaactctaaatacaacacaagtgtggaacacccctttgaaaaggtaaaacgcaaaggtacatcacttcatgacacttctccattacctttgtgccatataaggacccttttcatgatagtgtgttcaaatcttgattaatcataatttctaccctttatattctttatatctttttttggagatttatgacaaagggggagaaattgtgcattaaagcttacctttagtcttatgtaactaagtgtaagaagacttttgaaaagggggagaaataattaacaaaagggggttgaatcataagtaaaacataagatgaaggaaattgatgagtgcataatatgtcatgagcatcacgtttattttatgacacaaagcaccctatgaatagtatgatataagttgtcttcactctttgacccaaatatgtggttttggcatttgagtacaaaattggtattttctgaaatgcacatatttagggggaggaaactatatcataggattcaaaatcttatttatcaaatcttatgtaagctttaaatgtgttgtcatcaatcaccaaaaagggggagat

>Copia17-ZM_LTR

tgaaagtgcattcatcccttttgtgagttttggtgatttggataacaacacatttaaaggtctaacaagtttgctaagtgttgaacaggaaattcagtatgatgaacatacttgaatagtgtataatgatcagtgaacaaggttcaacacaaggtcaaataaccagtgagacaatgcaaatggatataatatggtctctatattggtttgaatatatggacaagtcctgagaaatcactatgcataaatatgatcataatagaggttgaagtgattaagaggattggtcaagccaaagtgaataagatatgaggaatcgtgaattggcttgaccatattactattagtccatatatgaatatatgagaatcaaactagagcttgattgatcttagcagttatatctagatgacattcaagcaaggttcacaatattgaagaaatgattctctcaatggatgctcaataggatgtgactcaagaatggcttgatagggtgaagatagcaaggaaagggcttcgaggaactaagcgaaggtgaaggccaagcgacggcttgtagaccgaggtaccatggctaaggtgaagaagagagtacttgcactaagtcgatgaactaatcagctatgaagagttacaacatgttgatgcatcagtaaggtgacttgaagccatgatttgaactcatatatggtgaaatggcacaagtcacaggctcgatttgtgtttgcttcaaaaggtgagacaaagatgtttgtgatccttatgaagcaacaccatggagaaatcacacttgagacaccaatgactcaaggagtttacttaattatattttatttaacttgagtataggaatcgtcgtactatcaagggggatccataaagaaggttggtgttggtactaaagctcaaaatccttgatctaaaacttccattttacccttgttaatcacttagtgaaagtatgtagtacaacctttttaagtctatcttggccaaaattgctttttggaaaaacaggttcaaccggttttaaaacaggttcaaccggtttttgcactgttcaccctttttcaaaatactggttcagccggacactcaaccggacactcaaccggtttttgtctggtggaaacaggttcaaccggttttaaaacaggttcaaccggtttttgcactgttcacttttttctggccagtctccggctgtgtcagccaaaaagctgtgcgcagctttttgaaaaaccggttcaaccggttttgggaccggttcaaccggtttttgggcagaaaagtcaaaaacggctagcagcttttggagccccacctatatatactcactcccacactctctcctccacagaagagcacgcacaaaactccattcctaacctgagaaacacctcccactctctctcacacacctcttgcctctcccatttcaaatctttggagagaaatctttgagtgagtttgaagagctgcggtttttgtgcttcatctccaaatctctcttgctcttcttgattcgagctttggtactacatcgagttctttgtggattcattactcttggagcttctagctcctagacgactaggtgtctcttgtgagtctccaaatcttgtggaagaccacaagaaagtttgtattacccgctcgtttgagcaaagattattgtgtgggcttgacctttgtggtcggcaaagggaggattagggttgaaagagacccggctctttgtgggcgcctcaacgaggaagtagggcaccttggtggtgtgaccgaacctcgggataaatcttgtgtctcttgtgttcttgctcattgtgtttgtttgtgttcttcgttctctcaccattgcgtggaagatttgtttatatctttttggtgtgtggattttgagaagtgcccttctcagatctactactttgaaccctgtggatcatttagaacatctcatttccaaagttaactgggtgaatttcgagatcaattcagttttacccagtttgcttctagtttttgttgaaaaagttttaacttgcctattcaccccccctctaggcaactttca

>Copia18-ZM_IN

cgttatcagcacgctctacctcgacgttgctgcgagatcagatctgcatcaactcttcgtcaagccggcaggtccttggcctagccgaactgtcctacgcgacaggcttcgattctccctctacgcaatcgagcggtatgttttcttgatccgctggtgtgatctgtgcgccacgaacttgttgtctccgtcgtggtgctgcgtccgtgccggaagcgctcgcatcggagcctcgccggagctggtcyactccaatgtcatgcctctgccagtctgcctacggtgagactacgccagcgctcgagcatgtgccacgctggaagcgtccacgccgcaccggagctgtgccgacgccagaagccacgcctgtgcctacagtggggccgcgtcgcgcaagcagcaagggtaatacgagacccagcaccgcgacatgaatcatgatggtcggctgtggcggtggtgcgagagcccgctagaacaggaggatccattcccagcctaattattctcgaagatctggacggtttcccggcaggcccattcgtccgtaacgtccgcatttcaaacttcaatgcgggattgactaccgagccggccgttgaggcaacgaaacagaaaaaccgtgtgacgttgcgcatcgtgcatgcatggacggtcaacagaaaaaagggagcggtcgtgggtcttgctcatgcacaggaagcaacgactcctctttgacctttcttggttttattttcgaaatggtcgttaatctcaatttaattatttatataaataaaattcttggtgaatttatataaatttgtatgcatgctctatattatttattctatttattttctattgcaaataacaaatagaataaagcatgttttatgttgcaatttctactttaattaattatttcctgcagtaaataattagtagaaaaatacatgcttaaatatggaaaaatgcyttttacacctgatgtgcttatgttcatgcattaattctggtatttatttttctgcttttatttattgtagcagaaaattattaaaatgcatgtgaaggctatattgtgcatactcaaatcgctctaaatatttatgttgcgttgagcttttaatttggactcacgtcgagcgattaaattgtatagtgggtactcagacacgccgatcaggctacattattaatcggctgagttatggcattggctgagcattggctgcgccctggcaacacgacgcagtatttgtgtcgtacgaacttggctgcgtcatgtgattgctatgtcgcgctctcgcttaatcggatcgctcccgttgagtcggaccgccatcgccgagtcacgtatttttcgcatgccctgtaggcactgtctgtctgtcgtgatcatcgagccacaattgcgcctgttgcgcacgttgcgtggacttatcgtgtaccccaattaccgagccataattgtgcctgttgcgcttattacgaaggcttgttgtgttgtcgtagccctgatggccgcacacgcgttgacgccagatacgctagagcttgtactcgcgtggattgtggtgggttgttcaagcccctgaagtcacattatacgcaaccttcgtaaacaattttgtttggctctatattttatttgattgtgttgtgtggcatcatatatttatatatatcgccaagtggccataacaactatatcacttgaataatctgggaaattaaattgaaatacaatgaaacacagataagttaattctctgctctctcctcatgcataaagttttacccgaagtaacccgaagatatataatattaatgcatgaaagcctttttggcatagaagacatcacaaattgggattctattagtaagcaaaaagaccttaccaaatgatttaagactagaaatctcagtcatacattgctcaaccagatgttagcacattgctctctttgtcgctgaatgacatgaagcaaactatgagataaaaggacatgtaaccgctagcaacacgaagttgcgagtataccgagaggttattctgagaaatttttcactatgttggtgtgaaaaccatagtgtgttcccgtcgaaccactttactcgttctatctaggacctctatgcttgtgtcgcatacatctctcagaattgtaaatacttatagtcactgaattgactcgggatagtcctttgacaagtttggtatacactgtaagctaaatggtaaaggtcggtcctgaatggacacatacctggggtttatgtagccgaactacactgaattccaccctttggcatgcttaccattatctacctccgatctaccagaagtgagaagagtagacaaataactatgaaatcccacatcaccacagagcttgaagctaaattagacattaacatgtccttggtcctcggagccagaagagtcatatgtcttgaccactagactactagtgatgataatttgtgatagaaatatgaagatcttgtagagacctgttgtatcctctctactcctgatgatttcttcgtatgagagcagtactatctgttgttgaagatggatcctaggcggataccagtattgtttcgttcttgccaagcagttatcatcatttgcttcatcaaaagctagttatatggtgatttttcccagataaaatgaattcttggccttgtctgttctattccaaagcttctctttttgctgaacaatgaagagatcgaaataatgctatatagaacaatttggtatataatatgaggagaaatgtttgccctgctggcaacaccacaaataattaactattgttataagttctctctcaaaattattatacctaaaaatgttgcataaatcgatacccaatttcagaacggtatgagtaattgggtaaaccatgggcaatgataaaaacgagctggactatacatctctgcatataaaaatctttgcttggcagcattggcctgatgccgtgcactttactaccattatatacacacacattttcctatgtgcttcaaagcacaatgcaaccagtgaatgtctttgtgagcgttagaccctgatggtcattttacttgttgatctgaagtcaactaatggctccaaatgacgaatggtatgcatgagcccctgaaggacccttatggataaaatagtgttgtgcatatcagtctaaatcttaatgattgactgtgcatttggtaataataacaataagtttgcagaattgtaaccatgaagcaacttgttgttgcgtgtctcgctggatgttgagactaacccttcatgttgaaggacaaatatgtagtattcatgccactacattaatctaagtccaaagctcactgcataaaacccctatctgtaatgtgcgtaagatttcccaaataaatcaccacaatagcatacattggccacaactggatgattgtggttggggattttatctgtatgcttttagaacatctcggcattagggggaggaatgcccatatagtgtaatgcctcaatattctattaaacgcacaaaagccaaactgaacctgtcgttcggtgtgtactcctgtgtatatggagtttgccagaaatcgttgaggagtaaccatattggtctgaatgcttctacctgatgtagatgtggatatacccgggattaatatcatatccacatttgacttattggcatttgatctattctcggggacgcctgcgaatatgatccattggccttagtcaaagcatatgttctgattgcagattcacgtggtctgtgataactctcctccgattaattcaccctgatggtgatttgcctcacgaagaggttcatcttgatgatgaaattcctagcaatgcgcgcaatatcattgtgctgggaatacggaacaatgaatctttcgttttgggaaatgacgaagatcattattaaatgtgggagacaaatatgtcgacacctatcttgcctctctagattgcaaatggatccaaaacaaagtcctaggcatgagtgctttaagctctcttatcgggtcattgataaatgcaatcgaggctgaaccaataatcgcaaaatgaaagtcgcgagcttgaagttcggacatttatgggcactattgaaataatgtgtggtgaatgcgatggttcaagtggtcttgtgagagacccatcccacatatgtgttgaataaacactgttccgctatgtaatatatctggcaaatggcatgaattaaaatatgcagttaataggattctgaagatccgtcatgagatcctaggaggactcatatctttgaattataaatatgcaacatataaatctcgtaccgaataatacattcctgatgaatgatcactctcctatgtagagagaatatctcctagttgagattatagttcctagatggaacctttccagaagaaataaagtctgtgttaaacaccaaagtttgataatccctataaagggataaagacccatacagacccgaaaaggaataagatgaggtaccaaaggacttgaagttcaccgaataagcacatgtgcattccatgagttttactcatggtaatttccactagatgtggaattacggtatcctctaaagccctgatggcagaaacctataaggtttaggtgttactggcaaaatatccccattgtgccagaatgacagactataacgatgtatctgatcgatgaaaaatccctgatggattacgatctttgatggacttttgttacaccatcatagatgttgcaatggatgaacgcctggagcgatgtgtcatatttagaatatgtactattgcaactatattatcccctaagtcctattgaaggacatgttatttgctttgcacaactatgtataaattgtggtgtaagatagaaaatgatagcaccccaacctcatccattctcgttattccagatgaacaatgagacatatatcttgaagaatatgcttgagttatgagggataacgactttgacagatgtcatcgtcagggggagcgaatgcttatattgatcacaatcgtgagacaataaatgtcatattctatgccctgaaggcgtgagcttgatgatcaatatgtgaacctttatggaactttctatcaaatatatagatccagtcgatcgaacaccatcagtcaaagtggcttatttatattgatacgtgcacttacctcgtatatcctagaagtgagtagaggtaaagttcctgaaatagtccttgcaaggatatgcaatccgtttgctaaatctttatgtgcatatacttccagaagaagatgttttgccttattgatacggttttgcaaggatcagggggagcacatttctaaagttagcccttgtagaagattcgatagaatctagatcccagaggatcgaaatctgtcccgatgacagttgttgtactctttttcccttggtgagttttcttgaagtttctcacatgaggtttttaacgaggcaacaaagtgcaaatgcaatttgtatcaccatgcactctttctccacattttcccactgggtttttcggagttttaacgaggcatgtgttggtcgcggtattcgcccaagggggag

>Copia18-ZM_LTR

tgttaagtaaccctagttagggttatgtgggcaaatacctgctttgcccctgaggggtctcacatctctatataaggaacctgtacaccccttcataatacagagaacagaaagaggccagaggcccaaccctatatcctgtgtctcgtgtgtttcctctgtcgtgcttatgggaagggagacgggttctctacatcttcttgcgcctctactgctgacgggagggaagggagcggatctggtgatccgtggtaacgtagttctcaaca

>Copia19-ZM_IN

tggtatcagactaggtttagggttttcctcccacctccagcagccgccggctgctcccaaccccacagccgccagccccaggtccagccgccgccccgggaggctcatccttccctcccggggcggctcccttcccagccgcgtccgccacgacaggccacatcccagctgccgctgctcgtgctccccaccatcggccgctcgggctcaccttccctgcccacggcgcgggcgcgaccgcggccctcctcccccgccccgacctcctccggccccggytccccgtcgcgctgggccgccgcgactccctccgctccgacgcgcgtgacgtcgagggcaaccttggagcccgcggcggccgcctctgaagccggatgtagcgcgcgcgtcgcacatgggggagctcgcgacgtcttcctcgcccccgccgcccgccgtgctacaggtggtcttcgccgacrgcgggcagagcctggacctgggcacrgtcgccgtgcagccgtcgctcagcggcgcgaagcggctgcaggccatggtggccgaacgcgtgggcgtcgcgtcgtaccagaccccggcgtccctggcatggccccgccgcgcgcgccgtgttcccctcgacaacgccaccgacctcgccgccgccttactggccgatggtgcggccagtcttccccctctaggagcgggygcgggtctccgcaccgccgacctgctcgccgtgggcttcctccccctcttcgccggtcacacgctcgcgacggcgtcgctccccaggtcggcgcgacccccatggtcgcgcgccagccaccagcagcggcgcttctccccgctcaccggcgagtcccgcctctgctccccctgcagccaggcctccggctccccctccctcgcggccctcctccgcagccgcggccccctcccttccycggcggcycattccccgccggcggctccctccccacctgcgtccccctcgycgtcaatcggcatccctgcccgctcgtgcgctatgcagccccttgtcatcgcgctggccacctcgtccctgaccgcgctgacgccccagcagcgcctcacccttcgagcttctcccgtcgccggtgccgtcaacaccccttgccaggtccggcgcaggcccctgcccggctggacccgccccgccgcccacccccttcatcgccttcgttggatccgtcgctgccgcggccttcccggccggatccacgttgctgcgaccttcccggctcgatccgccgtccctttggctggatcgcgtcttcccggctggatccgtcgctgccgtggccttcccggccggatccgccgtttccccttctattctgccgcgggcgcggacaccatgaccggcgcagacgcgtgcgctgctgtcggcctccctggcggggctccttgacgcccggacgatcgaagaagcaggaaggtcggcctgccggtgcccttttgttgtgtcgccctgccgatcgttgacgctcgaacgtcgacccctcccgaagatcaggcttttgctgcgcgtcttccgaccgcgaccacctcgacttcggctacctcggcatctaggggctatcgtcttcttggagcacacaccggtctctactccagccgcaacattcgcaccatcacgacgctgcgactgcggggggatttcaacccgtcggctcctaccttcggcctctactccagtctcatcgtgtgtggtgcccccgttgcgactgcgggggga

>Copia19-ZM_LTR

tgttagactgtgtgtgtgtgagtgtgtgtgggccggcaccactgttgggctgccggcccattagggttagggtttcctgtgagtctctatatatatgtaccccatctctctatgcaatacatccaacagttcactcttctaca

>COPIA1_ZM_IN

tggtatcagagcctaaaccatctaccaaaaccctcatcatgtcgtccagtgcttcctccgccgcacttgttgcacatcccgtttctgagaagcttaccagggccaaccatgccttgtggaaaggcccaggtacgtgctgccatgcgcggcgcacgtctggagggatacctcaccggcgcaaccaagatgcctgatgaggagactgtcgacaacaagggcaagaagagccccaatcctgcttttgaagaatgggaggcgaaggatcaacagatccttagctatctcctctcatctataagcagagaggtgcagattcaagtcacctcagccaagactgcggcggaggcatggcactccatcgaggccatgttcgcatcacagacccgagcccgggctgtgaatcttcgccttgctctgtcaactacaaaaaaaggtagtatgaccgtcgccgagtactacaccaaaatgaagggctacggcgatgagatggctgcagcaggaagaccgctgcaagatgaagagatggtcgagtacatcctcaccggcctggaggaagaattcctaccgatggtgtctgcccttgtcacgagggtagatccaatctcacttgaagacctctactctcagttgctgaattttgagaccaagttggatctcatgagaggtggaggcgagcagcatcaaggatcagcaaatatggctggtcgcggtggccgtggaaatcaacgtggaggcagtggtggtcgtggccagcgtggtggccgcgggtcatctcgtggtggccgtgctagttggtccggagggcgccaaagcaaccagggaggctacatcaggcgttccaacaactccagcgacgagaggccggtctgccaggtctgcttcaagaagggccacaccgcagccaggtgctggcacaggtttgaagaagacttcgtcccagatgagaagcttgctggagccgccaccaactcctaccacgtcgacaccaattggtacacggacaccggcgccaccgaccacataacaggagagcttgagaagctctcaatcagggagaagtacgctggtggtgatcaaattcacactgccagcggttcaggtatgacaatcagtcacattggtcaatctactgttaatacccctaatagaaatcttcatctaaatgatgttctttatgttccacaagctaaaaaaaaccttgtctcagttcataaattaacttcagataactctgccttcatagaatttcatccaaatttttttgttatcaaggacaaagacaccaagaacattttgcttaaggggcgatgtcacaaaggactgtatcccatccccgctacatccacaataaagaacgctcttggtgctgtcaagccctccatgtcccgatggcacaatcgtttaggtcatccatcttctttcattgttcgtcaggttgttagcaagaataaacttccttgtttaggtccctcaacagatgaatccgtgtgtgatgcctgtcagaaagccaaagctcatcaattcccatatcctagatccactagcgtatctagctcccctctcgagctagttttttctgatgtatggggtcctgcacccgaatctgttggcagaaaaaaatattatgtgagctttattgatgattatagtaaatttacctgggtttatcttattaagtataagtctgaagtttttcagaagtttcatgaatttcaaagccttgttgaacgactttttgacaggaagattcttgctgtccaaactgattggggaggggaatatcagcgcttgaataccttttttaataagatcggcatttctcatctggtttcttgtcctcacgctcaccaacaaaatgggtcggctgagcgaaaacatcgacacatcgtagaagttgggctttctttgcttgcacatgcgtctatgccccttaaattttgggatgaggcatttctagctgccgcatatctgatcaatcgcactcccacaaaaattcttaatctagacacaccttttgagcgcttgtttcataaacaacctgattattcagttctccgcatttttggatgtgtatgttggcctaatcttcgcccatataactctcacaaacttcagtttcgttccaagcaatgtgtttttcttgggtatagtagcttacacaaagggtttaagtgtttagatgtctctactggcagggtatatgtttctcgagatgttacatttgatgaaaatttttttccctttgcttccttgcatagcaatgcgggtgcacgattgagatctgaaatccagcttctttcacctgatcttttaaatcctgccacatttaattctggggtagacagtttgattgatcatgcggctgatatgtctactgaccctaaccaaatttctggagacaattctgttcaagatcaggcagaacacacaccaaatatggatgtcctagcgccttcagtaccaaacactgctcctgaagctgatgcggtgcatattcctgggagtgcttcgcactcgggatctgctcctgcagcgccatcgacagctgcaccgtctccaacctgtcgtggcgactcgcgcgttgccacgcacccttcagctagccacgattatgtggcttccgtcgacatgtcccgcggcattgcgggacgggaagagagcttcttgacagctcccactaccgcgacatcaccagcgagttctgagccgtctggttctctgcctgcgaccgctgctgatgcacaagtccctgaaccgattttgggaggtgtatcttctatggaccctgcatcaactcgcccaaggacaagacttcaacaaggggtcaggaaaccaaaggtttatacagatggcactattcgatatggttgttttacttcttctggtgaaccttatgatctaaatgaagctttaggagatgttaattggaaagatgctatggatatagaatactcagctcttatgaaaaataaaacttggcaccttgttcctccaaaaaaaggtaggaacgttataggatgcaaatgggtgtataaaattaaaaggaaagccgatggaagcttggataggtacaaggcacgactagtagctaaagggtataaacaacaatatgggatagattatgatgatactttcagtcctgttgttaaacatgctaccattcgtataattctttctattgctgtgtcacgagggtggagtctatgtcagcttgatgttcagaatgcattccttcatggagtattggaggaggaagtctacatgcagcaacctccaggatatgaggattccacaaaactcaactatgtgtgcaaacttgataaggcactctatggattgaaacaagctccacgtgcatggtattcacgattgagcaataagcttctttctcttgggtttcaagcatctaaggctgacacatcattattcttctacaataagggaagtgttactatttttgtgcttgtgtatgttgatgatataattgttgcaagctccactcacaaagcaacagaagcattactaagtgatctcaacaaagagtttgcactcaaggacttgggtgacctcaactacttcctaggaatagaggtgaataaggtacgagatggtattatcttgactcaagacaagtatgcttcagatttacttaaaaaggttggtatgtctgactgcaaaccgatcagtacacctctttccactagtgagaagctatctatacatgaagggtcacctttaggagaaaaagatataacccagtacaggagtatagtgggggctttacaatatcttactttaactagacctgatattgcattttcagttaataaagtttgtcaatttcttcatgctcctactacgttgcactgggcagcagtcaaaaggatacttagatacatcaagcaatgtacaaacttagggctgcatatccatagatcagattctactcttgttagtgccttttccgatgcagattgggctggcagtgtagatgatagaaaatctacaggtgggtttgctgttttcttaggatcaaatcttgtctcatggagtgcaagaaagcaacctacggtgtcaagatctagcactgaatctgaatataaagctttggccaatgctactgcagagttgatatgggtacaaattttactcactgaaattagtattaaaagtcctagagcagctaagttgtggtgtgacaatttgggagctaaatatttgtcagccaatcctatatttcatgcaagaacaaaacatattgaagtggattatcattttgtgagagatcgggtcgcaaagaaactacttgatatagaatatgtgcctactggagatcaagttgcagatgggtttactaaacctctttcagttcggcttctggagaatttcaaagtcaatctcaacctcactcggttatgattgagggagggtgttagaagataagattagattgtaaacaaatatctgtaacaaacaagagtaagcatctctgggaagataaagatagggatattctgttgtaactagaattcctgaagcttctctagtttgttacgatcctgtaaatcctgcgcatgttgtttatgcctatataaacatgtagggcggccctccaacgaggcagcacgctttaacctattgccctctgattttcacactacggtcccatgaaatcatactgtttctctttctcccccgtgtgtgtgcttcacgtaacacatgtttggttgcatcaattcccatccaaacatctgttgttgttggtgatggagtcgggacgggaaggagca

>COPIA1_ZM_LTR

tgttagaagataagattagattgtaaacaaatatatgtaacaaacaagagtaagcatctctgggaagataaagatagggatattctgttgtaactagaattcctgaagcttctctagtttgttacgatcctgtaaatcctgcgcatgttgtttatgcctatataaacatgtagggcggccctccaacgaggcagcacgctttaacctaatgccctctgattttcaca

>Copia20-ZM_IN

attggtatcagagccgtactcttcatctaagggactaaccgcccgaagagatggatcctaagggaaaggggatggtggtcaacgacaaggagaaggagtccctcttcaacgagccaagagacgacaagcccactgactcaggctcgagtcacaagaagagggacgggaagaagaagagacgcatcaagaagatcatctactacgacagcgatgcatcttcttcttcaccaagggacgacgacgacgacgactcctcgtccaagaaaaagacggttaatcaaaattattcttttgattattctcgcattccctataattcaaatgcgcatttactttcaattccccttggaaaacctccacactttgatggagaggactattcattttggagtcataaaatgcgtagtcatttattttctctccatcctagcatatgggaaattgtagaaaatgggatgcattttgatagtacggataatcctgtgattattaatgagcaaattcataaaaatgcccaagctactactgttttgctagcatctctttgcagggatgaatataataaagtgagtggcttggacaatgccaaacaaatctgggacaccctcaagatatcacatgagggaaacgatgccactatgatcacaaagatggaactagtggaaggcgagctggggaggttcgcgatgatcaggggagaggagccaactcagacctacaacaggctcaagaccctggtcaacaagatccgaagctatggaagcacaagatggacggatcatgatgtcgtccgactcatgctaaggtcatttactgtaattgacccccatcttgtcaatcttattcgtgaaaaccccaggtacaccaagatgacgcccgaggagatccttggaaaattcgtgagcgggcgcatgatggtgaaggaggcgcgatacgtggacgatgcgctaaatggtccactacccatctacgagccacagcccgttgctctcaaagcaacaagcagcagggaggcgctaccaagcaaggtggcacaagtggaggctgccgggctcaatgaggatgaaatggcgctcatcatcaagcgcttcaagaccgcgctcaaaggacgcaaggagtaccccaacaagaataaaacaaggggaaagcgctcctgcttcaaatgcggtaagactggtcatttcatagcacaatgccccgataatgataatgaccagggacaagaaaagcacgggaagagggagaagaagaagaactacaggaaggcgaagggcgaggcgcaccttggaaaggaatgggattctgactgctcttcatccgactccgacgatgaaggattggctgcctcggccttcgacaagtcttcgctcttccccaacgaacgccatacttgcctcatggctaaggagaagaaggtacgtattcgagattcccctaagtactcttcttctagtgatgaggaatcttctgatgatgaagtagattacactgacttgtttaaaggattagatagagctaaggtagacaaaattaatgaattaattgatgctctaaatgaaaaggatagattgctagaaaagcaagaggacattttgtatgaggaacatgacaagtttgttagtgttcaaaaatctcttgctttagaaattaaaagaaatgaaatgctatcctctgagttgtctgcttgccatgaatctgtctctagtttaaagaatttaaatgatgaattaaatgctaaactagaggaagttaataaaactagttcatgtgtagagcatgttgttatttgtaatagatgtaaggattttgatgttgatgcctgtgatgaacatcttgcttctattactaaattaaataatgaggtggcaagtcttaatgctcaacttaagacttgcaaagttgattttgataaattaaaatttgctagggatgcctacactgttggtagacacccctcaattaaggatggacttggttttcgaaaggaaaccaagaacttaacaagccaaaggactcccgttctcaacaaggagaaagggaaggcccctatggctagtagtcctcaaaggaaccatgcttttatttatgataggaaaattgctagccgtgctcattataataagagttatgttcatrctgcttataatgattcacatgctatgtttgcctctagctctacttttgttcatggtagaagtaggcctaggagaaatcatgttgtgtctcatgctcctaggagaatgtgcaatgaaccttctactatttatcatgcttgcaatacttcttttgtactttcatgtaagaatacaaaagtagttgctargaagttgggatccaaatgcaagggagacaagacttgtatttgggttccaaaagttattgtgactaaccttgtaggacccaacaagagttgggtacctaaaacccaagcttaaattgccttgcaggtttatgcatccgggggctcaagctggattatcgacagcggatgcacaaaccacatgacgggggagaagaagatgttcacctcctacgtcaagaacaaggattcccaggatacgatcatctttggagatgggaaccaaggcaaggtcaaaggcttgggaaagatagccatcactaacgagcactcaatatcgaatgtatttttagtagagtcgcttgggtacaaccttctgtctgtaagtcaattgtgtcacatgggttacaattgtctgtttacaaatgttgatgtatctgtctttagaaggagtgatggttcattagcttttaagggtgtattagacgrcaagctctatttagttgatttttcgaaagagaatgccgatctagatgcatgcttaatagctaagactaatatgggctggctttggcatcgccgtctagcacatgttgggatgaagaaccttcataagcttctaaagggagatcatgtgttaggactaaccgatgtctgttttgagaaagacagaccttgtgcagcatgtcaggcagggaaacaggtgggaagcactcatcacggcaagaatgtgatgacaacatcaagaccactggagcttcttcacatggatcttttcggacccgttgcctatctaagcatcgggggaagtaagtatggtcttgtaattgttgatgatttttcccgcttcacttgggtgttctttttacaggataagtcagaaacccaagggaccctaaagcgcttcttaaggagagctcaaaatgagtttgagcttaaagtgaaaaagataaggagcgacaatgggtccgagttcaagaatctacaagttgaagagtttcttgaggaggaaggcatcaagcacgagttctccgctccctacacaccacagcaaaacggtgtggtagagaggaagaacaggacgcttatcgacatggcgagaacgatgcttggagagttcaagacrcccgagcggttttggtcggaagctgtgaacacagcctgccacgccataaaccggctctatctgcatcgcctcctcaagaagacctcctacgaactccttactggtaacaaacccaatgtctcttactttcgtgtatttgggagcaaatgctacattctggtgaagaaaggtagacattctaaatttgctcccaaggctgtagaagggtttttactaggatatgactcaaatacaaaggcgtatagggtcttcaacaaatcatcaggattagttgaagtctctagcgacgttgtatttgatgagactaatggctctccaagagagcaagttgatcttgatgatatagatgaagatgaagttccgacggccgcaatgcgcacgatggcgataggtgatgtgcgaccacaggaactacaggaacaagatcaaccttcttcctcgacactggtgcatcccccaactcaagatgatggacaggtacctcaagaagaggagcgtgatcaagggggagcacaggaagaacaggttatggaggaagaagcaccacgggcccctccaactcaagtccgagcgacgatccaaagacatcaccccgtcgatcagattctgggtgacatcagcaagggagtaactactcgctcacgattagctaatttttgtgagcattactcgtttgtctcttctattgagcctttcagggtagaagaggccttgcaagatccggactgggtgttggccatgcaggaagagctcaacaacttcaaaaggaatgaagtctggagcctggtgccacgtccaaagcaaaacrttgtgggaaccaagtgggtgttccgcaacaagcaagacgagcacggagtggtgacaagaaacaaggctcgacttgtggcaaaaggttatgcccaagtcgcaggtttggattttgaggagacttttgctcctgtggctaggctagagtcaattcgaattttattagcctatgccgctcaccattcttttaggctgtttcaaatggacgtgaagagcgctttcctcaatgggccaatcaaggaggaggtatacgtggaacaaccccctggctttgaggatgacaggtatcccgaccaygtgtataagctctctaaggcgctctatggacttaagcaagccccaagagcatggtatgaatgccttagagatttcttaattgctaatgctttcaaggttgggaaagctgatcccactctttttaccaagacttgtgatggtgatctctttgtgtgccaaatttatgtcgatgacataatatttggttctactaaccaaaagtcttgtgaggagtttagcagggtgatgatgcaaaagttcgagatgtcgatgatgggcgagttgacctacttccttggattccaagtgaagcaactcaaggacggcactttcatctcccaaacgaagtacactcaagatcttctcaagaggtttgggatgaaggacgccaagcccgcaaagacaccgatgggaaccgacgggcatgtcgacctcaacaaaggaggtaagtccgttgatcaaaaggcataccggtccatgataggttcattactttatctttgtgctagtagaccggacattatgcttagtgtatgcatgtgtgctagatatcaatccgaccccaaggaatgtcaccttgtggccgttaagcgaattcttagatatttagttgctacgccttgcttcgggatctggtatccaaaggggtctacctttgacttaattggatacgcagactccgattatgctgggtgcaaggttgataggaagagtacatcagggacgtgccaatttctaggaaggtccctggtgtcctggagttccaagaaacagacatccgttgccctatccaccgctgaggccgagtatgttgccgcaggacagtgttgcgcgcaactactttggatgaggcaaaccctccgggactttggctacaatctgagcaaagtcccactcctatgtgacaatgagagtgcaatccgcttggcggataatcctgttgaacacagccgcactaagcacatagacatccggcatcacttcctgagggaccaccagcaaaagggagatatcgatatttatcatattagcaccgagaaccagctagccgatatcttcaccaagccgttagatgagaaaacrttttgcaggttgcgtagtgagctaaatgtcttagattcgcgtaacttggattgatctatagcatacatgtgttctatgcctttgatcatgttactttgagcatttacattattcgttgtatatttatggtgctcaagttgtacaagtcatccccggacctcacaagtccatatgcaaatggtgcacatatttagggggagatgtgctacaacttgaccctttgagactaacatgtttgtttaagtacacctgatgtagtctcaaaggtacattgaaagggaaactgaacttggacgacgcaaagacttccactgcactccggtattagtgtatttaattccaagttcatacttatgctctcattgcctttttgctcttaattgacatttttggtgaggcaatggggttaaagggccaagaatgatcccgttttggtgcttaatgccaaaggggagaaattaaggccaaagcaaaatggatcagccaaccacttgtgaattttgaaaataatagagtaagaatttgtgatttgctcaaaatactcttattgcaaaatttggtctcttgtgggggagaattttgattatgggaaaaagggggagtttttggcacttgattaattttactcttggaatatctctctatgtgcccaaacaagtgtgtttgacttagagataggaaaaagaatttgatttgcaaaaacaaaccaagtggtggcaaagagtgatccaaatatgccaaatcctaagtaaaaagaatttggtcttcatttgcattgatgttgcacttcttttgttgctttttgatgtgttggcataaatcaccaaaaagggggagat

>Copia20-ZM_LTR

tgaaagggaaatgtgcccttgggccatttctataagtgttttggtgattaaatgcccaacacatattgattaagttcttatgtgctaaatagagtgagaagtgcaaatcaaattacaaggtatgtttctagacttagtacattgtttttgaatactaatgtgtttgtctaagtgctagaaacagtgccaagagaagaagaaaagaaatggaaaagacttggctgtgtgcagccaaactccagctcggcctggcacaccggactgtccggtggtgcaccggacagtgtccggtgccccaggctggccgacggtgaaccagctgctctcgggaatcgacggagttgtacggctataaatcaccggactgtccggtggtgcaccggactgtccggtgagtcatccgcgacgaactcgtcgctctcgggaaatgaaaaaggcgacgtggctaaaattcaccggactgtccggtggtgcaccggactgtccggtgagccaacggcgccagcggccaacggtcggccgcgcgatcagcgcgcgacacgtggcccgctccaacggtcggctgggcgcaccggactgtccggtgtgcaccggacagtgtccggtgcgccaacgggccccgaggagcaacggtcggctgcgcccgatatggaaggagatcgcgcaccggacaatgaacagtggctgtccggtggtgcaccggactgtccggtgcaccactcgacagaaggcaagaattgccttccaagttgatctccaacggctcctagctgccttggggctataaaagggacccctaggcgcatggaggagaacaccaagctttcaagaaacattctaagacacccagactccgcctccacgcatttgattcattgtgttagtgatttgagctccatttgagttgtgaactccctgtgttgtgttttgagctcaagtcttcacttgtgtgcgtggttgtgctgcggatttgagtcttgtgtgtgttgctctccctcccttacttctgtgcttcttttgtgatcatctattgtaagggcgagaggctccaaattgtggagattcctcgcaaacgggagaaagactataaggaagaaagccgtggtattcaagttgatcattggatcgcttgaaaggggttgagtgcaaccctcgtccattgggacgccacaacgtggaagtaggcaagtgttacttggccgaaccacgggataaaaatcgcgtgtctcgtgtgttgcttcttgtgattgttttgttcgcacgagcgtgttccatagccacttgatttcactaacactttaataatcaagtttgtggctatttagtgtttgattttacaggatcacctattcaccccccctctaggtgctctca

>Copia21-ZM_IN

tggtatctagagcctattcctcattaagctcatccatggcgtcaccatccaatcctcctctttccctcccttcctccctgaatatcaccatcaccgagaagctctccaggacaaatcacgtcctctggcaagcccaagtgctgccggcgatccgtgctgcccaatttgagggttatcttgatggatccattgctgcgcctcctccggccatcaatgaaaaagacggcgataaggtcatctccaagcccaacccggagtatgctcgctgggttgcgcaggaccaagccgttctcagctatctgttctcatcgctcacaagggagatccttacaactgttgcttcgctccgcacctcggcgcaagtttggtctaccttggagcagatgttcacgtgccaaactcgggcgcgttccgttagcacgcgcattgcgctgcatactctgaagaaagggaattcttcagttgctgactattactccaaagtccgtagcctcgccgatgagcttggtgcctcgggcgtggttatctctgatgatgaactggtgtcctacatcttgtcgggccttgaagaaaattacaatccggtggtctcggcggttctagcccgtgaagaaccagtaacgcctggggccctgtatgctcagcttttgagccatgaatctcggctagagctgcaatctggtgcaagcaacatccagttctcagccaatatggcgtcctatggaggtcgcaataggggccgatctggctcctctcgtggcggtgggcagtcccgtggtcgtggccgattccagggtgcctactctagcaacagcaccggccgccacaataggcaagacactggcaatcgctccaagaaacccacgtgccaactatgcttcaaagttggacacgttgttaccaattgttggtacagatttgacgaaaatttcgtccctgatcaacgcctcgccgcagcagccgctccatccactactgatccatcctggtacacagacacgggcgcaactgatcacatcacaggcgaattggacaaacttgctgtttatgatcgctataatggcactgatcagatacacactgcaagcggcgcaggtatgaacattcgtcacattggttattcgtctattcatacccctggtcgtaatttacatctccacaaaattcttcacgttccccagacatcaaaaaatttagtctctgttcatcggctcactacagacaataatgtttttattgaatttcaccccaaatttttcttgattaaggatcaagcaacgaagcaggtgcttcttcgaggaccatgtgaacgtggactctaccccttgccgtccccttctcagtcatccgataataaggcggcgtttcatgtttccacagcgatgccgtccctttcaaggtggcaccacaggctcggtcatccctcctatgctacggtgtcgcgcgttgttcgtgaacacgcattgccatgttcttctgagtccttgtcccagtccgtgtgtggagcttgccaacaggcaaagtctcaccagcttccttatagtgtgtccactagcaagtctagctttcctcttcaattagttttctctgatgtatggggaccggccattgattcttttggtcgttataactattatgtcagttttattgatgattatagtaaattcacctggatatatctcatgcgccataagtcggatgttttttgttattttaaagagtttcaagctcttgttgaacgccttcttggacgaaaaatcattgctatacaaacggactggggaggcgagtatgctcgcctcaacacctttcttcgacaggttggcatctcccatcttgtctcttgtccccatgctcaccaacaaaatggtgctgcagagcggaaacaccgccacatagttgaaatcggcctatctcttcttgccacggcaaatatgccgctcaaatattgggatcaagctttcttggctgccacatatcttattaatcgaactcctagttccgctatccagtttcaaactcctctctttcgcctccttgatcgcacaccagactaccaaaatcttcgaatatttggttgtgcatgttggccttgtctacgaccatacaacacaaaaaaacttcaatttcgatcccttcgatgtgtctttttaggatacagcaacatgcataaagggtttaagtgtcttgaccctgccactggtagaatatatatttcccgtgatgtcatctttgatgaatcagttttcccttttgcatcgacgtcttgtactggcgctcgttttagtagtgaagtccttcttcttcctacaccagggatgaatacgaataattctgttgacgatagttcacctgcttctatttttccaatgcttcgtccagtttctggctataatgacatgcaggacacatcaggctccctgatcgggaatcgtctagatcaaacccctggtgaaaataataggagggctgcttccccgggtccagctggagctgctctggattccacgacagacgactacccatcatcagttgttccatcgctcactccggtctcgatcgagggggacagtccggacctcatctcgccagatgcacttgacaccgcgagtgttcgggtcagctccgcctcgcatgaagcgccatcaactgcgacagacaacaacgatgagtctagtttgggatcggtgcccatatcatcctctactgcaccggatcctgaatcagcagctgaagaacttgttcctgccttatctcctgcgtctcctgttcaccgtaccaggcttcagcaaggtattcgtaaaaccaagcagtatactgatggcaccatacggtatggatttcttattactacaagtgagccatcaaatttaactgatgctcttaatgatgcgaactggaagctagcaatggatgttgaatatgcagctctcgttcgaaacaagacctggcatcttgtgcccccaaaacaaggcaggaatataattgattgtaaatggatatacaaaattaaacagaaggcagatggatcagtggatagatacaaagcacggttagtggcaaaagggttcaagcaacgttatgggatcgattatgaagacacctttagcccggtggtcaaacctactacaattagacttgttctctcaattgctatgactaaaggatggtgcctgcgacagctggatgtgcagaacgcgtttcttcatggtgttcttgaggaagaagtgtatatgagacaacctcctgggtatgaagatatcactaagccacatcatatatgcaaacttaataaggccctttatggtctaaagcaagcgccacgtgcttggtatgctagacttagtgataaactcaagatacttggttttcgatcatcaagagctgatacctctttatttttctataagaaaggcatgatcaccatttttcttcttgtatatgtggatgatatagtggttgtaagctcctcgtcaaaggcagtccaagcgttacttgaggatctcaaaactgattttgctctaaaggacttaggggaattacattattttttgggaattgaggtcaaaagaatcagagatggaattttgttatctcaagagaaatatgccactgatcttctaaagaaagctggtatggccacctgtaaacctacggttactcccttgtcaacaagtgaaaaaactgtcagctcaggtcggggatgctttaggctcagaagatgctacaaaatataggagtgttgttggaggcttacaatatttaacacttacaagaccggatatttcattcgccatcaataaggtgtgccaatatttacatgccccaactactcaacattggactgcagttaagaggatattacgttatgtgaagggaactgccgacataggattaaaaattaagcaatcatcgtcctggttaatcagtgccttctctgatgcagattgggcaggctgtttagatgatcgaaaatccactgggggttttgctgtattcttgggatccaatcttatatcctggagtgcaagaaaacaagctaccgtgtcaagatccagtactgaagcagaatataaggcaatggggaatgctactgcagaagtaatgtggatccaaactttgctcgatgaattgaacatagcatgtcctcgtactgctcgattatggtgtgacaatatgggtgctaaatatctatcatcaaatcctgtatttcacgctagaacaaaacacatcgaagtagattatcattttatccgtgatagagttctacagaaaagattagacgtgcaatttatatccaccaatgatcaagttgcagatgggtttacaaaaccattgactcaacagaagcttgaagaatttaagtgcaatctcaaccttgggcggttatgat

>Copia21-ZM_LTR

tgactggggttgttaagatatgatgctaatcctgataacgcgtgtgttatcagatatagtcagttagtaatatcctatattttgggactagatacggcagagtttgttcagcaatacatagatcctgtaggtcccatcttaaaggttgttactcatgtaaatctaagcatcattgctttcctatttaatagaatcatgcggcctcgacagaggtgagaacgcttcatcaatttctaca

>Copia22-ZM_IN

agtggtatcagagcctggttctctcatttggtggtcttaaccgacccgagagtaagctggatggcgactaaagggctagaggttacttgtgaaccacatatttttgatggcacacattttgcacagtggaaaacttgcatgagccattattttcatacaattgatgtaaaattgtggtggatcataagtgtaggcttttatgaaccttttgatgagaacacaaaagatctcacccaagccaaaaagaaatgcttacatcttgaacatcgagctactaacattttatatcaatggataagtgataaggtgtttaaggaaattatgtatatggagaccgctcatgatatttggacatatcttgatgacatatatgggaggatctctagcgagaatgttgagcacatccacaacacggtggttgtgaaagattgcttcacctcatggtcaagtgatgatgatgaaagacacaccacaagttcacttgataataatgattgtgttggctctaactcaagtaatgcaaatgatggttctatctcaagcatacttgatgattatggtgattgttcatgctcggacaatgatatcactactacaagcccatccattacatcacattgcttcatgtcacaaggtaatatgaaggtatataatgctaatgtgactaatcattcatattcatatgatgagcttgttggtatacttgcaagcatgaacattgctttagaaattgagaaagataaaacaagaaacttggaaaatgaaaactcatttctaaagtattcatgtgaacaacaaaagcatctactttatgttgtaacttgttcacatgaggagctaaaattgagtcatgaggaacttagtgttgctcatgaaaatttgatacaagagcatgcttttctcactaacgagttttctagtaaagaaaataaaactagtgataattcatcccatgaattatatgatcaattgaaaaatgttgctaatccatgtgatgaagacaagaagcatgtatccacctcttgtgatgatttattagctatgccatgttcttcaagtatagattattactattcttccttgtcttgtgagactaaccttttgaaggaaaataataagctcaaaaacaaagtgaagaatttaagcaacatgtttgagagatggcataaatcaaaagtcactcatgatcttatattgaaaaatcaaagaagatatgatgacaagagtggccttggtttcaacaagagcaatatcaaaggcagacaattcaaattaaaaggaaatgagctatctcacttcatgtgctataaatgccatgagatgggtcatcttgcaaaggcatgccccaataaaaggaagctcaagttgaagaaggaagaaaggaggctaaagcatgacaagtgcttcaagtgccacacttggggtcacctcacctcaatgtgcccaaccaagaaattagtgaagccacaagagaagcctcaaccaaagccacgagttgagcaaaggaaggatccccaaaagcaaatcaagattaaccatgaatataatggtgatgtggggaagaaaaagaaaacaagaagaagacattcaatatataatcaagataacatgatgagcacaaatcaaaagaagaaggatggtttggctcaaatcaagtgctacatgtgtgatgatatgggtcactttgcctcaaggtgtcccgacaatcttgagaagaaggctcaagcaaatgagaagaggcaagactatgtgaagcaaaatttgagaaagaaagagagggctcaaacaaagagaacttgctacacatgtcgggaaaggggacacatggcaaattcatgtcccttaggtaacaattcgaagcctattttaattcataagaatattatgcttagaaaggatggcaatggcacctcatttgttgcaattgcaaaacatcccgctattcatactaaggcaatgcctaagtatgttgctcctaacttaagaggacccaacttagtttgggtaccatcaaaacatggatgatcataaacaggtaccttgggcattggaggcttgattcaataatattcatttaatcatcatctattgaattaagtgttgaagactaataaaattctatcccaatgccaagtcaataataagtgaagtccaaattctaccaaatacaagtgctttattcaagttagttaaattatgaatggattgcaaataagttaagttcaaacttgtattggatgatcatgaggtatataaagtataatatttgttgatcaaattcatttgaatgcatatgagttgattgaattggatatatatgcatatgttgcttgctataagatgattgaatggacaatttgctaaataatcaagtttggattgatttatgttgaataaattcaagagacttcatttagtaagtggtttgaatggatatttgtcttttgaaagtagttcatttcagatttgattcaacttgaagaagagtgactcaatttactgaaatctggccaacattgaaaatctgacaaggctgtgctcataaccatgtttgagtgatcaaatctatttggattggcttgaaattcagtttacttgctctatacatatgatagcaaagtctatgcaaatttcataatcattggataagtgaaacttcttgattaagtcaatcttgtcagcacaaacacagcagttttcagcatgtacctactgtgggagatattatggctggcagcacatatgaagtcaaatgagtctcaactttttacagctgttagcagacttattaaagcacatctccaccaattgtcgtggtcataggatctgtacatcaaaaattatgaatgtttctttggagtatacagaatctgtcataagtgacaagtatttgaaatattgaaagtcactttaattggaaggctctcatataggataaatcacataagttgtctgagagaccatagtttggtttttaattgatctataatcatggaaagtgaaggggtacaaggatatttgttgagaaatattctggcacttatcttggtactccaagttgaagatcaagaccaaagatgatcaaagttgaggttatcatataaccgattgcataatcttctttcttacatatggcaatccaaatgcttaaatttgtttccagtgaaaagagattccatatgttcaaattggttactcctcaccaaaaagtaagaaaaggattgctagaggcaaagtggtacttaattggagcaatcaaaagattgaccgagaaagagagcaagcaacacattcaaaggcaaatcaagcaaatatgattgatgtttcaaacatggattgattttgagtattttccaagtatttttggtttgacatcaattcatgatcacttatactcaaatcatgtacattcatgcaaattatttatatatggttgaatactaatgcttggtgcagtcataaagttcaatgatcctattttaaaattcaactattgagaagttaacagtcagctcaatggaatgcaagttccaggtctcggaccgtccggtcctaggccacggacagtccgagagatcaggccacggacagtccgagctcccactgcggaccgtccgtgagtagcagtcgtgcataaacaaaggaattttctggaccaatatctccacaagtggtatcatccttacaagtgatattcctatattccgtgcaaattcataattctctcacatacaagtggtattgaaggaacaaatgcatgtgaactacaacattcaaattgtgcacttgatcttaaatgggttctcgacttcatactggtgaagaatggatttcttattgataattggtcttcaccaagctattcaattggacttcacattgctattggagagctaatcaaaagaatcttgactatcctctactccaacatagcaaaggtatcattgtgatgtgcatgatcatatcatcccctactagtatgtggttagtgcatatagtacatacttaatagtatcatgcatatttaatgaaatatttaaattcatgttctaccactattggttattttcgttgattgaaactagtggtagcatatgagtttgttcatgagcaaagtgaacccatgtacttaatttattttggattgcaacaagctacaagaacaacctcgataagataaccgcttaatgcgtgtgaagaagcttaaagagggttcaacccgaacttggaagcttaagcaaatctatttggttaagtcacatattgagctgctcatgaggatgacttatgaagaagataccaatgcaaatgaatatcaaaaccttcaagtggcatcaatatctacagagaaatgtctaggccaatattattccttcaattggtattcactaagtggtttctagatcaatcatacaattgatatcctttgagtggtacaagacttcaagtgatccacaaccttaagtgatcatcaactcaagagatcaactctctcctaaaataagttcatgcaagtgatgtcaaggatttttatcatgcatatggtgtccatcaaaaacttattaagtactttcaagttggtataatattcacaagtacattttcccagagccttagaacaaccctttttatggtaattaatgacaaagggggagaagttggaacaaagatatgtaaatgtgcaagttggaacaaagatatggaaatatgcacaagagaaaattggacatgaactcagggggagctattgatcaattgagttagaatgaatatgaatggatcaagagcaaagtataatggtatgatattttattgaacaagagatgcacacaagtagggggagcaagctcgtgaatatatgtttgatgcatttgtttgtgcactacatttcttgcttgcatcacatttgtcttatattcctacatacttgtgtgtaagtgtttgctagaactaagacttggatgatgattgatttagtgatatttataagactagtcttagtgtgaaaggagtaactagaatcctatttattataatctcatgtggtatctagtttcacttgtttctagcaagatgatgctaaggaagaaatgaaaagtagtaaacacgctacaaaggtacatttctattttgtatactttagcatcatgtggtagtgataatgtcccctcaattttcatatctttgcatgtgtgtaagcttgaaaccaaaccatccaagcacacatgttggcgaggatatcactaccaaaccgtttttatggtgcttatccaaatcttaacaaatgagcttaaagttggataaggaacataagaactttgccaaactagcaatttacacttgtgtgaaaagctagcttggaaaagaacaaaaacgacaaatctttgtagagttgtcatcaattaccaaaaagggggagat

>Copia22-ZM_LTR

tgtgagaccctgtttggttttggtaattgattgacaacttatgtggactaataagtgtttatgttgagatacacaggtgattagtccacaggtacactagtgtgagttattttagccatggtgatgatgtggcttggtgatattgagatgctcaactagtgtgataaaggagctcattgcatatgaagacatgacatggtgtcatgtggttatatggagaagatcaagagatatggcttggcttcgatggaccaagtggctagtgtgagggcaagtcacaggctaatgtgtgagggaccgcgtggcgggtgaagcaagagcaaggactttagcacccgatggacataaagcaatggtgaaagagcaagctatgttgagatttattgaacccaagaaaggccatgtgataacatggagtggatcatatcattatagaattatcaagacatgtgttgatctgggatattgatcaaggagcttgatgatgatggtggaggaacttcatgctatggacaaatggtaaaaggtatcatggttggctacacttatggatgcgcattattcatcatttaaggagatggaattgaatgctcaaggcaaaggtataacaataggttttttgttttaccggtcaaaaggtgcttagaggttgataattgaccgggttagtaggctagatagccgtactatcaagaggggtcaattatcttacttgacatgtctttagtgcctcattgctcatatagttggtgcatttgtatgagggctaacaacgctttggttcgtgaaagaaacttattcaaaagcgatttcttgaatgtgggctgaattgatgtaccgcggaccgtccgggccagcatggcggaccgtccgcaggtctaacttgagatgatgggcagaaacacggagtttctgtgtttcgtgaagtttttgtactgcggaccgtctggtcccaggtggcggaccgtccgtagttgaaggactggtgtttggacagaactcaagtagttctgttttggttttattttttgactgcggaccgtccggggcctgagtgcggaccgtccacagtacaaatatttttgtattgcggaccgtccagggtccaggtgcggaccgtccacagtacaaatattttgtattgcggaccgtccggggtccaggtgcggaccgtccgcagtacaaatttcagcaaatgtgtagagtgagcaaaacttcgttttggctcagttttatgtatcgcggaccgtccgaccttgaggggcagaccgtccgcaagtcatttttgaatgctctgacatttttataaccgtttatagccgttgggattcttgtgcggacggtccgggcttgtattgcagaccgtccgcatgtgcgcagaaaaggggcaattggcccataacggctagttttggagagggggctatatattcttgtgttgcccgagttgatagtgtgaaggatgccatttggaacacttgtgagcatattggagcctttcctctccctctctctcactcatattggttgggattgcattctagtgagattgagagccatctagtgcattgcatcaagttgttgatctttgaggcactagggagatcatcaagtgaggtcgttggcttgttactcttggaggttgccgcctcctagacggcttggtggtggttccccgtgagctcttcaaaggagattgtgaagaggccgcggtggtgattgtgagaggcgttgtgcttgcctcgccggagcggtgaaaagcaactctagtggaatcaaggtttgagggttcattgaccaatccggctcaagagatcaagtggagacttgatagaggagcggttgggagctttgaatctacctcaacgtggactaggggtgaccggcaagtcatcgacaccacgggaaaaaatcattgtgccacgtgtcatcatcttcccgttggtttgcaactttcctcactagcttgtatttacttgttcatatacttatgctagtgagtatttgttgctcgtgtatcctcttgtatatctagtcatacctataataaaatagttgctcacttgtcgttagcttgtgtagctaatctgttttacttagcttgtgtagctaagtagttgtttctctctagtagtgcttgtgctttgtgctctttgcttactagtatgtttaggagctatagttatgattaaaattacatttgcataatttatatgtcgttgctaactagatttgtatgggagagctctttcactatcttggcaccttagttgtttcaattgaaatcttttgtcaggtgtttgtaatagttagaggagtgtagtcgtggctaacccaaaatagttttaattccgtatttatttgtgttagccattgtaattagttttagaaaggactattcaccccccctctagtccgccatctcgaccctaca

>Copia23-ZM_IN

cgtggtatcagagccgaggggagaggtatacggtggatccagtggtccatcaagagagagagtgaaccgaggaagagatcccaagctccgccgctaaatcaccgtcggccgccatgggcgacacatccaccaagtctaccgtgaaggagagttccatcatgtggccgatgctcacgagcgacaactacaccgagtgggccatgttgatgcagtgcaattacgaagcgcttgaaatctgggaagtgatcaccccaggtgacaagccgaagcgggcacaagatcgacaggccatgagtgtgctcctgcgctcggtcccgaaggagatgtggcagacgttgaggaggaagaacacggtgaaagaggcgtgggaagctgtgaagacgatgcgtgtgggcgcagatcgcgtcaaggaggttaatgcccaaagactcctgaaagattttgagaacattcagttcaaggagggggagtctgtcgacgatttcggcatgagaattaccaatctggttggaaatctcaagattttgggcgaaacgatcgacgatttccgggtagtgaagaagtttctgcgcgtggcgccatcacggttctcgcagattgtggtgtccatcgagatgttcgtcgacctcaagaccctgacagttgaagagctggtcggtcggttgagggcggtggaagagcgttttgatgacaagatcgatcagatcgtggacaaggcagagcggctgctgctggccgaggatgtttggctggagaagcataagcatcgttttcaccccggcaacaagtctggagggagtggtggtggcggtgtctcctccaagggcaaggcagtagcgcgctctgacggcggcgcctcgggtcagataagactgacatccatggggacgccaagaaggaaaggccgatgccgaaattgtggcatctacggtcactgggcggaggactgcaaacgcccgaagaaggagaagaaggaggtggcgcagccggaggcgaacgtggtcatcggcggcggtgaccactctggcgctttgatggtggcaacatgcgacatcgaagaggagccgtcccaggttcatctcactgagaaggtaacacatgtgcttgttcctgatggcgtgtgggttttggataccggcgctagtaatcatatgacagggaatagatcagtactgtcacagctgaatgagggtgttaaaggctcagtaaggtttggggatggctctaaggttagaatccaaggcatgggttcagtggtgattcaagatcgccaaaagggtcacaaggtcctcactgatgtgtatttcatccctgaactgaaaagtaacattgttagtttgggacagctggaagaaaaggggttccaatatgttggggagaatgggagattgtgtgtgtatgatcaggagaaaacactgctaatctctgcacctagagttgggaacagattgtacctagctaagtttgggttagactcaccagtttgcctgcaagctcaagtggatgatgtttcctggaggtggcatgctagatttgggcatttgcatttcaaagccctgagtgatctaaaatccaaaaacatggtcactggtttgccaacagtaacaagggtagagaaagtgtgtgatgggtgtgctcttgggaagctacacagatctccatttccaaaaatgtccagctatagggcagaaaagggcttagaacttgtgcatgcagatttgtgtgggcatatctctccaaagtctcttgggggagcatcctattttttgctggtagttgatgatcacagcagatatatgtgggtggaattgctgaagagcaaggatcaggctctggaatattttaagaaaataaaaaatagggctgaggttgaatgtgatggcaagttgaaggcattgagaacagatcgggggggagagttcaattccaatctattctcaattttctgcagtgaaggtgggataaagcattacactactactccctatactccccaacagaatggggtggtagaaaggagaaatcagagtgtagttgagatggccatatgcttattgaagacaatggcactaccttctgatttttggggcgaagctatatgtactgcagtgtatgtgttgaacaggtgccctacaaagagcctgaacaacatgaccccatatgaagcttggcatggaagaaaacccaacgtgaagcacatgaggatctttgggtgtgttgcatatgtgaagttggttggacctggtctgacaaagttatctgacagatcagccaagatgattttcatcggctatgaaagtggaaccaagggctatagattttttaaccctgctactggcaagctagtggttgggagagatgccatatttgatgaaaatgtgtcatgggactgggccaatgcagcaggcaacacagatcagctgtcaggagagtttattgttaattatgaagattctgacagtaactctacaataggagatcctaaacccacagaacagtcagctgaagatgcagtttcagaagatcaagggggtgctgctgattatgatcaagctgcacctggcacacctgtcacaccaccgagccaggattcagctttttctgatcaagggccgcatagatacaggctaatgtcagaactgttggatacaactgatgaagtgtaggactatgagtatagtggtatgtgtttactggcagcagatgaacctgctagtgttgaagcagctcttgaagaaggttgttggagggatgcaatgatagctgaactgaagtctatagaacagaacaagacttgggcttatgctgatttgcccaaggatcataaggctataggccttaaatgggtgtttaaggtgaagaaagatccatctggcaagattgtcaaacacaaagctagattggtggtgaaaggatatgctcaagttcagggagttgactatgatgaggtgtttgctccagttgcaaggatggaaacagttaggctgttgttggctcttgcagcacagggtgaatggcaggtgcatcatatggatgtaaagtcggctttcttgaatgacaatctggaagaagaggtgtatgttcacccaccgcctggattctctgatcccaaacatttagggaaggttctgagactcaagaaagcattgtatggcctcaaacaggccccccgagcctggaatgcaagacttgatcaagagttgaagtttttgggtttcaataggagtgttgaagagcatgctgtgtacagaagaggtacaggcaactctctgatcctagttggagtctatgttgatgacctgatcatctgtgggccagacagcaagaacattgttgtatttaagcagcagatgaagaaaagttttgacatgagtgaccttgggctgtttacctactatttgggtttagaggtgaagcagaaaccaggggaaatcactgtgtgtcagagtgcttatgctgaaaagattgttgagatctcaggaatgaaaggttgtaaccctgctgacacaccaatggaacagcatatcaagctgttgccgggcaagtcagagctggtagctaatgcaacaaagtacaggagcctagttgggagtttgagatatctggtgaactcaaggcctgatctagcttattctgtgggcatgattagcagattcatggagacaccgaactcagaacattggggggcaatgaagagaatcattagatatgttgcaggtaccaccaagcttggttgcaagtatgtcaaaggagcactttcagacttggttggtttcactgacagtgatcacgcaggtgatctggagaagaggaagagtacatcaggggtgttgttcttctatggtggcaatgcagtttcttggagttcacagaagcaaaaggtggtgtctttatcatcttgtgaatctgagtacatagctgcagctacaggagcctgtcagggagtgtggcttagcagattggtggcagacataacctgtagtgacgtgaagaagttcaagcttttcatcgacaacaggtctgccgaggagctgagtaagaatccagtgtttcatgagagatcaaagcacatagataccaggtaccactacatcagggaatgtgtggctgatggggtgctggaggtgcagcatgtaagcactgaagatcagctcgccgacatactgacaaaaccacttgcaaggattcgctttgcagagatgcggaggcagctaggggtggttaacatttgacagattaagggggtgaaa

>Copia23-ZM_LTR

tgttgcattgatctctttgtaatcgtgtcaaatgaacacgtatctcattttgatgcatgtaggtagtttgttttagtaacaaatcatgcatatttggcgtagaatcgcgggtgagctggagactgcccaatctcacgccgtgaccgcatttttgtcaggaaagaaccaccgcagcagatttgccgtgcatgtcgcactgggcgttccggaaataacggcgcaccgtgcagcgatctgttgcggtggggaacacgggggaatccctctataaaaggaacaaggattgcattggtgttgattgcacgcatttgcagagaaatctcgtgtcctctcttgtactctcgttcctaggtctgtagcgtccgcggtcaccaccggccggcgtcgcggactcgccggcgtcgatcttcgacaggtggttccaaca

>Copia24-ZM_IN

tggtatcagccgcggtttcgttccgctacacttccgcaaccaccactcgtcgcagatacgatcgccaccaccgtcgctgacttctcgtcgcagggggtgccgccggtcttcatcgccgagcgctcgcccagtgtgctgggcacttccaagttccacctcgccgggctctagcagccccgatctggtcgccgtgctgcttcttcctgatggagattaacgctgctgcagccacccaagctgcagaggctgcggctgccgcagcccgcgcggaggacgcgcgccgtgctgaagaagctcgccttcgcgctgctgccttggacgactacgagcgcgcacacgaggccatctgggcacaggccaccgccgtcgtcaacgttaaggccctcatccccgtcgtcctcgatcaggctgcgaacaactacaccaagtggcgtggcatgttcctcatcgttctgggcaaatatgcccttacccgccacgttctggaagacgacgccttccctgcgcgtccggcgtgggctcaggcggactgctgtgttctgacgtggatctacggcaccgtctccaacgacctccagcagtccctaatgatgcgacaacgtcctgcgcgcgaggcttggcgctacctcgaggacacgttccttggccagaaggaatcccgcgcgctcctcctcgaaacgcagttccgcaacctccggcaggactccatgagcatcactgactactgtcgtcgtcttgagtcgatggccgcttccctcgccgaattgagtgatcccatcggggatcggcagatggtgctcacgcttctccgcggactcagcggcaagttccgtcacatggtgtccatcctcaagctccaccagccgttcccgacgttcgcgcaggcccggacacacctcctactggaggaaatggagatcgacaaccgtccaccgtctccgccatccgccatcgtcgctacagctcctcgcccagcgacaactgggacgccagcacctccacgctcggggacgcctcccaccacgcgccctcctggtggccagcgcaacaaccgccgtcgtggccgcggcggccggggcaacccccagtcatcttatggtggtgttcctacaggccccaacggcagtgttattcccggtgtccatccatccttccagcatccgtggcaaggcaccgttcgcatgtggccttacgaccagtctgggcgttctccaccgcccccacctgctttcagtgccgttccccagtatgccggctttggtggcgtgcccaactcctacggcggcatctacgttcccccacctcctcagtacggcacgtactacgggggagctgcgtctccggcattccaggcgtcttcgccaggctcccagactctacccgcgtcaacgccttggaaccccatccacggcgggtcttggcatcaggactcgcttgcgcaatccttcaacaccatgactctgaacccaccggcatcttcttctgagtggtatgctgattccggtgccggttcacacatgacatctgacgctggtaaactttccaatatttccccaccttcttcctttactccttcctctattattgtgggcaatggtgccttactccccgtcactgccactggatcatccattttttctttaccacatcgtaatcttgttcttaacaatgtcttggtgtctcccaacattattaagaatttaatttccattcgtcgcttcaccactgacaataattgctccattgagtttgatccttctggcctttctgtgaaggacttgcacaccaggaacgtgatcgccaggtgcaatagctccggtgacctctacccgttctatctaccatccaccagcacctccgcgttcgtcgctgcacctatttccctttggcatcgccgtctcggacaccttggccatgaagctctgttcaagctccttagctccagtgctatttcgtgcaataaaaatgatgtcaagcatatatgtcatgcttgtcaacttggtcggcatacacgcttgcctttcagtttatctagctctcgagctgtttgcaattttgatttaatacactgtgatctttggacatctccccttgttagtgtgtcaggctacaaacattatcttgttattcttgatgattgttcacactacatttggacgtttccgcttcgccagaaatccgagacattttccaccatttccaattttcttgcttacattcgcacacaatttggcaccaccgtcaagagcatccaatgtgataacggccgtgaatttgataattctcaggcccgcacattttttctttctcacggtattgcactgcgcatgtcgtgcccatatacttctcaacaaaatggaaaagcagaacgttcccttcgcaccataaacaacattctgcgctccctattatttcaggcgagtctccctccggcctactgggttgaagcgcttcacaccgcaacatatcttgtgaaccgactccccactaaaaaccctcgccttctccacaccatacacctctctctactccaccaaaccttcctatgatcatcttcgagtttttgggtgtgcttgttatcccaacatgtcagccacagcacctcacaaactctctccccgctcatccctatgcgtctttctagggtattcatcagaccataaaggatatcgctgtctcgagctccagtccaatcgcatcattatttctagacatgtcgtttttgatgaatctttctttcccttctccgacatgtctaccacacccatggctccttctgtcctggattttttaaccgatgaatctgatcttaccactctagttcctggaactcgtattgtgcatgcaggtaccactactccaaccggtgctgtgcatggcgctactcccgtgcatggacctgttactgcacctgagcgctcatgggcgtcggattttggcgctgctgcccctgtgcctctcgcaccggttgctgccagcagcgccccgtccacctcgcatgtgccgaccggtgctgctgcccctgtgcatcccgcacaggttgttgccagcagcactattaccatgacgcccccatgtactgccggtcctgctgcccctgttgctcgcgctcaggttgttgccagcggaaccggccgtaccactgctactcaaccatgcgccatcaccccagtcactaatgtgcaccccatgcgcactcgcggcaaggccggcatcgcgcagcccgtggatcgccttaatctccacgctgtgcccatgtcacctttgccgcgctctgttcgtgatgctttggccgaccccaactggcgctctgctatgcaagctgagtatgatgcccttctcgccaatgacacctggagtctcgttcctcggcctcctggagttaatcttgtgactggcaaatggatctatcgtcacaaattacttgcagatggttctcttgatcgctacaaggcgcgctgggtcctgcggggtttcacacagcgccccggcattgattatgatgagactttcagtccagttgtcaagcctgcgacagttcgagtggttctctctctagctgtatctcaaaattggcccattcatcagctggatgttaaaaatgcatttttgcatggcacattggctgagacggtgtactgtgttcaaccgactggctttgttaacccatcacatccagattctgtttgtcggctcaacaagtctctctatggcttaaagcaggctcctcgtgcttggcaccacaggttcgcctctcatctcatgtcacttggatttgtcgagagcaagtcggatacttctctattcacatgtcatcgggggtctgacacggcctatttgctgctatatgtggatgatattgttctcaccgcctcgtcagctagattcctccaacatattattgcagctctccagcatgaatttgctatgactgacatgggccaacttcaccattttctgggcatttccgtgacacactgtgcagatggtctgttcctctctcagcgccagtatatattggatattttggagcgtgcagggatgagtgcttgcaagccttgcagcaccccggtcgacttgcactccaaactgtctgcagatggttccccggttgccgactccacacagtaccgcagccttgctggtgcattgcagtacctgattttcactcgccctgatattgcatatgctgtccaacaaatttgcctttacatgcatgatcctcgggagccacatctcgctgccctcaagcgaatactacggtacctgcagggcactcttgctctcggtttgactatgcgtcgttcaccgccgactgagcttgttgtctacactgatgccgattgggccggctgtcctgacactcgccgatcgacctctggttatgcagtatttcttggagacaacttggtgtcctggtcctccaagcgtcaacacactgtctccaggtccagcgccgaggccgaatatcgagctgttgctaatggggttgctgaagccacttggctccgccagttactcatagaccttcgtcacccgccgcgtcgtgccactctggtctactgtgataacatcagtgctgtgtatctctcttgcaacccagtgcaacatcaacggacaaagcatgttgagattgatctccactttgttcgggagcgggtcgccctcggccatgttcgggtactccatgttcctacgacatcccagtacgctgacgtcttcaccaagggtcttcctacttcattgtttcaagaattccgatccagcctgaacgttcgccatgctcctgatcagactgcgggggag

>Copia24-ZM_LTR

tgttagcaatcttgcatatcttgtatttatagtaacctctctccttgattgaaggctgtaatctctccctgtgcggttggcttgcatgtcacccacgggctatttatgtacccctgtacgccatatggaatacatcaattattccctaaactattaca

>Copia25-ZM_IN

tggtaatcagagccacctccttcaaaccctagccacccccagcccatagcagacgccaggagatctcatggcaagctcatcctccatggctatacctacacctttgctagggcaaactatagctgagaagctaaccaagtcgaactatgcgctctggaaggtgcaggttctttccatccttcgtggcgcacaactgtagggctacattgatggctctgccaatgcgcctgaatagcagatcgacgtcaagaacggcaacaagacagtgaaggaaagcaacccggagtacatccagtgggctgtcttggagcagcaggtcctcggcttcctcatcacctccatgtccaaagaagtcatgggtcaggtatcatcctacaccacgccccaggaggtgtggaacatgctagaacagacctatgcagtgcagtcccgggcaagaacagtcaacaccaggattgcattggccatgactcggaaaggtaacttgtcaatttctgagtatgttacaaaagtgaaggcacttgtagatgagatggcatctgctcggaagcccattgatgaagaagaactagtctcctacatccttgcaggactagatgaagaatacaaccctgttgtctctgcccttgttgcacagaaggattctgtctcagttggggaggcgtactcgcaactcctcaattttgagagcaggatggctttgttgtatggaggatcctagccatcggctaatgttgctactcgtcatggtcgtggtggttctggcctgcatggccggggtcgcaatggtcggtttaatcctggtcgcatcagcaacatcaatgcgccaggagggcgtggtggtccaggagggcacagatctgatgggaacacaataacctgtcaggtttgtgacagaactgggcattctgcccgcaagtgctggtaccgatatgatgaagacaactattccaactcaaattccaagagtgcagcagcagcaatgcacagctatggtgtcgatacaaattggtataccgatacagctgcaacagatcatctcacgtctcagctggacaaactgaccactcatgagaagtacaaggggacatatcagattcacactgcaagtggtgcaggtattaacatctgcaatgttggtcatgctgttattgatacccctactaaacccttgcatctcaataatatcttgcacgtcccaacagcacaaaaaagtcttgtttctgttcatcgtttctcatcagataatcatgcctcacttgaatactttccaaatcattttttgattaaggatctggacacgaggaaggttcttcttcaaggtagatgcaaggatggtctttatccccttccatcagacagttcttggcagggggcatttggcgccatcaagtcctctccatctctatggcacaatcgtctgggtcatccatcattgcagatcattcgaaaattagccaataataatcagatcatatgttctagtgagtcaagtagtctgtctgtgacgcttgtcaacaaggcaagagtcaccaattaccttatccagtgtcaacaagtgtctctaccaagccttttcaactcgttttctcggatgtatggggcccagcccccgagtccgttggtcgaaaaaaatctatgtcagttttatagatgattttagcaaattcagctggatttatttgctgaaacataagtctgaagtgttccaaagatttactgagttccaaaccatggttgagcgcctatttgacaccaaaattattgctatgcaaacagactggggaggcgagtatcagcacctaaattctttctttactaaactcggcatcattcattatgtgtcctgcccacatgcgcatcagcaaaatggacctgctgaacgcaaacatagacatatagttgaagttgggttgtctgtccttgctcaagcatccatgtcgcttaagttctgggatgaggcttttgccatagctgcatacctcattaatcacacccccacgaaacttcttgattactctacacctcttgagcacttgttcaaacagtcccctgactataattttcttaaagtttttggctgtgcatgctggccaaacctgcggccctataatactcgaaaattggctttccgctctacaagatgtgtctttctaggctacagcaatatgcacaaaggatacaagtgtctagaggtctctactggtcgtgtctacatctctagggatgttgtgtttgatgagcacatttttccctttgctgaacttcatgcaaatgctggagcacgtcttagagcaaaaattaatctcctgccttctaccttatttccttcatccaaccttgacaataggggcatttttaatacagctgatcacactttgcctgatgttactgaggctaatgcaattgatgatgttattcctagtgtggtctctgatgtcagaatgcaggagataggtactgagcagcttcaagtgcatgagcaggagcaggttcagcccaagagtgcaacaaaacaatatatgaaaccacctatcactaccacgtaccagagaagagagaagaacatatcaactccaccttcagctgcattaccagaaactgccacaacacctttttcttctgttgttccttcacaagaacctactgcagctccttcaccatcgtttcaaagtgcttcagaatcagaagaacatgtgtctctcatggaaggaactgaaagtgcttcagctgcaggtggttacatagatctgagacctcgtactcggcttcagaatggtacaatacagcgcatcaagtatggctgtttggctgccactagtgagccccaaatattagcgaaggccttgggtgatgaaaactggaagcatgcaatggatgctgaatttgaggccttggctaagaacaagacttggcacttggttccaccagaaggaatcaaaaatattattgattgtagatgggtgtacaaggtcaaaaagaaggcagatggtactctggatagatacaaggctagattagtctctaaaggcttcaagcaaaggtacgggattgactatgaggacacatttagtccagtagtgaaggcggtcactattcgggttattctctctctagctgtttcacaagggtggccactacgacaacttgatgtttagaatgcgtttcttcatggcattcttgaggaggatgtatatatgaagcaaccaccaggttacgaagacaagctgcagccacaccacatttgcaagcttgacaaggccatttatggtctgaagcaggcacctagagcctggtattccagactcagtgacaaactattgcagctaggttttcaagcatccaaaggagacacctctctattcttttacatcaaggaaggtatcaccatcttcttgcttgtatatgtcgatgacataactgttgcaagttcatctcagaatgttgtctcagctttgctcaaggatctgaaaactgattttgcactaaaagaccttggcccattacattattttcttggaatagaagtgcagaaggtagataatggtattcacctctcgcagagaaagtatgcctcagatgttcttagtcgagttggtatgataaattgcaagcctaccactacaccactctctacttcagaaaaaataagccttcacaatggtgaaccccttgggcctgaagattcaacacggtatagaagcatagtaggtgccctacaatatctaactttgactcgtccggatctcagttttgcagtgaacaaagtgtgccaatatttgcattcacctacaacagatcactggacagcagtcaagcgtatcttaaggtaccttcgatacactcttcaccatgggttgaagatttcaaagtcaccttctctgctggtcagtgccttacagggggatttgctgtttttttgggcagtaatttggtatcctggagcgctcgcaaacaaccaaccgtgtcaagatctagtactgaggctaaatacaaagctattgcaaatgccactgctgagttgatgtggattcagtctctattgaaggaactgaaaatttcttccccccctacagcaagaatctggtgtgacaatattggagctacctatctcacagccaatccagtctttcatggtcgtgttaagcatgttgaaattgacttccattttgtgcgtgaaagagtggcaaggaagctcctggatgtgagattgatttctacaaatgaccagatagcagatggtttcacaaaagctctgacgaagaagaagatggatctgttcagagacaatcttaacttatgcaagttatgattgagggggga

>Copia25-ZM_LTR

tgttagataatattaagaatgtatctgtaaagtaaggaaagggtgatgcgcctgtgtaaagataggaaaggatctgttcagttatagatataatctgaggcaatcaatcagatctgttatagatagaatccaatcaatcataatctgttttgattgatttgattgattgctggcgccagcccctttgtaatctgatgatggtattccatctcctataaatacatacaggcggcctactacggtaggtaacaaacgcttcccaacctggtgttctttca

>Copia26-ZM_IN

tggtaccagagccacgatctagacaccgagaaaatcgccatgtccaccatgtccaccgtcgatggcgtcgccgccgtgttcagctcgatgccgacgccggcgttcagccagatggtgagtatgaagctcaaccatgagaactaccttctctgggtggcacaagtcctcccatatctgcggagccaaggcttgagcggccacatcgatggatcgctcccagcgccaaggcagactgtcgccgtcgaccctgcagaaggctccggcggacggacgatcgccatcaaccccgagttcacgagctggtaccaccaggatcagctcgtcctgagcgtcatcaactcatccctttccgaggaggtcctggccacagtggttgatgccactactgcccgcggcgcatggagcactctggagaggatgtacgcgtcaagttctcgcgcccggatcatgcagatccgaatgcagctcgccaccatacagaagggcgatctcactgccgcagaatacttcagaaaggtaaaaaggcttgctgacactctggccgctgttggcaagcggcttgaagatgaagagttcatctcatacctccttcgtggacttcccgccgactacgactccctcgtcaccagcatcactacaaggccggacacctacaccatcagcgacgtctacgctcaccttctcagctttgagaccaggcaagaataccatactgctgttggtcagatatcctctgttaacaatgccaacagggtaccaagccgtggtggaggagcatttggccagaatggccgtggagggcgtgggcgcggtggacgcagccagcctgggcgaggcaacaacagaccaccagcaaggccaccgagcaacaacagcagttccagtggcacttgccagatctgtggaaaagggaaccacaatgcacttcagtgctggcatcgatttgatcaagcctatcaggcagagagcactgtcaagcaggctgcagcagcaacacatgagtatgcagttgatcctaactggtacatggacagtggggcaactgaccacattacaagtgatttagagaggcttaccaccagggagcgctacactggaggagacaagattcaagtggcaaatggagcaggtttgtctatatctcacattggtcattcttcagttcctggtttaagtaggcctctgtatctggatcatgtcttatatgctcccaaaataaataaacatctaatctctgtcaggaaattagctattgataacaatgcttatgttgaacttcaccctaattttttccttgtcaaggatcaagccacgaagcatcttcttctcagaggcaaatgtaggaatggcttatacactctaccaaataattgtcaagccttgctagcagccaagacttctcctgatctatggcatcaaagacttggacatcctgcccttccagttactcttagaattttgcagaacaataatattgctgtaaacacagaggttttaccttccttgatatgtaatgcatgtcaactaggaaaatcccatcagcttccttttagcagttcttcacatgtctccactgtacctcttgagcttgttcatactgatgtttggggccctgcactttcatcagtaaataattcaaagtactatgtttctttcattgatgactttagcagatatgtgtggatttattttctgaaaaataaagctgatgttgagaaagtgtttatgcaatttcaacaacatgctgagagaatgctaaacactaaaataagggctgtccaatctgattgggggggagagtatcaaaggctgcacagatatttccaagccactggcataagccacagaatttcttgccctcatacacatcaacaaaatggtctagctgaaagaaaacatagacacttggtagaaacaggtttagctctccttgctcaggctcatcttcctctcagattctgggatgaagccttcaatactgcctgctacctaataaatagaatgcctagcaaaaccattaaccatgacactcccattcataaactgtttggcacaaatcctgattacactcagctcagagtttttggttgtgcttgttggcccaacctgcgtccctataacaccaaaaaactcaacttcagaacaaaacaatgcactttcattggttatagctctgctcacaagggttataagtgttttgatcagtctacagggagagtctacatctctagagatgtggtgtttgatgagcagatcttcccctttgctagaaaatcatcagaatccacaaaatatccccaaacatcacaccatcctatcatgctacctgttctgtccaaaaacattcagtacactgaaaactctcttatgcagggcctgtttggaccagtagctgataatgctaatgttggggttaggcactctgttcctaatgctactaatgatgtttcccatgatagcaatcctttggcaggtggcattcttaatcaatctgaagaagaggggcaagaatcagagtctgaagcacattcaggctcactgcagcaagaaactccagaacctgagcagattcagggtgaagttgagagaaatcaacaagagcaagaggttcagcatcacatgagaacaagattaaaagacaacattgtcaaaatcaagaaactcactgatggtacagtcaggtatgctcagcaaggcagaggatttgtggtcactgaagaaaatccgacaacaactgcattgattgcctcagtccagaattcagtcagtgaaccatatgatctgcagcaagcactaaaggatccaggctggaagcaagctatggatgaagaatattcagcattgcaaagaaatcagacatgggaactggttcctccaagagcaggagtcaatctgattgacagcaaatgggtgtttaaggttaaaagaaaagctgatagttcagtggaaagactgaaggccagattagtggcaaaaggcttcaaacagaggtatggcattgactactttgacactttttgtccagttgttaagccgaccaccattagaattattctatcattagctgtcagtcagggatggagtatgagacagattgacatccaaaatgcttttctccatggactccttgaagaagaagtctatatgaggcagcccccagggtatattgatccaaacaagccaaattatatctgcaagttgaagaaggcactttatgggttgaaacaggccccaagagcctggcactcaaggctgaccaggaagctacaagaattgggttttcaggcttcagtagcagatgcttcactatttgtgttcaagcagaatggattgtcaatttatatgcttatatatgtggatgacataatcatagtcagctcaaatgattcagccacagataagctcatcaagaatctagcagatgattttgctgtgaaggatctaggaaatcttgagtacttcctaggaattgaagtgaagaaaacaagagaaggaattctgttgtctcaaaaaggttatgcacttgatctcctgaagaaggcaaatatggagaaatgcaaagcaatttcaaccccaatgtctgcaactgacaaactgtccaagaatcaaggaacaacactgaatgagaaagagcatttcagatataggagtacagttggtggactccaatacctgacactcactcgacctgacttatcatttgcagtcaacaaggtgagtcaatacctacaaagtccaactgatgtgcattggacagctgtcaaaagaatacttcgatttgtgaaaggaacaatagactatggactaaaaattcagaagacaccttcagtcatgttgtcaagcttctctgatgcagactgggctggctgcccagatgacaggaaatcaactagtggttttgccatcttcttgggtgataatctagtggcatggagctcaaggaagcaagccactgtgtccaggtcgagcaccgaggctgagtacaaggcaatagccaatgcaacagccgagctgatttggatccaagcactactgaaggagttgggaatttatttgcataggccaccaaggatgtggtgtgataacgttggagcaacctacctgactgcaaatcccaccttcaatggcagaacaaaacatgtcgaagttgattttcactttgtcagagaacaagttgctaggaaggcaatggaggtcaggatcatttcctctaaagatcagttagcagatgtcatgactaaacctctgtcaaaggcaccatttgtcaagaattgcagcaatctcaacataggtactctgttgtgattgagggggac

>Copia26-ZM_LTR

tgatagaaaatgtgttttccaccctgtgtacgtggctggcttagggcgtaaagttcggcatgcagtgactgtgtctcgttctgttaaggacgtggtcactgtgaccgagtattcaggcgtcactagtgcacgctccccgcgatcttatcacgatctgttgtactctgatgtaaacttgattgagctataaatagaacacgatcccatcgaggatcggcgcgccttttcatcaaacttttca

>Copia27-ZM_IN

tggtaccatagccagcctcttttttcatccatggcgcatgaattctcctccaccatcccctcctccttctcaattccagtttctgaaaaactgaaccgcacgaattacgtgctctggcgtgctcaaatcctgccgcccatccgcgcggcgcaactcgaagacgttctcacgggtgctgatccaaaagccccccggcccgtatcgcccagcaagatcggcgatgccctcgacgaccgaacccaaaccccgagtatgctcggtggattgcgcgtgatcaagcagttttggggtatctcttgtcgtcccctgacacgagatgtactcaccaacgtcgccacgctgccctcttcggccgaggtatggagcacactggccgcaacatatgcgtcccgtacccgtgctcgctccgtcaatacgcgcattgcgctggcaaccacaaagaaaggcactgccaacatgacggacttctacaccaagatgaaaggctatgcagatgagatgagtgctgccggacagccccttaacgacgaagagtttgtatcctatctcctcactggtcttgatgaggaacgctacaatcctctcgtttcatctattcttactcgcatagaacctgttacaccttctgaattactctcgcaaatgctgagttatgaactgcgcacggagaggcagtcgggggcctcctatcactcatccgtgaacgcggcctcccgtggtcgcggatcttcgtcgaaccgtggcgccaacaaatggcgtggacgcggtcgccccccgtcacgtgatcaatcatctgtcatgtcacgcgggggctcttctggtggatccaaccgtcgctcttccgctgattcatctgggggccaatctcgtcctcactgtcaaatatgtttcaaaatcggacacacagccaacatctgctggtaccggtttgacgaggatttcgtccctgatcaacgtcttgctgcatctgcgacgacatttcaagccagtgattcacaatggtatttagactccggtgctactgatcatatcaccggcgagcttgataaaatgaccgtgcatgaacgatataatggcatggaacaaatcaaggcggcaaacggcgcaggtatgaatattgactgtattggctcatctgtcataccttcgtccacccgccctctacaccttcacaatattcttcatgttccccttacacaaaaaaatcttgtgtccattcataagtttactctagacaataatacatttattgaatttcatcctcatgtctttttgatcaaggaccaggccacgaggaaggtgctggttcgcggcccctgtaagggtggtctctatcccttaccgacccagctgaaccccactcagaagctccttctatctgccatcaagtcttcacctcatcgctggcatgctcggctaggtcatccggctcatgagatcgtccttcgtgtcattagagataatggtcttttatgttccactctaaataataagtcatccgtttgtgatgcatgcatgcgtgccaaggcacgacagcttccatatcctatttcggttagtaggtctagtgctcccctggatctcatcttttctgatgtttggggtccagcaattgattctttcggcaacaataaatattatatcagtttcattgatgattatagtaaattcacttgggtttacttgttgcgtcacaaatcagatgcattcactatatttcatgagtttcaaaaccttgttgaacgcatgttcaatcgtaagatcattgctatgcaaacggattggggtggtgagtatgttcggctcaactctcttttccggaaaattggcatcactcatttagtctcctgccctcacgctcatcaacagaatggcgtggccgaacgaaaacaccgacatattgttgaggtaggattagctctcttagccactgcatctatgccattgaaatactgggatcaggcttttctcactgccgtctacctcatcaacagaacacccaccaaacttctaaattttgatacaccccttcataaacttcttggctccacacctgattactcggccctccgcatttttgggtgtgcctgctggcccaatttacgtccatacaattcccataagcttcaatttcggtctgtccgatgtgtgttccttggctatagtgttatgcacaaaggctacaagtgcctagatatctctacaggacgtatatacacatctagggatgttatatttgatgaaaatatttttcctttttctgaacttcattccaccgcgggcgccaggtatactgctgatattcttcttcctgatcctatttccacggttcctgcagatgagcctgcggtcaatactcatgcatgtgttttaccaattgttccaaatatgttgcctcagcctgttttgcagccactaacaatcccgggcgcagaatcctcctcagtttcccaaccaacagaaccggtcacagcagtcccgggagtctccgtaccggcgtcctccgctcctgccggatcgaatccggcgctgcctcgcctcgagccgctccaggccgcactcgacgccatgcaacaaccagacgccgcggtgggccccgacattcacgcgtcgatcagtacggacggtgttgttgcggatcccgccaattccgtgcggatccatcccgaggaagctgctgcaggcgcatctacctctgtttcagcgcctgatttgccgctaccaccggtacctgtctccgccgaatctgcacagggggccgtgccgaattttgctccacgcacccgactgcaagcggggattcgcaagcccaagcgtttttctgatggcacggtcccatatggccttgctacagtatctgatctggaaccaacatccctacgggaagcattgagtaataacaattggaaacatgctatggaatctgaaattgctgctcttcaacgcaacaagacttggcatctcgttcctcctgacaagaaccggaacttgatagattgcaagtgggtatataaaatcaaaagacatcctgatggctctattgatcgatacaaagctcgtcttgttgccaaagggtttaaacagcgttatggcattgattatgatgacacgttcagtcctgtcgtcaagtttgctactattcgacttgttttgtctattgcagtgtccaaaggttggagtctacgccaattggatgtacagaacgcgtttcttcatggtgttctagaggaagaagtgttcatgaagcaacctcctgggtttgaggatcctgtggttccctcttatcattgcaagttagacaaagccctctatggacttaaacaggcacctcgtgcctggtattcacgactcagcatgaagctacaagctttgggatttattccatctccagccgacatatcacttttcatatttcatcgtggctcgatcactatttatgttcttgtgtatgtggatgacattattgttacaagctcctcaccgcaggccattgatgctctgcttacggatctcaagcatgactttgctcttaaagaccttggtaagcttcattatttcttgggcattgaggtaacgcatgtaccagatggtctcatacttcaacaacacaaatatgctactgatctacttcgcaagtttgggatgtctcagtgcaagccaatggcgacaccgatgtctacttctgagaagctgacagcacactctggtactcctctcgatcacgatgatgtcacaaaatacagaagcatggttggtggtctgcaatacctcacactcactcgtcctgacctgtcgtttgttattaacaaagcttgtcagtacttgcaatcacccacctctacgcacatggcagctgtaaaacgcatcatgcgatatgttcaaggcactctaactattggtcacaagatccgtcagtgtaactccacaatgctcagtgcgttctcagatgcggactgggtcgggtgtgctgatgatcgcaagtccacaggtgggtttgcagttttctttggctcaaatctcatctcctggtgtgctaagaaacaaccgacagtatctcgatctagtaccgaagcagaatataaagctatggcaaatgctacggctgagttaatgtggctgcgcagtttgatcaaggagcttcacattcaaagtcccaaagctgctcgtctctggtgtgataatatgggtgccaagtaccttgcttccaatccgatatttcatgggcgcatgaagcacgttgaggtggattatcactttgttcgtgatgaagttgttcgtcggctcttggatgtacgatttatctctaccgatgatcaagtcgcggatggcttcaccaaaccaatttcagctcaacgtctgcatgagtttcgtcacaatctcaaccttcagaacggctgagattgaggggggg

>Copia27-ZM_LTR

tgttagacagaattgtaatccctatcctctggtgcctcgggtggttagttggttagccatagatagagattaggctatagataaagataagcctcggctgtgtaatcttaaccacctgaaactctatccgtgtatacacccttcgcccggtatacggcgctatataaacatgcaacctgcggccaccaatggtggttgaacgcttcatcctattaggtttaca

>Copia28-ZM_IN

tggtatcggagctatcaccaaataggaatcccacgatcccatcttcaagcccttcagttatagctctcatggctggagcattatcatctactatcccaaacctcaccaccatgaatctccatcctattgttgacaagctcgacaaaggaaatcatccaatctggcgcgcacaagttatggctaccattcgtggcgcgcgccttgaagggttcttaacaggaaagaagacgaagccagcagaagaagtagaaatcaaggatgctgatggcaatctcatcaagactgcaaattcagaatatgaagactggatcgctgcagatcagcaggttctcagctacttgctcgcatctgtgtccaaggacattcggatgcaagtggcatctaagagttccgcagcagaagcatggagcaccattgaagtgctcttcacatcccagactcgtgcgcgtgctgtcaacacacgccttgcactggcaaccacaaagaagggtgcaatgaaggccaccgagtacattgccaagatgcgtgcactcggtaatgagatggctgtcgctggccgtcctttagaagaagaagaattggtcgagtacatactagcaggacttgacgaagaatatgattcggtggtgaactcagttctcgccaagaccgagcctaccaccgtaagcgaactagttgcccagatttttgcttttgaaactcgtgttaatctacacagttatgattcttctggatcatcggtgaatgccacaagccgtggtcgtggccgtggtggaccatcacgtagcggcttcagtcgcggacgtggtggtggtcgggcgttcaacagctcgacacaaagtggtcgagggtccttccacaataataaccctcggtctaatggtcgtagtgtcaccaatcgcccacaatgccaagtctgtggcaaaattggacacacggccgatcgttgctggcatagattcgacgaaaatttcactccagatcagaagcatgttgctgcagctgccacaaactcatacaatgtcgacacaaactggtatatggactcgggagctactgatcatataacaggagaacttgacaaattggttgttcgtgacaagtaccatggcgcggaacaagttcacacggcaaatggagcaggtatgaaaattagtcacgttggaaaatctattattcataccccttcacgaaatcttgaattacgcaatgtccttcatgtcccacatgcaacaaagaatctgatatctactcatcgattttccttagataataatgtattttttgagattcatccttggttctttcttgtaaaggatcgggacacgaggaccactctccttaaaggaaattgtcaccgaggcctgtaccctttgccatcctcttgccacaagaagcttgctcttggagtcaataagccatcccttgtcagatggcacgaccgtcttggtcatccagcttttcaagtcgttgaaagagtccttagagattataatcttccacatcaagttgagtccaataaagagtatgtttgtggtccttgccaacaagcaaaaagccaccagcttccatattcaaagtctagtagtgtgtcaaatcaacctctagaacttattttttctgatgtctggggacctgctcccgaatctgtaggcagatacaagtattatgtcagttttgtggatgattatagcaaatttacttggatctatttattgaaattcaagtcggaagtttttaaaaaatttcaagaatttcagaatctagttgagcgcttgttcaatagaaaaattattgcggttcaaagtgattggggaggagaatatgagaaacttaacccttttttcactaaaattggcatctctcatcatgtctcgtgtcctcacgctcatcaacaaaatgggtctgctgaaagaaaacataggcacattgttgaggttgggctatctcttttagcacgagcatccatgccactaaaattttgggatgaagcgtttcttgctgccacttatctcataaatcgtacacccagcaaagtccttcaatactccacatctctagaaaaactattcttggttaaaccaaaattttcctccttgcgtatttttggttgcgcatgttggcctaatcttcgccctttcaatttcagaaaacttgaatttcgatccaaagagtgtattttcttagggtatagcaatatgcacaaaggcttcaaatgtcttgaggaaaatacagggcacatctatatttctagagatgttgtttttgatgagaatatctttcccttctctaaattacataaaaatgcaggagctaaattaagatctgaaatattacttttaccgccagtcttgcttcctagccatttcgggggtacgaatatggattacgtgactaatctttctaagaatcaagatactgcgacaagctgttctgtgcagattccagcagaaaaaaggagcatgcaaggtgcccctacaagcaacgtgggcaactctgcagcatgggagcattatccaccactaccaatacgtgtaccaacatgcagtacgccaccagcatgcagcacgccatctgctccaatctgtgcaccgcctgacagctctcctgtaaatgtatgtgcaccgcctgacagctctcctgtaaatgtattatcttctgcagcacaccgctattttcttcacccacgcgtccagaggatatgcccagcattgccagcacaacgcctgatcaagctggaccttctgcggcacaagggggagaaattgaacaagcaatagttcccataccgcaagctccagcaatagagaaaaatccaccgaggacaagacttcagcatggtattcgacaacccagagtttacactgatggtactattcgctatgcttttcttgtctctgctgacagtgagcctagaaatctcgaagacgctattggtaataaaaattggaagcatgcaatggatcttgaatttaatgctttgattaaaaataacacatggcacctggtacctccatgtaaaggcagaaacattatcgactgcaaatgggtctataaaattaaaagaaaggcggatggcagtttggatagatacaaagctagacttgttgcaaaaggatttaaacagcagtatggagtagactatgaagaaacatttagtcctgtagtcaagcctgcaaccataagaacaattttgtgtttagctgtgtcaaaaggatggtgcttgagacaattagatgtgaataatgcttttcttcatggatatttagaagaagaagtttacatgcgtcagcctcctggttatgaagataaaacatgccctgattttgtatgtagactagacaaagctatatatggattaaaacaggcacctcgggcgtggtattttaggctcagcaacaagcttctcgaatttggatttaagatgtctttatccgataactccttattttatctggataaagatggtgcaaaaatgttcattcttgtttatgtggacggcataattgtggctagtgcttcatcacaggctgtcacaacccttctaagaaaacttggagatgagtttgctcttaaggatttgggagatctgagttactttcttggaattgaggtaaataaagttaatgatggtataattcttactcaaggtaaatatgccaatgatcttctaaaaagagttaacatggcatcttgtaaatcggttagcacgccaatgactgttggagcaaagttacattcggctggtggtacgttacttggtcaaaatgatgcaacacggtacagaagcatagtaggagcacttcagtatttgactctcaccagaccagatatttcttttgcagtaaacaaagtatgtcaatttttacatgctcctacagatgaacattgggctgctgtgaagagaatactgcgatatataaagtcatgcaccaaacttggcatcaaaattgctaaaaccaattctttacttgtgagtgctttctcagatgcagattgggctggttgtcttgatgatagaaggtcaaccggaggatttgcagtgttcttgggaactaacttaatttcttggagtgcacggaaacaacctacagtatcaagatcaagcacggaagcagaatataaagctgtagctaatgcaacagctgaaattatgtggattcaaactttgttacgggaaattggggttcaaagccctcaacaagcaaaaatatggtgtgataatataggtgccaaatatctatccaacaatccggtctttcatggtcgaactaagcatatagagatagattatcattttgtttgagaaagggtggcaagaaaactgttgcacattgattttgtgtcatctggagatcaggtcgccgatggattcacgaagccccttgccggtaaaacaattagaaaattttaaacacaatctcaacttagctaagttgtgattgaggggggtg

>Copia28-ZM_LTR

tgttggaaaaatagagtttgtttaagtatgttgcgtaaatattggtttgtttcctgacgcacctcttcctgaggctagaagataggaatatagtttgttgaggtgataacgcttgtaacagaaatatgttgtaatctccggcttcctttgcctatattaacatgcaagctcggcagagaacaaatgctagagtttaatcaaaaggcttccgctcctctgatttctttca

>Copia29-ZM_IN

atggtaccagagccataatctaggtgttgatctagttggggaaaagttttaatcaacgaaaatgaaaagagatcgaatctgtttggatttagaaataaaacaacagaaaagaaagccaaatggcgactggatttacccctgcaaaaggaaacagaaacaaccctcacgtcaaatcaaaagaaagaaagaaaaggaagcactcacgtgcaagtgcaggggccttgccttgccgttcggctgcctgctcgccgcgtgcaggacacgggctgggtggaggccgccggattctcgccatttcggctgcctcgtctctctctgctgctggccgcatggcggctgcctcgtctctctgctggccgcgcgcaggccacgggctgaaaggagaccgccgcccgcgtagccgttgtttcctcccagcgctagacagaagatggcggcactcttggactcggcgtccatggcgtctgctgtcgagggagctggagctggagctcgaggcccgctaggccatggcggcgtgcgcaaagttagggccgagggcgaggcctgcggtttcggagccagccgccagccgttaccgtgaccgagaaagacgctaacagaaagaagtgtggcggctggaagagggaaaagaaagacactacgtatatgtactagtgcaaaccctaaactggacgggccggtttcagcccaactagaagaaagaaacagtaaacaatttttttttcagaggccattatttaattattgatgttctatctctattcaaatttgaaccaacgagataatttgattagagaatagaaaagtatttgttttatgcttctgaataaaagttttattaatgtttccgctgcgtattcaaagttattattttcattattaaattcgaaccaacgggagaatttaattttgaaaaatggatatgattttcatattattatagtattgttattattctgaccaacgttgattaatagcaatattataatattatagtgttttttgttttacattaattctatttctgcccaacggtgatgtagaattgatataaactggataaattgtatgctttaatttttgttggaactgaagcatgcaattaatgtaatttgttctcacttacatgaattgtaatttcaggaggatactccttgatggggtttattaaagatattccacccctaaaaggagataattatacagaatggaagaaaaagattgacctagccttcgttttggctgaggtggactgggtagtcaccacaccgtgtcccactgagcctgtggcaccagtgagagagacagatgaggctgagtctgcttggcagaacagacagagggaccatgctcccaaacagatggcttatgaccttgaacatagaaagtgggttattgccaacaaaaagtgcatggcagtgctaaagaatacagttgagccttctattgcgggctcaattccagagtgtgacactgtcacagaatacctcgacagaataaagggtcagttctatggctcttcaaagacatatgcaactcagctgataaagcagatgatgacagagaggtattctggaggaggcaatggcactggcataagagagcacatactaaagatgagtcatatgaactcaaagcttaaaccaatggatctgcatgttaaggatgaattccttgtccatttgatttttgcttcattgccaaaagagttcgacacctttgttgtcaactacaacattcaacccgagaaatgggatcttgatcgttgtatggctatgtgtgtgcaagaagaggagagaataaaagctgccaatggtggcactctccactatgttaaagataataagaaaagaaattttaatgccaatgccaactctcccgcaaaagcaaagggaaaggctcctcagcaccatcagcctcaacagaaaaagtttatggtaaacaaagatcaatgtctccactgtaagaagacaggacattacaaaaaggactgtcctgaattcttaaaatctatcatggcaaagaatggtgagaacattattacgttcattaatgaatccctgtatgtacaatattcaaaatctacttggtggattgactcaggggcaactatccatgttgctaattctttacagggattccgttcgacgaggactatgcaaagaaaagaaagacacattaaagttgcaaatggagtgcaagcagatgttgaagctgttggcgaccttcctctagagcttgctaatggcttcatacttgtacttagagatgttctttatgttccctctttacagaggaatttgattagtgtctcatgtttggacaataatggttttgattgtcattttggagatggcaaatgtgagatatttaataataaagaaagtgttggtcttgcttttcaaaaggaagatctttatttgttatcgcttcgtgaaaatgtgaattccgtgagcgatatgaatgaaaatatatcctcatcactgaatgaaaacagaaaacgaaagagaactcatgatgaatcatcgaaattatggcactgtcgtttaggccatatttcgagagggagaatagagagactagttaagaatgatattcttcctccattagagttctcagacttagaacaatgcagagaatgcattaaaggaaagtatgcaaagaaaattaaaaagaacgccaaacgaagcacaggaattctacagataatccacacagatatatgtggtccatttcctatgaaaagtgtggatggttttgactcgttcataacattcacagatgattactctcgttttggttacatttatccaatcaaagaaagaacagaagcgttggataaatttaagatatttaaggctgaagttgaaaatcagcatagttcaaagattaaggtagtccggtctgaccgtggtggagagtactatggtcggcataccccatatggccaagttcctggaccttttgcaaggttcttacaggaaaatggtatagtagcccagtactcaacaccgggcgaacctcagcagaatggagtagctgaaaggcgtaaccgtaccctgatggatatggtgagtagtatgatgagttactctaccttgccattgagtctatggatggaggcactgaaaaccgccattcatattctcaatagagtaccaagtaagtcgggtgcccaaaacaccgtatgagttgtggacaggaaaagtaccctcactaaaccacttacgtgtgtgggggagtcctgctgagggctaaagtatttaacccaaacattgggaaactagatcccaaaacagtaagttgccatttcattggctatccagaaagatcgaaagggtttcgtttctactgtccagatagatatacaaagtttgtagaaacgagacacgctgtgttcctagaggatgaaatgatgagggggagcatggtaagtcgaaaaattgatcttgaggaaaagcgggtatatgtgcccactccaatgtttcaggaaccaatttttgaattacctgttcctgttgcacgaacagtacaagacactgtagtgacaacacctgttgttagttctcctgtggcgacaatgaatggacatgaggaacctatccttcaggatcctatagaaaatgatgccacaagtgagggggagcaacaacagccccagacagcagatgtgccaaatgtagaggccccaagaaggtctcaaagaattaaaagatcagctattcctgatgattatgaggtgtacaacaccgaaggacttcaaatggagggtgatcccacctcatttgaagaagccatgagaagtgagcactcgtcaaagtggcttaaagccatggaagatgaaatggaatcaatgaatgctaacaaagtttggggtttagagacaattcctaaaggagccaaaacagtaggctgtaaatgggtctacagaacaaaactcgactccaaagggaatattgaaagacataaagcgcgacttgtggcaaaaggctttacacaaagagaagggattgattacaatgagacattttctccagtctcatgtaaggactccttcagaattataatggcattagtggcacactatgatttagagttacatcagatggatgtaaagacggcattcctcaatggggatttggaagaaattgtttacatggcacaaccgaaaggttttgttgtgaaagaaaaagaaggcatgggatgccgcctaaagaaatcaatttatggtttaaaacaagcttcaagacagtggtacttgaagtttgatagaacaataaaggattttgggtttaaagagaatgttgaggacaactgtgtatatgcaaagtttaagaatggaaagtacatcttcctaatcctatatgtggatgatatcttacttgctagtagtgatgtcagtctactgctggagacaaagaagtttttgtcctcaaaatttgacatgaaagatcttggtgaagcgtcttttgttttgggcatagagattcaccgagatagaacaaaaggggtattgggactgtcccaaaagttatacatagaaaagattttaaagagatttaatatgcacaaatgtagtgcatcacctgcaccaatagtcaagggcgacaaatatggggattttcaatgccctaggaaccagtacgagctcgaccaaatgaaagtggttccatatgcttctgctgtcggaagcctgcagtatgctcaagtgtgtacgcgccctgacttagcttttgttaccgggttgcttggcagatttcaatgtaatccagggattgagcactggaaattggtaaagaaagtcttgcggtatttgcaaggcacgaaaggcctcatgatgacgtatagaagaacagattcactccaaatagtggggtacacagattctgattatgcgggagatgatagaaaatccacatcgggatatgtgttcactctcgcaggtggagctatttcatggaaaagctcgaaacaaaccgtcactacatcgtccacagagtatgccgagtttgtagcgtgttttgaggcttcggggcaggtgaactggttaaagaagttcatacccggtttaaaggtggtcgacagcatacataaaccactaaagttatactgcgataatgctccagcagtacagtatgctcacaacaataggtcaagtggtgctgccaaacacattgaaataaagtactacgttgtgaggataaagtccgggatcaaataataagtcttgagcataaaagaagtgaaaagatgctcgcggatccgcttacaaaaggcttaccacccaaaatgttcaaagaacatgtagccagcatgggtttaagggaaagcctatgattcctggactataaatagggcctaaagttaaagtaactgtttcagaccggagatgtgtattgtagctgtcaaatctatcgacgattgatcgtgacgatgaaacatgctctatgcacttatctgtgatggaacgagtaaagtgaaaagtgttgaagttaaagtttaaagtttaaagtgaacgatgagatcaagggggagaa

>Copia29-ZM_LTR

tgttggattgatctccaccaagatgggcccaacggacatcttgggcctttgatcgcgccctgatcgggggcgcccaaccctatatggttggtgggcccctgtcacactgcgctataaataaaagggtgggggccggcggctcggatggtgtgtttcactagagccgtactccccaccgacaaaaccctaacatcgatctgagagggtgcgcagccagtgatgggaagacaccgccgttcatcgcagtcgacaacctcgctgcatcaaccttcatcaacgaacctgatggcggacggatctacatcctccaacacgcctgcgcttcccaacgacggtaacaatctaatactgttttgttctagaaattaggaacccgaaattactcaatagaatacagattaccccgctgatctactcttagagatcctgtaaatctaaca

>Copia2_ZM_IN

atccaaaaacctaatgttaggcatttttctgtcagaggttttgctgctgtgctaaagcctgatccttttgatggtaaaaacttcttgatatggaaagctaaaatggaattgtggctaactgcaatgtcttgttttcatgccgctgagggcaagcctgccaacttacctcctgaggatgaggctaagtttaaggctgaagacaacctctttcgaggagcagtaattagcgcactggatacaaaattccagaaaagctatatcatccttcccacagggaaagagctgtgggatgcacttgttggaaagtttggagttactgacgctggtagcgagctgtatctcatggagcagctgtatgactacaagatggttgagaaccgatctgtagtggaacaggctcatgagtttcaggcactagctaaggaactcgaactttttccttgtcctttgcctgacaagtttgtggctggcggtataatcgccaagttgccaccttcttggaaggactttgctacctctctcaaacataagagacaagagttcaatgtggaagagctcattggtactcttgatgttgaggaaagggctagaacaaaggacaatggaaaaggtgttgagacctctactgctaatgtggtgcagaagagaaacttccgcaagtttaacaagaagaaaaaccagaacaaacaagagaatgcaaataaacctgttcaaacagcacagtttaaaaagaagaacaacaataacaagggaaagggaggatgctttgtctgtggcagtgatcaacattgggcaagagagtgccctgatcgcaagttcactcaagacaagaaatcagctaatgttgtaaccactgaaactgaagatggaacatctgggtatggtaattctttaccatttgttctttcagtttgtaattcacctgagtggtggatggacagtggtgcaaacattcatgtgtgtgctgatgcctctatgttcacttcctaccaggtcgggaggtctggcgccttgttaatgggaaatgggtcgcgtgctcatgttcttggtgttggtacggtcattctgaagtttacttcgggaaagacggtgccattgaagagcgtgcagcatgtgccctctatcaagaagaatctcgttagcgcttctatgctatgtcgagatggatacaaagttgttcttgagtctaataaatgtgttgtgtcgaaacatggtacttttgttggtaaaggatatgactgcggaggcttgttccgcttatcactgcatgatgtgtgtaataaactggtgaattctgttaatttttctgatgagtcagatttatggcattcacgtttttgtcatgcaagctttggctgtcttatgcggttagcaaatataaatttaattcctaaatttaacttggtcaaaaagtctaagtgccatgtgtgtgttgaatcaaaacaaccccgcaagcctcacaaggctgctgaggcgaggagtttggcacctctagaacttgttcattctgatctgtgcgagatgaatggaattttgaccaaaggtggtaaaagatactttctcacttttatagatgactccactagattttgttatgtgtatctcttaaaaacaaaagatgaagcgttcaattattttaaggcctataaagctgaagttgagaaccaacttgagaggaaaataaaacggttaaggtccgatcgaggtggagaatatttctctaatgtgttcgatgagttctgcgtggaacatggtattattcatgagaggacaccgccattctcaccacaatccaatgggattgctgaaaggaaaaaccgcactctaacagatttggtgaatgccatgttgagtacagcgggattatccaaggcatggtggggtgaggcgattttgacagcatgtcatgtcctgaatagagttccaacaaagaacaaagagatcacaccatttgaggaatgggaaaagagaagattaaatctctcatatttgcgcacttggggttgcttggctaaagtgaatgtgccaatcaacaaaaagcgtaaacttgggcctaaaactgttgattgtatattccttgggtactcttttcacagcactgggtataggttcttaattataaaatctgatgtgcctgatatgtatgttgatactatcatggaatcaagagacgcaacattttttgagaatgagtttcccatgaagaatacacctagtgatacaagtcatgagactataattccccatgagcacgaactgtcgattcctatagatcatgctgaggattctcacgtgcacatccctgaggaggatgacactatagtcactcgaaagagcaagagacagagggttgcaaaatcctttggtaatgactttatagtgtaccttgtggaagacacaccaactaccattagtgaggcatattcctctcctgatgctgacttatggaaggaagcagtaaggagtgagatggaatctattatgtctaatggaacttgggaggtcgttgaccgtccttatggttgtcaacctataggttgcaaatggatcttcaagaaaaagcttaggcctgatggtacaatcgagaggtacaaggcaaggcttgtggccaaaggatatacccaaaaggagggtgaagatttctttgatacctactcaccagtggctcgattgactacaattcgcacattaatagccgtggcagcctcttatggtcttatcattcatcagatggatgttaagacagctttcctaaatggagagttggatgaggagatctatatggatcagccagaagggttcattgcggatggtcaagagaacaaggtgtgcaggttgataaaatcattgtatggcctaaaacaagcacctaagcaatggcatgaaaagtttgataatactcttacagcagctggctttgttgtaaatgaatctgacacgtgtgtatactatcggtatggtgggggtgagtctgttatgctgtgcctttatgttgatgacatcttgatctttggatcaaatctcaatgtgattgaggaagttaaaaatcttctatcgagcaatttcgagatgaaagatttgggagaagctgatgtcattctaaacatcaagcttgttagagaagctgatggtggggtaactttgttacaatcccattatgtggaaaaggtattgagtcgctttggttttagtgactgtgatcctgctccaacaccttatgaccctagtgtgctattgagaaagaatcggagaatagcaagggatcaattgacatactcccagatcattggctcgctcatgtaccttgcaagtgcaacaaggccagacatctcttatgctgtgagtaagctaagtcggtttgtgtcgaaaccaggagatgatcactggcgtgctcttgagagagtgttgcggtatttgaaaggtactatgacatacggtattcattataccggaaacccaaaagtgctggaaggctattgtgatgccaactggatttctgatgctgatgagctttatgccacaagcggatatgtgtttctgtttggaggtggcgctgtttcctggaagtcttgcaagcagactatcttaacgaagtctacaatggaagcagaactcgcagcattagacactgctggggctgaggccgagtggcttcgtgatttcctattggacttaccggtagttgaaaaaccgataccggctatttccatgaactgtgacaaccaaacggtgattacaaaggttaacagttctaggaataacatgaagtctacaaggcatgttaagaggagattgaaatctgtcagaaagttgaaaaactccggagttataactgtggattatgtccacacatcgaataatctggcagatcaattcactaagggtctgtcacgcaatgtgatagaaagtgcatcgagggaaatgggtatgagacccatgtgagatctactctagtggtaacctgctctatgtgatcggagatcccgtgaagtagagtggagaaacaagctaggagtagattgtgaggaaagatcccttctttaactcatttctgatgcacatctttcctatctgtaaggcaggatggtttttaccttaatgtattccaagagtcttataaaggtgagatgttgtcctacagaacatcttctgaggaatacacctatatgagtcagactgctggtcacagtctatgggacttgggtaatccctaaatactcatgaaaggcactgaagtgtgacttatatgcttctaaacagcgggaatacccttttgcagcctagtatcagcaaaggatttgagtgaatcttatttcgcacaaaactgtcaattcaaggcttagtccattgttcagttgtgaatgagtgaaactcttattctagatggatgttcaacttaacagtctccatcgaaacactggtatatcaaaggattgtgattctgatacttcatcattacaaaccctagagtttggtggggat

>Copia2_ZM_LTR

tgttggatcttttatgggcttggcccatttattgaataaactctatggtgtgtaatggtggagaataccaatagtaccacattggaagtccaagggtcttttgccttgacttatatggtgggatttattccacttaacttgagaagtcaagaaatggacaagggcgtgccacacgcgcgcgcgcgcgccgccgccgccgccgccggccgggccgggcgtggcgtggcgtggcaggcaggcgggcrggcgggcgggcgggcgggcgggcgtggcgtggtgtggttgtgttaatttttagcactcactaaccgcgtacgtcagccgagtgaccctgtcccttcagctgccgagtgaccctgtctcttcgtcagccagccgagtgacccttctccttcagctgccgacttatggcgtgactcggcaagagctggccgactcatggcataactcggctagagctggccgactcttggcgtgactcggcgacctttctccttcagcttccctctgcgagaggttaaatagaggagcacccctgtcactcggtacacgcgagaaaacactttcagactcacagtccagccgccgagtgagggtatttccatctcgtgactctgcgcgcacagagaagcgagagggcaggtgcctccgaagcccttgccgttcgagaccttgcacgggggatcggcaattaggtttttggggagcgtctacgcgactgcccaaaacatcttcttcctggygacatgacaagggaagctggacaaggatctggatcatcagagcgcatgggagggtatgatcaatttatttccttactgttttgcgttctgcagattatatatgtttcatatattcatctatgttgtttcatatgtagcaatgattttatcttatctgttctgtcttattatagtaatatgatakatctgtctgttttaattttacagtcatgctgtttatgttgctatctgtttattttcagttgttccgtcagaatacatgtttatcatgtttatatgcttattatatttatatgattcatatgctttatgttctcatgatccatattgttatggatatatttgagatcatgatttctatggttaattatcttttatatgtcatcatcataatgttaatttatggaattaaaatgatacggaaaatgcctataattctaaca

>Copia3-ZM_IN

acccctgtttagggtatgggttcctattccaacatggtatcagttaggtttagtttcttctcctcttcccagcagccacagccgccgggctycccctccccagccgccgccgcctcctccagcggycttccccttccctggccgccggccctctccctccctccctcggccgtcggcctccacccttccccggcgctcctcccgccgccggagccgccggccgagcrctgcgcgcgctgcccygacgccgcgcgccgttcgcggagcgcccctgcttcccygcnccggcgccgtcagcgctgcggccgccggctgcgmggctctccctccccggatccgcgagcaccctgcacgagcgccctgcagtgccgccccccacggagccgccgcgggcccgcgcctcggcgcctcgcctgcggctcggtctcaccgcmcggtggccggcggcggcttcacctccggcttccaccgcccgtccaccgtgccgcctcctgcgctccagctgcagcytcgtcggcctccaccagcgctgtcgcagcagcagcctccgtcgccgatccggacgcctgccgccgccgtcgcctgtccggccgcctgcgcaccgacaacagccctccgtcgctcccctgcgctccggncgcctgcgcgccgccggctgcctrccctcgcccctgygcagccagccgtcgccgaccatctcccgtaggcccggccgccgcctcctgctggcgtccccgctgctcgacagccttctccttcctgctgttcctyctcgccgcgcccctgctgtcccgtgccttggacgcccgacgccgagcaatctgccgcccctgctgttgcgcgcccggttgtccagtccgcgcctccctgcgtcggcctgcaccggtcgccatgagctttacctcgcccgccgctctcgtgactgtgcgtgtcctcctccacgacggccagctcatccggggcatgttcgtgtccttcgatccagtctggaaccctattcttcgggactgcgtcgaggtccaccctcaggcgggccgtcacgtgcctggaccgcttgtcttcccacgtgctgcgatcgcctccctagcggttgagcccgtgtcatctccctcccttcctagtgcctctgtcggggcaccttctttggccctctccgctcacaccgtaccttcgccacttccacctccccgaaccaccaccgattgggtggttgactccggggcctccttccacactacccccaccactagttcgttactccactcccatccaccccacccctcacatcctacctccatcgtcgtcggcaacggttccaccctcccggtcacctcagtaggtacatcggttcttccaggaccattctgccttaacgacgtccttgttgctcccggactgactcatcccctcctctcggttcgtcgcttcactagtgacaaccagtgctctatggagttcgacccctggggcctcaccatacgccaccttcccacacatgctgtgctcgctcgctgtgacagctccggccccctctattctcttcacttgccctccactccccacaccagagtagcctcatctcctgcacttactactaccaccacatcacatgctcttgctactaccaccacctcctccgccatttggcaccgtcgcctcgggcaccctggacctgacgtcatgtcccagctttccggccactccgacatatcatatactcggggctcttctgagcgcctctgtcatgcttgtcagctcggccgtcactcccgtcttcctttttccacttccacgtccagggctgttcaggcctttgatcttgtccactgtgatctctggacatctcctgtccttagcctctccggctacaaatactacctggtcattctggatgactactcccatttcctttggactttcccccttcggctgaagtccgacacgtttaccaccctcactcacttcttcgcctgggtatccacccagttccggcgcccggtccgtgccctgcagtgcgacaatggccgcgagttcgacaacaacgcctcccgctctttcttcctcactcacggcgtccagttgcgtctctcgtgtccctacacctctgctcagaacggccgggccgagcgcatgattcgcaccaccaccaacatgatccgctgccttctctttcaggcgtctctccctgccacctactgggcagaggcccttcataccgccacgcatctcctcaaccgtcttccctcgaaggcggtgagccaccctacacctcacttcgccctgtacggcacagccccttcctacgaccacctgcgcgtgttcggctgtgcctgctaccctaacatttccgctaccgcccctaacaagctttctcctcgctccactcgctgcctcttcctcggctactcccctgaccacaaggggtatcggtgcctggacctcacctcccaccgcatcatcatctctcgtcacgtcgtcttcgacgaagatgtgtttccccttgcaggctccaccccacccaccgatcttgactccctcctcgagtccgacccggttcctcccccacccccggcgccccgtcttgcgccgttacctgctcctcgtgcagccacgacggtcctgcatgcgccacgcgcggccccgtcgctcctgcctgcgccacgcgcggccccgtcgaccccgcctgcgccacgcgcggccccgtcgaccccgcctgcgccacgcgcggccccgtcgatcctgcctgcgccacgcgcggccccgtcgatcctgcctgcgccacgcgcggccccgtcgaccccgcctgcgccacgcgcggccccgtcgaccccgcctgcgccacgcgcggccccgtcgaccccgcctgcgccacgcgcggccccgtcgaccccggctcgcttcgccaaccccacgctcgtctaccaccgccgcggccacgccactacctcggcgccccccgactcgggcccgtcgacgagcgcgacccgcttcgccgaccccgccgtcgtctatcaccgccgcgagccggccacacccgccgctctcgacgttccggcggtccgctccgagccgtccgtataccacccggtcgccatccaccgcgaccccgggcacgtccacccgatggtgactcggcgcgccgctggcgttcttcgccccgtcgaccggctgatcctggcagctgatacgtccagcactccacccgacgcttccccggtgccctcctccgttcgcactgccctcgccgacccacattggcgtcgtgctatggaggaggagtacgcggccctcttggccaaccacacctgggacctggtgccgtgtccaccaggcaccaacgtggtcaccggcaagtggctatttcgccacaagctgacctcggatggctccctcgaccgctacaaggcccgttgggtccttcggggcttcacacagcgccccggagtggactacgacgagaccttcagccccgtcgtcaagttcgccactgttcgcgccgtcctctccctcgccctctcccgcgactgggcgatccatcagctcgatgtcaagaatgccttcctccatggcactctgacggagactgtctactgcagccagcccaccggcttcgtcgacgctgaccgtccggatctggtctgccggctgaaccggtccctgtacggcctcaagcaggcgccacgggcatggtacagtcgcttcgcctcctacctggcctccatcggcttcgttgaggccaagtcggacacgtccctgttcatctaccggcgcggcgacgacaccgtctacctcctgctctacgtcgacgacattgtgctcacggcatccaccgccgaccttctacaccgcacgatcgtcgcccttcagcgggagttcgcgatgaaggacctggggcccctacaccacttcctcggcatcaccgccgagcgccggcctcagggtctcttcctccaccagcgccagtacgccatcgacatcctggagcgggctggcatgtctgactgcaagccctgctccacgcctgtcgacactcaggcgaagctctctgaggacgacgggcctccggtcgccgacgcgacgtcctaccggagcctgaccggcgcgctccagtacctcaccttctccaggcccgacatcgcatacgccgtccagcaggtgtgcctgcatatgcacactccgcgggagccccatctcaccgcgctcaagcggattctgcgctacctccgcggctccctcgactacggcctccttctccgaccatccccgacgtcggagctcgtggtctacaccgacgctgactgggctggctgtcccgacacgcgccggtccacctccggttacgccgtgttcctgggcgccaacctcgtctcttgggccgccaagcgtcagcccgtcgtctctcgctccagcgctgaggccgagtaccgtgccgtggccaacggcgtggcagaggcctcctggctgcgccagctcctccacgagctccacagtcccctccagcgcgccaccctcgtctactgcgacaacgtcagcgcggtctacctctccaccaaccccgtgcagcatcagcgcacgaagcatgtggagatcgacctgcacttcgtccgcgagcgtgtcgctgccggtgacgttcgggttctcagcgtccccaccacgctgcagttcgccgacatcttcaccaaggggttaccgtcgagtgtatttttagactttcgatccagtctcaacatctgtacaggatagagttgtgactgcggggggg

>Copia3-ZM_LTR

tgttagaatacccgtctagggtttgggtcttccccgtgtaatgaccgtcttacccctctgtaatgggcctggcccagttactcagtctattaatayaacaccca

>Copia30-ZM_IN

tctggtatcagaggcaggggaagttctacagtggttgtgtgagcccgccgtgagaatttcggaacaagaagcgtgtgatcccacgccggtctttgcagaagacgatggcggacacacgcacaggatccggcgctgcgacttcagtcatcaaggagagctccctgggatggccaatgctaactcgatccaattatgcagagtgggctatgttgatgaaaatcaattacgagtcgatggaaatctgggatgtgatagacccgggtaccaacgtccgacgctcacaggatcgacaggctatgggtgcgttgatgagatccgtacctaaggagatgtggggaactctcggtgcaaaacagacagtgaaggaagcctgggagacagtgatggtgatgaggattggagctgatcgggtgaaagaggtaaatgcacagaaactcttgaaagaatttgagaacatcgagttcaaagaaggggagagtgtagaagattttggtatgagaatcattaatctcgttaccaatataaaatcccttggggaaactgtagatgatgctcgagttgtgaaaaaattttctgagagtggttccacctcgttttaatcaagtagcagtttcgattgaaatgttctgtgatacaaagacactcactgtggaggatttagttggtcgtttacatgcagctgaagataggtttgagaataatttggatcatgtcaccgacaaagcagggaggttactattagctgaggaggattggcttgaaaaacataagcatcgttttcaggccaatacaaataaagacagtggtagtaatgcttcaagtcagtggaaggcaaaggcttcacatcgatcagatggtgggagttcaagcagctcaaacaaagggaagctcacgtcacagggaacaccaaggaggaaaggaagatgtcgcaattgtggtatttacggacactgggcacaagactgtaaaagacccaagagggagaagaaagaacagaagcaaccggaagcaaatgtggttgttgaaggtgaagagcatggagcactcctgctagctgaactagagagagttgaaagtgtgccttcacagtttgttcatctttcagaagagaaactggtgccaaaaatgtgtccagaagggatgtgggtgctggacacaggagccagtaatcacatgactggttcaaggtcagtactgacacatctcaataataatgtccgtggctctgtgagatttggggatggatctgcagtagaaatatgtggacttggatctgtagtgatacaaggtcgacagggtcaacacaaggttttaaccagagtgtattacatcccgaagttaaaaagtaatattgtcagtcttggacaattagaggaggctggttgcgacatcaggttatttgatgggtgtctaaaaatatttgacccagaccacaatctgttggtcagtgcaccccgcactgataacagactctacacagtaaagctagctgtaacacctcctgtttgtctactaatgaagctgaatgacactgcgtggaagtggcatgccaggtatggacatctcaattttagagctcttcgtgaacttggcaagagacaaatggttgtgggaatgcctgtagtagatcgagttgaacaagtatgtgatggctgcactctaggaaagcagcatcacactccttttccaaagcagtcctcttttagagctaaaaagggactggagttgtttcatgcagatctttgtggacaagtcaggccacagacactagggggaaagaattatttcttactggttgtagatgatttcagtcgtttcatgtggatagagctgttaagaaccaaagatgaagcgttattctatctaaagaagataaaagagagggctgaggttgaccaggaaggcaggttgaaatcattgagaactgatagaggaggtgaattcaattcgagccagttttctattttctgcagtgatcaagggataatgcattacactaccactccgtactcccctcagcagaatggggtggtagagaggagaaaccagtctgtggttgaaatggcaagatgtatgttgaaaagtaagggtgtccctcctagatactggggagaggcagtctctactgctgtgtatttgctgaacaggtcacctactaaaagtgtacaagggaaaacaccgtatgaggcctggcacggcaagaaacctcaagtgggacatttcagaacttttggctgtattggacatgtcaagaaaataggccccggagtagataagctggcagatagatccactaaaatggtcctgttgggctatgaaccagggacaaaaggctacagaatggttgatccaattactgaaaaattgtgcatcagtagggatgtgaagtttgaagaagatgaggcatggaactgggacagtaataacaaagatcagttgactgaagttggctcagaaaactcaaattttcagtttcagttcactgttcccagtcaggctatgttgtatccttctggtacaggtgaggagggtgagagttctgggggtggtgaagtaccctcagtgtcaccactaccacaatcacctgtgacccctcaaaattcaagaactcaacctatactttcaggatcacaagcacaaaatccaggctcagtttcagttactagttacaattcacagccatctagttcagaaagttcaggaatggttcctgcgaaatataggacattgactgacctgtttgaaaatactgaagaaatactcgattatgagtacagtgctgtatgtatgttagcagtggatgaacctgtggatgttgaaacagctctgaagagtctgaagtggagagaagctatgcaatcagaactcaaatcaatagaagccaacaacacttggttttggtctgatctgcctagaggtcacaaagccataggtttgaagtgggttttcaaacttaagaaagatgctgcaggtaatgttgtcaaacacaaagcgagacttgttgccaaagggtatgcacagaaagaaggtgtggactatgatgaagtttttgcaccagtggctagattagagaccgtcagagttctgttggcactggcagcacaaggaaactggcaggtacatcatatggatgtaaagtcagctttcttaaatggcaacctgcaagaagaggtgtatgtattgcaaccaccaggttttgaagacctaaaaggaattagaaaagttctgagattgaagaaggctttatatggcttgaaacaagctccaagagcttggaatgccagacttgactctgagttaactcttctgggttttgagaaatgtaaggtggaacatgctgtgtacaggaaagggactggtcattctttgttggtggtaggagtttatgttgatgatcttattatctgtgggccctctgtgacagaaatcacaattttcaagcagcagatgaagcatactttcagcatgagtgacttaggcttgctcagttactatttaggtatggaggtcaggcaattggagagtgagattacaatttgccagagctcttatgcagcaaagatcgttgatcagtgtaatatgacaggatgtaatcctacagacactcccatggaacaacgaacaaagctcacatccggtgcaggaggaactgaagttgatcaaacaagatatagaagtatcattggcagtctaaggtatttggtgaatacaagacctgatttgtctttctcagttggacttgttagcaggttcatggagactccaaacaaagagcactggaatgctgtgaaaaggattgtgaggtatatagctggaacaatcaattttggagtaaaattcaaaaaaggaggaggagggggtctgtctttactaggatacactgacagtgactgttcgggtgatctagttcacaggaaaagtacctcaggtattctattctttttgggcatcaatccagtaacctggtcatctcaaaaacaaagagtggttgctctatcatcttgtgaagcagaatacatttcagcagcattgggtgtgtgtcagggggtctggctaagtatgctgttagccgatattacaaaagaagaggtgcagaagtttagtctactcatcgataatatgtcagcaatagaactaagcaagaatccagtgcaccatgataggagcaaacacattgacactcgctaccattacatccatgattgcattgaacaaggcatggtagatgtagaccatgtgggaactgatcatcagcttgcagatatactgaccaagccattgggcagaatcaagttcgtggagctcaggacaaagttgggagtggctccggttcaactagattagggggtgatt

>Copia30-ZM_LTR

tgttagtttttccataaacatttgctttctgtaatctagttgattgttgaggcgtagaatcgcgagcatgtcagtgcggtgcctgactgcgtcgtgaccgtctggttagatatcccgtcgcctgaacatgctgcacctgtccactgtgttccttaaaagggggagaacgtggggcatggactagtggcggcgggagatccgggcattaggccatgtaattgctttttactttaaataacagaaaagcaaaaggttgcgctgcggcagttgcgcagacccatctcattttccctctgtgcttgtgttctcatacctggggttttctctggcggcgctcgctccaccacggcgaccgcgcccagaagcccgactcgccgcccaacaccgtcgtctgcaaagccagctgccgagtccaggatccaaca

>Copia31-ZM_IN

acttggtaatcagagcctaggtcattttttcccacccaccctcgctatggcttccgactccatttcatccggtgctgtcacctccaacaacacctccaccatctccatgccctccatcagccatgccatcaccgttcgcctaactcgcgataattttttcctgtggaaagcccaggttacacctgttcttagtgcccatcgtctctttggctacgtcgatggctccattccagcgccgcccatggaatgcatcgagggcaccggtgctgcggcgcgtcagattgcgaatccagcctttcttacctggtatactcaggatcaacttgttcttagttctctggttgcatccatgagtgaagatatgttgggtcagatgactcaacacaccagttctgctgccgtctggactgcattgcacgccatgttctcatcacagaaccgtgctcaacttatgcaagtccgttatcaactctccaacgccaagaaggctgacatgactgcagcggcatacttccagaagatgaaaagctacaccgacacgatggcttcacttggtcatcccctgtccgatgaagaggtcctgggttacatgcttgccggcctcggttcagactttgaacccctcgtcacctccattgccacccgtgatgatcctgtaagtctcaacagtttttttgctcatcttctgagtgccgaagttcgtcttcagcgcaacacttcggttggcgagattcagtcatctgccaacgctgcttctcgtcaccacaatgatgctcgtagtggtggtcgcggtggtcgtggccgcagtgccgcgggacgcagcggtggacgtggtcgtggtggaaacaagccaacttgtcaggtttgtctcaagtatgggcatgatgctcttcactgccgccagcgcttcaatcactcattccaacctgaagacctgcgtgaacgcactggtaatgtcgcgaacactgggtcatacaacgtcgacaccaactggtacatcgatagcggcgccaatgatcatctaaccagtgacttggatcgcttgactgtgcacgatcgctacactggaaaggactccgtgcaggttgccaacggtgcaggactgtcaatctcccatattggtcatactatgcttcctggcttatctaaacctatacatcttcgaaatgttttacatgtgcctaatcttcataaaaatcttctctctgttcaaaaattagcccttgataataatgcgtttattgagtttcatcctcatttctttcttattaaggatcaagcaaccaggaaaactctactccgcggtaggagtcacaacggcctttatccagtgccaatttcctcttcgccaccagatcatgctgccttgtccagtgtcactgctcgtgctgcttctgttcatctatggcattatcggcttgggcatccgtcctctagtattgttcagtctgtcattagctcaaataaattggcttccacgcctcgtccattgtcactagtttgtgactcatgtcaacgagctaagattcatcagttgtcattcaataaatctactcatgtcacttatgctcctcttcagcttgtgcacactgatgtttggggtccagctgttacctctgtaggaggatttcgatactatgtcagttttgttgatgatttcagccgtttcacatggttatatctattgaaacaaaagtccgatgttcagcatgctttttctttatttcaaacccatgtagaacgtcttcttgatacaaaaatccaaactgtccagtcagattgggggggagaatatcgccccctttctcgctatctcagaagtcaaggtattcatcatcgtattacctgtcctcacacgtcccagcaaaacggcattgccgagcgcaaacataggcacctcgtcgaaaccggcattgctcttcttgcccactcctcccttcccatgcgtttttgggatgaagcctttctcactgcctgttacttaatcaatcgcatgcccactcgaacactacatcactcctctcctttggaatcacttttccataccaaacccgattatatcttccttcgtgcttttggttgtgcctgctacccaaatcttcggccctacaactcccacaaactttcttttcgctcagtacaatgtgtatttttgggttatagctctatgcacaagggttacaaatgtcttgaccgcacgtcaggacgaatttatatatctcgtgatgttgtgttcaacgaatccctgtttccttttgcttctactgaaccacccttgtctgcatctacttctgcccctacttcagatgagccactagcatataatgatcatatgcggcgatatagacttgaactgttagcatctgacagttctccaaagtgttcttctgtgcaggttttaggttgttcccaggagccgttcacaacaacaacgacatcgacttcatctgatgtttctctgataacattgcccctgccagctccgtgccttcctctgcatactcagacaactgatattactgacgtacactcgccatcagcttctgagacccaaccgctaatagaggaagtgtctgcagctatgccctctagaacagatgaagtgtctggagctatgccctccacatctacggtgcttactcgactacgcaacaacattgtcaaacccaagattccaacggatggcaccattctttatcatcactctcgtaagggtttctttgctgcacccacctcttacaagactgccttgactgatgaaaagtggtatgctgccatgaagtctgaattcagtgctcttcagcaaaacaacacatggacattggtccctaaacctccaggtcagaacatcatcagctgcaaatgggttttcaaagttaaagaaaagccagatgggtctattgacaagttcaaagcacggttggttgctcggggcttcacgcagcaatatggaattgattatttagagacttttagcccagttgtaaaaccagctactgtccgtctggtcctatcacttgctgtctcgcggggttggtacactcgtcagattgatatcagcaatgccttcttgcatggcgtccttgaagacactgcctacatgcagcaaccacctggatttcaggacatctctcggcctcatcatgtctgcaaactgcacaaagctatttatggcctcaaacaatcacctcgtgcttggtattctcggctaagcgaccggttgtgtcagctaggattccatccctcagttacagatacttcactctttgttttttcagagggatctctcactatgtttatgttggtgtatgttgacgacattgtcatagtcagttcgtgttccccggccatcaagaagctcctgactcagctcactagctcctttcctgtcaaggatcttggtcctctgaatttttttctaggtattgaagcggtttccaattccggggggatggttcttactcaacaaaagtatgctatggacattctcagacgagtacgtatggagaattgtaagcctattcaaacccctctttgtgcatcagaaaaattgtcaagcttcagtggcactcctcttggcgaccaagacacctttctttatcgcagcacagtaggggccttacagtaccttacccttactcgaccagacttgtcatttgcagtaaataaggcgtgtcaatttctttccaagccgactgatgttcactgggaagcagtgaaacgaattcttcgatttgttaaaggaactgctcatacaggactgaaaatcagaaaatctccatctaccctgctgagcattttcactgatgctgattgggcggggtgtgttgatgatcgaagatcgactggtggattcgctgtctactttggaccaaaccttatatcatggagtgcacgcaaacaaccaactgtgtctcgatcatcaactgaagctgaatacaaggccatagctaatggaactgcagaaggagtgtggattcagtcattactgaaagaactaaatgttattcagaaccggcctccagtcctatggtgtgacaacctcggtgcaacatatttgagctcgaatcctgtgttccatgcacgcactaagcacatcgaggttgatttccactttgttcgagagaaggtggctttacgcgcacttgaggttagacccattgcgtcaggagatcagattgcagacattttcaccaagccagctaccaaacaaatgctagaccgattcaaacccaatctcaatcttgtggcgacaggttgatattgaggggga

>Copia31-ZM_LTR

tgttaacaaagttgttacctctgtaacctacctggaagataggaatggttagctgaaagataggaatggttagccggatagatacgttcagttagcttcaacaccggataactattcacagttttatgtaaatccttttgtgtattctaatctatataatgagtgctggccgccctgattagcggcgagccattctgtatctca

>Copia32-ZM_IN

agaaataagatcatggccggcaattcctcctccctcacatccagctttactcccatgattactcatcccatctcagagaagcttgccaaaacaaactactcaacctggaaggctcaagttgttgccaccatgcgtggtgctcgcctagaaggtttcctcaccggcaaggctataatgcctgatgccgaactccaaagcagagatggagacaaaatcatcaatatctccaatcctgcttatgaagactgggtggccttggatcaacaagtcctaagcttcctgcttgcttccatatcaaaggaagtattaatccaagtctcctccaagacaactgctacggaggtatggcgtcatatagaaactatattttcatctcaaacacgagctcgtgctgtaaatactcggctagctctgtccaccgcccggaaaggcagcagctctgtggccgactatgtcggcaagatgaaggccctcggcgacgaaatggctgcagccgggcgacctcttgaagatgatgaactggtggagtatatcctcaccggtcttgatgaagaatacgactctgtggtgtcatctgtccttggccgaactgaaccaatctctgttagtgaattatattcacaattacttgcatttgaaacaagggtgaatctgcgtaacaatgggcaatcgtctggatactcggccaactctgcaaatcgccgtggtcggggtagtacaggacgccacaatcgcggcggaggccgaaatgggagaaacttcaactctcgtggcaataacagttcgcgacaggcaaatcaaggaccccgaaacaacaccaccactctgtctgatccatgtcaagtgtgtttcaagaaaggacatactgccaacaaatgctggtacaggtatgatgagaattttgttcctgatccaaggcatgttgctgctgctgccatgacatcctataccgtggatactaattggtacacggatacgggtgctactgatcatataaccggagatctcaataagttggccctccgggagaaatacaatggtgatgatcaaatccacacagccaatggagcaggtatgaacattaatcatactggtaaaactattattcacacccagactcgtaatcttaatttaaatcatgtcttacatgctcctcaagccaccaaaaatcttatttctgttcatcgtctcacttcagataataatgtctttcttgaatttcatcctaattatttcttgataaaggatcggagaacgaaggacactcttcttaaaggaagatgtcacaaggggttgtaccctctaccttctgcatcagcaaagcaagcctttggagcagtcaaaccgtcttttgaacaatggcatagccgcctaggtcatccagcagcacccatagttcaaaaagtaattagtagttttcatctcccatgtcaaatcaagtcaaataaagagtatgtgtgtagtgcttgccagcaagccaaaagccaccagttaccctttcctaaatcttcgagtgtctcgaaacatcctttagatctcgtgttttctgatgtttgggggcctgcacccgagtctgctggcagaaataaatattatgtaagttttattgatgattttagcaaattcacatggatttaccttctcaaattaaagtctgaagtgtttcagaaattttgtgaatttcaaaatcttgttgaaagaatttttgatcggaaaatcatcactgttcagactgattggggtggagaatatgaaaaacttcatcccttcttcaccaaaataggaatatctcatcatgtttcctgccctcacactcaccaacaaaatggatccgcagaaagaaaacatcgtcacattgttgaagtaggattatctctccttgccaaagcctccatgccacttaaatattgggatgaagcatttctcgctgcaaccttcctcatcaatcgcacaccaagcaaagttatatcctaccagacacctctagaacgcctatttcataccaaacccgagtattcatcccttagaatctttggttgtgcctgctggcccaacctccgaccctataatcaacacaagcttcaatttcgttccaaggaatgtgtctttcttgggtatagcagcctccataaaggttttaaatgccttgatatcacaactgggcgagtctatatttctcgcgatgtcatattcgatgaggaaatttttcccttcgtcaaacttcatacaaatgctggcccccgtcttcgagctgaaattcctctagtacccaccaaattatcaaagtctggggatgaatacatagttgaatcggtaattaatgatcctgccgctaacatttttcctgcagcaaataaatctccacaggatctgccgttattatctccagaaccggattttttggtggataatcagcttgggtcggtacatgcacctgttccattggataacgtggggcatgctcctattcccataggtgacgtgtggcactcaacaatccaagactcctcttcggttcaagaggtgacacgccatggggatcatggtgctgccatgccctcggccgatgacgccacgtctcctggctcaccctcacctgtcgagcatggggggcatcacaatgacacgacatcaactagtacaacagcagctagtggtgatacagcagcagctagtggtgatattgctccgtcgacagaagctgcagacgctacagctcatgctcctgcattgactgggacacgtcctaccaccagactgcaagcagggatcaggaagcctaaggtatatcatgatggcacaattcggtatggtttatttactgcagctagtgaaccacgatctctggtagatgcactaaaagataaaaaatggaagaatgcaatggataatgaatttgatgctttgatcaaaaacaaaacatggcacttagttccacctcagaaaggcaataatataatagattgtaagtgggtttacaaaatcaagagaaaagcagatgggagcctagatagatacaaggccagacttgttgcaaaaggttttaaacaacgatatggcatagattatgaagatacctttagtccagttgtcaaaccagctaccatcagaatagtcctctcgctagctatttccagagggtggagtttgagacagttggatgttcaaaatgctttcttgcatggatatttggaagaggaagtttacatgaaacaacctccagggtatgaagataaaaccaaaccccattatgtgtgcaagttggataaagcattatacggcttgaaacaagctccacgagcttggtactcccgtctaagctctaaattacaaagtctagggtttaattcctcaaaggcagatacatcgttattttacttcaatgaggaaaatgtgaatatatttgttctaatatatgtggatgacataatagtagccagctcaacacacgatgccatggcatcactgctgcggcaactcggtcaagaatttgccctcaaggacctaggcaggctaaattatttcttgggaattgaggtaaaccatgttcacaatggtattatcctgacacaagaaaaatatgcatctgacttactaaagaaagtcggcatgaacaattgtaaagccgtgtcaacacctttagtagttagtgagaagctctcggcatctaaagggactcctctgggtcaaaatgattcaactcagtatcgaagtattgtgggtgctcttcagtacttaacacttacaagaccagatattgcctttcctgttaacaaagtttgccagttcttgcactgcccaacgacagatcactggatggctgtaaaaaggattcttagatatttgaaacaatcagtgaagactggactcaagattgaaagatctagttcctctttaatcactggtttctcagatgcagattgggcaggatgtcttgatgatagaagatccaccggaggatttgctgtgttcctaggatctaatctgatttcatggagtgcacgtaagcagcccacagtgtcaaggtcaagtactgaagctgagtacaaagcagtagctaatactacagctgagattatgtggatccaaactcttcttcgtgagttaggagttcaaactccacgagcagctaaactctggtgtgataatattggagctaagtatctctcggcaaatcctgtgttccatgctcgaacaaaacatattgaagttgattatcattttgtaagagaacgtgttacgcagaaattattggagattgactttatttcttcaaaggatcaaattgcagatggttttaccaaacctcttcccgcaagacagttagaaaacttcaaacacaatctcaacctcatacggttgtaattgagagggga

>Copia32-ZM_LTR

tgttagatagattctatgattcatgtttatcttccttgtatgcttatccccttgtaactcggaacaaaccagtagagtcggccaaggaatagattagattggtttatttgttagcccttatcttggaccgaagatatctcatggtttgttacgttaatgtaaatctgaagcgcgctttgtcgctataagtaacacgcagacagccacggcaaaggtgagctgcttcaccccaatcttccgcgcctctgattttctatatggtatcggagcca

>Copia33-ZM_IN
[truncated: 1,372,592 more chars]
