# Supplementary material for: LTRtype, an Efficient Tool to Characterize Structurally Complex LTR Retrotransposons and Nested Insertions on Genomes
Source: Front Plant Sci. 2017 Apr 4;8:402. doi: 10.3389/fpls.2017.00402 (PMC5379124; doi:10.3389/fpls.2017.00402)
Supplement: Supplementary file 2 [file Data_Sheet_2.docx]

**Supplementary Library S2. The LTR retrotransposon sequence library of *A. thaliana.***

>ATCOPI1_IN

tggtatcagagctcatggaaataagatccatggcgccgtatactgtcccacctctcaatatttccaactgtgtcacagtcacactcaatcaacagaactacatcctttggaagagtcagtttgagagtttcctgtcaggacaagggttgctcgggtttgttaccggctcgatctctgctccggcgcagactcgttctgtcacacacaacaatgtcacttctgaagagccgaatccggagttctatacttggcatcaaacggatcaggttgttaaatcttggctccttggttcttttgctgaagacattctcagtgtggtggtgaactgcttcacctctcatcaggtatggctgactcttgccaatcattttaacagagtctcttcatctagattgtttgagctgcaacgaagattacaaactctagaaaagaaagataacacaatggaagtttttcttaaagatctgaaacacatttgtgatcagttagcctctgttggtagtcctgttccagagaaaatgaaaatcttttctgctttgaatggtcttggaagagagtatgagccgattaaaacaaccattgaaaactctgttgactccaatccaagtctgtctcttgatgaggttgcttctaagctcaggggctatgacgaccgtcttcaaagttatgtgactgagcctactatctctcctcatgtggctttcaatgttactcactcagattctggttattaccataacaacaacagaggtaaaggacggtcaaactctgggagtggcaagagttctttctccaccagaggtcgtggttttcatcaacaaatctcccctacttctgggtctcaagctggaaactcagggttagtgtgtcaaatctgtggaaaagctggtcaccatgcgttgaagtgttggcataggttcgataacagctatcaacatgaagacttacctatggctcttgctacaatgcgcatcacggatgtcacagaccaccatggacatgaatggattccagactcagctgcttctgctcacgttaccaacaaccgtcatgtcctgcaacagtctcagccttaccacggttctgactctatcatggttgcagatggtaacttcttgcccattactcacactggttcaggtagtattgcttcttcttcaggtaagattcctttaaaagaagtgcttgtttgcccagatattgttaaatctttgctatctgtttctaaactcacaagtgactacccttgctctgttgagtttgatgctgatagtgttcgtataaatgataaggcaaccaagaagctgctggtaatgggaagaaatcgtgatggtctctactcgttggaggaacctaaactccaagtcctctactccacgcgtcaaaactctgcaagtagtgaagtgtggcatagacgccttggccatgcaaatgctgaagtactccatcaacttgctagctcaaagtccatcatcatcattaataaagttgtgaagactgtgtgtgaagcctgtcatctaggcaagagcactaggcttccttttatgctatctacttttaatgcttctagacctcttgagaggatccattgtgatttgtggggtccctctccaacaagttctgttcaaggattcagatactatgtagtgtttattgatcattactctagattcacttggttttatcccttgaaattaaagtctgacttcttctcaacatttgtcatgttccaaaagttggtcgaaaatcaattgggacacaaaatcaagatctttcagtgtgatggtggtggtgagtttataagctctcagtttctgaagcatcttcaagatcatgggattcaacaaaacatgtcatgtccctacactcctcaacaaaatggtatggctgaaagaaaacacagacacattgttgaattgggtctgtcaatgatttttcaaagcaaactgcctctgaagtattggctagagtctttcttcactgcaaactttgtgataaacctcttgcctacttcatctcttgacaacaatgagtctccatatcaaaagttatatggtaaagctcctgaatattcagctttacgtgtctttgggtgtgcctgttatccaactctcagagattatgcctctactaagtttgatccaagatctttaaagtgtgtttttcttggatacaatgagaagtacaagggctatcgttgcttgtatcctccaacgggtcgaatttacattagcagacatgttgtctttgatgagaatactcacccatttgagtccatttacagccatctccatcctcaagacaaaactcctcttcttgaagcttggtttaaaagttttcatcatgtgactccaactcagcctgatcaatcaaggtaccctgtctcttctattcctcagcctgaaactacagacttgagtgcagctccagcaagtgttgcagcagaaactgcaggtcctaatgcttccgatgatactagtcaagataatgaaactatctcagtggtgagtggcagtcctgagcgtacgacaggcttagattctgcttctattggcgacagctaccattcaccaactgcagacagttctcatccttcacctgcgaggtcttccccagcaagttctcctcaaggctcaccgattcaaatggctcctgcacaacaagtccaagctccagttacaaatgaacatgctatggttacaagaggcaaagaaggaatctctaaaccaaataagagatatgttttgctcacccataaagtttcaattcctgagcctaagacagtgactgaagctctaaaacatcctggttggaacaatgctatgcaagaggaaatgggaaattgcaaagagactgaaacatggactcttgtcccatattcaccaaacatgaatgtgcttggtagtatgtgggtttttcgaaccaaattacatgctgatggttccttggacaagttaaaagctcggcttgttgctaagggatttaaacaagaagagggaattgattacttggagacttatagtccagtagtaagaacaccaacagtaagacttatactccatgttgctacagttttaaagtgggaacttaagcaaatggatgttaagaatgcttttctccatggagacctcactgaaactgtgtacatgaggcaaccagcaggttttgttgataaaagtaaacctgatcatgtgtgtcttctccacaaatccttgtatggtctgaagcagtctcctagagcttggtttgatagattcagcaattttctcttggagtttggattcatctgcagcttatttgatccttcattatttgtttactcaagcaacaatgatgtgatcttactgcttctttatgtagatgatatggtcatcacaggaaataactctcagtccttaactcatctcttagctgctctaaacaaggagttccgcatgaaagatatgggacaagttcattatttcctgggaattcaaattcagacttatgatggtggcttgtttatgtcacaacagaagtatgcagaggatcttctcatcactgcttcaatggcgaattgttcccctatgcctactcccttgccactgcagcttgatcgagtttcaaaccaagatgaagtcttctctgatcccacttattttcgcagccttgcaggtaaacttcaatatcttaccttaacaagacctgacatacaatttgctgttaactttgtgtgtcagaaaatgcatcaaccatcagtgtctgacttcaatcttctcaagagaatcctgagatacatcaaaggcacagtctcaatgggaattcaatacaacagcaattcttcttcggttgtgagtgcctatgaaagtgattatgacttgagtgcctacagtgacagtgattatgcgaactgtaaggaaactcgtcgttctgtgggtggatactgcacgttcatgggacaaaacattatttcctggtcctcgaagaaacagcctacagtgtctcgcagctccactgaggcagagtatcgatcgttgtctgaaactgcctctgaaatcaaatggatgagctctattctgcgtgaaatcggtgtctctctgcctgatacacctgagttattttgtgacaacctctctgctgtgtacctcaccgcaaatcctgcttttcacgctcgtacaaagcacttcgatgttgatcatcattacatccgcgaacgtgttgccttgaagacgcttgtggtcaaacacattccaggtcacctccagttggctgatatcttcacgaagtcgctgccctttgaagcttttactcgcttaaggttcaaactcggtgtagacttccctcccacaccaagtttgcgggggtg

>ATCOPI1_LTR

tatcagcactaacaacagaaacgacatcacttctggttttggatctctgaagaaactgccattgggccgtgagcccaccaagaaatctcctttgggccacgaaatgtcaaagccgaagcccatgtcgcagcaacggtcttcttctccaaatacagacaaaaccagagctggcaccatgacgtcttcgagctgtacgaactctgcttctcccataaaactcagcaatcgattctcagcccttgattcaagctgatgttcatctatccgttcaatcttagggtttctttatgtttgtccctttgtataaatagtaagcttaagcattgtaaaacatttaagccgtaatgcaaatcaaagcttcaactttcgtttctatgttttgattttgccttca

>ATCOPIA10_IN

gattggtatcagagcccaggctttgggtgtttcgttcacatcgcatcggtcgatggcaacgacgacgagagtggagatcaaagccttcgacggcgataacaatttctcgttatggaagatcaggataatggcacagctcggagttcttggtttgaaaggaactctaactgactttgctttgacaaagactgagacattaacaaagagtgaggagaagcaagtagcttctggagatgaatcatcggattcaagtgctgtgttgactaaagaagttccagatccgatcaagattgaaaaatcagaacaagcgatgaacattatcatcaatcacataagtgacacagtcttgagaaaagtaaatcactgtaagactgcagctacattgtgggaattgttgaatgaattgtatatggagacgttgttacctaaccggatctatgcacaattgaaattctactcgtttagaatgatgacttcaaagacgattgatcaaaacgttgatgattttctgaggatagttgcagaattaggaagtcttgatatcaaggttgcagaagaggttcaagcgatcctaatcttgaattctttgcctgttacctatgatcagttgaagcacaccttgaagtatggaaataagaccttgtctgtgaaagatgtagtatcttcttcaaagtctcttgaaagagaaatggctgagcttaaagaaaacactaaggtggtgaatacaactctatacactgcagaaagaggcagaccacaaacccgaaatcaaaatggtagtcaaggcaacaatcaaggtaataaccaaggtaaaaatcaaggaaaaggcaaaagcaggtcgaattccaaatctcgtgtaacctgctggttctgcaagaaagagggacacgtaaagaaggattgttttgctaggaagaagaagttcgaaaatgaagaacaaggtgaggcaggtgtgattactgagaagttggtgtactcagaggcacttagcatgcatgaccaagaagctaaagagaagtgggttattgactctggatgtacctaccatataacttcaagaatggactggttcacagatttcaatgaaaatgagtcaacactaatcttgttgggtgatgatcacactgttgaatcaagaggttctggcatagttaagatcaacactcatggtggaaccataagaatgttaaagaatgtcagattcgttctaaatctgagaaggaacttgatctctacaggtactttggataagttgggctttaagcatgaaggtggagacggtaagatcaggttttacaaagaaaacaaaacagctttgcgtggaaatttggttaatggactgtatgtccttgatggtcacacagttctgaatgaaagctgtaatgctgaagggtcaacaaaaaggacaagtttgtggcattgcagacttggtcacatgagtgtgaataacatgaagattctgactgagaaaggtttgattgaaaagaaggatatcaaagagctgggtttctgtgaacactgtgttatgggaaagtcaaagaagttgagttttaatgtgggaaagcacaatactgaggatctactaggatacctacatgcagatctttggggatctccaaatgtcaccccatctctctctggtaagcagtactttctgtctataatagatgacaagactcgtaaggtgtggttgatgttcttgaaaaccaaagatgagacatttgataagttctgtgaatggaaagagttggtggagaatcaggttaacaagaagatcaaagtgttgagaacagacaacggcttagaattctgcaatctgaagtttgatgagtattgtaagaagaatggtattgagagacatcgaacttgtacctatacaccacagcaaaatggtgttgcagaaaggatgaatcgaactctcatggaaaaggtgagatgtcttcttaatgaatcaggtctagatgaaagtttttgggctgaagcagcctcaactacagcctatttggtgaaccgatcacctgcatcagtagtagaccacaacgttcctgaagaattgtggctaggcaagaaaccaggttataaacacctaagaaggtttggctcgattgcttatgttcatcaagaccaagggaaattaaagcctagagctttaaaaggcgtttttctgggttatccacaaggagttaaaggctacaaagtgtggttgttggatgaagagaaatgcgtcattagtcgaaatgttgtatttgatgaagattcagtctacaagagtctgctacctgaaagtgataaagaacagattgatgggaaactcagtaaagagactaccgttactgtgaatgacagtgttaaagaaaaaggagaaagttctgcttcaggtggagctattgaggaaatcagtgacagtagtgactcagaggttgctgctacagaagaagactcacccatacagactgtaaatctcgaaaactaccagctagctcgagacagaacccgaagggttactagaccacctactaagctgtcagactatacccattttgcttatgcgttagtaatggcagaagaacttggtgaagaagaagaacctcaatgctatcatgatgcacaaaatgataaagactgggagaaatggaatggtgggatgtctgaggaaatggattcattactgaaaaatgaaacctgggatattgttgataggccaaaggatcaacatgttattagctgcagatggctatacaagataaaaccaggaattctaggtgttgagtcaaagagatacaaggccaggctagttgcaagaggtttcactcagaagaaagggattgactatgaagaggtatttgctccagtggttaaacacatttccattagaattctaatgtctattgtagttgcagatgacatggaattagagcaaatggatgttaagacggctttactgcatggagagcttgatcaagtgctatatatggagcagcctgagggatttgaagcagatccaaacaaagatcaagtgtgtttgttgaagaagtcactctatggcctgaaacaagcacctagacaatggaacaagaagttcaatgctttcatgatggatcaaggctttactaggagcttacatgattcgtgtgtatacgtcaaagaggtcatccctgatcagtttgtgtatctactgttttatgtagacgatatgttgatagcaggaaagagtatggctgaggtcaataaggtcaaagaaggattgagtttacattttgagatgaaagatatgggtgcagcgagtaggatactgggaattgatattgaaaggaatagagaggaaggaactttgtgcttatctcagtcaaagtacttagagaaggtcattcaacgttttagaatggcagatgcaaaaggtgtgagtactcctattggtgctcatttcaagttgtcagcagtcaggaacaatgatgagagtgttgacacagaagtttgtccttactcaagtgtagtaggaagtgttatgtatgctatgatagggaataggccagatgtagcgtatgctctcggattggtgagcaggtttatgagtaacccaggtcatatgcattgggaatcagttaagtggttactaagatatcttaaaaggtcaatggacctgaagttggtttacacaaaaggaaaagacatgaagatacatggtttctgtgactcggattatgctgcagacctcgataagagaaggtccataagtggatatgttttcacggttggtgggaacactgtgagttggaagtcaagcttgcagcatgttgtagcattgtccactacagaagctgagtttatggcacttactgaagcagtaaaagaagccatttggatccgaggtctcttggatgatatgggattgaagccagaagcagcttcagtgtggtgtgactctcagtcagcgatttgtctgtcgaagaacaatgcatttcatgaaagaacaaagcatattgctgtgaatttctatttcattcgagacataatcgaagctggtgatgtagaggttgagaaaatccacacttcaaggaatcctgcagatatgcttactaaagtcatacttgtgcacaagtttgaagcagctttagatcatctaaagctcctcaagtgatacttggaattatgattgccgaaggttaagttcaagtagtgcaattcactggagaatgttgaaagattgaatcaaggtggag

>ATCOPIA10_LTR

tgttgtggatcttaattctgattcaactttaagtgaccggtttaggtgctgtggttaagaagaattgaggaccggttagatggagtgttttaaaccggattgaagaggctaaaggaaaccttcttttcacgagagagggtcttctgtttctatttcggacttagtataaaaggcctttatttcttcttcttcattattcgtgttttcatctgtaacaaacaaaaagaaaagcttgagactccattgttgagctctgcttgtactctgtaattctcattctgttctagtggattgccgaactgattcggccccagacgtagacttgatcataccgatcttgttgaactgggtaatcaaatctcttgtgttgtttctttcgttcttcaattcgtttctgttcttgagctgattcaaagtttcgattactgagttttcgaaagtttgtgaagttattgcgttgtgaatcgagcataaaacgtaaca

>ATCOPIA11_IN

tggtatcagagcaaaatatctcgatctcagtcccgtaacaaacttctccgattagatctcaccggaatctcaccggaatctcaccggagtttgttcatcttctcattgttcttcttctctcttctacacgttttcctttcgtctcctttgcgattcagtctctttggttagctgtttcgattctccataggtaaacgaaatggaaagagtgttcgtcggagtagttcaccggatcttcctgtcattgcttcaccgaagattcatgagcagtcttctggttcatcagcgagagctccaggatcctcgattcatgctgcgattcatcaaccagctcaatatagctcatcgcgtcactttgaggtatcggattctcctgataatagtcatagtccttatcatcttgttagctccgatcatcctggtttagtcctagctccagaactgttagatggaaacaactatggaacctggattatagctatgactactagcattgaagctaagaataagcttggatttgtggatggatctattccaaaacctgatgatgatgatccttattgcaaaatctggcgtcgttgtaacagtatggttaagtcttggcttctgaatagtgtatcaaaggagatctatacaagcatcttgtattttcccacagccgctgcaatctggaaagatctctatactcgatttcacaaatctagcctccctcgactttacaagctgcgtcaacagattcactctcttcgtcaaggcaatctggatttgtcatcgtatcatactcgtactcaaactctttgggaagaattgacgagtcttcaggctgttccacggactgtagaagatcttctgattgaaagagagactaatagagtcattgattttcttatgggactcaatgattgttatgatactgttaggagtcagattctcatgaagaaaactttgccatctttgtctgaggttttcaacatgattgatcaagatgaaactcaacgttctgcaaggatttctactactccaggaatgacttcatcagtgtttcctgtctctaatcagtcttcacagtctgctcttaatggagatacttatcagaaaaaggagaggccagtttgttcttactgtagtcgacctggtcatgttgaagacacttgttacaagaaacatggttatccaacttctttcaagtctaagcagaaatttgtgaagccttctatctcggctaatgctgctattggttcagaagaggttgttaacaacacaagtgtttcgactggtgatttgacgacttctcaaattcaacaacttgtgtcctttctgagttccaaacttcagcctcctagtactcccgttcaacctgaagtacactctatctcagtatcttccgatccttcttcttcttccacagtgtgcccaatttccggtaacctttttccctctatcctttgttcttttgccggaattgctaggccttatgtctgttctttagatagcaatgtcactgctattcattcttgggtcatagatacaggagcaaccaatcatatttgccatgataaacattttttttcttcctttaaatatcttcatgatactactgtttccttgcctaatggaattcgtgtgagcattgttggtataggttcagttcacttgggtagacatctcatccttaatgatgttcttttcattccccagttcaaattcaatcttcttagtgtcagttctttaactaagagtatggggtgtagaatctggtttgatgaaacttcctgtgttcttcaggatgctacacgggaattaatggttggaatgggtaaacaagtagctaatctgtacattgtggatcttgattcattatctcatccaggtacagattcttctataactgttgcgtctgttactagtcatgatttgtggcataaaaggctaggtcatccttcagttcaaaagttacaacccatgtcctctttgttatcttttccaaaacagaaaaataatacagattttcattgtcgggtctgtcatatatcaaaacaaaaacatctcccttttgtttctcataataataagagtagcagaccttttgatttaatccatatagacacttggggtcccttttctgttcaaactcatgatgggtatagatatttcttaaccattgtagatgattattctagagctacatgggtatatctcttaagaaataagtctgatgtccttactgttatccccacctttgtcactatggtagaaaatcaatttgaaaccactattaaaggggttaggtctgacaatgctcccgaattaaatttcactcaattctatcattctaaaggcattgtgccatatcattcttgtcctgaaacaccccagcaaaactctgtggttgaaagaaaacatcagcatattctcaatgttgctagatcccttttctttcaatcacacattcctatttcttattggggagactgtattcttactgcagtgtatctcattaatcgtcttcctgctcctattctagaggacaaatgtccttttgaggtgttaactaagactgttccaacttatgatcatattaaagtttttggatgtctatgttatgcttccacctctcccaaagacagacataaattttctcctagagctaaagcttgtgctttcattggttatccatctggatttaaaggatacaaactcttggatctagagacacattctataattgtttctagacatgttgtgttccatgaagagctctttccctttcttggatcagatctttctcaggaagagcaaaatttctttcctgatctgaatcctacacctcctatgcaacgtcaatcatctgatcatgttaatccttctgattcatcatcgtctgtagagattttgccttctgcaaatcctactaataatgtccctgaaccttctgttcaaacatcgcataggaaggccaaaaagcctgcttatcttcaagattactactgtcattcagtagtgtcttcaactccacatgaaattcgaaaattcctctcttatgataggattaatgatccttatcttacttttctcgcttgtcttgataaaactaaagaaccctctaactatactgaggcagagaaacttcaggtttggcgtgatgctatgggtgctgaatttgatttcttagaaggtacacatacttgggaagtttgctctttgcctgcagataaaaggtgtattggttgtcgctggatctttaagatcaaatataattctgatggtagtgtggagagatacaaagctcgtttagttgctcaaggttatactcaaaaagaagggattgactacaatgagactttttctcctgttgcgaagcttaactctgtcaaacttcttcttggtgtggctgctcgttttaaattgtctcttacacaacttgatatatctaacgcttttcttaatggagatctagatgaagaaatatatatgcgacttcctcaaggctatgcatcaagacagggggactccttacctccaaatgctgtttgtcggttgaagaagtccttatatggactcaaacaagcctctcggcagtggtatctcaaattttcatctaccttacttggtctgggttttattcagtcttactgtgatcacacttgttttctgaaaatctctgatggcatctttctctgtgttctagtttatattgatgatatcatcattgctagtaacaatgatgcagcagttgatattctaaagtcacagatgaaatcattcttcaagcttcgtgatttgggtgaattgaaatattttcttggcttggagattgttagatcagataagggtattcatataagtcagcgaaaatatgctttggaccttcttgatgaaacaggtcagcttggttgtaagccatcaagcattcctatggatccaagtatggtttttgctcatgatagtggtggtgactttgttgaagttggaccttatagacggctcattggacgattgatgtaccttaacatcacacgccctgacatcacttttgctgtcaataagctggctcaattctccatggcacctcgtaaagctcatttacaagcagtttacaagattctgcaatacattaaaggtactattgggcaaggtctcttctattctgcaacttctgaacttcagcttaaggtctatgcaaatgcagattataactcctgtcgtgatagtcgtcgctctacatcaggttattgtatgtttcttggtgactctcttatttgctggaaatcacggaaacaagacgttgtttccaaatcatcagcagaagctgaatatagaagtctctctgttgcaactgatgaattggtttggcttacaaatttcttaaaggaattgcaggttccattgagtaagccaactcttctcttctgtgacaatgaagctgcgattcacatagctaacaatcatgtcttccatgagcgcacgaagcacatagaatcagattgccacagtgtcagagaacgacttcttaaaggtttgtttgaactttatcatatcaatactgaattgcaaattgcagatccgttcaccaagcctttatatccatctcacttccatcgtctaataagcaagatgggtctactcaacatcttcgtctcatcttgagggggac

>ATCOPIA11_LTR

tattaatgtatatagcttagattgttgtatataagcttagtttggttagtatagatttcatatagagtctgtacaggttggtattctataaaccaatcttaattttggtttagaggcttatggtttaggccaatgtcagtttgtatataaacccccaattgtactcttttgctaattaatgagaatgagattgttttactaata

>ATCOPIA12_IN

tggtacagagctaaagatctagcaaaaaaaaaaaaacatctaaattttttcttttttttatcgattgtttcgatctttgtttttcttgcgtgacaagaaaacatggcatcgatagatgatggttcatctacaacaaggatgacgtcttcctcgtctccggtggttacgatgggcgtttctcctttaccttatcaagttctgaaaatccaggaactacgatctcatctgtggtgttgaacggtgacaattacaacgaatggtcttcggagatgataaacgctctccaagctaaacgcaaaatagggttcatcaacggatcgattccaaaaccttcagctaatgatcctaattttgagaattggatggcagtgaattcaatgatagtgggttggattcgtacgtctatcgaacctaaggtgaaatctacggtttctttcatctccgattcacaccaactttgggaagatttgaagcaacgtttctccgtgggaaacaaagtgagaattcatcaaatcaaagcacagatcgcacgatgtcaacaagacggccaatcagttctggattattacggtcaactgtgcactctttgggaagaatatcaaacctacaaaccagtgactgtctgcaagtgtggattgtgcacatgtggtgctactcgtgaaccggcaaaagagagagaagaggagaagattcaccaatttgttttgggtctcgatgaatcacggtttggaggtctcagcacccctctcatcgccgtggatcctttgcccactcttggtgaaatttattctcgagtagtgagagaagagcaacgattggcttctgtccaggttcgtgaacaacgacaagatgcagttggttttgtggctcgcaacgatcagagttccttcccatcctcacgacccgatatgcaaaacggaggccgtttagactcctctatcattaaatctcggtctgtgacttgctcacattgtggccgtgtgggtcatgaaaagaaggactgctggcagattgttggatttccagagtggtggactgatcgcaatggaggtggtcgtggatctggttcccgtggaagaggtggtcgtggatctggtcgaggaagagaccaatctgtgacagctcatgcaacaagctctaacccgaccagccttccagaattcacaccagctcagctgcaagtctcgcacaaatgattaaaggtcaacctaacaacagcagctccgacaaattatctggtaagacaaatcttggcaatgtaattcgtgatactggagcatctcatcatatgaccggaacactatctctgttgtctaacatggtctccataccaccttgtgcggtgggttttgcggacggaagcaacacaatggctatgagtgtgggagtgttatctctttccaatgatgttgcgttggttgacgttctttatgtcccaaatttgacatgcactttaatctctgtttctaaaattgtgaaacaaataaagtgcattgcaacgtttactgatacgtattgcgttttacaggaccgtttcacgaggactttgattggaagcggtgaagagcgtggtggggtttattatcttacggatgtcgctacagcaaaaatccatacggcaaaaatctcctcagatcaaactttgtggcatcaacgtttagggcatcccagtttctctgttctttcatctttaccaaagttttctagtacttcaattcctgttggttctcgttcttgtgacgtatgctttcgagctaaacaaacaagggaggtttttccccttagtattaataaatcaagcgagtgtttctctttgattcattgtgacgtttggggtccatatcgggttccttcatcttgtggtgcagtttattttttaacgatagttgatgacttttctcgagccgtttggacctatctactactagcaaaatctgaagtgcgctatgtactcatgaattttatggcttacactgaaaaacaatttggcaagtccgtcaaaacagtgcagagtgacaatggcacataatttatgtgtcttgctgcttactttcgtgaacatgggatcattcatcagacctcttgtgttgggactcctcaacaaaatggtcgagtagaacgcaagcatagacatatccttaatgtggctagggctctattgtttcaagcaagcatgccggtcaaattttggggtgaagcggtactaagtgctgcatatttgattaaccggactccttcatctcttcacaaaggtatctcgccctatgaaattcttcacggttgtaaaccaaattatgatcagctccgtgtgtttggatcagcttgttacgttcaccgcgtcacccgtgataaagacaaatttgggaaacgaagtcgtttgtacgttttcgttggctacccttttgctcaaaaaggctggaaagtgtttgacattgacaagaatgagtttatcattactcgtgatgtcgtgtttcaggaagacgtcttcccgtatgatgctaaggatgatcattcttcacttgtacctccatctcctatagtttgcgattcagattggatgatcacactgtctctctcgaacagggggagttctgtcatctttgactccaacactgaatttgatgccggtgagaacgtgatctctatttcaaccccgacgcttccctcggtttttgaaccggaatctgaatctgcatctgtaccagtttctgaaccggattcggaatctgcatttgtaccagtttctgaaccagaatctgattctgcatctgtaccagtttctaaaccggattctgatcaaattgaaaaatcagtctctaaaccaattgtaccggttcatacgtctataccggtcgtaacagaaccttctcgtgttgctgactcaagcgctgcttctccgttgtctgtgacaaggactccagctaaaactaagctcaaatttgttcccgtttccacaccagctcgggcaaccaatctgattgttcaatcagagacaccatcgcctcctccacgtcaaggtaagcgaaaacttatacaatcagttcgtttgcaagactatgtcctctacaatactacatgttctccgataaacccccacgctctccccgattctactcaacagtcctcgtcaacagtcctaggtaattcgttggattccctaacaaattttatttctgacgatcaattctcgtcaggtcatcgtgcctatttgacagctataacgagtactgttgagcccaaacattttaaggaagctgttcatattaaagtttggaatgatgctatggttaaggaagttgactctcttgagattaacaaaacatgggatattgtcgatttaccagctggaaaagtggctattgggagtcaatgggtctacaaaacaaaatacaatgccgatggttctattgaacgttacaaggcttgtcttgtcgttcttgggaacaaacaggtagcaggcgaggactacaatgaaacttttgctcctgttgtcaaaatgacaacggtccgtactcttctttgccttgttgctgcaaatcaatgggaggtgttccaaatggatgtcaacaatgcttttcttcacggcgatcttgaagaagaggtttacatgcagctgcctccgggatttcgtcattctcaccctaacaaagtggctcgacttcgcaaatcgttatatggtctgaagcaagctcctcgttgttggttcaaaaagctttccgattcattgctcaagtttgggtttgttcagtcctatgatgattactctctcttctcctacactaaagaaaacattgagctgcgtgtgttgatttatgttgatgatcttctcatttgtgggaatgacggttacatgatacaaaaattcaaggactatttgagtcgttgcttctctatgaaagatttgggcaagctcaaatattttcttggtattgaagttagtcggggtcctgatggaatttttttgtcacaacgcaagtatgcgtctgacattgtctccgacaatgggcttcttggtactaaaccggctgcaactccgttggaacaaaatcataggcttgcaagtgatgatggtccgctccttgctgatccaaagccatatcgtcgcttagttggccgtttgatttacctcactcatacacgccctgatttgtgctattcagttcatgttttatctcagttcatgcagactcctagggaggctcattgggacgcggcacttcgtgtggttcgtttcttacaaggtacttcgggacaaggaatattgctttctgcaaactctgatcttaccctcacagttttttgtgactcggattgggcgtcttgtcctttgactaggcgttcccttagtgcatttgttgttttacttggcggttctcccatttcatggaaaaccaagaaacaagacacagtttctcactcttccgcagaagcagaatatcgtgctatggccgctgcacttcgagagattaaatggcttcgtcggttgttaaaagaattaggcatcgaacagcatgcacccactcgtcttttctgcgatagcaaggctgccattcacattgctgctaatccggtgtttcacgaacgcaccaaacacatcgaatcagattgtcactcggttcgggatgcggttcgtgatggtatcattgagacgcaacatgttcgaaccaccgaacagcttgctgacgttttaacgaaagcccttggtcgtctacattttcttgatttagtatccaagttgggtgttcggtctcctcccacgccaacgtgagggggag

>ATCOPIA12_LTR

tattgaggataagattgtatcttatgcgataagtttgaatcctagttatgatatgtttaggaatggacatttaagggtttggtgtttatcttgtgttgattccctcttgtataaatagcaatgttaagttcattaataaagcaagtcagttcgatatctaattctata

>ATCOPIA13_IN

tggtatcagagctctaaagatctaaacctataaaaattttcgtttcttattcgatagtttcgaattcgtttccgcttttctctttaaaaccttggttcttgcgtgtcaagaaatcatgtctattgcagaagaagtctcctctgcaacacatccacgtaccaatcaacaaccagatgtaaccaaagtgtctccgtacactctggccagctccgacaacccaggggccatgatttcctctgttatgttaaccggtgacaattacaacgagtggtcaacagaaatgttgaatgcacttcaagccaaacgtaaaactggttttatcaatggttcaatctcaaaaccaccattagataatccggattatgagaattggcaggcggtaaactcaatgatcgtgggttggattagagcctccatagaaccaaaagtcaaatccacggtcaccttcatatccgatgctcatcaactatggagtgaattaaaacaacgtttctctgtgggaaacaaagttcgtgttcaccaaattaaggctcagcttgctgcgtgtaggcaagatggacagcctgttattgactactatggtcgcttgtgtaagctttgggaagaatttcaaatttacaaaccaattactgtttgtaagtgtggcttgtgtacctgtggtgctactcttgaaccttccaaagaaagagaagaagagaagattcatcagtttgtgttaggactcgatgattcacgctttggagggctctctgctacacttatcgcgatggatccgtttccatctcttggagaaatctactctcgtgttgtgagagaagaacaaagactcgcatcagttcagattagagagcaacaacagtcggcgattgggtttcttactcgtcaatcagaagttacagccgatggaaggactgattcttcgattatcaagtctcgtgaccgctctgttctctgctcacattgtgggcgaagtggtcatgagaagaaagattgctggcagatagtcgggtttcctgattggtggacagagcgaaccaatggaggtggacgaggatcaagttctcgtggtcgaggaggacgtagctcgggttctaataactcaggtcggggtagaggacaagtcactgcggctcacgctacaacctccaatctctcatcctttccagaattcacaccggatcagttgcgggttattacgcagatgattcagaataaaaacaatggcacctccgataaattgtctggtaagatgaaacttggcgatgttattcttgatacgggagcatctcaccatatgactgggcagctttctctcttgactaacattgttactatcccttcgtgttctgtgggatttgcggacgatcgaaaaacgtttgctataagcatgggaacattcaagctttcagagactgtgtctttgtctaatgttctttatgtcccagcgttaaattgctcattaatatctgtttccaagctagtgaaacagatcaaatgtttggcattgttcactgatacaatttgtgttttacaggaccgattttcgaggactttgattggaaccggggaggaacgtgatggagtgtattatctaacggatgcggctacaacaacggtgcataaagtggatgtcacaacagatcatgctttgtggcaccaacgtttaggacatccaagtttttctgttctttcatctttacctttgttttcgggatcctcttgttctgttagctctcgttcttgtgacgtatgttttagagcaaaacaaacaagagaagtatttccagatagtagcaataaatcaacagattgtttttctttaattcattgtgatgtttggggaccatatcgagtgccatcttcatgtggagcagtttattttttaacaatagtggatgatttttcaaggtcagtttggacatatctactattggcaaagtcagaagttcgtagtgttcttactaattttcttgcatacacggaaaaacagtttggaaaatctgttaaaatcattcgcagtgataatggtacagaattcatgtgtctttcatcctactttaaagaacaaggcattgttcaccagacttcgtgtgtaggaactccacaacaaaatggtagagtcgagcgcaagcataggcacattctgaatgtttcaagagcactcttgtttcaagcatcactaccaatcaagttttggggagaagcagtcatgacagcagcttatttaatcaatcgtacaccgtcttctattcacaatggtctctctccctatgagttgcttcatggatgtaagccggattatgatcaattaagagtcttcggctctgcttgctatgctcatcgtgttacacgggataaagataagtttggtgagagaagccgtttatgcatttttgttggctatccatttggacaaaaagggtggaaagtatacgacttgagtactaatgaattcatagtctctcgtgacgtggtgtttagagaaaatgtgtttccttatgcaacaaatgagggtgatacgatctacacaccaccggtcacttgtccgatcacttatgatgaagattggttgccatttacgactctagaagacagggggagcgatgaaaattctttgtctgatccaccggtttgtgtcactgatgtttctgagtcagatacggagcatgatactccacaaagtcttccgactccagttgatgacccactttctccgtctacaagtgtcactccaactcagacgccaactaactcttctagttcaacatcaccgtctacaaatgtctctccaccccagcaagacaccactccgattattgaaaacactcctccgcgacaaggaaaacgacaagttcaacaacttgctcgtctgaaagattacattctatacaatgcttcttgcactccaaacactcctcacgttctctctccttctacatctcagtcctcgtcatcgatccaaggtaactcacaatatcctttaacggattatatttttgatgagtgtttttccgcaggacataaggtgtttcttgctgcaatcactgcaaatgatgagcctaaacattttaaagaagctgttaaagttaaagtctggaatgatgcaatgtataaagaagttgacgcactcgaagttaacaagacatgggacattgtcgatctgccaactggaaaagtagctattggaagtcagtgggtttacaaaacaaagtttaatgctgacggaacggttgaacgttacaaagctcgccttgtagttcaaggcaacaatcaaattgaaggggaagactacacggaaacctttgcaccagtggtaaaaatgacaacggtccgtactcttcttcgtctagtagctgctaatcaatgggaagtataccaaatggatgttcataatgccttcctccatggcgatcttgaagaggaggtgtatatgaaacttcctcccgggtttcgacactctcatcctgacaaagtttgtcgccttcgtaagtcactatacggtttaaaacaagctcctaggtgttggtttaagaaattgtctgatgctcttaaaagattcgggttcatccaaggttatgaggattactctttcttttcttattcctgtaaagggattgaacttcgcgtcttagtatatgtcgatgatttaataatctgcggtaatgatgagtatatggttcagaaatttaaggagtatttgggacgttgcttttccatgaaagacttaggcaagctcaaatattttcttggcattgaagtaagtcgtgggcctgatggtattttcttgtctcaacgaaaatatgcccttgatataatttctgatagtggcactctaggagcgagaccggcatatactccgttggaacaaaatcatcaccttgcctccgacgatggtccgttacttcaggatcccaagccatttcgtcgtctcgttggtcggctactatatcttcttcatactcgcccagagttgagctactccgttcatgtattgtcccaatttatgcaagctcctagggaagctcatttggaggctgcgatgcgcatagttcgttacttgaagggttcaccgggtcagggcattttgctgagttctaataaggatttgactctcgaggtttattgtgattcggattttcaatcttgtcccttgactcgacgttcacttagtgcgtatgtggttctgcttggtggctcgcctatttcttggaaaaccaagaaacaagacacggtttctcactcgtctgcggaagcagagtatagagcaatgtcggttgctctcaaagagatcaaatggcttaacaagttgttaaaagaattaggtatcacactcgctgcgcctactcgtttgttttgtgatagtaaggctgctatttctattgcagcaaaccccgtctttcacgagcgtacgaagcatattgaaagggattgtcactcagttcgcgatgcggttcgtgatggcattataacaacacatcatgttaggacctcggagcaactagctgatattttcactaaggctcttggtcgtaatcaattcatttatcttatgtccaagttgggtattcagaatcttcacactccaacctgagggggag

>ATCOPIA13_LTR

tattggggatagagtagaatctcttgtgatatattttgtgacagagttatgtcctataatttagggattcgagtttattttatacgatgactctcttgtataaatatcacactttgtgaatcaataaactcaagttagtgcaatacctaattctaca

>ATCOPIA14_IN

tggtatcagagcaaaacctaaactactcaagggtttgttcgattctatgatctaaacttccgctaaaacaactctgtttcttattttctgttttttaaaactatctcacaaaggagaaaatcatggccacgaattctgctacgtcgtcggctctgataacctccaccaccaatcgccgtacaatatctccatacgatttaacttccggtgagaaccccggaaccgtgatctccaagcctttgttgcgtggtccaaactacgacgaatgggccaacaacatatgcttggctttgaaagctagaaagaagttcggttttgtcgacggttctatacctcaaccaccagacgacgatcccgactttgaagactggattgctaacaacgcgcttcttgtctcttggatcaaactcacgattgaagaaaagctctcttcgtccctatctcacatcaatgactctcatgaactctggactcatattcaaaagagatttggagtgaaaaacggccagagagttcaaagattgaaaactgaattggcaaattgcagacaaagaggtcttgctatcgaagcatattacggcaaactaactcagctgtgggggagtcttgccgattaccaaaacgcaaaaacaatggaggaggttcgaaaagagcgagaagaggacaaattgcatcagtttttaatgggtctagatgataccatctacggagccgttaaatcaaatttgctttcacgtgttcctttaccaagcctagaagacgcttacaacgctttaactctggacgaggaatccaaaaatctaagtcgcactcatgaagaaagatcagatggcgtcagttttgctgttcaaaccaattcttctaggaatttttctgacaacagaggaagctctgtttcatgtaccatctgtggacgcaccgatcacattgccgacaactgcttcaagaaaataggctatctagattggtgggagcacaatccgaagtcaaattccagaaataaaaatggaaacaattcaaataagggaaattcaacttcgggtggtgttggccgtggaacaacaacctcttcggtgggctcaatgacgggcaaaagcttcgggactggtcaagttaatcatgaaaccacttcaaaccagaatcagttgcaaagttttggtgtcaacacagccttatctgcgtcaacgtcctctctcacatccgcagatcgcgtcgggataattggtttgagtaacgacgaatgggactcactagtgcaaacacttgcaaacaggaaactcaactcaaaagaaaaatcaggtatatcttttcttgagtcatggattattgatacaggagcttctaatcacatgacaggcactcttgaatttttaatcgatgttcgcgatatggctcctgtactcatcaaacttccagatggccgtttcaccatggctactaaacagggtcgagttgtttttggttctgcgctgtgtattcaagatgtgttttttgttgatgggctgcaatgtcaccttatttcagtatcacagttaactagagaacgtagttgtgtatttcagattactgatcggctatgtctcattcaggaccgcatcaccaagaccctgattggagcaggtgaacaggagaatggtctttattttttgagaggagtaggagtagctgcagccgttcattcaatggattcatcatctatggaagtatggcattcgcggttgggacatccttcttctaaagctatggatatgttaaagtttttggattttagtactaagtttgatactaaggcgtgtgaagtttgtatccgcgctaaacaaacgagagagtcttttcctttgagttataataagacaaatgttgcttttgaactgattcactgcgatctttgggtccttatcgtacatcttccatttgtggctctaaatactttctcaccatacttgacgactattcgagagcagtctggatttacttgttacattccaaacaagaagctcctacacagcttaagaattttgttgctttagttgaacgtcaattttctactaaggtgaaaacaattcgaagtgacaacggatgcgaattcatctgtctaagagattactttaatcaacacgggatcatacatgaaacatcctgcgttggcactccgcagcaaaatggccgtgttgaacgaaagcataggcatatattgaatgttgcccgaggtctacttttccaagctaatcttcccattgagttttggggttattgcgctttgacagccgggtacctcatcaatcgtactcctactgaaatacttggtggaaagactcctttcgaactcatttataatcgacctccaccaatgaatcacattcgtgtttttgggtgtttgtgctatgttcacaaccaaaaacatggaggtgataaatttgcagccaggagtacttaggcaatctttgttggttatccatttggcaaaaagggatggcgtgtttataaccttgaaaccagagcagttacggtctctcgagatgtaatctttcaagaatctgttttaccttatacaattgagctcccaaatcaaccatctctctcgtcaagcttaagtaatgaagaagaagctatcggtttgaccaacggttttgatgataatgaaccggaatcttctcactctccaaccgaaccggatcctcctcaatctccaaccgaaccagagtcttctccgcctctaactgaaccggaataacctcaacctcaaaccgatccggttgattctactacgcatacagaaccaaacccgcagacacatccggttgattcaccttcatcgtctccaaaatctatatctcgtcctgaccaatcagaagatatcaatgaagcgtatgacaattctctggccgatcatgattctgccattgatgacgtcactgataattccacttcttcgtcttcaacggatacaaacagtaactctgaaccagaacaactcggctttggattcaggaaaaagaaacctccaacaaaactcgctgactatgtcacttctctccttcataatccacatccatcatcaacgccatatccaattgacaatttcatatccagctcacaattttcagataaatatcaacattttttgttggctattacttcaggagttgaaccaaagtcttatcaagaagcaattgaagatgaaaattggagatatgcagtcaaagatgaaattgatgcgttggaagagaatggaacttggactgttgaaacccttcctgcaggtaaaaagcccattggttgcaaatgggtttttcgtctaaaattcaattctgatggtacgttagaacgacacaaagctcgtcttgttgttcttggaaataatcaaaaagaaggtatcgactactccgaaacatttgcaccagtcgctaagatgatcaccgtgagaacatttttgcaacaggctgcatctttagattgggaagttcatcaaatggatgttcacaacgcatttctacatggtgatcttgatgaggaggtttacatgaaatttcctccgggttttcgcacagatgataacactaaagtctgtcgtcttcacaagtcgctttacggtctgaaacaagctcctcggtgttggttcgcgaaacttggttcagctttgaaacaatatggttttgagcaagactattctgattattctctcttcgtcctcgcgaccggtattacaaggcttcacgtgttggtctatgtcgatgacttgattattacgggaacctcgattcatattgttaacgaattcaaacattatttgagttcttgttttcatatgaagaatttgggacctctgcgctatttcttgggcatcgaagtggctcgtagccccatagggatgtatctatgccaacgtaagtatgctttggatattagttcagaaacatgtctcatgggagtcaaaccaacaacgtttcctttggaacaaaatcacaagttgtctcttgcgcaagatggttctttttcagaccctacaaggtatagacgtcttgttggtcggcttatttacttgggcaacacacgacccgagctgtcctatgtcattcatattttgtctcagtttatgaatgatccacaaaacgctcattggaacgctgctctccgtgttgttcgctatgacaaaaacagtcctggtcagggcattctacttcgtgctaacacgcccttaacattgactgcttggtgtgactcggatcatggtgcttgtccgatcactagtcgttctcttaccggttggtttatacagtttggcgactcaccgttatcttggaaaactaaaaagcatgatgttgtctctcgttcttcggctgaagctgaatatcgcgccatggcagataccgtgagtgaaattctttggctttgtgagttattaccaactcttggcattaaggttaacacaccaatcactcttcactccgatagcttatccgccattatgcttgctgctatccggtgtttcatgctcgcaccaaacatgtcagacgtgatgttcatttcgttcgtgatgagattattcgtgtggtcatagcaaccaaacacgtttcgactacgactcaattggcagacattatgacgaaggctttgggacgtcaacaatttgaagattttcttgtcaagctgggtatttgtaacctccatactccagcttgagggggga

>ATCOPIA14_LTR

tattgggcctatgggcttagtcggttttgatatttagaataggttaactttatgctcaagttgtaacctatatataaaccaagcaatgtaacctaaaaccctaagagattcagtttatacaaacaagagtttcttgaca

>ATCOPIA15_IN

tggtatcagagccaacgagctaagtttttgttgtttccgatcagatttcttcttctccgatctcgtttttttcttgttcactactcgtttttctgtaaagatggtggctgttgctcgagtaactcggaaatccactcgttcaaaggcgtcgataagctccgttccatggaaatcgtcgcgatccactggtctcacagtctctcctccgatctctcctccagctgtagatctgctctctgctggcgcatcgcgagctgttgtgacttcagattccatggatccgactcagtctccgttttttatgcacagtggtgatcatccaggtttgaatctcgtctctatccgcttagatgagactaactatgatgattggagtgcagcgttgcggatctcgttagatgctaagaataagattggttttgttgatggttctttgcctcgtccacttgaatcggatgggaactttcgcctttggtccagatgtaatagtatggtgaaatcatggttgttgaactctgtgtctccgcagatttatcgtagcattctacgcatgaatgatgctacagatatctggcgtgacatttatggacgttttcatatgactaatcttcctcgtacgtacaatctgacacaggagatccaggattttcgccagggttctctttctctatctgagtactatactcagttgcgtattctttgggatttgttggacagtacagaggaacctgatgatccttgtacttgtggtaaagttcttcgtctgcaacagaaggcagagagagctaaaactgtcaaattcttggcaggtttgaatgagtcctactctatagtcaggagacagatcattgcaaagaaagccttgccttctctggttgaagtttacaatattcttgaccaggattacagtcagaaaggtttctctaccaatgtttctcctcctgctgcttttcaggtctctaagatatcttctactgctttgactcctaagatttgctatgttcagaatggacctaataaagggcgtccaatctgttcattctgcaacaaggtgggacatattgcagagaaatgttacaaaaagcatggttaccctcctggttttaaaggcaaactaccagagaagggtaccaaacctcaaccggttgctgctcaggtctctttattgcctccgatggttccaactcaggctactctagatggtctgcttggtaatctctccaatgatcaacttcagaacttcatagccttgttcagttctcaactgaagtcacaaccaactgcttcttcttcagatgctggaatttcacgttctcctattgattacactggtatctcattctctaactctacatactactttgttggtatattgaatgtatcccaacatactctttccactgaaacttgggtgatagactctggtgcaactcatcatgtttgccatgataagagcctatttgtttctttggatcattctgtggtgagttatgtgaatctcccaacaggatcacgtgttaaaatcagtggagtgggttcagtacagataaatgaaaacattcttcttcgaaatgttttatttctccctgagtttcgtttgaatctgatcagcatcagtagtttgacttcagatattggttcaagagtgatctttgatccgtcatgttgtgaaatacaggatcttaccaaggacctgagaattggcagaggtagacgcattggcaacctctatgtgttggatactacacctccattggattcagtctctgttaatgcggtggtggatgttagtttatggcacatgaggatgggacatccagcttactcaagactggatgctatttcagacattttgaggactactaaacacaagaataaaggatctgcttattgtcacatttgtcatttggctaagcagaagaagttgtcttttcagtcttcaaacaatatttgtaattcaacttttgagttattacatatagacctttggggacccatttcagtggagactattgatggttttaaatattttttgaccattgttgatgatcattcacgagctacctgggtttacttactcaagactaaatctgaggttcttagtgtgtttccggcttttgtcacccgtgtagagaatcagtacaacgtcaaagttaaggcagttcgatctgataatgcacaggaattgaaatttactgagttttacaaggctaaagggatagtttcttatcattcatgtcctgagacacctgaacagaactcagtggttgaaaggaaacatcaacatatactcaacgttgctagagctttgatgtttcaatccaaggttgctttaccttattggggagactgcatattgacagctgtgtttcttattaatagaacaccatctcaattgctttctaataaaactccatttgaggttcttacaggtaagaaacctgattatgagcagattcgcacctttggatgtctatgttatggttctacttctcctactcaacgtcacaagtttcagcctagagctcgggcatgcatctttctgggttaaccgtctagctacaaagggtacaagatcatggatttggaaagcaataaagtcttcatttccagaaatgttacctttcatgagacagtctttccaatggcatcaccacactctgctgactctccatcccttgtcacaccaatggattctttgccctgaggtaatcccaattccatttctccctctccatcacaaatttctccatcaacacaaatttcttcccatcgagcacgcaaacttcctgctcacttaaatgactatcattgctattctttaaacaatgaagtcactcatcccatttcttctttcatctcctactcaaaaatctctccctctcacttgttatacattaataacatcactaaaattccaattcctcaatcatattctgaggcaaaggattctaaagagtggtgtgatgctattgatacgaaaattggtgcgacggaagagaaagacacttgggaggttacaagtttacctaaaggaaagaaggcagtgggttgtaagtggatttttactctgaagtttcatgctgatgggagtcttgagcggcataaggctaggctggttgctaaggggtacactcagaaagaaggtttggattatactgacaccttctctccggttgcaaaaatggctactattaaaatgttgcttaagatttctgcatctaagaagtggtttttgactcaacttgatgtttctaatgcgtttttgaatggagatttggaggaggatatatatatgcgtttacctgaaggttatgcagacaataagggggaccaattgccaaagaatgttgtgttaagactcaagaaaacgatttatggtctcaaacaagcatctcgtcaatggttcttgaaattttctaattgtctgttggatttgggttttattaaaggacatggagatcacactttgtttgttcgctgctttggtggtgagtttcttgctgtcttggtctatgtagacgacattgttattgctagcacaatagagggtgttgcagctcagttaaccaatgctctgaaggagagtttcaagcttcgtgaccttggccctttgaagtactttttgggtctggaaattgctaggactgctgcaggcatatccatttgtcaacgtaagtatgctttggaacttcttgctttcactggaatgttggattgcaagccttctactattcccatggttccaaatctgaaactttcaaaggctgatggagaattgttggaggatcgtgagttttatcgcagtctggtggggaagctgatgtatctcactataacaaggcctgatattactttcgctgtgaacaagctttgtcaatactcagctgctccacggacttcacatctgactacagtctacaaagttttacaatatattaagggtacagttggacagggacttttctactcttctgatcctgaccttacccttaaaggctttgcagactctgattggggaacctgtcctgacactcgtcgttcaactacagggttaactatgttccttggttcttctttgatcacgtggaggtccaagaaacagcccactgtttcaagatcttctgctgaagcagagtatcgagcattagccttggcttcctgtgaaatggtatggcttgcttctttactcttagacttgaagatcatcacagggtcagtccccatagtcttctctgatagtaccgctgcaatttacatcgctaccaaccctgtttttcatgagcgaacgaagcacatcgagatcgattgccatttagttcgagaacgcttggataaaggtctcatcagaatgttgcatgttcggacagaggatcaggttgcagacatcttgactaaaccattatttccgcatcagttctcttaccttatgtccaagatgagtcttcataatatttttgcctcatcttgagggggac

>ATCOPIA15_LTR

tcttggagtatatataccatccgatttagttagaatagattattctccggtttaattgactggttaagttagaccaattgtatataaggaaattgttgtacactttactctttaagaaataagaatcgaaaatcttaacaagttttctccctagctcagctcaatccttatcttgtaata

>ATCOPIA16_IN

tggtatcagagcatttcctcacacaaatctggtgattttcaccatcgtttcttcaatttcttcttaatcacaccttttctgatcttctttcttctccgattgtctttccgcgaccttggatttgaagaaaaatggtgaagaagggaggaaaaatgaagactcgcggttctacacctgatgatgaaggttcagctgtcagatctgttcgtgaagcagcaatccttgaagatcagaagctcacgacactgaaacaacagctagctcaacgatcggacgtttatggtggaaatgggtttaaggtttctgactctggtgaaaacccactgctcctccattcctccgatcatccaggtttatctattgtagctcatattctggatggtagtaactataacagttggtcgattgcaatgagaattagcttagatgcgaaaaacaagctgggttttgttgatggttctttgttgcgaccctctgttgatgattctacttttcggatctggagtagatgcaacagtatggtgaagtcatggattcttaatgttgtgaacaaggagatttatgacagtatcttatattatgaggatggtgttgaaattggattgatttgtttactcgttttaaggtgaacaatttgcctaggaggtatcaacttgaacaagctgtgatgactcttcagcagggcaaactggatctctctacatactttacgaagaagaagaccttgtgggaacaacttgctaacacaaagtctcgcagtgtaaagaaatgtgattgtgaccaagtgaaggagctgctagaagaggcagagacaagtcgagtgatacaatttttgatgggtttgagtgatgattttaacactattcggagtcaaattttcaacatgaagccacgaccagggcttaatgaaatctacaatatgctagatcaagatgaaagtcagcgtttggttgggtttgctgcaaaatcagttccaagtccaagtccagctgcttttcaaactcaaggagttctcaatgatcagaacactattctactagctcaaggtaacttcaagaaaccaaaatgtactcattgcaaccggattggccacactgtggacaagtgctacaaagtccacgggtatcctccaggtcatccacgagccaaagagaacacttatgttggaagcacaaacttggctagtactgatcagattgagactcaggcacctccgactatgtctgccactggacatgagactatgtctaatgatcacatccaacaactgatctcatacttaagcactaagctccagtcccctagtatcacttcatgttttgacaaggctatagcttcaagttcaaatcccgtaccttctatctcacagataactggtacattcctttctttatatgactctacttattatgacatgttggactggagcttagtgcttaagtatgagatcagttgttggatgtgatcattagacatactctcatgtccagtggcagacaaggctatagcttcaagttcaaatccagtaccttctatctcacagataactggtacattcttttctttatatgactctacttattatgaaatgttaacctcttctatacctattgaaactgagttgtctcttagagcttgggttatagattcgggggctagtcatcatgttactcatgaaaggaatttataccacacttacaaagccttagatagaacctttgttagactccctaatggccatacggttaaaatagagggcactggttttattcaactcactgatgctttgtctttacacaatgttttgtttattcctgaatttaaatttaatcttttgagtgttagtgttttaactaaaactttacaatctaaagtgagttttacatctgatgaatgtatgattcaggctcttaccaaggaattgatgcttggcaaaggtagtcaagtcggcaacttatatattctgaatctggataaaagtcttgttgatgtgtcgtcttttcctggtaagtctgtatgttctagtgttaagaatgaatctgaaatgtggcataaacgtttaggtcacccttcttttgcaaagattgatactctttctgatgttttgatgttacctaaacaaaagattaataaagattcttctcattgtcatgtttgccatttatcaaaacagaaacatttaccttttaaatctgtgaatcacattcgtgaaaaagcttttgaattggttcatatagatacttggggaccgttttctgtgccaactgttgatagttataggtatttcttgacaattgttgatgattttagtcgtgctacttggatttatttacttaaacagaaatctgatgttctcactgtttttccttcattcctgaaaatggtagaaactcaatatcacactaaagtttgttcagttaggtctgataatgcacatgaattaaaatttaatgagttatttgctaaagaaggaattaaagctgatcatccatgtcctgaaactcctgaacaaaattttgtagtagagagaaagcatcagcacctgttgaatgttgctagggctcttatgtttcaatctggaattccattagaatattggggagattgtgttttaacagctgtgtttcttattaatagattactctcacctgttataaataatgaaactccttatgaaagactaactaagggaaaaccagattactctagtcttaaagcctttgggtgtttatgttattgttctacttctcctaagtctaggactaagtttgatcctagagccaaagcttgcatatttttaggctatccgatgggttataaagggtacaagctattagatatagagacatactcagtttctatttctcgacatgtcattttttatgaggatatttttccttttgcttcatctaatatcacggatgctgcaaaagacttctttcctcatatttatctccctgcaccaaacaacgatgaacatctgcctctagtgcaatcatcttccgatgctcctcacaatcatgatgagtcatcatccatgatttttgttccttctgaacctaaatcaactcgacagagaaaactgccttcccatttgcaggactttcattgctataataatactcctactactactaaaacttccccttatccattaacaaattatatttcttattcatatttatctgagccttttggagcttttattaatataatcaccgccacaaaattacctcaaaaatattctgaagctaggctggacaaagtatggaatgatgctatgggaaaggagattagtgcatttgtgagaacaggtacatggagcatatgtgatcttcctgctgggaaagttgcagtaggttgcaaatggataatcactatcaaattcttagcagatggaagtattgaacgtcataaggcgcgtcttgtagctaaaggttacactcagcaagagggcattgacttctttaatactttctctcctgtggctaaaatggttacagtcaaggttcttctttcacttgcaccaaagatgaaatggtatttacatcaacttgacatttctaatgcccttcttaatggtgaccttgaggaagagatatacatgaagctgccacctggatattcagaaatacaaggtcaggaagtctctcctaatgctgtgtgtagattacacaaatctatttatggtctcaaataagcttcccgacagtggttcatcaagtttaaaaccactttggttagtcttggatttgagaaatgtcatggtgatcatactctatttgtgaaagctcaagatggattctttttggtggttctggtttatgttgacgatatacttattgcgagtactacagaagctgcatctgctgaactaacttcgcaactgagtagttttttccagttgagagaccttggagaaccaaaattctttcttgggattgagatcgccaggaatgcagatggaatatccttatgtcaacgaaagtatgttcttgacttgttggcttcttctgatttctctgattgcaaaccctcctccatccccatggaacctaatcagaagctctctaaagatactggaactttacttgaagatggtaaacagtatcggcgaattcttggcaaattacaatatctctgcttgacaaggcctgacatcaactttgctgtctcgaagcttgcccagtattcttctgctccaactgatatacatcttcaggctcttcacaagattctgagatacttgaaaggtactattggtcagggtctcttttatggcgctgatacaaactttgacttgagaggtttctcagattcagattggcagacttgtcctgatactagaagatgtgttactggttttgctatatttgtgggtaattctctggtttcttggcgctcgaagaagcaagacgtggtctctatgagttcagctgaggctgagtatcgtgcaatgagtgttgctactaaggagctcatttggctgggatacatcctcacagcttttaagattcctttcacacaccctgcatacttatactgtgataatgaggctgctttacatattgcgaataattctgtcttccacgagcgaaccaaacatattgagaatgactgtcacaaagtcagagagtgcattgaagcaggaattctcaagacgatatttgtcaggactgataatcagctcgctgacacactaaccaaacctctatatccgaaacctttccgggaaaacaacagcaagcttggtcttctgaacatttatgaagctcaagcttgaggggggc

>ATCOPIA16_LTR

tattagattgtatatagtattccggtttgattagattggtttagctatttagagattgtacattggtttagatggtttagagtaattgtatatatactctcattgtaacacatttattcttcgaggaaatagaaaatcattctacaattctctttgttcatgatttctaata

>ATCOPIA17_IN

tggtatcagagcatatcatttgagcttccgcatatcatcggagatgagtctttcattgaatcatctttctcttatgagagtttttcgattcctacaatcgtttgttgcaatttgcaatttcttcgtcagctatctaattttggttctgattcttcttctttccttcgattgaccttttgtggtgacgaatctcactttcttttcatttcgtctcactttctttgatttttctttaacaatttgttaagaatgggttctatgaacaatccgttgaattcttcaagtcttcgtccacctccagctagtgttcctgaacaatcgcgatatcaagctgatcaatatgagaatccgcatcatttgcatacgagtgaccacgcaggtttggttctagtctcagaacgccttaatacagcttcagatttccattcttggcgaagatctatttggatggcattgaatgttaggaataagcttggattcatagatggaacgattgtgaaaccaccattagatcatcgtgattatggtgcttggtctcgttgtaatgatactgtgtctacttggttgatgaactctgtgtcaaagaaaataggccagagtctccttttcattccaactgctgaaggaatttggaagaatatgttgtctaggtttaagcaggatgatgctcctagagtgtatgatatagaacagagacttagcaagattgagcaaggttctatggatattagtgcttactatactgaattgcagacactttgggaagaacataagaattatgtggatcttccagtgtgcacttgtggtcgttgtgagtgtgatgctgcagttaaatgggaaagattacagcagcgaagtcatgttactaagttcttgatgggtttgaatgagagttatgaacaaactcgtaggcatattttgatgctaaaaccgatacgtacaattgaagaagctttcaatattgttactcaagatgagagacagaaagccatacgtcctactcctaaagttgataatgtggcgtttcagactgtttcttataacagtggtgatccttatatggagaatatggaaaatggattcattgcggcttataatacagcaagaccagctcaaattacctctgtgtactaactgtggaaaggttggtcatactgtgcagaagtgttacaaaatcattggctatcctcctggctataaggcagctacatcatacaggcaacctcaaatacaaactcagcctagaatgcagatgccacaacaatctcaaccaaggatgcaacaacctgtagcagcacaaatgcagacaaacatgccaaatacaggaccaatgcagatgatcccttatgcaaatcaaatgcagatggttccatactcgaattcaatgcagatggcgaatgctgcttatgctgaataaggttcttttgttccaatgatgccgcatgttaccagtggaggtaataatctaaacttgcaagatctttctcaagatcagatacaacatcttatttcacaattcaatgctcaagttcgggttcaagagcctgcagctacttctatttatacctctagtcctactgctactattactgaacatggcttgatggcacaaacatctacttctggtacaattccttttccttctactagcttgaaatatgaaaataataatctcacctttcagaatcatactctttcttccttacaaaatgttttatcaagtgatgcttggattattgatagtggtgcttctagtcatgtctgttcggatttaacaatgtttagggaattaatacatgtgtctggtgtaactgttacattaccaaatggtactagggttgcaattacacacacaggaaccatttgcatcacgtctactctcattctgcataatgttttacttgttcctgatttcaaatttaacttgattagtgtctgttgtcttgttaaaaccttgtcttactcggctcatttctttgctgattgttgttatatccaggaacttactcggggcttgatgattgggaggggtaaaacctacaataatctctatattcttgagacacaacgtacatccttctcaccatcactacctgcagctagttcattcactggaaccgttcaagatgattgtcttctgtggcaccagcgtctaggcatccgtcattacctgcattacagaaacttgttagttcaattccttctttaaagtctgtttcatctactgcttcgcattgtagaattagtcctttagctaagcagaaaagattagcatatgtttctcataataatctagcgtctagtccatttgatttgattcatttagacatttggggtccttttagtattgagtctgttgatggttttagatacttcttaactttggttgatgattgtacaagaaccacttgggtttatatgatgaaaaataaaagtgaagtttccaatatatttcctgtctttgttaagctcattttcactcaatataatgccaaaattaaagcaattaggtctgataatgtaaaagaactagctttcaccaaatttgttaaagaacaaggaatgatacatcaattttcttgtgcttacactcctcaacagaattctgttgttgagcgtaagcatcaacacttgttaaacatagctaggtctttattatttcagtcgaatgtacctttgcaatattggagtgattgtgttcttactgcagcttatttgattaatcgtttgccttctccattgttagacaacaaaactccatttgaacttcttttaaagaaaattccagattatactcttttgaaaagttgtctttgctatgcatcaactaatgttcatgataggaataagtttagtcctagagctagaccatgtgtgtttttaggatatccttcaggatacaaaggttataaggttttagatttagagtctcattccatttccattactcgtaatgtggtttttcatgaaacaaagtttccatttaaaacaagtaaatttttaaaagaatctgttgacatgtttcctaattccattttaccattgcctgcaccattgcattttgtagaaagcatgcctcttgatgatgatttacgtgctgatgataacaatgcttctacatctaattctgcatcatctgcatcatctattccacctttacctagtactgtgaatactcagaacacagatgcattagatatagatactaattctgtacctattgctagaccgaaaagaaatgctaaagcacctgcttatttgtctgagtatcactgtaattcggttccttttctttcttcactttcacctactacttctacttccattgaaacaccatcatcatctattccacctaaaaagatcactactccatatcctatgtccacagccatttcctatgataaacttactccactttttcattcttatatatgtgcttataatgttgaaactgagccaaaggcttttactcaagccatgaaatctgaaaagtggactagagcggctaatgaagagcttcatgctcttgagcagaataagacttggattgtggaatctttgactgagggtaaaaatgttgtaggttgcaagtgggttttcacaataaagtataaccccgatggaagtattgaacgatataaagcacggttggttgctcaaggctttactcagcaggagggaattgattatatggagactttttcacctgtggcaaagtttggcagtgtgaaattgttacttggacttgctgctgcaactggttggagcttaacgcagatggatgtttctaatgccttcttacatggagagcttgatgaggaaatctacatgagtctgccacaaggttatactccaccaactggtataagtttgccttctaaaccggtatgtcgactcttgaaatctctttatggcttaaaacaagcatctagacagtggtataagagattatcttcagtcttcttgggagcaaactttattcagtcacctgcagataacactatgtttgtgaaagtcagttgtacttcaatcattgtggtgttagtctatgttgatgacttgatgattgcaagtaatgatagctcagcagtggagaatttgaaagaattattgagatctgagttcaaaataaaggatttaggaccagctagattttttctaggattggagattgctagatcttctgagggaatttctgtctgtcaacgcaaatatgctcaaaatttgcttgaagatgtagggctttctggttgtaaaccaagctcaatccccatggatcctaacctgcacctgactaaagagatgggaaccttactgccaaatgcgacttcctatagagagcttgttggacgattgttatacttgtgcattactcgtcctgatataacttttgcagtacatactttgagtcagtttctctctgcaccaactgacattcatatgcaagctgctcacaaggttctgcgatatctcaaaggtaatcctggtcagggtttgatgtattctgcttcttctgagttgtgtttaaatggtttctcagatgctgattggggtacttgcaaagattcaaggagatctgttactggtttttgcatttatcttggtacatctttgattacatggaagagtaagaagcaatctgtggttagtcggagtagtacagaatctgaatatagaagcttagcacaagccacttgtgagattatctggttgcaacagctgctcaaagacttgcatgtgactatgacttgtccggctaaactcttttgcgacaacaaatctgcactacacttggcgacgaatccagtctttcatgagcgtaccaagcatattgaaatcgactgtcacactgtaagagaccaaatcaaggctggaaaactgaagactctacatgttccaacaggaaaccagcttgcagacattctaaccaagccacttcacccaggtcctttccatagtttactcaaacgcatgtcattatcaaatctttatctccctcttcaacagcaaggaagataagatttgaggggggtg

>ATCOPIA17_LTR

tattggtgattggtttaatctggttaactccggttatctagatactggtttaatatcatttatcttgtgtatatataaactaaactttagtttattttgttgatgaacgttgtaacaaacacttaattctgttaataaagattcagtttaatctccgatcttttctcttcttttccgttttaccggaacttcgccggtgttaata

>ATCOPIA18A_LTR

tattaagctataaagtagaataagtctcctttagcttatctattatatatatgaatgtaatgtcacatttgaggtttaatgagaaattcacaatctgttaata

>ATCOPIA18A_IN

tggtatcagagcaaaacaaactttttctcattcactttctctctcactttctcggggaagtttaacaaccaaattttccaggaaaattcatcttcttccatcaatcttcttcgtttatcttcgatttattcaattcatcaccatctaaatcgattgcacacatcatcttctccattcatcatcgtttctcagctcaaattgctgatttcttgaagtcgaaaagcaacatcaagctttcgatctttctttttcatcatcgcttcattgattcacattgaatcttccattatcatcacttcatttcattcgtttctggatttggttgtttctttactcatcttcttcaacaatgtctcaatcgacggatcaatatgacaatccattttttcttcacggctcggatcatgctgggctcgtcttagtatcggatcgattaacaaatggtgcagactttcattcatggcgacgatcagtgcgtatggctctcaatgttcgaaacaagcttggatttattgacggtacaattactaaacctactgagaatcataaagattttggatcttggtcaagatgcaatgatatggtagcaacttggttgatgaattctgttagtaaaaaattggacaaagtttattgtttatcaacactgcggaagggatatggaatagtctaatgtcaaggtttaaacaagatgatgctccaagagtgtttgaaatagagcagaggttgagtgctattcagcagggaacaatggatattagtgcatattatacggagttagtaactttgtgggaagaatataggaattttgttgagcttccattgtgcacttgtggaaagtgtgaatgtaatgcagctgctttgtgggaaaagctacaagcgagaagtcgtgtgatgaagtttttgatggggcttaatgactcgtatgaggctacacgatgacacattcttatgctcaagccgattccttctatagaagaagtgtttaacatggtaacacaagatgaaaggcagaaaaatttgaaaccatttcacaagagtgataatgtagtgtttcaagcttctgataatgctaagttctctgttgatcagaatcctgcagcttatactgagtactcatataatggtcctgcagataataatgcttgtgcagtacagaatagttataaaccaagaggtgcttctcgtcctttatgtacttactgtgggatgacaggtcatgtaattcagaagtgttacaaacctcatgggtatccacctggatatattcctggatacaagagtaatccaacatctccagggtatcaaccaaggccaccatcatctcaacaatcgtttgctcaaccatcgtttcagcaatcatttggtcaacctcctcaacctagagcgccgttctctagtcccagacctcctctacatgctgtagctaatgtcatgactggtccattcaacacacctaatgtctgtgttccaccggtggttactcaacagaacacacctgttactaatgtggacttcaatcagatgaacaatgaccagattcagactttgcttcagcagcttaatactcatgttgagatttcagaacaccaagaaccatcttcatcactttctttgattactgaacacggtgctatgcatccgtcatcttcttctggtactgtctcatttccttcaacttctcttagatatgagaatgataaattgacatttcaacatcaatgtctttcaaccctttattcaaatcttccacatggaagttggataattgacagtggagccactagtcatgtttgttcagatttgggattttttaatgaaactgtaacagtgtctggagttacagtctcattgcctaatgacactagggttgacatcacgcattgtggacgcatacatttgtctgaatcattaatattgcatgttgttttgcatgttccttcttttaagttcaatctcatatcagttagtagtctgttaaagcataatcatttgtcagctcatttttatctggatttttgctttattcaggagtctattcagggcttgatgattggtagagggattcttctgtacaatctttacattttacaacttgatgcatcatctgcagtatcttcatcacattcatctcatttctctggatccttggcggtcgatggaaagatttggcatcaacgcctaggacacccatcttctaacaagttaaaagttctttcgtgtacactttctttgtctaagagtcatgtgttagagtctcattgtgatatctgtcctttagctaaacaaaaaagattgtcttttgagtcaaataataatcagtctgttaggccatttgatttgatacatatggatgtgtggggtcctttttcagtagagtctgttgaaggatatagatactttcttactattgtagatgactgtactcgtgtaacatggatttatctcttaaggaataaaagtgcagtttcatcgaagtttcctgattttattagtcatgttagaactcaatataatgcagtcataaaagctattagaactgataatgcccctgagttagcctttcatgatttagttgcaaaacatggtatgttacatcaattctcatgtccttatacacctcaacagaactctgtagttgaaagaaaacatcaacatctgttaaatgtagcacggtcattactttttcagtctaatattccattagcttattggactgattgcattcatacagctgcttttctgataaatagaataccatctgttttgttagataacatgagtccttatgaaaagttaacacagaaaagccacagtatttgtttcttcgctcatttggttgtttgtgttatacttctacattgcaaaaagataggcataagtttagtccaagagctgataagtgtgttttcttaggatactcttcaggatttaaaggatacaagggtttacacttggatacaaatatagttttagtttctcgcaatgtagtgtttcatgaaaatgttttcccttttaaaactgataagagttacatgcaggataatgatcttttcagtgattctattctaccattaccaatagattcacacttgcatatggaaccaaactctgtagttaatccagatttaggaatacattcatctcatgatactccatcatcttcatcatcatctcataatgcatcatcatcatcatcacaacctagtgtcacaaattcttttcttgagactgtaactacagggacaaccacggtttcattgcaagatgctagaccaaaacgatcgactaaggttccaggttacttatctgactaccattgtgctcttcttcaatccactacctcaccagaagtcctcacgactaaactaaaggtcatcacgactccctatcctctttcttcttttctttcttatgcaaacataaaacctgcatatcaaaattttattctatcaatatcggttgagacagagccaaagaattttaaagaagctatagcgtctgttcagtggactaaggctatgaatgtggaactaggtgctatggaattgaataagacatggagtgtagtttctttaccaccaaataagaatgtagtggggtgcaaatgggtttttactattaagtataatgcagatggctccatagaacgttataaggctagattagtggcaaaaggcttcacacagcaggagggtgttgattactttgatactttttctcctgtggctaagttagctagtgtgaaactgattctaggattggtagctaagaagggctagagtacaccacaaatggatgttactaatgcttttttgcatagtgatttagaagaagaaatatacatgagtcttccagaaggttatacatcatcatctggagtcttcccacctaatccagtttgtaaacttcacaagtccatctatggtttgaagcaggcttcacggcagtggtacaagtgtttatcacaggttttgctggatgttggatttcagcaaacttattcagacaatacaatgtttgtgagactcacgggtacgtctttcatagctttattagtctatgtagatgatatcttgatggtgagtaatgatgatgaggcagttcagtctattaagagtgttcttgctgcaagcttcaagataaaatatatgggtcctgcaaagttttttctgggcttggagattgctcggaactctgatgggatttcaatatgtcagaggaagtattgtcttgacttgctgacaaagacaggtttattaggctgcaaaccaaagtcagtgcctatggatccaaaggtgccgatgaacaaagaaacaggtactcttctgactgatggaacaccatatagagaactaattggcaggcttctttacttgtgtgtaactaggccggatatcacctttgcggttcattgtttgagccagtttttgtcctgcccaactgatgtccatctcaatgctgctcatcatatactgaagtatctcaagaataacccagggcaaggtctattttattcggcagacacagaattgtgtttaaatggttttgttgatgcagactagggtacttgcaaggatagtagaaggtcagtaagtggtgttgcagtatttataggttcttctcttataacttggaagtctagaaagcaggatgtagctagcagcaatagcacaaaggcggaatataggagtatggctgttgctacaaaggagctactatggctgagtcaaatgcttcaagagttgcacattaaagttgacttcaaggccaagctatttgcggataacaaatttgccatgcacattgcgaataatcctgtctttcacgagaggacaaaacatgtggaaatagattgccacaatacaagggatcaagtgaaaaaaggttttctgacagttcatcatgtgactacagaaaatcagctggctgatatcttgactaaggcgttacatcctggtccgtttcattcgattcttagtcggctatcagtttcaagtctgtatcttcctcagcagcaaggttcacaagatccagcttgaggggggtg

>ATCOPIA18_IN

tggtatcagagcaaagaaaaattgttttctcattcactttctcgagaaagttctaacaaccaattttccaggaaaaattcaccaaattcatcgtcttcttcatcatcttcttccattttttctgcttttgtcttcaaatcgtcatcatcaacgacgattctcacattatcatcttcatccatcatcatttcgcagctcaatttgctgttatcttcaatcgataagcaacatcaagctttcgatctttccttcaattcatctctacatcacaatcatcgcgattcacattgaatcttcttccgctttcattcacttcatcgatctggatttggttgttacttcttgaacttactcgattctttgattctttcattgaataatggcaggctaaaacgatcatctaaagctccaagttacttatcggattaccattgttctctcatacacaccaatcctacaccaaaggtcattactactccttatcctctttcttctttcctttcatatgctcagataaagcccacctaccaaaattttgtgctttctatttcggttgagacagaaccaaaaacatttaaagaagtcatagcatctgttcagtggactatggctatgaatgtggaactaggtgttatggaacttaataagacttggagtgtagtttctttaccaccaaataagaatgtagttgggtgtaaatgggtatatactattgagtacaatgcagatggctctatagaacgatataaagcacggttggttgctaaaggtttcacacaacaggaaggagtagactactttgatactttctcccctgtggctaagcttgctagtgtgaaattagttctaggattagtagctagaaaaggatggagtacgactcaaatggatgtcacaaatgcatttttgcatagtgacctagaagaagaaatttatatgagtcttgctcaaggatacacaccatcttcaggatctttaccaccaaatccagtttgcagacttcataaatccatatatggattgaaacaagcttcacgacaatggtataagtgcttgtctcagactttacttgatgatggctttcaacaatcttatgtggataacacgttgtttgttaagatcacatccactgctattgtggctatgttgatatatgtggatgatatcttgattgtgagtaataatgatgaagttgtttgtgctgtgaagagtgttctggctgcaaggtataagatcaaggatttgggtccagcaaagttttttcttggcttggagatagcaaggaactctgatggtatttcgatatgtcagagaaagtattgtttggatttgttggctaattctggtctattaggatgcaagcctaaatcagtgcctatggatccgaaggtggttcttacgaaggatttaggtactttacttgaagatggaagaccttacagagaattgattgggaggttactgtatctttgtgttacaagaccggacattacatttgcagtccataatctcagtcaattcttatcttgcccaacgaatgttcatttgcatgcggcacatcaggttttgaaatacctaaagaacaatccaggtcaaggtttgttttcctctgcaggaacagaattgtacttaaatggctttgctgatgctgattggggaacatgcttggactccagaagatcagtaagtggtgtgtgtgtatttcttggcacttctctgattacttggaaatctaaaaagcaggaagtagccagtggaagtagcacagaagcagagtacaggagtatggcagtagcgactaaggaattgttgtggttagctcaaatgctgaaagacttacatgtggagatggagtttcaggttaagctcttttgcgataataaatcggctatgcatattgcaaataactcggtgttccatgagcgaactaaacatgttgaaatcgactgtcatactacacgagatagagtcaagaatggcttcttgaaggttcttcatgttgatacagagaaccaacttgctgatattctgactaaggcgcttcaaccaggtccatttcggtctatccttggtcgtctatcagtgtcaagcctcttccttcctcaaaaaggtcaagctatacaggattcggcttgaggggggtg

>ATCOPIA18_LTR

tattaagcttatatgagagttagttatagtttgttactttactctcatattatatatactcttgtaatgtttactttgttcattaatgagaaatatactttacatttgataaagttaata

>ATCOPIA19_IN

tggtatcagagccatggatagatccatggaaatttattcatgtccgtcgttagacatctcaaattgcgttaccgtaaagcttacagatcgcaactatttactatggaaatctcagttcgaatcgtttctctctggccaaggccttctcgggttcgtcactagcgcaatctcggttccagcaagcaccattccggttcctcacattgaaggtcacacggatacagctgcaaatccagatttccaggcgtggcaccgttcagatcaagtcgtcaagtcatggcttctgggttcgcttactgaagacattcttagtgtggttgtcggttccaaaacgtctcaagaggtatggctaaacctcgcaagtcattttaaccgtatttctgcatctcgtgtctttgagcttcagcgtcgtctccatggtttgtcaaaagaaggaaaaaccatggatgagtatttgcgttgtctaaagaatatttgcgatcagcttgcttcagtaggaagtcctgttacagaaaagatgaagatttttgcaatggttcatggtctaactcgtgaatatgagccactgattacttcacttgaaaacaccttggatgttctcctggtccatcctatactgaaatcatgtttcgtctaaaaagctatgatgataggcttcaaggatacactgcagcagatgtatcaccacacctagcatttggtacattcaacaactcctctcgaggtcgaggtaaaggatctagaggaagaggacgcggaaactactcaactagaggtcgaggttttcagcaacagtttctttctttaagttctcagtcgtccggtgaaagaccaacatgtcagatctgtggtaaaagaggacaccctgctattcaatgttggcatcgttttgatgaaagctatcagtactctgaagctgctgctgctgcattctctgctttgcatattactgatgttacggatgatggtgcttgggttcctgactctgcagcaactgctcacatcaccaacaatggtcagagactgcaacaaagtcagccctattatggtaatgacacaataatggcgagtgatggtaatttcttacccataactcacattggttctgctagtctaccatcaacgtcaggtaatctgccattaaaagatgtattagtttgtcctgatatagctaaatctctgttgtcagtatcaaagttaacaaaagattatccatgctcttttacgtttgatgctaatggtgttcttgtaaaggacaaagcaacaagcaaagttttgacaatgggaagcagcactagtgatggtctctataagttggagaatccaaagtttcagatgttctactcaaccagacaagtcaaggcatctgatgcagtgtggcatatgagattgggacatccgaatcctcaagtcctccagcttttatctttaagtaaggcgatcaaaatcaataaaagtaccaatgcggcgtgtgaatcctgtcgacttgggaagagttcaagattgccattttcttcttctgaatttattgcttcaagacctctagagagagttcactgtgatctctggggtccagctcctgtttcttctgttcaaggtttccagtactatgtaatctttattgataatctatcaagatttagttggttctatccattaaagaggaaatcagatttctgctcaatattcatcaagtttcagagttctgttgagaatcttttacaaacgaaaattggaacttttcagagtgatggaggaggagaatttattagtcaaaggtttttcaagcagttacaagattcaggcatacaacgttatatgtcatgtcctcacacacctcaacaaaatggcatggctgaaagaaaacatagacagttgacagagatgggactcactctaatgtttcaaagcaagacaccacagaaatattgggttgaagctttctttacatcaaactttctcagcaaccttctaccaacaatagctctagcaaccaagattagtccatatgaagctttgtttggaaaagttccaaactactcggctttaaggactttcggttgtgcatgctttccaacacttcgagactatgctcgcacaaagtttgatccgtgatctctgaaatgtatctttctaggatacactgataaatacaaaggctatcgatgtttctatccaccaacaggcagagtttatctgagtcgtcatgtcttgtttgctgaagaatctttcccattcgcagacacttacatcaatcagcagactgcatctccaactccattgtttgcagcttggcttcaaggtttttccactacttcgtcatcagaaaccaatacgcaagaaaatggaacaactgcttctacagagaatcatcaacaagagtccatgtcagaaaatcaaccaatttttggaaatggtttgagtgaggagaactctgaaaatatgcaactaacgattgcagatccaataagtgctataccgttacaaacagttcttcagcaagataatgaaacaacaccggagcaaaggtctagaagtgatggaaaacgtcctgagtgtacggctagcttagatcttgatcctataggcaacatcgtttctccgttaccttctagactagagcaacttgcatcaagaacaactacagctacagaatcaactcatcctatggtaacaagatccaaggcaggtattagcaaaccaaataaaaggtatgccatgcttgctcataaagtctctcatccaatgcccaaaacagtaactgaagctttaaaagatgaaaagtggactgctgcaatgaatgtgttaggttccaagtgggttttcagagtaaagcttctagctgatggatctttagacaagtataaagctagattagtagctcaaggctttaatcaagaagagggaatagattaccttgagacctacagtcctgtggttagatcagcaactgtgagagcagtcttgcatcttgccaccatcatgaattgggaactgaagcaaatggacgtaaagaacgcatatcttcacggagatcttacagaaactgtgtacatgaagcaaccagctggatttgtaaaaaaggcattaccaaatcatgtgtgcctgctacacaagtctctctatggactaaaacagtccccacgagcatggtttgacaaattcagtaacttcttactctcttttggttttgtatgcagtttttcagatccatctctctttgtctgcgtaaagaactcggatgttatcatgttgcttctcaacgttgatgatatggtcatcactggggacagctcaaagctactgtcaaatctcctcaacgaactcaacaaacaattcaagatgaaagatctaggcaaactcaactattttttgggaattcaggctcaatttcatcaaaagggtctcttcttatctgaaccaaaatatgctgaagatctccttttcacagctggcatgaaagactgtgctccagtggcaacaccattaccaattcagccagacaatgctcctcatcagtcacaactctttgataatccaacatattttcgcagtctcgcgggaaaattgcagtatctgacactcacaaggccagacattcaattctctgtgaactatgtctgtcagaagatgcacgctcctacgctatcagatttccacctactgaaacgcattttaagatatatcaaaggtaccactacaatgggaatttctttcaacaaaaacacagactgtaaattgagggcttatagtgatagtgatcatgctggatgtcatgcttcacggagatccactggaggtttctgtacctttctaggtaacaatctcatatcgtggtcatcaagaaaacaaccaacggtagcaaagagctccactgaagctgaatacagggcaatgtcagagacggcttcagaaatcacctggattgtcaatcttctcaaagatctaggagttccttagttgcagaaaccagaactattctgtgacaatctttctgcagtttgcctcactgcaaatccatcattccatccgagaacaaaacacttcgcaactcactatcattatgtgcgtgaacaagtcgcgtttagggagctcatcgtcaatcacatcccagggtatcttcaactagctgacatcttcacaaaatctcttccagcagctccgttcacaactctaagattcaaacttggcgtcgatttgccaccaactccaagtttgagggggag

>ATCOPIA19_LTR

tattagtaactcagctcaaaacgacaacacttcgcagttgggcctaaagctacagaagcccaacacgatccataacacaacaaagccctgtcgtcctcaacggttatctgcagaacaagacaaaagcagaggattagcagaacagtcctgcagtacggaggtagcgacaatgaacagatttgaaatgcttggagcgtgcgatgattaagtcaaacacatagcattcaaagacaactaagcaagacaaggagacactaggttaaagaatagaaactctataaaaccaatgtatctttgtcattgaaacttaagctaaataaaatcaaaacatcaaagtttacagttttggcttctttacttta

>ATCOPIA1_IN

tggtatcagagcaatttctgctcttacttctctgatttctcatcgatttctcattgctttcttcgtttttcttcgttttccaccgttaaagtgttgaatcgatcccggatttgtgttgttttgagctcaagctgaccagaatcaccttctgaagcttcgattcaagttccgataacacatttgagcttcgatctctctatccatggtgaaacagaagaagaaaacgcagaatcggagttcttctcctacatctgagtattctccgatgaagactcgagctggttcttcatatccagtcgccgatgacatcgttgaacctccgtctctcaaggttatggctaatcgcgatcgttttcaatcaaattccgcatcgattgaatcgtacgataatgctcatagtccgtacttccttcactcatcggatcatccaggtctgaatattgtttctcatattcttgatggaacgaattacaacaattggtcgatagctatgcgaatgagtttagatgcaaagaataagttgagctttgttgatggatctcttcctagacctgatgttagtgatcgtatgttcaagatttggagtagatgcaatagtatggttaaaacttggttgttgaatgtggtgagtaaagagatttatgatagcatcttgtattatgaggatgcagctgaaatgtggaatgatctattctcaagattcagagtgagtaatcttccgaggaagtatcaacttgaacagtctatccacactctgaaacaaggaaatctggatttatccacttactatacgaagaagaagacactttgggaacaactggctaacactagagttctcacagtgaggaagtgtaattgtgagcatgttaaggaattgctggaagaggctgagactagcagaatcattcagtttcttatgggattgaatgataattttgctcatattagagggcagattttgaacatgaaaccacgaccaggcttgactgagatatacaatatgcttgatcaagatgagagtcagcgtctagttggaaatcctactctatcaaatccgactgctgcctttcaagttcaagcttctcctattattgattctcaagtcaatatggctcaaggttcatacaagaagcctaagtgttcttactgcaacaagcttggtcacttagtagacaaatgctataagaagcatggttatccaccgggttctaaatggactaaaggtcagaccattggttctactaatcttgctagtactcagctccagccggtgaatgagacaccaaatgagaagacagattcttatgaagagttctcgactgatcaaatccagactatgatttcttatcttagtactaagcttcacattgcatctgcctcacccatgccgactacatcttctgcttcgatttctgcaagtccctccgtacctatgatctcacagatctcaggtacattcttgtcactattctccaatgcctattatgacatgcttatttcttctgtatctcaagaaccggctgtgtctcctagaggttgggtaatagattcaggagccactcaccatgttacacacaatagagacctatacttaaactttaggtccttagaaaatacctttgttagactcccaaatgattgtacagtcaagatagcaggcataggattcattcaactgtctgatgcaatctctctacataatgtgctttatattcctgaatttaaattcaatttgatcagtgttagtgttcttactaagtttttgaaaacaaaagtgagctttacatctgatgaatgttttgttcaggaacttacaaaggagttgatgattggtagaggtagtcaagttgggaatctttatgttctggatttcaatgaaaataatcatactgtgtctttaaaaggtactacttctatgtgtcctgagttttctgtttgttcttctgttgttgttgattctgttacatggcataagagattaggtcatcctgcttattctaagattgatttgttgtctgatgtgttgaatcttaaagttaaaaagataaataaagaacattccccggtttgtcatgtttgtcatgtttgtcatttgtcaaaacaaaaacatttatcttttcaatctagacaaaacatgtgttctgctgcatttgatttagttcatatagatacttggggacctttctctgtccctactaatgatggttttaaatactttctaactatagttgatgattttagtagagctacttggatttatctgttaaagaacaaatcagatgttttgcatgtttttcctgcttttattaatatggttcatactcaatatcagactaaactcaaatctgttagatcagacaatgcacatgaattgaaattcactgatttatttgctgcacatggtattgttgcatatcattcttgtcctgaaactcctgaacaaaattctgtggtagagagaaagcatcaacacatcttgaatgttgctagagccttattatttcaatccaatataccactagagttttggggtgattgtgtcttaacagcagtgttccttatcaatagattacctactcctgttttgaataataagtctccttatgagaagcttaagaacatacctcctgcttatgagtctttaaagacatttggttgtttgtgttatagttcaacttctcctaaacagcgacataaatttgaacctagagctagagcctgtgtatttctaggttatcctttaggttacaaggggtataagctattagatatagagacacatgccgtttctatttcaagacatgtcatttttcatgaggatatttttcctttcatatcatccactatcaaggatgatatcaaagacttctttcctctattacagttccctgctagaaccgatgatttgcctctagagcaaacatcaatcattgatacacatccacatcaagatgtgtcatcctccaaggctttggttcctttcgatccattgtctaaacgccaaaagaaacccccaaaacacttgcaggattttcattgctataataatactagtactatcttatatcccatcacagattatatctcttattcatatatagctgagcctttccatgcctttattaataatatcacaaatgcggttatcccacaaagatattcagaagctaaagattttaaggcatggtgtgatgcgatgaaagaggagattggtgctatggtaaggactaatacctggagtgtggtaagtcttcctcctaataagaaagctattggttgtaaatgggtcttcactatcaaacataatgcagatggtagtattgagagatacaaggcacgtttggtagcaaaagggtatactcaagaagaaggtttggattatgaagagactttctcacctgttgccaagttgacttcagtgagaatgatgttgttgcttgctgccaagatgaagtggtcagttcatcaacttgatatttctaatgcatttctgaatggagacttggatgaagagatatatatgaagattcccccaggttatgcagatcttgttggtgaagctttgcctcctcatgctatttgtcgtttacacaagtccatttatggactcaaacaagcctctcggcaatggtaccttaagctgtctaacactctaaagggaatgggttttcagaagtcaaatgcagatcatactttgtttatcaagtatgctaatggagttttgatgggtgttttggtttacgtggatgatataatgattgtgagtaacagtgatgatgcagtggcacagtttactgcagaattgaagtcttatttcaagttgagagatttgggtgcagctaagtactttcttggtattgagattgcacgttcagaaaaagggatttcaatttgtcaaaggaagtacattctggagctgttatctactacaggttttcttggtagtaaaccgtcttcgattcctttggatccaagtgttaagctgaataaagaagatggtgttcctttgactgattctacctcttatcgaaagttggttggaaagttgatgtatttgcagattacaagacctgacattgcatatgctgtcaacactttgtgtcagttttctcatgctccaacaagtgttcatttgagtgctgttcataaggttcttcgttatctcaaaggcacagttggacagggtcttttctactctgcagatgacaagtttgacttaagaggttacacggattcagactttggttcttgtactgattctaggcgttgtgtggctgcttattgtatgttcattggagattatctagtctcatggaaatcaaagaagcaagatacagtctctatgagtactgcagaagcagaatttcgagctatgtcacagggcactaaggagatgatttggctttccagactttttgatgatttcaaggtaccattcattcctccagcctatctctattgtgataatacagctgctttgcacattgtcaacaattcggtgttccatgagcgcaccaagtttgtggagcttgactgttacaagacaagggaagcagttgaatcaggttttcttaaaaccatgtttgtggagactggagaacaggtggcagatcctctaaccaaagctatccatccagctcagtttcataagctcattggcaagatgggagtgtgcaacatctttgccccactgccatcttgaggggggg

>ATCOPIA1_LTR

tattagcttatatatatttagtctatacttgtatataatagcagtgattaagggttcggtatagtcccggtttattcatctaattaaggtgaatctctggtttattattggtttagttggtttgttgtctatacaacattgtaacaaactttcaagattattaatgagaaattgagctttcaccatgttttctctctcgagcatggttcctatta

>ATCOPIA20_IN

tggtatcagagccataaagctccatatactcattctactctacatcattcttattcttcttcctcttcaataaaacgagtccttttactcttcggttaaaagctttatcctccggtccgttcagatctaaaccatggagaacactaaagctttgtttgttccggttacactcaaaggagtgaactaccttctgtgggcaagaaccacgaagacaaccttgtgcagccgaggactttgggctcacatcttaacgagtgaagcaccatccgaggcgaccataagagaaggcatggagatagttcatgtcggtgaagaaaaatggttccaagaggaccaatcagtcttagctctactccaaaactcgcttgaggcttcccttcttgaagcttactcatattgtgagaccgccaaggagctatgggaaactctcttcaatgtgtttggaaaccagtccaacttaagtcgagtgttcgaagtaaagaaagccatcaacgatctttctcaaggagacatggagttcactcaacactttggaaagtttaggtctctttgggctgaacttgagatgttgagacctaacactctagatccaaaggtcctaatcgaaaggagagaacaagataaagtgtttggtcttctccttactttgagctctacctacaatgatctcatcaaacacctccttcgagctgacaagctcccaaatcttgaggaagtatgctctcaaatccaaaaggaacaaggttcactcgggttatttgggaacaaaggagagctagcttgagtcaatctaagtgatctcgctacagctaaccgagggaattacaagtatgacaacaacaaaaaggctctatggtgtgaacattgcaagagaagcggtcacacaaaggagaaatgttggacacttcaccctcacctaagacccggaaggagagaaccaagagcgaatcaagtaaccggagagaactttggaactcaagagcaatccggaacatcaaaccaacacttgggaggtaatggggcagcaatggcagcttcctctgacctagtgaggcgttccgacctcaaagctctcattaaggctctaaaggagtcctccggtaaatcttatcatgcccttagctctttaaagcctcttattattgactcgggagcttctcatcatatgattagtgattcgaagttaataagtaatattgaaccggctttaggaaatgtagtaatcgctaatggtgatagaattccggttaaaggagtaggtgatctagatttgtttgataaaagctctaaagctttctatatgcctacattcacctctaacttactatccgttaagaaagctacaaccgatttaaattgttatgctatctttggtcctaatgaggttcattttcaggatattgagactagtagagtgcttggtcaaggagttacaaaggatggactttatgttcttgaagacacaaagccatccgttcccttatcttctcattttagttccattcttggtaatgcgaatagtgaaagttggcatgctagactaggtcatcctcactctcgcgctttaaaacttttgttgccaagtacttcttttaaaaatgatgaatgtgaagcatgtattcttggtaaacattgcaaatccgtgtttcccaaatctagcactatctatgagaaatgttttgatcttattcactcggatgtgtggacatcaccttgcttatctagagaaaatcataagtattttgtaaccttcattgatgaaaaatcgaagttcacttggtttacgttattaccttctaaagatagagtcctagaagcttttactaactttcaaacttatgtgactaatcattatgatgctaagataaaaatcttaaggtcagataacagaggggaatacacaagtcatgcctttaaacaacatctaaacaaacatggaataatccatcaaacaagctgcccatacacaccacaacaaaatggagtagctgaaagaaagaataggcaccttatggaggtcagacgagtcatgatgttccatacaaatgttccaaaacatttttggattgatggggtggtctcagcgtgttacttaatcaaccaaacaccaactaagatcttactagactcttctccctttgaagtgcttaataaagttaaaccgtttatcaatcacttacgagtatttgggtgtgtgtgctttgttcttatttcaggagaacaaaggaacaagctacaaccaaaaagtacaaaaggcatgttcattggttactctattaatcaaaaggggtataaatgttatgtactagaaacaagaaaagttcttatctctagagatgtgaagtttctcgagtctaaaagctactatgataagaagaattgggaagatattcaagatcttactgactctccctcggatagagctacaaatctccggatcatccttgaaagacttggtgtaagtaatattcagactcaaacaacaccaagaacctcaaatccggagactatcacacaaccagagaacatggaagaagaagaagaagaagaagaagaagaagaagaaaaacaaggaaaagaacaagaactaattactcttgaagaaacggagagttctaaggttcaagagaaggatacaagcttacttaacgatgacaatggtcatactaataaccaagaagaagactctaactctcgagaagaaccgagaataccaagaagaagtgaacatcttaaagacaagagagtctactacaacaaccaagtctactttgacaatgtcgtggaacacccaattcaagttgtgtgcaccctagctcatctacccgaagaacatcaagtcttttttggaaaagtagaccaacattggatccctcaaacttatgaagaagctataactcatcaagtatggagggacgctattgcagccgagaaacaagcaatggagaacaaccatacatgggatgaagatgagcttcctagaggtaagaaagttgtcacctcgaagtgggtcttcgccattaaatataagagtgacggggagattgaaagatacaaggcaagacttgtggcaagaggatttactcaaacgtatggggaggactatctagatacgtttgctccggtcgcaaagttacatacagtccgagtagtactctccttgacgaccaatctcgagtgggatctttggcaaatggatgttaaaaatgcgtttcttcaaggagaactcgaagaaaaagtgtacatgaagccaccaccgggtctagaagacattaatgctcccaataaagtctttaagcttaagaaagccatctacggtttaaagcaatctccgagagcgtggtaccacaaactcagcaccacattgatgggaagagggttcaaaaggtctgaagccgataacactctcttcactttaccaagccaaaaaggtatagtagtcattctcgtatatgtagatgatattatcatctccgggaatgacaaggtaggtattcaagatacaaaaacttttcttaaaagtgtttttgatattaaagaccttggtgagttaaaatatttccttgggatagaagtttgtagatcaaaggaaggtctcttcttatcccaaaggaaatatacacttgatcttcttgcccaagtaggtaaactcggggtaaaaccggccaagactccactagaggacgattacaaggccaaacgaaagggggagcatgataacaaaccatttgaagatgctacgagatatagaagacttgttggtaaacttatctacttaaccataacctgacctggcatttgttttgttgttaatgtagtaactcaacacatgcaagctcccacaatacatcattggaacatggtgaatcgcattctcaaatatctcaaaggagctccgggtcaaggcatttggatgggatgcaatagaaacacggagattgttggatattgtgatgcggattatgcctgacatcaacaagataggcgatctacaacgggctattgcaccttcattggaggaaacctagtaacatgaagaagcaagaaacaaaaagtagtgtctctctcaagtgttgaagccgaatatagagccatgagaaagctcactactgaactcatgtggttgaaagctctccttaaagactttggcactgatacaccaaaccccattccaatgcattgcgacaatcaagctgcgattcacattgcctccaactcggtgtttcacgagagaacaaagcacattgaagtagattgccacaaagtacgagaacaagtacaactcggagtgatcctaccacattacactgagggtgaggaacaattagccgacgtcttcaccaaaggagcgagcaccaaggtgtgtgaatatattcacaacaaacttggcctagttgatctaacccggccatgatcctcttatcaaatatcccatactctttttcccttgtgtgtagttttatcccaagaggtttttctacactaaggtttttaatgaggtggttgaatttggttccaagcttaatctcgactcatctccggttaagcttgagggggag

>ATCOPIA20_LTR

tattgaacctaatgaaggaaaccccttccgcaagttaacctagacgaccgtacggacgaagagaagcccatccgtacaacatcacaatgagagaagcccacataaactcttcttcaccttaagctcttctagaagacctagaagactcctcatgtagccgttgaagcttaagcgcccacgtcaaaagaagatcacatgtgtttaaagcaaaccaaacgttaggtcatgtctctttctttagtacgatagtgatttgtctacacgatgtggtcttgtatagagtgcgacgtgtttagcatattcttaggagatctttacttacctaatgtccatatatagcttgtatcgtatgtatgtttttaatcaagaaatagagaaaatctcttttctcacaatctctcttctatttccgcaatactatctcttccttatgttcatttctcactctcaaccctcttaatcaagagtcccattctcgcaacctaaagctccatattcttaca

>ATCOPIA21_IN

tggtatcagagcagtgattctaaaagacctaattttttttttttttttaaaacaaaagagccgcctctctcttcgacctttcttcttcttctcgctccccttctttctctacaagcttgctcttcaagcttcatcatttccggctatgtcttcctcctcagaaaccgttgttgttgctgattctacctctctcctcaatgtgaacatgtccaatgtcacaaagctgacatcaacaaactttctcatgtggcgtcgtcaagttcatgcgctattggatgggtatgacctcgccggttacgttgatggttcgatcgaagaacctcacaccacagtcactgtccatggtgttacctctcccaatccagaatacaaactctggaaacgccaggataaactcatttactctggcctcatcggtgccatctctgttgcggttcaacctctcttgtcccaggctaccacctctgcccagatctggcgtaaactcgttgacacttacgcaaatcctagccgtggccacaagcaacagatccgtgaacaaatcaaacaatggaagaaaggttctcgttccattgatgactacgttcttgggctcactactcgttttgatcaacttgcactacttgaagaagccataccacatgaagatcaaatcgcgtacatacttggcggtctctccgatgattataggcgtgtcattgatcaaattgaaggacgtgatatttcgccttcaatcacagagctacacgagaagctcattaactttgagcttaaactccaagcgatggtccctgattcctctactccggttaccgctaacgcagcctcctacaacaacaacaacaacggccgtaacaatcgctcttcttctcgtggcaatcaaaacaaccaatggcagcagaatcaaacacaacagtctcgctccaacaatcgtggctctcaaggcaaaggctatcaaggtcgttgtcaaatctgcggtgtacatggtcatagtgcgcgacgatgctctcagtttcagccctatggtggcagtggtggttcccaatcagtgcctagcggttatccaaccaacggttattcaccttctccgatggctccatggcaaccacgagctaacatcgccactgctcctccgtttaacccatgggttctagatagtggtgcgacacaccatctcacctcggatttggcaaatctctcaatgcatcagccctataccggtggagaagaagtaaccatagcggatggttcgggtctacctatttcacacactggttcagctttactccctacaccatctcgatctcttgctcttaaagatatcttatatgttcctaatgttagcaagaatcttatatcggtttatcggctgtgtaatgctaaccaagtttcggtggaattctttcccgcacattttcaggtgaaggatctcaacacgggggcccgattactccaaggcagaactaggaatgagctatatgagtggccggttaaccagaaatccatcactatccttaccgcgtctccatcaccaaaaaccgacctctcttcttggcatcaacggcttgggcatcctgctttacccattttaaaagatgttgtttctcattttcatttaccactttcaaacactattccaaaacagcttccatgttcggactgttctatcaataaaagtcacaaattaccatttttcacaaacaccattgtctcttctcaacctcttgagtatctatacaccgacgtttggacgtctccatgcatttcagttgataactacaagtattaccttgtcattgttgatcacttcacaagatatacatggatgtatcctctcaagcagaaatcgcaagtgaaagatgtgtttgtggctttcaaggctttagtggaaaaccggtttcagtcacgaatacgaaccttatactctgacaatggcggtgagttcattggccttcgcccgtttcttgctgctcatgggatttctcatctcacaagcccgcctcatacaccagaacataatgggttagctgaacgaaaacatcgtcatatcgtagaaaccggtcttgcgcttctcactcatgcgtcattacccaagacattttggacgtatgcttttgcaactgcggtgtatctaatcaataggatgccaacggaggtcttacaaggcacgtctccttatgtcaaactctttcaaatgagtcctaattatcttaagctccgagtatttggctgcttgtgctacccatggctacgtccgtacaacactaataagcttgaagcacggtcaacgatgtgtgtgtttcttggatattcgcttacacaaagcgcatatttgtgtctcgacatagccaccaaccgcatctacacttctcggcatgtccagtttgttgaatcaagttttccgtttgcttctcctcgaacatcggaaaccgactctacacagacaatgtctcaaccgaccaccaccaatgttatcccgcttctacaacgaccgcctcatatagctcctcctactgccttgcccttgtgtccgatctttcactctccgccgcactcaccgtcgtctcctgcctctccaccttcagagcatgtgccattgaccgccgcttcatcatcgtcaaacgccattaacgacgacaatatttcctccacaggccaagtaagtgtttctggcccaacttcacaaagcccacatactacacctacaaaccaaaacacaagcccactaagcaaaagcccaaatccaaccaatacaaatcaatcccaaaattccacgcctcctacttcgccaactacgtccgttcaccaacactcacctactccgtcaccactaccacaaaatccaccgcttccaccacctccacaaaacgaccaccctatgcgtactcgagccaaaaatcaaatcacaaaacctaaaaccaaatttaacctcaccacatctctcacgtcttctaaaccgacaataccgacgacagtagcacaagctctcaaagatccaaattggagaaatgcaatgtcagaggagatcaatgctcaaatgaaaaaccacacatgggatttggtgtcaccggaggaagctaaacatgttatttcttgtaagtggattttcacgcttaaatataacgttgatggctctattgcaaggtataaggcgcgacttgtagctcgaggcttcaaccaacagtatggcattgactattcggagacgtttagtccggtgataaagtcaaccactattcgtactgttctagaggtggcggttaagcgaaattggagtattcatcaagtcgatatcaacaatgcatttcttcaaggaactctaaacgaagaggtgtatgtttcgcagcccccgggatttatagatcgtgatcggccaagtcatgtttgccgcctcaataaagctttatacggcctcaaacaagctcctcgggcttggtatcaagaactacgacgatttctcctacaagctggttttgtcaattctctagccgatgcctctctatttatctataatcggcataataccttcatgtatgttcttgtatatgtcgatgacataatcattgcaggagaaaacgctcttgtacaagccttcaatgcctcacttgccagtcggttttctctaaaggatctagggcctctcagttatttcttggggatcgaggccactagaacttcacgcggcttacacctaatgcagcgaaaatatataaccgacttactaaagaagcacaatatgcttgatacaaagcccgtgtccacaccgatgtcaccgacgccaaagctctctcttctctccggaacggctcttgatgatgcaacggagtatcgcaccgtccttggcagcctccaataccttgcgtttacacgcccagatatagcttttgcggtaaatcgcctttctcagttcatgcatcgtccaaccaatgaacattggcaagctgcaaaacggattcttcggtatctcgcgggaacaaaatcgcatgggatcttcctccgctccgatacgcctctaacaatacatgccttctcggatgcagattggggatgtgatctggatgcatatctctcaaccaatgcctacatcgtttattttggtggcagtccggtatcatggtcctcaaagaaacaacgaagtgtcgctcgctcatctacggaggcagaatatcgagctgttgcaaacaccgcctcggaactacgttggctctgctctcttcttttggaaatgggcatatcacaaaccacagtcccggtcatttactgcgacaacattggcgccacatatctatgcgcgaacccggtgtttcattctcgtatgaaacatgtggctcttgattatcactttgtcagaggttacattcaatccggagcattgcgagtttctcatgtttcgactaaggatcagctagctgacgccttaaccaaacccttaccacgtccacgttttacggagctcaatagcaagattggagttcaagaattacctccatcttgagggggtg

>ATCOPIA21_LTR

tataaggagttgtatatattaaggatataattgtcattagtagttgtgtataccctagactaagtactatatatgttgtaacacattcatcataataagacaattcactctcttatcaca

>ATCOPIA22_IN

tggtatcagagcgcaatctgggtttttcgaattgagtatttcaagtgagttttctctgaagctatggctgatgctccaccaccacctccatctgtaattgaggtccgacgcacaatttcgccgtacgacttgacggcagctgacaattcgggagctgtgatctcccatccgatactgaagacaaataactatgaagagtgggcatgtggtttcaagacggcgttgcggtctaggaaaaagtttggtttccttgatggaaccattcctcaaccactagatggttctccagatctagaagattggttaacgattaatgctttgttggtctcttggatgaagatgactattgactcagagctgctgacaaatatctctcaccgtgatgtggcgagagacttgtgggaacagattcggaagcgtttttctgtttctaatgggccaaagaatcagaagatgaaggcggatctcgcgacttgcaagcaagagggtatgacagtggaaggatattatggaaaactcaacaagatttgggataatatcaacagctatcgccctcttcggatatgtaaatgtggtaggtgtatctgcaatcttggaacagaccaagaaaaagatcgagaagatgacatggttcatcaatttctttatggactgaatgaaactaaatttcacaccatacggtcaagtttgacgtctcgtgttcctctccccgggctggaagaggtttacaacattgtgcgacaagaagaagatatgttgaacaacagatcgtctaatgaagagagaaccgatgtcactgcttttgctgttcaaatgcggccacgttctgaggtaatctcagagaagtttgcaaattcagagaagcttcaaaacaagaaactttgtactcattgtaaccgtggaggtcattcgccggaaaattgctttgtgttgattggatatccagagtggtggggagatcgcccaagaggaaaatcaaattcaaatggctcaacaagtcgtggcagaggaagatttgggcctggttttaatggaggacaacctcgtccaacgtatgtgaatgtcgtcatgactggaccgtttccatcttctgaacatgtgaaccgggttatcactgatagcgatcgtgatgcagtcagtgggcttactgatgagcaatggcgtggagttgttaagcttctcaatgctggaagaagcgataacaagtccaatgctcatgaaactcaatcaggtacatgttctcttttcacttcttggatactcgacacaggtgcttcacaccatatgactggaaacctggaattgttgagtgatatgcgatccatgtctcctgttttaattatcttagctgatggaaataagagagttgctgtcagtgagggaacagttagattaggctcgcatctgatattaaaatcagttttctatgtcaaggagttggaatcagatttgatctctgttgggcaaatgatggatgagaatcattgtgtggttcaacttgctgatcacttccttgtgatacaggaccgcactacgaggatggtgactggaattggtaaaagagagaatggaagtttttgttttcggggaatggagaatgctgctgctgtgcacacgagtgtgaaggccccttttgatctatggcataggcgtttgggacacgcatcagacaagatagttaatttgttgcctagagaacttttgtctagtggtaaagagattttggagaatgtatgtgatacatgtatgcgagctaagcaaacccgtgatacttttcctctaagtgataatagaagtatggatagttttcagctaattcattgtgatgtgtgggggccttatcgaacaccttcttactccggtgctcgatatttcttgacaatcgttgacgattattctcgtggtgtgtgggtttatttgatgactgataaaagtgaaactcagaaacatttgaaagatttcattgctttagttgaacgacaatttgacactgagatcaaaacagtaagaagtgataatgggactgagtttctatgtatgcgtgagtactttctacacaaaggcattgctcatgaaacttcatgtgttgggactccgcatcaaaatggaagggttgagaggaagcatcgacatattttgaatatcgcgagagccttgcgatttcaatcgtatttgccaattcagttctggggagagtgtatcttatctgcggcttaccttatcaaccgcactccctcgatgcttcttcaaggtaaatctccatatgagatgttatataaaacggctccgaaatactctcatcttcgtgtatttggttccttgtgttatgcacataaccaaaatcacaaaggagataagtttgcggcacgatcaagacgatgtgtatttgttgggtatccacatggacaaaaagggtggaggctgtttgatttagaagaacaaaaatttttcgtgtctagagatgtgattttccaggaaacagagtttccatactccaagatgtcgtgcaatgaggaagatgaacgtgtattggtggattgtgttgggccaccttttattgaagaagctattgggccaagaactattattggacgaaatattggtgaagctacagttgggccgaatgttgcaactggcccaataatacctgaaatcaatcaagaatcttcaagtccaagtgagtttgtctctctctcctctctcgatccttttttagtatcaagcaccgtacagacagcagatcttccactgtcatctacaacaccggcgccaatacaacttagacgtagttctcgccaaacacaaaaaccgatgaagttgaagaattttgtcaccaatactgtgagtgttgaatcgatttcccctgaggcttcttcctcctctctgtatccaattgagaaatacgtggattgccatcgattcacttcgtcgcacaaggcttttctcgcagctgttactgctggaatggaaccaacaacttacaacgaggctatggttgacaaagcttggagagaggctatgtctgctgagattgaatctctgcgagttaatcaaaccttctcgattgtaaatttgcctccaggaaagcgggctcttggcaacaaatgggtatacaaaatcaagtatcgatcggatggggcgatagagcgatataaagctcgtttggtagtcttgggtaattgtcaaaaagaaggcgtggattatgatgaaacttttgctccggttgcaaaaatgagcacggtgcgtttgtttttgggagtggctgcggcccgtgattggcatgtacatcaaatggatgtgcataacgctttccttcacggtgacttaaaggaggaagtgtatatgaaattgcctcagggatttcaatgtgatgatcctagcaaggtatgtcgtcttcataaatctctgtatggtttgaaacaagctccaaggtgttggttttccaagctctcttctgctttaaaacaatatgggttcacacaatctttgtcggattactctctgttcagttacaacaatgatggtgtctttgttcatgtcctcgtatatgtcgatgacctgatcatttcgggtagctgccctgatgctgttgctcaatttaaatcctatctagaatcatgttttcacatgaaagaccttggcttgctcaaatattttcttggcattgaagtgagccgcaatgctcaaggattctacctatcccaaaggaagtatgttcttgatattatttctgaaatgggacttttgggggctaggccgtctgcgtttccgcttgaacagaatcataagctctcgttgtctacatctcctctgctttcagactcttcacggtatcgtcgtctagttgggagactcatttacttggctgtaacacgtcctgagctatcatattctgttcacactcttgcacagtttatgcagaatccgcgtcaggaccattggaacgccgccattcgtgttgttcgctatttgaaatctaacccaggacaagggattttactctctagcacctctactctgcagattaatggttggtgtgatagtgactatgctgcttgccctctcactcgtcgctctctcacaggatactttgtacagcttggtgatactcccatatcttggaaaacgaaaaagcagcccactgtcagtcgctcttccgcggaggctgaatatagagctatggcattccttactcaagaactcatgtggctcaagcgggtgctttatgatcttggtgtctctcacgttcaggctatgcgcatcttctctgacagtaaatctgcaattgctctcagtgtcaatccagttcagcatgagcgcaccaaacatgttgaggtggattgtcattttatccgcgatgctattcttgatggtataattgcgacttcgtttgttccctctcacaagcagcttgcagatattctaactaaggctttgggtgaaaaggaagttcgttactttctgcgcaagttgggcattctcgatgtgcatgctccaacttgagggagga

>ATCOPIA22_LTR

tgttgaagctattgctggtcaatattttagttagcttagtcaacaatatatatcctgtttagtcatttcctttgtgcaacactttaggttatatcttttgctttgtataagtaaggttttaacgcctctttaataagataagacaacataaaccaaactacatcttgtcttaagtttctca

>ATCOPIA23_IN

agtggtagcagagcttttgttcttaaacgctcaaagaaatgagtgagaaagatggtttgagcattccaaaattcgacggagattacgaacactgggcaatgctcatggagaatctcatgaggtccaaggagtggtgggatctcattgagcatggagttatgcagccagagagaaacgtgatcctgacgggagcccaaagaacagagcttgcagagagtaagctcaaagatttgaaggcaaagaattacttctttgcttcaatcgacaaggcaactctgaagacgattgcaaagaaggacacagctaaagatatttgggagtccatgaaaacaaagtaccaggggaacaagaaggttcaaagcgcccaactgcagagactgcgaaggaactttgaagtgttagaaatgaaagagggagacaccattgatgggtatttctcaaaggtgatggtggtggcaaatgacatgagaaaccttggtgagagcatgccggatgcaaagattgttgagaaaatcctgagaacattggtggagaaatacacatatgtggtgtgtgcaattgaggagtcaaatgacatcacagaactctcagtggattcattgcagagctcactactagtgcacgaacagaagctgagtagacatggagatgatgagcgtgcacttaagattgagggcaggtgggattctagtggaggaagaggtcgtggtggacaccaaaaccgtggaaaaggaagaggaggatatcaaggacgtggcagaggtagcacaaacttcaacaaagaaacagtggagtgctacaaatgtcacagactggggcatttcaaattcgaatgcccggagtgggagaagactgctcattatgcagaactggaggaagatgttctgctcatggcgcatgttgatattcaaagagcaggaaacgatgaggtctggttcttagattcggggtgttcgaatcacatgtgcagcacaagagagtggtttgtggagtttgactcaacattcaagcatagtgttaaacttggagataacaggaagatgagtgtagaaggaagaggaagtctccgactcatcattaatggggcagcacaaaagataacaaaagtttactatgtcccaaagttgaagaacaatctcttgagtgttgggcagctgcagcagaagggactgagaatcatcattgaagatgatgtgtgtgagatctggcacaagcagcagagaagactactcatgttctcaactatgacaaaaaaccggatgtttgtcatacaggcagcagtgagaggagacaaagaaaaagaagaagggaactgtctgcaagctgtttcagatgagaaagttgaggaactgtggcataggcgacttggacacctgaatcaaggaggaatgcagagtttgtcagagaaacagatggtgatagggatgcctatgcaaacaaagaccaatgctgcagagttgtgtgatgtatgcatgaagggaaaacagaacaggcttagcattccaaagaagagttcatggagagcaagtcggggtctccaattggtccacacagacatctgcggaccaataactcccacatcagaaggtggcaagaggtacatcattaactttattgatgactatagtcgtaagtgctggacctatttcttgtcagagaaatcggaagctttgagagtgttcaaagagttcaaggtggctgcagagagggaactcggagtgagtttgatatgcttgagatcagacagagaaggcgagtacaactctaatgcgtttcaggagttctgtaaggaacatgggattaaaagacaactcactgcagcatacactcctcaacagaatggagttgcagagaggaaaaacaggagcctgttgaacatgacaaggtgtatgctgtttgggatgtcagtaccaaggagattttggccggaagctgtgcaatatgcagtacacatattgaatagaagtccctctaaagctcttgcagatgctacaccagaagaaatgtggagcaagcataagccatcaatcgatcacctaagagtgtttgggtgtgtagggtacgcactagtgccatatgagaagagaattaagttggatgaaaagagtagaaaatgtgtgatgctgggagtgagtaaagagtcaaaagcataccgcctgtatgaccctgagacaaagaagatcattatcagtcgtgatgtgcagtttgatgagaaactaaggtggaattgggaagaggatcagcaagaagactcacctaaatgggaagagagtgatgctgattctgaaaaaactgcagagaacgctgaaaatgaggagagagaagcagaagaggacgaacctgcagcagtcactgatacacctgcagcagtcacagatacacctgcagaaaacgaacaagctccagtcagaggaaccagtggcagaacgatacagccacctgtgtggatgaaggattatgtaacaggaggaaaaagcttgtttgttattgatggtgatgaggtaatggcgctgtatactgcagcagaggatccagagagcttcgaggaagcagcccaacatgagaaatggagaaaagctatggaagtggagataaaatccatagaagataacaacacttgggagatcatagagctgcctgaaggtgcaaaagtaattggagtaaagtggatttagaaaaccaaatacaatgagaaaggagaagtagacaagtttaaagccaggctcgtggcaaagggataccaccagaggcaaggaattgacttcgaagaggtgtttgctcctgtggccagatgggatactataagaaccctacttgcaaaggctgctcaaaaggggtggaaagtgtttcaactggatgtcaaaagtgcattcttacatggtgaattgaatgaagatgtttacatcgaacaaccacagggctttgaagtagaaggagaagaggagaaagtttacaaactcagaaaagctctctatggtctgagacaagcacctagagcatggtacagcagaatagaaagttattttgctaaggaggggttcaagaaatgctactgtgagcataccttgttcgtcaaagcagagcgtggaggggttctgattgtgagtgtgtacgtggatgatctcatatatacaggtaactcagactccatgctagctgactttaaggcgtcgatgacaaaagagtttgctatgacagacttgggtttgatgaaatatttcttgggggtggaagtgattcaagatgacaaaggaattttcatcaaacaacaaaagtatgctgcagagatacttgaaagatttggtatggagaggtgtaattcggtcaaaaatccaattgttcctggaaacaagctttcaaaggaaggtgctggagcagaagttgatccaacaacattcaaacagctggttggaagcttaaggtacctaactgcaactcgaccagatctcatttcttctgtgaatttggtgagtagatacatggaacatccaaatgagcaacacatgttggcagtcaagagaatcctgaggtatgtacaagggacagcagggtttgaaatttagtattggcgtggtaacaaggaggagctggttggtttctctgacagtgagtatgcaggagacattgatgataggaaaagcacttccggctatgtgtttatgttgggaggaggagcagtatcatgggcgtcaaagaaacaagccatagtaacgttgtcgacaactgaagctgagtttgtagcagcagctcatggagcgtgtcaaggaatctggttgagaaatgtacttgaagaaatcggtgaaacactagaagaaggaacagttatgtactgtgataacagctctactatcaagctctcaaagaatcctgtactgcacggaaggagcaaacacattcatgtcaggtatcatttcctgcgtgaacttgtcaatgatggagtaatgcaaatcaagttctgtcctacacacgagcagttggctgatggcatgacaaaggcacttaagttggcgacgttcgtgtatctaagagagaagatgggagtatgtctgaaagaagagtaaactggaaaggagttccagtttaagggaaggat

>ATCOPIA23_LTR

tgaagaataaagtccgttgctttcagtttcagtttgaataagtcttatgactttgtttgtctttcaaataacatccctaagtattaggagatagactagcatttcagttccgtttatttagcagtctgttgcattcttgttatctttgtaacttgatctatttaagatctctatcgtctatgagaataacacacaagttacacatttgttctctgagtcactagttcgtttacaaca

>ATCOPIA24_IN

tggtatcagagcacaataacattccgcgatcattgtgttcatcggaagagagctttgttctttgtgttacgttttcttcttcgattctttgaagtttctgattccgattatccggaattttccgttctgtttcattttttctttcattttgaccttaacgttgacgattctcgttttcgattcatcaaattcgtcacgttttctttgtaatttctcattcttacactgagaatgggatcttatgtacctcaatctactattcgggtgactcgtcctccgatcaacaacaatggtgatcaatttcgttatccagctgatcaatatgagaatccttactatcttcatagtgcggatcacgctggactcattcttgtatcagatcgactaacgactgcttctgattttcattctggcatcgatcgatcttgatggctttaaatgttagaaacaagttgggattcataaacggtacgatttctaaaccaccaaaagatcatagagactttggtgcttggtctagatgcaatgacattgttagtacttggttaatgaatttggttgataagaagataggtgagagtttgctttttataaaagatgaaagacagaattctgtgaaaccatctactcggattgataatgtgtcttttcagagtgttgctcccatgatgaatgatgtagagaatgcatatattgcaacttataatactgtcaaagcaagtgagaaaccgatttgttctcactgtgggaaagtaggacatacggttcagaaatgttacaaggttcatggttatccaccagacatgaaaataggcaatcaaggatatacttacaaggttaatccacagttacaagttcaacctaggatacagatgatgccgagtcagccaaggatgcaatttcctaatcagatgcaaatgaagccatatgctaactcaatgcagaaagctaatgatgtggctcatgtttacaccgagaattgtgcttatctgtctgaaggctactcttttaatcttatgatggttccttatggttcatatggttctaatattcaacagatgcctcatgtcacacaaggtggaaacaatttaagtctacaagattttactccacaacagatagaacaaatgatttcacaatttcaagctcaagttcaagttcaagagcctgaagcttcttcgggtaatcctagtccaacatctacagtctcagaacatggtcttatggctcttacttctacttctggtactataattccctttccttctactagccttaaatatgagaataataatctcacttttcaaaatcatatactttcaactcttgaaacatttctatcgcataattctcggattatagatagtggagcttctagtcacgtttgttcagacttagtaatgtttagggaattaaagcctgtttctggagttactattactgttaccaaatggtactagggtctctatcactcatacaagcactgtgcatataactcataagttgattttgcataatgttttgcatgttcctgattttaaattcaatttgattaaaactttggcatgttcagctcatttctatgtttcttgttgtttaattcaggaactttctcagggcttgatgattgggaggggtaaactatatcacaatctttacattctcgagacaggaaataaatccctctctacatcaatacctgaagcttgttcttttactggatctgttcttgatgatggcaatttgtggcaccagggtttaggtcatccatcatcgcctatcttacaaaaacttgttagtcatattccttctttaaagtcatttcattgtgatgtttcttcttgtaaaatctgtcatttagctaaacagaaaaggctagcttatgtgtctcatgataatctagctgaaaaactatttgatcttgtttatttagatatatggggtccttttagtattgaatcaattgaaggttttagatattttcttaccttagttgatgattgtacttgtttagttgatggactacttggatatacatgttgagaaataaaagtgatgtgtctagtgttttcctgcatttctgaaacttgtttctacacaatttaatacaaaagttaaagcaattctttctgagaacgcgcctgaattagcattcactgaattgattaaagaacaaggcatgattcattacttttcatgtgcttatactcctcaacagaattctgttgttgagagaaagcatcagcatttgcttaatgtagctagggcattactctttcaatctaaagttcctatgcaatactggagtgattgtgtaatcactgcaactttcttgataaatcgtttgtcttcaccattgcttaacaacatatcaccttatgaacttctacttaaaaggaaacctgattacagtttattaaagaattttggttgtctctgttatgtttctacgaatttacatgaaagaaataagttttcttctcgtcctaaaccctttattttcttaggatatccttctggatataaaggttataaggttttaggcttggaatctcacactatttctgtttctcgtaatgtgatctttaaagagactgattttccttttaaaattagtgaacttctttctaaagttgttgatgtgtttttgaatactatcttgcctttacctgctccattagattttgttgactcattgccattaatagatgaagatacattgattcctactgcaccatcatcatcatcatcatcatcatcatcatcatcatcatcatcatcatcatcatcatcatctgcatcatcatcatcatcatctatattagtccctagtacggttaatcataataaagaagcattaggaacagaccttaatactgtacccattactaggtctaaacgaactactagagctcctggttatctttctgaatatcattgctcattagttccttttacttctaatctaccacctacaaatactattccctctgagactttattatctgttacacttcgtgaagcaccatcacctaaaaagactacaccatatcccatttcatccgtcgtctcttatgatacgtatacacctctttttcaatcctatatctttgcttctaatgctgagatagagccaaagacgttttcaccggctatgaaatccgaaaaatggactaaggctgctaatgaagagcttcatgctcttgaacagaacaagaattggatggttgaatcattacctgaagggaagaatgttgttggctataaatgggtgtttaccattaaatacaaccctgatggaacaattgaacgctacaaagcaagattggtagcacaaggattcactcaacaggaaagtattgattacttggacactttctcacttgttgctaaattgactagtgtaaagatgatacttggtcttgctgctgcaacgggatggactctaacacaaatggatgtgtccaatgcattcttgcatggagagcttattgagatcttcaagagtattcctcaaggatacaccccacctgcaggtgtatctcttccccctaatccggtatgtcgattactgaaatccttatatggccttaaacaagcttcacgtcaatggtataaaagattgtcatcagttcttcttggtgcaaacttcatccaaattcctgctgacaacactttattcatcaagtttacttctgcatcttttgtggcagctttggtttatgttgatgatatcatgattgcaagtaatgatgataaggctgtggaagaacttaaagctttgttaaggtctgagtttaagattaaggatcttggtcctgctagatttttccttggattagaaatttcaagattttcacaagggacttcagtctgtcagaggaaatatgctcagaacttgttggaggatgcaagattacttggatgcaaaccaagtttgatacctatggatccgaatctgcatttggttaaagatatggaactctattaccaaatccaactacttatagggaactcattggtcgactgctttatctcactattacaagaccagatataacatttgcggttcatcaactgagtcagttcatttcagctcctactgatattcatcttcaagcagctcataaggttctccgatatatcaagacgaatccaggtcaaggattgatgttctctgcaaatgttgaattgtgtttgaatgcattctctgatgcagattgggaagcttgcaaagatactagacgttcagtatcaggcttctgtgtttatttaggtacttcactcatctcttggaagtctaagaaacaggcagttgctagtcgaagtagtgctgagtctgagtacatggctatggcacaagctacttgtgaagtgatttggctacagtagttgctcaaggatctgtatattcaagttacttgtcctgcaaaattattttgtgacaataagtctgctctacacatctcgatgaatccggtcttccacgagagaactaagcacattgagatcgattgtcacacggtaagggatcaaattaaagctggaaatctcaaagccttacatgttccgactgagaatcagcatgctgacattcttacaaagtctttacatccaggtcctttccaccatcttctgaagcaaatgtcgttgtcaaatctctaccttccaaacgagacatttaaaagatagagatttgagggggta

>ATCOPIA24_LTR

tattgcagatataaccggttattgatgttggttatttccggttatagatgttggtttaatcttaagtatataagttgagttaatgacattgtaaatgattaagttgttaacattctgttttactcaataatgaaagtttgttagcttcttcactctcttctccgatttacttatccggtacattgtaccttcaata

>ATCOPIA25_IN

tggtatcagagccatggaacaatccatggaactctactctcttccatctctaaacatctcaaactgtgtcactgtgaaactcaccgaacgaaactatatcttatggaaatcccaatttgaatcgtttctgtccggtcaagggcttcttgggtttgtcaatggcgccgtctctgctccactcagcaccgttcctggaccacaaaacaatggtgtcaccgaagctgtctcgaatccggagcatcaagcttggctgcgcttggatcaggtggttatggcgtggctccttggcttgttgtctgaagacatccttagtgtcgttgttgggtctgcaacatctcacgcggtatggtctaacctagctaagcacttcaatcgcatctcatcttctcggatttttgagcttcaacgtcgtctacatggtttatcatatgtgaccaacttgcatctgtgggaagtccagttgcagagaagataaaggtctttgctatggttcacggccttactcgtgaatatgaaccgcttatcacctctcttgaaagttctctggatgctttccctggtccaccctatgaagatattgtctatagtctcaagggttttgacgaccgtctccaacgctatgcggtcacatctgacgtctctcctcacttagccttcaacactttcagatcttctaaccgtggtcgtggccagtcctctggaaggggaggaagaaacagaggccgtggtagtggaaacttctccacaagaggacgcggttttcatcaacagttctctggttcatcttctggagatcccaacactgagaaacccatgtgtcaaatttgtggtaaaagaggtcatcctgctctgcaatgttggcaccgtttcgatgacagttaccaagaggagtccgatgttgcagcagctgcgttcaatgctttacacattagtgaggtaacagatgatgccggctggtttccggatacaggagcaacagctcacattaccaacaatgctcaacgtctcagtcaatctcagccctactatggaacagatactgtaatggctagtgatggtaacttcttaccaatcactcacattggctctgcaaaacttccatccacgtcaggtatgcttcctttaaaggatgttttagtttgccctgatattgctaaatcactgttatctgtgtcaaagctcaccaaagactatccttgttgcttcacttttgattctgatggtgttgttgtaaaggacaaagcaacacacaaagttctgactctcggaaaaacaagtgatgggctttacacgttggagaatccaaagtttcaaatgttctactctactaggcaaatcacaacatctgatgaggtttggcatagacgcttagggcatccgaatgatcaagttctccaacatttatcttccaacaaggcgattaaaataaagaagagtaccaacaagctctgtgaaccatgtcggcttggaaaaagtacaagatagccatttttcccttctgcttttactgcttcaagacccctagaaagacttcattgtgatctctggggtccagctcctgtaacttctgttcagggtttcagattttatgtagtcttcattgataatttctcaagatattgctggttttatcttgaagaagaagtctgatttctatgatattttcattaaattccagagtcttatagaaaatcagttaaaaaaccaaaattggtacattccaatgtgatggagggggagaatttacaagtcaaagatttctgtctcacttacaagagtctggaatacaacaatacatcttatgccctcacacacctcaacaaaatggtctagctgagcgaaaacataggcaaatcacagaaatgggtcttactcttctctttcaaagcaagtgtcctcagagattttgggtagaagcttatttcacagctaactttctctcgaatcttctgccaaccactgctctggatactaaagcaagtccttatgaagttctctttaacaaagctccagactacttagccttaagacgttttggatgcgcatgttttcccacacttcgagactatgccatcaacaagttcgatcctcgatctctacggtgtatctttcttggctatactgagaaatacaaaggctatcggtgttactatccgcctacgggtcttgtttacatcagtaggcatgttgtatttgatgaagcctcatttccatttactgatacgtatagtaactacaccaactcctccaatacaccattacttgctgcttggctcaagagttttcctgcttcagcacctgaaacttttactcacacctcaaccactgctcctagtatcagtcccgtggaatcagtaacggttataacagaacctcatcctcagataataaatgcagagggggagaacatacttcacatcactgcaacagcgtctactgattcaagtcctatgagtgatgaagagtgttctgagtgtacggcaagcattgatcttgatcctattggcaacagtactacttcatcatctcctaggactgaacaatcagaagcttctgctgctcaacctactcattcaccgacagttgttcaacctattcatccaatggctacaagatcaaaatctggaataacgaaaccaaatcaacggtatgctcttcttacacatagagtaacatatcctatgcctaagactgttactgcagctttaaaggatccatattggactgaggctatgaaagaggaaattggaaactgctcagaaacaaacacatggtctttagtcccatttacacctgaaatgcatgtattgggatctaaatggatttttcgagtaaagcttaatgctgatgggtctctggataaatttaaagcaagattagtagctcaggggttcaatcaagaagaaggaatcgattaccttgagacatacagtcccgtagtcagaacagcaacagtgagagctgtacttcatttggctacaatcatgaattgggaattgaagcagatggatgtcaaaaatgcgttcttacacggggatctcactgagactgtgtatatgaagcagcctgctggatttgttgatacagaaaagcctgatcatgtgtgtctgctacacaaatctttatacggattaaagcagtctccaagggcatggtttgacaaattcagtacctttctccttgcatttggttttatctacaatttttctgacccttcactatttatttgcgtcagaaacaaaaatgttatcatgcttctcctatatgtggatgacatggtcattacaggtaacaactctcagcttcttaccaatcttctcagtgaactcaataagaagttccggatgaaagacatgggacagttgcattattttcttggaattcaggcacacttccatgaagatggtctcttcatgtctcaacagaagtatgcagaagacctcctgactgttgctgcaatgtcagactgttccccagttgcaacaccgcttccgcttcaactgaacaaagttcctcaccaaaaagagttgttcgaaaatccaacatattttagaagcctagcagggaaactgcagtacctcacactaactagaccagatcttcagttctctgtcaattatgtgtgtcagaagatgcatgagccaaccgtttcagactttttcctgttaaagaggattcttagatacatcaaaggcaccatcacaatggggatttctttcaataaacgaacagatacaaagttgcgggcttacagcgatagtgattggggaggttgtcatagaacgagaagatccactggaggctactgtaccttccttggcaataatctcatctcttggtcctcaagaaaacagccttctgtctccaaaagctccacagaagccgaataccgatcaatgtccaatacagcatcagagattacttggcttgttaatcttcttcgaagtctcagtgtccctcaattagagactccagaactcttttgcgataatctctctgcagtgtacctgaccgccaatccagcttatcatgcccgcaccaagcattttggaattcattaccactatgttcgcgagcaggttgcgtttggagaactcattgtcaatcacatcccagctcaccttcagcttgccgatatctttacaaaatccctggctgcagctccatttgagtctctgcgattcaaacttggtgtctgttttcccccgactccacgtttgagggggag

>ATCOPIA25_LTR

tgtcaaagctaaacagcaaaacgacgttacttctcaattgggctttcggacaacaaagcccaatccaacaaaaccaaacgctcagcccaaaccggagcccacagacgtacaacggtcacctgcaacacaagacaaaaacagagttgcagaacgatccctatcgccttacagtacggagctaaaggttcaaaaccgatttgaggcactcgcagcgcgtgatgacgcttaaccaaattccaagagacaaattcgcagccaaggatacattaggttaaatcatgtagctctatataagagcagcctcattgtaatccaaaattaaaaagttgaaagcaatataaacaaatatctttcgatttctcatttactttca

>ATCOPIA26_IN

tggtatcagagcacaatacctaactactaaaaggtttttgtgatcgattctgtgatctctccttccgctataagagatcgctttcttattccttctaacttctctacttaatacttgcatcaaactctccgttcatcatgagtactgaattggctctcgctagctccaccacaccagctaggactgaaacaaggcgtacaatctcgccatatgatctcacatccggtgataatcccggaactctcatctccaaaccgttgcttcgtggacccaactacgacgaatgggccacaaatcttcgtttggcgttaaaagctagaaagaaatttgggtttgctgatggatctattccacaaccagttgagacagatcccgatttcgaagattggactgccaacaatgctcttgtggtttcatggatgaagctcaccatcgatgaaactgtttccacatctatgtctcatctcgacgattctcatgagctctggacacacattcagaaacgctttggtgtcaagaatggacaacatgttcagcgactcaaaacagagttagcaacctgtcgtcaaaaaggagtggccattgaaacttactacggctgtctctctcaactgtggtgtagcttggcggattaccaacaagctaaaaccatggacgacgtcagaaaagaacgtgaagaagacaaattgcatcagtttcttatgggacttgatgaatctgtgtacggagctgtcaagtcggctcttctctctcgagtgccactaccttctctcgaggaagcttacaatgctttgacacaagacgaagaatccaaatccttgagtcgcttacataacgagagggttgacggtgttagcttcgcggtccaaactacttcacgacctcgagattcttcagaaaacagagtctgtagtaattgtggccgtgttggtcatctcgccgaacaatgtttcaaattaatcgggtatcctccttggctcgaagaaaaattgcgtcttaaaaacactgcatcttcttcacgtggtggtttaagttcgttcaaaggaaaacagagtcatggtcgtggatcttctattaatcatgtcgcttcttctggaatggctgccaatgttgttacgaattcatcgctcacttctccattgacatcagatgatcgcataggacttagtgggctgaatgattcacaatggaaaattttacaaacgattcttgaggaacggaaatctaccttaaatgatcatcagtctggtaagtattttcttgaatcttggataattgattctggagccacaaatcatatgactggttctcttgcatttctccgtaatgtttgtgatatgcctcctgttttgattaaacttcctgatggacgttttacaaccgctacaaaacaaggcagtgttcagttaggctcttctttagatcttcaagatgttctgtttgttgatggtcttcactgccatctcatctccgtttcacaactgactagaacacgtcgctgcatttttcagatcactgacaaagtttgcattgtccaggaccgcaccactctgatgctgattggagcaggtagagagctgaatggcttatattttttccgaggagtggaaacagcagctgcggtgactagtaaggctttgccgtcttcacagttatggcaccaacgtttaggacatccatcctcgaaggctttgcatttgttacctttttctgatgttactagtagtacttttgattcaaagacttgcgaaatctgtattcaagctaaacacactagagatccttttcctttgagcagtaataagacgagttttgcttttgaattagtgcattgtgatctctggggtctgtatagaaccacttcaatatgcggctctcgttattttcttactctagttgatgattattctcgagcagtatggttatacctcttaccatcgaaacaagaagctccaaagcatctcaaaaacttcatcgctctagtagaacgacaatacactaccaacatcaagattatccgaagtgataatggttctgagtttatttgtctctcggatttctttgcacaaaaaggaatcatacatgaaacttcttgtgtaggtactcctcaacaaaatggtcgcgttgaacgcaaacaccgccatatccttaatgttgctcgtgctctccgatttcagtctggtttacctattgagttttggagttattgtgctcttactgcagcatatctcattaacagaactccaactccattacttaagggaaagacaccatttgagctcatatataatcgtcctccaccactgcagcatattcgcatttttggatgtatttgttatgttcataatctgaagcatggaggagataaatttgccagtcgtagccataaatccatttttcttggttatccatttgcaaagaaaggatggagggtctataatatagaaactggtgtggtttcagtttcacgtgatgtagttttccgtgaaacagagtttcatttccctatctctgttatggattcgtcaccatcacttgatccggttctcgttgattcatctgaattagaagaaatctctatgactcctccggtcactccgtcttctccggctactccatcttctccggtcactccgtcttctccggttactccgtcttctccggttagtccgtcttctccggttactccgtcttctccggtcactcctctatcttcaactacaacctcagctgcgatagacaccattgaggatattaccactgatcttgaggattcaacctctatggatttttttcctgatgatgaagatgagttttctcccactgctacagaatctcctgcttcatcgtcatctcctgtacaccctccagcagttcaactagagcttctcggtaaaggtcatcgtccaaagagacctcctgttaaacttgctgactacgtcacgactcttcttcatcaaccattcccttccgcgactccgtatcctttggacaactacatctctagttcacgtttctcagataattatcaagcatatattctcgctattacatctggaaatgagcctcgaaactataacgaagctatgcttgatgatcattggaagggtgcggtttcagatgaaattggttcgcttgaaaatcttggtacttggacagtcgaagacttgcctccagggaagaaagctcttggttgtaaatgggtgtttcgtctaaaatacaaatctgacggcacacttgaacgacacaaagcacgtttggttgttcttggtaacaatcaaaccgaaggtcttgattacacagaaacttttgctccggttgcgaaaatggtcacagtccgcgcttttcttcagcaagttgtttcacttgactgggaggttcatcaaatggatgttcacaacgcgtttctccatggtgatcttgacgaagaggtatacatgcaatttcctccgagatttcgtactggtgacaagacaaaagtatgtcgtttacgcaaatctttgtatggtctcaaacaagctcctcgttgttggttcgcaaagctcacatcggcattgaagaactacggttttattcaagatatttcagattactctttgtttatctttcacaaaaacggagttcgacttcatgttttagtatacgtggacgatttgatcatcacgggtactactattgctgtgattacagagtttaaacattacttaagctcttgtttctatatgaaggatcttggaattttgcggtacttcttgggaattgaggttgctcggagtcctgaaggtatctacttatgccaacgaaagtacgcacttgacattatcacagaaacgggtctccttggtgttaaaccggcttcttttccattggaacaaaatcataagcttgcttttgccactggtgaaacaatagacgacccacttcgataccgtcgtctagttgggcgcattatttatcttgcgactacgaggcctgaattatcatatgttattcatattctctcacagttcatgcataatcctaaaccggctcattgggaagctgctcttcgcgtcgtcagatatctaaagtctagtcctggtcaaggtattctcctacgtgctaatacacctttggttctctctgcctggtgcgattcagactttggagcatgtcctcattcagatcgatctctcaccggttggtttattcagttgggtggctctcctttatcttggaagacacagaaacaaaatgttatttcccgctcttctgccaaagctgaatacagggctatggcagaaacagtgagtgaaatcatatggatccgtgaattacttcccgctctcggtattccgtgtactgctcctaccacactccattcagatagtttatcagccataagtcttgctgctaatcctgtctatcatgctcgcactaaacatgtgcgtcgagacgtccatttcattcgcgatgagctcgtcaatggcactatcgctactaagcacgtctcaaccacttctcagctagcggacattctcactaaagccttaggacgaaaagagtttgcagactttcttgccaagcttggtatctgcaatctccatataccagcttgaggagggg

>ATCOPIA26_LTR

tgttgggctgtgtggccttagcccatttatattgtataaattaactaggtctaaacttatcgtgtatataaaggcattgcctcttgccgtaatcaataagaatcttcttttcacaacctcgattgattcttgaca

>ATCOPIA27_IN

cgttatcagcacgagcttgctcttaacaaaaccctagcagccgtcgctcaaccctagctcgtccgccgtccgccgtccgctgttccgtcgaccgcctgtcgaccgtttgctgaccgtctgtcaaccgacgatacttccgtcgaccgccgactgtcaaccagcgaccaccgtttgataaccaaggtctttagctcccggttccagtccgtacaaccccaaaggagaaaggtaattcattaaaccctaaaactaaagaaccctaatcgaacatggatctaacaaacggctcccggttgattagtgtttgcaatatgattgcttgtttgctcgattgtttgtcttgttattgcttatcctaaacttgcaaatcgaaaacatcttaacctagaatccgaaaccctaaaagacataaaccctaaatcaaaaacactataatcggatcaaaaccctaattctatttcgtgattgtttgaagttatctagggtgtttagtttgattgatttcatggttgcatgcatgaattgttaaaatccctcatgttgcataaaccctaaaaccaaaaccctaaggaaaatagaaaatcgctaaaaaggaaaatttaccctaagaccgaaaaaggaaactctcccaaaaggaaaaagaaaaaataatccctagagaaaagaggaaattctcctaaagagaaaagggaaattatcctagagaaaagaggaaattgtcccaagggaaagaggaaaagagaaagtcaaatccttgagtaaatagaaaaatcgatttatggtcttcatgacatttacgatttgtccattacatgatattgactcgtaacttcttcattgtagatgtcgaagctcacaaacctcatgtacgctgcactcttggcatctggtgacaattaccttcaatgggcattggacaccaagattgagctgaagtctaaaggactcgccgagtgcattgtggaaggcaatgagaaaacggagaggtcaaagtatcaggcgatttcaatcatacgccatcacatagcagagagtttgaaaaatcagtaccttacggtcgaggatccacttgagctttggcttgagttgaagaatcgatacgaccaccaaaggaccatacaactacccaaagcccaacatgattggcttaacctcaggatccaggattacaaatccgtggaggagtataactccgagctctttaagattgtctctatcctgaggctgtgtggtgagaaggtgactgagaatgacatgctcgagaaaactttctcgacctttcacgcgaacaacgtactgcttcagcaacagtaccgtgcgaaagggtttacgacttacacaagtctagcctcctgcttgcttcttgctgagaagaacaatgagctgttactcatgaacagcgcattgagacctcccggatccacagctgtgcccgaagccaatagagccgaaatggccaaagcgcctaatgaacctcaggcgaccaaggaatccaactacgtccataggggtaaccctcacggccgtggccgtggcagaggacgtggtggaagaggtcgtggcaatttctacggccaaggaaaccactatggtggtagaggccgtgggaactatggccgaggtagaggtagaggccgcggtgttaacaaaccgcgaggaaaggctaagtccgtgtgctataggtgtggcatggacgaccattgggcaaagacgtgcagaacctctaagcacctcattgaggcatatcaagagatgataaagcaaaagggacccgaagccaatttggttcatctcgatggtgaaggggatttcgaccacgagaacgatgacctaatggattatgagacttcggacatcttgggaaaatatacatctagttttaaatttgctttgtttgctttgctttgtagtttgatctctattgcgcgatgtcttggatttatttattttaatggaattgctttgcttcattattgtttgctttatcgcctaagatatagttcttgtccacatttagaaatggccgaggacaaggacatactcatagtggacagtggctcaagccacacgatattgagagacaaaagatatttcataaatctaacattgagaaatgccaacattagcactatagcgggtatagcgagtctcattgagggctacggccaagcccatgtactcttaccaaacggcacacatcttgaaataagtgatgccttgtattcacccagctcaaagaggagcttgttaagtttcaaagacatacgtcttaatggtttccacgttgaaacaaagggtgaagggaatagagaattcctacatgtaacagagatcgcccaaggacataagagagtcctagaaactatacccgcattgtccactggtctttatcatactaagatcaacatgatcgaagctaatttggcaatgaacaaagagttcatagaggagttcactttgtggcatgaccggcttggccatccgggtcataacatgatgcgaaagctaatgataagttcaaaggggcacaccctaaaagaaaagagagttatcccaaagaagctcacgtgtgctgcatgtgcacaagggaaactcataataaggccattaccggcaaaggtcaataaagagaccataaactttctagaaaggatacaaggggacatatgtggaccaatacacccaccatgtgggacgtttcgatacttcatggtcctcattgacgcatcgacaagatggtcacatgtttgtctgttgtcctctaggaaccaagcatttgctaggttactgacccagatcattcgtctgagagcccattttccagattttccgcttaagactatacgtctagataatgccggtgaattcacgtcccaagcgtttaatggctactgtatgtccatgggggtaagtgtggaacatcccgtggcacatgtacatacacagaacggattagcggaatccttcattaaacgaatccaattgatagctcgaccattactgatgaggtcgaggctcccagtagccgcttggggacatgctgtattacatgcatcggaacttattcgtatcaggccatctagtgaacacaaatattcaccatcccaattgttaacgggtcatgagccagacatatcccatctaaggatattcgggtgtgccgtatatgtaccaattgctccaccacagagaacaaagatgggacctcagaggaggatgggaatatatgttggatttgattctcccaccattataaagtatctagagccaacaacgggtgatctatttaaggctagatacgcggattgtcacttcagtgaatccgagtttcctgcgttaggtggtgagacgaacaagctgggtaaagaaataaaagagatagcatggaatcaaacatccttgaattggcaagatcctcggactctgatgtgcgattcagaggtccataaaattatacatttgcaaaagctagctaatgagttgccagattcctttgctgacccaaagagagttgtgaaatcgtacataccagcttgtaatgcaccagtacgtattgatatccagaagggaatcaatgtaattgctaccaagtctaacccacagaagaaacgaggtagaccagtaggttccaaagataaaaatcctcgaaagactaagaaaggtgcaatgggaaccgaggtaaaggaaaccatagacatggccgcggcaagtcctaaggaaccaccaaatgagatttgggacgccgaagctcatggtcctgaaggaattgataatgatgagatctcaataaactacatcatgtctggaataaaatggaaccgaaaagaagtcgacgtcgacgagaaatttgcatatgaggtagcatatgagataaatgaggatcatgaacccacgtctatcatagagtgcactcaaaggtcagattggctaaagtggaaagaagccattgacgtggagttaggttcattaaagagaagagatgtgtttggtccaatattgaggacaccatctaatataaagccagtaggacacaagtgggtctttgtgaggaagagaaatgagaaaaacgaaatcgtgaggcataaggcacggcttgtggcacaaggattctcacaaagaccgggagtagattatgaagagacatactcccctgtggtggatgcaacgacttttagatatctaataagtctggcaataagagagaaccttgacttacggttaatggatgttgtaaccgcatatctatatggtccactggataatgagatatatatgagattaccagagggtattgagctcaaaggaaaagataagaatgggtctcgagaccaatactgcataaggctgaacagatcgctttatgggctgaaacaaagtgggcgcatgtggtacaataggttaagtgagtacttagtcaaagagggctataaaaatgaccccatcagtccatgtatcttcatcaagaagtttgctagcaaaggatttgtgataatagcagtatatgtggatgatttgaacatcctgggaacctctggggaaatcgcccaaacagtcgaatatctaaagaaagaattcgaaatgaaagacctaggcaagacaaagttctgtttgggattgcagcttgagtacgtagataaaggaatccttgtgcatcaaagggcatatacagaaacagtactcaagagatttaatatggacaaggcccacccattgaccagcccaatgcaggtgaggagcctaggattggatagtgatccattcggtccaaagaaggatgatgaagaaattctcggtcctgaagtgtcatatctcagtgccataggagcgttgatgtacctgtctagccacactagaccggacatatgttttgccgtgaacctcctctctagattcagttcttgtccgaccaagaggcactgggaaggaatcaagcatttgcttcgatacctacaagggacaattgattttggtttatattatactaaccataacaaagaaggtttagttggttttgctgatgcaggatatctttcagatccgcacaatggaaagtcacaaacgggttatgtgtttactcatggtggaaccgcaatatcctggagatcaatgaagcagaccatcgcggccacttcctctaaccaggcggagatattggcgatgcacgaggccagccgcgagtgtgtgtggttgaggtcaatgactcaacacatacgagccgattgtggaatggtcgaagccaaggagccgactatcatatacgaggacaacgcggcatgcattgctcagctcaaggaaggatacatcaagggagatcggacgaagcacattctgcccaagttcttctacacgcatgacctacagaaggatggtgaagttaaggtggtacaagtacgttcaagcgacaattcagccgatctattcaccaaggcattaccagcaacgacattgaggaagctcgcgcatcagattgggatgcggaggcttaaggacttacagggatgaccaattcagggggagtaatgcgtgctgtactctttttcctcactatggttttgtcccgaaagggttttcctagtaaggttttaatgaggcagcatccccctagcgcattactgagatctcgtaggcatctacacggttatgtcatccaagggggag

>ATCOPIA27_LTR

tgttgtaaatcacttgttggatggactccataaccgtggcccatgtccttggaggataggtccgtcagcccattggtgtgacggcccatcgaccagtcggcccaccagtccaccggtccatcggcccgtcggtctgtcggcccggcctatggagagaagccacagttggtccggcccgtaggtccggcccatggagactagacataggtcggtcatatacttaagtagttatgggcttttgtaacctagcccattagacatgtaacctcctcaagtatgtatatatatgtgatgtaacctcattatgaatcgataagaaacaagttagttcttcaca

>ATCOPIA28_IN

cgttatcagcacgagctgcctaagctaatagatactaaaaaaaaatttctgataaaaaaccatatttattatcgactcatgattcataatatcaatctcatatcatttttcgatttaaaaccatgatggcttgaatccgattcatacgttaatcggttgatgatggcttgagtttcttctttattatttctatgccgccgtgtgcttcgtcaataatttaatacacccatacgtttgccgttcaaatcagtttttgataaatcgtatattatttattattattttcatcacgtcctgattttgataaattgtacaccacatgtttaatcgttatatatcatggtcgtcatctcgtttatcttgttgttatgataagtaaatatttttcatttttattaaatatgctatcttaaagaaaagtcaaacatgaatatacggttcatttcatttgccatcacatgtctaatattatatgaaaattaatttaataatctgatcaagttacacgatcgatgatgacttatgctcatttgataaatttattgcttcatatttttttatattattttattgactggtttagttggtacaattgtttgatttggtttgataaattgaaatcacaatctgttatttattgataatataaatatgacatcgttattcatttatttttatatacaattattgtaaaaatatatatatatatatatatatatatatatatattaattgtatcatttatgaaatttaattaaatttcttaatatttcagtgaatcatgtcaaaactcatgagccttgaatttggagctcttgatatcacaggagataactgcttggcatgggcactagatgctgaaattcatctagattcaaaggatctccttgatactattaaagatggtaatcaaactccaagcagagaaaaatcaaaagctatgatttttctgcgacaccatttccatgaggatctcaagaatgagtacctcagtgtgaaagatccacatatcctttggaacgatctaaaggataggtatgatcatcaaaagacagtgatcttacctaaggcccgatatgattggactcatctgagactccaggactataagtctgtaagtgagtacaactcagccttgttcaaaataacatccaaattggagttatgtggagagaaaatcacggatgcatataagttggagaaaacattctctacttttcatgctaataacattgtcctgcaaacacaataccgtgaaaagggattcgtgaagtattcccaacttatttcttgtctccttgtagctgaaaaaaaataatgagttgcttttgaaaaatcatggactacgcccatcaggttctgctccattccctgaagcgaatgtgacatcctatggtcaagaaagtggttacaaccgtggccgtggccgtggttatggtcgtgatcgtggtcgcggtcgtggtcgtgggcgtggatacactggaggacgaggcagtggagttcactttaaaaactcaaactctcacaagaagtgggaaaacaaagatggtaacaaacaagtgaaaacaccatatgcgaatatctgttaccgttgtggagcgaaagatcactggtctcgtacgtgacgtacaccaaagcatcttgttgagctctatcaacaatcagtcaaaggcaaggggaaggatgttgaaacaaatttggtgtatgaagacggagttgatgattttgactttgatgatttcactcacttggacattgacgattttcttcctgaaaaagagacaaagtgatcaaactacaactatttgaagaactatgttttatttctttatttattttttttttgctttcaattatttgatttttatttatttggctttcagttattttattttacgaattattattaaagttgatttattttaattattggattcattatcttatttatgatattaatttattttatttatttgaagaaacaatgaatggtggtgatatttgcttggcagacagtgcaactagtcatactattttaaaaaataagagatatttctcttctttgacaatgaaagaagcaagtgtaactaccgtaacaggtagtgcaaagataattgaaggctctggaagagcaaatatttcaatgcctatgggaacacaattagaaattgtggatgctttatactctcctaaatctcaaaggaacttattaagttttaaagacatccgcagaaatggatatcatattgagactatgagcgaaggtaatatcgaataccttcaaatcacaagtaatatccaaagctctaaaaatattctcgaaaaactacctgctttctccactggtttatattatactacaattaatacaattgaagtaaatgctacagtaaaccagaagtctacagaaaacattaaagtttggcatgaccggttaggccatcctggtacaataatgatgcgaaagataatcactaattcatgtggacattcattaaagaaccagcagattcgtcctaatgatttctcatgtgttgcttgttcacaaggcaagttaattatacggccttcaccaaaaagtgtgatcaaatcacatataccggctgcgaatgctccaataagaataaatatcccagaaggataaaatcaaactgcaaatgagtctaaggcacgtttaaagcgtggtagacctattggttccaaagataaaaatcctcgaaaaagaaaaggggcagaaattggcatagataaaacagaggaaataattacaaatgaagaatctccagaagagaccttagacatgacacagaaagaaattcaggtacctgacaatgaagagatctcaattagttatgtcatgtctggattaaaatggaaccgaaagcaaatcgacgtcgatgatatttttgcatgcaatgtagcagttgatgttatggatgaggatcatgaaccaacatctattgaagagtgtacacaaagaagtgattggccaaaatggaatgaagcaatagatgcagaattaaaatctctggcaaagagagaagtgtttggatcagtagtccgtatacccaaaggtgtaaaaccagtaggacataaatgggtctttgtacgaaagaagaatgcaaatggtgaaattgtgaggcataaagcacgtttagtcgcacaaggattctcacaaagaccaggaattgactatgaagatacatactctcctgtggtggatgcaactacattcagattccttattagtctggcagtaaaagaagggcttgatttacgtttgatggatgtagtaacagcctatttatacggtccactggaaaatgacatttatatgaaagtcccagaaggatttaaaatgcctgaagcagtaaaaacaaattctcgagaacaatactgcataaaattaaatcgatcactttatggattaaaacaatcagggcgtatgtggtataatcgtcttagtgagtacttgataaaagaaggctataaaaatgaccctattagtccatgcatctttataaaaagatttgagaaaggatttgttataatgcagtttatgttgatgatttaaatattatgggaactcctgcagagatttccttcacagtagaatatttaaagaaagaattcgagatgaaagatcttggaaaaacaaaattttgtttgggactacaaatcgaacacctaaagaatggaattctggtgcatcaagagacatatacagaaatgttctcaaaagattctatatggatggagcacaccccttgagtagcccaatggttgtaagatcacttgatgtggataaagatccattccgccctcgggaaagcaatgaagaagttcttagtcctgaagtaccatacttaagtgcaataggagcactgatgtatcttgctagtcatacaagacccgatatatcatttgcggtgaatttattagcaaggtttagctcatgtccaactcgaagacattggaatggaattaaacatatacttcgttacctccaaggtacgaaagacatgggtctgttttttcctaaccaatccaaggaagatttaattggttttgcagatgcaggatacttatctgatccccataatggtagatcgcaaacaggatatgtcttcacatgcgggggtacagctatttcttggcgctctatgaagcaaactattgcagccacatcttccaatcatgcagagattttagcaattcatgaagctagccgcgagtgtgtatggctaagatccgtagttcaccatatacaagaagattgtggtatgtatgcagggaaaaagactccaacaattatgtatgaagacaatgcagcatgcatcgcacaactcaaagacggatacatcaaaggcgatagaacaaagcacatcctaccaaaattcttctacacacacgatctacaaaagagcggcgatgtacgagttctacagatccgttcgaatgataatctggctgacttattcaccaaggcgctacctattgctactttcaagaagttagtctatcgtatcggattacgccgtctcaaagatctcgacgaatatactcataagggcgagtaattgtatgttgcactctttttcccttaaccatggtttttcccattgggttttcctggtaaagtttttaatgaggcaacatttcaaatactttacgagttatgatactataatggtcatcagggggag

>ATCOPIA28_LTR

tgttatgaatattagatggatgcccattataaggcccatcttctagatccttctagaatagaatattctagaattatcctgtagatgctttgtgaaaccctaaccttatactcctttataaaggacctctcattcattggaataatacacacaaatcttcctcttcttcttcttttctaaca

>ATCOPIA29_IN

cgttatcagcgcgctttaaaagaaacaatgaatggtggagaaatatgcttggcagacaatgcaactaatcatactattttaaaaaataagagatatttctcttttttgacaatgaaagaagcaagtgtaactatcgtaacaggtagtgcaaagataattgaaggctttggaagagcaaatatagcaatgcctatgggaacaaaattagagattgtggaggccttatactctcctaaatctcaaaggaacttattaagttttaaagacatctgcagaaatggatatcatattgagactataagcgaaggtagtatcgaataccttcgaatcacaagtaatatccaagaatctcaaaatattcaggaaaaactacctgctttctccactggtttatattatactacaattaatacaattgaagtaaatgctacagtaaaccagaactctacagaaaacattaaagtttggcatgaccggtaggccatcccagtacaataatgatgcgaaagataatcacgaattcatgtggacattcatgaaagaaccaatagattcgtcctaatgatttttcatgtgttggttgttcacaaggcaagttgattatacggccttcaccagctaagataaatttcgaatcaatcaattttttggaatgaatacaaggagatatttgtgggccgattaacccatcatgtggaactttataatcctttatggttttaattgactcatctagaagatggtcgcatgtatccctattatcaactcgaaacctggcgtttgcaagattgcttgctcagttgattagaatacgagcatattttccagactttcctttaacaaaaatgtgtcttgataatgttggtgaattcacatctcaagcttttaatgactattgtatgtctattaggataagtgttgaacatcctgtagcacatgttcacacacaaaatggtttatcagaatcactaatcaagtgtcttcaattgattgctagaccattacaaatgaaatcaaaactcccaatatctgcttggggacatgctatattacatgtagcatcattgattcgcatcaggccaacaagtaatcataagttctctccatctcaactggttctcggtgaggaaccaaatatttctcatctcaaagtttttggattgtgctctatatgttccaattgctccaccacagccatataagatgggacctcaaagaacgttgggagtatatgttggttatgattccccatcaataattaagtatctcaatctatctataggagatttattcaaagctcgattcgtagactgtcattttaatgagtctgttttcctaacattagggggagaaaataaatagtcgggaaaagatatagtttggaatgaattatcaatgtcttatctttatcctcggactaaagagtgtgaattagaagtccaaaagataattcatttacagagtttagcaaatcagctgccagatgcattcattgacccaaaaagtgtgatcaaatcacatataccggctgcaaatgctccaataagaataaatatcctagaaggacaaaatcaaactgtaaatgagtctaagacatgtttaaagcgtggtagacctattggttccaaagataaaaatcctcgaaaaagaaaaggggcaaaaattggcataggtaaaacagaggatataattacaaatgaagaatctccagaagagagcttagacatgacacataaagaaattcaggtacctgacaatgaagagatctcaattacttatgtcatgtctggaataaaatggaaccaaaagcaaatcgatatccatgatatttttgcatgcaatgtagcagttgatgttatggatgaggatcatgaaccaacatctattgaagagtgtacacaaataagtgattggccaaaatggaatgaagcaatagatgctgaattaaaatttcttgcaaagagagaagtgtttagaccagtagtccgtacatccaaaggtgtaaaaccagttggacataaatgagtttttgtactaaagaagaatgcaaatggtgaaattgtgaggcgtttagtcgcacaaggattctcacaaagaccatgaattgactatgaagagacatggtgacttgatttacgtttgatggatgttgtcacaacctatttatacggtctactagaaaatgacatttatatgaaagtccgagaaggattaaaaatgcctcaagcagtaaattcaaattctcgagaacaatactgcataaaattaaattgatccctttatggattaaaagaatcagggcgtatgtggtataatcgccttagtgagaacttgctaaaagaaggctacaaaaatgaccctgttagtccatgcatctttataaaaattttgggaaaggatttgttataattgcagtttatgttgataatttaaatattatgggaactattgcagagatctcattcactgtagaatatttaaagaaagaattcgagatgaaagatcttggaaaaacgaaattttgtttaggactacaaatcgaacacctaaagaacgtaattctggagtatcaagagacatataccaaaaatgttctcaaaagattctatatggatggagcataccccttgagtagtccaatggttgtaagataacttgatgtggataaacatccattccgtccttgggaaaacaatgaagaagttcttagtcctgaaggaccatacttaagtgcaatatgagcactgatgtatcttgctagtcatataagaccttatatatcatttgcggtgaatttattagcaaggttcagctcatgtccaacttgaaggcattggaatggaattatacatatactttgttacctccaaggtacgaaagacatgggtctgtattttcctaacaaatccaagtagcgagtgcgagtgtgtatggctaagatccatagttcaccatatacaagaagattgtggtttgtatgcagggaaaaaggctccaacaattatgtacgaagacaaatgcagcatgcatcccacaactcaaagacggatacattaaaggtgatagaacaaatgcagcatgcatcccacattcttcttcacacacgatctacagaagagcggcgatgtatgagttctaccgatccgttcgaacgataatctggctgacctattcaccaaggctctacctactgctactttcaagaatttcgtctatcgtatcggattacgccgtctcaaagatctcgacgtatatactcataagggggagtaatggcgtgttgcactctttttcccttaatcatggttttcccaatgggatttcttggtaaggtttttaatgagatatcatttcagatgcactacgagctatggtactataatggtcatcaagggggag

>ATCOPIA29_LTR

tgttacaaataattgatggatgcccattataaggcctatcttctagaattctctagaattatttgtatctagaattttattttgtcaccaagtaaaattcttcacttgtactcctatataaaggaccctttattcattggaataatacactcattttgtacaatctctttctctagttacaaca

>ATCOPIA2_IN

atggtatcaagagcttagatccataaaaaaataaaaaaaattaaaaaaaaatttttcctctttcttcgttctctctccttcaatggctactcacgctgaagaaatcgttctcgtcaacaccaacatcctcaacgtgaacatgagtaacgttaccaaactcaccagcactaactacctcatgtggagccgccaggttcatgcgctcttcgatggctatgaactcgccggttttctcgatggctctacacccatgccgcctgcaaccattggcacagatgcggttcctcgtgtcaatcccgactacactcgctggagaagacaagacaagctcatctacagtgcaatccttggagccatctctatgtccgtacaacctgcggtatctcgagccactacagcggctcagatctgggagacacttcgcaagatctatgcgaatccaagctatggtcatgtcactcaactccgtacgtagctgaagcaatggacgaagggtgccaaaaccatcgatgactatatgcaaggattcatcactagattcgatcaacttgcgcttcttggaaaacctatggaccatgacgagcaagtagaacgtgtgctggaaaatctgcctgatgattacaaaccagtgatagatcaaattgccgccaaagataccccaccgtctctcacagagatccatgagaggctgataaatcgagagagcaaacttcttgctttgaactcggcagaggtcgtaccaatcactgccaatgtcgttactcaccgcaacaccaacaccaaccgtaatcagaacaatcgtggagacaaccgcaactacaacaacaacaacaatcgctcaaactcttggcagccatcgtcttctggctctcgatccgataatcgccagcccaaaccctacttgggccgatgccaaatatgtagcgtgcaaggacacagtgccaaacgctgccctcaacttcatcaattccaatcaaccaccaatcaacagcaatcgacttctccgttcactccatggcagccccgcgccaatctcgctgtcaactctccatacaatgccaacaactggcttcttgacagcggagcaacccaccatatcacgtcggacttcaacaatctgtctttccatcaaccttacaccggtggtgatgatgttatgattgccgatggatccacaattcccataacacacactggttctgcctctctacctactagttctcgctcactagatcttaacaaagttctatatgttccaaatatacacaaaaacttaatctctgtgtatcgcttatgcaacactaatcgtgtctctgtggaattctttcctgcatcctttcaggtgaaggatctcaacacgggggtcccattactccaaggcaaaactaaagatgagttgtatgagtggcctattgcatcgtctcaagcggtatctatgtttgcgtctccatgctctaaagctactcattcttcatggcattcccgtctcggccatccttcattggcgattttaaattcagttatttccaatcattcacttccagttttaaacccctctcataaacttctttcttgctcagattgttttatcaataaaagtcataaagtgcctttttcaaattcaacaatcacttcgtctaaaccattggaatacatttattcggatgtgtggagttccccaattctttctattgataattatcgctattatgtcatctttgttgatcatttcacccgttatacatggttatatccattaaaacagaaatctcaagtgaaagatacgttcattatcttcaagagtcttgtggaaaatcgttttcaaacaaggattggtacactttattcagataatgggggcgaatttgtggtcttacgggattatttatcgcaacacgggatctctcatttcacctctccaccgcatactccagaacacaatggtctatcagaaagaaagcatcgtcacatagttgagatgggtctcaccttactctctcatgcctctgttcccaagacgtactggccgtatgctttttctgtggcagtttaccttataaatcggcttcccacaccgctacttcagcttcaatcgccgtttcagaaactttttggacaaccgccgaattatgagaagctcaaagtttttggatgtgcctgttatccatggttaagaccgtacaatcgtcacaagctagaagacaaatcaaaacaatgtgcattcatgggttattctcttactcaaagtgcttacttgtgtcttcacattcccactggcagactctacacatctcggcacgtgcagtttgacgaacggtgctttcccttctctacaacaaattttggtgtatccacgtcacaagaacagcgttctgattcagctccgaattggccatctcatacaactctacccacaacaccgttagttttgccggctcccccgtgtttgggtcctcaccttgatacgtcgcctcgtccaccgtcgtcgccgtctccactatgcaccactcaggtatcgtcttctaacttaccttcctcttcgatttcttctccctcttcctctgagcccactgctccaagtcataatgggccgcaacccacggcccagccacatcaaacacaaaactcaaattcaaatagcccaattttaaataacccaaatccaaacagcccaagtccaaatagcccaaatcaaaatagcccattaccacaaagcccaatctcatcaccccacataccaacaccttcgacgagtatctctgaaccaaactcgccgtcctcctcatcaacctctacgcctccgttacctcctgtgttacctgctccaccaattattcaagtcaacgctcaagctccggtcaatactcattcaatggctacaagggcaaaggatggcattagaaaacccaaccaaaagtactcttacgccacgtcactggctgcaaattcagaaccacgcacagcgatacaggcaatgaaagatgatcgttggagacaagcaatgggatcagaaataaatgctcaaatcggcaatcatacatgggatcttgtaccgcctccaccaccatctgtcacgatcgttggttgtcgttggatattcactaagaaattcaattcggatggctcactaaatcggtataaggcaaggcttgtcgccaaaggttacaatcaaagaccgggtcttgattatgcggaaacctttagtcctgtaatcaaatcaacttccattcgtatcgtcttgggtgtggctgttgatcgatcttggcctatccgtcaattggatgtcaataatgcatttctacagggtacacttaccgacgaagtatacatgtcgcaaccaccgggttttgttgataaagatcgtcctgattatgtctgccgcctcaggaaagctatttacgggctgaaacaagctcctagagcatggtatgttgaacttcggacttatttactaacggtgggttttgtgaattccatctcagatacgtcactttttgtgttgcaacgaggtcgatcaattatctatatgcttgtctatgttgacgacatattgatcacagggaatgacacggtcttgttaaaacatactctcgatgcattgtctcagcgtttttctgtcaaagaacatgaggatctacattattttctgggtattgaagcaaaacgagtcccacaaggtcttcacctcagccaacgtcggtacacacttgatcttctcgctcgcaccaatatgctaaccgcgaagccggtcgcaacaccaatggcaacatcaccaaagttgacattgcactccggtaccaaactgcctgacccgacagaatacagaggcatagtgggcagtttacaatatctagcattcactcgtcctgatctctcttatgcggtcaaccgtctgtcacaatatatgcatatgccgacagatgaccattggaatgccctgaaacgtgttctgcgatacttagctggaactccggatcatggcatatttcttaagaaaggcaacacattatcattacacgcatactctgatgcggattgggccggcgataccgatgactatgtctccactaatggctacattgtttatctcggtcatcatcccatctcttggtcctctaaaaagcagaaaggtgttgtgcgctcatctaccgaagctgaatacaggtcagtggcaaacacttcatctgagctacaatggatttgttccttattgacagaacttggtattcaattgtcacatccaccggtcatctattgtgacaatgtgggagcaacttatctatgtgccaatccggtgtttcattctcggatgaaacatatcgcgttggattatcacttcatccgtaaccaagttcaatctggtgcacttcgtgttgtccatgtctccactcatgatcaattagcagacactcttacaaagcctctgtcccgcgtcgcttttcagaacttctctcgcaagattggagtcatcaaagtccctccatcttgtgggggcg

>ATCOPIA2_LTR

tattgaggatatgattgatatcctagattgctactgtcatggatacacatatgtaaagtcgtatatattctcattcattacgttgatgtaccctagctactcactcttgtatatatattgtgattctcactatgtaatgaaatcatctcttctataaacatctata

>ATCOPIA30_IN

agttatcagctaaaaggtagatattctcatttttttttatagaactatgtttatttatcgactcatggttcatagtatttatttttctattattgaaacaaacaatatgagccgatcgataattatgatatagattggtaaaagcatgttttcaatgattcagtaaagaagataatcatgcttaccatttatttatctaattattatagcatgattcaatgattctataaagaaaatcgtcaaaaaaaaagaatcatgtttgttgattgttaatttatgtgaaatctattataataatataaaatatattatttaatttgatattttatctagttaatattgcaactattaaatgcccggtggtgaatgatgcaagcatgcattagagcatcattattggtaggtttttaagaaattcttagcattaactttgacaaaaattaaatgaataattggtttaaattctaagaaccagctcttatataagagccaaagaactagttcttagcattaattcctagtatttttctatcttcttcgttttggatagttgcatattcaatatagttcagttaatattttattatctaaaattcttttacgattgattcaatattatttagttaatactttatttttgataattcatatttcacaaccatgatcgtcgtacatatattattacttttgatattgacttttcatatgtagatagatatctaatattctgatttccatatatttaatacttgattgttgattttcatatatttaatatttgattgtcctattacttttttttaacaaaaagcgttagtcaacccaattctacgttttttaattttctatgaaactttccttgcaaagtaatactttattattataagataattataatatcgttcagaaggaattgtctgtagtaaaatcgtctttgtaacagttctcataatttgcaataaaaataaatctaagacgaaaaaaaagataattgtaatattttatttgttaatgtattttgaaaattaacacatatttttttatttttgaaatctagtcatatttaatcaatttttttaatattttagtaaaccatgtcaaaacttatgaatctttaatttggaactcttgatatcacaggagataactatttggcattggtactgaatgctgaaatttatctagattcaaaagatctccttgataccatcaaagatgggaatcaaactcaaagtaaagaaaaattagaagctatgatttttctgcgataccatctccatgaggatctcaagaatgagtatttcagtgtgaaagatccgcagatcatttggaacaatctaaagcacagttatgatcatgagaagactatgatattatctaaggtccgatatgattggactcatctgagactccaagactttaaatatgtaagttagtacaactcagccttattcaaaataactttaaattggaattatgtggagagaaaatgatggatgcagataagttggagtagacattctctactttttaatgctaataacattgtcttgcagacacagtacggtgaaaagggattcgtgaagtattcccaacttatttcttgtctacttgtagctgagaaaaacaatgagttacttttgaaaaaccatggactatgcccatcaggttttgctttgttctctgaaacgaatgtgacatgctatggtcaaaaaagtggttacaaccgtggtcgtggccgtgtatacactagaggactaggcagtggagttcattttaagaactcaaactctcataagaagtgggaaaacaatgggacctcaaagaaggttgggagtatatgttggttatgatttcccatcaataaaagtatctcgagccatccacatgggatttattcaaaagctcgattcgcatactgtcattgtaatgagtctgtttttccaacattagagggataaataaacagtaagaaaaagatatactttggaatgaattatcaatgtcttatcttgatcctcggactaaagagtgtgaattagaagtccaaaagttaattcatttacagagcttagcaaatcagttgccctatgcatttactgacccaaaagtgtgatcaaatcacatataccggttgcaaatgatccaataagaataaatatctcctaaggacaaaaacacgtttaaagcgtggtaaacctattggctcaaaaataaaaatccttaaaaaagaaaagggacagaaattggcataggtaaaatagaaaaaataattacaaatgaggaatctcccgaagagactttagacatgacacagaaagaaattcagttacctgacaattaagagatctcaactggttatgtcatgtctagaagaaaatagaactgaaagcaaatcgacgtcgatgatattttgcttgcaacatagcagttgatgttatggatgaggatcatgaaccaacatctattgaagagtgtacacaaagaagtgatttgccagaatggaatgaagcaatagatgcagaattaaaatttcttgcaaagagagaagtgtttggaccagtagtctgtacacccgaaagtgtaaaaccagtgggacataaatgggtctttgtacgaaagaagaatgaaaacggtgaaattgtaaggcataaagtcgcacaaggattcccacaaagacccggaattgactatgaagagacatactctcttgtggtggatgcaactacattttggttccttattagtctagcagtaaaagaaggacttgatttatgtttgatggatgtagtcacaacctatttatacggtccactgaaaaatgacatttatatgaaaatcccagaatgatttaaaatgtcagaagcagtacaatcaaattctcgagaacaatactgcataaaattaaatcgatccctttataaattaaaacaatcatggtgtatgtggtataatcgccttagtgacttgctaaaagaatatacaaaaaaagactatgttagtccatgcatctttataaacagatttgggaaatgatttgttataattgtagtttatgttgatgatttaaatattatgggaacttctgcagagatctccttcacagtagaatatttaaagaaagaatttgagaagagagattttggaaaaacaaaattttgtttgggactacaaattgaacacctaaataatgtaattctggtgcatcaagagacatatacagaaaatgttctcaaaagattctacatggatggagcagtccccttgagtagcccaatggttgtaaggtcacttgatgtggataaagatccattatgtcctcgggaaaacaatgaaggagttcttagtcctgaaatatcatacttaagtgcaagttcatgtccaactcgaagacattggaatggaattaaacatatacttcgttatctccaatgtaagaaagacatgagtctgctttttcctaaccaatttagaagatttaattggttttgcagatgcaggatacttatctgatctccataaggttttgcagatgcaggatatttatctgatctccataatggtagatcgtaaacagaatatgtcttcacatgtattgctatttattggcgttctatgaagaaaactgttgcagccacatcttctaatcattcagagattttagcaattcatgaaactagccgcaagatttttagcgtgtatggctaagatccatagttaccatatacaaaaagattgtggtatctatgcaaggaaaaaagctccaacaattatgtacaaagacaatgcagcatgcatcgcacaactcaaaaacatatacatcaaaggcgatagaacaaaggacatcttaccaaaattcttcttcacac

>ATCOPIA30_LTR

tgttctaaataatagatggatgcccattataagattcattttatagaagtctctaaaattacttgtacatagggttttactttgtcacccaagtaaaacccttcccctccacattgatataaaataccctctattcattagaataatacacacgtttcttcgatctctatctctatttacaaca

>ATCOPIA31A_IN

ataagtggtatcagagcttgtcacgagcttggtacgagagagaaaaacatcatgagtgaaaaagagtctgtgatcataccaaaattcgacggtgactatgagcattgggctatgctcatggagaatctgataagatcaaaggagtggtgggatatcatcgagacgggaatcccaaggacggagagaaacgtgattctcaccggaccgcagaggacggagctggcggagaagacggtgaaagatcacaaagtgaagaactatctatttgcgtcaatagataaaacaatcctcaagacaatcttgcagaaggaaacgtctaaggatttgtgggagtccatgaagaggaaataccaaggaaacgatcgagttcaaagcgcacaactgcagagattacgcaggagctttgagattcttgagatgaagctgggagaaacaatcaccgggtatttctctagagtgatggaagtcaccaatgatatgagaaatctgagtgaagacatgccagattctaaggtggtagagaagatcttgcgaactctggtggagaaattcacatacgttgtatgtgccatagaagaatcaaatgacatcaagatgatgacagtagatggtctgcaaagctcgttgatggttcatgagcagaatttaagaagacatgatgtagaagataaagtcttaaaagctgagacacaatggagaccggatggtggaagaggtagaggaggaagtccaaaccgaggaagaggaagaggaggttatcaaggaagaggacgtggccatgtgaatagagacacggtggagtgtttcaaatgtcacaaaatgggacacttcaaggcagaatgtccaagctgggagaaagaagcaaattacatagagatggaggaagatttgcttctcatggcgcatgtagaacatattggtgaagaagagaaacatgtttggtttctcgattccggatgcagcaatcacatgtgtggtgtcaaagaatggttcatcgagttggaccatgacttcaagcagaatgtaaggctgggagatgaccggaaaatgactgtggaaggaagaggaaagctgagacttgaagttgacggaagaaaccaagtaatctcggacgtgtactttgttccggggctgaagaacaatctccttagtgttgggcagctgcaacaaaaggggttgagatttataattgaggacgatgtgtgtgaagtgtggcataagttggaacagagaatggtgatgcactcaaccatgacaaagaatcgcatgtttgctattgttgcaactgttagagaggctagagagactgaaggaaaaaggtgtcttcaagtgatcgaagctcaagacaatttatggcacaagagattcgggcatcttaatcaccatggcttaagatcacttgcagaaaaggaaatggtaaagggtttaccaaagctcactcatggagacaaagatgtgacatgcgaaatttgtctaaagggaaaacagatcagagagtctattccgaaagagagcatgtggaaatcgactcaggtcctgcagcttgttcacacagacatatgtggaccaataaaccctgtatcggccagcgggaaaaggtatatccttaactttattgatgatttcagtagaaagtgttggacctatctgttgtccgagaagtcggaagcatttcaggccttcaaggaatttaaagctgaaactgaaagagaatctggtcaaaaggtggtctgtttgagatcagatagaggaggagagtataactcaaaggagtttgaggagtactgcaaagaatttggaatcaagagacagctcacagcagcttacacgccacaacagaacggcgtcgcagagagaaagaatcggagtgtgatgaacatgacgcgatgtatgctgatggagatgtttgtaccaagaaagttctggcctgaagcggttcagtacgcagtgtacatactgaatcgaagtccgtcaaaggctctaaaggagataacaccagaagagaagtggagtaattggaagccatcggtagagcacttgagaatttttggttgcttagcatatgctttggtaccgtaccagagaagaatcaagcttgatgagaagagtatcaagtgtgtaatgtttggagtaagcaaggaatctaaggcgtatcgtttatatgatcctgctacaggaaaaatcttgattagcagagatgttcactttgatgaaaccagaggctgggaatgggaagataaattgctggaacaagagcttgtttgggaagattctgttaaagaacctgcaggggaagagggtacagaagcagatcaacctgagcaacaagaacaagcggaatcagaagaagaagtagtagaagaagaagctcatcaaaatcaaaatttgccgtctgttgaaacaggagctgctagacagagacaaccacctatttggatgaaagattatgtcatcggaaacgcaaaggttctaatagcagaagaagaagatgagttgtttgcgatgtttgttggaccagaagatccaggaaactttgaagaagcggttcagatggaggtatggagaaaggctatggaagctgagatcgcctcaataaaagaaaataacacatgggaactggttgagctgccggaagaaggaaaagtgattgggttgaagtggatcttcaaaaccaagttcaatgagaaaggagaaatcgacaaatttaaggcaagactggtcgcaaaagggtaccatcagagatacggagtcgactttcatgaggtatttgcgccagtggctaagtaggacacaatccggttaatactcgccttagcagcagagagagattggagagtctttcaacttgatgtaaagagtgccttccttcatggagacttgaaggaggacgttttcgttgagcaacctcagggattcaaagttgaagaagaatccaacaaggtatacaagcttaagaaagccctatatggactaaaacaggccccgagagcatggtatagccgcatagagggttactttatcaaggaagggtttgaaaagtgctactgtgagcatactctgttcgtgaagcaggagagaagtgatgttttggttgtcagtgtttatgtggatgatctcatttacacaggaagttcaatggatatgattgagaagttcaagacatcgatgatggaggaattctcaatgactgacttgggaagaatgaaatatttcttgggagtggaagttattcaagatgaaacaggtattttcattaaccaaaggaaatatgctgcagagattctcaaaacgtatggaatggaagattgcaacccggtcaagaatccaattgtgccaggacagaaattaaccaaggaaggagctggtgaactagtagattcaaccaagtataaacaactcattgggagcttgaggtacctaaccactacacgaccagacttgatttattctgtaaatctagtcagtaggtacatggaaagtccaactgagctgcacatgcttgcggtgaagagaatcttgagatatgtgaagggaactcaaggttacggaattcagtataaacgtgggagagttgcagagctggtcgggtttgttgacagtgattacgctggtgatgttgatgacaggaagagtacgtccgggtttgtgttcatgttaggaggaggagcaattgcttgggcatcaaagaaacagcccatcgtgactttatcaacaacagaagctgagttcgtgtcggctgcttttggtgcttgtgaagcgatatggctcagaaatgtgttagaagagattggttgcagacaagaagaaggtacacttgtgttctgtgacaacagttccactataaagttgtcaagaaatcctgtgcttcacggaaggagcaaacacattcatgtgaggtatcattttcttagagaactggtgaaagaaggaactatcaggcttgattactgtgctactgcagaccaaattgcagacatcatgaccaaggctgtgaaacgagacgtgttcgaggatcttcgtgaaagaatgggagtaaggattagagaagagtaaactgaagagaaggagtttcagtttaagggagggaa

>ATCOPIA31A_LTR

tgaagaataaaggtccaatgagtttgggcctgtggagtttgggcctacgggtttagttacgcatctttcgttttcaagttgctgtgttttgttctgttttcgtttttgaataagtcagctcctttgtcggagaaagactcgggcgtttagctagtagcttaagtttaggagctgcagtttgattatcttgtaaggtctatttaaagactatcactgaagtcaattgaagtaagaagttttcagtctctgtttcttgcatactctgtttcaaattgagattagaaaaca

>ATCOPIA31_IN

gtggtatcagagcttgtcacgagcttggtacaaaagagaaacaccatgagtgaaaaggagtcggtgatcataccaaaattcgatggtgattatgagcattgggctatgctcatggagaatctgataagatcaaaggagtggtgggatatcattgagacgggaattccaaggccggagagaaacgtgatcctcaccggagcgcaaagaacagagttggcggagaagacggtgaaggaccacaaagtgaagaactatctatttgcatcaatagataagacgattctcaagacgattctgcagaaggagacgtcgaaggacttgtgggagtcaatgaaaaggaaatatcaagggaatgatagggttcaaagcgctcaactgcagaggttacggagaagctttgaagttcttgagatgaagattggagaaacaatcacgggttacttctcaagagtgatggaaatcactaatgatatgcggaatctgggagaagacatgccagactccaaagtagtggagaaaattctgagaacattggtcgaaaaattcacatacgttgtgtgtgctatagaagagtctaacaacatcaaggagcttacggtggatgggcttcaaagttctttaatggtgcatgagcagaacttgagtagacatgatgtggaagagagagtgttaaaagctgaaactcaatggagaccggatggaggaagaggcagaggaggaagtccaagccgaggaagaggtagagggggttaccaaggaagagggcgtggctatgtgaacagagacacggtagagtgtttcaagtgccacaagatgggacattacaaggcggagtgtccaagttgggagaaagaagcgaattatgttgagatggaggaagatctgcttctcatggctcatgtagaacagattggtgatgaagagaaacaaatttggttcttggactcgggatgcagtaaccacatgtgcggtacaagagagtggttcctcgagttggatagtggttttaagcagaacgtgagactaggagatgatcgaagaatggcggtagaaggaaagggaaagctgagacttgaagttgatggaagaattcaagtgatctcagatgtgtactttgtgccagggttgaagaataacctctttagcgtgggtcaactacaacaaaaagggttaagattcatcattgaaggtgatgtgtgtgaagtgtggcacaaaacagagaagagaatggtgatgcactcaacgatgacaaaaaatcgcatgtttgtggtttttgcagctgtgaaaaagtcaaaggagactgaagaaacaagatgccttcaagtgatcggcaaagctaacaatatgtggcacaagagatttggacatctcaaccatcaaggcttgagatcgctagctgaaaaagagatggtaaaaggcttaccaaagtttgatcttggagaagaagaagcagtttgcgatatatgcttaaagggtaagcaaatccgagagtcaattccaaaggagagtgcgtggaaatcaactcaggttctgcagcttgttcacacagacatttgtggaccgataaatcctgcatcaaccagtgggaaaaggtacattttgaactttatagatgactttagccggaagtgttggacttatttgttgtcggagaagtcagaaacatttcagttcttcaaggaatttaaggctgaagtcgaaagagaatcaggaaaaaaattggtatgtttgcggtcagacagaggaggagaatataattcgagggagtttgacgagtactgcaaggaatttggaatcaagagacaactcaccgcagcatatacgccacaacaaaacggggtcgcagaaagaaagaatcggagcgtgatgaacatgacgcggtgtatgttaatggagatgtcagtgccaagaaagttctggcctgaagctgttcagtatgcagtgtatatactgaatcggagtccgtcaaaggctttaaatgacataactccggaagagaagtggagtagctggaaaccatcagttgagcatctaagaatatttggcagcctagcatatgctcttgtcccgtaccagaaaagaatcaagcttgatgagaaaagcatcaaatgtgtgatgttcggtgtaagcaaggaatccaaagcctatcgtctctatgaccctgcaacaggaaaaatattgattagtcgggatgttcagtttgacgaagaaagaggctgggaatgggaagataagtcgctggaggaagaacttgtgtgggacaattctgatcatgaacctgcaggagaagagggtccagaaataaaccacaacgggcaacaagatcaagaggaaacagaagaagaagaagaaacggtagcagaaacggttcatcaaaatctcccggctgttggaacaggaggtgttagacagagacaacaaccagtttggatgaaagattatgttgttggaaacgcccgggtgctcataacacaagatgaagaagatgaagttcttgcgttgttcattggaccaggtgatccggtttgctttgaagaagcggctcaattagaggtgtggagaaaggcgatggaagcagagataacctccatagaagagaataacacatgggagttagttgaattacctgaagaagcaaaggtgattgggctgaagtggattttcaagactaaattcaatgagaaaggagaagttgataagtttaaagcaagactggttgcaaagggctatcatcagagatatggagtggatttctatgaggtatttgcaccagtagctaagtgggatactatcagattgattcttggcttagctgcagagaaaggatggagtgtttttcaactagatgtgaaaagtgcgtttttgcatggagacctaaaggaagatgtgtttgttgagcagcctaagggattcgaagtggaagaagaatccagcaaggtgtacaaattgaagaaggccttatatggcctaaaacaagctccaagagcttggtacagccgtatagaagaattctttggtaaagaagggttcgaaaagtgctactgtgaacatacactgttcgtgaagaaagagagaagtgacttcttggtggtaagtgtgtatgtggatgatcttatttacacaggaagctcgatggagatgattgaagggtttaaaaattctatgatggaggaatttgctatgactgatcttggaaagatgaagtacttcttgggagttgaggtcatacaagatgaaagaggtattttcatcaaccaaagaaagtatgctgcagaaatcattaagaagtatggaatggagggatgtaattcagtcaagaatccaattgtgcccggacagaagttaacaaaagctggagctggtgatgctgttgatccaactgagtttaagcaactcattgggagcttaaggtacctaaccaccactcgaccagacttgattttctctgtaaatctagttagtaggtacatggaaagtccaaatgagcaacacttgttggctgtgaagagaatcttgaggtacgttcaaggtacgttggatctcgggattcaatatgaacgtggtggtgctacagagttggttggcttcgttgatagtgattatgccggtgacgtcgatgacagaaagagcacctctggttatgtgttcatgttaggtggaggagctatagcttgggcatctaagaagcaaccaattgtgacattatcaacaacggaagcagagtttgtgtcagcttcttatggtgcgtgtcaagcggtatggctcagaaacgttttggaggagattggctgcagacaagaaggaggcacacttgtgttctgtgataacagctccaccataaagttgtcaaagaatcctgtactacatggaagaagtaagcacattcatgtgaggtatcattttctcagagagttggtgaaggaaggaactattagacttgactattgtactactacagaccaagttgcagacatcatgactaaggctgtgaaacgtgaagtgtttgaagagcttcggggaagaatgggagtaagaagaagagaagaataaaccgaaaagtgtgttttcggtttaagggagggat

>ATCOPIA31_LTR

tgaagaataaagcccaatcggtttggatttgggcctgcatctttgtgttggttctgcaactttattggtttttctagttttgaataagtctccacctttgtaggagaaagaatcgtgacattagtttagttgcgagagtttcgtagcttcagttgctttctttgtaaagtctatttattgactaatctgaattcaatgcaagtaagagtttttcagtatctgttcttgcaaatctttgtttttccttaaagtgagatttgataaaacaaca

>ATCOPIA32B_IN

gattggtatcagagcaccatgttggtgttctttgtggttctgaaaagggtttaagatcttgaaccatgtctacaactagaatagaagttgagaagtttgatggtcgttgtgattacacgatgtggaaggagaactgttggctcatatggatatattgggtctgtgtactgctctcaaagaatcagagagcaagggtgaggagttatcaggtccagacgaatttgatgaagactataaagagaagctcgaaaagttcaaagctttagaagaaaagaagagaaaggctaggagcgccatagttcttagtgtcattgatagagtcctaaggaagataaagaaggaatcgacagccgctgcaatgttgttagccttggataggctatacatgtcgaaagcctttccaaataggatctatctgaagcagaagctttacagctacaagatgtctgaaaatctgtcagtagaaggtaatatcgatgaattcctttagattattacaaacttagaggacatgaatgttaatatctctgatgaggatcaagctattctgctgttaacatcgcttccaaaagcttttgatctgcttaaggatactcagaaatatagctcgggtaagtctatattaaccttagatgaggttgttgcagccatctactctaaagagttggagttagggtctgtaaagaagagtatcaaggttcaggctgaaggtctttttgttaaagacaagaatgaggctaaaggaaagggtgaacaaaagggaaaggggaaaggaaagaagggcaaatccaagaagaaacccgggtgctggatttatggtgaagaaggacacttcagagcttcctgtccaaaccagaacaagccacagttcaagcagagtcaagtagcaaagggagaatccttaggaggaaaaggaaacatagctgaagcagctggttattatgtatctgaggcgttgtcatcaactgatatccgtttagaagatgaatggatactagatacaggctgtagctatcacatgacctacaagagggaatggtttgaagagttcgatgaagaagcaggaggttgtgtcagaatgggaaacaagactgtttctcgggtaaaaggaattggaaccatcagggtcaaaaatgatgatggtctgtctgtggttctcacaaatgtgagatatattcctgatatggatagaaatctgctatctttggggacttttgaaaaggcaggttacaagtttgagtcagaaaatggaatcttaagcattaaaactggaagtcaagtgcgacttactggaagaaggtatgacactctttatttgcttaactggaaacctgtagctagtgaatcacttgatgtagttaggagaatggatgatactgtgttatggcatagaagtttatgtcatatgagtcgaaagaacatggagattttggtcaagagaggacttctagatggtaagaaagtctcagttctcgacacctgcgaggactgcatttatggtaaagccaagagaattggtttcaattcggctcatcatggtaccactaagaagttagaatatgttcattcagacctttggggagctccattagttccattctcattagggaagtgccaatacttcatgtcagttattgatgattacactagaaaggtgtgggtttattttctgaaaaccaaagatgaagccttcgagaagtttgttgattgggtcagtttggttgagaatcagagtgacaatagagtgaagactcttagaacagacaatggtctgtaacaagttgtttgatggtttttgtgaatcaaaagggattcaaaggcacagaacgtgtgcttacacacctcaacagaatggggttgctgaacgcatgaatagaaccatcatggagaaagtgagaagcatgctgagtgattgaggtctacctatgaagttttgggcagaggcaactcacactgcagtacttctcatcaataaaacaccatcatcagctcttaattttgagattcctgacaagaagtggtcaggaaaaccaccggtttacagttatctgagaaggtatggatgtatagctttcattcactctgatgatggaaagcttgaaccaagagctaagaagggagtgtttattggttacctgttggagtaaaagggtataaagtgtgactgctggatgaaagaaagtgtgtggttagtagaaatgtcatctttcaagaaaatgttgtgtacaaggatctgatgctgacacgtgagaatgtgtctagtgaagaagatgatcaaactggatcatatctagatcttgaccttgaagcagaagatgatgttatctcaggtggagatcaagggatgtcttagacaactcctgtaccagaaagtccagtacacgctactccactcagtaaaactgcaaatgataatggtgtttctgaagtaaatcagtctcctccgagttatcatttagtaagagacagagacagaagagagattagagcaccaagacgttttgatgatgaagattactatgctgaggcattgtatacaactgaagatggtgatgtagttgagccggagagtttcaacgaggcaaaaatggactctaattggaacaaatggaagcttgctatgaatgacgagatcgattctcaagagaaaaacaacacgtggacagtagtcacgagacctgagaatcaaagaatcattggctgcaggtggatctacaagtataagcttgggattctgggtgtagaagaaccaagattcaaagcaagacttgttgccaaggggtattctcaacgagaaggaattgactatcatgagatctttgctcatgtggtaagacatgtatctataagggttttgctgactatagtatcacaagaggacttggaactagagcagttagatgtgaaaacagcttttctgcatggagagctgaaggagaagatctacatgtcacctccagaaggttatgagtcaatgttcaaagagaatcaggtatgtcttttgaataaagccctgtatggactgaagtaggcaccaaaacaatggaatgagaagtttgacaacttcatgaaagagattggttttgtgaaaagtcagtatgataactgtgcttacactaaggtgctgcagaacggttcaatgatgtatcttctcatctatgtggatgatatgcttgtagcagcaaaggataaagaagcaatagcctcttaaaagtctgagttgagcaagcgttttgagatgaaagatttgggggctgctaagaagatacttggtatggagatcaacagaaacagatcagagggtgctctatggctgtctcaggaaggttacttgaataagattcttgaaacttacaatatgtcagaaccaaagcctgcaatgactccacttggagctcattttaagtttcaagctgcaacagaacagaaactggtcagagatgaagactttatgaagtttgttccctactcaagcgctgtgggaagcatcatgtatgccatgattgggacacgtcctgaccttgcttatccagtgggtatcgtcagccgcttcatgagtaaaccgagtaaagatcattgggttggagtgaaatgggtgttgagatacattaaagatactttgaaaacaagactgtgttacaagaaaagctcaagtttcaagattgtgggctattgtgatgcaaattatgttgatgaccttgataagagaagatcaatcactggtcttggttttacacttggtggaaacaccataagttggaagtcaagtctacaaagagttgttgctcagtcaacaacagaatcagagtacatgtcacttaatgaagcagtgaacgaggcagtttggttaaaaggtttgttgaaagacttcaggtatgatcagaagagtgtggaggtcttctgtgattctcagagggctattccactctcgaagaacaatgtgcatcatgaaagaaccaagcacattgatgttaagttccattacatccgagagatcatatcagatggaactgttgaggtgttgaagataactacagagaagaacccagaggatatattcaccaaagttttggcagtgagtaagtttcaaatagctttgaacttgctccgtgtcaagtctgagtagtaaactcggagaatccgagactagaatccagaggctagaatcaggtactctttctctctcttgtaactctaaacagtttgaatgtttgtggcttatgctatctagtggagatt

>ATCOPIA32B_LTR

tgtgaagactgatagcttgaagcacaaggtgatattcaaatgtttggccttaaccttaagacttaacccaaggtaaagtaactcaaccttggttagtctacttttgggaccacaacaaagcaaacggctatgaaggagcttgcctcgcaatggcatcggtttgctttaaggacagtttgtccatttgtgtgtgcaaagagaggaggttaaagaaggagacaacaatcgctttcaagctggcagttttggcgttttaaggagcgtaggctgcggcttgttatttgctgaggtgaagccctaatctactttgaagtataaataggtgcttaagtcattgagagagtgtgcgagacattttagagagtttgggaggtgttaagagagaaagctattcatagtataaacttgtattaactgttctttgtgggataacagagtagtgcagagcaatcctaagtactagtgaagggtattaggtgtattgcttgtagactcttgttgtaaacactataagattctagtggattccgaggagagtctcggcccagacgtacctaccctatgggagtgaactgggttaccaaattctcgtgtgtgttctctattttgtttctctatttcttgcaaagtcttgttcttcttacaaacattcttgaacgagtttctgaagtacatcactcatctctgaagtagttctgttctaggatctgttttgagtcactgagtaaagtaaagtcgacgtctttagttctgtcgtttcttaaca

>ATCOPIA32_IN

gattggtatcagagctgtaggttattgcttgtggttctgtaaagtctcacaatcattgaaccatgtctacggctaggatagaggttgagaagtttgatggtcgtggtgattacacgatgtggaaagagaagctgttggctcatatggatatattgggtctaaatactgctctcaaggagtcggagagcaccggtgagaagaaatcagttctggatgaatctgatgaagactatgaggaaaagcttgaaaagttcgaagctttagaagagaagaagaagaaggctaggagcgccattgttcttagcgtcacagatagggttctaaggaagataaagaaagaatcgacagctgctgctatgttgttagccttggacaagctatacatgtcgaaagctcttccaaacaggatctatccgaagcagaagctatacagcttcaagatgtctgaaaatctgtcggttgaaggtaacattgatgagtttcttcaaattattacagatttagagaacatgaatgttattatctcggatgaggatcaagctatcttactgctaactgcacttccaaaggcatttgatcagctgaaagatactttgaaatatagctctggtaagtctatattgactctagatgaggttgctgcagctatttactcaaaagaattggagttaggctcagtgaagaaaagtatcaaggttcaggctgagggtctttatgttaaagacaagaatgagaataaagggaagggtgaacaaaagggaaagggtaaaggaaagaagggcaagtccaagaagaaaccagggtgttggacttgtggtgaagaaggccacttcagatcttcttgtcctaaccagaacaaacctcagttcaagcagagtcaagtagtgaagggagagtcctcaggaggtaaaggaaatctggctgaagcagctggatattacgtgtctgaagctttgtcatcaactgaggtccatttagaagatgaatggatattagacacgggttgcagctatcacatgacctataaaagggaatggtttcatgagttcaatgaggatgcaggcgggtcagttagaatgggaaacaagacagtgtctcgggtcagaggtgttggaaccatcagagtcaagaacagtgatggtttaactatcgttctcactaatgtgagatacattcctgacatggacaggaatctgctgtcattgggaacttttgagaaagctggttacaagtttgaatcagaagatgggatattgagaattaaagctggaaatcaagtgttattgactggaagaaggtatgacacgctttatttgcttaattggaaacctgtggcaagtgaatcacttgcagtagttaagagagcagatgatacagtcttgtggcatcagaggctatgtcacatgagtcaaaagaacatggaaattctagttagaaaggggtttctagataagaaaaaggtatcaagcttagatgtatgtgaagactgcatctatggtaaagccaagaggaagagtttcagtctggcacaccatgatactaaagagaagcttgagtacatccactctgacttgtggggagctccatttgttccgctctcactagggaagtgtcaatacttcatgtccattattgacgactttaccagaaaagtatgggtttattttatgaaaactaaggatgaagcattcgaaaagtttgttgaatgggtcaatctggtggagaatcagactgataggagagtgaagactctcagaacagacaatggtctcgaattctgtaacaagttgtttgatggcttctgtgaatcaatagggattcataggcatagaacttgtgcgtatacaccccaacagaacggtgttgcagagcgcatgaacaggacgatcatggagaaagtaaggagtatgctgagtgattcaggtctacctaagaggttttgggcggaggcaactcacacaacagtgttacttatcaacaagactccctcttcagctctaaattttgagattcctgacaagaagtggtctggaaatcctccagtttacagctatctgagaagatatggctgtgttgcgtttgttcacacagatgatgggaaactggaacctagagctaagaaaggagtgcttattggttatcctgttggagtaaaagggtacaaggtgtggatacttgatgagagaaagtgtgtggttagtagaaacatcatctttcaagagaatgctgtgtataaagatctgatgcagagacaagaaaacgtttctacagaagaagatgatcaaacaggatcttatctggagtttgatcttgaagcagaaagagatgttatctcaggtggagaccaagagatggttaacactatacctgcaccagaaagtccggttgtctctacccctacaactcaagacactaatgatgatgaagattctgatgtaaatcagtctcctttgagttatcatttagtaagggatagagacaaaagagagataagagccccaagacgtttcgatgatgaagattactatgctgaagcattgtacacaactgaagacggtgaagcagttgaacctgagaattaccgaaaggcaaagttagatgcaaactttgacaagtggaaactagctatggacgaagagatcgattctcaagagaagaacaacacatggacgattgttactagacctgagaatcagaggatcattggttgcagatggatttttaagtacaaacttgggattcttggagtagaagaaccaaggttcaaagcaagacttgttgcgaagggttatgcacaaaaggagggcattgactaccatgagatctttgctcctgtggtaaagcatgtatctataagggtgttgctttcgatagtagctcaagaagacttagagctggagcagttagacgtgaaaactgccttccttcatggggagcttaaagagaagatctacatgtcacctccagagggatatgagtcaatgttcaaagccaatgaggtatgtcttcttaataaagctctatatggactgaagcaggcacctaaacagtggaatgagaagtttgacaacttcatgaaggaaatctgttttgtgaaaagtgcttatgacagttgcgcttatacaaaggtgttgcctgatggttcagtgatgtatcttctcatttacgtggatgatatactggtagcatctaagaacaaagaagctattacagccttgaaggctaatttgggtatgcgttttgagatgaaggatctaggagctgctaagaagattttgggtatggaaatcatcagagacaggacattgggtgttttatggctgtctcaagagggttacttgaataagattcttgaaacctacaatatggctgaagcaaagcctgcaatgactccactgggagctcatttcaagttccaagctgcaacagaacagaagctgatcagagatgaagactttatgaagtctgtcccctactcaagtgcagtggggagcattatgtatgccatgcttggcacacgtcctgatctagcttatccagtgggtatcattagccgttttatgagtcaacctatcaaggagcattggcttggagtaaaatgggtgctaagatacattaaaggtactcttaaaacaagactgtgttacaagaaaagctcaagtttcagtattgtgggctactgtgatgcagactatgctgcagaccttgacaagagaagatcaatcactggtttagtgtttacacttggtggaaacacaataagctggaagtcaggcttgcaacgagttgttgctcagtcaacaactgaatcagagtacatgtcactcacggaggctgttaaagaagcaatttggttgaagggcttacttaaagacttcggttatgaacagaagagtgttgagatcttctgtgactcacagagtgctattgcactctcgaagaacaatgtgcatcacgagagaacgaagcacattgatgttaagtaccatttcatcagagaaatcatttcagatggtactgtggaggtgttgaagatatctactgagaagaatccggcagatatattcaccaaggtattggctgtgagcaagtttcaggctgctttgaacttgctccgagtcaagtctgagtaatggactcaggatccgagactggaatccagaaactagaatcaggtactctctctctcttttctaaactctagatgttgtgaatgtttactgcttatgctatcaggtggagatt

>ATCOPIA32_LTR

tgtggagatgatagcttgtagcacaagaacattcaaatgtttgactcaacctgaagagtgaaactaaccaaggttgattaagtcgaaccatggttgagttacttttgggaccagtttcttgaggcaacggctatgaaggagacagcctggaaatagccatgttttgcgcttaaggacattttgtcttgtgtgtccaaaagagaaacaggatcagagtggctgcaacaatcgttttcaaagttgttagagttggcgtttagcaaacgcaggcggctacttgtttgttgctgaagtgaagccctaattgttacttggagtataaaataggtgcttgtgtgataagagagagagtgtgagagaaataaaagaagtgaggtgttcttgagagaaaagcattacattgtgtaaacttgtattgagttgttctctatggattacaagggtagtgtagagtgacctaagtactagtgaagggtattaggttgtcacttgtaaactcttgttgtaaagaaacaccatctgatttctagtggattcccgaggcaagtctcggcccagacgtagctacccttacgggagtgaactggattaacaaatcctgtgttcttcgtttttgttttctcttctgttgctagtcttgttcttctgaaacacacacacactcagttctctgttccatcattctgttctctgttttagtgcctctgttctgtctttgttgagtgtctctctagaggaagtcgacaccttttagagtaagagatcttaaca

>ATCOPIA33_IN

aattggtatcagagctctggttcatcaataatctgaagatcggttataagaatgacggattcgaaagtaaagattgctcaattcgatggatagggtgacttctccctctggagaactcggatgttcgctcacctcagagtgttggggctaaagactgctctggtggagcaaccaccacataaacctctcacggaagaagaagaaggagatcctgcaaagaagaagaagtggatcgaggaggaagaggctagaatagagcgagatgagaaggcgatggacattatattcatcaatgtcgcagataaggttctaagatcaagaaaacattcaaaatctgcagcggaagcttggggaacgcttgagagattgtatttggtaaagattttaccaaatcgtgtatatcttcaactaaaggtttacaactacatgatgcaagaatcgaagactcttgaggaaaactttgatgagttcttaaagatgattttagatctgaataatcttcagattcaagtcccaaatgaagttcaagcaatcttgatcctgagtgctcttccagagaagtatgatatgttgaaaaaaacatcgaaatatggtagagaaggtataaggctggatgatgtaataagtgttgctaagtcaaaggagctggagctgagggatagcttaggtgattcaagaccggttggtgaaggcttgtatgtgtgaggaagaccacatgcaaagggtaatgatcaaatgtttgggaaagagggcaagaaaatctgttggatctgcggtaaagagggacattttaaaagacagtgctataagtggattgagaggaacaaggctaattctcaacaaggagaatcagctctggttaaagatgatgcacaagatctgattggactagtcgcatctgaagtgaatctcactgagaatgcagatcaagatgagtggataatggacacaattgctcgtttcacatgactccaagaagagatgtgctcattgagtttcaggaaattggaacagggatggttagaatggcaaacaacttattcacagaggtcaatggtattggcaaagtgaggtttgtgaactcggatggtaccacctttatgcttcacgatgtcagatatatgccagggatgtcaaggaatctgatctccatgggaactctggactctaaaggatgtgagttcgaaggcaaaaacggtgtgttaaaggttatgaaaggggatatcacgtacatgaaaggcacaagaagagagtcttaatacattttacaagcaggagctaagaagtcagagtgtctggcacttgaagagaaacctgcagaagaagatctaacaaaactttggcacaatcgtcttaggcatgtgggacagaaagggatggatggcctagcagagaaagggtgttttggaaaagacaaagtatcaagtattaagttctgtgaggattgtgtgtttggaaaaacacacaaagttagcttcagctcagcacaacacgtcactaaagagaaactagactatgttcattcaaatttatggggctctccaaacgtcccattgagtcttggaaggtgtcaatacttcatctccttcactgatgattggtccaggaaggtctggatctatttcttgaggacaaaggatgaagcatttgacatgtttgttcaatggaagaagatggttgaaacacaaagtgaaaggaaagtaaagaagcttcgtacggataatggtcttgagttctgcaataatagatttgatactttctgcaagaaggaaggaatggtgagacacagaacctgtgcctatacacctcaacaaaatggaattgtagagagattaaatagaacgattatgaacaaagtcagaagcatgctcagtgagagcggattaggacaacagttttgggttgaggcagcttccacattagtgtacctaataaatagaacaccatcatctgctattgattttcacattccagaagagatgtggacttcaactgtgccagacttatcaggtttgcgaagatttagatgtctcgcttacattcactcaaatgatggtaagctaaatttgagggctaagaaaagaatctttaccggatatccagaaggagttaagggcttcagggtttggttgttagaagacaggaagtgcaccattagcagaaatgtggtgttcaaagaggatgtgttgtacaaggatatcatggctcagaaacagtcatgtatgatctctaaccctttaccaatgttatctgataaagctacttgtgatattgcaggaaataacaggtctgaagaggatatttcttaaggtggagctttaaagcaagatactgtgataaattcaggacaaagttcagtggaaatgtcaacaagagttgagaatcaaatgtcagggagctatcagatagctaaacacaggccaagaagacagatcatgagaccaaccagactagaagactaatacactgaagaagcagaattagatgagatagccggatacgcatacttcgtaacagaagacggtggcaagtctgagccctccagttttcaggaagcattaagagatgctaatagtgacaagtggttcacagctgcagatgaagagatagagtctctcatgaagaacaaaacttggtcactagtagacagagatgaaaaacagaagcctataggctgcaagtgggtcttcaagagaaaacctggaatagtgggtgtggaaggtcctcgatttaaagcaagagtagtagctaaaggttactcacagaaagaaggggttgactatcaggagattttttcacctgtggtgaaacatgtgtcaattcgttttctgctcagtattattgctcatttggatatggaactccaacagatggatgtcaagactgctaacttgcatggatacttataagagaccatttacatggatcagccagaaggttatgtagatgaaaaatatccaatgaaggtgtgtctgctacaaaggtcgctatatggtctcaagcagtcaccaaggcaatggaataatcgattcaacgagtttgtgtagtcacatggatatgaaaggagtcaatatgatagttgcgtttactctaagagattacagagtggagactatgtctatatgctgctatatgtcgacgatattttaatagcttcaaaggataagaatcatatagatgagttaaaaacacttctcaactcagaatttgaaatgaaagatctaggagaagctaagaagatccttggaatggaaatatcaagagatagacagaaaggtactctcactgtatctcaagatggatatctcctaaaggtccttggaacttatgggatggaccaatccaaaccaataggaacaccaatgggaatacacttcaagttgcgtgctgccacagatgaagaattaaggattcagtctgaatctatgaggggtgtgccatatcaaagtgcagttggaagcttaatgtatgcaatgataggtaccagaccagatctcgcatatcctgtgggattggtatgcaggtacatgagtaaaccactcaagcaacattggcaagcagttaagtggattctaaggtatatcagtgggtctatcaagaagaagctttgctataaaaacaaaggagattttgtgattgaagggttttgtgattcagactatactgcatgacaggagaagatctacatcaggtatagtttttacttaggaggcaacgttatcagctggaaatcaagtctgcaaaaggatgtggcattatcgtctactgaggctgaatacatggctctaacagaggcagctaaggaaacagtttggttactaggactgatgaatgaacttgggtttaagcaggattcagtaaatattcactgtgactctaagagtgcgattgctttggcaaggaacacagtgttccatgaaaggactaaacacatagataccaagtatcatttcataagagatttggtaagtgatggggttataaatctggttaagattgcaacagagtgtaatcctgcagatatctttactaagacagtaccagtgggtaagcttcaagaagcgttagagcgactcaggattacagaagcataatggagccataaggctcctcaacaagctgagtgggtttactcacgtgatctgaagcttagtgggtttaatcaaaaaggcttagaaggctgagtgagtttagtcaggtatacggagttggagtccaagttcggctgctaggtcgctgcattggggtcaaggtggagaac

>ATCOPIA33_LTR

tgagagttgtgacccaaatgcctttattctctaagtatcagagagtctctcaaagaaactgaaggttcaagtcttggggttaattatcacacgtgtggttaacaggtgagaagtcaacgaaggagtattagcgttatatctttagctcattctgtttcatcgttcattgagttgttgtaacaagataagagagagagagagatcctttgtagagagagaacgttatcagcgatctagtggattccggagaatctccggcgagacgtaaactaaccacatcagttagttgaactagttaaagcttggtgtcgtttcttcattcttaaacaaacacacgagagtctagagtgtttagtgagctaaacttgaagacgattcatcttgaatcgtaca

>ATCOPIA34_IN

aaactggtatcagagccaggttactttgttcttctttcgtcctgcgaattttttgcataagaagttttgtgcagaataaggttctggaaattccatgttttgtaatctggtgagaagatgtcttcgatgaatgtgaagattgataagttttctggaagaaacagtttcagtctctggcaaattaagatgcaagccctgttaaaacaacaaggcttgtgggcgccgctgtctaatgacagtaaagggaagagcgatgatgctgagatggatgtcatggacaagaaggcacactccacaatcatgttgtgtcttgaagacgaagtcatcattgaggtttcaagagaaaccacttctgtcaatctgtggaagaagttagagaatttgtatatgacaaagtttctacagaacaagctgcttctgaaacgacgcctgtttgcattaaggatgcaagaaggtacaccactcaaagatcacttagaaaagttgaagtcagtattactagaactgcggaatattgattttaaagttgaagatgaagatgctgctttgcttctgttggtacctttaccactatcatatgagaactttgttgaatcatttatagtgaataaagttacagttactttggaagaagtcagatctacgcttcacaggatctactgctatagcagaaaatgacactaagtctcaatatgatgttgctcttgtggctgctggaaacgcccacaactctgatgtgtgggttctggatacaaggggcatcatttcatatgtgtccaaggagagagtagttctcaacatatgtacatgtagaaaacggctgcatcaagatggctaacagttctgtctgttaggttgctggaatcggctctatacagatcatgacacatgatggcagattttgcacactgaacgatgtcagacatgttccgtcgacggagaaaactttgatatcactgagtctgcttgatagcaaaggtctaaaatattctggtggagacagaattttgcaggtctgtcaaggttgttataagtggtcctctttatatactacatgggttcactgttataggttcaatgaatgttgcatcttcagagattcacaaggaggatatgactaagctatggcctatgaggcttggtcatatgagtgaaagagggatgaaaattttgtccaaggaagatcttttgtgtggccatgaggtcaagagtcttgagttctgtgagcattgtgtgtatgggaagcttcaccgtagtaagttccctaaggctgttcacataaccaaaggtacattagattatattcactctgattgttggggtccagctcaagttgaatctctaggaggtcatagatattttgtgtcgatgattgatgattactctaggaagacttgggtgatcaagctaaaacacaaaagtgaagctttcaataacttcagggagtggaaaattttggtggaaaatcaaactggaaagaagatcaagaggctgcggacagacaattgtctggagttctgttcatcagagttcaatcagctgtgtaaggatgaaggggttgctcgtcatcacactgtcagagatacaccataccagaacgttgtagctgaacagatgaatcagactttattgaagagagctaagtgtatgctctctaatgcttgtttagaaaaaagattctaggctgaagcagtaaatatagcctgttacttgataaatcgtgggccgcacacatgaatcaagtgtaggacacctgcagagatgtggtctggtaaatctgctgattactctaatcttaagatttttggctgcacagtttataatcatgtaaatgaaggtaaattggagccaacagcaaggaagggagtatttctaggctatggagatggagtcaagggattcagggtttggtctccatcagagaagagagtgattttgagaaggaatgttgtctttgacaaagtttctatgctctgattttcagagaagtctacaactacagaagagaacagtagctttgataaataggtggagctgacagctaatcagaaagctgatttacagaaaccagaagacagtaaggggctacaggcagcagatgggtcacctgacactatcaagcaagaatcaaaaccttactgtatcgctcaaaaccgaacaaagaggattggagttggaccaccgcaaagatacggttatgaggatatggtaggattctgtcttgaggatatggtagggtatgctttacaggttgcagaagaggtagatactcatgagccagccacttatcgagaagttttttctgggattgaggctgagaaatggtttcctgcaatgagagatgagatggaatctcactgtaaaaatcagacatgggatttggtcaaacgaccaccgggaagaaagattgttacttgcaagtggatcttcaagataaaggaagggatatcaccagtagaaggggttaagtacaaggctcgagttgttgcgagaggtttcagccaaagagaaggggtagactacaatgagattttctcacatgtgatcagacacacttcaatcagaatgctgctagcaattattgcaaatcaggacttggaacttgaacaacttgatgttaaaactgcttttcttcatggtatattagcagaagaaatatacatgactcaatcaaatggtttccaagttcctagaaagggagatcacgtctgtaagttgaacaagtctttgtatggcctcaagcagtctccaggcagtggtacaagaggtttgacaactacatgatggagctgggctacaacaggagtccgtatgattgctgtgtttacactagcaagctgaaagatgaatcatacgtctatctggtgctctatgtagatgacatgttaataaatgccaaacagatgtctgatattcagagattgaaagatttgttgagtgctgagttggagatgaaggatctgggagcagcaaagaagattctagggatggagattctcaaagacagaagtcagaataagcttttcttgtcatagaaaggctacattcagaaggcgttggataggtttggtatgttatcagcaaaacctgttgatactccttttgctgctaatattcctctcaccatgtttgctccttagtctgaagaggacaaagagtatatgtctcgtgttccttacgctaatgctgtgggatgtttgatgtatgctattgtctgtacaagaccagatcttgcacctgtagtcagtgatgtcaataggttcatgggacaatcatgaaaagaacattggctggctgtgaaaaggattttcaagtacctgaaaggtacgtctgatgttggactcctatacagaggtgaggctctgagcctgattgcaggctattctaattcggattatgcaggagatatagacagcagaagatcaatgactggttatgtgtttgccttgggtaattctgtaattagctggaaagcaactctactgccaactgtgacgctgtcaaccacagaagcagagtatatggcttcaacagaagctgctaaagagggtatttggctgaaaggattggtcagtgatatccgtttgcatcaagatcaggctacaatgtattgtgacagtttgagtgcgatttctttagccaaagatcatgtccatcatgagcggaccaagcatattgatgtgagatatcattttctgagaaatgagaagaggtttcaagtgaagaaggtagggactgctgataaccctatagatatgttcactaagctggttccttagagcaagtttaaacattgtttagacttgctgaatgtctcaagctgttaatcgccctgaagggcaaacaggcccctaacggggcagcaagtccctggagggacatctggcctctgaataggcataaggctccttatgaagcatttggccccagtggagcgttctggtttctgatgaagcattttggtcttggtaggcgttctggcttctgaataagtattctggctcagtgaagcatctgacttgacgaaggagttggcttgcaggggcatctggtccctgatggaatatctgaggcaaatgagaattttggtacatatctctgctacttttggttggtacgtctagcattgggataatgcatatagtacaaccgtctggtagcttaatctagtacatctatctaagggcgaagtctggtacaacactgtgagcagtgtgtcaggtacagctgtttggaggattcaagtcacgttggagattt

>ATCOPIA34_LTR

tgttcgatatgacttgaattggaatgtacccttaactttaggccaaatcagtttagggttaattagggcttcttttgtataagtactcttcttaacctagcctccctgtattctgtactctgttttctcaagtaataaatctgtctctttgcccgtggacgtagccaacagaaagtgttggtgaacaacgttaaatctgtgtcgtgttctttacgcttttttgtcttgctcacatctgttataaca

>ATCOPIA35_IN

gttcttaatctttcttgaaaactagatttggtatcacagcaagatcgatcttgcagaatgagtgaaataattcccgcagcttcacccgcagctaaagcaatgagagaagtgagtagatcttcttcgattcaatgtcctatgctaaacgcaacgaattacacatgatgggccatgaggatgaggagagcgcttaaggttcacaagacttgggatgtgatagagaaggttacggagccgtttgatgaagacaagagtgatgttgcatgcaccttgttgtttcaatccattcctgaatcataagcattacggattggagaacttgagacagcaaaagaagtatgggattcgatcaaagcaagagatgtgggggtagagagagtgaacgatgctagattgcagaccctaatggctgaatttcatcgactgaagatgaaggattctcataaaatagatgactttgcaggcatgttgtctgagatctcagtaaaatcggctgctttgggtgtaattattgaagaatcaagacttgttaaaaagtttcttaagagtgttcctcggaagagatacattcacattgtggctgctttagaacaagtcttggatcttaatgccataacttttgatgacatagttggtcgtttaaaggtgtatgaggaacgagtttacgacgaagacgatcctgaagaagaccaaagcaagcttttgtattcaagtatggactcaagtaaacgtgaatcagagggtgaggaggaagaggatcctataggggaagaggacgaggacggaatggtggcagagatgcatcacatataacatgttttagatgtgataagatcggccactatgcttcgtattgtcctgatcgattgttgcaattgcaggaaactcaagaaaatgacaataaggagacccaagtcgcggatgagcttatgatgcatgaagtggtttatctaaatgaacaaagttgtatgccaagcagttttgaaacaaacgttgatggagagaatgtttggtacctggacaacggagctagtaaccacatgacaggagatcgtcgatactttgacaagatggaccattctattaccgggaaaatacgctttggagatgattcacgcatcgacattaagggaaagggcacaatagctttcatagacttgaatggtaaaccaagagtaatgttagatgtctattttattcctgaactcaagagcaacataattagccttggacaagctactgagtcaggatgtgaaataaaattgaaggatggatatctaactatgcatgatcaagaaggaaagctgttggtaaaagctgagaggtctaggaatagtctatacaaagtaaagatgggcttaagaaaggaagcatgtttatacctaacaagtacaagtatgtcaagtctgtggcacgcaagaatgggacacgtgagtctcatcacactaaagtctatgattgataaggaattgatccaaggagctccaagtttagtgattgagtcggaggtttgtggttcttgcttacttggaaagcagacaaggcagtcattcccacaagcgaccgtctttagagcaaccaagaagcttgagctgatacacggagatctgtgtggtccaataacaccaaaaacaagtgttggaaatcgttatatctttgtactcattgatgattattctcgttacatgtggacagtgctgttaaaggagaagggagatgctttttttaagtttaaaaacttcaaagctttggtagagaaagaatctggagagaagattcaaactttcataactgacagaggaggagagttcgtgtctggagagttcaattctttctgcgaaaggacagggataagaaggcatctcactgctccatatacaccacaacaaaacggtgaagtagagagacgtgacaggactctattggagatggcgagaagcatactgaagcatatgcacatgcctaattatttgtggggggaagccataagacactctacctatctaatcaacagagtcgctacaagagctcttaaggatagaaccccatatgaatgcttcagagagaagaaaccaatcgttgaccacatatgaatctttggttgtacggcatatgcaaagatagacaagcagtttttgaagaaattagatgataggtctcgagtactcattcatttagggacagaatcaggatcaaaagcttacagattactcgatccacaaacaaagaaagtagtggtcagtcgtgatgttgtctttgacgagacaaagggctggaactggacacaggatgactcaacacaagacggagaaggaagcttcaaagtcatgattgaagactttggtgatcatgaacttaaagagaaagaaggagatgcagacacagatcagatttctccagaacaaaatgatgattctaattctgagacaagtcaagaagcaatagaagaagcaacagagtcaagtgaaagtgtttctcctctgcaagcattaagaagatcaactagacaggttagtagaccaaaataccttgaggattacgtattgcttgcagaagaggagggtgagatgttacttttgactctaaacaacgagcctagatgtttttatgaagcaagagaacataaggagtggatacgcgcctgtgaagaagaaatcaactctattgagagactagagacatggaatctcgtgaacttaccagtttgagtgaagccgattggtctgaagtgggtattcaaacttaaaagaaactctgatggaagcataaacaaatacaagggtcgccttgttgctaaagggtatgtccagaagtatggagttgatttcgaagaagcctttgcacctgtagcacgaattgaaacaataagattgctcatcgacttagcagcagctcatagttgggagattcatcacctcgacgtcaaaactgccttcttacacgaagaactaaaggaaatagtttatgtcttacaactagagggctttgagaagtttggaaaagaggataaggtttataaacttaaaaaggctttgtatggattgagacaggccctaagagcttggaacaacaagctaaatcacattcttgttgaacttctttttaagaagtgctctaaggagccatcagtctaccgcaaagaagttaaaggtaatctcctcattgttgttgtgtacgtcgatgacttatttgtcacatggacaagcttagaattgatcaacaaattcaagagggagatgtctgctaagtttgagatgagtgatctcgggaagttgacttattatctggggatagaagtaactcaacacaatgaaggaataatattgtcataaagtcgatatgctctcaagattttagaagaagctgggatgaaagactgcaacctagcacacactcctatggaaattggtttgacttggaaaaatcagaaaatgaaaaggaagtgaatacaacagattttagaagaaacgttggttgtctcagatatctgctacatacaagacctgacttgtcgttttgtgtaggagttttgagtatatatatgcagagtcgcagagaatcacatgcggctgcgatgaaacaatgtttaaggtacttaaagggaactactacacttggtatatcgttcactcgaagcatggagataccaagactagtaggatacagtgatagcagccataatgtggatccagatgatgggagaagcacagtaggacatatattttatcttggcaaaggaccaatttcttggtgttctcagaaacaagacactgtggcgatgtcgtcttgtgaagcagagttcatggcaggaacagaggcaacaaaacaagcaatttggcttcaagatcttttgagtgagattacaggtacatcaagtgagagtgtaaccatacgcatcgacaacaaatcagctattgcccttacgaagaatccggtctttcatggtcgaagcaagcacatacataggagataccatttcattagataatgtgttgagaatggtcaaattgaagtggaacgtgtttcagagatgaacaaaaggcagacattttaacaaaggcacttggtcgatccaagttcaaagagatgagaagcttcattggagttcaagacttaaagggagaagagttcaagcttaagagggagaa

>ATCOPIA35_LTR

tgttggataagcttgagaaaagtaacttgaggagtaagttactaaagaagaggaaaaggagattaagctttattattctaggagatatctagaatatattgtttggagttatcttatgatattaggagttatctaattgttaagataaggttaaagctttgttatatagggaggtatcaatgttgtgatacaacttatgggatttgagagagattgagactttaggttttgagagagtttcctaaagataataaaagagagttattcttttatatacttgt

>ATCOPIA36_IN

tggtatcagagcttatcgattgagaggacaagagaaaacactaaaaagagagagaggcaacaaaagtgagatcgtatacgtctcagcaaaaagcgagacgatgcaaggctcctcacatcaagtcatccctatattcgatggggagaaatatgacttctggagcattaaaatggcaaccattttcaggaccagaaaactatggtccgtggttgaagaaggcgtaccggtcgaaccggtacaagcggaagaaactcctgaaactgcaagagcaaagacgctcagagaagaagcagtgacaaatgatacaatggctttgcaaatcttgcaaactgccgtaaccgatcaaatcttctcacggattgcagcggcatcatcatcaaaagaagcgtgggatgtgttgaaggacgaataccaaggatctccacaagtccgattggttaaacttcaatcgctgcgtagagagtacgagaacttgaagatgtatgacaatgacaacatcaagacattcaccgataagttaattgttctggaaattcaattgacttatcatggtgaaaagaagacaaacactcaactcatacagaagatactcatctctctcccagccaagttcgacagcattgttagtgttctagagcaaactcgcgacttggatgcactaacaatgtcagaacttcttggtatcttaaaagcacaagaagcgagagtaactgcaagagaagaaagcacaaaagagggagcattctatgttcggtccaaaggaagagaatccggtttcaaacaagacaacaccaacaaccgtgtcaatcaagacaagaaatggtgtggttttcacaagagtagcaaacacacagaagaagagtgtcgagagaaaccgaagaatgacgatcatggcaaaaataagagaagtaacatcaagtgttacaagtgtgggaagatcggccattacgcaaatgagtgtcgctcgaagaacaaagagagagctcatgtaactcttgaagaagaagacgtaaatgaagatcatatgttgtttagtgcaagtgaagaagaatcaacaacgttaagagaagatgtttggttggtagacagtggctgcacaaaccatatgacgaaagaagagagatacttttcaaacatcaacaaaagtatcaaagtcccgattagagtcagaaatggagatatagtcatgacagccggtaaaggagatattaccgtcatgacaaggcatggcaaaagaatcatcaagaatgttttcttagtgcctggtttggagaagaacctattgagcgttcctcaaatcatctcaagcggctattgggtacgttttcaagacaagagatgcatcatccaagatgcaaatggaaaggagatcatgaacattgagatgacggataagagcttcaagatcaagttaagttcagtcgaagaagaagccatgacagccaatgttcaaacagaggaaacatggcacaaaagacttggccatgtaagcaacaagcgcttgcaacaaatgcaagacaaggaattggtaaatgggttaccaagattcaaagtgacgaaggagacatgtaaagcgtgtaatcttgggaagcaatcacgcaagagctttccaaaagaatctcaaacgaagacaagagaaaagcttgagattgtgcatacggatgtgtgcggaccgatgcaacaccaatcgatcgatggaagtcgctactatgtgctgtttttggatgattatactcacatgtgttgggtatacttcctaaaacaaaagtcggagacgttcgcaacgttcaagaaattcaaagcgttggtggagaaacaatcaaactgttccatcaagacgttgagaccgatggaggtggtgagttcacttcacgagaattcaatcagttctgtgaagatgaaggaatcaataggcaagtgactttgccttactcacctcaacaaaatggtgcagcagaacgcaagaatagatcattggtggagatggcaagatcaatgcttgttgaacaagacttacccctcaagttatgggcagaagcggtatatacctcggcgtatttgcaaaatcgcttaccatcaaaagcaatagaagatgatgtgacgcctatggagaagtggtgtggacacaaaccaaacgtgtcacacttgaggatatttggaagcatatgttacgtgcatataccggatcagaaaagaagaaagctagacgctaaagcaaagtgtggtattctcatcggttatagcaatcaaaccaaaggctatagagttttcttactggaagatgagaaggtggaagtatcaagagatgttgttttccaagaagacaagaagtgggattgggataaacaagaagaagttaagaaaacttttgtcatgtccatcaacgacatacaagagtcaagagatcaacaagagacatccagccatgatttgtctcaaatcgatgatcatgctaacaatggagaaggagaaacctcaagtcatgtgttatcccaagtcaatgatcaagaagaacgagaaacttcagaatcaccaaagaaatacaagtccatgaaagagatcttggaaaaggcaccaagaatggagaatgatgaggcggctcaaggcatagaagcttgtcttgttgcaaatgaagaaccacagacttatgatgaagctcgtggtgataaagaatgggaagaagccatgaatgaggagataaaggtgatcgagaaaaacagaacatggaaactagtagacaaaccggagaagaagaatgtgataagcgtgaagtggatctataagatcaagacggatgcaagtggaaatcacgtcaagcacaaggcaagactagttgcgagaggtttttctcaagaatatggaatcgattatcttgagacattcgcccctgtttcaagatatgatacaatacgagctttacttgcgtatgcggctcaaatgaagtggagattgtatcaaatggatgtgaagtcggcgtttctaaacggagagcttgaagaagaagtttatgtcacacaaccacctgggtttgtgatagaaggcaaagaagagaaggtgttaaggctttacaaagccttatatggtcttaagcaagctccacgtgcatggtatgaaagaattgattcttacttcattcaaaatggttttgcaagaagtatgaatgatgcagccttgtacagcaagaagaaaggagaagacgtgttgatcgttagcttgtacgtcgatgatcttatcatcactggaaacaacactcatctcatcaacacattcaagaaaaacatgaaggacgagttcgagatgacagatctcggtttgctaaactacttccttggaatggaagtaaaccaagatgacagtggcatttttctttcacaagaaaaatatgcaaacaaacttattgataagtttgggatgaaagagagcaagagtgtaagcaccccacttacaccacaagggaaaagaaaaggagtagaaggtgacgacaaagaatttgcagatccaacaaagtaccggagaattgtcggaggattgctctatctatgtgcatcaagacctgatgtgatgtacgcaagttcttacctatcaaggtacatgtcatcaccaagcattcaacactatcaagaagccaaacgagtgttgagatacgtcaagggaacgtcaaattttggagtactctttacaagcaaagagacaccaagattagttggctactcggacagcgattggggtggttctctggaagacaagaaaagcaccacaggttatgtctttactcttggtttggctatgttttgttggcagtcttgtaagcagcaaacagtggctcaatcaacggcggaagcagagtacatagcagtgtgtgctgcgacaaaccaagccatatggttgcaaagactttttgaagacttcggcttaaagttcaaagaaggtattcccatcttatgtgacaacaagtcggcaattgcgattggaaggaatcctgtgcaacatagaagaacgaagcacatcgagatcaagtatcatttcgtgagagaagccgagcacaaaggactcattcaacttgagtattgcaaaggagaagatcaactcgcggatgtacttaccaaggcgttaagtgtttcaaggtttgaaggtcttagaaggaagcttggagtgaagccgagatatgattaagggggag

>ATCOPIA36_LTR

tgttgaagaataatcaaatctcgtataagtcaaagtcacattcgtataagttagttattaagttacgagtcgtgtctgttaacacagttgtgtctttatcaagagtcatgtcttttacacaagacaatgtttcatttcatataaatagatcgagtctctgtttcctgtaacacaattgagttttgtaatacaatcaagtcaataagattgattacattcatactctgttttttcgttcttactctgttcttctttcataacaaaacattaaca

>ATCOPIA37_IN

agtggtatcagagctccaggcttgtatagggttgttaaggagtttcagctaaggagtttcagatcaggggttacaagaagttttaaaagatgagtagtaacgctcgggttgaggttgacagatttgatggaaccggtgacttctctttgtggaaggtcagaatgttggctcattatggtgttttagggctaaaaggtatcttgaacgatgagcagctgctaagagatccacctgttatagaagaggaagctgcagtagctggaagagactatcacgtcggagattttgagctgccttccaatgttgatcttataaagtttgaaaagtcagagaaggcaaaagatcttatagtactaaacgtgggaaatcaagtactaagaaagatcaaaaactgtgagacagctgcagccatgtggtccacattaaagaggttatacatggagacttctctacctaatcgaatctacttacagcttaagttctatacttataagatgactgattctagaagtatagatgggaatgtggatgatttccttaagctagttactgatctaaacaacataggagtagatgtgacagaggaggttcaagctatactattgttgagttcattatatgaccggtatgatcagctcaaagagacgttaaagtatggcagagacactttgagcttgaatgaagtcattggagctgcaaaatcaaaggaaagagaactcacagaaagtggaaagctctcaaagtgaaggtctgtatgtggaacaagaggcagatcagagaagaggtttgaaaaaggaaaaggcaaaccatggagaggacgttctaagagcaaaggcagatcaaaatcaaggccaaagtacaataaaaacaacaagggttgtttcatctgtggtaaagaaggacattggaagagggagtgtcctgaaaagtcttcacaagccttcaagttcagcaaacattgcaacggagccaaaacagccactagtattaacagctagccctcaagacactaaggaagaatgggttatggattctggatgtagttttcacattacactagacaaggattctctgtttgacctacaagaatttgatggaggcaaagttttgatgggtaatatgactcacagtgaagtgaaagggattggtaagattaagatcttaaatccagatgattatgtggtcatattaacaaatgtgcggtacatacctacaattggagaagagcggctgcaagtatgaaggaaaagactttatagtcactttctacaaagatggacagaaagtcatttctggaaaatatcaagatgggctgtactatctagaaggaaaagtagtaaaaggagaagttgcagtggcaagaccagacattgacatgactagcttatggcattctaggctgggtcatatgagcttgaagaacatgaatgtgctggttgaagaaggttacttgtcaggaaaagaagtagacaagctggaattttgtgagagttgcgtacttggaaaatctcacaaacaaagtttcccaactgctaagcacactactaaaggtatattagattatatacattctgatctctcgggatctccttctactccagaaagtcttggtggttgtaggtactttgtgagcttcattgatgatttctcaaagaaagtatgggtgtattttctgaagaccaaggatgaggcatatcacaagttcagagaatggaaacaagcagttgagaatcaaacgggcaagaagattaagtatttaaggactgataatggtcttgaattctataatacacagtttgacaacttgtgtaaggaagatggaattaaaaggcacagaacgtgcacttatacaccgcagcaaaacggtgtatcggagaggatgaacaagaccattatggatatagttagaagtatgcttgctgagacaggaatgagtcaagagttttgggctgaggctacttctacggcagtctacttaatcaacagaacaccgaactcgtttattggtttcaagctacctgaggaagtgtggacaggtacaaaaccagatttaagtcatctcaggaggtttggatgttcagcctatgtccatgtcactcaagacaagacaagtccaagagcagtcaaaggagttttcatgggatatccttgtggtattaaaggatatagagtctggttaccgaaagaagggaaatgcactacaagtagaaatgtggtgttcaatgaaactgagttgtacaaggacacattaagtagtgcagatgaaaggaaagaggaagctgaaaaagagtacaagaaattgaagaaagctagaaaaagagtgtccttcagccatgatcttctcagaggtccttcaactagctgctgtgatttggatgactcatcttctcaaggtggagaaacctcctcatcttcatcagaaagttcagaaaacttagaggaatcagaaatgaatgaggaagttgttggatctgaaaatgagcagtctctggatgattacttattggcgagggacatgaaacgaagaagtaatatcaggcctccttccagatttgaagacgaagatttcgtagcttatgcattggcaacagcagaagacttggaagaagaagagcctaagtcttatgaagaagcattgaaaagtagtaagagaaagcagtgggaaaatgcaatgaaagaggagatggattctcataaaaagagtcacacttgggatttaattgagaaaccagaaaagcagaagctgattggatgcaagtggatttttaaattaaagccaggcataccaggagttgagaaacagagatacaaggctagacttgttgctaaaggtttctcgcagcaagaaggtattgattataatgaagtgttttctttagtggtgaagcatgtctccatcaggttaatgttgtcacttatggttaacatggattatgagctggagcaaatggacgttaaaacagcttttctgcatggtaatctagaggaaaggatcctaatgagtcagcctgaaggattcatacaagagggaaacgaaaataaagtttgtttgttgaggaaatccctatatggcctaaaacagtctcctagactttggaatcagaggtttgatgcattcatgaaggatcagaagtttgaaagaagctgttatgacccatgtgtctacatgagagacactcaaacagacaaagctatctacttgttgctctatgttgatgatatgttgatagcttcagggaatatggctatcatacaagagcttaaaaacaagctgaattgtgagtttgagatgaaagatttgggaaggcttcaagaattctgggtatggatatcataagagacagagaaaagggagaattaattctgtctcaaggaaactacttggagaaagtgttaaaacgtttggaatgcttgaagcaagaccagtgattacaccaactgctgctcacttcaaattcagaagtttgtcagaagaagaaaagaaaacagaagctgttcacatggaaagaattccatatgcaaatgttgtggggagtttaatgtacgccatggttggttcaaggccagatttagcatttgtagtgggttttatcagcagatttatgtcaagtccaggaagggagcattggtcagcagtaaaatgggtgttaaggtatttaaaaggtgcatacactcaaaacttgatttttaagaaggattctaagttctgcattgaaggattttcagattcagattatgcaacagacttggacaggagaagatcagtgacaggatatgtgtttaaagttggtggaaacacaatttcgtggaagtcaagtttacagtctgttgtggctctttcaaccacagaagcagagtatatggctcttactgaagcagtgaaagaggccatttggttgaaaggtttgtgttcagaattgggttttaaacaagataatgttatggttcattgtgattcgcagagtgctctagccctagcaaagaattcagttcatcacgaaagaaccgagcacatagacactaaattcacacaacagtcaatccgacagatttcttaactaagacggtacctggtcaaaagttccagctttgctacgagcttctcaacatccactgagatggagcagcggctacaagtaactccaacagtctcgcaaaacaggaagaaacaagatgagtcagttttgtgagaaatactcaagtgtcataattgagtaaggcattaaattggagttggtgttacctgttaagtgctggaactgcgtaggagttgcaagttccaggtggagtatctgactacacagtttcagaaagaaattgggttaggaaatctcataatgttgtgagattgaaaaattgctacaagagatggtgattaacggcaggtaattacgtgaaagctgttgttattgtgctgagaggaatatcagtttcaggtggagtcctctaagaagccagctgaaatcgttgggtgtggtgattcaactattaacaaagcagatcgagtcagatcacttggttaaaatttgaatcgcaaaatcagttggtgaggaagagcttacctaaggtcgaaattgattgggtattatctcatcgaagaagttgtggagttcgccagagaagttgtgccaatcacccgaattacattaaaaaaaaaaaaatcaagttttttcagattctctggaaatattaaaggatcctagtctctaagtgtgacaagaaagagattagagagtacctttgatgaacctggagttacaaaggaggctgtggtcagaatctaaaattgattccaatttttatcatcttctcttcatcacgggaaagagaggaagcttggaagagattggtgaagatgagaatgggagattatgaggagatcacagccggagctaatcgctggagaagacggccatgatgtgtcatggaaggaaggtgaagatgagagacaacgagatgtctcagattcgccaaagtttgataccaacgggaacagaggtgtcagccggtgatggagaacgtcgccggtgtaaaacgccggaggtaatggcaatccggtggtgtcgttgttggcttcacgaagggacaaagggaaaaggtggtttcagataacgatttcgtcgaggaagattgtgttttgtcaaaaggtggagaat

>ATCOPIA37_LTR

tgttgtttgggtttgacataacacaattattgggccaacaaaattggcaaggtgaggaagaacacaagtgtgggcaaaagtatgtggcccatttacgtcacctataaatgcattacaacgtacgacgtatagtggaggtaggtgagaaaagaatctaaagagagttatagagaagagagataaaagagcttccaaagcaagctttcttcttcctctttgttgtttttgaatctttgtttttcttgagtgcaaatctgtaaaaaccaaaagacaacatcacaagttagtggtgtgtttgtaatccctttgatgtagtgaaacttgggagcaagagatctcccccgagatgtaggggttgaggctgtgaactcggttatcaattttctttgtctgtttatttctttaccaagcaatctctatcacttttttctccaca

>ATCOPIA38A_LTR

tatcagatatgtatattggtttagttgatataagttggtttagtagaaccattgtatagtgtaaagtatataagacatttgtacagaatcattttacaattaatgagaaaagccttcataatatcttcaacaaacttttctctcatctctattccgctaata

>ATCOPIA38A_IN

tggtatcaaagccttccaagctcaaaactccattgttttcgatctgatttcttcattctcagctcaagttcgtcttcttcgttgttctcgctccagatacaatggtgacagctagagttactcggaaatccactcgatcgaagtccggtacaagttctgcagctcgaaaatcatcgcgatccaccggtgttgatgaatctcctccgatctctcctccagttcacaaatccaacgtttctggagcttcgcgagtcatcgcaccaccggaatccgttgatccgactcagtctcccttcttcttgcatagtgcagatcatccaggtttgaatatcatatctcatcgtcttgatgaaacaaactatggtgattggaatgttgctatgcttatctccttagatgctaagaataaatctggatttattgatggaactctccctcgtccgttggaatcggataagaattttcgtctttggtctagatgcaaaagcatggtgaagtcttggttattgaattctgtatctcctcagatctataggagcattctccgtatgaatgatgcttctgatatatggcgtgatcttcacagtcgtttcaatgtgactaatctcccacgcacatataatcttactcaagagatccaggattttcgccaaggtacattgtctttgtctgagtactatactcgcctcaagacattgtgggatcaattagaaagtacagaggaactagatgatccctgtatttgtggtaaggctatgcgtttgcaacagaaggcagagcaagctaagatagtgaaattgttagctggtttgaatgattcttatgctatcattcgcagacagatcattgctaagaaagcactcccaagtttagcagaggtgtatcacattctggatcaagacaacagtcaacaaggtttttccaatgtggttgctccaccggctgcttttcaggtctctgaggcaatgatggctactaacaatgatgtgaatatgctatgttcagagtggtccaaacaagggcagaccgatttgttctttctgtaatagggttggacatattgcagaaagatgctataaaaagcatggattccctcttggttttactcctaaaggcaaaggtggtgacaagtctcaggtgatagattcaggagcaatacatcatgtatctcatgataagagtcttttcttgaatttggatacttctgttgtgagtgcagtgaatttaccagctggtcctactgtaagaatcagtggtgttggtacattgcgacttaatgatgacattcttctcaagaatatcttattcattcctgagtttcgcctgaatctaatcagtataagttctctgactgatgacattggttcacgagtaatatttgataagacttcttgtgaaattcaggatcctatcaaggctcggatgcttggtcaaggtaggagaattgcgaatctatatgtgctggatattgaagatcctattgtctcagtgaatgcagtggttgatatcagtatgtggcatcgaagacttggtcatgcttctctacagagattggatgttatttcagactctttggggactactaagcctaagaataaaggctcagattattgtcatgtttgtcatttagctaaacagagaaagttacccttcccttctcagaataaagtttgtaatgaaatctttgatttgttacacattgacatttggggtcctttttcagttgagacagtggatggctataaatattttttgacaattgtggatgatcattccagagctacatggatttatttgcttagaaacaagtcagaagttcttactgtgtttcctgctttcattgaacaagttgagaatcagtataaagtgagagtaaaggcagtgagatcagataatgcaccagaattgaaattcacaagtttatatcagcagaaagggattgtttcgtttcattcctgtcctgaaacaccggagcagaatttagtggttgagcgtaaacatcaacacatactcaatgtttctcgggctctcatgttccaatctcaggtaccactctctctttggggtgattgtgtgttaacggctgtgtttcttatcaacaggacaccttcccagctcttatcaaacaagactccatatgagattctcactggtacagttcctgtttatgagcagtttcgaacctttggttgtttgtgttacagttctacttcgccaaaaaaagacataagtttcagccgagatctaaagcgtgtgtgtttttgggatatcctgctggttacaaggggtacaagttaatggatttggagagtcacactgtgtttatctcaaggaatgttgtttttcatgaggaggtatttccattggctgtggatccaaagtcagagagttctttgaagttgtttactccaatggttcctgtgtcctcaggtattacacaatctcctatctcttctcttccatcacaaatttctgatctaccaccacaaatttcttcacagagagttaggaaacctcctgctcatctgtcagattatcattgcaacatcacgcaatctaatcacaaatatcctatttcctctactatctcttactctaaaatttctccatctcacatgtgttatatcaataatatcacaaaaattcctattcccaccacatttgctgaggcacaggacaccaaagaatgatgtgaggctgttgatgcagagattggtgctatggaaactacacacacttgggagattactactttacctgcagggaagaaggcagtagggtgtaaatgggtattcagtttgaagtttttggcagatggcagtcttgagcggtataaggctagattggttgctaagggttacactcagaaagaaggtctagactatacagatactttctctccagtggcaaagatgactacaatcaagttgttattgaagatttctgcctctaagaaatggttcttaaaacagcttgatgtgtctaatgctttcttgaatggtgagttagaggaagagatttacatgaagttacctgaaggttatgcggcaaggaaaggtatcactctacctcctaattcagtctgtcgattgaagagatccatttatggtttgaaacaggcctctcgtcagtggtttaagaagttttctgcttcattacttgatttgggttttaagaagacacatggtgatcatactttgtttattaaagagtatgatggagaatttgtgattgttttggtttatgttgatgatattgctattgcgagtacaagtgaaggtgctgcaatacaattgactcaggatttacaacagcgattcaagttaagagatcttggggatttgaaatattttttgggtcttgagattgctagaactgaagctggtatttctatctgccagagaaagtatgctcttgagttattagcatctactggtatggttaactgtaaacctgtttcagttcccatgatcccgaatgtgaagatgatgaagactgatggtgatttgttggatgatagagaacaataccgtcgtattgtaggaaagctgatgtatttgacaattactagacttgacatcacctttgcagttaacaagttatgtcagttttcctctgctcctaggacgtctcatcttcaagctgcttatcgagttttacaatatatcaaaggcagtgttggccaaggtctgttctattctgcatctgcagatcttactcttaaaggttttgcagattcagattgggcttcctgtccagatagtaggagatcaacaactggattcagcatgtttgtgggtgattcattgatatcttggcgttccaagaaacagcatgtggtttccagatcttctgcagaggctgagtaccgtgccttagcactggttacgtgtgaactggtatggcttcacactttgctcatgtctttaaaagctagctacttggttcctgtgttatattctgatagcacaactgctatttacatcgccacaaatcctgtgttccatgaaagaacgaagcatatagagctagattgtcacacggttagggagaaactagacaatggcgagctgaagttgcttcatgtgcgaactgaagaccaagtagcggacattcttactaatccgttatttccccatcaattcgaacaccttaaatccaagatgagcattcttaatatcttttcatgcacatcttgagggggca

>ATCOPIA38B_IN

tggtatcagagccaacaacgctctcatctccatcgtttttttttccgacttgatttcttcgttctgagctccgattcgtcttcctcgttgcatttccttaagatccaatggtgacggtagctcgtgtgactcggaaatcgactcgctcgaaagctggtacaagttcagtgactcggaagtcgcgatccaccggtgctgttactactcctccgaattctcctccggttaacagatctggagcttcgcgagccttaacatcatcggaatctggagatccgactcaatctccctttttcctgcatagtgcagatcatccaggtttgaacataatctctcatcgccttgatgaaacgaattatggtgactggagtgttgctatgcttatttctttggatgctaagaacaaaactggattcatagatggaactttatctcgtcctttagaatcagatttgaattttcgtctatggtctagatgtaatagcatggtgaagtcttggctattgaactctgtttctcctcagatttatcgaagcattctccgtatgaatgatgcttctgatatatggcgtgatcttaatagtcgattcaatgtgactaatcttccacgcacgtataatcttactcaagagattcaggactttcgtcagggaactttgtctttgtctgagtactacactcgtctcaagacattgtgggatcaattggatagtacagaggctttggatgagccttgtacttgtggtaaagcgatgcgtctgcaacagaaggcggagcaagctaagatagtgaagtttttggctggtttgaatgagtcttatgctattgttcgcaggcagatcatagctaagaaggctttacctagcttgggagaggtgtatcacattctggatcaagacaatagtcaacagagtttctccaatgtggttgctccacctgctgctttccaagtttctgaaataacacaatctcctagtatggatcccacagtttgctatgttcagaatggtcctaataaaggtaggccaatttgttctttctacaatagagttggtcacattgctgagagatgttacaagaaacacggttttcctcctggtttcactccaaaaggtaaggctggtgagaaacttcagaaaccaaaacctttggctgcaaatgttgctgaatctagtgaagttaatactagtttggagagtatggttggaaatttgagtaaggaacagcttcagcaattcattgctatgttcagttctcagcttcagaatactcctccgagtacttatgcaactgcaagcacttctcagtcagataatcttggtatttgtttttcaccatctacctatagtttcattggaattttgactgtagctcgtcatactctttcttctgcaacatgggtgatagactctggagctacacaccatgtgtctcatgatagaagtttattctcaagcttggatacttctgttttgagtgcagtgaatctgccaacaggacccactgtgaagatcagtggtgttggtactttgaaactgaatgatgacattctacttaagaatgtgttattcatcccggagtttcgactgaatcttataagtataagttctttgactgatgatattggctcccgtgtgatatttgataaaaattcttgtgaaattcaggatcttatcaagggccggatgcttggtcaaggtagacgagtggcgaacctctacttgttggatgttggagatcagtctatctcagtgaatgcagttgtggacatcagcatgtggcataggaggcttggtcatgcttctttgcaaagacttgatgctatttctgattcattgggaactactaggcataagaataagggttctgacttttgtcatgtatgtcatttggcaaaacagcgaaagttatcttttcctacttcgaataaagtttgtaaagagatatttgatctactacacattgatgtttggggtcctttctcagttgaaacagttgaaggatataagtatttcttgactattgttgatgatcattcaagagcaacatggatgtatttgcttaagactaaatcagaggttcttacagtatttccagcgttcattcaacaagtggagaatcaatataaagtgaaggttaaggctgtacgttctgataatgcacctgagttaaaatttacaagcttctatgcagaaaaagggattgtttcttttcactcatgtcccgagactccagagcagaattcagttgtggaaagaaaacatcaacacattttgaacgttgctcgagctctcatgtttcagtctcaggtacctctttctctgtggggtgattgtgttcttactgctgtgtttttgataaacaggactccatcacagctattgatgaacaagactccatatgagattctcactggtacagcacctgtttatgagcagcttcgtacttttggttgcttgtgttatagttctacatctccaaaacaaagacataagtttcagccaaggtcacgagcttgtttgttcttgggttatccttctggttacaaaggttacaagctgatggacttggagagtaacacagtgttcatctcaaggaatgttcagtttcacgaagaagtgtttcctctggcaaagaatccgggttcagagagttcgttgaagttgtttacaccaatggttcctgtgtcctcaggtatcatatctgatactactcattctccctcttctcttccatcacaaatttctgatctaccaccacaaatttcttctcagagagttaggaaacctcctgctcatctgaatgattatcattgcaacaccatgcaatctgatcacaaatatcctatttcttctactatctcttactctaaaatttctccatctcatatgtgttatatcaataatatcacaaaaattcctattcccactaattatgctgaggcacaggacaccaaggaatggtgtgaggcagttgatgcagagattggtgcaatggagaagacaaatacatgggagattactacgttacctaaagggaagaaagcagttggttgtaaatgggtgtttactttgaagtttttggcagatggtaatcttgagaggtataaggcacgtttggttgcgaagggttacacacagaaagaaggcttggattacacagatactttttctccggttgctaaaatgaccactattaagctgttgttgaaagtttcagcctctaagaagtggtttcttaagcaacttgatgtttctaatgctttcttgaatggtgagttggaagaagagatattcatgaagatacctgaaggatatgcagagagaaaaggaattgtgttaccatctaatgttgttctaaggttgaagagatcaatttatgggttgaaacaggcttcaaggcagtggttcaagaaattttcaagctctttactcagtttgggttttaaaaagactcatggtgatcatactttgtttctgaagatgtatgatggtgagtttgtgattgttctggtttatgtggacgatattgtgatagctagcactagtgaagctgctgcagcacaattgactgaagagctggatcaacgtttcaaactgagagatttgggtgatttaaagtattttttgggtcttgaagtggctaggacgactgcagggatttcaatttgtcaacgcaagtatgctttggagctattacagtctacaggtatgcttgcttgtaaacctgtttcggtccctatgattccaaatttgaagatgagaaaagatgatggtgatttgatagaggatatagagcagtatcgacggattgttggtaagctaatgtatttgaccataacaagacctgacattacatttgctgtcaacaagttgtgtcaattctcatcagcacctcggactactcatctcactgctgcttatagagttctgcagtacatcaaaggcacggttggacagggtcttttctattcagcatcttctgatcttactctcaagggatttgctgattctgattgggcttcttgtcaagacagtcgacgctctactacaagtttcacaatgtttgttggtgattccttaatatcttggcggtctaaaaagcagcacactgtttcaagatcttctgcagaggcagaatatagagctttagctctagccacctgtgaaatggtctggttgtttactctgcttgtctctttgcaggcttcaccaccagttcctattctgtactctgatagtactgcagcgatctatattgctacaaacccggtcttccatgagcggaccaagcatataaaattggactgtcacacagttagagaacgcttagacaatggcgaactcaagttgctgcatgtacgaacagaggatcaggtggccgatatactgactaaaccattgttcccctatcaattcgaacatctcaagtccaagatgagcattctcaacatcttctcatgctcatcttgagggggcc

>ATCOPIA38B_LTR

tatcagattagataccggtttagctggtttagtatagctggttaacttaaatcaattgtatagctatagtataaatacatgtacattacacattatgtgattgaatgagaaaatcagattaatatcctcaacaaactctctttgatcttcttcttcgtaata

>ATCOPIA38_IN

tggtatcagagccatccaagctcaaatttcattggttactcgatctgatttctccgttttcaagctcagaatcgtcttctccattgctcaagctcaagatccaatggtgacagtagctcgagtgacgcggaaatcaactcgatcgaagtcaggaacaagttcagctcgcaaacagtcgcgatccaccggcattgatgattctcctccgaattctcctccagttgctcgatctaagactactggagcttcgcgagtcatcgtttcaccggaatcaacggatccgactcaatcaccgttcttcttgcacagcgccgatcatccatgtttgaatatcatctctcatcgtcttgatgaaacgaactatggagattggaatgttgcaatgctcatttcgttagatgctaagaacaaatctggattcatagattgaactctgcctcgtcctttagaaacggataagaactttcgtctatggtctagatgcaataccatggtgaaatcttggcttctcaattctatgtctcctcagatttacaggagtattcttcgtatgaatgatgtttcggatatatggcgtgatctcaatagtcgattcaatatgactaatcttccacgtacatataatcttactcaagagattcaggatttgcgtcaaagtactttgtctttgtccgagtactatactcgtctcaagacattgtgggatcaattgaatagtacagaggagttggatgatccttgtacatgtggtaaagctcttcgtctgcaacagaaggcagaaagagctaagatagtgaagtttttagctggtttgaatgaatcttatgctattattcgcagacaggttattgctaagaagatacttccgagtttagcagaggtgtatcacattgtggatcaagacaatagtcaacaaggattttcaaatgtggttgctccaccagttgctttccaagtgtctgaagtcactgttgctaatatcattgatcctactatctgctatgttcagaattgtcctaataaggggagaccgatgtgttctttctataatagggttggtcacattgcagagagatgctataagaagcatggttttcctccaggctttactcctaaggataaggttggtgataaaacacaaaaaccaaagtctgttgttgctaatgttgctttggctactacaaaatctgatgatactcattctggtctggagagtttggtgggcaatctgagcaaagaacaacttcactagttcattgctatgtttagctctcaacttcagcctcaacctcacagtaattctgcagtagctagctcatctcaagctgataacattggtatttctttctctccttccacctatagttttattgggattttgactgttgctcaacatactctttcctctaaaacatgggtgatagattcaggagctacacatcatgtttcacatgatagagatctgtttttgactttgaatacatctgttttgagctcagtgaattaactagctggtcccactatgaagattagtggagttggtactttgagactcaatgatgatattcttctcaagaatgttttgttcatacctgaatttcgtcttaatctgattagtataagttcattgacagatgatattggttcaagagtgatatttgatcaacatgcttttgaaattcaggatcttatcaaaggccggatgcttggtcacggtaggagagtggctaatctttatgtcatggatgttgaagatacaaatgtttcagtgaatgcagttgtggatatcagtacgtggcataacagactcagacatgcttctctacaaagactggatgttatttcagaatcattgggaactactaaacacaagaataaaggatccgattattgtcatgtttgtcatctagctaagcacagaaagttgtctttcccttctcagaataatgtttgtaatgagatttttgagatgttacacattgatatttgggggcctttctcagttgaaacagtggatggttatcaatatttcttgacaatcgttgatgatcattctcgtgcaacatggatttatttgctcaaaactaagtctgaagttcttacaatatttcatgatttcattcagcaagtggagaatcagtataaggttaaagtgaaagctgtaagatctgacaatgctccagaactgcgttttacaagtttgtatcaacggaaagggattatggcttttcattcttgtccagagactccagaacagaactcggttgtagaacgtaaacatcagcacattcttaatgtggcaagggctctcatgtttcaatctcaggtaccactttttctatggggtgaatgtgtcttaacagctgttttccttatcaacaggacaccttcacagcttctttcaaataagactccttatgagattctcagtggtactgctccccaatatggacagctcagaacatttggttgcttatgttacagttctacatcaccgaagcaaagacataagtttcagcctcgatcaaaagcttgtatcttcttgggatactcgtctggttataaaggttataaacttatggatttggagagtaatgccatttttatttctaggaatgtggtgtttcttgaggaggtgtttcctttggctggtactaagaagtcagcagattctctgaagttgtgtacaccattagttcttgtgccttcaggtattcaacaacaatcttctttttcttctctttcatcacaaatttctgatctaccaccacaaatttcttcacagagagataggaaacctcctactcatctgtcagattatgtttgcaacaatatgcaatctgatcaaaaatatcctatatcttctactatctcttactctcaaatttctccatctcacatgtgttatatcaataatatcacaaaaattcctattcccacgaattttgctgaggcacagggcactaaagaatggtgtgaagctgttgatgttgagattggtgctatggaatctacgaatacatgggagattacaacattaccaaagggtaagaaggcagtgggttgtaaatgagtgtttactttgaaatttttggcggatggtagtcttgagaggtataaggctagactggttgctaagggttatactcagaaggagggtctagattatacggatactttttctccagttgctaagatgacaacgatcaagttgctgttaaagatctctgcttctaagaaatggtttatgaaacagcttgatgtgtctaatgctttcttgaatggggagttggaggaagaaatatacatgaagttacctgaaggatatgctgagagaaagggtattatcttaccatcaaatgttgtttgtcgtttaaaaagatccatttatggtttgaaacaggcttctcgacaatggtttaagaaattttccgcatctttactcagtttgagattcttcaaaacacatggagatcacacattgtttctcaaagattgtggaggtgagtatgtggttgtgttagtgtatgttgatgatattgtcattgctagtactaatgaggcagcagcagtacagttgagtcaggatttacagaatcttttcaagttaagagatcttggagatttgaagtattttttgggattggaaattactagaactgaagcaggaatctcattgtgtcagcgaaaatatgctttggagttgttggcatctactggtatgataaattgcaaacttgtttcagttccaatggttccaaatttgaaactgatgaaagttgatggtgagttacttgaggatagagaacagtatcgtcgaattgttggcacgttgatgtatttgactataactaggccagatatcacgtttgcggtgaacaagttatgtcaattctcttatgcacctacgactgcacatcttcaggctgctcacagagttcttcaatacattaaaggcactgttggtcaaggcttattctattctgcttcttctgatcttactttgaaaggatttgcagactcggattgggcgtcttgtcctgatagtcgtcattccactactggtttcactatgttcgtcggtgattcccttatatcattgagatccaagaaacaacatgtggtttctcggtcatcagctgaggctgaatatagagctctcgctcttgctacttgcgagttagtatggttacatactttgcttgcgtctttgactgctgctactacaattcctatattattttcagacagtacagcagcgatttacatagcaatcaatccggtcttccatgagcggaccaagcacatcgagattgattgccacaccgtgcgagaaaagatagatgatggtgagttgaagttgttacacgttcgcacggaagatcaagtagcagatatcatgactaaacctctgttcccaaaccaattcgaacatcttaagtcgaagatgagtattctcaacatttttgaatcctcatcttaagggggca

>ATCOPIA38_LTR

tattagagagtgttggtttatttatgtggtttagttcattatgtatttcggttatgttaaccggttgtataatcatagtatatatacttgtacatatacattttacagagttaatgagaaaatatcttcttctatgtctaacaaattttctcttctctctcaatcgtaata

>ATCOPIA39_IN

tggtatcagagctacgctgattactggaaccaaacgaaatggctcaagatctcgggatgaaaacgccggtgaccaagagggacaccaagaaagaaagaaaaggctcacaaaagagcaaactgggaatggctacagagacagcagaagaagtgaagaaaaatcttgtgtttattgcaagacaagagattaaagaaccaagtggaggaaacacttggttgatcgacagtggatgcacaaatcacatgacaccaaatgagaagctgttcacaaaaatcaacagagacttcaaagttccaataagagttggaaatggagctgtaatgatgagtgaaggaaaaggagatattgaggttatgacaaggaaagataaaagaggcatacgtgatgttctccttgtacctaaacttgggaaaaatttgttgagtgttcctcaaatgatcatcaacggatatcaagtcacattaaaaaacaactactgcaccattcatgatagtgcaagaaagaaaattggagaagtagagatggtgaacaagagttttcacctaaggtggctttcaaacgaagaaacagcaatggtggctaaagatgaagcaacagagttatggcacaagcgacttgggcatacaggacactcaaaccttaagatcctgcagtcaaaagaaatggtaactggcttgcctaagtttaacgtggaagaaggaaagtgtgaaagctgcatcttaagcaaacacagtcgtgatccatttccaaaagaatcagaaacaagagcaaaacacaagcttgagctcattcacagtgatgtgtgtggacctatgcaaaactcatctatcaatggtagcaggtacatacttactttcattgatgatgctacaagaatggtttgggtctacttcctgaaagcaaaatcggaggtatttcagactttcaagaaatttaagaatcttgttgagaataatgcaaattgcagaatcaagaagcttagaatagatagaggaacagagtatctttcgaaagaattttcagagtttctagaagggaatggcattgagaggcaacttactgcagcatactctccacaacaaaatgaagtctcagaaagaagaaacaggagtttagtggagatggcaagagccatgattaaagcaaaagatctgccattaaaattatgggctgaagctgttcatgttgcagcctatgctcaaaatagaacaccaacaagaaccctgaagaacaagacacctctagaagcttggagtgactcaaaaccgtctgtaagtcacatgaaagtgttcgggagcatatgctatgtccacatacctgatgaaaagaggaggaaatgggatgacaagtcaaaaagagctatatttgtcggatacagctctcaaacaaaaggttacagagtgtacctgttgaaagaaaacaagatagacatttccagagatgtcatctttgatgaagacagtaaatgggactgggaaaagaaggaagtaatcaaacattatgatatgtcacgtgaaccagaagacagaggagaccaacaagcagatgagcagaactcaagagataatgaagctagagggagaagaaacgttttaccaagtcccttgaatctgaattttgacagtcctggaggcagcacaagtcagccaaacaagaaaaccagatcgattaatgacattctcctcactgctccatttgcagatgtttaatactaaggagcatgtgaaagctgctatactggtgcagaagagcctgcagtatttgaagaggctgcaaaacatgaggaatggacacaagcaatggaagaataaatattaatgatcgagaagaacaaaacgtgggagctggtgaaaagaccaacaaacaaaaatgtggtaggcgttaagtggatattcaggctgaaaactgatgcagaagggaatgtggtgaaacacaaggcaagacttgtagcaaaagggtttactcagcaacatggggttgactatctagagacatttgctcctgtctctagacatgaaacaatcaggttgattcttgttgttgcagctcagaggaaatggaaattgttccaattagacgtcaagtctgcatttctaaatggtactcttgaagaggagatatacgcagaacaacctcttggatttgaggaagaaggaaaagaagatcatgttctgcgtctacacaaagctttgtacggtctgaaacaggcaccgagagcctggtatagtcgaattgatgaattcttccagcgagagaacttcacaagaagtgacaatgatcatgctctatatactaaagaagttctgggaaagctattggtggtgtgcatatatgtggatgatctcattgtaacaggtgatgatgaggaaatggtggaagaatttaagactgcgatgaagaatgagtttgaaatgtctgatttgggattacttaactactttctgggaatggaaattgtgcaaagtcttgaaggaatttttctatctcaagagtgctatgcgagaaaactgttgaaaaagttcaacatggaagacagcaagatcatgagtacaccactgttgcctcaaagaaaagatcaagaagaagatgagagtctggccgactcaaaagtttacagaagtctggtagggggtttgttatacctaacaagcactcgtcctaacttaatgttttttgcatcatacttgtctaggtacctaaaggaaccaaaaatcaaacactttaaggaagctaagagggttcttaggtacattaatggaacaattgatatgggaatgaggttcacttcaactatggagccaaagctgattggtcactcagacagtgattagggaggttgcagagaagatctgaaatcaactacaggatattgttttagtattggatcagctgtgttcacatggcaaaccagcaagcaagagaccattgctcagtccactgcagaggcggaatatatggcgttgtgtgcagcaacaaatcaatcaatgtggttaaagcgattgttagaagacttgaagttcaccactcaagagggagtacctatttactgcgatagccagtcagctatgccattggtaagaatcctgtccagcatagaaggacaaaacacattcagatcaagtatcatgtcgtaagggaagcagaaagaaatggggatatcaaactgttatactgcagaagtgaagaacagttggctgacatactaactaaagcattgggaagacagagctttgaaggatttcgagaaaagcttggtttgagttgcaaaatggcaagagggag

>ATCOPIA39_LTR

tgttgaataatgcctattttgctttcaaaccatactgaagaagtcaaagagagaacaagtagttacatgtgaaagaagttggtttgaatctagttgcttttgtttacttggaaaactaagctataatctagagtaagcatgctagtagtgggagcagcctgcaacagtgtaatcgtgtaggtgtcatcgtgtaggtgtcagaagctagtattttgtgttttactttatgttttgctataaggatgtatgtgtgtgtaagaaagaattatgggaaaattctaagaaaaacagagtttatgcatctctctctcaacttctaaattgttttaattcacaaaacatgagtgaaagtgagaatcacaaca

>ATCOPIA3_IN

tggtatcagagctttaaacgatccctaaacctaaatctagccgctactctgtttttcatcatttttcattatttttctttctcttcaatggcttctgagactgtcattacatctgattcaaccagtcttctcaacgtcaatatgactaatgttaccaaactcacgtcctctaactttctcatgtggaggcgtcaagttcaagctctactcaacggctacgatctcaccggctacattgacggctccattgtcgtcccacctgcaaccatcactgcaaatggtgcagttacggtcaatcctgccttcaagcactggcaaaggcaagatcaactcatttacagtgctctactaggcgctatttcaatctcggttcaaccgatcctctcccgaacaacaacatctgctgaaatttggactaagctgatggacacatatgctaaacccagctggtctcacatccaacaattacgacaacagatcaagcaatggaagaaggacaccaaatccatcgatgaattcttccaaggcttggtgatgcgatttgatcaactagctctccttggcaagccgatggagagtgaggaacagatggaagtcattgtggaagggctctccgatgactacaaacaggtcattgaccaaattcaaggtcgtgaggtacctccctctctcactgaaattcacgagaagttacttaatcatgaagtgaaacttcaagctgcggcatcgtctcttccgatctctgcaaatgctgcaagttatcgtcctccagccaacaacaagcataacaactccaacaactatcgcggacaaaaccgtaacaacaacaatcgtggtgctaattcataccaacagccaagaaacgatcagccctcttctcgtggttatcaaggaaaatgccaaatctgtggagtttttggccatagcgctagacgttgttcacagcttcagatgtctggcgcttactccaccccatctccaagtcaataccctaatgctactgttccatggcagccccgtgcaaatatggcagccatgtcgtacaatccttggcttcttgatagcggagcaacacaccatcttaccacagatttgaacaaccttgctcttcatcaaccttacaatggtggagaagaagtcacaattgctgatggctctactcttccgatcactcacactggttcatcaactctctctactcaatctcgttcccttgctttaaacaatattctatatgttcccaacctgcacaaaaatctgatttcagtttacaaattgtgcaatgctaataaggtttctgtggaattctttcctgcccactttcaggtgaaggatctcagcacgggggcccggttactccaaggcagaactaaagacgagttatacgagtggccagttccttcaaacacacccatctctttattcgcttcacctacaccaaaaacaacacttccttcatggcattcaaggcttggtcacccgtctccgcctgttttaaaatcccttgtttctcagttttctttaccagtttcaaattcctctcaaaaacattttccttgttctcattgtctcattaataaaagccataagcttccgttttattcaaacacaattatctcctacacacctcttgaatacgtttactctgatgtctggacttctccagttacatctgtcgacaactttaaatattatttaatccttgttgatcattatacccgatatacatggctgtacccattgaaacaaaaatcacaagtacgggagacttttgtggcgttcaaagctctagtggagaatcgttttcaaaccaaaatccgaacattatattctgacaacggtggagaattcattgcgctacgacaattcctgctaacacacggcatttcacacttgacctctcttcctcacacgccggaacacaatggaattgcagaacgaaaacatcgtcacatcctcgaaaccgggttaactcttctcactcaagcctctattccaacttcatattggacatatgcgtttggaactgcggtttacctcataaatcgcttgcccagttctgtgctcaataatgaatcgccgtattcgaagctcttcaagacttcaccaaattacctcaaattaagagtttttggatgttcgtgcttcccatggctccgcccatataccaatcacaaattggaaagaaggtcacagccgtgtgtatttctcgggtattccttaacccaaagtgcatatttatgccttgatagaagctctggtcgtgtctacacttcaagacatgtccagtttgttgaggatcaatttcctttttccatctccgatacccattcagtttctaattcaagcccagaagaagcttccccttcatgtcaccaacccccatctcgcattccgattcagtcatcatctccgccactcgtacaagctccgtcgtcgctgccgcccctaagctcagattctcaccgccggccaaatgctgaaacttcatcgtcttcgtcctcgacgaataatgatgttgtggtatcgaaagataacacacaagtggataacaggaataatttcataggcccaacaagcagttcttcagcccaaagccaaaataattctaatccaagctcatccatccaaacccaaaatgagcccaatccgagcccgtcaccaacacctcaaaattctagcccagaatcttcaccatcgtcttctacctccgccacgtccacagtcccaaatccaccaccaccacctccgacaaacaaccacccaatgcgaacccgagctaaaaatcatataaccaagcccaaaacgaaactctccctcctagcaaaaacagttcaaacccgacctcaaataccaaacacggtgaaccaggctttacgagatgaaaaatggaggaacgctatgggagaagaaatcaatgcccaaatacggaataatacatttgaattggttccaccaaaaccaaatcaaaatgttatttccacaaagtggatttttacactaaaatatcttcctaatggtactcttgacaggtataaagcaagattggtggctcgaggttttcggcagcaatatggactccattactctgaaactttcagtcctgtggttaaatctctcacaattcgcctagttctacaattggcagtgtctcgctcatggacaattaaacaactagacgtaaacaacgcttttcttcaaggtacattgacggatgaggtgtacgtcacacaaccacctggttttatcgaccccgatcgaccacaccacgtttgtcgccttaagaaggcgctttatggcctgaaacaggctccacgcgcttggtaccaagagttgagaaattttgtttgctcgttgggattcaccaattctctcgctgatacatcagtgtttgtttacatcaacgacatccaaatcgtctactgcttggtctatgttgatgacattattgtcacgggaagtagtgatgcgttggtaatggctttcatcaccgctctttctcggcgattctcgttgaaggacccgacggatttggtatattttcttggtatagaggcaacaagaacctcacaaggtcttcatttgatgcagcataaatatgtttatgacttgttgtctaggatgaagatgttagatgccaaaccggtgtccacaccaatggcgacacatcctaaactttcactctactctggcattgctctagacgaacccggagaatacaggacagtgatcggcagcttgcaatacttggcgtttacacgaccggatattgcgtatgctgtgaatcgattatcacaatttatgcatcgtcccacagacattcattggcaggctgcaaaacgcgtccttcgatatcttgcgggaactgctactcatggtatattacttcgctcaaactctcctctctcgcttcatgcattctccgatgctgattgggctggggacaatgacgattttgtttccaccaatgcatatattgtgtatcttggctctacacccatagcttggagctcgaagaaacaaaagggagtcgcacgttcttccaccgaggccgagtacagggctgttgcaaacacaacttcagaaattagatgggtatgctctcttcttactgagttaggcatcacattaccaaagatgcctgtgatatattgtgacaatgttggtgccacatatctctctgcaaatccggttttccattcaagaatgaagcatctagcattggactatcattttattcgcgacaatgtgagtgccggtgcactacgagtctctcacatatcaacacatgaccaacttgccgatgctctaaccaagccattgcctcgtcaacactttcttcagttttcaagcaagattggagtgagcaaactccctccatcttgagggggca

>ATCOPIA3_LTR

tgtagagtatataagtaaaggatagtttaataaatcattaatccctaattcattctaatgtagtcactatatatattgtaattatatcatctaatgaattattcagccttcaatactata

>ATCOPIA40_IN

tttggtatcagagcatccaggttttcgatcaaagcaaaggattgaaaacatgagtgagattattgcagtaaacaacaaatcaaaagatggaggaggatcatcgtctattcaatgtccgatgttgaataacacaaactatacggtatggtgtatgaggatggaggctgcactacgagtgcataaagtctggggaacaatcgatcctggattagaggacgaagagaaaaacgacctagccgagcccttctcttccaatctattccggagtcacttattcttcaagtcggaaaactgaagacttcaaaggcagtatgggataagattcaatctaggaatcttggagcagaacgagtcaaagaagcaaagctaaaaactcttatggcagagtttgacaaactcaagatgaaggacaatgaaactattgatgaatgtgcagggagactttcagaaatttcaacaaaatcaacatctttgggagaagatattgaagaaacaaaagttgttaagaaatttttgaaaagcctaccaacaaagaagtatattcatatagttgcagcccttgagcaagttctagacctcaagaacacaacattcaaagacatagtagggagaataaagacctatgaggacaaagtatgggatgacgatgattaactagaagaccaaggtaaattaatgtatgtagatttgggttctcattacatgtctcaagaaggaggcagaggaagaggaaaaatccgtggtcggggtagaggcagaggaagagttggttataagcagagagatcactctaaggttacttgttatcgctgtgataaattatgcctctaattgtccagaccgactattgaagttaatcaagctccaagaaaggcaacaagaggctgaagacgatgatgatgatgaagtggagtctcttatgatgcatgaagtagtgtatctcaacgagggaaacatgaatctagagatttatgaagcatgttctgacaaagcttggtatttagacaatggtgctagtaatcatatgactgggaatcgtgattggttttgcaaactcgatgagatggtcactggaaaggttaagttcggagatgattcacgaattgacataagaggcaaaggatcgatattgtttttgaccaaaaatggagagccaaagacactagcaaatgtctattacatacccgatctcaaaagcaacatcataagcttgggacaagctactgaggctggctgcgacgtgagactaaaggacaactacctgacattacatgatcgtgatggtaacctgttggtgaaagcaacaagatcgaggaacagactgtacagagtggagctaaaagtcaaaaacactaaatgtttacaacttgcagcacttaacgacttaacaaagtggcatgctcgattaggtcacattaatcttgagaccataaaggcaatggtaacaaaagaatttgtgattggaatacctagtgccccaaaggagaaggagatctgtgcttcatgtatgcttgggaagcaagcaagacaagtttttccgaaagctactacttatcgtgcttctcagattctagaactcatccatggagacttgtgtggacccatttcaccacctacagctgcaaagagaaggtacatactagttctaattgatgatcactcacgcttcatgtggtcatttcttttaaaggagaagagtgaagcattcggaaaattcaagacttttaaagcaacagttgagcaagaaaccggagaaaaaatcaaaacactcagaacagatagaggaggcgagttcctatctcaagaatttcagacattttgcgaagaagaaggaatcattaggcacctcaccgcgccttacactcctcagcaaaatggagtagttgagagacgaaacagaaccttgttgggaatgactagaagcatcatgaaacacatgagtgttccaaattacttgtggggagaagcgataagacatgcaacatatctcattaacagagtgggaacaagagctcttatcaaccaaacgccctatgaagctctcaagaaaaagaagccaaatgttgaacacttacgcgtgtttggttgtgtgagttatgccaaagttgaattcccacacctaagaaagcttgatgagaggtcgaggattctagtctatctcggaacagaaaccggctccaaggcttatacattactagatccaaccacgcggaaaatcatagtaagcagggatgtagtgtttgatgagaataaaagctggaagtgggctaactctgaattgattgaaattcagaaagaaccaggaatgtttactcttgctcaaacagagtttcacaataacgaagaagttgagaacgaaacctcagaagaaatagaagaaaatgaagcagaggactcaactaataaagacgcagaagacggtcttgatgaaccaagtgttcctgaacccaatcacagcgatgatgtaccagtctttacacgctcaggaagacaggtcataaaaccggcacacttaaatgattatgtgttgttggctgaaattgagggtgaacgacttctgcttctgattaatgatgaaccctgggattttaaggaagcaaataaatacagagaatggcgtgatgcgtgtgatgaagagatcaagtcaataataaaaaacagaacatggagcttagtctctctacccgtcggtatcaaaccaatagggctgaagtgggtgttcaagataaagagaaactctgatgggaacataactaaacacaaagcaagactcgttgcaaagggttatgtgcaaaaacatgttgtagactttgatgaagtgttcgctccagtggctcgcattgagacagttcgattcatcatcgctcttgctgcatctaatgggtgggaagtgcatcatctagacgtcaagactgcattcctacatggcgaattaaaggagaatgtttatgtcacccaaccggagggctttgttacaaagggaagtgaagagaaggtctacaagctgcataaggctctctatggtttgcgccaagctccacgtgcatggaacattaagctcaatgagattctttagaagttgttattcgaaaggtgctctaaagaaccttctctgtatcggaaacaagagggtaagcatcttcttatagttgcagtctatgtggatgatctacttgtcacaggttctagtccaaaactgatagatgacttcaagaaaggaatgtcaaagaacttcgaaatgagcgatttgggaaggctcacatattatcttggcattgaggtcacacaagaagaagacgggatcatcttaaaacaagaaagatatgctaaaaagatccttgaagaagctggaattaatgagtgtaaatcaattcttgttcctatgaattctggtctagaactatcaaaagcacttgatgagaagagcattgatggacaacaatatcggagaagtattggttgcctaaggtacttactacacacaaggccagatctctcttactccgttggtgtcttaagcaggtatatgcaagatcccagagaatctcacggagccgccttgaagcaagtcctgaggtaccttcagggaactacgggttacggtcttgtatttaagaaaggcgataagactggattagttggttacagtgacgctagccatagtgtagacgcggacgatggtaagagtaccggagggcatgtgttttacctaaatgagtgtcctattagttggtgctcacagaaacaacaagttgtagctctctcatcttgtgaagccgagtttatggcagctacagaggcagccaagtaagccatttggcttcaagacctgcttgctgaagttattggaacaccatgtgagagagttacattacgagttgacaacaaatccgcgattgcactcacaaagaacccagttttccatggaagaagcaagcacattcatcgcaagtatcatttcataagagagtgtgtagagaatggtcaagtctcggtagaacatgttcccggagtcaagcaaagagcgaatatactaaccaaggcactcgcgaagatcaagttcaaggatatgagagatctcattggagttcaagacttgtcaaatgaagacttcaagcttaaaagagtgaa

>ATCOPIA40_LTR

tgttggagtaagcttgaagataatgacttaagttataagttatttaaatataggaatagattaaatcttaaggagatatcttaggagttaagttaggagttatctaaccctaatcctattgttatttggttttaaatctctatatatatgagtcccaaagttgtggtgaaccatatgagttttagagagattaagactttgaaattgtttaggtttgagttatttcctaagcttaataagagagtaattcttatatatctttgagttcttaatcttctttgaaactaga

>ATCOPIA41_IN

attggtatcagagctgacacctaacacaagtggtatctgttcaacaggtgagatcttgctacagggtaatggagaaatcacaacagtacgttacaatacaacagccattgaagttggatgctgagcattatggctactggaaggtgtctatccgataggctatccaaagtgctaaaatggatgcgtggttcgcagtggaagaaggatggaaagctccgatggtcatggatgccaaggggttagaaataccaaaatctaagaaggattggactactgaagagaagacagctgctaagaacaactcaacggccttgtcaatcatcttcagatcattgccaatgagtcaattcacgcatgtgcaagggagcacatcagcaaaggaagcatgtgacattctagaaacaacctttgagggaaccagtaatgtgaagaggaccagattggatattttagcgtctgaatttgaaaacttgacaatggagaatgaagagtcgattgaggaattcagcagtcggctgagttcaatttcctagtaaactgtcgtcttgggaaaaacgtacaaggacaagaagctggtcagcagtcggaaccaaggatttggataaacttttaaccatgggcagaacgtctaatgttacgtggggtcttggatacaatggaggaaatacaaaaggtgagactcagtttgtcaaaggatccacttcagacgataaatctcaggtcaaacccacaaccactgttcggaatctgtttcgctctaagcctaaacgagcatctcaggcttacaacttgagacaaacttacttcaaacctgagttcagaagcaagagaacaggatgctggtactgtggaggtctgagtcactacaaagcagactgctacaactacttgaaacatgttgttcagactaggaggaattatcaagtgggtgctcaaggacgacgagtcagacaagtgtatgtcaagaaagaacatctatagtgtcatgttactcaaacatcagcgaatgcagaactcaaggatctgatgtggtgctttgatagtggatgctccagacacatgactggaacactagacaatcttgctggatataaggatgtgccatctaggaaagtcagatttggagatggaggtcatgccttgatcaaaggaaagggatatacttccggacaagccttgcctcatctaaccgatgtatatcatgttgatggacttaaggcaaatctgatcagtatcagtcaactgtgtgacgatggtttgagtgttttctttacacaaacggaatgcaaagcctttgacaaacatggatttgtaaagatggaaggacaccgtgctgcaaataactgttacatgtggaatccagaagagtcatgctacactgcatcacagatcgatcaatctacttcagaagatcaatcgctagtgtctctgctagaatctgatcatatgggtgaagccatgagttcggttcaggagtttaccatatacacaatgagctggcaggctccattcaagttcacaatcaacaaggtatgcaattgtttagcttggtatctgaccagtctgtatggcttacttgtctcaggtggttggtgggaaggtcagctgatctcaggaaacaagcaatctcagttccagttcctcaaacacacggttcacgtcatctcacaaccaatacagtgtggaatctcattaacactagtgtcatgaatgagattcaagggggaagagtgatcttggattcttcgtgtgttaacaattaggaagatgcaatggagtgggaactgaacttacttaagtctgtcaagctattgggatctgatatctgcaatcagatcattgatgaagccggtgttgcaatccgtcttagacatctgctgttggctcagggctatattcggatggaagacgagtcaaagattgatacacatcttgcttgatgtgaatctatcaggtttctgctaggatgtgtgtgtgcaacacagttctatgtttatcagatggtactccagaaagggatcttgagtagtgaaccccagagggaatggtttgagagtcacccaagagcattcggtgttccgggacatccatactcggctcatagattgaaaacagttgtctactgcttaaagcagcttccggagaaaatgtgttgtatggctcgacaaatcaaacacttgtacaagcttttgttgaacttattaccagagagtttgaagtaagtatgtacggagttctgaggtacatgcagggcatacagattaagcaaactgatgagggcatatctatgtcgcagaatatatacgcaagaacaatgattgagaagttcaaactggatgcacaagagattgtcacaacaccgatgaagatctcgacaagacttactgcagatgagagaggaggagatgtaaatgttagtatgtatcaggggatgatcgagagtcttcagtacttgactgtgagtagaccagatatctgtcatgcagtgaatgtgtgtgctcagtatcaagtcaaccccaagatgtcgcacttgtcagctgtaagaagaattctcaagtatgtgaatggtataccaactttcgagctgtactacaccaaggacactgacaatagactgaagggatattgtgctgcggattgggcttgatctttggatgacatacggggatcacttagaggatgctattttgttggaaacaacatggtgtcttggaagagcatgaagcagaacagtcagcttctatcaactgctgaagcagagctaagttctttggagagttcgagttctcaacttgtacgactgaaacagctcatagaggagtgtggtatgatctctgattttccagtactgtattgcaataatcatagtgctatacattgtttccaagaacctgctaggaagtctcgtacaatgcacatagatcataaacataactatattcatgaattggttaaggagaaactaatagcaattgaacatgtgggtactaaagctcaactagctgatgtgtttatcaaacctctgatgtctaacaaattatgtactctacgaatgttcattggaatgtttgaactataattgtgtttgagttgtgctgatctagaatagggacctgaagcaagaacaagtcatggaaatccaaagatgcatagaggatcagcacttagcaaagtcatgtggaaaatagttgcatgtcatgccggttcaaatcagacgtaaaaagttgtcgtctaacaggaggttaaagagctgagaagatcaacaaactgaatgaacttggtacacaaaaaaaaaaaaaaatgtgtatcaagttcaccaggttcgaatatcaagcaattgagaagtcaagtgatattgctgatataacaaaataagggagtaattcaaaagaattcatatcatcacgcccctgtttagtcaaactatcttcaaaggacattccagctgcctgttacacgttgatctgattcatgagagctaaaggcatgacagtgtgaaagactagccactgaataatgagctgattcatcaacgatctgattggttatgacagtttcatgcggaagtcgtgaacacctgattggcacaaagcacattttcaagtactaggaagaggtaaactttgaaatcagtgttggaaagcttttaccctcatgtgtcaattagctgtttaggtcactaaactagttcaggctataaaatgttcaatgttgctctgaatgtgtttgaaatttaatcacgtgtttgattgaaaattctcttggcaaagaagcacttatgtgtttgttaagtgtttcagaatattaaaacagtttgggcccaagcctgtttcaagcccacagaatttcaaggaggagtgctcgagtattggagggaaaaagaggagttagggtttgaggagtcaaatcaatttcttatttggaagtcgagaaggcaatcccctgtgttcaacgcaatttcgagtgagatcgagcatgaaatcacttctctccggtctgcaacgcctggttcaatagaaagccgagtccgcatcttctgcagacatgttcggtgcacaatctagtgattcgttgccagagatttcacggccgactgcaacagaatgtcaagaggtgaaatccgatgaagcactcactgaacccaaccctgattgggctctagttctcgtcaaagacccatcgcctccactcatcatcgaggttgaggatgagactctgccttcaatcgacagtgttcagaaccctaaaccgaaggatcctgaggctaccccctcgcaaccctctgttgcatctcgattgcgcaagaggaagtcatctgctgcggatccacgcatcaaaaggatgaagcagggaaagggagttaccggttcatcttccttcgatggaattcgatttgtctctacggaggctgaaaagaggtatgctcaattctctcaacgaaatttcattgaggaagtagaactgtctaggaagacaaatgaggcagctagggagtttataaagcaggcaggactgattcgaacggttactaagttcaacccgttcactcagaatctagtgtttgagttctgggctaatctgcccactatgaaggtagacacgtatatggtcaaagtcttggtgcgcaatcgggagtatgagctctcacctgggaagatcaacgagatgtatggtctcccttctgttgatgctagacagcagcggatggatatcgctggtctggttgatgaacaagtggctgaatttctcactggtgggaaagtcagtgttctgagcaagcttcaggtgagtgcctttacacctacgagtttggaactgtttaaactctgctgctcaaattcgtctcccacatccaacgctgggtatgctcagcctgatcgattgtatattacatttctttgattgagtatttgatttttgactaagttctgtgctcaagcagggggagataaggccccttcggactccgttgatgctgggggagattaagttgtgggggagctgtgttgtttaagtttttaattgttataagcttcattgtatttcagaccaaaacagtttcttttgtgtttttgtttaaaccgatgattatatcatcgtaaacatgggtctgtaatatttttaacacaatgtttactttcagactaatcatttatgctagatgatgtttctagagtatgtgtgttatgtctccgatttgagtttcaggttatatcaataaaggagtgcttgagtatcaaactcagtcaaaaagggggagat

>ATCOPIA41_LTR

tgaagatgcagagttcaatcaatcagctgatggcgatcagagcgttgatggatttcaggaagctgcatcgagtttcatcagacgcttgatttgcagacatggatgcgagagcaaacgtggcaagctaaggcggatgatgtggagagccaaggatggaagctgacttggcgtggtgtcaaagacaagaattaccaagaagatatggaagatatcatctcgaagatttaggaaggaataagcttgaagaaaacaagaaagattccttaaaacgagattctcggagatcctaggtttagggttctggagagtaggtataagtattctagagtttagtgtgaggcgctttgagcactctagaatactacgatacgattgaaaacttcagagcattgcaaaagcttgaaggtgagtgtgtaattctctttgtcttcacgtgagagtgataagaaacagagaagagaattaggcttagatattcttagtcgggtttgatcttttgggtagaatagatcaagtaatctccgagataacatttgtaaacagtttgttatcaataagaatatagttataagcaattctctgtgttgtgttgatctctatacccttta

>ATCOPIA42_IN

attggtatcagagcggacacctgataaaagaatttgttaatcttttcaacaggtgagatcttgcgacaagggatggagaaagcacaacggttcgttgcgatacctaagccactgaagctggatgctgagcattacgggtattggaaggtgttgatcaggcaatcgattcaaagtatcgacatggatgcatggtttgcagtagaagatggttggacgcctcctaccacaaaggatgcaaagggagacattgtcttgaaatcaaggactgaatggactccggatgagaacacaacagccaatcacaactctcaggcactgtctgtgattcttggatctttgccaaggaataagttcactcaggttcaaggatgcttatcagcaaaggaagcatgggacattctgcaagactcattcgaaggcaccaacaatgtgaagcgtactcgtctggacatgcttgcgttagagtttgagaatctgaccatggaagttgaagaatccgtggatgacttcaccggcaaactgagctccatcacacaagaggctgttgtcctaggaaaaacgtacaaggacaagaagatggtgaaaaaatttctcaggagtctgccagacaagcttcagtcacacaagtcagcgattgatgtatctctgaactcagatcaattaaagtctgatcaggtcgttgggatgatgcaggcgtatgatactgattcagtgaacgatgagcatggattctctttggagaaagtcaggattctaattgaggagttgattctgaaaaggaaggaaaataaggagctcatttctgaaaagggaatcttgatggaaaaagtttctgcacttgaaaaggagcttggtgaagagagaatcaaatcccaaggactggagaaacagctagaagaccaactgagaaacatcaagatgctgagtagggggactaaagacttggataagctcctgactgttggaagaacttcaaacgttacttggggtcttggatatgatggaacaagttcaaaaggaggaacgcgttttgtcaaagggacaacttcagatgagaaatctgatgatatccaaccagcagaagcacaccgcacggatgcaccttcgaaggctcgaaaggcgttgaccaggagtatgcctactcataactggagacagtcagattatcaggttgatcacaatcatctgaggagcagaagaacaggatgttggtattgcggaagtcagaaacactacagagctgattgttacagcttcctgaatcgtgttacgcaagtcaggcaccacaagcagcaccacaagaacgataagcaagggaatcaagtctacataaagaaagatgatctttatcgtaatggtggatattcatgtacctcaattaaggttatgaatagagctgttcttgttaacaactttgctaagtctggttatgtgaaggcaaggacgagatcagaagcaagagttagtcagtcgagtgtcaaacagctgagcagaagaacgaggaattgtttctgtagtgaccaaggacagattacagctagttgtaacttgtgttacaatcgagtaactaaattgctgaagcgaaacaaatatcacagcgatatttgtatatccaatcgagattggatgaagaaacctaatgtttgccgtcatgttgctgataaacaaggacgtacgaccctcaaaagtgtgcgaactggtgactgctgttacatgtggaatccatccaatccacattcaaagcagtttgttcagagaggtgttctgaatccgaagtgcctacatagccaggacttggccactagtagcaaggtgcagcagtctggtaagtgttcgaatcatatcccacatgatgctatgggaaggattcggggggagaaagttaccaagcctgctggtggcggatgaatcaggaactgaatctgatggtgtttgtacatatgtctgtacatatctggagtcaaagttgatgagtctctgtacatatcaggggagttgcagtgcatggataccattacaagagtctgatgcagtgcaagatgctgactggactggatccgtggaagattgctgaagtacaagtggttgatgcttcttcatgggtagcaacatggtctcgaggtagagtaaggagcagaatattgtgtctctatccattgctgaaatagagtactttgctctagggagttgctacactcaaatcatgtggatgaaacaaatggcagctgactatggtatgatctctgattctttactaatttattgtgataatcagagtgcattaaacataggaaataatcttgttcaacattcacgcactaagcatattgatattttacaccatttcattcgtgaacttgttgaggcgaaactgatagtagttgatcatgtgagtactgaatatcaactatctgatttgtgtaccaaaatcttggagtttattagcctcagtgatctgagaaagttaattggtgtgcgtgagatctaatctgtttcgtgagatgtgttgatctaagacaggaacaaaaaatgcaagaacaggccatggaaatccaaggatgaatagaagatcaacactctgtgaataaaggtgtgtggaaaatagctgcctgtcatgccgattctgtagtcagccgtaaaacgttgtcgtctaacaggaggtcaaagagctgaggagatcaatttattgtgcaaaccaaactaagggtatctaaagcctaatttggtgtcaagtgaccatcatgcactgatatcagcagttgagaagtcaagtgatgctgctgatatgaaactgacagatcaacagggattcaaagtttaaaaaaagaaaagaagaaaaaggagaaccctgttgtatctaaatctgaggaagtgtggaaataaaagcagcacactcctgattctttgagcaatctccaaaggaaaattccagctgcctgttacacgttgagccgatctactggaattgaaggcatgacagtgtgaaagactagccactgtacccagagttgatctatcaaagatctgagtggttatggtagcttcttgctgagttctgaacaactggttggcacggaacacggaacagatctcaaaggtataaaaatgttttgaatccaacgtagtgtactcacatgtttcaataactttctgggtcactaaactctgttcatgagtgaatggttgttcgctaaatgatttttaatctgaactatgctaacatgtgatgttgatcaatgggatgatatgaatcaatgggattatttgcaagtgtgcactaatgtttttgtttagtgtttcaggctctaattattttaggtatagcctaagcccatttgttttgaaggcccatgaacaaattaagcccattacttaatgtctaggttggagggaaaaagagttagggtttctcatgtcaaatcaaacctgcctagggagtcgttaactcgaagacttgtgctcaaagagaatcaagttgaaatctttcttgaagatgaatgcgattttctctggacttcaacggctcgtgcaacaaagatctgtgacaccgtcgtcgaacctgtcagaagcagcctctgaggagcatccatcgcagtctctgaaagctgaagaggaagaaaatctaaacaaagctatggtgttgtatgctgagcccgaaccaacatctctcagatctgagcttccaaacaaggagaaccaagatgaacaggcagccgtatctccagagccaccggtgattgtggaactgcctgacgagtgtgaatctactgttgttcatgtgcatagatctgagacacccattcaagctgagaccagaaaccagacttgtgaccaagttccaccactcaacaacgagaatcctcaagtggtgcagatctctgatgcgtctgaatccgcacaagctgttgactcaacagatctgtctgtgtcctcgcgtctgctgaaaaggaaacagtctgctgttgtggagaggatgaaaagacagaagactaacgaaaggaaggaagctgctggttcaagtgcttgtggagaaattggtcttcaacggcttgcgacttatcactcatgatgctaagaagaggtcgaagacgattctgtcaaggtatgtgtgcgaggcatggagtacgaattctctcctgccaagatcaacgttttgtttggtctgcaatcagttgatgccagagctcaacagatgcaaattgctggtctgatggatgatgaggtcaccagctatctgactgatggacaagtgaaggttcttcagagtcttccgatgagcaccttctcgaaaaactgtaggaagctgttcaagttctcgtgcagaaattggtctccaacaaccagcgagggatatgcaagtacagacagggctttgcttgtgtatcagattgcacacaagttggcttttgactttgggaagatggtgtatgagcatatcatgcagcttgctttgaaacccgaggcaaagttctacattccgtttccgagtcttgtgtatcaacttcttcagatgcagcatcctgtgaagtttcatgttgagaagccagagcccttagtccagactaagaagaagactgcaaagaagccatcaacacaaggagtgcaaactggtgactccactaatggttcaggacatcgcagagcaatgaaactggccattgaagttctgcagactgctttagatgcaggtaagtgtttctctgttttcctctgaaatattatatctctaatgatgtttgtgtgatttttgactaagtcatgcacttaagcaggaggagatgtgtctgattctgatgatgatgggggaaattaatttgtgggggagcttgatgtttttaacgtttaaaatctagtttttgacaagcttgtttttatttcacaccatgcttttgtttttgttctttttgaactgatgatatgtatcatctgaaaacttgggtctgtaataagttaaacacagctgcttggacgaatcttatcttttgatatgtatgctagaaacgtttctagattttgtcttgttgctgtgtttgggtttccttctgtttgactttcaggtcttgtcagggaga

>ATCOPIA42_LTR

ttgagtcacacggctggtgaaakcaaacccggtttggctgcagtagtctgagaagtggttggtcatcaaagtgttttacgatacactratwtcaggtaagtgtttctaactacttatcytgatgtagatcttctgtaactagttgtttttaacttagttgtgcactcaagcagggggagatgastctggtttatagaatgcttgattgtgggggagtttgacwractgctgaytacttagtaatgttcatagttgactgatyacttgatragtattgttgactaattactagttgagtagtgttcactgtttatgggggagacttgtttsaatttrattcgttttaagtttagacatagtttcactgcctgktrttrgaggagttcgttgttagaatycttctgagtgatgtgaacggttaagaacttgatttycgytgctattgatkttttgcgttcttaacacagttctgatatgaacaagctttwatctctagctagatattgcttkttctgtttctttttggttggtttgtttagtatcttttyaggttgarccagaaggatgcttgagtatcaaactaagtcaaaaagggggagattgaagatgcagattctgacaacaacgagctgatggacatcagggagctgttggatatcaggaagctgcatcaaatctacatcaaacgtctgagttgctgacatggacgcgtgagctgacatggcggagctggagtggttgatgtgacgtgccaagaagctttgctgacttggcrtggtgatcaaagacgaaagtcggttgagaagatttggtgaatattcttctcaacaagttaaggaaagagataggcttgaatatcaaggaggatctcgaacaaggaaagagataggcttgaatatcaaggaggatctcgaacaaggaaarggataggcttgacgattcaagaaagatyccgctcattgattctcggagatcctagggattagggttagcgagaatagcataaatattctaggagtaggcattgagccgcctaagcactctagaatattgccatacgtgtaaagctttcgaagcctagcaaaagcttagaaagtgggtgtttgtaatccctcctcttcacaggcgtgtgataagaaacagaggagtgattaggcttagatttgttcttagtcggggttgatcattcgggtagaataggtcaagtaattgccgagataatagaatccttgtaaaagatttattatcaataagaacaagtaaaaarcgtttgtttgacgcgtttcaaacaaaatcagcgtgttgtgtatcctctataccctctta

>ATCOPIA43_IN

attggtatcagagcgggtaaccaatctgagattagtatatctgaacaggttagatcctagttatggaaccaacggatgttagtactggtacaggcaaggtattactgctagatactaagcggtatggatactggaaagttcgtatgacacaaatcattagaggccaaggtgaagatgcttggactgcagtagaggaaggatgggaacctccgttcgatctaacagaagatgggttcaaaatcactaaaccaaaggcgaattggactgcagaagagaagcttcaatcaaagtttaatgcaagggctatgaatgctatcttcaatggtgttgatgaagacgagtttaagcttattcaagggtgcaagtcagcgaaacaagcatgggatacgttacaaaaatctcatgagggaacttcgagtgtaaagagaacaagactggatcacattgctactcagtttgagtatctcaagatggaaccagatgaaacaattgtgaagttcagttcaaagataagtgctcttgcgaatgaggctgaggtcatgggaaagacctacaaagatcaaaaattggttaagaagctgttacgttgtctgccaccgaagtttgctgcccacaaagcagttatgagggttgcagggaatactgataaaatatcatttgttgatcttgtgggaatgctcaagtcagaagaaatggaagctgatcaagacaaagtcaaaccgtcgaagaatattgcatttaatgcagatcaaggctctgagcagtttcaggagattaaggatggaatggcactgctagcaaggaattttgggaaagctctgaaacgtgtggaaagaggtcagaatcgtgacagtacatcctggagcaataaagatggagagagatcacgtggaagattctccagatctgagaatgatgactcaggaaagaagaaggaaattcaatgctatgagtgtggtggttttggtcacattaaaccggagtgtccagtcaccaagagaaaagaaatgaaatgtcttgaatgcaaaggtgtgggtcacactaagtttgaatgtcccaacaagagcaaacttaaagagaaatctcttataagtttcagtgattcagaatctgatgatgaaggtgaagagctgttaaacttcgtggcgtttatggcaagttctgactcaagcaaggttatgagtgacacggattctgactgcgatgaggaactgaatcccaaggatgaatacagagtgctatatgacagctgggtgcaactaagtaaggacaagctgcagttggtaaaggaaaagctaactctagaagcaaaacttgctaatgtgagcacagaggataagcagaaactgagtggaatcactgttgatggaaattcacaggactactatcaaaagaaacttgactgtcttcaggaagagtgtcacagggaaagagatagagctaaacttctggaaagagagttgaatgacaaacacaaacagatcaggatgctcaacaaagggtcagaaagtctagacaagatcttggcaatgggcagaactgattctcaaccaaggggtttgggttatcaaggttatacaggaaagatcaacaaggaagaaggaagagtcatcaactttgtcagtggtggttcaacaagtgaaaccgtggtgagacaaagctatactgaaccgaagaaacaagtgaagtcacatgttgaaacaaaaggagaatctgttgtgagaacaaggatggtgggtgtgatttgctgcgatcattgtggtaagagatttcatatgagagaacagtgttacaaattcaaagagaaagtcagaacgctgtggaatttagcaaagtgctacattgagccatcaagattctgcactgtgtggattaagaagaaagatctgtatggtgatatggaagctgaaaggtacaatcacaagttagaaagaatgtacgaagaagaggaaatgcattacgccagttgctgcaatcaggtcaacaaagaaatgcaagacgaggcaaatcttgtttgcaactggaatctttctgagttggatgactgcactccacaagccaaagtggcttatacttcagctgtttctcaagacaacagagcgtggtatttcgacagtgggtgttctcgtcatatgaccggtgtacaatctgttctaaatgacttttctgtcatcaccaatgggaaggtcacttttggagatggtggaaaaggaagcattaaaggaaaagggaagatcgagatagatgatcaaccgcatctgtcaaatgtgtactttgttgagggactcactgctaatctaataagcatcagtcaactgtgtgatgatgacttaactgttacgtttacaaagactggatgtgttgcactcgatattgctggcaacaatgttctatcaggagtttgttcaggcaacaactgctacatgtggaaagactctgaagtctgtttgtctgcaatcacatccaagcttgatctgtggcatcaacgacttggacacatgaatactcaaagcttggtcaagattgtgaatgctgatgttgtcagaggaattccaaaacttgaaggcagtacaagtgttgtctgcaaagcttgcagccaaggcaaacaagttaaagtgcaacataagagggccacgcatattggaactactagtgttcttgaattggtacacatggatcttatggggccagttcagacagaaagcttgagtggtaagaagtatattttggttctggttgatgactactctcggttcacatgggtgagatttctaagagaaaaatctgaggcagctgaaagtttcaagattctggctttacaacttcagactgagaaagggaatcttgttcaaatcagaagtgatcatggtggagagtttcaaaatgaagaatttgagaaattctgcagaattcaaggaatcagacatcaattctctgcacctagaacaccacaacagaatggtgttgttgagagaaagaacagaactttacaagaaatggctagagctatgattcatggaaacaatgtgtcaccaagattctgggctgaagctgtcaacactgcttgctacatcgtgaatagggtgtacgtaagacctgggacaagcacaactccttatgagatatggaagggaaggtcaccaaatctgtgctacttccatacttttggttgtgtgtgctatgttctgaacgataaggatcatcttggaaagtttgatgcaagaagtgatgaagggatatttctgggttatgccacaaacagtatggcgtacagagtctacaataagagattgaagagagttgaagaatcagttaatgttgtgtttgatgacaagcatcctacaagactcttcacggtggaacaagatgatgatgagcaagtagaggaaacaagacattcaacatctcttgttgaatctggaacttcaaaagatgttgaacagactactgaaccacgtacatcatcatctcgcctcacagttcccaaaagtcactcagaaactgacgttattggagagttggatggagacagagttaccagggggataaagatgaactacagagatatgattctgtttacctgttttgtgtcaagcattgagcctaacaacattgaaattgccttagaagatgaattctggtaccaagcttgccatgaagaactaaatcagttcagtcgtcatgaagtatgggatttagttccaagaccggttcatgtcaatgtggttggtactaaatggatttttaagaataaaactgatgaagaaggcaatgtgacacgtaacagagctcgactggttgctcagggatactctcaggttgaaggaattgattttgatgagacttttgctccagtagctcggctggaatctattcgtcttcttcttggaatatcgtgcttgctgaaaatcaaactgtttcagatggatgtcaagagtgcttttctgaatggagttattcaagaagaagtgtttgtgtctcaacctaaaggctttgaagattcaaactttccagatcatgtgtacaagcttaaaaaggctctttatgggttgaagcaagcacccagggcttggtatgagcgtctcactctgttcctaatagaaaaaggtttcaaacgtggaagtgtggacaagactttgttcatccttgttgatgaaaaggacatcctcattgtgcaaatttatgtggatgatattgtgtttggaagcacaaaacagaaacttgtctctgactttgtggaatccatgaccaaagagtttgagatgagcatggttggagagatgaactacttcctgggtctacagatcaaacagactgatgaaggagttcatatctctcagtctacgtatgccaaagggttgattcagagatttgggatgcagacagctaagacctcaaaaactcccatgagtgctactgctaaactgtctgcagatgaagctggtctgagtgttgatgagaagctgtatcgtggaatgattgggagtcttctgtacttaacagcaagtagaccagatctgtgtttcagtgtgggtgtgtgtgcccgttatcaggctaatccaaagcagtctcatctgaatgctgtgaaacggattctcaaatatgttaaaggtacaaccgatgttggtctgttttactctaaacaaactaatcaaaatcttgttggattctgtgatgctgattgggcaggaaacctggatgatcgaagaagcacaacaggagggtgtttctttttgggaaacaacttagtttcatggcacagcaagaaacaaagctgtgtatccttgtccactgcagaagctgagtacattgcactagggagctgctgtactcagttgctatggatgaaacagatgcttctggattacggtatgacatctaacaccctgcttgtgtactgtgataatatgagtgcaattaacatatcaaagaatcctgtgcaacactctcgaacgaagcatattgacatcagacaccatttcattcgtgagcttgttgaaaacaagattgttgagatctctcatgtttcctctgaaaaacagttagctgatatatttacaaaaccattggatttgaacagtttcttgaatctgaaaaaatctattggtctgagtgaatactaatgtgtcttggtgttaaagctattgcaataggttcctttgtacagagttgcattagctttgtgttttcatcaatatgtatatatatatatctattgtgtctgtctttctggcacttcatttcaggaaaatagctgctcttcatgctcttcctcaatgaagtttccaaaaaggatgtgtgtgtggtgtgtaataataataaaaaaaaaggcaaaaagaaaaaaaaaaaggcctgaaggaatgaacaaatgaaagttcaaaaaaaaaaaaaaacaaaggttgtgtgttggcaggaaagggattggtattctttcttctcttggggtatatatgtatctatgagtaccccctcaaaacattgttcttcattacaggctctaccaatccccctcgccgtgttacccacacacatcatgaggttgcttctttggagagtatgacgagtgtagactatcctgtcttgatcagtgtgtatggttcatctcactgggccaggaagctgctattttctccatactaaaagtaagctatgcacctctgtactttggaatctgtccttctcgcttggatgcatttcgcatgtgggttccttgcctagtagaagtttctttaagctatatagtatatatgtctatatcttaagattatgctatgttttatctttcagaggatgacttgatgagatctcacattcagtttgaatcacagttcttgaatgacagtggctgttaatttcaagtgttgatttggttgaatttgtggttctatatggtttagtcctactttatgttgcaatcatattatgtacatatgttggctgtctactacgtctctgggtttctgttgtgatttgtttttgggcctggtataaggccctgtcgattgtttaacggatctggtttagtgttttgcagcctgtcttggttttcgtgctgtttttagggtttcccttgatgcgctcgtgccttttaaacctagcagctctcacagaaggctcattctccttctctgctactcaaacctcaaaattcaaagggtttctttctctaaaaacacaagtcaccatgaatcaagaagcgacctcgttgactggagtctctggtgctggtcagccttccgctgcgactccttctgaccctccaccgcctccggttgctccaatgttcattcccaagactgaaccaggcatctgcatgtctaagaaaaccaagtccaaggtctctgctccgactcagcgcaagagacagtgtacggctgcatctgccaagtccagatctaaagcgcctgctcctgctctccgacgtctgtcaagcaggaaatccaaccgatctgcctctgctgctggacaaactgaagaacttgtccgtgactcttcagatctgcaagatggtgatgtcaccgagattgctccgcctctctttctgtctcgttaccgagagaaccgtcggcttcttgctgttcgtgatcgtaagtatccggagctcaaattcccatcggagactccctacagtagctgttttctgactgaggaaggtcttgggcgctacaaggtcatcgggaatcgctgcttcaacgacatgcgtttccttcctctggatggaaacaacactgcgagtactcagcaacttctcttcaatgctggtctgcttcctactgtcaccgaaattgactcttatgtgcatgaggttgtcatggagttctatgcaaaccttcccgatggtgaagaaggtgatcaacttgcctactccgtgttcgtcagaggcaacatgtatgaattctccctggctatcatcaatcaaatgtttcagcttcctaatccttcctatccgctggatggtatgtctgagattcaagttcctgagtccatggatgaagttgcgattgctctgtcaaacggcaaagccaactcctggaagatgctcacttctcggctgttatctcctgagctagctctgctcaacaagatctgctgtcacaattggagtcccactgtgaaccgctctgttctgaagccggagaggatgactctgctttacatggtggccaaagctctaccgttcaacttcggcaaactcatctttgatgaaatctgggcgtgttcgaatgcagttgcgaacccctcctctactctccgacttgtgcttccgaacctgattgatcaaatgctgcgctttcagcgcattgtccgctctgatgaaggagataccacttcgtctgcaccgttgaagttcaccatggaagtgaaacctgttcctgtgctacagtctgacagtccaaccttggatgcagctctggagaccctcattgcatctctcacaacgatgcgtgttcgtctggcaggtaagcctctctcactcaacttacactctgttatcttttgagaatggttttttatatgagtgtgcactcaagcagggggagagtattctgatgtgtcgtactctgttccggatgatgtgggagatgagaatgtggaggaagaggacgatgatgatgagttagatgacaacacttaattgcttagtcctggtcgttttttagttgtgcattgtgttagggggagtttgtccctgattgtttttgtgtgtttgtgaaaactccttgatcttgttctttatcggcttcttatcggatcttctgtgtgtttaatgattggtcttatgtatggaaccaagaactcatttctgtttcatgtttaaatggttatgtcttttggcagtagttgcttaaacttgagcctttggtttctatgtttggtatggaatattgcttggcttgtatgtgtggtctctactcttttgcagattacttgaggtgtcacactcagataaaaagggggagat

>ATCOPIA43_LTR

tgaagaaacagagtgtctgaagttctacaggcgtgtctgaaacaacrcagagcagaacagaggaacagaggaagcgggaaagtagcagttgcttttgcsagcgtggcatgttgattggagtgaaggaaaggcgtatcactttagacaaaggagacgaakkcgtcagacataggacgtgtgcctagtgtcaagataatgatgaggagctacacttgaacctgctggataaagtrgagcatcctgycttttccgagaaactacataagagaagttttggtctcttattagggttagccactttacttgagaaaagtggaagagaagaaaagttaagtagaacttgttcttgtgggtttcattggtgagtgaacaaagagagcacacggctgttgctgtgtttgtgagctgggtagtttttaggttctgacagtagcaaatcaggttgttgatgttgcataayactgtccaaacttatctgtgattgatcagtttgaaagttgttctaataaagtgtgtttattggagttgtgttctttatcttgtcaaagtacttagggttcttttacttca

>ATCOPIA44_LTR

tgttgttggtttatatctgattcaaaatctgaagttggtttggttaagtcaattgaagattgaagcagtcggttatctctggttaagggatttctgattaaaccggaaattgaagaagagaagtcaaagactctaactgcagtggagggtttgactattatataaagagtaacagagtctgttacgattatcatcttcttcatctgttttcttctttgaagttttcttgtatcgagagagagagaacttgagagaatcaccattgttgtaagctcggtttgagctgtactcttcataaagcttagtggatttgccggattgattcggctccggatgtaggcttgatcacactgatcttgctgaaccgggtaaacaaatcgtgtgtctttgttcttcgttttctgttctctgatcttgcataagttgagatcgatcattcgcaacatcatttcgatcgttcttaacgctgaattgaatctaatcggagctcaatttcgtaaca

>ATCOPIA44_IN

aagtggtatcagagccttggtttctgaattgaggaggtttgatgcttcgatagcgttaatggcgatgtcgtcaaaggtagagatcaagacatttaatggagatagagacttttctctatggaagattcggattgaagcacaacttggagttctgggtttgatgaacactttaacggattactctttgacaaagtttgttccagtcccaaagagtgaaggaaagaaacctgaaaccgatgaagaatcatctccgactgaagaagttccagatctgatcaagattgaacaatcgaaacaagctaagaacatcataatcaatcacattactgatgcggttcttcttaaagttcagcattgtgtatctgcagctgatatgtgggcaacgctaaacaagctctacatggaaacatctctgcctaacaggatctatactcaacttagactttactcattcaagatgcttgaaacaatgagtattgatcagaacattgatcaattcttaagaattgtggctgaacttggcagtctgcagattgtagttgctgaagaagtgcaagcaatcttgatcttgaattcattgcctgtgagttatatccagttgaagcacactttgaagtatggtaacaagactctctgtgtgcaggacgttgtatcatcagctaagtcattggaacatgaacttgctgaatctaaagagtctgaaagaggctcttcaactgtgttgtatacaactgaaagaggtagaccccaaaacaggtctcagcagcaaggaaacaaagggaaaggcaggagcagatctaattccaaaacaaaggtcacctgctggttctgtaaaaaggaaggtcatgtgaagaaagattgttttgctaaaaaaagaaaactggaaagtgaaggtccaggagaggctggtgttatcattgagaaactagaagtttctgaagccttaaacattggtgacagattggtcaaggacatgtgggtactagactctggatgcacatcacatatgtcatcaagaagagactggtttagtgattttgaggagaatgatggcacaacaattcttcttggtgacgatcacacagttaagtctcagggacaaggttctattcggattaaggcaaatggtggatcaatcagaatcttgaagaatgtcaagtatgtgcctaatctcaggcgaaatctaatttcaacaggcactctagataaactgggatatcaccatgaaggtggagatggtaaagtgagataccataagaacaatgcaactgcattagttggacgtttaatcaatggactgtatgttctggatggagagaccattatgtctgagagctttaatgcagaagacactaaaagcagtactgaattatggcatagcagacttggccatatgagtttaaacaacatgaagatactggctggaaagggactgctacaaaagaacgatgtcaaagaactagagttctgtgagcactgtgtaatgggaaaatccaagaagcttagcttcaatgtcagcaagcacatcacagaggaagctctaggatacgttcatgcagacctatggggctctccaaatgtaactccatcactcttaggtatgaaatattttctgtctattgttgatggtaagacaagaaaggtttggcttatgtttcttaaatctaaagatgagacatttgaccgtttttgtgagtggaaagaacttgttgaaacacaggtgtgaaagaaggttaaagtgctcaggacagataatggattggagttttgtaattccaaatttgaagattacctcaagaagtttggtattgaaaggcacaggacatgtgcttatacccctcagcagaacggtgtagcagagagaatgaacagaactcttatggaaaaagtgagatgtcttttgagtgaatcaggtcttgaagaaatattctgggctgaagctgcttcaactgttgcatatctggtgaacaggtcacctgcttttgcagtggatcacaatgtacctgaagagttgtggttaaacaggaaatctgggtacaagcatttaaggaggtttgggtcagttgcttatgtacaccaagatcaaggaaagcttaaaccaagagctttaaaaggtgtgttcctcggttatccgcaaggcactaagggatacaagatctggctcttagaagaaatgaaatgtgttatcagtcgaaatgtgatatttcatgaggacttggtgtataaggatttgcagttaaaagagaagtctgaacaagaagaaagagcagagaagattactcaagcagaaaagactgtctctgaaatagtaagtaaccaacagcaggttggtgagagttctgttgcaggtggaacagttgatgtttcatctagtgatgatgagtcagagtattttgaacccgaaggagaagctccagcaagcagtgaaagactgagcaattatcagttagctagagatcgggttagaaggcaaatcagagcacctataagattctctgattactctcaatttgcatatgctcttatggcagctgaagatatggacagcagtgaagaacctagctgttatcatgaagctaaagaaaccaaagagtgggaaaaatggaatgcaggaatgggagatgaaatgcagtcactattgaaaaactatacatgggatatagtagaccatcccaagaatcagaaaatcatcagttgtagatggctgtacaagaagaaaccaggaattcccggtgtggaacctgaaagatacaaagccagactagtagcaagaggctttactcagagaaaaggaatcgactatgatgaagtgtttgcacctgtagtcaagcatgtgtcgataagaatcttgatgtctattgttgttcaagaagatctagaattggaacaaatggatgttaagactgcgttcttgcatggtgatctggaccagccactttacatggagcaacctgaagggtatgttgctgatgaacaaaaggatcaagtgtgcttgttaaaaaagtcactctatgggttaaaacaagcaccacgtcagtggaacaagaagtttaactcttttatcatggatcagaactttatcagaagtggtcatgattcgtgtgtttacataaaacaggtgagtgatgaagagtttgtgtatatactgatatacgttgatgacatgttgatagcagctaagtcaatgactgaaatcaacaagatcaaagaggcgttgagtacaggatttgaaatgaaggatatgggtgcagctagtcgaatactgggaatcgacattataagagacagaaaagcaggtacattgcggttgtctcagacaggatacttagagaaagtgcttcacatgtttaatatgactgaagcaagacctgtaagcacacctatgggagcacatttcaaacttgcctcagtagttgaggaagaagagtgtgtggacactgataaagttccaaactcaagtgctattggcagcatcatgtatgccatggttggcaccagaccagatatagctcaagctattggagttctaagcaggtttatgagcaaaccaggtaagattcattggactgcagtgaaatggttgctaagatacttaaaagggtctacagatttgaatctggttttcaccagagaaaaggatttcagagttcaaggctttagtgactctgactatgcagcagaccttgacaggaggcgttcaacaacaggttatgtattcactgttggtggaaatacagtaagttggaagtcaaatctgcagagcatagtggctttatcaaccactgaagcagagtatgttgctttaactgaagcagtgaaagaagctttgtggattcaggggttgttaacagaaatgggattcaagcaagagaaggttactttgtggtgtgactcacagtcagcaatcagtttggcaaagaacaacacgttccatgaaagaactaaacatattgcgatcaagttcaactttatcagggatgtgattgaagaaggaagtgttgaagttcttaagatccatacttctcagaatcctgcagacatgcttaccaaaggcattcatgtgcagaagtttgagtcagctttagagtttctaaagctactcaggtgaggtggaaagacatctcagccgaaggtgaaatccaagtaagtgcaagtccactagttatggagagatttgaatcaaggtggag

>ATCOPIA45_LTR

tgttggagtaagcttgaagcttacttgaagaagaagcaatcaagcaaaaaggaaattagaattatactttgtgtttaggaagttgcctaaatacttatgtgtgttttcctaataggattaggaaattgtctagttcttttcatatataaaggagttgcaagagtgttgcaaaacttatgagttgagagattaagattgtgtgtttttaggttttgagttattttcttaaagcaataaaagagagagtcttttattcataatcttgttcttattcttgagtttgatatatagtttgaatcaata

>ATCOPIA45_IN

attggtatcagagcataatattgattcaagcatctttacgatcaaacaaaggcaatcaacgaagttagcgttgattccgtacggagaaagaaggtaacttgtgaagaagacatgggcgatatagtaccagtaacgaacaacaccaaagaaggtagcagctcttcctctattcaatgtccgatgcttacggcaacaaactatacgttttggacaatacgtatgacgatggctctaaaagttcacaaggtatgggaaacgattgaagaaggattagatgatatcgataaaaacaatatggcaagcgctctccttctccaatctatccctgaagcattaaccttgcgagttggaaaacttaaaaccgcaaagaagatatgggatgcgataaaggctagaaatctaggagctgatagggtcaaagatgcaagacttcagacattgatgggcgagtttgaaaggataaagatgaaagaaactgagaagatagatgacttcgctggaagactctcagaattgtccaccaaatcagcagatcttgggaatgatattgaagaacctaagttggtgaaaaagtttctcaatagcctaccacggaaacgttatatacatatcattgctgcactagaacaagttcttgatcttaatacaacaagtttcgaggatatagtcggcagactaaaggcatatgaagagagaatctgcgatgaagaagataaccaagatgatcagggaaagcttttgtatgcgaactctgaagaaaagtcagctcagaacaattggaatcccaataaaggaaaaagtcaaggtggtcgaggatacggacgaggaagaggcagagatcggtttgggaatgcacaaggcaacagagacatgacaaacgtgatttgctataggtgtgataagctaggacactatgcttccgactgtcccgatagattgcttaaacttcaagaaacacaagatgttaagaatgatgatacacaaaaggctgacgcattaatgatgcacgaagtagtgtttcttaacgagaagaatgtaatgccaaataaacttgaggcgagcttggatgttgataatgtgtggtacttggataatggcgcgagcaaccatatgacgggaaacttggcttactttggtgagattgacgaaagagttacgggaaaggttcgttttggtgacgattctcgtattgatatcaaaggaaaaggctcgattacgtttatagctaagaatgaagagagaaaaatcttggctgatgtctattacattcccgatttaagaagcaacatcgtgagtcttggtcaggctaccgagtccggatgtgatgttagaatgcgagatgaccatctaactctatatgatagagatggaaagctgctaataaaagcgacaagatccagaaatcgactttacaaggtgatcatggaagtcgatgatacaaagtgtttgcaacttgagagcttgagtgaaacgacaaagtggcatgcaagattgggtcatattggaactgacaacttgaaaagaatgatgcaaaaggaattggtcattggcattcctaatatcaaagtcgagaaagaaatgtgtggctcatgcttgcttggtaagcaagctagaaaaccatttccacaagcaactccatatcgtgcaactagcatactcgagctcttacatggagatctttgtggaccaattacgccttctacagtagcataaaataggtatatctttgtcctaattgatgattattcacgttatatgtggtcacttcttctcaaggaaaagagcgaagcgttcaataagtttaaaagctttaaggcatgtgtggagcaagaaactggtgctaccattaaaacgtttcgaacggatagagggggagagtttgtttctcaagagtttcaagcgttttgtgacgcctccgggattaaaaggcacttaactgcaccgtattctccacaacaaaacggagtggtcgagaggcgcaatagaacgttaatggaaatgactagaagcatcttgaaacacatgagtgtcccaaattatctatggggagaagcagtgagacattcgacttatcttataaacagagtagcgacgagaactttggtagatcaaaccccatacaaagtcttaaagagtaagaagccaaatgtggagcatttacgggtctttggttgcattggttacgctaaagcagaggctgtacatttgagaaagttagacgatcgctcacgaatgctagtacacctaggaacagagcctggatcaaaaggctatcgtttattagatccaacaaggagaaaggtgatagttagcagagatgttgtatttgatgaagaaaaaaggtggaagtggaataatagtgaagatgaaattaataatgttccaggaatgttcagtcttagttttgaagaatttggcaacaatggtataagagaggaggatgatatcacagaagaaacagagatcaatgatggagagaatcatgatcgtgaagctgaaattcctactcaagcaatagagactgtagaacaataagtagagccgcaagtcacactaagaaaatccgtgagagtgatctcaaaaccaagttacttagacgattatgttttgttggcctcaatcgaatgtgaacggctcttacttatgataaatgaagaaccatgggactacaatgaggcaaaggaattgcaagaatggaagaaagcgtgtgtagaagagattgcgtcgataactaagaaccacacttgggatttggtggatcttccgatcggagctaaaccaattggactcaaatgggtctttaagttaaaacggaactctgatggaagcgttaacaaacacaaagcaaggcttgtagctaagggttatgttcaacgacacggtatcgactttgatgaagtatttgctccggttgcccgaattgaaaccgttcgacttatcattgctttagcagcttctaatggatgggaaatacatcatcttgatgtgaaaactgcatttcttcatggagaattaaaagaagtggtctacgtttcacaacctgaaggatttgtgataggaggaagtgaagataaagtgtacaagctgaacaaagctctttacggactcaaacaagctcctagagcatggaataacaagttgaacaagattctcatggaacttaagtttactaagtgctctaaagaaccgtcattatattgtcgaagagacaaggatgagctacttgttgtggtggtctatgtggatgacctacttgtcacgggatctaacttacaagtcatacttgagtttaaagaagagatggcaaagaagtttgaaatgagtgatcttggaaagcttacgtattacttaggtattgaagtgtttcaacatgaaggcggcattatgcttaaacaagagaggtatgcaaacaagatcctagaagaaacaaaaatggatgattgtaatgcggttcagattccgatggatgcaaacctaaagctaagtaaagcacaagaagagaaaaacatcgatgagaaggaatatagaagaaatattggatgtcttcgatatttgctgcacacacgtcctgacctttcttattgtgttggagtgcttagtagatatatgcacgagccaaaggaatcacatggagcagccttgaaacagattcttaggtacttgcgaggaactcaatcttttggtctctgtttcaaacgaatgaataaaacagagctagtaggatttagtgatagcagtcacaatgttgatgaggatgatggaagaagtacgacgggtcatattttctatcttaatgactgtcctatcacttggtgctcgcaaaagcaagaaactgtggctctatcgtcttgtgaagctgagtttatggcggctacagaggcagcaaaacaagcggtttggcttcaagagttacttgaagagattgttggaaaaacgtgtaagcaagtgttgatattaattgacaacaagtcggctatagcactcacgaaaaacccggttttccatggacgaagcaaacacattcacaagaggtatcactttattcgtgagtgcgttgcgaatgaacaagttgaggtggagcacgtccccggaaccgaacagagagccgacattctaacaaaggcactcgggagaatcaagttcaaagagatgagagatcttgtgggagttcaagatatgacaaagtgtagcttcaagcttaagggggaaaa

>ATCOPIA46_LTR

tgtgaatcagagagaacctcctctacaatcagaaactttgctttctgcgaatggacctgcaacactcaacaatactttgggcctcagtcaaagtgtcaagcccatcaacctcaagtcttctctaccttgcaacggtaacaagattcctcaatttccagcgcatcaacgtacgggattcacagctaatcatttgtcccttcataacaaatttgatcctctttgttcctctgtcgtgatgtgcagctaatgacgtgtgacctagtgtctcaccattttggggttcatatataaactcttctctgtcattgtaaaccttaagctgaaatattcaataaagagatttctcttcatcatcgttttatcaatactttca

>ATCOPIA46_IN

tggtatcagagctcagtcgaaaatggctgacaactctgattcatcttccgctctctgtttttctcattatgttactctgaaactctcaactgcaaactacctcttgtggaagatccagttcgagacttggctcaacaatcaacgtttgcttggttttgttactggtgctaatccgtgtcctaatgctacgagatctatcagaaatggagatcaagttacagaagctacaaatcctgactttctcacatgggttcaaaatgaccagaaaatcatggggtggctccttggttcgttgtctgaagatgctctgcgttcagtctacgggttacacacatccagagaggtatggttttctcttgctaagaagtacaaccgtgtctctgcctctaggaaaagtgacttgcaacgtcgtttgaaccctgtctctaagaatgagaaatctatgcttgagtatctaaactgtgttaagcaaatctgtgaccagctagactctattggttgtcctgttccagaaaacgaaaagatttttggggtcttgaatggtttaggtcaggaatatatgcttgtgtctacaatgattaaaggttctatggatacctatcccatgtcctttgaggatgttgttttcaagctgattaattttgatgataagctgcaaaagtatgcagaggttcctaatgtttctcctcacttggcttatgctactgaatcaaactactcaaagtggtcaatctggtggaaatcgaggtcgcaacaactacaccactaaagggagaggtttccctcaacaaatctcaagtggctctccctctgactcaggaacaagacctacctgtcagatctgcaacaagtatggtcattctgcttataagtgttggaagcgttttgatcatgcttttcagagtgaagacttcagtaaagcctttgctgcaatgcgtgtctctgatcagaaatccaatccttgggtgactgactcaggcgctacttcacacatcacgaactctacttctcagcttcagtctgctcaaccttacagtggagaagattcagttattgttggcaacagtgactttctccccataactcacattggttcagcagttctgacaagcaaccaaggtaatctgccactaagagatgttcttgtttgtcctaatatcactaagtctcttttatctgtgtctaagcttacctcagattatccttgtgtgattgagtttgactctgatggtgtgattgttaaggacaagctgacaaaacagctcctcacgaaaggaacccgccataatgatttgtacctgttggagaatccgaagttcatggcttgctactcttccagacaacaagctacaagtgatgaggtgtggcatatgaggctaggtcaccctaatcaagatgttctccaacaacttttaagaaataaagccatagttataagtaaaacaagtcacagtttgtgtgatgcttgccaaatgggaaagatttgcaagcttccttttgcttcttcagattttgtttcaagcagactcctagaacgtgtccactgtgacttatggggtcctgcaccagttgtttcttctcaaggttttcgatattatgtaatctttattgacaattactcaagattcacttggttttatccattgcgcctcaaatcagatttcttctctgtctttctcacgtttcaaaagatggttgagaatcagtgtcaacaaaagattgctagttttcagtgtgatggagggggtgagtttatcagcaatcagtttgtttctcatcttgcagagtgtggaatcagacagttgatctcatgtccctacactcctcaacaaaatggtattgctgaaagaaaacatcgtcacattacagaacttggttcgtcaatgatgtttcaaggcaaagttcctcagtttttgtgggtagaagctttttacacttcaaatttcctgtgcaaccttcttccatcctcagttctcaaagatcagaaaagtccatatgaagttcttatgggaaaggctccagtctacacttctcttcgagtttttggatgtgcctgctatcctaatctcagaccttatgcaagtaacaagtttgatcctaaatcgttactatgtgttttcacaggttataatgagaagtacaagggatacaagtgttttcatccgcctacagggaagatatatatcaacagacatgttctatttgatgaatccaaattccttttttcagatatctatagtgacaaagtttcaggtacaaactcaactttagtctcagcttggcaaagcaactttctcccgaagagtattcctgcaactccagaagtcttggatatctctaatactgcagcttctttcagtgatgaacaaggtgaattctctggtgctgttggaggaggaggttgtgggtgtacggcggacttagattctgttcctataggcaacagtctcccttcttctccagttactcagcagaattcaccacaaccagaaactcctatctcttctgcaggttcaggaaatgatgcagaagactcagagttgagtgaaaattcagaaaattcagaaagttcagtgttcagtgaagctacaacagaaacagaagctgctgacaacactaatgatcaaagtcatcctatgataacaaggtctaaatcaggcattttcaagccaaatcccaaatatgctatgtttacagtaaagagtaactaccctgtaccaaagacagtcaaaacagctcttaaagaccctggatggactgatgcaatgggtgaagagtatgactcttttgaagaaacacatacttgggatttggttccacctgactcgttcattactcctttaggctgcaggtgggttttcaaaacgaagctaaaggctgatggaactcttgatagattaaaggctcgtcttgttgctaagggctatgaacaagaagaaggagttgattacatggagacttatagtccagtggttcgcacagccacagtgagaacaatcttacatgttgcaaccattaacaagtgggaaatcaagcaacttgatgttaaaaatgcctttttacatggtgatttgaaagagactgtgtacatgtaccaacctccaggttttgaaaatcaagacagacctgattatgtgtgcaagctcaacaaagccatttatggtcttaaacaagctccgagagcttggtttgacaagttcagtacttttctcttggagtttggtttcatctgtacctattcagatccttctctgtttgtgtttctgaaaggaagagatctcatgttcctcttgctctatatggacgacatgcttctcacaggcaacaatagtgaactgttggacacactgcttgtctctctgaataaagagtccgcatgaaagacatgggaatgctgcattactttctcggaattcaggctcattttcactgagaagggttgttcttgcatcaagagaaatatgctatggatctccttgttgcagcaggaatggcagattgtgccccaatgccaacccctttgcctcttcaattggataaagttcctggtcaacaagaaagctttgctgatcctacttattttcggagcttggctggtaagttacaatatcttactcttactcgtcctgacattcaatttgcagtcaatcttgtgtgtcaaaagatgcattcccccacagttgctgatttcaatctcctcaaaagagtcttacgctatctcaaaggaaaagttcaaatgggtctcaacttacacaacaacacggatatcactttgcgtgcttatagtgatagtgactgggcaaactgcaaagagactagaagatctgttggtggtttctgcacttttcttggcactaacataatcagttggtctgcaaaacgtcatccaacagtctcaaggtcctcaacagaagctgagtataggaccctctctatcgcagctacagaagtgaagtggatctcatctttgcttcgtgaaataggcatctatcaacctgcaccaccggagttgtactgtgacaacctctctgctgtctacctcactgcaaatccagcaatgcacaaccggtctaaggcctttgatgttgattttcactatgttcgagaacgtgttgccttgggagctttggttgtcaaacatgtccctgcttctcatcaacttgcagacatcttcaccaagtctcttccccaacgtccattctttgacttaaggtacaaacttggcgttgttttacctcccacaccaagtttgagggggtg

>ATCOPIA47_LTR

tatcagtgagaacttgacctttgggattcaagaaccttcaaagctgcaaaacccaaaaaagaaagaagttgggccactggaagcaaagcccaagcccaatgcgcagcctttgaaagatcctcagcaacggtcatgtccacctgtcaataaagccaaaactgcagtcatcacacagctaagagaacgtacagattcaacgatacctttgaagaaccaatttgagtgtcttggatccggtgatgattgccaagactgacacctgtcattattagggtttaggctacatcaccttgtattcaatataaaaggactttatgatgtaatgtgaaacctaagtcgaaatataagaaagataagttctctatatttcagattacgttca

>ATCOPIA47_IN

tggtatcagagccatggcaaatcagtccatggagctctattcctatcctgttctaaacatctcaaactgtgttactgttaagcttactgagagaaattacctgctctggaaaactcagtttgagtccttcttgtctggtcagaaccttctcggcttcgtcaatggcgctaccaagagtccagatcctgtctcagctgtcacgaacattgatggtgttgttacagagatcccaaatgctgactacagtgctaggcacagatctgaccaagtggtgaagtcatggatcttaggctctctctctgaagacattctggaagaggtcatcactgaatccactgctcaacaagtctgggaaggtctagccaggtatttcaatcgtgtctctactgctcgcctatttgaactgcaaagaaaacagcagactatgtgtaagcatgatacacctatgatagattacattaagggaataaagaacatttgtgaacaacttgcctctgctggtagtcctgttaaggaacaaatgaaattttttgctgctcttaatggtcttggtcgtgagtatgaacctattaagacatccatagagggtagtatggaatctacacctgctcctactctagatagtatcactcctaggcttacaggctttgctgaccgtctcaagagctatgaagtggtgtctgtcactccccatctagtcttcactgcagatgctcctgatgctggcaactactatcagaccaactacagtggcagagggaatttttctggcaagggaaacagaggcagaggagcatacaacaccaagggacgtggctttcatcagcagaccacaactggctcttccatgtcaggagaaaacagacctgtctgtcagatctgtggaaagctaggccatcctgctctcaaatgttggcacaggttcaacaatagctaccagcatgaagaactgcctagtgccttgacagctatgcacatcacagaagttacagaacacaatggacaagagtggttccctgatacaggagcttcagctcatgtgaccaacagtcatcagcatctgcaacaatcaagaccatacaatggttcagacgctgtgattgtaggaaatggtgaattccttccaatcacccatactggctccacaaacctgtcatcaacctcaggtaaacttccacttaaagatgttttagtctgtcctgacattgcaaaaccattactgtctgtgtccaaacttactagagattacccatgctcctttgaatttgattgtgacggtgtccgtgtacatgataagggaacaaagaggttgctaattctgggaacaagtaaagatggtctttatgtgctgaagaacactcctatccaagccttctattccacaagacaacaagcgacatcagatgaagtatggcatatgaggcttggccatcctaaccctcagattcttcagtacctgtcaaagatcaatgctatcaagatcaataagagctccaagtctatgtgtgaagcgtgtcaacttggaaaaagctcaagattaccattttccctttctacttctgtaaccactaaacctttgcaaaggatccattatgatttgtggggtcctgcacctatagtgtcaggtcagggctttaaatactatgctatcttcattgacaactactcccgtttttgctggttctatccattgaagttaaaatctgatttcttcactgtgttcatcaactttcaagctctagttgagaatcagttttctacaaaaatacaaagctttcagtgtgatggaggaggagagtttaccagtaatcagtttgtcaatcacctacaagcgtcaggaatcaaacaactcatctcttgtccccacacacctcagcaaaacggactagctgaaagaaaacacagacacatcatagagttaggcttatccatgatgtttcaaagcagaatacctcaaaagtattgggttaaagctttcttcacggcaaattttctgagcaatctgcttccctcatctgtcttggatgctcagaaaagtccctatgaagtgttacttggcaatgcacctgattacacttctcttcgcacgtttggttgtgcttgttttccaaccctgcgagactacacacaaaacaagtttgatcctagatctttacagtgtgtgttccttggatacaatgaaaaatataaaggctacagatgcttacttccatcaacaggcagagtctacatcagcagacatgttttgttcgatgaacaagtgtttccctttgcaacactcaacaaagaagtcacttccaacctacaaactccactgatgcaggcttggcagaaaagttttcaagtgttgcctactacctcacctcaaccagcttctccactattttcagaaactgacttcccaccattacctatcagaatacctgaacagtctactgtgaggagtgaagggagatctgggtgtaccacaggcctagatcctgcttctataggcaacagtctctctctcattcctcagaggatggatagttcagaaagtacctcaacatcgtcagaaatcccagaattagcaatcgtcactcagccaactgaagaatcaacagagaatcctgcagcttcaacaacgacttctactcagactgaatcagacactcctgcaacagctcaacatccaatggtaaccaggtcaaagtcaggaataacaaagccaaatccgaggtatgcactactaactcacaaagtgtcttacccggagccaaaaactgttacttctgcacttaaagatcctggctggaatggagcaatgacagaagaaataggcaactgtggtgaagcagaaacctggtcattgactcctagaactccagaaatgcatgttcttggctgcaaatgggtgttcaggactaagttaaacgctgatgtctccttgaataaactgagagccagattggttgctaagggattcaatcaagaagaaggaatcgactatctgaaaacatatagcccagtggttagatctgcaacagtaagaggagtgttacatgtggcaacaataatggaatgggaaataaagcaaatggatgttaaaaatgctttcatgcatggtgatttgacagaaactgtctacatgactcaaccagcaggcttcgtggatccagacaaaccaaatcatgtctgtcatctacacaagtcaatctatggtcttaagcagtctcccagagcctggtttgataagttcagcacattcttactagagttcgggttcacttgcagttatcctgatccatcactattcatctacatcaagaacaaagatgtcattcttctattactctatgtggatgatatggtgataacaggtaacaattcaaaggccttatcgaatctattggcagagttaaataagcagtttcgaatgaaggatttgggtgagctacattacttcttgggaattcaggttcagaatcattcagaagggctattcctatcacagcaaaagtatgcagaagacttacttgttgtggctgccatgtctgactgcagtcctatgccaacacctctccctctgcaaattcataaagagtctgatactgatgacgccttccctgatccttcttacttcagaagtcttgcgggtaaactccaatacttgactctaacaaggccagacatacaatttgcagtgaattttgtttgcaggaagatgcatgctccttctcagttcgatttcagtctgttgagaaggatattgcggtatatcaaaggaacaattacaatgggaataacattcagaaaagacactgattgcacattgagagcttacagtgatagtgatttcggaggctgcaagtcaacagtcagatctacaggtggtttctgtaccttccttggcagtaatttaatctcctggtcatcgcagaagcaggactcagtctctaagagctcaactgaagccgagtacagagcaatgtcagaagcagcttcagaaataacctggttgtgctcattcctgaaggaacttggaattcctcttcatgagaccccaagtctgtactgcgacaacttatcagcagtctatctcacggcaaatccggcgtttcacaatcgaaccaaactcttcctgcgacattatcactatgttcgtgaaagagtagctcttggagcgctgattgtgaagcacatcccgtctcatcatcaaatagctgacatcttcaccaagtctcttcctcacggaccgttcagctcattaaggttcaaactcggtatcgattcaccaccgatcccaagtttgcgggggta

>ATCOPIA48_LTR

tactagaccactgctccaaaacgacatcactcctgcaagtggacctgcaacagaaacaaaagtgttgggccaaaacacacttaagcccaaaccatttaagttcaagacggaaacgacgacgtccagctctcaacgttcgtcttcaatgttcctgcaaaaagacaaaggaaacagagaaacagagatgacgaaagcaaaagcttgtacggagacgaccaagctttcaaaccaattctcagcccttgatgggttagagacaacaggatgaagacacctgtaagggtttagctagggtttcagttctcaagtgttcttgtataaaagctaaagcttgagccgcttgtaaaactcttaagaatgaatgaaatctgaaagttaagttacaaagcttctcatagattcataaatctcttca

>ATCOPIA48_IN

tggtatcagagctcatggaaagaagatccatggagctctatactgttcctcaactcaacatttcaaattgcgttacagtcactcttacgcagcagaactatattctgtggaagagtcagttcgaatctttcctttctggtcaaggcttgcttgggtttgtcactggatctatttctgcaccgtcaccaaccattcctgttccagatatcaatggtgtcaccacagacagaccaaatccagagtttgatgtttggttcaagacagacaaggttgtcaagtcttggcttctagggtcctttgctgaagatatcctgagtgttgttgtgaactacgtcactgctcatgaggtatggtctactcttgcaaatcacttcaatagagctacttcatctaggctatttgagcttcaaaggcgtttacaaactctagaaaagaaagataaacctatgcaagtctatcttaaggagttacaaaccatctatgaacagttagcttctgtagggagtccagttcctgagaagatgaaaatctttgctgctcttaatggtctaggtagggaatatgagcctatcaaaacaagtattgaaggttccattgatattcctcccactcctaagcttgatgaaatcatgcctagactcaacggttatgatgatagacttcaggcttatgcagcaaactctgatgttagtcctcatctagctttcaatacagttcaagctaactctgtcttctacaccaaccgcggcagaggtcaagggaaccgtcggtttggtgggtcacgaggccaaggttccttctccactcggggtcgtggcttccatcagtagctctcatacaatgattcttcttccaacgcctctgcagaacgtccaacatgtcagatttgtgggaaacatggtcatcatgctctcaactgctggcacagatttgataatagttatcagcttgatgtgttaccacaggctctcccagcaacgcagatcacagacattactgatcactctggcagcgaatgggtcacagacagtgctgctactgctcacatcaccaactcgccacgtcatctgcaacagacaaagtcctatgctggttctgattctgtaatggttggcaatgggaattttctacctatcactcataccggttctacaagtattggttctacttcaggtaagcttcatcttaaagatgtattggtttgtcctctaattactaaatctttgttgtctgtgtcaaaagtcacaaaggattatccctgcatttttgagtttgattgtgatgaagttcgtgtgcgtgataaggaaaccaagaagcttcttcttcagggaagtaatcgagatggactctatgtgctggatgaaccaaagcttctggtgttctactcttctcgccaagttgcagcgtctgatgaagtttggcacagacggttaggacacccaaatccccatgttctccagcagctatcctcaacaaagtccattcttattaataaacacagcaaggctatttgtgaagcatgtcagtctggtaaaagctcaagactgtcattctctgcatcatcttttgttgctagtagacccttagagagaattcattgtgatctctggggtccttctcctgttatgtcagttcaaggattcagatactatgttatatttattgacaattactctagatattgctggttctatcctctcaagttaaagtctgatttctacacaatctttgcaaagtttcaagctttggttcagaatcagctacaaagcaaaatctcaatttttcaatgtcatggagggggagaattcaccagtaaggtctttctcaatcatcttcaagaacatgggattcaacaatacatctcctgtccttacactccccaacagaatggtcttgctgaaagaaaacacagacacattactgattttggtttatctatgctttttcaaggcaaagtccctcaaaaacattgggtagaagccttttatactacaaactttctcagtaatctgcttcctcatactgctcttactgatgctaaaagtccttttgagctgttaaacaagaagaaaccggattatcaggccttgagaatctttggatgtgcttgttttccgacactccgagattatgcacaacacaagtttgatcctaagtccttaaaatgtgtgttcttgggctacaatgaaaaatataagggctaccggtgtcttcttcctaccacaggcagagtctacataagtcgtcatgtcatttttgatgaacactccttccctttctcagatacttatatgcatctgcaacccactggtgttacacctttgctctctgcctggcaacaaagttttatgcctcagacaactgcttcttctacagcctctgcaactgcaacctctccattcaatgctgcagagattcaggtttctcctgtgattaccagtaacaacaacacaggcgcttcagtgttagaaaatggttcatctcagctgcctatacagaattcatcagtcttgtctactgtggctagtgaagagagttctgagtgtacggagagcatcaatctcttacctattggcaatagctcttcttcacttgctaacaggacggataatgctgatacttctcctcttcaagaagctgcaacagaaacaaacagctctactgtgcaagaagctgcagaatcaacaactagctctacaatgcaagaacctgcttcaaatcagtctactcatccaatgataacacggtctaagaaaggtattacaaagcctaatccacggtatggacttcttacacacaaagttaaatatgcagaaccaaaaacggttacagaagctcttaaacacccgggatggactgctgcaatgcatgaagagtatgataattgtacagaagcacaaacgtggagtctagttccgtatacttctgatatgaatgtccttggaagtaaatgggtgtttcgaaccaagttaaatgcagatgggtctttggacaagttaaaagctcgcttggttgcaaaaggatttgatcaagaagagggaattgattacttagaaacatacagccatgtggtaaggtctgcaacagtcagaatggtgcttcatgttgcaacagtcatggactgggaagtgaaacaaatggatgtgaagaatgcatttcttcatggggacctcactgaaactgtttacatgcttcaaccagctggctttgtgaataaggaaaaacctactcatgtctgccatctacataaagctctttatggtttaaaacaggctcctcgggcttggtttgacaaatttagcaattacttgcttgaatttggtttcaactgcagtattaaagatccatctctgttcatttatttaaaagggaatgatcttatactcttacttctttatgttgatgacatggttttaacaggtagtaactctgcaactatgatcaagctgcttgaggatttgaatacacaatttcgtatgaaagatcttgggcaaatgcattattttcttgggacacaagcttaatttcacgagaattgatgatgtttatacactctttcaggcctatttctatctcagcagaagtatgctgaagaccttctcaccattgcagcaatggacgaatgctccccaatgccaactccactgccacttcagcttcacaaagttcctcatcaagaagaactctttgctaatccaacttatttccggagtcttgcagggaaacttcagtacttgacattgacaaggccggatcttcagttttctgtaaactttgtgtgccaaaagatgcatcaaccaacagtttcagattacaatcttctcaagcggattcttcggtatgtcaaaggaactctatcaatgggaatccacttctccaagcactctgatttccagctccgggtttacactgaaaaagaccctgcttttagtcttcgtgcttacagtgatagtgactggggtggttgtaaagatactcgtcgttccacaggaggctattgcacatttcttggcactaacctcatctcctggtcgtctaagaagcagccgactgtttctcgaagctcgactgaagcagaatatcgatcactctcagaaacagctcaggaaatgacttggatctgtcatttacttcgagagcttggcatacctcttcctgtcacacccgagctctatggagacaacttatcttctgtgtaccttactgccaatcctgccttccacgctcgtagcaaacactttgaattcgactatcattatgtccgtgaaagagtcgccttgggatctttggtcgtgaaacacattcctgctcatcaacaaattgttgacatcttcacgaagtctttgccttatgaagctttctgtaatctcaggttcaaacttggtgtggatttaccacccacaccgcgtttgagggggag

>ATCOPIA49_LTR

tgtaagaagtgactcagattttagtcttcgggtctaagtgtgggagaaactctcggtgaaagagagggttcgaagtctgttgtgattcaagcgaagcacgtgagagagctgactaaagtcataacacgtgaggcgagctgactaaagtcataacaagcttgagggcgttttggtcttttcgcatgagctctgtaactatatcttcttcttcttctctgtaattctgttagatcgkgataagartttgttgagatcaaagagagagaggagtgtagtgagagaaagcttgaaagcattgtaacagagactaagttyccaagttctagtggatttccggagaggtttctccggcgagacgtagcgttccggtttggaacgtgaactcgttaaattctctgtgttcagcttttcttttactttctttcagttctgrtcaattcgattcttagtgctgaagttgattcgtgtgtttgtgagattgaagtgaagttagatctttaca

>ATCOPIA49_IN

aattggtatcagagctcacggttgttgagcgagtgcgaatctaagatggcaggaactccgatcgcgatctttgatggatcaggagatttttctttgtggaaaacaaggattatggcacatctaagcgtcattggacttaaggatgttgtcatcggaacatcgtctccgccgctcactgcagaagaagaagaagatccggagaaaaagaagaaacgagatgcagatgatgcagcaaggcttgagcgatgtgataaagcgaagaatgtgatcttccttaatgttgcagataaggtcttaagaaagatcgagctatgtcaaactgcagcagaagcttggggaactctagatcgattgttcatgattcgatctctacctcatagagtctttactcagctaagtttctatacttttaagatgcaagaaaacaagaaaatcgatgagaatattgatgatttcttaaagattgtggctgatttgaatcatttgcagattgaggtgactgatgaggttcaagcaattctgttgctaagttcgttgccttcaagatatgatggtttagttgaaaccatgaagtatagtaatagccgagaaaagttgaggttagatgatgtgatggttgcagctcgagacaaagaaagagagttgtcacagagtaatcgatctgtatcagaaggaaattttgctagaggaagacaagaaggatcttccaacaataaccaaagaaataagggtaaagggagatcaagatctaagtctcgagatggtaagcgagtatgctggatatgtggtaaagagggacactttaagaaacagtgtttcaaatggcttgaaagaaacaaagataaaggatcaggatcaagttcagacaaaggagaagcgagtatagctaaagctgaatatgatccagcaatggttcttatggcagaagaagagaatctatttgtttcagggaatactgcagacgaatgggttctagatacaggctgttcttttcacatgacaccaaggagagattggttttcagattttagagaagtgaaatctggttatgttaaaatgggaaatgattctttgtctcaggtaaaaggaattggaaacataagaatcaagaactctgatgggacacagattactttaacagaagtgagatacatgccaacaatgtctaggaacctaatttctttaggaaccttagaagacaaaggatgctggttcaagtctcaagatggtattctcaaggtggttaaaggatgctctactgttcttaaaggacagaaaagagaaacgttgtatatacttcttggagaagctgagattgctgaatcaaatgtctccgagaaatccaaagatgaaactgtgttatggcacagcagacttggtcatatgagtcagaagggaatggagatattagtgaagaagggttgtttaaacaggaaagtgattcatgagttgaagttttgtgaagactgcatttatggaaaaaatcatagagtcagtttcccatctgctcagcatgttacaaaggagaagctagcttatatccattcagatttatggggatctcctcataatccagcatctttgggaaactgtcaatacttcatctccttcattgatgactattccaggaaagtgtggatatattttctgaagaagaaagatgaagcttttgagaaatttgttgaatggaagaaaatggtggaaaatcagtctgataagaaggttaagaaactcagaactgataatggcttagagtactgtaatcattactttgagaagttttgtaaagaggaaggcattgtaaggcacaagactgttgcttacacacctcaacagaacggtgtcgctgagagactcaatcgaactataatggataaagttcgcagcatgttaagtgaaagtggtatggaaaagagattttgggcagaagctgctgctacagcagtgtatttgataaatcgatctccctctactgcaacgaactttgagctgccagaagaaagatggacaggagcgttaccggatatgagttcattgagaaggtttggttgtttggcttatgtacacgcagatcaagggaagttgaatcctagagcaaagaagggaatattcacgagctatcctgagggtgttaaaggatataaagtttggctacttgaagaaaagaagtgtgtcattagtagaaacgtgatattcagagaggaaatgatgtacaaagaccttaaaactgattctcagaacagtttctatgaagaagtcatggagaacataggagaaggttctaatcagctgatatctaatatcacagatcaaagtattacagaacaagaaagcgttgagcaaggtggagttactgaagaacagattgttaatgaacagaatcaagtgcagacagaaactcatgaagaagaaggaagcagtagtgataattctactgaagaagtggatctcagtaactacctactagtaagagacagggagaaaaggactgttaagttgaacagaagatataatgagtctaatatggttgggtttgcctacaatacagaagatgggggtaagtctgagcctaaaacataccaagaggcattaagtgatcaagattgggagttatggaatggagctatgaaagaagagatatcatccatgggaaaaaaccacacgtgggacttagtagataaaccagtaaatgcgaaaatcattggttgcagatgggtcttcacaaggaaagctggtattcccggagtggaagcacctagatttaaagctcggctggtagccaaaggttttacacagaaagaaggtgtagattataatgaaatcttctcaccagttgttaaacatgtgtctatcaggtttatgctctcaatggtggctcagtttgacatggagctacatcagatggacgttaaaacagcgttcctacatggatttcttgatgaagagatattgatggctcaacctgaaggttttgaagataagaaataccctgaaaaggtatgtttactgaaaagatccctatatggtctaaagcaatctcctagacagtggaatctgagatttgatgagtttatgaagagtattgatttcactaggagcgcttatgacagctgtgtatacttgaagcagcaaagtgataagtcatatgtgtatctactcctatacgtagatgacatgttgatagctgctaaagaaaagtcaagtatcatggagttaaaacagttactgggtaaagagtttgagatgaaggatttaggtgaagctcagaaaattttaggcatggagattgctagagatagagctgcaggtgtgttgactctgtcacaggaaggttatgtgaagaaagttttgagatctagtcagatggatcaggctaaacctgtgtcaacaccattaggaatccatttcaagctccgagcagctactgaaaaagagtatcaggaacaatttgacagaatgaagattgtaccttactcaaacactgttggaagcatcatgtattcaatgataggcacgaggccagatcttgcttatccagttggagtgatcagtcgctacatgagtagaccattaaaggatcattggcaagcggctaagtgggtcttgaggtacatgaaggggacagagaaaaagaagctttgcttcagaaagaataaagacttcttattaagaggttactgtgactctgactatggtggtgattatgataaccggaggtcaatcacaggttatgttttcactattggtggaaacaccattagctggaaatcacggcaacagaaggtggtagctatatctactacagaggcagaatatatggcattaactgatgctgttaaagaggctttgtggctgagaggtttttctgaagaacttggttttgcacaggaaagtgtagaggtgaattgtgattcagagagtgttattgctttggcaaagaactctgtacatcacgaaagaacaaagcatattgatatcaggttacatttcattcgggatatcataaatgcaggtctggttaaagtggtgaagattgcaagtgaatgcaatcctgcagatatttttacaaaggtattgcccgtggagaagtttgaaggagcattgcatatgctccgagttactgagaactgagaggtgaatctcaggtaccggaggggagatccaagaactagaacaagttgagacaaggaagacaatcagagtcaaggtggagaat

>ATCOPIA4_IN

tggtatcagagcaaacgataccctaattttttttttcaaaacacaaacctagccgcctaacatgggctcctccgcaaacggtctcccagccaccactgatgaagcaattgtcttcactccgcaaacaatcttcaacattaacacgtctaatgtcacgaaactcacctccaacaattacctcatgtggagccttcagatccacgccttgcttgatggatatgaactcgcaggacatcttgatggttctatcgagactcctgctccaacactcactacaaacaatgttgtctccgctaatccacaatacacgttgtggaagagacaagacaggctcatcttcagtgccttgattggcgccatctctccaccggtgcaaccattagtgtctcgtgcaaccaaagcctctcaaatctggaaaaccttaaccaacacgtatgctaagtctagctacgaccacatcaaacagctccggactcaaattaagcaactcaagaagggaaccaaaaccattgacgaatacgttctgagtcacacaactctccttgatcaattggctattctcggcaaaccaatggaacacgaagaacaggtggaacgtatccttgaaggtcttcctgaagactacaaaactgttgttgatcagatcgaaggcaaagacaacactccctctattacggagattcatgaacgactcattaatcatgaggccaagcttttgtccactgctgctctgtcatcctcgtcgcttcccatgtcagctaacgttgctcaacaacgccatcacaacaacaatcgtaacaataaccaaaacaagaatcggactcaaggcaacacctacaccaacaattggcagccctctgcaaataacaagtcaggtcagcgccctttcaaaccttacttggggaaatgccagatttgcaatgttcaaggacacagtgcgcgtcgatgcccacagctgcaggcaatgcaaccgtcttcgagctcctcggcctccacgttcacaccatggcagccacgagctaacttagcgatgggagcgccatacacagcaaataactggcttctcgatagtggagctacccatcatatcacgtccgatctgaacgctcttgcccttcaccagccctacaatggtgatgatgtcatgatcgctgatggcacaagtcttaagattacaaaaactggttccactttcttaccttctaatgcccgtgaccttactttgaataaagtgttatatgtacccgatatacagaagaatttggtctcagtgtaccgcctatgcaatactaatcaagtgtccgttgaatttttccctgcctcttttcaggtgaaggacctcaacacggggaccctgttgctccaagggagaactaaagacgagctctatgaatggccagtgactaatcctaaagctacagctctgttcacaacaccaagtccaaagaccactctttcttcctggcattctcgcctaggccatccttcttcttctattctaaacactttaatttcaaagttttcacttcccgtttcagtttctgcttcaaataaacttgcttgttcggattgtttcattaataagagccataaactcccattttctatctcatccattaaatccacctcaccgcttgaatatatattttctgatgtctggatgtctcccatattgtcaccagataactacaaatattaccttgttcttgttgatcatcacacacgatatacatggctttaccctttgcagcaaaagtctcaagtaaaatccacttttattgcgtttaaagcgttggtcgagaacaggtttcaagcaaaaatccgaacactttactcggacaatggcggagaatttatcgcactacgagagtttctcgtttccaatggtatctctcatctcacctctccaccacacactcccgagcacaatggcctatccgaacgcaagcacaggcacatcgttgaaacaggactcaccttactcactcaagcttcggttccacgagaatactggccatacgcattcgccgcagctgtttatctcattaaccgaatgccgactccggtgctatccatggagtcaccgtttcagaagctgttcggatccaagccgaattatgagcgtctacgagtattcggttgtctgtgctttccatggctcagaccttacactcacaacaaattagaagaacgatcgagacggtgtgtgttcctcggttactctttaactcaaacagcctacctctgtttcgatgttgaacataagcgactttacacatctcgccatgtcgtgtttgatgaagcctcctttcccttctccaacctcacatcccaaaattctctccccaccgtaacctttgaacagagctcctcgccgttagttacgcccatactctcatcatcgtcggttctcccatcttgtttgtcttccccgtgtacggtccttcaccaacaacaaccgccggtgactacgccgaactcaccacattcatcacagccgacaacctcaccggctcctctgtctcctcaccggtcaaccacaatggactttcaagtcccacaggtacgctcttcgtcacccttattatcttcttcttcatctttaaattctgagcccactgctccaaatgaaaatgggcctgaacctgaggcccagtcaccacctataggcccactgtcgaatccaacccatgaagcctttattggtccactcccaaacccaaaccgaaacccaaccaatgaaattgaaccaacacctgcgcctcaccctaaaccggtcaaacccacaaccaccactaccactccaaatcgaaccaccgtctccgacgcctctcaccaaccaactgcaccacaacaaaatcaacacaacatgaaaacccgagctaaaaacaatatcaaaaagccaaacacaaaatttagcctcactgctactctcccaaatcgttctccatccgagccgaccaatgtcactcaagcccttaaagacaaaaagtggcgttttgccatgtccgatgagtttgacgcccaacaacgaaatcatacatgggatctcgttccccatgaatctcagcttcttgtcggttgcaagtgggtcttcaaactcaagtatctcccaaatggtgccattgacaaatacaaagcacgcttagtggccaaggggttcaatcaacaatatggtgtcgactatgcggaaacgtttagtccagtcattaaatctacaacaattcggcttgttcttgatgtcgcagttaagaaagattgggagattaaacaactagatgtcaacaatgctttcttacaaggaactctcaccgaagaagtatatatggctcagcccccgggtttcatcgacaaagatcgtcccactcatgtttgtcgccttcgcaaagctatatatggactgaaacaggccccccgagcgtggtatatggagctgaagcaacacctattcaacatcggcttcgtcaactcactctccgatgcgtctttatttatctactgtcatggcaccactttcgtctatgtacttgtctatgttgatgatattattgtcacagggagcgacaagtcatccatcgatgcggtgctgacttcccttgcggaacgtttctccatcaaagatcccacagatcttcactacttccttggtatagaagcaacccgaacaaaacaaggtttgcaccttatgcaaaggaagtatatcaaggatcttctcgcaaagcacaacatggctgacgcaaaaccggtgttaacacctttacccacctcaccaaagctcactctccatggtggtacaaaactcaacgatgcatctgaatatcgatcggtggtgggtagcttgcaatacttagcgtttacacgtcctgacattgcgtatgccgtcaaccgattatctcagctcatgcctcaacccacagaagatcattggcaagcagctaaaagagttcttcgatatcttgccggcacatcaacgcatggtattttcctagacactacctcaccattgaatctccatgccttttcggatgcagattgggccggggattccgatgattatgtttctaccaatgcatatgtcatctatctgggcaagaatccgatctcttggtcctctaagaagcagcgtggtgttgcccgctcctccacagaatccgaatatcgagctgttgcaaacgctgcatctgaagttaagtggctttgctcacttctctctaagttacacatccggttaccaattcgcccttctatattctgtgacaacattggagctacctacttgtgtgctaatccggttttccactctcgtatgaagcacatagccatcgactaccatttcgttcgcaacatgattcagtccggtgctcttcgagtctcacatgtatcaacacgagatcaactagcggatgccctcaccaaacctctctctcgagctcactttcagtccgcacgtttcaagattggagttcgtcaactccctccatcttgagggagcg

>ATCOPIA4_LTR

tatagagaatatatgtcataatgtcttaaaggacatgtttgtaattaatcaatccctaatcatatgtctgtatcttagtataaatatgtacattacattcttaataaagtcaatacattctccttccata

>ATCOPIA50_LTR

tgttaagatttagcaacaaagaacacaacatgaagaagcttgaggaagaagaaatgccaactcagcaaaggggacacgtcaacagcaggaccacaagacaaagaaggagccgttagcgccaaccagggttatgagtcgcacgactaaagcattcttgtcttggtttggtaaagttgcacgcgggttaagcccaatccaaattggtggaaggtgacaaatttggaggcagcttggagacagctgtagaggagcttgtagaggcaaaccaagaaggatccagaagggaacctaggttaaaacctctaagtccatttaagctgaaggtggtgcttagagacagagttagaaacgtagagacattcattagagagtaaacttgtagtgagtgttcttagggaaacaatgagtgagagagagtgaatcatattggttctgtaaggggctgagagggtgagagatctctttgtaaaactcaaaggttatagtggattccgggaaagatttcccggcccagacgtagcaccgttggtgtgaactgggttaacaatctgtttgtgtgctctgtgttgttatgtttcactttcagtcacaagtcttcttatctcaaagttctgttcttgttcgatttcaagtttcttagacatcaagtcttgaaacccttagtctgtatttggtctaaacactaca

>ATCOPIA50_IN

aagtggtatcagaacgaaggttctttgtattggcggttcagtaaggttcttacaaacaaagaagccatgtcgtcagcaagggtagagatggagaagtttgatgggcatggagactacacattgtggaaggagaagctgatggctcacatggatctgttgggtttaacggtggctctcagggaaacacagtcggtctcagatcctctagaatctgaggaagaaggaaaagaatctgaaaaaggagataaggaagcactcatggaagagaagagacagaaagcgagaagcaccattgttttaagtgtctcagatcaagttttgcggaagagtaagaaagagaagactgcaccttctatgttggaagccctagacaaactgtacatgtctaaggcacttccaaaccgcatttatctgaaacagaagctttatagctacaagatgcaggagaacttgtctgtagaaggaaacatagacgagtttctacgtctcatagctgatctggaaaacacaaatgtgttggtttcagatgaagaccaagctattctgctactgatgtctttaccaaagcagtttgatcaactgaaggatactctaaagtatggctcagggagaacaacactatccgtcgacgaagttgttgcagccatttactcaaaggagctagagttaggctcaaataagaagagtataagaggacaagcagagggcctgtatgtcaaagacaaaccagagacaagaggcatgtcagagcagaaagagaaagggaacaaaggacggtcaagatctagatctaaaggctggaaaggatgttggatctgcggtgaagaaggtcacttcaagacctcatgtcccaacaaagggaagcagcagaacaagggaaaagaccaagcaagcggtagtaaaggcgaagctgcaacgatcaaaggaaacacatccgaaggttcaggctattatgtgtcagaagcattacattcaactgatgtaaatctggggaatgaatgggtcatggacacgggttgcaactatcatatgactcataagaaggaatggttcgaggagctgagtgaagacgctggagggacagtgaggatgggaaacaaatcaacctcaaaggtcagaggcattggaagtgtcaaaatcctgaacaaggatggaacaacagtttcgtgtaaagtacattcctgacatggatcgtaatttactttctatgggaactctagaagaacatggttatagttttgagtcaaagaatggagtcttagtggtgaaagagggaactaggactttattgataggcagcagacatgaaaagctttacttactccaaggaaagcctgaggttagtcattctatgactgttgaaaggagaaatgatgatacagttctgtggcacagaagattaggccatataagtcagaaaaatatggatattctggtcaagaaaggatacttagatggaaagaaagtttctaagttagagttgtgcgaagactgtatttatggtaaagctaggaggctgagtttcgttgtggctacacataacactgaagacaagttgaattatgttcattctgatttgtggggagcaccatcggtccctctatccttaggaaaatgtcagtatttcatctcgttcatagatgtttactcaagaaaaacgtgggtttattttctgaaacacaaggatgaagcttttgggacctttgctgaatggagtgttatggtagagaaccaaacaggaagaaagatcaaaattctgaggatagacaacggtctggagttttgtaatcagcagtttaatgatttctgcaaggagaagggtattgtgaggcatcagacatgtgcttacacacctcaacaaaacggcgttgcagagaggatgaatcacacaatcatggaaaaagtcagaagaatgttaagttattctggcttacctaagacgttttgggcagaagcaactaacactgtagtgactctaatcaacaaaactccctcatctgcagtcaactttgagatttctgacaagagatggtcaggtaagtctcctgtttataactacttaaaaaggtttggttgtgtggcttttacttatgcagatgaaggaaagctagtacctagagccaagaaaggagtgttcttaggctatctcagtggagagaaagggtacaaggtctggttgttagaagaaagaaagtgcagtgttagtagaaatgtgacatttcaagagaatgcagtctaccgagatgtgatgcagaagaacaaagactctgaggaagtggacacatcaagcaggtctcttgatattgatcttgaagatgttggtgatttgagcttaggtggagatctcttggaagaaacatgatccctgggagatcactcacctgctcaggaccatcaccacactgaagacgcggagatcaatgaagaaacggtacctgaaacaccaaacagctaccatttggttagagacagagttagaagagaaatcagagctccaaaacgttttgatgtggagggttattacagtgagtttactgatgatgaagaagaatcatttaatgttgaagccttggtcaccacagtagatggagacaccagagaacctggaattatcaagaagctctaagagatgatgattgagaactatggaaagtcggcatggatgaagagatggactctctgttgaagaatcacacctggactgtagtaaagaagcctgttggagaaagagtgataggctgcaaatggattttcaagcgtaagcctggaactcctggaattgaacaacctagattcaaggctaggttagtttcaaaagggtatgcacaaagagaaggaatagactacacagatatcttctctccagtggtaaagcacgtttcaatacggatattgctggctattgttgcagaggagaattatgagcttgaacagctagacgtcaagactgcttttcttcatggagatctagaagaaaagattttcatggaagcaccagaaggttatgaaagtcagttcaagcaaggagaagtgtgtctgttaaacaagaccatgtatggtctaaagcaatcaccaagaagatggaatcaaaagtttgatagttacatgctggaaatcggctttgagagaagccctcgtaataagtgtgcatacattaaatcacttgaggatggctcaaaggtctatctacttatctatgtcgatgacatgttggtggctgctagagatatgcaggtcatttctgagttaaagcagaaactcagtgagaagtttgaaatgaaggatctcggagctgctaagaggatacttggcatggagatctcaagagacagagttaagggaactctcacactgtcacaagaagattacttgagcaaggttctggaaacatacaacgtggatcaatgcaagtttgtggtaacacctctcggtgctcacctaaagatgcatgcggcaacagaacaacaactgctcagtgatgaagagtacatgaagtcagtgccttattcaaacgcagtaggaagcattatgtactctatgatcgatacaagaccagatttggcttattgtgttggcatcatcagtcggtttatgagcaaaccagttaaagagcactagttaggggtcaagtgagtacttaggtacattaaaggaaccctgaacatgaagttgtgctagaagaagggagcagacttaaccctcagagggtattgtgactccgactatgctgcaaacctggagaacaggcgatcaataagcggaatggtattcacacttggtgggagcaccataaacttgagatcatgtctgcaaaaagtagtggtaatgtcatccactaaagccgggtatatgtctctcactgaagcagtaaaggaggctatatggcttaaaggtttgttacaggactttggctatgaacagaagacggtggagattttctgtgattctcaaagcgccatagccctgtcaaagaacaatgtgcaccatgacaggacaaagcacattgacatcaaataccacaagatccgcgaggtcattgcagatggagtggtagaagtgaagaagatatgcacactggtgaattcagctgatatcttcacaaaggttgtacccgttagcaagttcaagacagctttgcactcgctacgagtaaagactgagtaatctactcaggaccgggaaaggaatcctaaactataacaagttattctctctcaaaaaggtattctcgcttctgtcaattcgtgtttcatgttcttctattgctaggtggagatc

>ATCOPIA51_LTR

tgtagaagacaacccaataaatagaacgggctttgacaaaaacaaagacatggtagcccaaactcaacaaagcccactacagaagcccagtcaacatatgcaaaagaagataaagaccaaaagctctttacctgcaaaacagaagactgtcactaatacggagaagaagacaataacacttgaaaacaaattcaagccgttgcagtcgctgccgttgcagtaattcacaataaatagctattgattaaagatagcattgtcaaggattctaaattgttctaaagaagtcataaaagaaagatgtaaaactccattgaaagataagcaaaataaaaacattttttgtcaagaaataatcacagttttatttttctcttca

>ATCOPIA51_IN

tggtatcagagctattataccttaacaggtgattcaatggcccctgcttacccttttccagacaatgtccatgtctctagttccgttaccttaaagctcaacgatagtaactacttgttgtggaagacacagtttgagtcccttctatcgagccaaaagctcataggttttgtcaatggagtcgtcactcctccagctcagactcgtcttgttgttaatgatgatgtcaccagcgaagttccgaatcctcaatatgaagactggttttgcacagaccagctcgtccggtcgtggttgtttggtacgctttcagaggaagtgcttggtcatgtccacaacctcactacatctcgtcagatttggatctctctagctgaaaatttcaacaaaagtagcatcgccagagagttttctcttcgtcgtaatcttcaacttctgacaaaaaaagataagtctctatctgtttactgtcgtgattttaaaataatatgcgactctctaagctccattggcaaaccagtagaggaatccatgaaaatctttggctttctcaatggactcggcagagagtacgatcctatcaccacagttatccaaagctccctaagcaagctccctgctccgacgtttaacgacgtcatctccgaagttcaagggtttgacagtaagctgcaatcttatgacgacactgtctctgttaatcctcatcttgcgttcaatactgaaagatctaactctggcgctcctcaatacaattccaattcccgtggtcgtggtcgttctgggcaaaacagaggacgcggtggctactctacacgcggcagaggattttctcaacatcaatccgcttcaccatcatcaggacaaagaccagtttgtcaaatttgtggtcgcataggacacactgctatcaaatgctacaaccgatttgacaacaactaccaaagtgaagtccctactcaagcattttctgctctccgtgtctctgatgaaaccggcaaggaatggtaccccgattctgcagccacagcccacataacagcctcaacatctggtctgcaaaacgcaacaacatatgagggaaacgatgcagtcttggttggagatggaacatacctccctattacacatgttggatccaccacaatttcctcatccaaaggtactattccgttgaatgaagtcttagtgtgccctgctatacaaaaatctcttctatctgtgtccaaactttgcgatgattatccatgcggtgtttattttgatgctaataaggtttgcataattgatttaaccactcagaaagtggtgtccaagggtccacgaaataatgggctctacatgctggagaattcagagtttgtagcactctattcaaatcgtcaatgtgcagctagcatggaaacatggcatcatcgacttggccactcaaactcaaagattcttcagcaacttttaacccgcaaggaaatccaagtgaataaaagcagaacttctcccgtttgtgagccttgccaaatgggaaagagcactagattacagtttttctcttctgattttcgagctttaaaacctttagatcgagttcattgtgatctttggggaccatcaccggttgtatcaaaccaaggattcaaatactatgcagtttttgttgatgatttctcaagattctcttggttttttcctttgcgcatgaagtcaaagtttatttcagtgtttattgcatatcagaaattggttgagaatcaacttggtacaaaaatcaaagagtttcaaagcgatggagggggagaatttacaagcaacaaattaaaagaacactttagagagcatggcattcatcatcgtatatcttgtccatatacaccgcaacaaaacggtgttgccgaaaggaagcacagacatttggtagagcttgggctttcaatgttatatcacagtcatacacctctcaagttctgggtagaagctttcttcactgccaactatctcagtaatctcttgccttcttctgtcctcaaggaaataagtccctatgaaactttgtttcaacaaaaagttgattatacacctctccgagtgtttggtacagcctgctacccctgcttgagaccgttagcaaagaacaagtttgatccacgctcgttgcaatgcgtgtttcttggctatcacaaccaatacaagggataccgctgtttgtatcctcctaccggtaaagtctacatctctagacatgtcatttttgatgaagctcaattcccatttaaagaaaagtaccacagtctggttccaaaataccagacgaccttactacaggcttggcaacatactgatctcacaccaccttcagtgccttcttctcaattacaacctcttgcaagacaaatgactcctatggcaacaagtgagaatcagccaatgatgaattatgagacagaggaagccgtcaatgttaatatggaaactagctctgatgaggaaactgaatcaaatgatgaatttgaccacgaagtagctcccgtactaaatgatcaaaatgaagacaatgcactaggacaaggctcattagaaaatctccatcccatgattacaagatcaaaagatggaattcagaagccaaacccccggtatgctctcattgtctctaaatcctcttttgatgaaccaaaaactattactactgctatgaagcatcctagctggaacgctgcagttatggatgagatagatcgcattcacatgctaaacacttggtctctagttcctgcaacagaggacatgaatattctgacatccaaatgggttttcaagactaaactcaaacctgatggcaccatagataagttgaaagctcgtctagttgccaaagggtttgatcaagaagaaggagtcgactatcttgagacattcagtccggttgttcgaactgcaactatacgtcttgttctcgataccgctactgcaaatgagtggcctctcaaacagcttgatgtgtccaacgcgtttctccatggagaattacaagaaccggtgtttatgttccaaccctctggttttgttgatcctaacaagcctaatcacgtttgtcggctcaccaaagctctttatggtctaaaacaagcgcctagagcctggtttgacacctttagcaactttcttcttgactttggctttgagtgcagcacatctgatccttccctcttcgtttgtcatcaaaatgggcaaagtctcatactcctcttatatgtcgacgatatactcctcacaggaagtgatcaactgctcatggataaacttcttcaagctctcaacaaccgcttttcgatgaaagatcttgggcctcctcgctattttttgggtatagaaattgaatcttacaacaatggtctatttttacatcaacacgcatacgcttccgacattcttcatcaagcaggcatgacagaatgcaaccctatgcctacccctctgccacaacacttggaagacctcaattcagaaccctttgaagagccaacatactttcggagtttagctggcaagttacaatacttaacaatcacaagaccggatattcaatatgccgtgaacttcatctgccaaagaatgcacgctccgaccaactctgattttggccttctcaaacgcatactcaggtatgtgaaaggaactatcaacatggggcttccaatcagaaaacaccacaaccctgttctttcgggattttgcgatagtgattacgctggctgcaaggacactagacgctccactactggtttctgcatcctcttgggatctactctgatatcttggtctgcaaagagacaacccactatctctcactcctcaacagaagccgaatatagagctctttccgatacagctcgagaaatcacttggatttcctctcttctccgagatcttggaatctctcaacatcaacctacacgagtgttctgtgataacctatctgctgtctacctctctgcaaatcctgctcttcataaacgatctaaacacttcgataaagactttcactacatcagggaacgtgtggctctcggtctcatagaaacgcaacacatcccagcaactattcaacttgctgatgtcttcaccaagtcactaccgcgacggccctttatcacgcttagagccaaactcggcgtgtctgcgtcaccggtctcacccacgccaagtttgaaggaggg

>ATCOPIA52_LTR

tgtgagtgagatgtctgaagctaaggagatgggccaaacccataacaaatcaagtccaactataaagcctggtcaacaaatacagaagatgacaaagatcaaaagctgtctctcatccgtacatgtacctgcaggaaacattgaaagagaaaggaagacgctcctctaaaactaattcaatcctctttcatccttatctaataaccaaaaattacatacatctgtatagtgtcgagagtgttctagatccttctccctcaatctctataaaaagaagaatgtaatctcattgtaattattattgaaaaacagagtaatattattaaaaggttcaaattttctcttctttctca

>ATCOPIA52_IN

tggtatcagagccattctaaactctcacaggtccaaaaatggcagatccatacccttttccggacaatgtccatgtctctagttctgtaactctcaagctcaatgactccaactatctcctttggaagacacaattcgagtctttgctatcttgtcacaaactcatcggctttgtcaatggtggaatcacaccaccaccacgtactctcaacgtcgtcacaggagacacctccgtcgatgtcgcaaaccctcagtatgaaagttggttctgcactgatcagctcatccgttcatggctttttggcacgctatcagaagaagttctcgggtatgtccacaatctccaaacctctagagatatttggatctccttagcagaaaacttcaataagagcagtgttgctcgtgagttcacgctccgccgtactctgcaactcttgtcaaaaaaagacaaaactttatcagcgtattgtcgtgagtttattgctgtgtgtgatgctttaagttctataggcaagcctgtggatgaatcaatgaagatctttggttttcttaatggtctgggaagagagtatgatcctatcactactgttatacaaagctctctgagtaaaatttctccaccaaccttcagagatgtgatttccgaggttaaagggtttgatgtgaagctccagtcctatgaagaatcagttactgccaatcctcacatggctttcaacactcaacgtagtgaatacacagacaactacacttccggcaaccgtggtaaaggtagaggaggctatggtcaaaatcgcggcagaagtggctactctacgcgtggaaggggtttctctcagcatcagacaaactccaataacacaggagagcgtccagtgtgtcagatctgtgggcggactggacacacagctctaaaatgttacaacagatttgatcacaactatcaaagtgttgatactgcccaggccttctcttctctgcgggtttcagacagttctggtaaagagtgggtacctgattctgcagctacagctcatgtgacttcttccacaaataatctacaagctgcatcaccctacaacggcagtgacacagttcttgttggcgatggagcatacttacccatcacacatgttggatccaccaccatttcttctgattcaggtactcttccactaaatgaggtcttagtatgtcctgatatacaaaagtcccttctatcagtatccaaactatgtgatgactatccttgcggtgtgtattttgatgctaataaagtatgcattattgatataaatactcagaaagtggtgtcaaagggtcctcgaagtaacggtctatatgtgttggagaaccaagaatttgtagccttctattctaatcgacagtgtgcagcatccgaagaaatatggcaccatcgcttaggacattcaaattctcggattcttcaacaactcaagtcaagcaaggaaattagtttcaataagagcagaatgtcccctgtttgtgagccttgccagatggggaaaagttctaagttacagtttttttcttcgaattctcgtgagttagatcttttaggtcgaattcattgtgacctttggggcccctcaccagttgtatctaaacaaggtttcaagtattatgtggtgtttgttgacgattactctcgctactcatggttttatccattaaaagcaaagtcagatttttttgcggtatttgttgctttccaaaacctggttgaaaaccaatttaatacaaagatcaaggtgtttcagagtgatggaggtggtgagtttacaagtaacttaatgaagaagcacctaacagactgtggaattcaacatagaatctcttgcccctatactcctcaacaaaacggtatagcagaacggaagcatcgtcactttgtcgagctcggtttgtcgatgatgtttcacagtcacacaccgctacagttctgggtagaagcgttcttcactgcaagtttcctaagcaacatgcttccctctccgtcattaggcaatgtaagtccccttgaagctttactaaaacagaaaccaaattatgcaatgcttagagtgtttggaacagcatgttatccctgcttaagacccttaggagagcataagtttgagcctagatcactacaatgtgtatttcttggctacaattctcagtataaaggatatagatgtctatacccacctaccggaagagtgtatatctcaaggcatgttatcttcgatgaagaaacatttcctttcaaacaaaaatatcagttcttggttccacaatacgagtcctctcttctcagtgcttggcagtcatctataccacaagctgatcagtcactcataccgcaagctgaagaaggaaaaattgaaagcttagcaaaacctccatcgatccagaagaatacaattcaggatactacaactcagcctgcaattttaactgagggagtattgaatgaagaagaggaagaagactcctttgaagaaacagaaacagaatctctgaatgaagaaacacacactcaaaatgatgaagcagaggttacagtagaagaagaagtacaacaagaaccagaaaacactcacccaatgacaacaaggtctaaagccgggattcataaatcaaacacacgatatgcacttcttacctcaaaattttcagttgaggaaccaaaatcgattgatgaagccttaaatcaccctggttggaacaatgcggtgaatgatgaaatgagaacaattcacatgttgcatacatggtcattggttcagcccacagaagatatgaatattttgggatgcaggtgggtgttcaaaactaaactcaaaccagatgggtctgtggataagctaaaagctaggcttgttgccaaaggatttcaccaagaggaaggtctagactatcttgaaaccttcagtccggtggtcagaacagccactatccgtcttgttctcgatgttgctactgctaaaggatggaacataaagcaacttgatgtgtctaatgcgtttcttcacggtgaattaaaggaacctgtctacatgcttcagcctcctggttttgtggatcaagaaaaaccttcatatgtgtgccgtctcaccaaagctttgtatggcttaaaacaggctcctagagcttggtttgacacgattagtaactatcttcttgactttggtttttcttgcagcaaatcagatccttctctattcacatatcacaagaatgggaagactttggtgttacttctatatgtagatgacattcttctcaccgggagtgatcacaatctacttcaagagcttctcatgtctctcaacaaacgtttttcaatgaaggatctgggcgctccaagttatttccttggtgtggaaattgagtcatcaccagaaggtctcttcctccatcaaactgcctacgctaaagacattcttcaccaagccgcaatgtcaaactgcaactctatgcctactccactacctcaacacattgagaacctgaattcagacctcttccctgaacctacttacttcagaagtttagctggaaagcttcaatatttaaccatcacccgacccgacatacagtttgctgttaacttcatttgccaaaggatgcattctcctactacagcagattttggtttgctcaaacggattctgagatatgtgaaaggaactattcacttgggcttacacatcaagaaaaaccagaacttgtccctcgtagcttacagtgatagcgactgggctgggtgtaaggaaacaagacgctcgacaaccgggttctgtacactacttggatgcaacctcatttcgtggtcagccaagagacaagaaacagtgtcaaaatctagcacagaagcagagtatcgagctcttacggcagtagctcaagagcttacttggctgtcttttctgcttagggatattggagttacacaaacccatccaaccttggtgaaatgtgacaatctatcagcagtttatctaagcgccaatcctgctcttcataacaggtctaagcactttgacacagattatcattacatcagagaacaagttgctttgggtcttgtggagacaaaacacatatctgcaacgctgcaacttgcagacattttcacaaaaccgctaccaagacgagccttcattgatctcagaatcaaacttggtgtagctgaaccacccaccacaagtttgagggggaa

>ATCOPIA53_LTR

tatcaaatatgtattggtacaatgacccggtttaccggtttatctatttttgtcttggttaagtagattgtatttgaggatcaaaccttgtttcctttattgtcaatgaagcatatttctaatctaaggtaaagtttgttagatctaaaa

>ATCOPIA53_IN

tggtattagagcttttcccagcttcattcatcttcttctgcaaattttcttctattctctgataatttctcttgattctcttttgtttctcaatctttgtttcattctctatcgtttgatcggagaatttctttgatcgcgtttcattccgatcattttttcattttctggttttgttactcttcgcgatggctaatccagctccgacgagctccaatccttactcaaatccattgtgtcttcatgctgctgacaattcgggtgttaaatctcgttctcgacaagctcatcggtgaatcttactatcttacaaggcgtcgttctatcatcaaagctctcaacgctaaaagcaagctcggattcatctatggaagtgtcgttaaacctccggaagatcacaatgactatggatcttagactcgctgtaacgatatggtatgtacctggattacgaactttgtttcaaaggatttaggatctggaatggtctatttttatgatgctcatctcttatggttgaaactagaaggtagatctaggcagagtaatttgtctaagatctatagtgttcaaaaccaactagatcgtctacatcaaggatcattagatctcagtgcttattacactcgtcttacggttaccttcattagatctcagtgcttatcatgggaagaattgaagaattttgaagagttaccttcctgtacttgtggaaaatgcacttgcggttcaaatgatcgttggattcagttgtatgagaagcacaacattgtcagattcttaatgcgtctcaatgaatcgtttattcaagcgcgtcgtcagattcttatgatggatccacttccagaattcacaaatctctataacttcatctctcaagatgatcaacaaagaagttttaattcaatgcctaccacagagaaacctgtttttcaagcttctataactcaacagaaacctaagtttttcaatcaacaaggaaaatctcgtcctctttgcacttactgtggtttattgggtcatactaatgctcgatgttataaacttcatggatatcctccaggctacaaggttcctgttggtacatgctacaataatgacaaatcaagaggtcagccttatccacataatggcattcacatggtttattctcaacctcccgaacagatgatgtattctctagaaaagctccacagattcaatctatgattccatagagtcatcttatcacttacaatgggaactcatatgctccaattgttcaagctaataacaatgctccatatgctctttataatcaagcttataatgggaattcgtatgctcctatggctcagaactttgctggaaatcatattatctctgatggatcttcaatgtctgctggtaatgtgactagtgaatcgccaactgtaaatcattctgtcaatatgatgaattctggaagaggttttcttggtagtagttcacatggtagggaacaggttaatcagatggttactcaattgaacacacaacttcaaggttctccgtatcaagttattcgaccaccaactgtctcgcaaaatcatggttctatctcagcacaaggtatgtcacctataccttctcactatatttctgctttcgaaccatgtcttataattccacaaaatacctggagtcttgatactagagctagttgtcatatctgttgtgacttgagtttgttctgtaatgtctaccatattgatcacacgaatatcactctaccaaataatatcaaaatttccattaacatagctgaaacagttaaactcaatgataggctcattcttcatcttgttttctatgttccttcttttcatttcaatcttattagtgttagttctttaacaaaaacaaataaaaaaaattcagtgcatttttatcataagaattgtttcattcaggatcatactcacgcatggacgattggtagatgagattagcccaatgatctttatctcttaaatcatcaacctccacctgatacataacatcatgctgcatcattcattcaacctgattcgcaactcatgttgcatcattcattgttgtttcacctaagctttggcatcagcgtctaggtcacccatctatgtcaagggttcaggctttatcttcaaatttacatattcctcagaaactttctgaatttcattgtaagatttgtcatttgtcaaaacaaaaatgtttatcttttgtttctaataataaaatttatgaggaacctttccctttgattcatattgatgtttggggtccttttcatattacttctgtcaaaggatatcgttaatttcttactattgtcgatgattgcactagaatgacatggatttatttcttgcgtttaaagagtgatgttacaacgatttttccagaatttttaaaacttgttcaaactcaatttggttgcacagtgaaaagcataaggtctgataatgcacctgaattacaatttaaggacttactagcaacttttggcatttttcattatcattcatgtgcatacacacctcagcaaaattatgttgtcgaacgtaatcatcaacatcttctgaatgttgctagatctctttattttcaatcaaatattccacttgcatattggcctgaatgtgtttctactgctgcatttcttataaatagaacaccaactcccaatttagaacacaagagtccttatgaggttctttataagaaattacctgactataattctcttagggtgttttgttgtttatgttatgcatctacacatcaacatgaacgtcataaatttactgaaagagctacaagttgtgtttttattggttatgaatctggttttaaaggatataaaattctggatcttgagtcaaatactgtttctgttacacgcaatgttgttttccatgaaactatttttccttttattgataagcatagtactcaaaacgtttctttctttgatgattctgttttacctatttctgaaaaacaaaaagaaaatcgttttcaaatttatgattatttcaatgttttaaatcttgaggtttgtcctgtcattgagcctactactgttccagcacacacacacacacgatccctcgctccattaagcactactgttactaatgatcaatttgggaatgacatggacaacacgctcatgccccgcaaagagacacgtgctcctagttatctttcacagtaccattgctctaatgttctaaaagagccttcatcttctcttcatggtactgctcattctctctcttcccatttgtcttacgataaactctctaatgagtatcgtctgttctgctttgccattattgctgagaaagaaccaaccacctttaaagaggcagctttgttgcaaaaatggcttgatgcgatgaacgtggagttggatgctcttgtgagtacttccaccagggaaatttgttctttacatgatggaaaacgtgccattggttgtaagtgggtcttcaagattaagtataaatccgatggtactattgaacggtataaggctcgcttggttgctaatggctatactcaacaagaaggagttgattacattgatacattcagccctatagctaagcttacttcagtcagattaattcttgctttagctgctattcataactggagtattagtcagatggatgttactaatgcttttctccatggtgattttgaggaggagatatatatgcagctaccacaaggttatacaccaagaaagggggagcttttaccaaagagacctgtgtgtcgcctagtcaagtccttatatggtctaaaacaagcttcacgtcaatggtttcacaagttctccggtgttctaatacagaatggttttatgcaatctctctttgatccgactttgtttgtcagagttagagaagatacttttctggccctgttggtatatgtggatgatataatgcttgttagtaacaaggattctgctgtgatagaagtcaagcagatattagccaaggagttcaagctaaaagatttgggacagaagagatattttttgggattggaaatagctcgctctaaggaaggtatctctatttcacagagaaaatatgctttggagttacttgaagagtttggctttcttggttgtaaacctgtcccaacaccaatggagctgaaccttaagctaagtcaggaagatggtgccttacttctggatgcctcacattacagaaaactcattggaagattggtttatctcactgtgactcgaccagatatatgttttgcagtgaataaactcaatcagtatatgagtgctcctagggaaccacatttgatggctgcacgtcgcatcttacgttatttgaagaatgatccaggtcaaggtgtgttctatccagcctcttctactctgacttttcgtgcttttgctgatgctgattggagtaattgcccggagagtagcatatccatttctatagtttgtgtgtttcttggagattccttaatctcttggaaatccaagaaacaagatgctgttagtcgcagtttggctgaagctgagtacagaagcatggctagtgctatctttgcctgacacaatttttgtgtactatgacgatgagtctgcacttcacattgctaagaactcagtcttccatgagagtaccaagaacttccttcatgatattcatgtggtgcgagaaaaggttgctgttggttttatcaagactcttcatgttgatactgaacacaacattgttgatctgctcactaagcctctcacagcgcttcggttcaattatctactgtccaagatgggactgcatcacttgtactctccatcttgagggggag

>ATCOPIA54_LTR

tgttgggttaagtttgaatatgacttgagcactaagtcattaaatccttgagagattaggactagaatatctaggagtgatctagaaaatatttaggagttatctaaatatacttctattaagattatgattgtttaagagatctctatataaggagatgtaagtatgttgcgtaacttatgagttgagagaattaagaacaagagagtttaagttttgagtagttttcttaaactaattaagaaagttattctttaatctttgtgttcttgacaatttcaaactata

>ATCOPIA54_IN

tttggtatcagagcggttataatcattttgtgatcaagagagattagaagagagtagagatcaacgtttcaagctagaaacatgggtgacatagttgtggcaaaaccaaaggagaatatctcatcatcaataacatgtcctatgctcaatgctacgaactacacgggttgggccatacgtatggagattacgcttagtatacataaggtgtgggaggtaataaatccaggatctgatgatgttgacaagaatctcatggctaggggtttcatattgcaacctataccagagactttgacactacaagtcgggaatcttaatacaacaaaaaaagtatgggaatcaataaaaactcgacatgtaggagtggagagggtcaaagaagcaaggttacaaaccttgatggcagagtttgagaaaataaagatgaaggaaagaacatattgataacttcgttggaagactttcggaactctctacaaaatctgcggaactaggagttgagattgaagtaccaagactcgttaagaaatttcttaacggtttgccaagaaagagatatatacaaacaagttcttgaccttaataatacaagatttgaggatattgtgggccgtatgaaagtatatgaagaataagttggtgatgtaggagatgagcaagatgacataagaaaactcatgtaagttaatactaattcacaatcctatcaagataactgctagtagaggaagaggtcaaagaggacgatttggtggaagaggaagaggacgtggtcgtaatacaagagataagtcaaagatcatgtgttacaggtgtgataagatagggcattatgcttctaattgtccagatagattacttaagcttcaagaagcatgtgcaaacaagaagaagaaactcaagaagcggatgagctcatgatacatgaggtagtctatttaaatgaaaagaatgtcaaacttttgaaacacaatcagatggagataatgtgtggtatcttgacaacggggcaaggaatcacatgacagaaaaccgttcttatttctctaaaatcgacgagtcaatcacagggaaagtgagatttggagataactctcgtattgatatcaaagggaagggctcaatactctttgtaagtagaagtgtactacataccggatctaaagagcaatatcataagtcgtggtcaagccaccaaagcaggatgcgatgtgaggatgaaagaaaactatctaacattgtatgatcgtgatggaaagttgttggtgaaagcgataaggtcaaagaatcggctttacaaagttaccatggaaaccgaagctaagaagtgtttacaactaaatcttatcgacgattcatcaatatggcactcaaggttaggacatgttgggttaaacactatgaggtgaatgatgaacaaagagttagttgtcgggttaccaaagatcacagtcgaaaaggaaacattggcctcatgttcgcttgggaaataagtaagaagaatattccctcaagctacttcttttcgagcctcacgactacttgaactcatacatgcggatctctgcggacctatcacacctatgacagcagcacaaaataggtatatctttgttcttatcgacgatcactctcgttatatgtggacagtgctattgaaggaaaagagtcaatcattcgacaaattcaaaaaatttaaagcactagttgaagaagaaacaggagcaaagatcaaaacacttcgtatagatagaggtggtgagttcacttcacatgaatttcaagatttttgtgataaatccggaatcacaagatatttgtgataaatccagaatcacaagacacataactgcaccttactcaccacaacaaaacggagttgttgaaaggaggaatagaaatttgctagagatgaccataagcatcatgaagcacatggatgtaccaaactatctatggggagaaccagtgaggcatgctaccaatctttttaatagagtcgcaataagatcactggttaaacaaactccatatgaggtattcaagggaagaaggccaaatattgaacatttacgtgtgttcgggtgtatcggatatgcatagactgagagtccacagttaaagaagctagatgacaggtcgagaaggttagttcatctgggaacagaacctggctctaaagcttatcgcttgttggatccatctaggcggagaattattgtgagtagggatgtcgtttttgatgagagtaaaaactggtcttggaatgagacaaaaaacgagacaagtgagagcccatgaacgtttaaagtcagctttggaaacaatggtattgaaaatgaggactcagtacaagaaacagaggagaacggagccgatgagaataacgagggttcagttgaagaggaagaagacattccaaacgataacgatcaagatgaacagactaatgaggtcatcttaaggagatcagagagacaacgtcatagacctaatcatcaagatgactatattttgtttgctgaacttgaagtcgaaaaactcttgatgacaatcagtgaagaaccatgggattacattgaagcaaaagagctaaaggtatggagagactcgtgtagaagaaatcatgtctattaccaaaaataaaacatgggacctagtagaacttccagtcggagtcaaggctataggactaaagtgggtgtttaaactaaagcaaaattctgatggtagtattaacaagcataaagcaaggttgtagcaaaatgttacatacaaagacatggaatagattatgacgaagtcttcactccggtagcaagaaaagaaaccattcgccttatgcttgttcttgctgcttcacatggatagcaagttcaccacctcgatgtcaaaacggcgtttctacatggggagctgaaagaagaagtttatgttatacaaccggagggttttgttacaagagggagtgaggagaaagtttataagttaaacaaagcattgtgtggcctcaaacaagcgcctagggcctagaatcataagcttaactcgatacttaatgagttaaagtttgtcaagtgtcctaaggaaccttcattgtatcagaaacaagacaaagataaagttcttctagttgcagtctatgtggatgatctattaatctcggggtttagcttgaagttgattctcgagttcaagaaggaaatggcgaaaaaattcgggatgagtgaccttggtttgttaacatactatctcggtcttgagtatgtcaacacgaaggaggtattacgttgaagcaagaaaagtatgcatcaaaaattctaagtgaaactcaaatggaagaatgcaatgttgtagacataccaatgaacgcgaacttaaagctaagtaaagcacatgatgagaaaaacatcgatgagaaggagtatagaagaaatatcgggtgccttcgatatttacttcatacaagccctgatctttcttatagtgttggagtcttgagcaggtacatgcatgaaccaaaggagtctcatggtgcagctctaaaacaaatacttaggtactgacaaggtacacgggcttatggtctctccttcactcagaaaaacgaagccaagttgataggcttcagtgatagcagtcacaacgttgatgaggacgatggaaggaaaacaatatgtcacattttctatctcaacaagtgtctgatcacttggtgctcgcaaaagcaagataatgtggctttatcatcatgtgaggccgagtttatggccgctactgaggcagcaaaataagcactatgacatcaagagcttcttggagagatcaatggaaaaccatgcgagaagatgctgattttacttgacaacaaatctgcaattgcactcaccaagaacccggtgtttcacggacgaagtaagcatatacacaaaaggtatcattttattcgtgagtttgtgacgaatgaacaagtggaggtagagcacgttcctagaaaaagaccaaaggcagatattctaaccaaggctctaggaaggatcaagtttaaagaaatgagggagctagttggagttcaagatgtgtcgaagtatggcttcaaacttaagagggtgaa

>ATCOPIA55_LTR

tgtaaaggagaagcttcagtcaacgacaacagagattgggtcaaaccctctgctcagctcagagtctcccaaacaaaatgctatgggccaagacaccaagcccaacaccattcaacataacgaaaagcccaacaacagaagattgcctctgcaactcacaaacgacaaagagaacagagttgcaggcgtgaaggagagagccgttgcaaaagcaacaatgctgagctgtgaaaccgtaccgaaggtagagacaaagaacagatatgacgctcttaacctcctgtgctcaaacgacatgtgttaaggcacctagggtttatgagaggttttgagttgtataaataagagagacaatcgcttgtaatcaactaagagagaagagcttaataaagttataatatttctccttcattctaagtttcttaca

>ATCOPIA55_IN

tggtatcagagccatggagcaacctattgagctctattctcaaccactcttaaacatttcaaattgtgttactgtcaaactaaatggaaggaactatcttctgtggaaaacacagtttgaatcgtttctctccggccaaggtttactgggtttcgtcaccggcgctctcaaaccaccagatcctgttcttgcgactccactcaccgctgaagctgcagctgtggagacagtgaaccctgcgtatctctcttgggtgaaatctgatcaagtggtccggtcatggcttcttggatctctgtctgaagacattctctctgaagtcgtcaacacaaccacgtctcaggaggtatggctagctctagcaaaacatttcaatcgtgtttcttcttcacgcttgtttgaactacaaagaaagttacaaaccattgaaaagcgtgacagatccatgagtgattatttgaaagagattaagtctatctgtgagcaacttgcttctgttggcagtccagtgaatgaaaagatgaaaatttttgctgccttacatggtctaggcagagagtacgaaccgattaagacatctattgaggggtccatggatactgttcctacaacctttgaagacatctctcctcgtcttactggttttgatgatcgtcttttggcttacactgacgctgcaagcatcactcctcatcttgcattcaatacacagcgttatgactcaacaacctactacaacaaaggcagaggcagttcatctcaaaagtccaaagggcgtggaggctatacaacacaaggaaggggatttcatcagcaaatctcctctggttcttctgtgtcttcgggtcagtctgttgaaagaccagtgtgtcagatttgtggaaaaataggacatccggctctaaagtgttggcatcgctttgacaatgcatatcagcatgaagatatgccaactgctctcgctgctctccgaatcactgatgtcacagatcaagcaggcagtgaatggtgtgcagactctgcagctactgctcatgttacaagctcacctcatcacctgcagcagagtagagcttattcaggatctgacacggtcatggtaggagatgggaacttcttaccaatcactcacacagggtctgctctcttaccaacgacatcaggtactctccctcttcttgatgttttagttgtccctgatattgcaaagtctctgttatcagtttcaaaactcacaaccgattacccatgtactcttgaatttgatgctaatggggtcattgtaaaggacaaggtaacaaagaggcttctcactctgggtcaaaataagaatggtctgtacacgctgaaggatccacctgttcaagccttctattcatctagacagcaagcagcctcagatgaagtgtggcatagacgtcttggacatccgaatagtaagatcctgcagcagttagtcagtactaaagctatcatcatcaataagagcaccaataggatgtgtgaatcatgtcagattgggaagagtagtagactttctttttcagattctcagtttgttgcaactagactactagagagagttcattgtgatctttggggaccctctccagttttgtcaaatcaggggtttaagtactatgtaatcttcattgaccattggtctcgttattgctggttttatcctttgaaatgcaaggctgatttctacattactttctgcaagttccaaaagtttgttgaaacacagtttaatcaaaagatcagtacctttcaatgtgatggagggggtgaatttataagccatagatttctcaaacatttagaggaaagtggtatacaacagtcaatatcgtgtccttacacgcctcagcaaaatagacttgctgagaggaagcacagacacatcacagagcttgggctgtcaatgctgttctcagctaagctgccacaaaaagtttgggtggaagcgttcttcacttcaaatttcctgagcaacattcttcctacaactactctaccaaatcagatgagtccatttgagagattacatggccatcaaccggaatattcagctttaagaacctttggctgcagttgttttcccactctaagaaactatgcatcaaataagtttgaccctcgttctcttaagtgcgtgttcttgggctacaatgatcgctataaaggctatagatgcatctatcctccaacaggaagagtttatattagccgccatgtgatcttcgatgagtcttcttttcctttccaagatacctatcttcacctgcagaacttgggatcaacaaagcttcttgaagcgtggcaacagaatttcatgccttctcaaaagaatcaaagtgaaactcaagctgcttctgtgttctctgaagacgactttcctcctctaccagtcacacgggttcaagtttcaccaccaaatgtcacacctcaagctgctcagtccacagtacaacgagaagaacaacctgcagatacagacattcaatcaaactcaccaagaaatcaagccgagtcaccggctcttgtggacagagagtgcattgagcgtacgacaggctcagatcctgcttctataggcgacaacgctctcagtccacaagacagtgccactcaacgttctcctgttcagtcaacagaaacagctggaacttcagatcaaaatcagaggacagaagctgcagttgatccggttcagcaagttcacccaatggtaacaagatcaaagaagggagtagtcaaaccaaaccccagatacgtccttctaacacagaaagcatcacatccagaaccaaaaactgtgacacaagcactgaaacatgaaggctggaaaggtgctatgggcgaagaaattgacacttgtgttgaaaccaacactttttctttagtcccatacacacctgacatgaatgttttaggaagtaaatgggtgttcagaaccaaaataaatgctgatggcagtttgaacaagttgaaagctagactagtggctaaaggatatcaccaagaagaaggaatagactacttggagacctacagtccagttgtgagaacagccacagtgagacttgtcttacatatagcaacagtgatggaatggaatctgaaacagttggatgtgaagaatgctttcttacatggagacttaaatgaaacagtctttatgcatcaaccagctggatttgtggataagacaaaaccaaatcatgtttggcatctccacaaatctatatacgggttaaaacaatctccccgagcctggtatgataagtttactaactacttgttggagtttggttttgtttgcagcatacaagatccatcactattcttctatgaacaaggacgagatgtgctcattctacttttgtatgtagatgatatagtcctaaccggtagcaacaacattctcatggatagacttctgcaggaaatgagcaaggagtttcgaatgactgacatgggatctctgcaatactttctcgggattcaagcacagaactctgaccaaggcttgttcttatctcaacagaagtatgctgaggatcttctacaagtcgcaggaatgatcgattgtgcaccaatgcctactcctttgccagttcaacttcacaaagttcctaaacaaaatgagctattctcaaactccacttacttccgcagtttggctggcaagcttcagtatctgacattgactaggccagatattcagttttcagtaaacttcgtatgtcaaaagatgcacgctccaacaacagctgattacaatctgcttaagaggatccttaggtatgtaaagggaaccataaccatggggttactcttcaacaagaacacagacttcactcttcgaacctacactgacggtgactatagtcaacactcaaagcaaaagaagtctgctacaaataatgatgcagtcttcaagcttcgagccttcagtgatagtgatgagaaacaagacgttctacaggaggattctgtacctttcttggcaacaatatcatctcctggtcgtcgaagaagcaaccaactgtctccaagagctcaacagaagccgagtataaagccttgtcagatacaacttctgaaatcatctggctcaataacatgctcagagatctccacattccacaacctgatccaccggagctctatggagacaacctttcctccatctatcttgctgcaaacccggtacttcacacacgctctaaacactttcaaactcactatcattttgttagagaaagggtagcgttgggttcgttgattgtcaagcatgtgccatcccaccagcagttggctgatatattcaccaagccattgcccttcgatgctttcacttcgctaaggtacaaactgggtgtagatttgccacccacaccaagtttgcgggggag

>ATCOPIA56_LTR

tgtgagttttggtctaaattcaccaagtatgagaaagactctattcggtatagagggttcgaagttcaaatgaaaagcaaggaagagataagagtttctgggtcgcttagagggttcactttatgcgccaagtttgagaaacaaaaggagatctcttcagctctctctcatgacatcacattgtcgagttcgtacaagcaacaagagcagacaaccagaatggtttcttcaatcaagccgcctaagagattgcagcaattcaggcccgtaattgcagctggagcacctgacttgtgtaaggttcaagcacgagcgtgtcgggtagctctagatccttctctttgtctctttaaaggagttggttgtgttgtgttgctttgtaaggtgtgagagaagtttgtaacagagagagaaagttgtagaaccctaaaggctcaagataagagcaagttcttagtggatttccgagaatatctcggcgagacgtagggattcaagtttgaatcctgaactcgttaattcctgtgtgttcttactttcaagttccgctttttacaaacgagtgaacaacagagtgagttgagaggtctgagattagacaaagtacaactcgagaacgttctaatcctcaca

>ATCOPIA56_IN

gtttggtatcagagcttccaggttactacctaggagcgagaagatctgctcaagatgtcttcgggcagagcagaggtggagaagttcgacggagatggggattacatcctgtggaaagaaaagttactggctcatatggagatgttgggacttctggagggtctcggggaagaagaggaagcagaggttgaagattctaccactgagattagtgatggaggaaaccaagacccagaaactgcaacttctaaactggaagacaagatcctcaaagaaaaaagaggaaaagccagatctaccatcatcttgagcctgggaaacaatgttctgagaaaggtcatcaaacagaagacagcagcaggtatgataaaggtcctggatcagttatttatggcaaaatctcttccaaatcgcatttacttgaagcagaggctgtatggctacaagatgagtgagaatatgacgatggaggagaatgttaatgatttcttcaagttaatatcggacttggaaaacgtaaaggttgtagtcccagatgaagatcaagccatagtcttgctcatgtctttaccaagacagtttgatcaactgaaggagacactgaagtactgcaagactacacttcatctcgaagaaatcacaagtgccataaggtctaagatcttggagttgggagctagtggtaagcttctcaagaataactcagatgggttgtttgttcaagacagaggcagatcagaaaccagggggaaaggaccgaacaagaacaagagcagatctaagtcaaagggagcaggaaaaacgtgttggatctgtggcaaggagggtcatttcaagaagcaatgctatgtatggaaggagaggaacaagcaaggttccacatctgaaagaggagaggcttctactgtaactgctcaagtcactgatgcagctgcactagtagtttcaagagctttacttggctttgctgaagtcaccccagatacatggattctagacacagggtgttccttccatatgacctgcagaaaggattggatcatagacttcaaggagactgcaagcgggaaagtaaggatgggcaatgatacttattctgaagtgaaaggaattggggatgtcagaatcaagaatgaggatggatctactatcttgctcactgatgtcaggtacataccagaaatgtcaaagaacctcatctcacttggaactcttgaagataaaggctgctggttcgaatcgaagaaaggtattttgactatttttaagaatgatcttactgtactaactggaaagaaagagagtactttgtattttctccagggaacgacacttgcaggtgaagccaatgtcatagacaaagaaaaggatgaaacaagtttatggcacagcaggcttggtcacattggtgcaaaagggctgcaggttttggtcagtaaaggtcatctggataagaacatgattaaagatttgcagttttgtgaagattgtgtgtatggaaaaacacgcagggttagctttggagctgcaaagcatgtcacaaaagataaactcgactatgtgcattctgatctatggggatcaccgaatgtaccattctccattggtaagtgtcagtatttcatcactttcattgatgattttacgaggagaacttggatctatttcattagaaccaaagatgaagctttcagcaagtttgtagaatggaaaacacagattgaaaaccaacaggacaagaagctcaagattctcagaacagataatgggctggagttctgtaaccaggagtttgattcattctgcagaaaagaaggagttataaggcacaggacatgtgcttacacaccacagcagaatggtgttgctgaaaggatgaacaggaccatcatgaacaaggtcagatgcatgttaagtgaatcagggttggggaaacagttctgggcagaagcagcgtctactgccgtgttcctcatcaacaaaagcccaagctcttcaatagagtttgatattcctgaagagaagtggactggtcatccaccagattacaagatactcaagaagtttggatcagtcgcttatattcattcagatcaaggaaagctgaatcctagagcaaagaaggggatttttctcggatatccagatggtgtaaagggattcaaagtgtggctgctagaagacaggaaatgtgtagtctctcgagacattgtttttcaagaaaatcagatgtacaaggaactgcagaagaatgatatgtctgaggaagaaaaacagctcactgaagtagaaaggactctcatagagctaaagaatttgtctgcagatgatgaaaatcagagtgaaggaggagataagtcaaaccaagaacaagcttcaacaacaagatctgcaagtaaagacaaacaagtagaggaaactgattctgatgatgattgtctagagaactatctactggccagggatagaattcgaagacagatcagagctccacagagattcgttgaggaagatgacagccttgttgggtttgcattaacaatgacagaagatggagaagtttatgaaccagaaacctatgaagaagccatgagaagtccagaatgtgagaaatggaagcaagctaccatagaagaaatggactccatgaaaaagaatgacacatgggatgtcattgataagcctgaaggaaagagagttataggctgtaagtggatattcaagagaaaagcaggaattcccggagtagaaccaccaagatacaaagctaggcttgtcgccaaaggattttcacaaagagaaggcatagactatcaggagattttctcacctgtagtcaagcacgtgtcaatcaggtatcttttatccattgtggttcaatttgacatggaattagaacagcttgatgttaagactgcgtttttacatgggaatctggatgagtatatattgatgagtcagcctgaaggatatgaagatgaggacagcacagaaaaagtctgtttgttaaagaaatctctgtatgggctgaagcagtctccaagacagtggaatcagagatttgactcattcatgatcaactcaggttatcaaagaagcaagtataatccatgtgtctacacacaacaacttaatgatggatcgtacatctatctactgttgtatgtagatgatatgctcattgcatcacaaaacaaggaccaaatccagaagttaaaagagtcactcaacagagaatttgagatgaaggatttagggcctgcaagaaagatactgggaatggaaatcacaagaaacagagaacaaggcactttggacctgtctcagagtgagtatgtggctggagtgttgagagcttttgggatggatcaaagtaaggtctctcagacgccacttggtgcacacttcaagttaagagccgcaaatgagaaaactcttgcaagagatgctgagtatatgaagtcggttccctaccctaatgcaattggaagtatcatgtactctatgataggatcaaggccagacttggcatatcatgtgggggttgtaagccggtttatgagtaaaccctcaaaagaacactggcaagctgttaagtgggtcatgaggtacatgaagggaacacaagatacctgtctaaggttcaagaaagatgacaaatttgaaatcagaggctactgcgattcagattatgcaactgatttagacaggaggagatcgattacaggatttgtattcacagctggtgggaatacaataagctggaagtcgggtttacagagagtggtggctctgtcaacaacagaagctgaatatatggcccttgcagaggcagttaaagaagccatttggctaagagggttagctgcagagatggggtttgaacaagatgcagtagaagttatgtgtgattcacacaatgccattgctttgtccaagaactcagtccaccatgagaggacaaagcatatagacgtgaggtatcacttcataagggagaagatagcagacggagagattcaggttgttaaggtttcaacaacatggaatcctgcagacatcttcacaaaaacagttccagtgagtaagcttcaagaagcgctgaagctactcagggtctcaagtaactagggagaccacagatccgagattggaagtcaagtaacactaagaagatgagttcagtgaactttataccaaggaggagttt

>ATCOPIA57_LTR

tgttagtttataattgagtttggtctataagttataattatgttgtattaaaaggtgtttgttagagtcgtgacttttagattaggttgtgtcgtttatcttagagagaaagagacacaactctttacttattgtgtctatttgttttctatttaaactttttgtatcgtgatgaaacgaacaagttctccattcaataaaagtcagtttctcttttgctttgtgtatctcataagaaacagaacaacaagtaaacacttgttttttctttgtaacaagaggcttcaatttccaaca

>ATCOPIA57_IN

tggtatcagagccctactgattcaagggccaaacatttcaagtaaagagtttaataaacacgagagagttatggagatggtgtcacacattgtgactccaatcttcaacaaagagaattatggtttttggcgcatcaagatgaaaacaattttccagacaaagaaactgtgggaaattgttgacgaaggagttccaaaaccacccgcagaaggagatcatagccctgaagcagtgcaacaaaagacacgatgtgaagcagcatccttaaaggatctaaccgctttgcaaattcttcaaactgccgtctcagattccatatttccgagaatcgcccctgcatcaagcgcacttggaaagccttggtattggaatttcaaggatcaccacaagtcaggatgataaagcttcaatctcttagaagggagtatgagaatttgaagatgaaagaaagtgacaatatcaacactttcatgaccaagttgattgagatgggaaatcaactgagagttcatggagaagagaaatcagactatcaaatagtgcaaaaaatcctaatttctcttccgaaaagatttgacatcatagtggctatgatgaagcaaacgaaggatctgacttcgttgtccgtaacatagttgataggcacattgaaagcacacgagaagcgggtagagatgcgagacgaatgatcaacggaaggagcgttctatggagaaaacaaacgtgaagacaagcgtcaaacgcaggctgggaaatggtgtgacgtttgcgaacgtaaaaatcacaatgagagtgattgctggatgaagaagaacaaaggtgttctttcgcaacaagtggggaacaatgagagaagatgtttcgtgtgtaacaaaccgggacatctagcaaagaactgcagacttagaaggaccgagcgagtggatctaagtctagaagaaacaaatgatgatgaggatcacatgctgttcagtgcagtcgaagaagaaacatcatcaaacgtgaatgatgagacatggctagttgacagcggatgcactaaccatatgaccaaggaagttaaatacttcatcactcttgatcaaagtgtaaaggtaccgatcaaagtggggaatggtcaacatgtaatgacggcaggaaaaggaaatatccaagtgatgacaagccaatgagagaagatcatcaaagaggtgtttctagtgccgggtttagcaagaaatctgttgactgtttctcaaatggtatccaagggatacagagtcttatttgaagacaacagatgtctcatcaacgatcctcaaggaagaaggatactggatatgaagatgatgcagaagagttttccattgagatggataaaggcgaacacaagtgctttactagcaagtgaagaaggagtaaacaaggcaaggttcgaagaactaaggaggaagcttggagtaagaccgaaactcaattaagggggaa

>ATCOPIA58_LTR

tgttagtgcaccctctgtcaggacaaaccagaaggagtgctgattgctggaacaagctggaagctgtttcagaagactaaagtgaagatcaagtaattgatgctgacgtggatatgccgccaaatgaacgaatgacgtggagatcacaacaggatgagttcagtttggagaggagatattcacgcgaagatttggagtgatccaagatctcgaaggctaacctagaagaccaaagacctcgatggtataaatagaggtgctaaggttcttgttagacttggccgtgattgtgagtgaaaaagctagtgcaacgcttttcattrttagagactaaggtttagtctgattggaattctagagagatcaaagagtgtgttcttgtgatctgctcttgtgtgtttagggaaatctcaggtgacaagctaaagtagtttagatcttgtgtaaaaccaattaaggtagttgtaaacttcttaatcagatttctaataaagtgatactcagtggttctgagtttggggaatacgattcttggttcatccggaatttca

>ATCOPIA58_IN

tttggtatcagagcgggcatctgaaccaagttgtacttaacaacaggtgcagatcctgcggagaggatggactaccccaaagagttcgttgcggtcggtaaagcaatcatgttggaaaaaggaaattacggacactggaaagtgaagatgagagctctcatacgtggtctaggaaaggaagcctggattgctacgagcattggatggaaggctccggtcatcaagggagaagatggagaagatgtgctaaaaactgaagatcaatggaatgatgcagaagaggcaaaggccacagctaattcaagggcactgtctttgatattcaactccgtgaatcaaaatcaattcaagcggattcaaaactgtgaatcagctaaagaagcatgggataaacttgctaaagcatatgaagggacaagtagtgtcaaaagatccagaatcgacatgttagcatctcagtttgaaaatctcagtatggaagaaacagagaacattgaggagttcagtgggaaaatcagtgccatagcgagtgaagcacacaatctaggaaagaaatacaaagataagaaactggtcaagaaactgttgaggtgtctcccatcaaggtttgaaagtaagagaacggccatgggaacgtcgttggacactgactcaatcgattttgaagaagtagtgggaatgctccaagcatatgaattagaaatcacttccggaaagggaggttactccaaaggacttgctttagctgcatcggcaaagaagaatgagatacaggaattgaaggatacaatgagcatgaagaatgagatacaggaattgaaggatacaataagcatgatggcaaaagacttcagtagagcaatgaggagagttgagaagaaaggattcggaagaaatcagggaactgatagatatcgagaccgaagttcaaaaagggatgagattcaatgtcatgaatgtcaaggatacggacatattaaagctgaatgtccctccttaaagagaaaagatctcaagtgctctgagtgtaagggtcttggacacactaagttcgactgtgttggatcaaagtctaagcctgatagatcctgcagttctgaaagtgaaagtgactcaaatgatggagactcggaagattatataaaaggtttcgtgtcttttgtaggaatcattgaagaaaaagatgaaagttcagacagtgaagcagatggtgaggatgaggacaactcagctgatgaggattctgacatcgaaaaggacgttaacatcaatgaagagttcaggaaactgtatgacagctggttgatgctgagtaaagagaaagttgcctggctggaagagaagctaaaagttcaagaactgacagaaaagctgaaaggagagttaactgctgcaaatcagaagaactctgagctgactcagaaatgcagtgtggctgaagagaaaaacagagaactttctcaagagcttagtgacactcgcaagaagatccacatgctgaacagtggaacaaaagatttggatagtatacttgctgctggaagagtgggaaaatcaaattttggtttaggatacaatggtgctggatcaggtacaaagacgaattttgtacgaagcgaagctgctgctccaacaaaaagtcaaacaggttttcgaagcaactatgatgctgttccagcaagacgcgtgtaccagaatcacgatcactatcattcccggagaactgtgacaggttacgaatgttactactgtggaagacatggtcatattcagagatattgctacaggtatgctgctaggttgaataagctgaagagacaaggaaaactatatccatatcaaggaagaacctccaagatgtatgtcagaagggaggatctctattgtcatgtagcatacacctcgattgaagaaggaataaagaaaccatggtattttgacagtggagcatccagacatatgacaggaagtcaatccaatcttgaaaattacacctctgtcaaggaaagtaaagttacttttggaggtggggataaaggaaaaatcaagggaaaaggtgatttgactaaagcagaaaagcctcagcttacaaatgtgtactttgtcgaagggcttactgcaaatctgattagtgtgagtcagctatgtgatgaagggctgactgtgagtttcaatagtgtaaaatgctgggctacaaacgagaagaaccaaaacactctcactggagttagaactgggaacaattgctacatgtgggaagaacctaaagagtgtcttagagctgaaaaagaggatccagtggtatggcatcaacgtcttggtcacatgaatgcgaggagcatgtcagaaatagtgagcaaggaaatggttagaggagtacaagagctgaaacacatagagaaaattgtgtacgatgcctacaatcaaggtaaacagattagagtccaacacaagagagttgtaggtgttgttgagagaaagaaccagacttttcaagagatggccagagccatgattcatggacatggagttcctgaaaaattctggacagaggctatcagtacagcatgttatgtgataaatcatgtttatgtgaggattggaggcacattcgacaagttggtcaaagcgtttgtgaagacaatgacaactgagttcaggttgagtatggtaggcgaacttaagtactttctggggttgcaaatcaatcagattgatgaagggattgctatctcgcaaagcacctatgctcagaacctggtgaaacgcttcgatatgtgttccagcaatccagttgaaactcctatgagcactaccaacctctgcagctgctgtactaaaattctttggatgaaacacatgggtttggattacggtatgtcattttctgaccctttacttgttaaatgtgataatgaaagtgctattgccatatctaagaatccggtacaacactcaatcactaaacacatagctataagacatcattttgttagagaattagttgaagaaaaacaaattaccgtagaacatgtgcctactgaaattcaacttgctgatattttcactaagcctttggacttgaacatgtttgtgaacttgcaaaagtccctgggtattggtgaagtctaactatcttcttgatgagtgtttgtgctgaaacagggttggttgtacagtgagaagcaagttttcatatctattttacaaagtgtcataaccggttttcaaatcttctgctgtaaaaagttcaccaagagaaagagctacccttcatggttcggagatgcaaagtcagtaaagtgtgtgacagacatagtgtgtagagcattaaggaagggatgaaatgcagaaaggggcagaaaagaagcatttcatttcataagatgaagcatgagcttatctaagacacgggggacaaaggagaaatcttgtcactaacccgatgggaacaaaggagaaatcttgttcacctacactttggagagaagggatgtcaaacaaaaatattgagagaacaagtaagaaagagcatcactacactctgcaacaagacaagagaaaaaaaaaaaaaataaaaaaaaaaaaggggggtgcaaaaacaagttctctcactgacatctcgtctctctgcagaaaggagcagtaagaagcaacaataagttctgaatacaaaagatcagacttgtaatttatcaatagcctgaatgttgctgcacagtctgtcacactccttgttaaaaggactcgctaacactctgagatatgacggtgtgaagactactcttggagaatcaacattctcttttgtgttgtgtcagcagattatgttctgaatttttttgaatcaagttacaatgagcttagtattgtacaatcacctctgggacagttctggacctcagtatgagggagagatggttattactgtgctgatactaatggggacttgttctgtgtatatcaagatggcttcagaaaaatgagttgactttcagaggtacttagttcaattctataagctaattgggttgttcggttttgttttatggtggttattgtgttttatgagatttgaccggaccaaaatccggttagcactgatttttggttttgagtttctatggtttgcataaagggatctatcgaatgaaacaagtgagaaaacccagaaaattgagctctcacgccgaaacactagcgatgggagaacacgcagcgatgcccggcgtatgcgaaagatgtttaatccagagtatgtgtttggcacatccttacctgtagatttgagatatgtctctaccttgctgacgacaataacgacacatatggaaggtatggtgtcttggtcctgtgctcagtgtctggtttaattggtagagtgtctagatagagtttttgatcaattgtgtcatgcaagcagggggagattatggactggtccctgagaactctaattcatttgtaaactctctagatgttgataacgaagaaggctttgaagatactcaatcttcttagtgtgtttagatctagttgtgggggagtttaggattaagggggagtttttaagctgtgtttgttaatcatgtccttttggttttagtaggtctttgtttctaagtactggatgatgtgttttgaatcttaaaaacttatttatgcaatctatgatgagaccctctattggttatttccgctgcttattatctctatgacttgatgtgtgcttgtgagttttctttgaggggtttcttgcaggattgcttgaattgacaaattgtatcaaaaagggggagat

>ATCOPIA59_LTR

tgagagactccattgttgtaaagctctttgagcagattcttcttctttgtgttagtggattgccggatcgaatctggccccagacgtaggcttaatcatatcgattagctgaactgggtaacaaatttgtgttcttcgtttattgcttttaattttgttcttgagttgagattcaacgagtttaaagagagaattgaatcagagtgactgtgagcatgaatcgtaaca

>ATCOPIA59_IN

aagtggtatcggaacgttggttcgaaagattcgctcgatggtaatgacgatgaaggtcgagattaaaaccttcaatggcgatcgagacttctcgttttggaagataagaatagaagctcagctcggcgtactaggcttgaagaacagccttacggattttaaattgaccaagacagttcctgtggcaaagaaagaggaaaaagaatcagaatatgaagatgatgcatctgatataaagcaagctactgaagaaccagatccgattaagtttgagcagtctgaacaagcaaagaacttcatcattaatcacatcactgatacagttcttttaaaggttcaacactgtaagaccgctgcagagatctgggcaacattgaacaaattgttcatggaaacttctttgcttaatcgaatatacacacaattgaagttgtattcattcaagatggttgatactttgagcattgatcagaatgttgatgagtttttgaggattctggctgagttaggaagtttatcaatctatgttggaaaagaggttcaagctgtcttgatcaaaatttactgccatctagttacatccaacttaaacatactcttaagtatgggaacaagactctctcagtacagatgtggtatcatccgccaagtcacttgagagaaagttatcagagactcaagaaagcaacaaaaatgtgtccatggctctctacacaacagatagaggcagacctcaagtgaggaatcaggataagcaaggtcaaggaaagaatagaggtaggtctaactctaaaacgcgagtgacttgttggttttgtaaaaaggaagctcatgttaagagagattgttttgcgagaaagaagaagctggaaaatgagaatagagcaacaatgatacttttgggtgatgatcacacagtagaatcaagaggatgtggtaccatcaaattgaacactcatggaggtttaatcagaatgctgaaaaatgtcagatatgttcctaacctaagaagaaacttgatctctactgggacacttcactctcttggttacaaacatgaaggtggagaaggaaaactgagattctacaagaatggcaaaactgctttatgtggatacttgatgaacggtctctatattctggatggacacacagtagctactgaaacctgcaatgcagaaagtgctaagaacagcacaaagctgtggcatagcagacttggtcacatgagcataaataatatgaagatcctataaggcaaacagactgattgagaaaaaggaattcaaggacttggacttctgtgaacactatgttatgggaaaatcgaagaaactcagttttaatgttggtaaacatgttacagaagacatacttggatatgttcatgctgacttgtggggatcaccaaatgttactccatctatctcgggtaaacaatacttcttatccattatagatgacaaatctagaaaagtttggttaatgtttctgaaatcgaaagatgagacatttgataagttctgtgaatggaaggaaatagtagagaatcatgtgggaaagaaggtcaaaactctaagaactgacaatggattggagttttgtaataacagatttaatgactattgtgccaagactggcattgaaagacaccggacgtgtacctatactcctcagcagaatggagtgactaaaaggatgaatagaacaataatggaaaaggtgaggtgcatgcttgatgaatcaggtcttgaagaaacgttttgagctgagactgcagcaacagatgcatacttgattaacaggtcacctgcttcagcagttgatcacaatgtgccagagcagttgtggctcaacagagaacctggatataagcacctgagaagattcagttctatagcctatgttcatcaagaacaaggcaagttaaaaccgagagtattgaaaggagtgtttctcggatatcctcaaggcactaaaggttacaaggtgtggttaattgatgaagaaaaatgtgttatcagcagaaatgtggtgtttaatgaagattcagtgttcaaggacttacagtcaggaagcaaagatgaagatgaaacagtaacacaggaatctcagattgagattgaaacgccgccaaaatcagaacttgaaacacagaatcaggttcaaggtggagctactcaagtccagctcaatgattctgaagatgaatataatgatgttgaaggagtagttacatgaagcaatctgaagacttatcagttggctagagatagggtcagaagaacaattagaccacctgcaaaacttactgactatactcagtttgcttttgctctagtcatggcagaagaagttgaatcagaggaaccagtatgttttcatgatgttaaggaagacaaagactgggaaaaatggcatggaggcatgatagaagaaatggattctctgttgaaaaatgcaacctgggatatagtcgacaaacctaagaatcaaaaagtgataagctgtcattggttgtacaagaagaagcttggaattcctggtgttgagctaccaagatataaggcaaggttggtagcaagaggattctctcatagagaaggaattgactaccaagaagtgttctcacctgttgtgaaacacacatcaatcagaatcttactatccctaatggttaaagaggacatggaattagaacagatggatgtaaaaacagcatttttgcatggagaacttgatcaaacattgttcatggaacaacctgaaggctttgaggtaaatcctgaactagatcaagtatgtttgctaaagaaatctctttatggcctgaaacaggcacctagacagtggaataagcggttcaatgcattcatgatggatcagaagttcagtagaagtgtgagtgattcatgcgtctatgttaaagaggtaagcaatggtgaatgtgtctacttgctgctctatgttgatgatatgttgttagcagctaagagtatgacagagattaaaaagcttaagaaagttttgagtagagaatttgaaatgaaggatatgggagctgcaagtaggaaattgggaatagatatcataaggaataggagtgaaggaactctatgcttgtcccagactagttacttggaaagagtaattcagaagttcagaatggatggggctaaggttgtgaatactccaattggtgctcatttcaaattgtcatcagttcataacgatgatgaaagagtaggttctgagaaagttccctactctagtgttgtcggcagtctcatgtatgctatgattggaactagaccggatatagcttatgctattggactagttagcaggtttatgagcaagcaaggagaagtacattggacagcagtcaagtggttgttgaggtatcttaaatggtcaataggattaaacttgatgtatactaaaggatttgattttaaagtgcagggttattgcgattcagatcatgctgcagaccttgataagaacatgtctattagcggatatgttttcacagtgggaggcaacattgtgagctggaagtcatgtctgcaaccagttgttgcgctgtcaaccactgaagcagaatacatagctcttactaaggctgtaaaggaggctatgtggataagaaacttgttggatgatatgatgcttggaactgagactgctgaagtgtgaagtgactcacagtctgcattatgtttatcaaagaacaatgcttttcatgaaagaacaaagcacattggtaagaagtatcatttcatcagagacattattgaagctggtgaagtcgaagttcataagattcacacgactaggaatccagctgatatgctcacaaaaggtataccagtttcgaagtttgaagcagcattggagttcctgaagctgctcaggtaaacaggcgagaattcgccaaaaccagaggttacatccaagtaatgcaattcacttgaaggagaaagaaacttgaatcaaggtggagtgttgtgatgttggtttctgattcagttttaaggtttggtttaacctagaaattgattggagattgaagtcggtttagttcggtttgtctcggttatcaattgaagaagaaaagtaggaagtcaaagcctctagtcgcgatagagtttttgttgtttcaagtataaaacataaattgttattttcttctttattcattctgttatctctgctattgagagagagaagtaagagagagagagagagagagagagagagagagagct

>ATCOPIA5_IN

tggtatcagagcattacgactctcagacctaaacttttttttttaacgccgcctcttcttcatacactcttcttcaagtctgtttcatttttcacgatgacttccacaccgtcgggatccacagaaacaattgctttctctgaaacaccaacccttctcaatgtcaacatggcaaacatcacaaaactcactcccacgaattacatcatgtggaaccgtcaggttcacgctttgcttgatggatacgaccttgctggctacatcgacggatccgtcaccgcaccatctgaaatgatcaccactgctggtgtttctgcggctaatcccgcctacaagttttggaagcgtcaagacaagctgatttacagtgctatccttggcacaatcacgaccaccattcaacctctcctgtcaagatccaacacggctgcagagatctgggagaaactcaaatccatttacgccacaccaagctggggccacatccagcaaatgcgtcaacacatcaaacaatggagcaaaggtacaaaaacaatcactgagtactttcaaggtcacacaacccgtttcgatgagcttgctcttcttggaaagccacttgaacatgctgaacagattgagttccttcttggaggtctctcggaagattacaagtctgtggtcgaccaaacagagattcgtgacaagcctccaactctcaccgagcttctggagaagctcctcaaccgtgaagccaaactcatgtgtgctgctgcaacaacgccatcgcttcctgccactgctcatgctgcaaactacaaagggaactcgaacaacaatcaatacaacaacaacaatcgcaacaacaaatctcatggtcgcaacaacaacaactggcagcaaaatcaacctcaatagcgacagaaccagtacacaccaaaaccctatcaaggacgctgccaaatctgcagcattcatggacacagcgcacgtcggtgtcctcagctccaaggtagcagctacaactcgcaacagtcaatcccagcctcaccattcacaccgtggcagccacgagcaaatgcagctatagcctcaccatacaacgccaacaattggttactcgatagtggtgcgactcatcacatcacttctgacctgaacaatctctcccttcatcaaccatacactggcggtgaagatgttactattgctgacgggtctggtttatccatttcccacaccggttcggctttaatttcaacaccctctcgctctcttgcattgactgatgttttatatgtgcctaatattcacaaaaacctcatatcagtttaccgaatgtgtaacgctaacaaggtgtctgtggaattctttcctgcacattttcaggtgaaggatctcaaaacgggggtccaattgctccaaggcaggactaaagacgagttgtacgagtggccggtgaatccacctaaaccgtcatcccacttcactacaaccactccaaaaaccgacctcacctcttggcattcacgccttggccacccgtctttatctactttaaaagttgttgtttcccaattttctcttcctgtttctaattctttgcaaaaacagttcaactgttctgattgccttttgaataaaacacacaagcttccttttcatacaaacactattacctccactcaaccacttgaatatctatacattgatctttggacatcaccaatagtgtccattgacaacttcaaatactatctcgttattgtcgatcactatacacgatactcatggttctacccgatcaagcaaaagtcacatgtcaaagatgtgttcatgaccttcaaagccttggttgcaaacaaatttcaacgtaaaatcattcatctctactcagacaatggtggtgagttcattgctctccgctcctttctctcctcaaatggaatcactcatttaacgacaccacctcacacgcccgagcacaatggcatctctgaaaggaagcaccgccatatagtagaaactggtcttacacttcttggacaagcctctatgccgaaatcatactggagctatgcattcaccattgccatatacctcatcaatcgaatgtcaagtgatgtcattggcggcatttctccatacaagcggctctttggtcaagcccccaactatctcaaacttcgagtgtttgggtgcctctgtttcccgtggcttcgaccgtatactacacacaagcttgatgatcgacctgctccgtgtgtgtttctcggctactcacaaacccaaagcgcctatctttgcttgaatcgaaccaccggccgtgtctatacatctcgacatgtccaatttgttgaaaatacctacccatttaccaaacccacactcgacccattcaccaaccttgaagaatcaaacaaccactccataaccacaacagtcccgtcaccgccgttcgtacagcttccgtcggtaccaccccctacaagagatcctcaccagccgccgccttctcaaccggcgccgtcaccctctccgctctcgccgccttccatgtcgtcaccggtcatgacatcgtcgccacaattctcgagcaatcgagactcaacgacgctccatggagattattctcacgtggactatggcctatcatcaccttccaatccaccaggcccaattacatcacctaccacctcaaaaagcccatctgagcccacttccagcccatcacactcaaaccaacccaataaaactccaccgaattccccatcgtcttcttcctcttccccgactccaattccctctccatcaccacagtcatcaaattccccacccccacctccacaaaatcaacattccatgagaacccgagccaaaaacaacataacaaaaccaataaaaaaacttacccttgccgccactccaaaaggtaaatccaaaatcccaaccactgtggccgaagctctccgtgatccaaattggcgaaatgcgatgtcggaggaatttaatgcaggtctccggaacagcacatatgacttggtcccgccaaaaccacaccaaaactttgttggtactcgttggatttttactatcaaatataatcctgatggctctattaacaggtacaaagcacggtttctagctaaggggttccatcaacaacacggtcttgattactccaacacatttagccccgtcattaagtccaccacagttcaaactgtccttgatattgcggtcagtcgctcttgggatatacgacaactcgatatcaacaacgcctttttgcaaggccgtctaaccgaggatgtttacgttgcacaaccacccggtttcataaacccagacagacccaattatgtgtgtcacctcaagaaggcgctacatggactcaaacaagctccacgcgcttggtaccaagaattgcgtgggttccttctcacatgtggcttcacgaattccgtcgctaacacctccttgtttatacgccaacataacaaagactacatatacatacttgtgtatgttgatgattttctcatcacaggaagtaactccaatcttatcgctcaatttattacatgtttggctaatcgcttctctttaaaagatttgggccagctgagttactttcttgaaatcgaggcgacaaggacgaaagcaggattacacttaatgcaacgtcggtatgttcttgacttacttaccaaaacaaagatgttggacgccaaaacagtctccactccaatgtcgccaacaccgaagctgacgctaacatctggcactcccattgacaatcccggaggataccgacaaatacttggcagcctttagtacctcggattcacaagacctgacattgcctttgcagtcaaccgtctctcacaatttatgcacaaaccgaccgatctacattggcaggccatgaaacgcgtactccgttaccttgccgggactccatcgaatggtatttttcttcgcgccaaaaacccgttaacacttcatggcttctcagatgctgattgggcaggtgataatgaggattatgtttccaccaacgcatacatcatataccttggcggaaatcccatttcatggtcatccaaaaaacaacgtggcgtcgctagatcctcaaccgaagcagaataccgtgcagttgcaaacgcaggctccgaaattcgatggtcatctcacttctcactgaatttggaataccattgcccactactcccgtcatctattgcgataatgtcggagcaacatatctctccgccaatccagttttttattctcgtatgaaacacattgccttggactatcatttcatacgagacaatgttcaagctgggatactacgtgttgctcatatctccaccaacgaccaactcgctgatgctcttaccaagcctctctcccgccaacgtttcacagaaataaataacaagattggagtcatacaactgcctccatcttgaggaggtg

>ATCOPIA5_LTR

tatagaggttatgtatagttagggtataattgtaattagtattaccctaatctctcgtgtatatatattgtaatcacaacctctaatgataattaagcttcacactctata

>ATCOPIA60_LTR

tattagtgaaaccagaccactgcaaagttctgtggttacaaaagaagagaatgagaagaagcagacagaagttgagcaagtacaatctagatctgggaaaagagaagtcaaagcgatattgaagacgcaaacatcagcaggtcaaaacgaaactgtcttgctaataggctcaaagaatgttcggcccaagaagattgtcaagactgtgtacagaccaaagcccatctccaagcccaacaatacttgtactaatcagcttcctgtcacacaacggctacctgcagaaaaagacaataaagataaagataagcaggaggaagtttcacctcaggaactaaagacaaagaccagctgtcatgttcaacaaatcaaaacgacaaacagatttgcagtattagacattgaggtggaagcgtgatgacctaattctaaagtgcataaataagccttaagttgtatcagaaactctaagagagaaagatataaaagaagcaatgcttatcttattctccaaacactagacactgtgtttactttca

>ATCOPIA60_IN

tgaaatcccacaccactgatgaaaatttggctacaaagtcttgatctgccaacaaagggaaccagaccactgcaaagagatcaagactatgactcaaatacagaaaaggtatctactacgctatttactccagatgattttccaccattacctgtcagcagtcgtgctgtagtgattcctgagagtggagagcagagttctgagtgtacgacaggctttgatcctgcttctattggcaacaactctacctcttcaccaccagggactcctacagctacacaagcttcatcaacatcatctgaaaagagtaaaactccaacatcaataccatctacaccaacaacaccacaaaggtcatcaactgaaagtactgagacaccgatgtcctcacctgtcataacacaaccatcaccatctgcaagcagtattcctacaatgcatgcagctacatcctctgcttctacatctcaacaatcatcagtggcgagcaataaaagcactactgatgttgttcaaatacaagaagcttctccgaaaagtacagctccttgtatagaagctcccgtggaaaaacagcctacacatacaatggtaaccagatcaaaagctggcatccataaaccgaatccaagatatgcacttatgcttcataaagtgtcatatcctgaaccaaaaacagtcactgcagccatgaaagatgagggatggaataatgcaatgcatgaagaaatggataactataaagaagctcagacttggtcgctcataccttatacaccagacatgcatgttcttggctgcaagtgggtctttagaacaaagcttaatgctgatggatcccttgacaagctaaaagccaggatagttgcaaaaggctacgatcaagaggaaggcattgattatttggaaacctacagctcagtggtcagaacagccacagtgaggtcagttttacatgtcgctactataatgaattgggaagttaaacagatggatgttaaaaatgcattcttgcatggagatttgacagagactgtgtatatgaaacaacctaccggctttgtagactctgcaagaccagattatgtgtgccatctccataaatttctctatggcctctaacaatctccaagggcctggtttgataagttcagcaattttctacttgagtttggtttcttttgcagtcaatctgatccttcgttatttgtctacatcaagaataaggacatcatcttgcttctcttgtatgttgacgacatggccatcacaggaaacagctccacagcattgtctaacctattagatgagctcaacaaacaattcagaatgaaggatatgggaaagctacactattttcttggcatacaagcgcactatcattcagggggactgttcttgtcacaacagaagtatgttgaagacttacttattactgcttcaatggctgattgtgcaccaatgccgactccactccctctgcagctaaacaaggtacctaaccaagatgaacagttctcagatcagagatattttagaagtttggctggtaagcttcaatacctaacccttacccgtccagatatccaatttgcagtcaattatgtgtgtcagaagatgcactccccaacagtctccgattatcacctgttgaagcgaattctcagatatgttaaaggaactacaacaatggggatttcatttgcaaaagatacggattgcacagttagagcttacagtgatagtgattatggtggctgcaagagaacaagacgctcgacagcagggttttgtaccttctttggctcaaatataatctcttggtgctcccagaagcaagaaacagtggcaagaagctccactgaggccgagtatagagctctgtcagatgcagctgccgagatcacttggttgtgtaaagtcctaaaggaacttcagatccctcttcatacagctccagaactatacgctgataatctctcctccatctacttgactgctaatccgtctttccacaagagaagcaaacacttcgagactcactatcactacgtcagggagagagtggctcttgggtctttgactgtgaaacatatcccgagtcacgtccagctggccgatatatttactaaatccttaccagtcggagccttcacaactctacgcttcaaactcgacgtttgttgtccacccactccgagtttgagggggac

>ATCOPIA61_LTR

taatagagtgtggtttagagataaccatacatttgtttcagtaatgtaaaccggtataagataaaccgggttttgccacttactataaaagaaacactctgtactattattacatttaatgagaaatacagagaaattttacctttcttctttctctcgtctctaacaaaattgtttctatta

>ATCOPIA61_IN

tggtatcagagcaatgaattttcaaaattcattcgattcttcttcgttttttgctttgttctttgatgattctcattgatcttcttcttcttcgatagattcgtttggtttacgtgccggaatcacaaatatcgatcaagttttcttcagattctgttaacaatggttgtaactcgcaaaatccctcgtcgatcatctcgtgtagcttcgtcagctcgaagacaagtgaatcgtgatgatgatgatgagagttcgccagaaattcctccgattgttcctccgattgtttctccgattgttcctccggttgttcctccggttgttcccgtttatacatcatcgattgataatccagatagtattcattctccatatcatctttctaacagtgataatcctggaatttctataatttctgaggttcttgatggtacaaattatgatgattggcatatagcgatgaaaacttctttagatgcaaagaacaagattgcttttattgatggatctatatctcgacctagttaaacaaacgctatgttcaggatttggtcacgatgcaacagtttagtgaaatcttggattctaaactcggtttcaaaacagatctataaaagcattcttcgttttagtgatgcatctgagatttggaaagatttggccacacgttttcacattacaaaccttccaagatcatatcaattgtcacagcagatatggtcactacaacaaggttcaatggatcttgctacgtattatactactctgaagacattgtggaatgagttggatggcgctaattgtgttacagtatgtaaaaactgtgattgttgcaaagctatggaaacaaaatcagaacatgctcgtgttattaaatttttggctggcttaaacgaatcctatgccgtcattagaagtcagataatcatgaagaagcatattccagaacttgctgggatttacaatttgttggatcaggatttgagtcaaagaaatatcactcctgttcagaatgctgtagcttttaatttttcagcaatggaaccaactcaagcttctgttaatgctacttataatcattcaaaactgcagcagaaagtgatttgttctcattgtggatacactggtcacactgtggataagtgttataaaattcatggttatcccataggatttaaacacaagaacaagaatgctcaacaggataaacagattgttcctccaaaacctgttgttgctcagttagcttttactaatgctacttcgagtgatttgttaacaggaatggcaaaaactctaacaaaagatcagattcaaggggttcttgcttatttttaaggaaaactcaatgatggttctgtgacatggcttctccatctggtgctacgattactgcattacctggtatagctttttcttcctccacattacgttttattggagccttgaaagctacaggaaatgttttatcttcatcctcatgggtgatagatagtggagctacacatcatgtgtgtcatgatataacattatttcagacattatctgaaaccatgaatgattctgttaccttacctactggttatggagttaaaataacaggaattggttcagtggagttaagtgatcatatgatcttgaagaatgtgttatattttccagactttcggttaaatttactgagtgtgagtcagttaaccaaagatttgggatatcgtgtaagttttgatgaaagttgttgcatgatacaagatcatatcaaggggttgatgattggtaaaggggaacagatttcaaatctctacgttttggatatgcaatttctcgtgaagaattcaactcaaccacttgttttttctgcaattcttgttgactctagtttatggcatagtagactaggtcattcatctaatgtcaaaacagatcttgttacagatgtacttggtttcaaacaaaggaataaagatgattttcattgtgttatatgtcccttggccaaacagaaacgattatcatttccatccaagaataacatctgtgaatctgcttttgatctcttacacattgatatatggggaccgttttctgtttctacatcataaggatacaaatactttttaaccattgtggatgatcatactagggtcacctggttgtatttgttgaaagcaaaatctgatgtattgtagatattccctgatttcttgaagatggtggagactcaatataagacagtggtgaaagcagtccggtctgacaatgcacctgagcttaagtttgtgaatttgttcaagtcgaaaggtatcatcgcatatcattcatgtccagagactccagaacaaaactcagttgttgaacgcaagcatcaacatattctaaatgttgcaaggtcattgatgtttcagtcacgggttcctgaagagtattggggagattgtgttctcaccgcagtcttccttatcaatagacttcccacaccactactgaagaataagtctccttatgaagttcttacttcaaagaaaccaaaatacaggcttacgtgtctttggctgtctttgttacagttcaacttcgtcgaagaacagacacaaatttcagccaagagccaaagcttgtatctttctgggatatcctagtggttacaaaggatacaagttattggatttggaaaccaagtccattcatatttcaaggaatgttgttttccatgaaaccagatttcccttcagttcaggtgattctgctggtcctttctctgatatttttggcagcataaatgaatctcttattgagaatgatacagaaattgttgacagtaacattcctgtagttgtgagtgaggctcctattttgagtgaacctcttacagttgtgaatgattctgtcaatgattcacatgaggattcagctccgaaatcagttcctacaacttcaacctctcgatctaaaagagagagtaaacaaccagctcatttgaaggattacttttgtaatctatcgagaaaaggagttcaatatccactttctgactatatgtcttatgatcaactttcaactccatatcgagcttatatctgttcagttacaaagttttcagaaccttcttccttttttcaagctaagaagtctgatgattggattaaggctatgaatgcagagttgcaggctctagaaggcactgctacatgggagatttgttctttgccttcaaataaaaaggccattggttgcaaatgggtttacaaggttaaactcaatgttgatggaaccttagaacgttataaggctcgtttagtagcaaaaggctacacgcaacaagaaggagttgattttgaagataccttctctccagtggcaaaaatgacaacagttaagaccttacttgcagttgcagctgctaagaagtggagtcttcatcagctggatatatctaatgcgtttcttaatagggatttgtatgaagagatttacatgaacctagcgccaggatatacaccaaaagaaggagaggaaatacctcctaatgcagtttgcaagctaaagaagtctctttatggactaaaacaagattcgagacaatggtttttgaagtttcgttctactttgctgtctctaggctttcaacagtctcatgctgatcacactctatttgtgaaaaaacaacaatggcaggtacattgctgttcttgtctatgtagatgacatagtaatagctagcaacaatgatgaagaggttgcaaatctaaaagatgatttgaagaaagcttttaaacttagagatttgggatctttgcaatatttcttcggattagaagttgcaagatctgcctcgggtatatctgtttgtcagaggaaatatgccctggatatcttagaagaaactggtatgttggcttgtaagccatctgctattccgatggaaccgagtatcaagttagttggtgatggagctgaacctgtcattaatgattcggcttcttatcgaagattggtagggaaactgatgtatttgactataactcgtccagaaatcacttacgctatgaacaagttatgtcaattcacttcagcaccaaaagggtcacatatgaaagctattcttaaagtcctgcaatatataaaaggcacaattggaaatggcttgttctattctgctacttctgactttgttcttaaaggatttacggatgcagattgggcttcatgtcgtgattcaagaagatctacttctggctattgcatgtttttaggtgaatcaatgatctcttggaaatctaagaagcagcaaatggcgtctcattcttctgcagaatctgagtaccgtgctatgcaatatgctgtcagagagattgtgtgttagttcatctcttatctgatcttcaagtacctcaacatgctcctgtggctttcttctgtgattctacaacggcaatacatattgcgaacaattctgtatttcacgaacggaccaagcatgtcgagctggattgtcacatcgttagagacagaattatgagtggtccgattaaaattctacatgtgactactatcttcagctggcagatgtgtttaccaagtctctgtacccgacacagttcaagtctattgttggcaagatgtcattgaagtacatgccatcttgagggggca

>ATCOPIA62_LTR

tattagagtatggtttagaataaaccatgattgagtctgtaaaccggattgaaccgggtattaatgttccttatataaacgagtctgtaatcaagtccagatttattaaggaataatatgagattcttatcttcttctccgtaacaaactcttctgtaaca

>ATCOPIA62_IN

tggtatcagagcatgaatggtgaatcattcatacgattcttgacctcgttttcttcttttctttttcatttcgatcagtttcttcagtctcgtcactgagaaactgttgtatcgaccaaaaatttcaacaaaaatttcttcctttgccgtgaaattggagcttctctaatggcgcctggacgtaaaatctctactcgtcgcacgattcgcgttcctgtttcagctcgtagatcggcaaatcgcgatgattcttcacctgaaggttcacctgaacctcgagctcgtactcgaaatccggtaactgaatctcatgatagtatacattcaccctattatcttacgaatagtgataatcctggagcttctattacttctgaagtgtttgatggaacgaattatgatgattggaaaatttcgatcaagattgctttagatgcgaagaataagcttgttttcattgatggatctgttcctcgacctcctgaatcagatcctatgtttcgaatttggtcccgatgtaacagcttggttaagtcttggctcttgaattcggtgtcaaaaccaatatataagagtatccttcgtttcgatgatgcctcagagatatggaatgatctttcaactcgttatcacattactaatcttccaagatcttatccgttaactcaacagatttggtcacttcaacaagggactatggatcttactacttactatacgacattgaggactctctggaatgaattggatggttctgattgtgtgactttgtgtaaacgttgtgattgttgcaaagctatggataagaaagctgaacatgctcgtgtgataaagtttttggctggcttgaatgaatcatatgccgtcattagaagccaaatcatcatgaagaaacatgtgccttccttagctgagatttacaatttgttggatcaagatcacagtcaacgcagcttcacaccggttccttctaatgcagctgcatttattgtatctgcgccagaacaagttcaaccttctgtgaatgccacattcaacaatgcgaaaccacagaaagtcatatgttctcattgtggttacacaggacatactgttgatcgttgttacaagattcatggatatccacttggttttaaacacaagaataagaaccaatctgataagagtgtttctttggaaaaatcagtttctacagttaaacctgttgttgctcatatggctttgacagatagtactacaaatgatcttattaatggtctgactaaggttcttaccaaggatcaaattaatggagttgttgcatacttcaattctcaaatgcagaattcctctattgcttcctcgtctggtgctactattaccgcattacctggtattgctttctcctcctctactcttggttttattggtgttttgaaagctactgttaatgttttatcctcggaaacttggataatagacagtggagcaactcatcatgtttgtcatgataagaatttgcttatgagattatctgaaactatgaatagttcagttaccttacctactggttttggagttaagatcacatgtataggtacagtgaagctgaatgagttcctcgtcttgaataatgtgctttacattccggattttcgccttaatcttctgagtgtcagtcagctgactaaagatctgggatatagagtgacatttgatgaggattattgccttatacaggatcatgtcaaggggctgatgattggtagaggtgagcagatcaacaatctatacgtcctggatgttccgagaattaaggattttcctactaaggaaataagttttcatgcaaacattgttgttgattctagtctttggcatagtagactaggtcatccatctgtaactacttctgatatagttactgatgtacttggatttaaacaaaggaatgaaagatcttttcattgcaccatttgtcctcttgcaaaacagaagcgtcttccctttgtttccaagaatcatgtttgcgactcagcttttgatttagttcatatcgacgtctggggtccattcaatgttcctactccagatggttttcgatattttctaaccattgttgatgatcatacacgggtcacttggttgtatcttatgaagaacaagaatgaagtgttgactatcttcccagattttctgaaaatgatagagactcagtacaagagtcaggtgaaaggtgttagatcagacaatgcaccagaattgaagtttgtgaagttgtttaaagaaaagggcatcattcattatttctcttgtccagaaacaccagaacaaaactcggtggtggaaaggaaacatcaacacatattgaatgttgctcgttctcttatgtttcaagctcaagtgcctgtggaatattggggagagtgtgtgttaactgcagtctttctcatcaatcgattgcctacaccattgcttcatgacaaatctccttttgaagtgcttactaacaaaatgcctgattttaatggtcttcgtgtgtttggctgtctttgttacagttctacatcaaccaaaaatcgagataagtttcaaccaagagctaaggcgtgtgtgtttcttggttatccaccaggtgttaagggttatcgacttttggatttggaaaccaatatcatatacgtctcacgcaatgttgtttttcatgaagacatttttccatttgctaaaagtggatctactgttcttcctgattattttgctactgaaacatctaatgttgatgcatcttctactgaagcatctacttctgaagcacctgcagttgtgaatgattctgtcactccatctaatatcaatcctgtagttgtgagtgaatctcctacggatactaatgatattgttgacagtactattcctgcagtttcttctacggataagacaagtaaaggccgaacaagtaagactcctgcttacctccaagactattattgtaatttgtctactaatggagtggagcacccaatttcaaatttcttgaactatgatggtttagctgattcacaccgagcatatatttgttctataacaaaatatgcagagcctacttctttcactcaagccaggaaatctgatgattggttaaaggcaatgaatgatgaattgaaggctctagaaggaacagcaacttggaagatatgttctttaccacctgataaacatgccataggctgcagatgggtttataaagtgaagttaaacgcagatggaagtttagagcgttacaaggcacggttagttgccaagggttacacacagcaggagggtgttgactttgttgacactttttccccaatggcaaagatgactattgtcaaaacattgttggttgttgcagcagcaaagaaatggagtttgcatcagttggatatatcgaatgcctttttatatggcgaccttgaagaagaaatttatatgactcttcctccgggttacacaactaaagaaggcgagactcttccacctaatgcagtctgtaagttgcaaaaatctctctatggtttaaaacaagcttcaagacagtggtttctgaagtttagtaccactttaatgctattaggattccaaagatcacaggctgatcacactttgtttgtgagaaatgtgaatgggaaatatatagcagtacttgtgtatgttgatgatattatcatcgcaagtaatgatgatgcagaggttgttgaactaaaagcagacttggaaagagcttttaaactgagagatttgggtactttgaagtattttttgggcttggagatagctcgtaatgcttcaggtatttcagtttgtcaacgtaagtatgcgttgggattacttgaagaaacaggtttattggcttgtaagccatctaatattcctatggaaccaagtataaagttgatatcggatggagatgagcctccgatggaagatccagcttcttacagacgcttagtgggtaaaatgatgtatcttaccatcactagacctgacattacatatgcagtgaatagactttgtcagtttacttcagctccaaaagaatcacatatgaaggcagctcacaaggttttacactatgttaaagggactgttggaacaggtctcttctattcagctgattgtgatatgacattacaggcatatactgatgcagattgggcttcatgtcgtgatacaagacgttccacttctggcttctgtatgtttctaggcacatctttgatctcatggaagtcaaagaagcagcagactgcatctcattcttcagctgagtctgagtatcgagcaatggaatttgcagttcgtgaggttgcttggcttgttaatcttctcagagagtttcaagcacctcagctaaagtccgttgctttcttctgtgattcaactgcagcaatacatattgcaaataatgcagtatttcacgaaagaaccaaacatgtggaacttgattgccacatccttagagacaaggttatgagtggtttgattaagactttgcaccttaaaactgatcaacaggttgcagatgtttttaccaagcccttatttccgactcaattcaaggctcttgttggcaagatggctctccaatgaatatacttgccatcttgagggaggc

>ATCOPIA63_LTR

tgttggacgtgatcaagtcttcatcttattttattatcttagaggagtagtatattatatagttaagtaactgatttataaactattcgtatcacaccgaaaagatacgtatagaattgttaactacaaaacagttacgtgtgtgtgtatgtgtctttacattgggttgtcttctagagacgtgtccctcagttaaagtgtgtcatgtaaacctataaatactgtttatgtatctaataaaagtgtgtggtttgtgtttacaataattagtgcaatacgttgagtgtcgaaagagaaacagagagagtaagacaaaacagagtaaaatctgtgttgtccgatccttaataactttgagagatcaaagttctaca

>ATCOPIA63_IN

tggtatcagagctatggagacaacaatgcagcaagttattccaatcttcaatggtgaatcgtatggcttttggaaaatcaagatgataaccatcttgaaaacacggaagctgtgggatgttatcgaaaatggggttacgtcgaattcgtcgcctgaaacctcaccggcgttaactagagagcgtgacgatcaagtgatgaaggatatgatggctcttcagattcttcaaagtgcagtttctgattcaatatttccgagaatcgctccggcatcaagcgcaacagaagcgtggaacgctttggaaatggagttccaaggaagttcacaagttaagatgatcaatcttcagaccttaagaagagaatatgagaatctaaagatggaggaaggtgaaaccataaatgattttacaaccaagttgattaatctgagtaatcaactaagagttcatggagaagagaagtccgattaccaagtggttcaaaagattctaatctccgtaccacaacaattcgacagtatcgttggggtacttgaacaaacgaaagacctatcaactctttctgtcacagaattgataggaacactgaaagcgcatgagaggcgtttaaatctccgagaagatcgtatcaatgagggtgcgtttaatggagaaaagcttggctcaagaggagaaaacaagcaaaacaaaattcgccatgggaagaccaacatgtggtgtggcgtgtgtaaaaggaacaatcacaatgaggtagattgtttcagaaagaagagtgaaagcatttcacaaagaggtggaagctatgaaagaagatgctatgtgtgcgacaagcaaggacacatagccagagattgcaaacttagaaaaggtgaaagagcacatctaagtattgaagaatcagaagatgaaaaagaagatgagtgtcatatgctctttagtgctgttgaagaaaaagagatctcaacaattggagaagaaacgtggttggtagatagcggatgcaccaatcacatgtctaaagatgttagacacttcatcgctttggatcgcagcaagaaaattatcatacggattggtaatggtggaaaggttgtgtctgaaggcaaaggagatataagagtatccacaaacaaaggagatcatgtcatcaaagatgtactttatgtacccgaactagcaagaaatttgcttagtgtctctcaaatgatatccaacgggtatcgagtcatctttgaagacaacaagtgtgtgattcaagatttaaaaggaaggaagatcttagacatcaagatgaaggacagaagttttccaattatttggaaaaaatcaagagaagaaacgtatatggctttcgaagaaaaagaagaacaaacagatctttggcacaaacgatttggtcatgtgaattatgacaagatagagacaatgcaaacgctgaagattgtggaaaagcttccaaagtttgaagtaataaaaggcatatgtgcggcttgtgagatgggaaaacaatcaaggaggagttttccaaagaaatctcaaagcaacacaaacaaaaccttggagcttattcattcggacgtgtgtggtccaatgcaaacggagtcaataaatgggagcaggtacttcctcacatttattgatgatttctcaaggatgacatgggtgtatttcttgaaaaacaagagtgaagtgatcacaaagttcaagatattcaagccttatgtggagaaccaatcggagagtcgaatcaaaaggcttagaacagatggaggtggagaatttctatcaagagaattcatcaaactatgtcaagagagtggcatacatcatgagataacaactccttactctccacaacaaaatggtgttgcggaaaggagaaacagaacactcgtagagatggcaagatcaatgatcgaagagaagaagttatctaacaagttttgggctgaagctattgcaacatcaacatacttgcaaaatagattgccatctaagtcactggaaaaaggagttacaccaatggagatatggagtggaaagaaaccaagtgttgatcacttgaaagtttttggatgtgtatgttacatacacataccggatgagaagagaagaaagctcgacactaaagccaagcaagggatattcgtaggctacagcaatgaatcaaaagggtatagagtgttcttgctaaatgaagaaaagatcgaagtgtcaaaagatgtgacattcgatgagaagaaaacatggagccatgatgaaaaaggagaaagaaaagcgattctatcgctagtcaagatcaattcacaagaacaaggaggagggaatgatctaaacgcccatatagatcaagtaagtaacgcattcaatcaacttcatatttcgagtagaggagtgcaaaactcacatgaagaaggagaagaatctgttggtccaagaggtttcagatcaatcaataatcttatggaccaaacaaatgaagttgaaggagaggctcttatacatgagatgtgtctcatgatggctgaagaaccacaagctttggaggaagcaatgaaagatgaaaagtggatagaagccatgagagaagagttgcgaatgatagagaagaacaaaacatgggaagttgtggcgagaccaaaagacaaaaatgtgatcagtgtcaaatggatctttcggttgaaaactgatgcaagtggagaagcaatcaaacgcaaagcaagattggtggccagaggattcacacaagaatatggtgtggattatcttgaaacttttgcaccggtttcaagatatgatacaataagaaccataatggcgattgcggctcaacaaggatggaaactcttccaaatggatgtgaagtcagcgttcttaaatggagacttggaagaagaagtttacatagaacaaccaccaggtttcatagaagaaaaagaagaaggaaaagttcttaaacttcacaaagcgctatacgggttaaaacaagcacctagagcatggtatggacgcattgatggatactttatcaagaatggttttgagagaagcatcaatgatgcagcattttatgtgaagaagacttcaaaggagatattggtggttagtctctatgttgatgacatcatagtcacaggaagcaacgtaaaagagattgaacgattcaaggaggagatgaagaatgagtttgaaatgacagacttaggagaactaagttactttcttggcatggaagtaaaccaagacgatgaaggcattttcttatcacaagaaaactacgcaaagaagcttttgaagaaatttggaatgcaagagtgcaagagtgtgtcaacgccattgacgccccacggtaaaatagaggaggtcttaagtgaaaagcttgaagacgttacgatgtatagaagcatgattggaggtatgttgtatctttgtgcttctagaccagacatcatgtatgcgagttcttacttgtctcgctacatgagatctccattaaagcaacatcttcaagaagccaaaagggttcttagatatgttaaaggtactttgacatatgggattcatttcaagcgagtagaaaaaccagaattggtgggattttctgatagcgattgggccggatcagttgaggacaagaagagtacaagtggctacgtctttacgatcggatcaggtgcattttgttggaattccagcaagcagaagacggttgcccaatcaacggccgaggcggagtacattgccgtttgctcagcagcaaaccaagcaatatggttacaaaggttggtgaatgagatcggtttcaaggcagagaaaggtataaggatcttctgtgataacaaatcagcgattgcaatcggaaagaatccagttcaacatcgtcggaccaagcatatcgacatcaaatatcatttcgttcgagaggctcaacagaatgggaagatcaaacttgagtactgtccaggagagttacaaatagctgacatattgacaaagcctttaaacacaacaaggttcgaggtcttacgagaagagctaggcgtaactatgaagccatgatcaaggaggag

>ATCOPIA64_LTR

tgttgggataagcatgaagtcaataaattagggaataaaagttatgttttaaaataggattaagataaatctttaggagtaatctaaagatatgttaggagttatctacatcttattctattgtgtttgttatcatccctctcctatataagagttgccaaagttgtggtaaaccttaagagatttgagagagagattgaaagctttgttttgagttattttcaaagctaataaaaggagtgcttttatatttgatctaagtattcaaatcttta

>ATCOPIA64_IN

tttggtatcagagcataacctaggtttcaatttttctttttcgaatatgagtgaaattgttgctgcaagcaacaaacctaaagaagggggggggggggtcatcatccattcattgtcccatgctgaacaacgctaattacacggtgtggactatgaggatggaggctactctccgagtgcataaagtttgggaaacaatcgatcctggatcagatgacatggagaagaatgatatggctcgagctcttttatttcaatccgtacccgaatcaactatcttacaagtcggaaaacacaagacctctaaagccatgtgggaagccatcaagacaaggaacctcggtgctgaaagggtaaaagaagcaaagctacaaactcttatggcagagtttgatagactaaatatgaaagataatgagacaatcgatgagtttgtagggagaatatctgagatttctacgaaatcagaatctcttggagaagaaatagaagagtcaaagattgtgaaaaaatttctgaaaagcttgccacgaaagaagtatatacatatcattgcagcattagagcagattctcgatctcaacacaaccggatttgaagatatagtaggaaggatgaaaacatatgaagatcgggtttgtgatgaggatgattcaccggaggaacagggtaaactcatgtatgcaaattcagaatcatcttatgatacaagaggaggaagaggtagaggtcgaggtcgttcttctggcagaggacgtggaggatatggttatcaacaaagggacaagagtaaagtcatctgttatagatgtgataaaacaggacattatgcatctgaatgtctagatcgtttgctcaaacttattaaagcgcaagaacaacaacaaaacaatgaagatgatgatgaaattgagtcgcttatgatgcatgaggtggtttatttgaatgagagaagcgtgaagccaaaggagtttgaagcttgctcagataatagctggtatttggataatggtgcgagtaaccatatgacaggtaatcttcagtggttctccaagttgaatgagatgatcacaggaaaggtgagattcggtgatgattctcgtattgacataaaagggaaaggttcaattgtgctcatcaccaaaggaggaatacgcaagacattgactgatgtctactttataccggatctaaaaagcaatatcattagtttgggacaagccactgaagcaggctgtgatgttagaatgaaggatgatcaattaacgttgcatgatcgtgaagggtgtctgttactaagagcaacacgctccagaaacagactttacaaagtcgatttaaatgttgaaaacgtgaagtgcttacaactcgaagcagcaagtgagtcaactaaatggcacgcacgtcttggacattaactttgacacaatccaagctatggtaagaaaggaattggttatcggaatctctaatattccaaaggagaaagagacgtgtggttcatgcctccttggaaaacaagcaaggcagccattccctaaagcaactacttatcgagcatcacaagtattggaacttgttcatggtgatctttgtggtccgataacacaatctacaactgctaagaaaagatacatactcgtgctgatcgatgatcacactcgttacatgtggtctatgctattgaaggagaagagtgaggcatttgaaaaattcagagatttcaagactaaagttgaacaagaaagtggagttaagatcaaaaccttcagaacagataaaggaggagagtttgtctctcaagaatttcaagatttctgtgcaaaagaaggcatcaacagacacttaaccgctccatacacaccacagcagaatggagttgttgaaaggagaaatagaacgttgctaggtatgacaagaagcatcttgaagcacatgaaaatgcctaactatttgtggggagaagcagtaaggcactcaacctatatcatcaacagagtaggaacaagatctttgcagaaccaaacaccatatgaagtttttaagcaaagaaagccgaatgtagaacacttacgtgtatttggatgtattggttatgccaagattgaaggtccacatttgagaaagcttgatgataggtcaaagatgcttgtttatcttggaactgaaccaggctccaaagcatatcgtcttcttgatccaactaaccgcaaaattatcgtaagcagagatgtgtactttgatgaaaacatgagctagaagtggaacaattcagattctgaaactcgggacatctctggaacattctcacttaccttaggagaatttggaaacaatggaattcaagaaagtgatgatatagaaacagaaaaaaacggagaagaaagtgagaacagtcacgaagaagaaggagaaaacgaacacaatgaacaagaacagattgatgctgaagaaacacaaccctcacatgctactcctctgccaacattaagaagatcaacaagacaagttggaaaaccaaattatttggacgattatgtcttaatggcagagatcgaaggcgaacaagttctattggcaattaatgatgaaccatgggattttaaagaagcaaacaagttgaaagaatggagagatgcttgcaaagaagagatcctgtcaatagagaagaacaaaacttggagcttaattgatcttccagttagaagaaaagtcatagggttaaaatgggtttttaagatcaagcgaaattcagatggcagcataaacaagtacaaggcaaggttagtagcaaagggttatgttcagaggcatggtattgactacgatgaagtatttgctcatgtagctcgtattgaaacaattcgagtcatcattgccttagcagcatcaaacggatgggaagtacatcacttagacgtcaagaccgcttttctccatggggaactaagagaagatgtttatgttacacaacctgagggcttcacaaataaagacaatgaagggaaagtctacaaattacacaaggctctttatggtttaaaacaagcacctcgtgcttggaacactaagctgaacaaaattcttcaagaactgaattttgtaaagtgttctaaagaaccatcagtgtacagaagacaagaagaaaagaagcttctcattgtggctatttatgttgatgatttacttgtgacaggctctagcttggatctaatactctgtttcaagaaagatatggctggaaaattcgaaatgagtgatctgggacagctcacgtactaccttggcattgaagttctccatagaaagaatggcatcatattaagacaagagaggtatgcaatgaaaattatagaagaagctggaatgagtaactgcaacccggtcttaataccaatggcagcaggattagagctctgcaaagcacaagaagagaagtgcatcaccgaaagagactacagaagaatgattggatgtctgagatacatagtgcatactcggccggatctctcttattgcgttggtgtactaagcaggtatctacaacaaccgagggaatcacatggaaacgcactgaagcaagttctaaggtatctcaaaggaactatgtctcatggtctgtacctcaagagaggattcaagagcggattagtaggctatagtgatagtagtcatagtgcggatttggatgatggaaaaagcactgccggtcatatcttctatcttcatcaatgtcccattacatggtgttcacaaaaacaacaagtagtggctctttcatcttgtgaagcggagttcatggcagctactgaagcagccaagcaagccatatggttacaagacctattcgcagaagtgtgtgggacaacaagtgagaaggtgatgattcgagtggataacaagtcggcgattgctcttactaagaatcttgtctttcatggtaggagtaaacatattcatcgaaggtatcatttcataagagaatgtgttgagaataacttagtcgaagtggatcatgttcccggagttgagcaaagagctgacattctaaccaagccacttggaaggattaagttcagagagatgagagaactagtaggagttcagtgtgtgttggaagatgacttcaagcttaaaggggagtt

>ATCOPIA65A_LTR

tgttggagtaagtttgaaatgacttgagcaataagtcattaaagaataagaaggattatgttaaatctttaggagatatctaaagatatatgttaggagttatctaatgctaatcctagtatgttttggtttattaatctctatatatatgagtcccaagctgtggttgactatatgagtttagagaaattaagactttgagattgtttacgttttgagtaattttcctaagcttaataagaaagtaagcttaataagaaagtaattcttatatctttgagttcttaatcttttttgaaactaga

>ATCOPIA65A_IN

atattaatacttcttgctgaagtcataggtacaatgtgtgagagagtaaagattcgagttgataacaaatctgctatagctctcacaaagaacccggtgtttcacgggagaagcaaacacatccatcgaagatatcatttcatacgggagtgtgttgagaatggtcaagttgaagtagaacatgttctcggagttcgacaaaaggcttacatactaacaaaggcgcttgggaagatcaagttcttggagatgagagatctcattggagtacaagaggtgtcaaaagaagttttcaagcttaaaggggagaa

>ATCOPIA65_LTR

tgttggagtaagcttgaaataacttaataaataagttattaaatattacaaaggattatgttaaatctctaggagttatctagatatttaaattaggagttatctaatgttaatcctagtatgattaggtctaattattctctatatatatgagtcccaaggtgtggttgaccatatgagttttaaagagattaagatttagagattgtttaggttttgagttattttcctaaactataaagagagttattcttaaatctttgagttcttaatcttctttgaaaactaga

>ATCOPIA65_IN

tttggtatcagagcatctaggtttgatattgaaacacaaacatgagtgaaatcgttgaagcaacaagcaaaggtaaagaaggtggaggatcagcgtcgatccaatgtccgatgctaaactccgtcaactatactgtatggaccatgaggatggaggctgtgcttagagtacacaaactttggggaacaattgaacccggatcagccgacgaagagaagaatgatatggctcgggctttgctctttcaatccatacctgagtcgttaattttacaagttggtaaacaaaagacttcttcagctgtctgggaagccataaaatcaagaaatcttggtgcagaacgagtaaaagaggcgagattacagacacttatggcagaatttgataagctgaagatgaaggatagtgagacgattgatgattacgttggtaggatctcagagattactacaaaagctgcagctttaggagaagatatagaagaatccaagatcgttaaaaagtttctcaaaagtttgccaagaaagaaatacatacacattgttgcagccttagaacaagttcttgatctgaaaacaactaccttcgaagacattgcaggaagaatcaagacttatgaagacagagtttgggacgatgatgactcacatgaagaccaaggcaaacttatgtgtgttaaaacataatcacaagatggcaaactcatgtatgcaaattcggattcacaagggcaatacgaatttcaggacagaggtagaggaagaggtcgtggacgatttggaagaggaagaggaagaggttatcaacaaagagataaaagcaaagtcacatgttataggtgtgatagactcgggcactatgcctctgattgtccagaccgtcttctcaagctgatccgactccaagaacagaaagaaaaagaggaagatgacactcatgaagcagaatcacttatgatgcatgaggtggtatatctcaacgagaagaatattcgcccaacagagttagaatcgtgtattaacaatgcttggtatcttgacaatggtgctagtaaccatatgacgggaaatcgtgcttggttctgtaagcttgatgagatgatcacagggaaagtaaggttcggtgatgattcatgcatcaatataaaaggaaagggttcgattccttttattagtaaaggaggtgaaagaaaaatactatttgatgtttactacataccagacttgaagagtaacatcttaagtttaggacaagcaactgaatcagggtgtgacatcagaatgagagaagactacttaaccttgcatgatcgagaaggaaatctactaataaaggcgcagcgatcaaggaacagattatataaagtgagtctagaagttgaaaactccaagtgcctgcagctcacaacaacaaatgaatcaacaatatggcatgccagactaggacacatcagttttgagaccattaaagctatgataaagaaagaacttgttattgggatatctagctcagttccacaagaaaaggaaacatgcggttcttgtttgttcggaaaacaagctagacattcattcccaaaagcaacttcttatcgtgcagcacaagtacttgaactcatccatggtgatctctgtggacctatttcaccatctacagcagctaagaagaggtatgtatttgtattgattgacgatcattcacgatacatgtggtctattctactaaaggagaaaagtgaagcgtttggaaagtttaaagagtttaaggcactagttgagcaagagtgtggggctatcatcaagacattcagaactgatagagggggagagttcttatcacacgaatttcaagagttttgtgcaaaagagggaatcaatagacacttaactgcaccatacacgcctcagcagaatggagttgtggagagaaggaacagaacactcctaggaatgacaagaagtattctcaaacacatgaacatgccgaattatctttggggagaagctgtgagacattcgacttatcttataaacagagttggaacaagatcactttcaaatcaaacaccttatgaagtctttaaacataagaagccgaatgttgaacatttaagagtgtttggttgtgttagctatgctaaagtcgaagttccaaatctgaagaaattggatgataggtctcggatgcttgtttatcttggtacagaacctggttctaaagcgtatcgactacttgatccaacaaaaagaagaatctttgtgagcagagatgtcgtctttgatgaaaacagaagctggatgtggcaagaatcaagctcagaaactgacaaggaatcagggacattcacaattaccttaagcgagtttggaaataatggagtcacagagaatgatatctctacagaaccagaagaaacagaagaagctgagataaatggagaagatgagaatatcattgaagaagcagaaactgaagagcatgatcaatctcaagaagaacctcaacccgtaagaagatcacaaagacaagtaatccgacctaactacttgaaagactacgtgttatgtgcagaaatcgaagcagaacaccttttacttgctgtcaatgatgaaccgtgggacttcaaagaagcaaacaagtcaaaagaatggagagatgcttgtaaagaggaaattcaatcaatagagaagaatcgcacttggagtttggtcgatctccctgttggaagcaaagcaataggagtcaagtgggtttttaaactgaagcataactctgatggcagcataaataaatataaagcaagactagtggcaaaaggatacgttcaacgacatggtgtagactttgaagaagtatttgctccggtggctcgtattgaaacagttcgtctcataattgctttagcagcctcaaatggttgggagatacatcatttggatgttaaaactgcattccttcatggggaattaagagaagatgtctacgtctcacaacctgaaggcttcacaaacaaagaaagcaaagagaaagtctacaaactgcacaaagctctctatggattacgtcaagcaccccgggcttggaacactaagctaaatgaaattctcaaagagttgaagttcgaaaaatgtcacaaagaaccctcattatacagaaaacaagaaggcgagaacattcttgttgtagccgtatatgtggatgaccttcttgtcacaggctctaacttagacatcattctcaactttaaaaagggaatggttggaaagttcgagatgagtgacttaggtaaactcacatattatcttggtatagaggttctacaaagtaaagatgggattacgctgaaacaagaaagatatgcaaagaagatcttagaggaagctggaatgagtaagtgtaacacagtcaatacaccaatgatagctagtttggagctgtctaaagcacaagatgagaagaggattgatgagactgattacagaagaaatattggttgtcttcgttacttactccacacccgtccggatctctcttacaatgttggcatactgagtagatacttgcaggaaccgagagaatcacatggagctgcactcaagcaaatcctaaggtaccttcaaggaacaacttcacatggactttacttcaagaaaggagaaaatgcaggattgatcggctatagtgacagcagtcacaacgtggacttagatgatggtaaaagcaccggaggtcatattttctatcttaatgattgtcccatcacatggtgttcacagaagcaacaagtggttacgctatcttcttgtgaagctgaatttatggcagccactgaggcagctaaacaggctatttggctccaagaacttctggctgaagtcattggtactgagtgtgagaaggtaacaattcgagttgataacaagtctgctatagctctcacaaagaacccggtgttccacgggagaagtaaacacatccaccggagataccatttcattcgggagtgcgttgaaaatggacaaattgaagtagaacacgttcccggagtaagacaaaaagctgatatactaacaaaggctcttgggaagattaagttcttagagatgagagagcttattggagtacaaggagtgtcaaaagaagatttcaagcttaaaagggagat

>ATCOPIA66_LTR

tgtaaaggtgtgattcaaacttctggtttagcctgatgtcgacttggtttaatcagttagttgttggatcacggttcaatgccggtttggtattggttgaaggagaaaggagaaatcggtttgagttcagtagaagattgaagagactctcttggtattagagggtttgacttggactctgttataaatagtcttcttcttcctttgttcgttgtaacagaaaattcttatctcaattcttctcctttgtaacacgatcttgagctctgggttttgagcttgagtaaatattgattcatagtggattgctggattgaatccagccccagacgtaggatcatcacaccggtgatctgaactgggtaaacaatcgtgtgttcgtttcgttctttgtttgattatgttcttgatcgatctattaagtgttccgcgaattgaagcttgatcgagttgtgttgtgattgaatcgagttgtgttgaatcgatctttaagttatcggattgtaaca

>ATCOPIA66_IN

gattggtatcagagcctaaggttctttaatggcgatgacgaacgaagcaagcttgaattcagggttaaaggcttcgtttcaagtctttaacgagaactcagacttctcgctatggaagacaaggatgaaagcacatcttggcttagctgggcttaaaggagtgatcgatgatttcacattgacgaaatttgttccgctaactaaaagtgaaggaaagaaggttgaggaaggtgatgatgatggatcagagtcatcacagactaaggaagtacctaatcttgtaaagatggagaaatcagaacaagcaatgaatgtgattatcgctcacgttggtgatgttgttctgaggaaaattgatcactgcaagtctgctgcagagatgtgggaaaccttgaacaagctgtatatggaaacatcgttaccaaatcgaatctatgtgcagcttaagttttactcattcaagatgaatgacacaatgtcaattaatgagaatgtgaatgaattcttgaagataattgccgagttaagcagtcttgagattgtggtaggtgaagaagttcgtgcaatattgttcttgaatggattgtcatcaagatactctcagttaaagcataccctgaagtatgggaacaaagctttgtctctgcaggatgtaatctcctctgttaagtcgttggagagagagctaaatgaatctctagatcttgaaagaagctcctcaacggtcttgtatactactgaaagaggtagaccactagtgagaaacaatgagaacaatcaaaacaatcagaagggtggtcaaggcagaggtagaagcaggtcaaactctaaaacaagagtaacctgctggttttgcaagaaagaaggtcatgttaagaaagactgttttgctagaaagaagaaaatggaaacagaaggtcctggtgaagctggtgtcatcatagagaaacttgttttctctgaagcactaagtgtgaatgatcagatggttaaagatctatgggttttggactcggggtgtacttcacacatgacttatagaagagactggttctgtgattttcaggaaaatggatctacaaccatactactcggagatgatcactcagttgaatcgcaaggccaaggttccataagagttaacacacatggaggatctataaagattctaaacaatgtcaagtatgtgccaaacctgagaaggaatctcatctccacaggaacacttgacaagttagggtatcaacatgaaggtggagcaggaaaagtgagatactttaagaatcaggttactgctttgtgtggaagcttagtcaacggtctgtatattcttgatggtgagacggtgatgactgaaagttgtgcagctgtggactcacagagtaaaacagcattgtggcatagcaggttagaccacatgagtttaaataacctgaaagttcttgctggtaagggtctattgaacggtaaagaaatcaaagattttggatttctgcgaacattgtgtaatgggaatgtccaagagactgagtttcaatgtgggaaaacatgatgttgtggaagcactgagctatgttcatgcagacctctggggatcatcaaatttatcaccctccttatcaggtaaacaatactttctctctatcatagatgataagactagaaaagtatggttatatttccttaggactaaggatgaaacttttgataagttttgtgaatggaaagaacttgtagagaatcgggtggatagaaaggttaagtgtttgagaacaaataatgggttgtaattttgcaatactaagtttgacaggtactgcaagacccatggtattgaaagacataggacgtgtgtgtacacaccacagcaaaatggtgttgcagagagaatgaacaggacgatcatggagaaagtgagatgtttactgaatgagtcaggtctggatgagagtttctaggctgaagcagctgcaactgctgcatacatcattaacagatttcctgcctcagccattgatcataatgttcctgaagaactatggctaaacagaaagccgggatacaaacacttgaagagatttggatcaatcgcatatgttcatcatgatcaaggaaagctgaagccaagagcattgaaaggagtgttcttaggttaccctgctggcacaaaaggttacaagatatggctgcttgatgaaagaaaatgcgtgataagtagaaatgtgatatttcgagaagatatggtctacaaagacctgaacaaagatgtgaatgatgcagtagcagaagacgctgaagcatcaacatcaaattctgatgttatttcagaattggtcaagaagcgagtcagttctaagcaaggtggagtaattactgagctggtagaagtctgtgaaagtgaatctgaagaagattctgaagaacctgcagaaactgcagttactcagtcacctgaaccaagtgggttgacaaattatcaacttgcaagagacagaactcgaagacagatcgtagctccggttaagatgaaagactattctcaatttgcatttgcgttaatgacatatgagatactgaatgtggaagaagaaccacaatgtcttcatgatgctcaaaaggatgaaaactgggagctatggaatggagctatcggtgaagaaatggattctttaacaaagaatggtacttgggaacttgttgacagaccaaaggacagaaaggttatcagttgcaggtggttattcaaaatcaaagttggtataccgggtgttgaatccaagagatacaaggcaagacttgttgcaagaggattctcacagaaaaaaggaattgactaccaggaaatatttgcccttgtggttaaacacacgtctatcagaggcttaatgtttgtggtggttaatcttgacttagagcttgaacatatggatgttaaaacagccttcttacatggtgaattggaagaagagttgtatatggagcagccagagggtgtggtgtcagttggaaacgaagacaaagtttttcttctcaagaagtccttgtacggtttgaagcaggcgccaaggcaatggaacaaaaggtttaacaagtttatgacagatgagaaatttcagagaagtgatcatgatcagtgtgtatatgtgaagacaatgaacaatggagaacttgtctatcttctgttatacgttgatgacatgttgattgcagctaagaatatgtcagaggttaacaaggttaagaagagactcagtagtgagtttgagatgaaagacatgggacctgcaaataagattcttggtatttaaatcacaagagatagagtgaatggagttttgtgtttatctcaagcaggatacttgaaaaaggtgctaaagagattcaatatgagtaattgcaagtcagctctaactcctattggtacacattttaaacttgcatctgtgcaggacgattcagagtgcatagatacagttaaaactccatactcaagtgctgttggaagtgtaatgttcgcgatgatcagcactagaccagacttagcctatgcaataggactagttagtcgtttcatgagtaaaccaagatcagttcattgggaagcagtcaagtggctgctaaggtacataaaaggatcacaggatctgagtttggtgtatactaaagggaaagatctcagtgtcattggttactgtgattcagatcatggtggagacttggataggaaaaggtctactagtggatacgttttcacagtaggaggtaatacaatcagttggaagtcttgtctacaatccgtagtggctttgtcatcaactgaagcagagtttatagctttgactgaagcagtaaaagaagctatatgggttaaagggttgcttgaagatctcggttttcagcaggataaagcacaggtctggagtgattctcagtcagcgatctgtttatcgaggaatagtgtgttccatgagcgaaccaaacacatggcacgcaagaggtcatttctgagtgagattattgaagaaggaaacattgaagttgtgaagattcacacttctattaatcctgctgatatgttgaccaagtacattcccgtgaaaagttttgattcagctttagatactctgaagctgatcgaatggaagtaagctcttaagcttgcattgctggaggttaagtccaagcatggcagtctactgtgttgaagaagatttgtatcaagatggagaat

>ATCOPIA67_LTR

tgtcaaggatactaattgatcataaaaatattttgattgacacacttatagaaactgtgtaaatgacgatagtctagaatcttcttctgtaattcctttataaaggattgtacattctctcgtgaataataagaaaacataacttccttaca

>ATCOPIA67_IN

tggtatcaagagcaaaaagataccctagaaacctctaaattctttttatttctcgcagccgcctctctcctctataatctcttttctttttcttctcctctgttttctatctcctgtttctttctctcacacaatggctgctattgatgcaatcgacactactcaggctctcctcaatgtgaacatgtccaatgtcacaaaacttaccgctggcaactacctgatgtggagccttcaggtccatgcacttctagatggctatgatctcgccggtcatcttgatggctcactgcctgcaccaccacccaccattgaaatcattggtgtcgttacaccaaattcagctttcaccaaatggaaacgccaggataagcttatccactcgtggcctcatcggtgcaatctctcttccggtccagtcaaccttctctagagcactcaccactaatgaaatctggaaaacacatgccaacatctatgccaagccgagctggggtcacatccaacaactgcgcatccaattaaagcagcagaccaaagatataaacacagtcgatgaatatatgcaaggtctcatcactaagtttgatcaactagcacttctcggcaaacctctacaacatgaaaagcaagttgaacacattctcctaggattgcgtgaagactacaaaagcgtcatagagcagattgaaggacgtgactcccctccctccattccagagattcatgagaagttgctcaacaaggaaaacaagctgctgtccacaactacgtccttctccccggtgattcccgtttctgctgacatcgctaacactcgtcctgcaaacagaataaccggtccgttcaaccatggcaatcacaaaccaacaatcagcagtttcagaggaacaccaataacaacaactctcgattcagcaaaggctatcaaggaaaatgtcaactgtgtggagtacagggacatagtgcaaaacgttgtctacaacttcaacaacatcaacaaaacaattcacggaatgtgtcttatccgccttggcagcccaatgccaacctagcgcttgcatccccgcacccagcaaatgcttggcttcttgatagttgcgcaacccatcacctcaccagtgatctcaacaacctcgctatccatcaaccttaccctggcgatgactcagtcctaattggtgatggctcgggtctacaaataacacatactggtttactttccttaccttcatcctctcgtaaccttaccttacagaatgttttatatgttccccacattgctacaaacctaatctctgtttatcgcaatgtgcaattctttctcacgctgggatgccaaagacgttctggagctatgcatttaccactgcaacatacttgatcaatcgccttccgtctccgacaatagccatggaaactccatatcagaagctctttggcagttcaccaaactacggaaagcttcgcacattcagatgtctttattttccatggttacggccatacaagctagaagagagatccactccgtgtgtcttcatcggttatgcaccaactcaaagcgcatatttgtgtctccaaacatccacaggcaggatttatgtttctcgacatgtgaaatttgatgagcaagtgttccccttcactcactcaactcctccatccgataaatcacttccaacaagctcaaattccccattcattcccgtctcccagattccggtcaccgtgccactcgctccgatgcctctgggacccacgagcttcgatcttcaccaatcaccacgttcgccggcaacttctacagagactgcgttagtagatacatcctcctcagccgaaatccaaccttcatcctcaagcccaaatctggcccatactgaaaacccagaaactcaaaactcaaattcaacccaagcccaattactaccaccacaaataaccaacaatccaaaacccaacccacgacacaaaccaatacttcaacgtcctcctcgcagccaaacgttccacctcgctcagtcgacaacgctcaccctatgaaaactcgtcgcaaaaaccatattaccaaaccaaacactaaatacaacctttctgttgccctctccacccacattaaaccagaaccacgaaatgtgaaccaggcgttaaatgatccgcattggcgtggagctttgtcagatgaaattgatgcttttgctaggaatcaaacctttgatttggtccctcggcaacctcagatgaatgttattggctgtaagtggatttttaaaaacaagtttcactctaatggctctcttaacaggtgcaaagcacgtcttgtagcaaaggggtacaatcaacaatttggtcgggattacacagatactttcagccctgtcatcaaagctacaacactccgcttagtccttgatgttgctgtcactcgctcttggccacttcaacaattggacgtcaataacgctttcttacaagggacattgaatgaagaagtttacatggagcagccaccgggatttgtggatgctgacaatccatcacacgtctgccgtctccgcaaagccatctatggactgaaacaagcccctcgagcttggtacatggagctgaaaaactatctcctcagtcttggatttcggaactcactcgctgatacatccttgtttgttctccaatgtggtacgcaatttgtttatctcctagtctacgttaatgacattattatcaccggtaacagtgacaatggcatcaaacgagtcttacaattgttggcagatagattctctgtcaaagatcccgaagaactacattattttctaggccttgaagaacaccgcacaccacaaggacttcatctatcccaacgaaaatacattttggatcttcttcatcgccacgacatgatcaatgcaaaaccggtttccacacccatggcgtcctcaccaaaactgaccaccaactcaggtacaacactctccgatccgactacatatcgacagcttgttggaagcttacaatacctagcatttacaagactagatatctcctatgcggtgaatcgtttgcctcaatttatgcatagaccaacagaagatcactcccaagcagcaaaaagagtactccggtatctcgcaggaaccacaacacacggtattttctttgctgctaagaatactctcactctccatacattctctgatgcggactgtgccggcgatacagaggactacgtctctgcgaatgcatatatagtatatctcggtagccacccaatctcatggtcggctaagaaacagagaggagtaggtcgatcttctacagaagcggagtatagagctgttgccaatgcatcgtcggaaatcagttggatatgcactcttctaagtgagctaggcattacgctatctgcacctccagtgatatattgcgataatgttggagcaacgtttctttgtgccaaccccgtctttcattctagaatgaaacacattgccattgattatcactttatccgtggacaaattcaaaccggtgctctccgagtccctcacgtcaataaaaaggatcaactggcggatgctttaacaaaaccactacaacgagcaaatttcttgaattacgagacaagattggtgttgctaaagcacctccatcttgagggtgcg

>ATCOPIA68_LTR

tgtgagtaagtgacccctatgctattaatatcctaagtataggaaagtctataaagaagaaagagggtcttcggttttgggttagtcaaagcttagcacgtgagacccacacgtgagtacgaataaaactgattctcagctttataacccgagctctgttttcttctgggattgattctattgtgatagaaagtagagagagagagagagaagagagagattattgtaaccgagtaacaatctttgtggcttagtggattccggagaatgcctccggcgagacgtagcgttacgatttggagcctgaactcgtaaaattgtctgtcttcttttctttcttgtaatcaaacgaatgacgagagtgatcggtgagctaaagtgagttgcgaatcaaatccgattcgtaca

>ATCOPIA68_IN

aattggtataagagctccaggttctcgtgttgactgaacgttgttacaaggatgacgaactcgaagatcaagatatcggtgttcgatggaactggtgacttctctctctggaagacacgcatgttctctaatcttcgaatcctaggcttgaaggatactctggtggaacaagcttcgttcccaccactgtcagaagaagatgaagcagatccgacgaagaagaagaagcggatcgaagaagaaactgagaagattgaacgatgtgagaaagctatgaacattatctttcttaatgtcggtgataaggtattgaggaagatcgatcagtgcaagactgcagccaaagcgtggatgttgctggaaagattatatctggtaaagactttaccaaatcatgtgtatttgcaactcaaggtatacaactataggatgcaagaatcgaagtcacttgatgagaacattgatgagttcttaaagatgatctcagatctaagtaacttgcaaatccaagttcctgatgaagtacaagcgatcttgattctgagttctctcccagtcaagtatgaaatgttaaaggagaccttgaaatatggaagagaaggcttaaaacttgaagaggtgataagcgcatctaagtaaaaaaaattagagttccaggcagactcaagctcaaggtctgcaacacaaggtcttcatgttaaaggaaatcagaataacaattcaagatcaagatctcaagacggcaagaagatatgttggatatatggcaaagaggatcatttcaaaaggcaatgctataagtggctagagaagaacaagtcaagtgggaacacacaagaagcaggtgcgtctaggctggcaaaggatgatgctcaagactaggttggtttagtagcttctgaggtaaatctgagtgaggatcgacttgaccagaatgaatggattatggatactggttgttcatttcatatgacaccaagaagagatatattcattgagattgaggagttgacttccgggaaagttagaatggcaaataactcattttcagaggtaaaaggtatcagtaaagtgagatttgccaacatagatgggactacatttgtgttacatgatgtaagatacatgcctggaatgtctcggcatcttattctgatgggtactctagaagcaaaaggatgtgtgtttgagggaaatgatggtgtattggaggtaatgcagggaaatacagtgttcatgaaagggtcacaaagagcttctttatacatcctgcaaggagaagctaagaagtcggaagctatggtggctgagtcaggagattcggatatggatctaatgcaggtatgacatagcaggctcggtcatgtaggatagaaaggaatagatgagctggccaagaaaggatgttttggcaaggataaggtctcaagtcttaagttatgtgaagattgtgtttttgtataacacataaagtgagctttgggcaagttcaacatattactaaggagaagctagattctgtgcactcagatttgtggggatcacccaatgtgccttttagcctcgataaaatgtcagtattttctcacgttgactgatgactggtctaggaaggtttgtatatatttcctcagaaccaaagatgaagcgtttgatgcgtttgtactgtgaaagaagatggttgaggttcagagtgagagaaaggtgaaaaggttgcgtactgataatggtctagaattctgcaatcacaagttcaatgacttctgcaaacaagaaggaatggtgagacacaggacatgcacctatacattgcaacaaaacggagttgctgaaagacttaagaggactataatgaagaatgtgagaagcatgctgagtgagagtggtcttggttagaagttctgggctgaagcagcttcaacgtctttgtacctaattaatagaacaacatcatcaacgattgatttccagatcccaaaagagagatggacttcagctattccagacatgttaggactcagaagatttggatgtctagtgtttattcactcagatgaagggaaactgaatcctagggaaaaaagaggcatcttcactggttatcccgaaggcgtgaaaggttttagagtatggttactagaagatcagagatgcatgattagcagaaatgtggtcttcaaggagaaagtgatgtacaaagatgttatagctcaagagaaatcaggtatgtcctctaacccgtttgatttatctactgatgagtgtgcttgttaaaatgcaggaacagatagagcgaagaaggactcattgtagagtggagctattgttcaaggtggagaaaccacacctgaacagaatactaacgagacaacaacagatgagattgagctagaagaagttgctgggtatgcttatttagtcattgaagatggaggaaagtcagaacaaggaagttttcaggaagctttagaagatgctgatcaagataaatggatcaaagcgtcagatgaggagatggaatctctcatgaagaataaaacctggatattggtggaaaggaacaaacaacagaaacctattggttgtaagtgggtgttcaagaggaaggctggaatagcaggagttgagggtcccaggttcaaggccagacttgtagctaaagggtattcacagaaagggaatagactatcaagagatcttttcctctatagtgaaacatgtctccatcagatttttactgagtatggtaactcattttgacatggagctttagcaaatggatgtcaaaacggcgtttctccatggatttttggatgaaacaatattcatggagcagctagaaggctcattgatgagaagaatctagataaagtgtgtctgttacaaagatctctgtatagcttgaaataatcacccagacaatggaacaccaggttcaatgatttcatggaggctcataactatgagaggagctcatacgacagttgtgtgtactttaagaaatatgcagacggtgattatgtgttcatgttgctatacgtagatgacattcttattgcctccaagaatatgaagcatgtaagtgaactcaaagcactgttgggatctgagtttgaaatgaaggacttaggtgaagcaaagaagatcttgggtatggaaatagacagaaacagacttaaatgtaccctttctatctctcgggagggttatctttcaaaactattgggaacgtttaacatggatcagtctaagcttgttttaatgccagtgggaatacatttcaagttgaggtctgctacagatgaggaagctaagagtcagtatgaatcaatgagagctgttccatatcaaagcgtagtgggaagtctcatgtatgccatgataggtacaagaccagacttagcacactctattggtctagtttgcaggtttatgagtaaaccacttaaagagcattggcagggagtcaagtgggttctcagatatattcagggttcactcaagcgaaagttatgatacagtaacaaaggggatttcataattttaaggttactgtgttttagatcatggtgcagatcaagatgggaggaggtcaacttcaggagtggtattcactgtaggtgggtaatgttgttagttggaggtctagtctatagaaagtggtagcattgtcatcaacagaagctgagtgtatggcattaacagatgcgtctaaggaagcagtttggttgttagggctcatgaatgagcttggttttgagcaagaagcagtggatatttacagtgactcgcagagtgccattgcattagctaaaaatatagtccatcatgaatggacgaagcatatagagatcaagtatcacttcattagagagctgatctacaatggcttagtaagaatgaagaagattgctatagaaaccaatcctgctgatatcttcacgaaggtcgtaccggtgggcaagcttcaggaagctctagagttgctcagagttactgagaactaagaagaggggttaagtcccaagacatgaagctgagtgggtttactcaggagataagtagttgagtgagtgtactcaaagtttaaaagttggttaagttgaatcaggttcggaggtggagtcaagaactaagtcacgtcattagggtcaaggtggagaat

>ATCOPIA69A_IN

aattagtatcagagccaggttggactcaaggattcgcagatctaaccggagatgtcaaacacaaagcttaagatttcacagttcgatggttccggtgatttttcactttggaaaacacggatgttctcacatctaagggtgatggggttaaaagatgctttggtggaacgagctccgttaccaccattaaaggaagaagatgaatctgatccagctaagaagaaacagcggatcgaggaagaaaaagcaagaatcgatcaagatgaaaaggcgatggatatgatcttcataaacgtcggagataaggtactaaggaacatcgaaaattctaagactgcagctgaagcttgggcaaccctagataaactgtatttggtgaagtctcttccaaaccgagtctatcttcaactcaaggtttataattacagaatgcaagactcaaaaactcttgaagagaatgtggatgagttccaaaagatgatttcagatctgaataaccttcagattcaagttcctgatgaagttcaagcaatcttgatattaagtgccttacctgatagctatgatatgctcaaagaaacattgaaatatggaagagagggcattaaacttgatgatgttattagtgcagcgaaatcaaaggaattagaactaagagatagttctggaggatctagacctgtaggggaaggtctttatgtccgaggtaagtctcaggccagaggaagtgatggacctaaatcaacagaaggaaagaaagtctgctggatttgcggaaaggaaggacacttcaagagacaatgttacaagtggcttgaaaagaacaaagcaaatggtgcaggggaaacagcactagtaaaggatgatgctcaagatttggttggtctagtagcctcagaggttaacatgagtgagggaaaggacgatcaagaagagtggataatggatactgggtgttctttccatatgacaccaaggaaggagtatctgatggactttgtggaagctaagtcaggaaaggttagaatggcaaataattctttctctgaggtaaaaggaattggcaaagtcaagtttatcaagaaggatggaacaagtattgttctacaaggagttagatacattcctgagatttgaagaaatctgatatcaatgggaactttagaagcagaaggctatgaattcaaagcgaacaatggtgtattgagagtcatgcaggactcaagtgaattcataagaggaataaggagagcttcactatatatactagaggcacaggctagtatggcaggatcagaatctctgataacaacagcaagtgaaacagatatggcacagcagaatgggacacatagggcaacaggccatggaggttttgagtaagaaaggttgtttcggagatgacaggatatcagaaattaagttttgtgaagactgtgttatagggaaaacacacagaacaagttttggtacagctcaacatgttacaaaggagaagcttggctatgttcactcagacctttggggatcacctaacgtgcctcacagtcttggcagatgtcaatatttcatctcgtttacagatgactggtcaagaaaggtgtgggtttactttctcaagacgaaagatgaggcattcgcatcatttacagaatggaaaaaaatggtggaaacacaaagtgagagaaaactaaagcacctaagaactgataatggtctagagttctgcaatcataagtttgatgaagtgtgcaagaaagagggtatagtaaggcacagaacatgcacatataccccacaacagaatggagttgcagaaaggttgaacagaaccattatgaacaaggtgagaagtatgctgagtgaaagtgggcttgataagaaattttgggctaaagcagcatcaactgcagtgtacttgatcaacagatcaccatcgtcatccatagagaataagattcctgaagaattgtggacatcagcagttccaaatttctctggattgaaaagatttggatgcgtcgtgtatgtttattcacaagaaggaaagctagatcccagagctaagaaaggagtattcgtgggttatccaaatggagtcaagggattcagagtttggatgattgaagaagagagatgttctattagtagaaatgtggtgtttagagaagacgttatgtacaaagacatcctaaaccaatcaacctcaggtatgagttttgatttcccattagctactaaccgaattcctagctttgaatgtgcaggaaacagaaaggaagatgaaatttctgttcaaggtggagtttcagatgatgatacaaaacagtcttcagaagaatctcctataagtacaggatcttcaggacaaaactcaggtcagagaacatatcaaattgccagagacaagcctaagaggcaaacaaagatacctgacaagctgagagattatgagttgaatgaggaagtacttgatgagatagcaggatatgcctacatgattacagaagatggtggaaatcctgagccaaatgactatcaaaaagcacttcaagactctgattataagatgtggttaaaagctgttgacgaagaaatagaatcacttctcaagaacaacacttgggtgttagttaacagagatcagtttcagaaaccaatcggctgtaagtgggtgttcaagagaaagtcaggaattgttggagtagagaaaccaaggtttaaagcaagactggtggttaaaggctattcacaaaaggaaggaatagattatcaggagatcttctcaccagtggtgaagcatgtctcaattcgtctgttactgtcaatggtgactcactgtgacatggaactccaacagatggacgtgaagacggcttttcttcatgggtacctagacgaaacaatctatattgaacaacctgaaggttatgttcataagagatatccggataaggtgtgcttgctgaaaagatctctatatggactgagacaatctccaagacaatggaacaacaggttcaacgagttcatgcagaagattggttatgaaaggagtaaatacgacagctgtgtgtatttcaaagagctacagagtggagaatacatttacctactgttatatgtagatgatattctcatagcttccagagacaagaggacagtatgtgatctaaaggctcttctcaactcagagtttgagatgaaagatttgggagatgccaagaagatacttggaatggagattgtgcgagacagaaaggctggaaccatgtcaatatctcaggaaggttatctgttaaaggtacttgggaattttggaatggaccaagctaaaccagtctttactccaatgggtgctcacttcaagcttaaacctgcaactgatgaagaggttatgagacagtcagaggttatgagggcagttccatatcagagtgcggttggaagcttaatgtactcaatgataggtacaaggccggatctggctcattcagttggattagtctgcagatttatgagcaaacctctaaaggagcactggcaagctgtgaagtggatactaagatacattagagggtcaatagacagaaagctatgttataagaatgaaggagaactcattcttgaaggatactgtgactcagactatgcagcagataaagaaggtagaagatctacttcaggagtggtatttaccttcggtgggaacacaataagctggaaatccaattttgcagaaggtagtagcgttgtcaagtactgaagcagaatatatggcattgacagatggagccaaagaagcaatttggttaaaaggtcatgtgagtgagctaggttttgtgcagaagacagtgaacatccactgtgactcacaaagtgcgattgcattagcaaagaacgcagtctatcacgagagaaccaaacatatagatgttaagtaccatttcataagggatttggtgaataatggagaagtgcaggtgttgaagatcgatactgaagacaaccctgcagatatattcaccaaggtgttaccagtgagtaagtttcaagatgctcttgaactactcagagtttcacagaattaaggtagagctaagctccggacaaaagaagctgagtgggtttactcagggagaagaagctgagtgggtttactcagggagaagaagctgagtgggtttactcaggaatcaaaaggtcaagtgagtctacttgaagaaaagctgaacaggttggttcaggtaccggagaggaatccaagttaaagctaaaacaggaagcattcggatcaaggtggagatt

>ATCOPIA69A_LTR

tgaagaagaatgatcctaatgctaagtatacgagacactctaggttttgacttttggtcaagtcgagtagctgagattgtgacacgtggttgaatctgaagcgtccatctctaacggacttttcttatcttcttcttcttcgtttctcttcttaaacacttctgttgtaattctcaaattcattcgatctagtgtgataaggatctagagagagagagagagcagaaacattgtagtgtgagaaagctcggtttgagcaaatttgtttcagtggattctggagaactctccagcgagacgtaggattccaaattggaatccgaactcgttaattcttgtgtgtctgttctaagttctttcaagtgtgacattaatcgcgagagtcagtgagttagatcggagttcttgaggatcaaagctcaca

>ATCOPIA69_LTR

tgaagaagtattgatctatatgctaagtataagagactctctaagtgtttcagaaggtttcagggtcaactggttgacgagctaaggagatcgtgacacgtggagcgatccaatccgtttactctcaacggcattttcttatcctttactcgtcttattctgtttcctttcagtattgttgtttctctgtagatttgattgatttagaaatttgttgaataagagaagtgagagagagaaaacagaacgattgtagagagagaaagctcgttttgagctaagtgcttttagtgaattccggagacatctccggtgagacgtagggttcctcatcggagcctgaactcgtaaaatcttgtgtgtctcttttaaaggttctttgtgttcaatctcacacgagtgtccgatcgatttcgttagtgttcttgagggttcgaggatcaca

>ATCOPIA69_IN

aattggtatcagagcccaggttctgagctcaagaatttcagatcgattcaaggatgtcgaacacgaaactgaagatttctcagttcgacggatcaggcgacttctcactatggaagatgggattaaaagatgcgctggtggaacaaactaagtcatcttcattgacagatgaagaagaagacgatccagcaaagaaaaaaaagattctcgaagaggaaaaagcaagaattgatcgagatgagaaagcgatggatatgatcttcataaatgtcggagataaagttctgagaaacatagaacattcaaagacagccgcagaagcatgggcaactcttgataaattgtatttggtaaagactctaccaaaccgtgtttaccttcaactcaaggtttacaactatagaatgcaagattcaaaaactcttgaagagaacatagatgagtttctaaagatgatatcagatctaagtaatctttagattcaagttccagaagaagtccaagcaatcttgattctaagtgctttaccagaaggctatgatatgcttaaggaaaccttgaaatatggaagagaaggcataaaacttgatgacgttgtgagtgctgcaaaatcaaaggaactagaactaagagatggtttaggaggatcaagaccggttggtgaaggtctctatgtaaagggaaagtttcaggccaaaggaagtgataacaacaaagggaataactcaacagaaggaaagaaagtctgttggatatgtgaaaaggaaggtcacttcaagagacaatgttacaagtggcttgagaagaataagggaaatggtgcaggggaaacaacattggtaaaggacgatgctcaagacttggtcgggctagtagcatcagaagctaacctaagtgaggataagagagatcaagaagaatggataatggacactgggtgctctttccacatgacacctaggagagactatcttgtagactttgtagaaggcaaagcaggaaaggttagaatggctaataattcattctctgaagtaaaaggaattggaaaggttaagttcacaaatgaggatggaagacagatcatccttcatggagtgaggtacatcccagagatatctagaaatctgatctctatgggaactcttgagtcagagggatatgagtttagaggaggtaacggtgtcttaaaggtaattcagggatcataagtgttcatgaaaggagtcagaagagcctcgttatacattttacaagcggaagcgagaaagtcagatgcagactctcttacaacagtctcaggtgaatcagatcagactcagttatggcatagcagaatgggacatataggacagcaggctatggaagttttgagtaagaaaggttgctttggtaatgacaagatatcagagataaagttttgtgaagactcataatagggaagactcacagagctagtttcggatcagcacaacatgtaactaatgagaaacttgactatgttcattctgatctatggggatctcctaacgtaccgcacagtcttggaaaatatcagtacttcatatcatttacagatgactggtcaagaaaggtttgggtgtactttctcaagtctaaagatgaagcctttgcttcattcactgaatggaaaaagatggtggagactcaaagtgacagaaaactcaagaaattaagaacagacaacgggttagaattctgtaatcaaaagtttgattgtttctgcaagaaggaagggagagtaagacatagaacatgtacttacactccacagcaaaacggagttgcagaaagattaaatagaacaatcatgaacaaggttagaagtatgctgagtgaaagtggcttagacaagaaattttgggctgaagcagtttcaacctcagtatacttgattaacaaatcaccatcatctacaatggagaataaaatccctgaagaactgtggacctcagtgatttccaatctgtcaagactaagaagatttggctgcattgtatacgttcattctcaagaaggaaaactggatcctagagccaagaaaggagtgtttgtgggttatccaagtggagttaagggttttagagtctggatgattgaggaagagaagtgcaccataagtcgaaacgttgtgttcagagaagatgtgatgtacaaggacatcatgaacgccacaacctcatgtataagtcttgaactccctttgactactaataaagttcccatcttcgaatgtgcaggtgccagtaaaaccagagacagttcagatcatggtggagctacagagagtatttctgatgagactacagaaatcattgacattgatcaggtagacactacaccagaaggaaatcagagaacaagacagatagctcgagatcgacctaaaagacaagtgattatcccatcaagactcaaggattatgagatggatgaggaagtattagatgagattgcaggctatgcttacctcataacagaggatgggggaaattctgaacctgagtgctatcaggaagcagttcaagaccctgatagtgagaaatggttagaagcagctgatgaggagatagaatctctgataaagaataagacatgggttcttgtagagagaaacagtctacagaagcctattggatgtaagtggatattcaaaaggaaagctggaattgcaggagtggagaaaccaaggtttaaggctaggcttgtagctaagggatactcacagaaagagggaatagactttcaagaaatattttcaccagtggtgaaacatgtctctattcgcctcctgctatcaatcgttgctcacctagacatggagttacaacagatggatgtaaagacagcctttttacacggctacctggatgagacgatctatatggagcaaccagagtgatatactcatgaaagatatccagacaaagtttgcttactgaagaagtcgctgtatggactgaagcagtctcctagacaatggaacaacaggttcaatgagtttatgcagaagattggatatgaaagaaacaagtatgatagctgtgtttacttcaagatgttgcagagtggagagtacatctacttgcttctatacgttgatgatatactaatagcatctaaggataaaaaggaggtatgtgagttaaaggttcttctaaactctgaattcgaaatgaaagacttgggggatgctaagaaaatcttaggtatggagatcgtcagagatagacaagctggaactctctccatttctcaagagggctatctcctgaaagttcttggagattttggcatggatcgagccaagacagtcaacacaccctggggatccattttaaactgaaacctgcaactgatgaagagattcagaaacagtcagaagtcatgagaacaatcccttatcaaagtgcagttgggagcttgatgtactcaatgattggtacaaggccagacctagctcattcagttggtgtagtatgcagattcatgagtaaaccattgaaggaacactggcaggcagtaaagtggatattaagatacattggtggtactttagaccgaaagctgtgctataagaatgaaggagagctagtcttagaaggttattgtgactcggactatgctgcagataaggaaacaaggagatccacttcaggagtggtgtttacctttggtggaaacacgataagctggaagtcaagcttacagaaagtagtagctctatcaagcactgaagctgagtatatggctctaactgatgcagcaaatgaagcagtttggctgaaaggtcttgtaagtgagttaggttttgcacaaggatcagtaaacatccattgtgactcacagagtgctattgccttgactaagaacgcagtctaccacgaaaggaccaaacatattgatgttaaatatcacttcatcagagaattggtgaacgatggtgtggtgcagatattgaagattgacactgaagacaatccagcagatatattcaccaaagtgctaccagtgagcaagtttcaagacgctcttgacttgctcagagtatctcaaagttaaggtggagctttgctccgggttttaagctgagtgggtttactcagggagaagaagctgagtgggtttactcagggagaagaagctgagtgggtttactcaggaaaaagaagctgaacaagttagttcaggtaccggagaggaattcaaaagctaaaggcagcagagcataaagatcaaggtggagatt

>ATCOPIA6_IN

tggtatcagagcttgtcgatacctaaatctaaagaaaaaaaaaagaaaaaaaaattccctaccgccgctctttcttcttctcttttccatctctctttttctctctcttctttcacgatgtctgattcaacctccccagtatctgtctccgagaccatcgccgtctccacttccaccttgctgaatgtcaacatgacgaacgtcacccgcctcaccgattccaactttgttatgtggagtcgtcaggttcacgccctacttgatggttatgatctagccggctacatcgacggatctatccccatcccaaccccgactcgcaccactgctgatggcgttgtcactaccaacaacgactatacactttggaagcgacaagacaagctgatttatagtgctctgcttggtgctatatctctctctgttcagcctcttctctcgaaggcgaacacatctgcggagatctgggaaacgctttcctccacgtttgctaatccgagttgggctcatgtccaacaattgcgtcagcagctcaaacaatggacaaaaggaaccaagtccattgtcacctattttcagggttttacaacccgctttgatcatcttgcgctacttggaaaggctcctgaacgtgaagaacaaattgagcttattcttggtggcctccctgaagactacaagacagtagtcgaccaaatcgaaggccgtgaaaatcctcctgctctcaccgaagtccttgagaagcttattaatcatgaagtgaagctggcagccaaagcagaagccacctccgtccctgtcactgcaaatgctgtcaactatcgtggtaacaacaacaacaacaacaactctcggagtaacggtcgcaacaactctcgtggtaacacttcatggcagaatagtcagtccacctcaaaccgccaacaatacacaccacgtccctatcaaggcaagtgtcagatctgtagcgtccatggtcatagcgcacgtcgttgtccacaacttcaacaacatgctggttcatacgcctctaatcagtcgtcatctgcctcttatgcgccatggcagcctcgtgctaacatggtttctgcgacaccatacaactccggcaattggctattggatagtggagcgacgcatcacctcacttctgacctgaataatctcgctttacaccaaccgtacaatggtgatgaagaggttaccattgctgatggttcgggtttgccaatctctcactctggttctgccttactccctactccaactcgctctcttgctttaaaagatgtcttatatgttccagatattcagaaaaatcttatctctgtttatcgaatgtgtaatactaatggagtatctgtggaattctttcctgcacactttcaggtgaaggatctcagcacgggggcccgattactccaaggcaagactaaaaatgagctgtatgagtggccggtcaattcttccattgctacctccatgtttgcttcacccacaccaaaaacagaccttccatcatggcatgcaagacttggccatccctctttacctattttaaaagcacttatttcaaaattttctttacccatttctcattctttgcaaaaccaattgttatgttctgattgctctatcaataaaagccataaacttcccttttactcaaacactattgcctcttctcatcctcttgaatatttatatactgatgtttggacctctcctattacttccatagataattacaagtattatctggtgattgttgatcattatacccggtatacttggctctatcctttacggaaaaaatcgcaggtccgtgaaatgtttatcacattcacagcattggtcgaaaacaaattcaaattcaagattggcacgctgtactccgataatggtggtgagttcatcgctatgcgctcatttcttgcctcccacggtatctcacatatgacgacaccaccacacacaccggagctcaacggtatctctgagcgcaagcatcgacacattgtcgaaactggccttactcttctaagcaccgcctccatgccaaaagaatattggagttatgcgttcgccacggccgtttacctcattaatcggatgctaacacctgttctaggcaatgagtctccatacgtgaagttgtttggtcaacctcccaactaccttaagcttcgaatttttgggtgcttgtgcttcccatggctgcgtccatacactgctcacaagcttgataatcggtcagtgccgtgtgtgctccttggctactctttgtctcaaagtgcctatctatgtctcgatagagctactggccgtgtttacacctccagacatgttcagtttgctgaatcgagcttcccgttttcgaccacatccccttctgtcacacctccatcagatcctcctctctcacaagacacaagacccgtctcagtcccgcttcttgctcgtccactcactacagctccgccttcgtcgccctcgtgctcagctcctcaccggtcaccgtcgcagtccgaaaatctctcgccaccggctccgttacaaccatcactgtcattgtctccaacttcgccgatcacgtctccttcgttgagtgaagaatctcttgtgggccacaattcagaaacaggcccaactggttcttctcctccactcagcccacagccacaacgcccacagccacaaagcccacaatccacaagcccacattcctcaagcccacaaccaaatagcccaaatccacaacattccccacgttcgttaacacctacactcacgtcctcaccatcaccctcacctccaccaaacccaaatcctccaccaattcaacacaccatgagaacacgatccaaaaacaacattgtcaaaccaaaccccaaattcgcaaaccttgctacaaaaccaactccactaaaacctataattcccaaaaccgtggttgaggcgttacttgatccgaattggagacaagccatgtgtgatgagattaatgctcaaacacgaaatggcacgtttgatttggttcctccagcacccaatcaaaacgttgttggttgtaaatgggtctttactttaaaatatttgtctaatggtgtacttgataggtacaaagcgcggcttgtggcgaagggattccatcaacaatatggtcatgactttaaggagacattcagtccagtgattaagtcaaccaccgtacggtctgtgcttcatattgccgtaagtaaaggatggagtatccggcaaattgacgtcaacaacgccttccttcaaggcaccctatcggatgaggtatatgtgacacagcccccaggatttgtggacaaagataatgcccaccatgtgtgtcgtctctacaaagctctctatggtctcaaacaagctccacgtgcgtggtaccaagaattgcgctcgtatcttcttacacaaggcttcgtcaactctgtcgccgacacgtctctattcactcttcgtcacgagcgcaccatactctatgttctagtttatgtggacgatatgctcatcactggcagtgacacaaacatcatcacgcggtttattgctaatcttgcagctcggttttcacttaaggatctaggggaaatgagttattttctcgggattgaagcaactcgcacatccaaaggacttcacctaatgcaaaaacggtatgttcttgatctgttggagaaaactaatatgcttgctgcacatccggttctcactccgatgtccccgactccaaagctatcgcttacctcaggcaaaccacttgacaaaccaagtgaatatcgggccgtgcttggtagtctccaatatctcttgtttacaagaccggacattgcctatgcggtgaatcgcttgtcacaatatatgcactgtccaaccgatcttcattggcaggccgccaagcgcattcttcgatatcttgctggaacaccatctcatgggatcttcatccgagctgatacgcctctcacactacatgcttactctgatgcggattgggccggtgatattgacaactacaactctaccaatgcttacattttgtatcttgggagtaatccgatttcatggtcatccaagaagcagaaaggtgttgctcgatcatccaccgaagcagaatatcgagcagttgcgaatgccacttccgagattcgttgggtatgctccctcctcactgaacttggcatcacattgtcgtcaccgccggttgtttactgtgacaatgtaggtgctacatacctatctgctaatccggtattcgactcacgaatgaaacatatagcacttgatttccactttgttcgtgaaagtgtccaagctggagctctccgtgttactcatgtctctaccaaagatcaactcgcagatgccttgacgaagcctctaccacgacaaccgttcactactctcatttccaagattggtgttgctaaagcacctccatcttgagggggcg

>ATCOPIA6_LTR

tgtaagggagaaatgactaaggatataattgtaaattatagatacctaattactcttgtaatcactatatatattgtacaaacatctatgtaataaactattgagcctcatttcataca

>ATCOPIA70_LTR

tgttggagttggagatttggtttaaaggtaaaccgggtttgctttagctacaaagttgcgtgaagaggaagttgccgttaaatcagcaatgtgtatcagattcattccataaaatctgcatcgttaatattgtttctatattttctgtaaatatacgatattgtgatagcatagagatcgtgttctctaaagactatatatgtacatgtaatcgttcataataaagatcagagaaactctgtttatttca

>ATCOPIA70_IN

tggtatcagagcttgagaaatcaattttgattttcttttgaaacgaagagtgacaatggttggagctcctgcgaacgtgaacacgaacacgaacacgaacccgatcccgaacccgacagtggaggtgagaagaacaattttaccctacgatctaacctccgctaataatccttgtgcagtaatctcacatccattgttgactggaagtaactatgatgaatgggcttgtagcatgaagacggcgctgtgttctcgcaagaaatttggattcctcgacgggacaatctcaaaacccgtagaaggatctccagatttggaggattggtggacaatttaagcactacttgtgtcttggattaagatgagtatcgatccggtggtgaaaaagaatgtttctcatcgtgatgtggcgaaggatctctgggatcatttgaagaaaaggttctctgtgatgaatgggccaaagcttcaacaactgaaatctgagttagcgtgttgtaagcaaagagggcttacaattgagacatattatgggaagctaacgaagatttgggatagtatggctagctttcaaaccggtgcgcatctgcaagtgtggtggttgtgtgtgcgatcttggtgctcttcaagagaaagatcgagaagaagacaaagttcatgaatttttgtctggtcttgatgatgctttgtttcgaacggtcagatcgagtcttgtgtcacgtattcctgtgcaacctttagaagaggtatacaacatagtgcgacaagaggaggatttgctcaggaatggggcaaatgttttggatgatcaacgtgaagtcaatgcatttgcagctcaaatgagaccgaagctttatcaaggaagaggtgatgaaaaagataagagtatggtgtgtaaacattgtaatcgcagtggtcatgcatccgaaagctgttatgccgtcattggctatccggagtggtggggagataggccacggagcagatcattgcaaactcgaggtcgtggaggaaccaattcgagcggagggcgtggacgtggtgcagctgcctatgccaatcgtgtgacggttccaaaccacgacacatatgagcaagctaactatgctctcactgacgaagaccgtgatggtgtgaatggtctcaccgacagtcagtggagaaccatcaaaagcattctcaactccgggaaagatgctgcaactgaaaagctaaccggtaagtttacttcaccctcttggataatggacactggagcctctcatcatttgacgggaaaatttgatatattgatgatgttagagatatggacccaattttgatagttctagctgatggtagggaacgaatttcagttaaagaaggaacagttcgacttgggtcaaatttggttatgatatctgtattttatgttgaagaatttcagtccgatttaatctcaattggacaattgatggatgagaatcgatgtgttttgcaaatgtctgatcgcttccttgtcgtacaggaccgcacttcgaggatggtgatgggagctggtaggcgtgttgggggaacgtttcactttcgcagtacggagatcgcggcatcagtcacggttaaagaggagaagaattatgagctgtggcatagtcggatgggtcacccagctgcgagagttgttagtttgatacccgagagttctgtttctgtttcttctacacatttgaataaggcatgtgatgtttgtcatcgtgctaagcaaacaagaaattcttttcctttgagtatcaataaaactttgcgaatttttgagttaatttactgtgatttgtgggggccttatcgcacaccgtcacataccggtgctcgctattttttaactataattgatgattactcaagaggggtttggttgtacttgttgaacgataagagtgaggcgccatgtcacttgaagaatttctttgcaatgacagacagacagttcaatgtcaagataaagacggtgagaagcgacaacggaacagaatttctgtgtttgactaaatttttccaggaacaaggagttattcatgagcgatcttgtgtggctactccagagcgaaatgacagagttgagagaaaacatagacatttacttaatgttgcaagggcgttgaggtttcaagccaatctaccaattcagttttggggagagtgtgtgttgactgcagcatatcttattaatcgtactccaagctcggtgttaaatgacagcacaccatatgaacgtttgcataagaaacagcctcgatttgatcatttgcgtgtttttggaagcttgtgctatgcacacaatcgtaatcgtggtggtgataaatttgcagaaaggagtcgacgttgtgtctttgttgggtaccctcatggtcaaaaaggatggagattgtttgatcttgaacaaaatgaattttttgtgtcgagagacgtggttttctcagaactagaatttccatttcgaatttctcacgaacagaacgtaattgaagaagaagaagaagctttatgggctccaatagttgatgggcttattgaagaagaagttcacttgggccaaaacgcaggcccaacacctccaatttgtgtttcttctccgattagcccttctgcaactagttcacgatctgaacactcgacgtcttctccgttagacactgaagttgttccgacgcctgcaacttcaacgacatcggcttcgtcaccctcctcgccgacgaacctgcaatttttgcccctgtctcgagctaagccaacgaccgctcaagcggtcgcaccaccggcggtccctcctccacgacgtcaaagtacaagaaacaaagcaccgcctgtgactcttaaggattttgttgtcaacacaacagtctgtcaagagtcgccatctaaacttaattcgattctctatcaattgcaaaaacgggatgatactcgtcgtttctctgcctctcatactacttatgttgctgtaattacatctgcgattgaaccatgatcgtataaacaagcaatgatggataaaagatggattaaagcaatgggtttggagattgatgctcaagaagaaaatcatacatggaccattgaggatctacctcccgggaaacgagcgattggtagtcaatgggtctataaggtgaaacacaattctgatggctcggttgaacggtataaggcgcgactggttgcgctggggaacaaacaaaaagaaggtgaggactatggtgagactttcgctcctgttgcaaagatggctactgtgcgcttatttcttgatgtagcagtaaaacgcaattgggaaatccaccagatggatgttcacaatgcgtttttacatggagatttgcgtgaagaagtatatatgaagctacctcctggatttgaagcttcacatccgaacaaggtttgtcgtctccgtaaagccctatacggattaaaacaggctcctcgatgttggtttgagaagttgacaacagctttaaagaggtatggatttcagcaatcactagctgattattcattatttactcttgtcaaaggttctgttcggataaaaattctgatttatgtggatgatttgattatcactggaaattctcaaagagcgactcagcaatttaaagaatatcttgcgtcttgcttccatatgaaagatttgggtccattgaagtattttttgggtatagaagtggcaagaagtaccacaggaatttatatctgtcagaggaagtatgcgttagatatcatttccgaaaccggtttgttgggagttaagccggcgaattttcccttggaacaaaatcataaacttggattgtcaacatcaccacttctcacggatccacaacgatatagaagattggttggtagattgatttatcttgcggttactcgacttgacttggcattttcagtacatattttggcacggtttatgcaagaaccaagagaagatcactgggctgcagcattgcgtgtagttcgctatctcaaagctgatcctggtcaaggggtttttcttcgacgttcgggtgattttcaaatcacaggatggtgcgactcggattgggctggtgatcccatgagccggcgttcagtaacgggttattttgtgcagtttggtgactctcctatctcttggaaaacgaaaaagcaagatacagtgagtaaatcatctgcggaggctgaatatcgagcaatgtcgtttcttgcttcagaattgttgtggttgaaacagctcctgttttcacttggtgttagtcatgtacaaccgatgataatgtgctgtgacagtaagtccgctatctatatcgctacgaatcccgtatttcatgaacgcacaaagcacattgagatcgattatcactttgtgcgtgatgagtttgttaaaggtgtaatcactcctcgacatgttggcacaacgtctcaactcgcagacatcttcacaaagcccttaggacgtgattgtttctcagcattcaggatcaagttaggcatacgaaatttgtatgctccaacttgaggggggg

>ATCOPIA71_LTR

tataacgagtattttactctatagtataaaatatgtattagagtataaatacatgacattgtacattttaccaaattaatgagaataaacgattctttgtttctgttatcttcttcctcttctttcactctctctctagagtttgttacagagattaaagaatcctgata

>ATCOPIA71_IN

tggtatcagagcatctccgatcatgagttgttcttcgattgaaatcgttctcattcatcttctttgttttcttttggattcatgttgtatcatctttctcgcgatttttcaattcatgttcgtgagagaaatctgagtttcttcttcgttttttttgaccttattgctttgattctctttatatcttcaatccggagcaatttctatcatcgtttgagctcaaatcaactatgggttcaacaccacttcgtccatcttttcttgatgcaacatcgaataacttcaatcaccacaacgatgtcgatagacaatcggttatggatcattacgataatccgttcttcctccatagtactgatcatgctggtttacaactggttacagatcgattaacctctggtgcagatttccattcttggagacgatcggtgaggatggcgttgaatgtgcgtaacaaattgggattcgtcgatggtacaatctctaaacctcctcccaatcatcgtgattctggatcttggtcaaggtgtaacgatatggttgcaacttggttaatgaattcagtgagtaaaaagataggtcagagtctattgttcatgtctactgctgaaggaatctggaataatttgatgagtagatttaagcaagatgatgcaccacgagtgtttgaaatcgaacaaagattgagtagtatccaacaaggatcgttggatgttagtgcttactatactgaattgataactctgtgggaagagtacaagaattacattgagttacctgtgtgtacttgtggtaaatgtgagtgtaatgctgcagtactttgggaaagattacaacaaagaagtcgggttactaagtttttaatgggactaaatgaagcttatgagcagactcgaaaacacattctgatgttgaagcctattcctagtattaaagaagcatataacattgtggcacaagatgcgtcaacgtgttgttaaaccgatgatcaagacagacaatgttgcttttcagacttctgaatcttatgatggtaattcatatcagggtgaacagccagaatttgttgctgcttataatgcatatcgacctaagagtaatcgaccattgtgtactcattgtgggaagttgggacacactcttcagacttgttttcaggttcatggttatccaccgggttacaagattccgggtcaggggaatactcaaggtaataaggcttcatacaatcctcgtggtcagacagatttcaactcaaagccagtcttcaggccacaggctaataacatgccagagcagagaacagttgccaatgtattcacaggacagcttcctgctagtgacacagttccttattatccttctccggccatgaatgcagtcaatcttgatgtcagtaggttgacacaagagcaagctcaaacactaatcagtcagctaagttctcatgctcaagttcctgtttcagagtctctaactcctcaattccctactattactgaacatgggattatggctgttcaatcttcatctggtacagttttcaatatctcttctaatcttagatatgagaataataatctcacatacaatcaccaatgtctctcttctttacatactgctttgcctaatgatgcttggataattgatagtggagctactagtcatgtttgttgtgatttttcaaaatttaaagagactagtttagtatctggaataacagtagcattacctaatggcattagtctccctatcactcatacatgcaccattcatttatctgattcattggttcttcacaatgctttgtttgttccatcatttcgttttaatttgattagtgtgagtactcttttacgagataataaatgttttgctcacttttatcctacatcatgttttttacaggagtgttctcggggatggatgattgggagtgctaatcttcatcacaatctctacatcctcaatcttcaatccctgaatacaccagttgcatcatctagtgtcatcaacttctgtggatccttgtcagctgatgggaatctatggcaccaacgccttggtcatccctcaactgccaagttagaacttctctctgattgtctttctcttaataagtctgcattagctagtcctcatcattgttctgtgtgtcctttagctaagcagaagcgtctgtcttttccttttaataataatgtgtcttctaatccatttgacttagttcatttggacatatggggacctttttcagtagagtctatagaaggatataagtactttctaactgtagttgatgattgtactagagttacatgggtttaaatgatgaaatataaaagtgatgtgttacagatttttacaaacttcattaaacttgttgatactcaatatcagataagaataaaagctattagaagtgacaatgctcctgaactgaaactaactgaattgataaaagaaaatggcatgattcactatttttcttgtgcatatactccacaacaaaattcagttgttggaaggaagcatcaacacttgttaaatgttgctcgtgcattgctttttcagtctaatgttcctatcatatattggagtgattgtgtttcgactgttgcattcttagtgaataggattccatctgtcttacttaataatgtcacaccctatgagaagttactgaaaaagaaacctacatatgcatcattgaaaatttttggctgtctatgttatgcaagtactttacaaaaagatcgtaacaagtttactctgagagcacaaccttgtgttttcttggggtatccctctggatacaaaggatacaaagtcttagatcttgattctaatgtcatttcgatcactaggaatgttgtttttcatgaacaaatttttccttttcataataaagaaaaatatcaaactgatttcttttcacataccattctgccaaatcctgttccatttattagtgaatcatctcatccatcatctgaaacgattattcctgctgcatctcatactcctgctgcatctgcatctgaaacaattgttcctgctgcatctcatactcctgatgcatctgcatctgaaacaattgttcctgctgcatctcatactcctgctgcatctgcatctgaaacaattgttcctgctgcatctcatactcctgctgcatctgcatctaataatgaaatatctgaatcggttttacctgctgcatctttgactgagatttttaaaacagcagttccctctacagtagtaaatgagacttatgataatgatgcatttgttcgtgacttgattgaaagtgaaaaaggaaatgttgcatcatcatcatcatcatcattacctcagagggagactaggttgcctaatgagaatgttcctgttatttcgaatattaaatctgtgtctttacctgttgaaaggactaagaggcagactagggctcctagttatttatctgagtatcattgtgcattagcacaaatttctactccattaccatctaaccatactactccatatcctctatcatctgttcttagctatgacctttttaaacccactttttgttcttatatcttatcttattccctggaaattgaactgaaaactttcaaacaagccatagtttctgacaaatggaaaggggctttaaatgaggagttacaggctatggaacagaacaagacatggagtgttacatctttacctcctggaaaaaatgtggttggttgtaaatgggtcttcacgatcaaatacaactcagatgggactgtagagcgttacaaagctcggctcgttgcaaaagggtttactcaacaggaaggggttgattttaatgagactttttcgcctgttgctaaactcacaagtgtctagatgatgttaggcttggcagcaagagaaggttgggagttgagtcaaatggatgtatctaatgcctttctgcatagtactttggatgaagaaatttacatgagcttacctcaaggctatactccaggtacttcagaacctttaccgccgaatgctgtgtgtagattacacaagtccatttatggcttgaaacaggcgtctagacaatggaatcaactttttacatctgtgcttctaactaatggttttatatagtctcagtctgatactacattgtttgtgaagtttacggagactagcttcatagctttgttggtttatgtggatgatatagctattgctagtaacaatgctgatgatttgaaatctttgaagatggttttagcaaaggcttttaagataaaagatctaggtcagctgaggttctttttaggcttggagatagctagaacttcaaaaggaatatctgtttgccagagaaaatacacgttggacttattagaagatgctggtcttcttgcgtgtaaacctagtgtagtacctatggatccctatgtcaccttgagtaaagacacaggagttccattggtatctgctacgccatttagagagcttattggcaggttgttatatttgacgataactagacccgacattgcttatgcggttcacaagttaagtcagtttattcaagctccaacagatgttcatttacaagctgcacataggattcttaagtatctgaaagatagtccaggacagggacttttctattctgcaacgactgatttgtgcattaatgcttttgctgatgctgattggggtacatgtcctgatagtagaagatcagtctctgggatttgtgtttacttaggcacttcgttgatttcctggaagtctaagaagcagcaagtggcaaggagaagtagtacagaggcagagtaccgaagtctagctgatgttactcgtgagattctatggatacaacaactattgaaagatttcagagtgactgtgactgcaactgccaaagtcttttgtgataacaagtctgctatctatattgctacaaaccctgtgttccatgaaagaaccaaacacatagaaatcgactgtcacacaacgagagaccaaatcaagcttggaaacttgaagctgctacatgttacaagtgagaaccaactggcagatattctcaccaaaccgctgcatactcgaatcttttattctttacttactcgaatgtctttgtcaaaccttttcaatcctactgttttggattcaaaggtttgcgggggtg

>ATCOPIA72_LTR

taatagagtagctaggtgtaaataaccggttctatgtaattagtcggtttagcttggtttagttattataccatacgtgtatataagagaaacaattgtacacattattattaagatgaataaaactttccctttctataacaaactctctctcatcgtttcttcatcgtgaaa

>ATCOPIA72_IN

tggtatcagagccatcgagctcaattttttcttgattttgttcgatttcttcttcttttgagctagttttcttcgtttcttcacgatccaatggttcctggaattcgtgttactcgaaaatcagctcgctcgaaggtgtcgacgggttctgttgctcggaaatcatcaaagtccaccggtgttctcgattctgcttccgattctcctccaatggctcgttctcagaccgctggagcttcgcgaggtgttttctcatcgggatttgatgatccgacgcagtctcctttcttccttcatagtgcagatcatccaggtttgagcatcatttctcatcgtttagatgaaacaacttatggtgactggagtgtggctatgaggatctcgttggatgctaagaacaaactaggatttgtagacggatctttacctcgtcctttagaatcggatccaaatttccgtttatggtctagatgcaacagtatggtgaaatcctggttgcttaactctgtttctcctcagatctatcgtagcatcttacgtctgaatgatgctacagatatttggcgtgatctctttgacaggtttaacctgacgaatcttccacgtacctacaatctgacacaggagattcaggatcttcgtcaaggaacaatgtctctatctgagtactatactcttttaaagactctctgggatcagcttgacagtacagaggctttggatgatccttgtacttgtggaaaagctgttcgtctgtatcagaaggcagagaaagctaagataatgaaatttcttgcaggattgaatgagtcttatgccattgttcgtagacagatcattgcaaagaaggcacttcctagtttggcagaagtttatcatatcttggatcaggataatagccagaagggattctttaatgttgttgctccacctgcagcttttcaagtctctgaggtatctcattctcctatcacttctcctgagataatgtatgttcagagtggaccaaacaaaggtcgtcctacgtgttcattctgcaacagagttggtcatatagctgaaagatgctataagaagcatggttttccaccaggtttcactcctaaagggaagtcctctgataaacctccaaaacctcaggcagtggcagctcaggttactctttctccggataagatgacaggacaacttgagactcttgctggtaacttctcccctgatcagatacagaatttgattgccttgttcagttctcagttgcagccacagattgtttctcctcagactgcttcttctcagcatgaagcaagttcttctcagtctgttgctccttctggtatcttattctctccttccacatattgctttattggcatcttggcagtttcacataactctttgtccagtgacacttgggttattgactctggggctacacatcatgtgtcccatgacagaaaattgtttcagactttagatacttctattgtgagttttgtgaatcttccaacaggtccaaatgtcagaatcagtggagtgggaacagttttgataaacaaagacattattctccagaatgttttgtttattcctgaattcagattgaatttgatcagtatcagctctttgactactgaccttggtactagagtgatctttgatccttcttgctgtcaaatacaggatcttaccaaggggttgacgcttggagaaggtaaaaggattgggaatctctatgtgttggacacacaatctcctgctatctcggtgaatgcagttgtggatgtgagcgtgtggcacaagagacttggacacccatctttttcaagactggattctctttctgaagttttgggaactactagacataagaataagaaatcagcttattgtcatgtttgtcatttagccaaacaaaagaagttgtcatttccttctgcgaacaacatttgtaattcaacatttgagctgttacacattgatgtttggggacccttttcagtggagacagttgaaggatacaaatatttcttaactatagttgatgatcattctagagcaacgtggatttatttgcttaagtctaagagtgacgtcctcacagtgtttcctgccttcattgacttagttgagaatcagtatgatacaagagttaaatctgtgagatctgataatgctaaagagttggctttcacagaattttacaaagcaaagggaatcgtttcttttcattcttgtcctgagacaccagaacaaaattcagtggttgagaggaagcatcagcatattcttaatgtggctcgggctttgatgtttcagtctaacatgtctttgccatattggggtgactgtgttttaactgctgtcttcttgattaacaggacaccttctgctttgttatcaaacaagactccttttgaggttctcactggaaagctaccagattactctcagctcaagacatttggttgcctttgctacagctctacttcatcgaaacagcgacacaagttccttccaaggtcaagagcgtgtgttttcttgggctatccgtttggttttaaaggctacaagttgttggatttagagagcaacgtggttcatatatcgaggaatgtggagtttcatgaggagttgtttccattagcgagttctcaacagtctgctactacagcttcagatgttttcacaccaatggatcctttgtcctcaggtaattccatcacttctcatcttccatcaccacaaatttctccatcaacacaaatttctaaacgtaggattactaaattccctgctcatctccaagactatcactgttattttgtcaataaagatgactcacatcctatttcatcttctctttcttactctcaaatctcaccatctcatatgttatacatcaataacatttccaaaattccaatccctcaatcttatcatgaggcaaaggattccaaagaatggtgtggtgctattgatcaggaaattggtgcaatggaaaggactgatacttgggagattacaagtttacctcctgggaagaaggcagttggatgtaagtgggtatttacagtgaagtttcacgcagatggcagtttggaaagattcaaggccagaattgttgctaagggttatactcagaaggaaggtttggattacactgagactttctctcctgttgctaagatggccacagtaaagttacttttgaaagtttcagcttctaagaagtggtatttgaatcagctggatatatctaatgcttttctcaatggagatttagaggaaaccatatatatgaagctgcctgatggttatgcagatattaagggaacttctctgccacctaatgttgtttgtcgtttgaagaagtccatttatggtcttaaacaggcatctcgtcaatggtttttgaagttttctaactctctgttggctctgggtttcgaaaaacagcatggtgatcatactctctttgttcgctgtattggttctgagttcattgtcctcttagtttatgttgatgacatagtgattgcgagtactacagaacaagcagcacagtcgttgacagaggctttaaaagctagctttaagctgagggaacttggtccactgaagtatttcttgggtttagaggttgctcgcacttctgaaggtatttccctatctcaaaggaagtatgctttagaattgctcacttctgcagatatgttggactgtaaaccatcctccatacctatgactccgaatattagattatctaagaatgatggtctactcttggaggacaaagaaatgtatcgaagacttgttggcaagttgatgtatctgaccataactcgccctgatatcacatttgcggtgaacaagttatgtcagttctcttctgctcctcgtactgcacatcttgcagctgtctacaaagtcttacaatacattaaaggtacagtgggtcaaggtctgttttattctgctgaggatgatctgactttaaaaggctatactgatgcggattggggtacttgcccagatagtcgtcgatcaaccacaggtttcactatgtttgttggttcctctctgatatcctggcgctccaagaaacagcctactgtctcacggtcgtctgcagaggcagagtatcgagcattggctttggcttcttgtgaaatggcgtggctgtctacactgttattggctttgcgtgttcattcaggtgtgcctattttatactctgacagtaccgccgctgtgtatatagccactaacccagtgtttcacgaacgaacgaaacacatcgaaatcgattgtcacaccgttcgtgagaagctggataatggtcagttgaagctgcttcatgtcaagactaaagatcaggttgctgatatccttactaaaccactcttcccttatcaatttgctcatttattgtccaagatgagtatccaaaacatctttgtattctcatcttgagggggac

>ATCOPIA73_LTR

tattgggaggcattgtatgattttgtaattgttagctggttaagttcggtttagtcaggtttagttggtagagtttacttgtatatatactcacaatgtacaggattttatcaataagagaaaatagaaacttcttcttttacaatctttctctctaaaaccctcttcttcgtctttactatta

>ATCOPIA73_IN

tggtatcagagcaatcacggttctaatcttcttctctccgatctgattttgttcttctcttgctggtttcctccatcttttgagctctttttcgttcaaaatggtgaaggcagctcgagtcactctcaaatcaactcgatccaagtccgttgcgagctccgttgcgagctccattgttcgccgatctactcgatctacctccgatggtaacgtttctcctctaatcgatcatccagcagctatatctcaagtttccggacttccaagaggtgttccatcgccagatctctctgatccaactcagtcacctctgtttatgcacagtgcagaccatccaggtttaaacatcatctctcttcgtcttgaagaaacgaattatgatgattggagctatgcgatgcgtatttctcttgatgctaagaacaaaataggttttgttgatggatccttgcctagaccattgtctatagacccgatgtttagagcatggtcgagatgcaactctatggtgaaatcatggttgttaaatgcagtttctccacagatctatcgtagcatcttgtgaatggatgatgcttctgatatttggtgtgatctctgcggtcgatttcatcttactaacttacctcgcacctacaaccttactcaggagattcaggatcttcgtcagggttctatgactttatctgaatactatactcgtttgaagacattgtggaaccatatggacagctcagaagaacctgatgatccttgtgtttgtgggaaatctgctcgtctgcaactgaaggcagagagagctaagactgtgaagtttttagctggcttgaatgaatcttatgcgattgttcgtcgtcagatcattgctaagaaagttttgccatcactggttgaagtctataatattctggatcaagatgacagtcagaaagtattcactgttgctactccaccctcaacttttcagatctctcaagcaacacctacacatgatgattctgtggttcctgatgtaatgtatgtccaaaatggtcctaacaagggaaggctaatttgttcctactgcaacagggttgatcatattgctgaaaggtgttacaagaaacatggatttcctccgggcttcacacctaaagggaaacccactgataaagctcagaaaccgtctccagtggctgctcaagttaccctgtctgcttcacctgctcctcaaggcaacattgcaggtttacagggaactttatctaatgatcagctccagaatatcattgctcacttcacatctcaactgacaactcaatctcctcttggtgctagcacttctcagtcagctctggatcatactggtatctctttctctaactccacttattcttttgttggcattctggcagtctctcaacacacactgtcacgtaacacatgggtgattgattctggtgctactcaccatgtgtgccatgacagaaatctgtttgtttctctcgactcttctgtggtgaattttgtgaacttgcctactggtcctaaagtgagaattagtggagttggattggttcggttaaataaagatattcttcttcagaatgtcttatttatacctaagtttcgattaaatctcatcagtatcagttcattgacttctgatattggttcacgagtggtttttgatccatcttgttatgaaatacaggatcctatcagggggttgatgaatggtaagggtcgccgtattgggaatctctatgtgttagatacagaatctccatccgtttcagtgaatgcagtggtggacattggtatgtggcacaagagactcgatcatccatctttctcaagactggatgttatatcagaagttcttggaactactcgtcataagaataagaagaatgctttttgtcatacttgtcacttagctaaacagaagaagctgtctttcccttctgaaaacaacatttgtaattcaacttttgagttattacatatagacatttgggggccattttcagtggaaactgttgaaggttttaagtacttcttgactttagttgatgatcactccagagcaacttggatttatctgctaaagaacaaaaatgatgttcttagtatcttccctgctttcatcactcaagttgagaaacaatacaacacaaaggttaaaggtgttagatcagataatgctaaggagttacagttcactcagttttacaaagatcatggaattgtttcttatcactcatgccctgaaactccagagcaaaactcagtggtagagcgtaagcatcaacacattttgaatgttgctcgtgctttattctttgagtccaagatgccattgtctcattggggtgactgtgttctcactgcagtgtttctgatcaaccgaactccttctcaggtactctctaataagactccatatgaagttcccacgggtaaacctccagattacactcgcattcgaacttttggctgtttgtgctatgcttcaacctcacctcagcaacgtcataagtttcagcctaggtcaagagcttgtgtttttctgggatatccttcaggttacaaggggtacaaagtcatggatttggagactaacaaagtctatatctctagacatgttgagtttcatgaggattcctttcctatggcagatttgaataaagaagctgcagtgaacgatgcagataatgatcttcaacagatgaattctttgccctcaggtaattcgatcattcctcatcttccatcacctcaaatttctccatcaacacaaatttcttctcatagagcacgaaaaccacctgctcatttaaatgattatcattgttactctcttgacaatgatgtcacccaacatcccatttcctcttctctttcatatttcaaactctctccatcacgtttgctatacattaatagcattactcaaattccaatccctcaatcttactctgaggcaaagaattcgaaagaattgtgtggggctattgataaggagattggagcaatggaaagtacaaatacttgggagattactgtgttgccaccagggaagaaagttgtgggttgtaaatggatattcactgtgaagttttatgcagatggaactcttgagaggtacaaagccaaaattgttgctaagggttttactcagaaggaaggcttggattataatgaaacattttcacctgtggctaaaatggttactgtgaaaatgcttcttaagatctcagcttctaagaaatggttcttgaatcagttggatatctcaaactcttttctcaatggagacttggatgaagatatctatatgagagttcctgaaggctatgccgagagtaaggggatcattttaccaaagaatgctgtttgcagattaaagaaatctatctgtggtctgaaacaagcttcacgataatggttcctcaagttttcagcttcacttcgtcagcttggcttcattaagtgtcatggtgatcatactctttttgttcgtccttatggttctgagttcattgcagttttggtttatgtggatgatataatgattgctagtactacacaagagggtgctacgcagttaacagaagcattgaagcagagttttatattgcgtgaacttggtcctttgaagtatttcttgggtttggagatagctcgaaatgattcaggtatatccatttgtcagaggaagtatgctcttgaacttcttacttcttcaggcatgcttggatgcaaaccatcttcagttcccatgattccaaatcagaagttacttaagacagatggtgaactgttggaagataaggagttgtatcgcagtcttgttggacggttaatgtatctcacaataactcaccctgacattacttttgctgtgaataagttgtgccagttctcttccgcgcctcgcacttcccacctcacagcagtttacaaggttctgcaatacatcaaaggcacggttggtctaggtctcttctactcttcagatcctgatctcactctcactggatttgcagactcagactgggcttcctgccaagatactagactatcgactactggtttcaccatgtttctaggatcatcactcatctcttggagatccaagaaacaacctgtagtatctcgatcttctgctgaagcagagtatagggcacttgctcttgcttcatgtgaaatgtcatggctggttattcttcttgttggtttgaggattggtccttcatcagtttcggttctcttctcagacagtactgcagccatttacatagcaaccaatccagtcttccacgaacggactaaacacatcgaaatagattgccaaacggttcgggataagctggatgcaggctcattgaagatgcttcatgttcgcactgaagatcaggttgcagacattctaactaaacctcttttccctcagcaattcaatcacttacagtccaagatgagtctcatcaatatattcggctcatcttaagggggca

>ATCOPIA74_LTR

tgtggaagtattgcctaaagtgtcataaagactctaatccaaagagaaggtctcagcttgtgaaagaaacgaagtcacgagaggcgcacgtgctttgtcatacagctcatcaaagctagagacgtaattaacctcttgccaatggttcaaccattttgtctctttgtctctgcatttgaaacagagtacggaagaagagagaggagcgtggcggtgagtagagttgtataaaggagaagataagtgagacattagggttaagagagtaagtgcaagagaggcagctgaaagtttattcagtcttgtttctaagggtttgtgtaaccaaggctttctagtggatttccgggtaatctcggtccagacgtagcgttcatcactgaacgtgaactgggttaacatttgcttgtgtctgttttactttctttgtctaaacacacgagtgatcgaacaaagagagatttagtgagagcttccgcataaaacagttaaagattcaagctttttttttttacatcaaca

>ATCOPIA74_IN

aattggtatcagagccaggttgagtttaaaggttgaagaaaggtttgaactttataaagtttgaagctttggtgataaagcaggttcagacttcataaaggttgaagctttggtgttaaagctttgaactttcttactaatcatgacttctgggcattcggaggtggaaaagttggatggagaaggagattacgtcctttggaaagaaaagttgcttgctcacatagagttgctgggtttgttagaagggctcgaagaagacgaagccattgaggaagaagagtcaacagctgaaactgattctttgctaacaaaaacagaagacaaagtcctaaaggagaagagaggaaaagctagatctacagtcatactgagtctaggaaatcacgttctaaggaaggtcataaaggagaagactgctgcaggtatgatcagagtgttagacaagctgttcatggctaaatctttaccaaataggatttatttaaagcaaaggctttatgggtataagatgtctgatagcatgactattgaagagaacgtgaatgatttctttaagttgatttctgatctggagaatgttaaagtttctgtaccggacgaagaccaagccattgttctgcttatgtcattacctaagcagtttgatcagctcaaagatactttgaagtacggcaaaaccactcttgctcttgatgagatcacaggagcaatcagatctaaggtattagagcttggagccagtggaaaaatgctcaagaacagctctgacgctctgtttgttcaagacagaggaagatctgagaaacgagacaagagtagtgaaagaaacaagagtcaaagcagatcaaagtcaagggaaaagaaggtgtgttgggtttgtggcaaggaaggacattttaagaaacaatgttatgtctggaaagaaaagaacaagaaaggaaacaactcagagaagggagaatcctctaatgtcataggacaagctgcagatgctgctgctttagcagtaagagaagagtcgaatgcagacaatcaagaagttgacaacgagtggataatggacacagggtgctcatttcatatgacaccaagaagggactggtttgttgagtttgatgagtcacaaaccggaagagtgaagatggcaaatcaaacatattctgagatcaaaggcattggaagcataaggattcagaatgatgataatacaaccgttcttctcaagaatgtgagatatgtcccaagtatgtccaagaacttgatctcgatgggaacattagaagatcaaggttgctggttccagtctaaggctgggacattaaaggttgtcaaaggctgcatgacactacttaaaggaaagaaagttggtactttgtacctcttgcaaggagttgttgtaacaggaaatgcaaacgcagttacaagctcaaaagatgaatcaaagatatggcacagcagattatgccatatgagtcagagaaatattgatgtgttgatcaagaaaggatgccttcaagctgaaaagataaatggtctcgagttttgtgaagactgtgtgtacgggaaaacacatagagttggttttgggtctgcaaaacatgtcacaagagagaaacttgagtacattcactcggatctttggggagcgccatcagtaccaaactcactaggtaattgtcagtactttatcacgtttattgatgaccttacaagaaaagtatggatttattttctgaagaagaaagaacatttcttctcttgtatgttgatgatatgttgatcatatcaaagaacaaggaaacagtcaaggaattaaaggaaagactgagttcagagtttgaaatgaaggatttggggcctgcaaggaaaattctgggaatggagataacaagaaaccgagaagaaaatattttggagttatctcagagaagttatctgcaaaaggtgttaaagacattcagaatggatgaatgcaagcctgttaaaacaccattggcaccacatatgaagtttgtagcagcaacagagactgaagcagaggagcaagctgatcaaatgaagtctataccttatgcaaatgcagtgggaagcataatgtattctatgatcgggtcaaggcctgaccttgctcatcctgttggagtcataagtaggtttatgagtaaaccattgatgaatcattggctaggtgtgaaatgggttctccgttacattaaggggtctattgattcaaggctgcagtacaagagagaaggtgattttgtggtgacagggtactgtgattcagatcactctggagatcgtgatcgaagtagatcgaccacagggtacacattcactgtaggagggaacatagtaagctggaggtcatgcttgcagccagttgttgcactgtcatcaacagaggctgagtatatagccttaacagaggctgcaaaagaagcatattggctaaaagacttgatgaatgagctgggatttaaacaaggagcggtagatatccattgtgattctcagagtgctatagctttagcaaagaatgcagttcatcatgaaaggacaaagcatatccagagaaggtttcattacatcagagatgcaatcactgatggagaagtgaaagtgctgaaaatatccacagttcataatccatcggatattttcacaaaggtggtaccggttaacaagttcctgagcgcgttgaagaagctcagggttacaagtgcctgataagggatcatggtccgagatggaaatcaagttacactagaagggaagcaaaggaaaggttgacactaaggcaaggtgaagttt

>ATCOPIA75_LTR

tattagagtagatacgatatatgtaaatcctagatttactagatacgttcttatcttctatgagcaactctccttgtataaatactctcttcgactattggaataaacacacttttacattatatttca

>ATCOPIA75_IN

ttgtatcagagcaaaagctcttttgacctaaatttttttccaccgcagccttcgttgttctgtttcttattctacttctattttctcctattactatacctctactatacgatgtcttcagcatctgccttggtaacgagtacgtcttcatctaacacgtcctccaccactgcatatcttatcaacgcatcagacaacccgggtgctttgatctcttctgttgttttaaaagaaaacaactatgctgaatggtctgaagaactacaaaactttcttagagccaaacaaaaacttggcttcatcgatggatccatccccaaaccggcagctgatcctgaattaagtttgtggatcgccataaattctatgattgttggatggatccgcacatctatcgatccaacaattcgttctacagttggttttgtttcagaggcatcacaactgtgggaaaatcttcgtcgtcgtttttcggttgggaacggtgttcgtaagacactgttaaaagatgaaattgctgcttgtactcaagatggacaaccagttcttgcatactatgggcgtttgattaaactatgggaagaattacaaaactacaagtctggacgcgagtgtaagtgtgaagctgccagtgatatcgagaaagaacgtgaagacgatcgagttcacaagtttcttctcggtttagacagtcgtttcagctccatccgatcttctattactgatatagaacctctacccgatctctatcaagtgtactctcgcgtggttcgtgaagaacagaatctcaacgcttctcgtactaaagacgttgtcaaaacagaggcgattgggttctcagttcaatctagtactacacctcgttttcgtgataagtctactctattttgtacacattgcaatcgcaaaggtcatgaggtcactcaatgctttctggttcatggatatcctgactggtggttagagcaaaatccccaagaaaaccagccttctactcgtggtcgcggctccaatggtcgtggaagcagctccggtcgtggaggtaatcgttcttctgctcccactactcgaggtcgtggtcgtgctaacaacgctcaagcagccgctcccaccgtctccggcgatggcaatgatcaaatagctcagctcatctctctccttcaagctcaacgtcccagcagctcctctgaacgtttgtcgggtaacacttgtcttactgatggggttattgatactggtgcttcccatcatatgacaggggattgttcgattttggttgatgtttttgatatcactccttctccggttaccaaacccgatggcaaagcctcgcaggctacgaaatgtggcacacttctcttgcatgactcttataaacttcacgatgtgttgtttgttcccgattttgattgcactttgatctctgtctctaaattacttaaacagacaagctcaattgcaatctttactgacacattttgtttcttacaggaccgttttttgaggactttgattggggcgggggaagaacgtgagggagtgtattattttaccggtgtattggcacctcgtgtacacaaagcttcgtcggattttgcgatctctggagatttgtggcatcgccgcctaggacatccttctactagtgttttgctttcgttaccggaatgtaatcgttcttcacaaggttttgacaagattgacagttgtgatacttgttttcgttcaaaacaaactcgtgaggtttttcctattagcaataataaaacaatggaatgtttttctctaattcatggtgatgtatggggtccatatcgaactccatctacaacgggtgctgtttactttctcacattagttgatgactattctcgttccgtttggacgtatctcatgtcttctaaaactgaagtctcgcagctcattaagaatttttgtgctatgtctgaacgtcaattcggaaaacaagtcaaagcatttcgtactgacaatggaaccgaattcatgtgtttaacaccctactttcaaacacacggcatacttcatcaaacatcatgtgttgacactccgcaacaaaatggtcgtgtcgaaaggaaacatcgacatatcctgaacgttgctcgggcgtgtctatttcaaggtaaccttccggtcaaattttggggtgaaagtattcttactgctactcatcttatcaatcgcacaccatccgctgtgttaaaaggaaaaactccatatgaactcctctttggcgaaagaccctcatatgatatgcttcgctctttcggttgtttatgctatgctcacattcgcccgcgaaacaaggataaatttacttctagaagtcgcaagtgtgtctttataggctatccccatggcaagaaggcatggcgtgtttacgatctggaaaccggaaaaatatttgcaagtagggatgttcgatttcatgaggatatctatccatatgcgactgctactcaatccaatgttcctctaccccctcctactcctccaatggtgaatgatgactggttcttacctatctccacccaagttgattccactaatgttgactcttcatcctcatcttctcctgcgcaatctggatcgatcgatcagccacctagatcgatcgatcaatcaccttctacgtcgacgaatccagtcccagaggaaattggatcgatcgttccctcatcttctccttctagatcgatcgatcgatccacatctgacttgtcagcttcagatacgactgaattactaagtacaggcgaatcttctactccttcatctccgggtcttcctgagttattgggcaaaggttgtagagaaaagaagaagtctgttcttcttaaagattttgtcacaaacactacatcgaagaagaaaacagcatctcataatatacactctccctcacaagttctaccctctggtctccccacttctctgtccgccgattcggtctctggtaagactctttatcctctctcagattttctgactaactctggttattctgcaaatcatattgcttttatggcagcaatccttgatagcaatgaacctaagcattttaaagatgctattttaattaaagagtggtgtgaagcaatgtctaaggagatagacgcactcgaagctaatcacacatgggatattacagatttacctcatgggaaaaaggctatcagtagtaagtgggtttacaagttgaaatacaattcagatggaacacttgaacgtcacaaagctcgccttgttgttatgggtaatcatcaaaaagaaggagtagacttcaaagaaacgtttgctcctgtggctaaattgactacagttagaaccattttggctgtcgctgctgcaaaagattgggaggtccaccagatggatgttcataatgcatttttacatggcgatcttgaggaagaagtttacatgcgactacctccaggtttcaaatgttccgacccttctaaagtgtgtcgccttcgcaagtccttgtatggtctcaaacaggctccccgttgttggttttctaagttgtcgaccgcacttcgtaacattggtttcactcagagttatgaagactactccctattttctctgaaaaatggcgacacgattattcatgtcctagtctatgttgacgacctcattgttgctggtaataatcttgatgccattgatcgattcaaatcacagcttcacaaatgttttcacatgaaagatctcggtaagcttaagtactttctcggtcttgaagtgtctcgtggtccggatggattttgtctctctcaacgcaaatacgcattggatatcgtcaaagaaactggtctgctaggttgtaagccttccgctgttcctattgctcttaaccacaaacttgcttccataaccggaccggtgtttactaatcccgaacaatatcgtcgcttagttggtcggtttatctaccttacyatcacgagacctgatcttagctatgcggttcacattctttctcagtttatgcaagctccgttggttgctcattgggaggctgcgctccgtctcgttcgttatctcaaaggctcaccggcgcaaggcatttttctccgatcagatagttctcttatcatcaatgcttattgtgactctgattataacgcttgccctctcacacgaagatctctctcggcatatgtcgtttacttgggggactctcctatttcttggaagacaaagaaacaagacacagtctcctactcttcggctgaggccgagtatagggcaatggcatatacgcttaaagaactgaaatggttgaaagctctactcaaggatttgggtgttcatcacagctctcctatgaagttgcactgtgatagcgaggctgccattcatattgcggcaaatcctgtgtttcatgagcgcactaaacacatcgaatcggattgtcataaggttcgtgacgctgttctcgacaaactcatcactactgaacacatttatactgaagaccaggtcgccgatcttcttaccaagtcgctaccaagaccgacctttgaaagactcttgtccacgttaggtgttacggattacgtaccatcaacgtgaggggggg

>ATCOPIA76_LTR

tgtgaagatgaaaccttgatgatcaagctactagtcccatcaagatgatgactcattgggtttaaatgttttactgaggtgaatgtaaatgagaccatgatgaaactgcttttgggaccacagataggggaaacggctacattgacagcaaggagtccgtactcgttcgtcctctggtgctcccaattggttaaagcagtctttgtcttgatttggtgatggcgactcctatgagctgtgctcagcgacaaagtgaaaggagcggctgctttaggtcattgcataggttacctcttgttgctatataagaggcttgttaggtttgagagagtgtagagtgagattgagtgatatagactaaggagagaatacttgtaataagcttaaacttttcttgtattctctaaggctagaaacacatagagtgaacatcaagtgctagtgaagggcattggtgtgcgtcacttattgtttctaggtgtaagttctatattgaacctagtggattccgagtatcatactcgacccagacgtagctacttcggtggtgaactgggttaacaaactctctgtgttcttcttgttatctcacacacaaaaacactcacctattgatctgttttctctgtctctcattcaagctcttatccgtcctttctgttcttggttttgtggggcttactctcaagttaagtcgacagcttgaggtcaagaaatccttaca

>ATCOPIA76_IN

gagtggtatcagagccataggttcattgttctgtggatctctaaagattcaaagatcctgaactatgtctgcagcaagaatagaggttgaaaagtttgatggtcgtggggattacacgatgtggaaagagaagctcatggcgcatctagacattctaggcctgagtgtggctcttaaggaagaagacgatttggtggagaaagttgcagagatgcagcttactgaagaagaagagaaggaagaggtactgagacgagagctcttggaagaaaaaaggagaaaggcgagaagcgccattgtgctgagtgttactgatagagtcttgaggaagattaagaaggagcagtctgcagctgctatgctaggcgtgcttgacaaactgtacatgtctaaggcgctaccaaacagaatctaccaaaaacagaagctttacagcttcaagatgtcagaaaacctcagcatagaaggtaatatagatgagttcttgcgtattatagctgatttagagaacacgaacgtgttagtttctgatgaagaccaagctatattactactcatgtcattacctaagccgtttgatcaacttagagatactctgaagtatgggttaggaagagtcactttatcgttagatgaggttgtagcagctatctactctaaggaactagagttagggtctaacaagaagagtattaagggtcaggctgaaggtctctttgtcaaggaaaagactgagacaagaggaaggactgaacagcgaggcaacaacaacaacaacaagaagtctagatccaaatctagatctaagaagggttgttggatttgcggtgaagagggacacttcaagagttcatgtcctaacaagaacaaaacacaacctcagacaaagacaactaacaacaacaacaaaggtgaatcatcaaacggtagcagtaactactctgaagctaatgggctttatgtttctgaagctttgtcttctacagacatacatcttgaggacgaatgggtcatggacactggctgtagttaccacatgacatacaagcgtgaatggtttgaagatttgaatgaggatgctggtgggtctgtgaggatgggaaacaagactgtttcaaaggtcagaggaataggcacaatccgggttaagaatgaagcaggaatggtggttcgtcttacaaatgtgagatacattccagaaatggataggaatcttttatctctgggaacatttgagaagtctggctacagtttcaagttagaaaatggaacactgagcatcattgcgggagacagtgttctactcacagtaagaaggtgttatacactctacctgttacagtggagaccagtaacagaagagtctctctctgtggtgaagagacaagatgacacaatcttgtggcatcgaagattgggacacatgagtcaaaagaacatggatttattgctgaaaaaaggtcttttggacaagaaaaaagtgtccaagctggagacatgtgaggactgcatatacgggaaagctaagaggattggattcaacttagctcaacatgatacaagagagaagctggagtatgtgcattcggatttatggggagctccatcagtgccattctctctaggtaaatgtcaatacttcatatcgtttattgatgattacactagaaaagttaggatttatttcctgaagactaaggatgaggcatttgataaatttgttgaatgggctaaccttgttgagaaccaaactgacaagaggataaaaactcttagaacagacaatggtcttgagttttgtaacaggtcatttgatgagttctgctcacagaaagggattctatggcatagaacgtgtgcatacacgccacaacagaatggtgttgcagagcgcatgaacaggaccttaatggagaaagtcaggagtatgcttagtgattctggtcttccaaagaagttttgggcagaggctactcatacgacagctatactcatcaacaaaaccccatcatcagctctaaactatgaagtaccagacaagagatggtcagggaagtcaccaatctacagctacttaagaagattcgggtgcattgcgtttgttcacactgacgatggaaagctcaatccgagagctaagaaaggaatactagtaggatatcctattggtgttaagggttacaagatttggttgttagaagagaagaagtgtgtggtgagcagaaatgtgatttttcaagaaaatgcatcttacaaggacatgatgcagagtaaagatgctgagaaagatgaaaacgaggcaccaccaagctcttatttggatttggatcttgatcatgaagaagttatcacctcaggtggagatgatccgattgtcgaagctcagtctccattcaatccaagtccggcaaccactcaaacctacagtgaaggagttaactcagaaactgatataattcagtcaccactgagttatcaactggtgagagatcgagatagaagaacaatcagagctccagtgaggtttgatgatgaagactatctcgctgaagctctctatactacagaagacagtggagaaatagaacctgcagattacagtgaagctaaaagaagcatgaattggaataagtggaaacttgctatgaatgaagaaatggagtcacagatcaaaaaccatacttggacagtggtcaaaagacctcaacatcagaaggttattggtagtaggtggatctacaagtttaaacttgggattcctggagttgaagaaggtagattcaaggcaaggcttgttgccaaagggtatgctcaacgtaaaggaatcgattaccatgagatctttgctcctgttgtgaaacatgtctccattagaatactgatgtctattgttgctcaagaagacttggagctggaacaacttgatgtaaagacagcatttctacacggtgagctgaaagagaagatttacatggtacctcctgaaggttatgaagaaatgtttaaagaagatgaagtttgtcttcttaataagtcactgtatggactcaagcaagctccaaagcaatggaatgagaagtttaatgcttacatgtctgagattggctttataaggagtttgtatgacagttgcgcatacattaaggaattgagtgatggttcaagggtttatctgcttctgtatgtggacgatatgctagtggcagctaagaacaaagaagatatatctcagcttaaagaagaactcagtcagagattcgacatgaaggatttgggggctgctaaaagaatcctcggtatggagattatcagaaatagagaagagaacactctgtggctgtcacagaatggctatctgaataaaattcttgagacttacaacatggcagagtcaaaacatgtggtgacaccacttggagctcacttgaagatgcgagcagccacagttgagaagcaagagcaagacgaggactacatgaagtcaattccctactcaagtgcagtaggaagtatcatgtacgcaatgataggtactcgccctgatctagcttatcctgttggaatcattagtcgctacatgagtcaaccggctagagaacactggcttggagtcaaatgggtcttgaggtacatcaaaggctcactgggaactaagttgcaatacaagagaagcagtgactttaaggttgtgggatactgcgatgctgaccacgctgcatgtaaagatcggagaagatcaattacagggcttgtgtttactcttggaggaagcactatcagttggaaatcaggtcaacagagagttgtagctctctcaactacagaggcagagtacatgtctctaactgaagctgtgaaagaagcagtgtggatgaagggtttgttgaaggaatttggttatgaacaaaagagcgtggagatcttttgtgattctcaaagtgctattgcactctccaagaataatgttcatcatgaaagaacgaagcatatagatgttcgatatcaatatattcgggacataattgctaatggtgatggtgatgtggtgaagattgacactgaaaagaatccagctgatatcttcaccaagatcgtgcctgtaaacaagtttcaggcggctttgaccttgttacaggtcaagcctgagtagtaaaactcaggaggaatccgagtatggaatcctacaactaggttctcggggtactctcttatctctctcattgacagtttgtgcaagtttttggttttatcatcaagtttcaggtggagatt

>ATCOPIA77_LTR

taatagcgtaatagcgtatcttaacgtatcaatgtgatacgctccaatatcaatcacatttattgtgaattgattctgatttgtatctctttaggaaagtagatatcgtctcttacctatataacaatgtaaaggctactgtgaataaggacaagaaactttttctcatacatcgatctctatttgttca

>ATCOPIA77_IN

tggtatcagagccctgacaatttttttttctattgtcttaaacatgtttgctttgaaacgatggccaccccagcaaactctgatgcgaccgaaacgaccacgacccctaacccgatggagacacgacgcaccatctcaccttacgaccttaccgcagccgacaatccaggtgccgtgatctctcatcccttattcaagggatccaactacgatgaatggtcttgcggcatgaaaacagctctctgctctcgcaagaaatttggttttcttgatggctcgattgctaggccagcagaagggtctgcagatcttgacgattggtggacgattcaggccttacttgtgtcctggatcaagatgtccatcgattcatctctccgttcgagtatttcccatcgtgatgttgccaaagatttgtgggacaacttgaagaagagattttctgtgacgaatggtccgcgtattcagcaactgaaggcggagttggcgtgttgcaagcaacgtggtctcgccattgaggcgtactactgaaaactgaaccagatttgggacaacatggctcattatcgtcccgtgcgtgtctgcaaatgtggaaagtgtgactgcgatcttggatttctccaagaacaggaccgtgaaagtgacaaagttcatgagtttctgtctggtcttgactacagttttcgaactgttcgatcttctctggtttcccgtgtgcctatccagtctctggaggaggtttacaatgttgttcgtcaagaggaagatctcaaaactacggtgcatcactatgaagattcctctcaagttgtggctcatgcggttcaaaccaagtcacgtcccatctccgatcgaattgatgctttcgagagagctgtggtttgtaaacattgtaatcgctccggtcatgcctccaacaactgctttgcagtcgtcgggtatcctgaatggtggggtgagagaccacgtagccgtgttgttaatggaagaggcaaaggcgtcacacccggagctgcttctgttggtcgagggcgtcatatcaacgtcaatgctgttcatgtttctcaacaaccacaaacagaacaagcgaattatgttatcactgatgcagaccgtgatggtgtgagtggctttagtgatgtggagtggcgtcgtttgaaggtcttactcaatggtggtgcaagcacgagcacagaaaaactctcgggtaagtctttaacatcctcttggatattagatacgggtgcttctcaccacttaacgggaaattttgatctgctcacaagtgttagagatatggatcctgttttagtcatactagctgatgggagacagaggatttccgtaaaagaaggctatgttgttttggattcccatcttgtgttaaaatcagtttattttgttgaggagtaaacaacggatctgattgttgttggtcagttaatggatgaaaatcgttgtgttgttcaactcgctgatcagtttcttgtggttcaggaccgcgtttcgaggatggtgattggtgcggtaggagagaggctggaacctacttttgcaaaacggagttggcagcatcggtgaggacacaagatgacaagtcatatgagctgtggcatcatcgcatgggacatccgtgtgcacaagttgttggctctcttaagaatgttacagtttcaattcgttctgatattttgaataaagcgtgtgatgtctgtctccgcgccaagcaaactagatctccttttccgattagtatcaataaaactacacaagcttttgagttgattcattctgatttgtggggtccgtaccgaacaatttcgcattgtggagctcgctattttttaacacttgttgatgatttctcaagaagtgtttggattcatcttcttaacgacaaaacagaggctcctacttggataaagaattttattgccatggtagaaacgcagttttcaacaagagtaaagagttttcgcagtgataatggaaccgagttcacaagtttggcaagctattttcgacagcaagggattctttaagagacttcctgtgtgggtacaccacaacaaaattgaagagccgaacgcaaacaccgacacattctgaatgtggcaagagcattgcgctttcaaggacacttaccaatccaattttggggtgagtgtatcctcacagctggttatttaattaatcgtattccttcatcagttctgaatggtctaacaccatatgagaaattgtataagaaagagcccgattatacacatctcaaagtatttgggagcttgtgctatgctcataatcaaggtcataagggagacaagtttgaatcgagaagttgaaagtgtgtgtttgttggctatccatatggcaaaaagggttggaggctatatgattgcgaaacagaggagtttttcatctctcgcgatgtggtgttctgcgaagatcagtttccgtttgataagtcatcttcatcatcggcttctcctgtacacgaagaagaagaagagttatgggcttcattcagtatgaacccattagctgaaattgaagataggcccgataagggcaaaagcccactaactgattcaagtccatcttcattatccagcccagatacaaacattgcttcttcatcctctacgagtctctcgacgcctccgtccacttcctctgattcatcttcgtcaccaccgacctccacaactcagtctgacaattccgatcaaggattgactaccctcgaagctacgtctaactccacgaatcctgtgccaccatcatcaaatctgttgggtcgaggaaagagaccgaaaattccatctgtcagactcaacaattatgtggttaatacggcacatggaaaattgctcaacaagtctggtaaaacccaatatcccattgcaaactatgtctcttgtacacgtttttcagagacacatcgtgtttatctagcggctatcactgagaatattgagcccaaatcattcaagtcagctatggagaatgaacgatggaagaaagctatgggcacagaggtgggtgcttcagaagaaaacgaaacatggacattagagaatcttccacctgggaaacgagccattgggagcaaatgggtctacaagatcaagtacaattcggacggcacgattgaacgttacaaggcaaggttggttgctcttggtaacaaacaaattgaaggtgaaggctatggagaaacgtttgctccagtagctaaaatgggtacgattcgtttgtttttaaaggttgcagctgggaatgattggccggtttatcagatggacgtttacaacgcctttcttcacggagatcttgaagaagaggtgtatatgaaaccaccacctggtttttatccaaaagatgagcagaaagtttgtcggttaagaaagttaatatatggccttaaacaagctccgaggtgttggttcgaaaaattcacatcatctcttcgtgactatgggtttcagcaaacacatgctgattattcgttgttcacctttgatagagatggtattcagattcgcttgctcatatacgtcgacgacatgattcttaccggtaacaatgacgcatcattggaagaatttaaggtctatctctcctcttgcttcaagatgaaagatcttggtcctttaaaatactttctgggtatagaggtctctcgcaacaaatctgggttctatctgagccaacgaaaatacgctttggatattgtcaccgaaaccgacatgttagcctccaagcctgcctcatttccgttggaacaaaatcaccaacttgctttatcaacgtcacctctgttggtcgatccgtctccatatcgccgcttaataggtcggtttatctacttggcaaccactcgacctgatttagctttttgtgttcatactctggcgcagtttatgcaacagccgcgtgaagatcattggcacacagctttgcatgttgttcgctacattaaaggtaccgctggtcaaggaatcctcttgagttctgccaatgattttaaggttaatgggtggtgtgattccgactggtcgagctgtcctattactcgtcgttcagtgacggggtattttgttcagttgggacaatctccaatatcatggaagacgaagaaacaagacacggtgagcaagtaatccgcggaggctgaatacagagcaatatctcatcttcgagatgaactattatggataaagaaggtacttctatcaatgggtgttcgtcatgatcaaccgatgaacattttctgtgatagcaaagcagcgatctacataagtactaatccagtctttcatgaacgtaccaagcacatcgagaatgactgtcatgttgttcgtgtgaaatacaaaaaggaaccatgtctccacatcatgtttccacaactcaacagctagccgacatttttacgaaaccgttaggacgacaagcatatgagctatttcgcagcaagctgggcattcttgatctccacgcaccagcttgggaggggg

>ATCOPIA78_LTR

tgttgaaagttaaacttgattttgaatcaagtttaattattggatcaattatccaataattaattatggccaaatccaagttctagagttttctctagaaatatcatcatttccacctccttaaaagattctagaaattttctagaatcatcttccacctccttaaacataaaaatctagatactctaatagaataatctagataatttgaataatgtaatctagatcttatgtaagaactctctagacttaggattaaaatattttagatattttgtagtttggaggctataaatacctcctccccctctcaaatgttgcaatgttgtgaagttgtattcaagtttaaagcaaagtaataaaagttctatttcctaaaaaactctctcaaaacacttaaacactttctccattacctctaaaagaattttactctaaca

>ATCOPIA78_IN

aaagtggtatcagagcttgaagatcctaaagatggcgagtaacaatgttcccttccaagtcccggtgctcacaaagagcaactatgataattggagtctacgaatgaaggctatcctaggagcacatgacgtgtgggagatagtcgagaaaggtttcattgaaccggagaatgaaggtagtctttctcaaactcaaaaagatggtttgagagactcaagaaagagagacaagaaagctctctgtctaatctatcaaggattagatgaagatacgttcgagaaggtcgttgaagctacgtcggcgaaagaagcatgggagaagcttcgaacctcttacaaaggtgccgatcaagtcaagaaagtacgtcttcaaactctaagaggagaatttgaagcactacaaatgaaggaaggtgaactcgtctccgattacttctcaagagtgttgacggttactaataaccttaaaagaaacggagagaagctagatgatgtgagaatcatggagaaagttcttagatcattggatctaaaatttgagcatattgtcaccgtcattgaagaaacaaaagatttagaagctatgacaatagagcaacttcttgggtcattacaagcttatgaagaaaagaagaagaagaaagaagatatcgtcgaacaagtcctcaatatgcaaattacaaaagaagaaaacggccaaagttaccaaagaagaggtggtggtcaagtacgaggacgaggtcgtggtggatatggaaatggacgtggttggaggccacatgaagacaacacaaaccaaagaggtgaaaactcatcaagaggtcgtgggaaaggacacccaaaatcaagatacgataaatcaagtgtcaaatgctacaattgtgggaagtttggacattatgcttctgaatgtaaagctcctagcaacaaaaaatttgaggagaaggccaactacgttgaagaaaaaattcaagaagaagacatgttattaatggctagctacaagaaagatgaacaagaagagaatcataagtggtacctcgatagtggtgcaagtaatcacatgtgcgggagaaaaagtatgttcgcggagcttgatgaatcggtgagaggaaatgtggctttaggagatgaatcgaagatggaggtaaaaggtaaaggaaacattctcattcgattgaagaatggagatcatcaatttatttccaacgtttactatattccgagcatgaagacaaacatcttgagccttggacaactcttagagaaaggttatgatattagattaaaagataataacctttcaataagagaccaagaaagcaatctcattaccaaggtgccaatgtcgaaaaatagaatgtttgtcctcaacattcgaaatgacattgcacaatgtcttaagatgtgttacaaagaggagtcttggctatggcatcttcgattcggacatctaaattttggaggattggagttgctttcaaggaaggaaatggtgagagggctaccttgtataaatcatccaaatcaagtgtgtgaaggatgtctacttggaaagcaattcaaaatgagctttccaaaggagtcaagttcaagagcacaaaaaccgttggagctaatacacaccgatgtgtgtggtccgatcaagccgaaatcacttggtaaaagtaattacttccttctctttattgatgatttttcaagaaaaacatgggtatattttttgaaagaaaaatccgaggtgttcgaaattttcaaaaagtttaaagcccatgttgagaaggagagtggtcttgtgatcaaaaccatgagatccgaccgtggaggagaatttacatccaaggagtttcttaagtattgtgaagacaacggcattcgaagacaattaacggtgccaagatcccctcaacaaaatggtgtagcggaaagaaagaatagaacaattcttgagatggcaaggagcatgctcaaaagtaagagactaccaaaagagttgtgggcggaagcggtcgcgtgtgcggtttatctattaaatcgatctccaacaaaaagtgtctccggaaaaacaccacaagaagcttggagcggaagaaagcccggtgtttctcatttaagagtctttggaagtattgctcatgctcatgtaccggatgagaagcggagcaaactagatgacaaaagtgagaagtatatcttcattggttatgataacaactccaaaggctacaagctctataatcccgatacgaagaagacaattattagtcgaaatatagtgttcgatgaagaaggagaatgggattggaactcaaatgaagaagattataacttctttccacattttgaagaagatgagccggagccaacaagagaggagccaccaagtgaagagcctactacaccaccaacttcaccaacaagttctcaaatagaagaaagttcgagtgaaaggactccgcgttttagaagtatacaagagctctatgaggtaaccgaaaatcaagaaaaccttaccttattttgtttatttgcggagtgcgaacccatggatttccaagaagccattgaaaagaagacttggagaaatgccatggatgaagagatcaaatcaatacaaaagaatgacacatgggagttaacttcacttccaaatggacacaaggcaattggcgtgaagtgggtgtataaagcaaagaaaaactctaaaggagaagtggaaagatacaaagcaagattggttgcaaaaggttatagtcaaagagccggaattgactatgacgaggtatttgctcccgttgctcgtctagaaacggttagactaatcatctcactagcggctcaaaacaagtggaagatacatcaaatggatgtcaagtcggccttcttaaatggagatcttgaagaagaagtttacattgagcaaccacaaggctacatagtcaaaggtgaagaagacaaagtcttgaggctaaaaaaggcgctttatggattaaaacaagccccaagagcttggaatactcgaattgacaagtatttcaaggagaaagatttcatcaagtgtccatatgagcatgcactctatatcaaaattcaaaaagaagatatattgatcgcatgcttatatgtagatgacttgatattcacgggtaacaatccaagcatgttcgaagaattcaagaaagagatgacgaaggagttcgagatgacggacattggattgatgtcttactatctcggaattgaagtaaaacaagaagacaatggaatattcataactcaagaaggctatgctaaggaggtacttaagaagttcaagatggatgactcaaatcccgtttgtacaccaatggaatgcggaatcaaactatcaaagaaagaagaaggggaaggagtggatccaacaacctttaagagcttggttggaagcttgagatacttaacatgcacaaggcccgatattttatatgcggtcggagttgttagtcgttacatggagcatccaacaacaactcatttcaaagcggcaaaaaggattcttcgctatatcaaaggtaccgtaaactttggcttacattattcaactactagtgattacaagcttgttggatatagcgatagcgattggggtggagacgtagatgaccgaaagagtacaagtggttttgtgttttacattggagacacggctttcacatggatgtcgaagaaacaaccaattgtcactctatccacttgtgaagcggagtatgtagcggctacgtcatgtgtatgccatgctatttggttaagaaacctcttgaaggagttaagcttaccacaagaggaaccaacgaagatctttgtggacaacaagtcggcaatagctttggcgaagaacccggtcttccatgatcgaagtaaacacattgacacacgctatcactacattagagagtgtgttagcaagaaggacgtgcaattggagtatgtgaagacacatgatcaagtagccgatatttttaccaagcctctcaagcgtgaagactttatcaagatgaggagtttgcttggagtagcaaaatcaagtttaagaggggg

>ATCOPIA79_LTR

tattagcaagagctctactgctccgccttcagctcctgaatctgaaacaagatcgaagaagaactcagatgcagctgaaagtcaaggagtcaagacgatgtcgttgcagtaaacaaagagaaagggatattgggccacttacctaacaagtccaatatcaacttaaatgagaagcccagcaagtctgagaagcccaacaagtcttcttcatcttctcaacggctaagtccacctgtgcaagagaacaaagccagaataatgacagagaagaggagcgcttgttgtacggctccaatcatcacagagaacaaatttgattgcttgatgacatgcgtagcataacaagattgagacatttgtcagttcacaatgattgaaaccctagcattgtttagctataaaagggaacttgtgatgtaacctagaactcaagtgaaataatatcaaaaggaaagttcattcaaacattacctaaagtagccgtcttgctcttactttca

>ATCOPIA79_IN

tggtatcagagcacagcggtctatgatggatcaatctatccaactctactcacctcctgttcttcacatctccaactgtgtcactgttaagctcaatgagcaaaactactctcttaagaagacacagtttgagtcctttctttctggccaaaaccttctaggctttgtcaatgggtcactcaagcctcctcctgcaacaactccattcaacaacatcgatggtcttactattgaggtaccaaatccagagtatcatacctggaacagatctgaccaagtagtccgagcctggctgttgggatctctgaatgaagacatcatgcaggaagttgtcaactgtgccacctcctatcaagtctggaatgctttggctcaacactgcaataaggtatcttcatcacgactctttgagctacagagaaagcttcaaaccatagataaacaggataaatccatggaaacttaccttaaggagataaagagggtttgtgaacagctagcttccataggcagtcctgttagtgaacagatgaagatatttgctgcacttcatgggttaggtagagagtatgagcctatcaaaacatctgtagaaggttcaatggatactcagccaccacctacctttgagagtgtgatttcaaggctgacaggatttgctgataggctgaacagctatggtcctgatactgaaacttcacctcacatggctttcaatgcttcaagatcagactcctctggttactacaacaacaacaacaaccgaggaaaaggcaactaaagatttgggagtggaaaaagcaaagattcattctccacacgtggtagaggttttcatcaacaaatttcacaaaactcaaatggagaaagagtcatttgtcagatctgtggcaaacctggtcatcatgctttgaagtgttggcatcggttcaacaacagctaccaagatgaggatctgccagcagctcttgcaatactcaggataactgatgtcacagatcaatccggtggtgaatgggtaggtgactctggctctactgctcacatcaccaatgcacctcacaagctcagtcagactcagctgtatgaaggaagtgactcagtaatggtgggaaatggaaactttcttcccataacacacacatgattaggcagtcttccttctacttcaggtaacttacctttaaatgatgttcttgtgtgtcctgatatctctaaaccctttttgtcagtttccaagctcaccaatgactatccctgcgtatttcaatttgactgtgatgatgttcgaatctatgataaggcaacaaggaaactgttgacaaagggaaagcatagtaagggactttatgtgttgaaggactcaccagttcaagcactctactcttcaagacagcaaggagcgagtgaagatatatggcatcaacgactggggcatcctcactctcaagtccttcaagtgctatctgcaaacaaatctatcagtgttggaaataaaagtaccaagatggtgtgtgagtcttgccagcttgggaaaagtgttagactccctttttctgcttcaactttttgtagctactagacctctagagagaatacactgtgatctctggggtccttgacctattaaatcaaatcaaggattcagttattatgcagtgttcattgataactactcaagattttgctggttctacccattaaagctcaaatcagactttactcatacctttactgtgttccaagatatggttgaaaatcaatatcaaaccaagattggatctttccagtgtgatggagggggagagttcacaagcagaaagctaactgatcacctacaacaatgcgggatcaaacagcttatctcttgtccctacacttcacaacagaatgggttagctgaaagaaaacatagacacattattgaacttggtctctcaatgatgtttcagagtacccgtcctcagaagttttgggtagaagccttctacactgcgagtttcttaatcaatctccttcctacaacagctctagatgagaagtacagtccttatgagaagctgcatggtaagcctccagaatactcagcactcagagtctttggttgtgcctgctatcccaccttgagagattatgcgtctaacaagtttgatcctagatcactcaagtgtgtgtttctgggttataatgataaatacaaggggtacaggtgcttcttaccttcaactggaagggtttacatcagtcgccatgtcatctttgatgaaactgtcttcccttttgctcagtctcattcaagaccaaattcaggaagtcttactcctttgatgctagcctggacaaagggaatcaacacagtaagtaaacctcaacctaaatcagatgacagtttattcacaagtgaagattttcctccactacctacaagagaaacacctatactaccaataccaataccaagaccgactgtagtacctgctgtggaagaggaaagaagttatgggtgtacggcgggcttagatcatgttcctataggcaacaacttctcttcttcttctcacagtcctggtattgcagaagaaacgtcagatcagtctacagaaagaatattagatcagttatcaacaacaacaactcaaaatgaaagtcaagaagaaatgttacctagttaagaacctgtgcaagagcctgttgtaactcagtctactcaccctatgaccacaagacaaaagagtgggatcagaaagccaaacccaagatatgctttactgactcacaaagtgtcttaccctgaacctaagacagttgctgcagctctaaaagacccaggatggacaggtgccatgggagaagaaatgggaaattgcaaagaagctgaaacttggtctctggttccttatacaccagacatgcttgttttaggcagcaaatggattttcaggaccaaactaaatgcagatggatccctgcaaaagctgaaagcaaggttagtaacacaaggttataatcaagctgaagggatagattacttagaaacttacagtccagtcgtcagaacagctactgtgagaggagttctacatcttgcaacgattatggagtgggatatcaagcagatggacgtgcaaaatgcatttctccatggtgacttaacagaaacagtctatatggctcaacctgcaggttttgtagatccagacaaaccaaattatgtctgtcatcttcataaatctttgtacgggatgaaacagtcacctagagcatggtttgacaagttcagtacatacctcttggaatttgggtttcactgcagcattccagatccatctctattcgtctacagcagaggaaaagacatcattcttcttctcctgtatgtcaatgatatgctgattactggaaacagctcagaaacacttgcctctctccttgcagaactcaacaaaagattcaagatgaaagatatgggtcagatgcattatttcttgggaattcaagctcaatttcattctgaaggtctattcctatctcagcaaaagtatgcagaggacttacccattgcagcatcgatgagtgactgtgccccaatgccgactcctctgcttcttcagctaaacaaacaaaggaaacagaatcaggataccttttaaaacccaacatatttccgcagccttgctggtaagctccaatatttgactcttaccagacctgatattcagtttgcagtgaactatgtttaccaaaagatgcatgctcctactactctggatttcttgttgctgaaaaggattttaagatatgttaagggtacagttactatgggaataaacttcaggaagaagtcagattgcacattacgagcttatagtgacagtgactggtctggttgtcctgaaacaagacgatcaaccggaggctacttcacttatctgggtctgaaccttatctcctggtcttctcagaagcagtcgtctgtgtcaaagagctccactgaagcagaatacagaactctctctgaagcagcttctgaaatcacatggctcagctcaattatgaaggaattgcgagtaccccttctaaaaccaccacaactatactgcgacaatctctctgcggtctacctaacagcaaatccagcgtttcacaagagaacaaagcatttcgagaaccactatcactatgtgagagaaagagtggctcttggactgttggaagtcagacacatacctggtcatgagcagatcgcagacatcttcactaaatcgcttccgttcaatgcgtttacatctctcaggtacaaactcggtgtagttgtgccacccacaccgggtttgcaggggga

>ATCOPIA7_IN

tggtatcagagcttaggactttgttcctaagccatggaagaatctatttttgtttcctctcttaacatctctcaagttgttactctcaaactcactcccacaaactaccttttgtggaaaacccaatttgagtcttacctttcatctcatcttcttctaggatttgttaccggcgccactcctcggcctgcctccaccatcattgtcacaaaggatgatattcagtctgaagaagcaaatcaagaatttctgaaatggactcgaatcgatcagttggtcaaagcttggatttttggatctctctctgaagaagctctcaaagttgttattggcttaaactctgctcaagaggtatggcttggtttagcaagacgttttaatcgtttttccaccactaggaaatatgatctgcagaaacgactaggcacttgttcaaaagctggaaaaacgatggatgcttatctaagtgaggttaaaaacatatgtgaccagcttgattcaattggctttccggttacagaacaagagaagatttttggtgttctgaatgggctaggaaaggagtatgagtccatagcaactgtaatagaacactctcttgatgtgtatccaggtccgtgttttgacgatgtcgtgtacaagctcactacgtttgatgataaactctccacttatactgcaaattccgaagtcactccacatctggccttttacacggacaaaagctattcttcacggggtaataacaatagccgtggtggaagatatggtaattttcgcggtcgtggttcctattcttcgcgtggtcgtggttttcatcaacagtttggttctggttcaaacaatggctctggaaatggttcaaaacccacatgtcaaatttgcaggaaatatggtcactcagcctttaagtgttacacacggtttgaagaaaactatcttcctgaagatcttcctaatgcatttgctgctatgcgggtttccgatcaaaatcaagcttcaagtcacgaatggcttcccgactcggctgctactgcgcatatcacgaacacaaccgatggccttcaaaactctcagacatactcaggtgatgattctgtgatagtagggaatggagattttctgcccatcactcatattggtactatccctctgaacatttctcaaggtacattgcctttagaggatgtcttagtatgtcctggaataactaaatcgcttttatctgtttccaagcttactgatgattatccctgctcttttacgtttgactctgattctgttgttataaaggacaaacgaacacaacaactcctcacacaaggaaacaagcataaaggtctctacgtgttgaaggatgtcccgtttcaaacttattactctaccaggcaacaatctagtgatgatgaagtgtggcatcagagattgggtcaccctaataaagaagttctacaacaccttatcaaaactaaagctattgtagtcaataaaacaagctccaatatgtgtgaggcttgtcaaatggggaaagtatgtcgtcttccttttgttgcttctgaatttgtatcttctcggcccctagaacgtattcattgtgatctatggggccctgcccctgttacttctgctcaaggttttcaatattatgtcatctttatcgacaattattctcgattcacatggttctatccattgaagttgaagtctgattttttctcggtctttgttttgtttcaacaacttgtcgagaatcagtaccaacataagattgctatgtttcagtgtgatggtgggggtgagttcgtctcatataaatttgttgctcatcttgcttcatgtggtattaaacaattgatttcatgtccacatactccgcagcagaacgggattgctgaaagacgacatcggtacttgactgaacttggcctttctttgatgtttcatagcaaggttcctcataagctatgggttgaagcattctttacttcaaactttttgagcaatcttctgccttcttctactttgtctgataataagagtccctatgagatgttgcatggaacaccacctgtttacacagccctgcgtgtttttgggagtgcttgctacccctaccttcgtccctatgcgaaaaacaagtttgatccaaagtctctcctctgtgtgtttttgggatataacaacaaatacaagggatatcggtgtctccatccacctacgggcaaagtctacatctgtcgacatgttttgtttgatgaaagaaaatttccctactccgacatttattcacagtttcaaacgatttcgggttctcctctgttcactgcttggcaaaaagggttttcctctaccgctttatcacgtgaaactccatctacaaacgtggaagacatcatctttccttctgcgaccgtctctagctccgtaccaaccggttgtgctcccaatatagccgaaacagctacagctcctgatgttgatgtggctgctgctcatgatatggttgttcctcctagcccgatcacatccacatcgcttcccactcagcctgaggagtccacttctgatcaaaatcactactctacagattctgaaactgctattagctcagccatgactcctcaaagcattaacgtctctttgtttgaggactctgactttcctcctctgcaatcggtgatctcgtcgactactgctgctcccgaaacttcccatcctatgataactcgagctaaatcaggtatcacgaagcccaatcctaaatatgctctgttctctgtcaagtctaattatcctgagccaaaatctgtcaaagaagctctcaaagatgaaggctggacaaacgcaatgggagaagaaatgggaactatgcacgaaactgatacatgggacttagttcccccagaaatggttgatcgtcttcttggatgcaagtgggtgtttaaaacgaaactaaattctgatggtagtttggatcgtttgaaagctcgtcttgttgcaagagggtacgagcaagaggaaggtgtggattacgttgagacatacagcccggttgtcagatctgccaccgttcgatccattcttcatgtcgctacaattaacaaatggtctttgaagcaacttgatgtgaaaaatgctttccttcatgatgagctaaaagaaactgtgtttatgacccaaccccctggttttgaagatccttctcgaccggattatgtatgtaagctcaagaaagcaatttacgacttaaaacaggctcctagagcttggttcgataaattcagctcgtatcttctgaagtatggatttatctgcagtttttctgatccatctctctttgtttatctaaaaggcagagacgttatgttcttgttactatacgtcgatgatatgattttgaccggtaataacgatgttctccttcaacaacttctgaatattctcagtaccgaatttcgaatgaaggacatgggagcattacattattttttggggattcaagctcattatcacaacgatggtctcttccttagtcaggaaaagtatacctctgatttattggttaatgcaggcatgtctgactgctcatccatgccgacacctcttcagcttgatctattacaaggtaacaacaaaccttttcctgaacccacttattttaggcgcttggcgggaaagctacaatatcttaccctcactagacccgacatccagtttgccgttaacttcgtttgtcaaaagatgcacgctcctacaatgtctgatttccatttactcaagaggatcttgcactatcttaaaggtacaatgacaatgggaattaacttatcttccaacacagattctgttcttcgttgttatagtgatagcgattgggctggctgcaaagacacaagacgatctaccggaggtttctgcacattccttggctataacataatctcgtggtctgctaaacgtcatcccactgtctccaagtcctccactgaggctgaataccgaacattgtctttcgctgcctctgaagtgtcatggattggtttcttgcttcaagaaattggacttccacaacaacaaatcccggagatgtattgtgacaacctttcagccgtatatctatctgctaatccagctcttcactctcggtccaaacattttcaggtggactattattatgttcgagaaagggtggctttaggcgctctcaccgtcaaacacattccagcttctcaacaacttgctgacatcttcactaagtctcttcctcaggcaccgttctgtgatcttcggttcaaacttggtgtcgtcttgccgcctgacacaagtttgagggggtg

>ATCOPIA7_LTR

tatcaaacaacaagagactccttctcctacagagacttcaatccatcgcaacaacattgtttctgtttcagtttcagttaaaggagattcaggcccaaaagaaaaagagaagcccacgaagcccatcctttactcatcgaaactctgcaacaatcaaagcagaggaacatcacgcgagacaccgccgtcgacaaacataaacgtgcacaatcattttgatgttcttggaagcagcagcatttcatgcaactaggacacctgtcattagctattatgtaatcctcctctttatatagttctctgcttgtaatcgaattcactaaggaagaaatacacaattattttctctctgaagcttttgcttctactttca

>ATCOPIA80_LTR

tataagagtattaagtaaatacttagttagggatatcaatgtaaattatgaaagttaactctaccctaattctctcatatgttctgtataaatagatgtaaaccatattctttgattaataagattatttcaatccttgta

>ATCOPIA80_IN
[truncated: 358,821 more chars]
