# Supplementary material for: LTRtype, an Efficient Tool to Characterize Structurally Complex LTR Retrotransposons and Nested Insertions on Genomes
Source: Front Plant Sci. 2017 Apr 4;8:402. doi: 10.3389/fpls.2017.00402 (PMC5379124; doi:10.3389/fpls.2017.00402)
Supplement: Supplementary file 3 [file Data_Sheet_3.docx]

**User guide for output files**:

Library.fasta.nhr/nin/nsq, blast.out and formatdb.log are the blast results between Library.fasta and Library.fasta.

Genome.fasta.alert/cat/masker/ori.out/out/ref/tbl gives the results after running RepeatMasker of Genome.fasta and Library.fasta.

blastpair: the pairs of LTR or IN from Libray.fasta; they are considered as the same family in LTRtype.

Lib_len: the length of each element (LTR or IN) in Library.fasta.

RM.out.org: Genome.fasta.out formats the remaining candidate fragments after removing redundant fragments.

RMclear_out: Genome.fasta.out formats the removal of redundant fragments.

RM.out: final candidate fragments after the defragmentation.

Output loopx folder:

I.x: detailed information on I structural types of LTR retrotransposons.

I-L: detailed information on I-L structural types of LTR retrotransposons.

I-L-I: detailed information on I-L-I structural types of LTR retrotransposons.

I-L-I-L: detailed information on I-L-I-L structural types of LTR retrotransposons.

L.x: detailed information on L structural types of LTR retrotransposons.

L-I.x: detailed information on L-I structural types of LTR retrotransposons.

L-I-L.x: detailed information on L-I-L structural types of LTR retrotransposons.

L-I-L-I.x: detailed information on L-I-L-I structural types of LTR retrotransposons.

L-I-L-I-L.x: detailed information on L-I-L-I-L structural types of LTR retrotransposons.

Other.x: detailed information on other structural types of LTR retrotransposons.

I.RM.x: candidate fragments to form I structural type of LTR retrotransposons.

I-L.RM.x: candidate fragments to form I-L structural type of LTR retrotransposons.

I-L-I.RM.x: candidate fragments to form I-L-I structural type of LTR retrotransposons.

I-L-I-L.RM.x: candidate fragments to form I-L-I-L structural type of LTR retrotransposons.

L.RM.x: candidate fragments to form L structural type of LTR retrotransposons.

L-I.RM.x: candidate fragments to form L-I structural type of LTR retrotransposons.

L-I-L.RM.x: candidate fragments to form L-I-L structural type of LTR retrotransposons.

L-I-L-I.RM.x: candidate fragments to form L-I-L-I structural type of LTR retrotransposons.

L-I-L-I-L.RM.x: candidate fragments to form L-I-L-I-L structural type of LTR retrotransposons.

other.RM.x: candidate fragments to form other structural types of LTR retrotransposons.

link.x: the link list of candidate fragments.

link.index.x: the index of link list.

RM.out.x: the x layer of the candidate fragments.

type.all.x: detailed information on all structural types of LTR retrotransposons.

type.insert.x: the x layer of the insertion fragments.

type.num.x: copy number of all structural types of LTR retrotransposons.

type.out.x: the identification of different types of candidate fragments.
